# Computational STAT3 activity inference reveals its roles in the pancreatic tumor microenvironment

**DOI:** 10.1038/s41598-019-54791-x

**Published:** 2019-12-03

**Authors:** Evelien Schaafsma, Yiwei Yuan, Yanding Zhao, Chao Cheng

**Affiliations:** 1 Department of Molecular and Systems Biology, Geisel School of Medicine at Dartmouth, Hanover, NH 03755 USA; 2 Department of Biomedical Data Science, Geisel School of Medicine at Dartmouth, Lebanon, NH 03756 USA; 3 Department of Medicine, Baylor College of Medicine, Houston, TX 77030 USA; 4 The Institute for Clinical and Translational Research, Baylor College of Medicine, Houston, TX 77030 USA

**Keywords:** Pancreatic cancer, Predictive medicine

## Abstract

Transcription factor (TF) STAT3 contributes to pancreatic cancer progression through its regulatory roles in both tumor cells and the tumor microenvironment (TME). In this study, we performed a systematic analysis of all TFs in patient-derived gene expression datasets and confirmed STAT3 as a critical regulator in the pancreatic TME. Importantly, we developed a novel framework that is based on TF target gene expression to distinguish between environmental- and tumor-specific STAT3 activities in gene expression studies. Using this framework, our results novelly showed that compartment-specific STAT3 activities, but not STAT3 mRNA, have prognostications towards clinical values within pancreatic cancer datasets. In addition, high TME-derived STAT3 activity correlates with an immunosuppressive TME in pancreatic cancer, characterized by CD4 T cell and monocyte infiltration and high copy number variation burden. Where environmental-STAT3 seemed to play a dominant role at primary pancreatic sites, tumor-specific STAT3 seemed dominant at metastatic sites where its high activity persisted. In conclusion, by combining compartment-specific inference with other tumor characteristics, including copy number variation and immune-related gene expression, we demonstrate our method’s utility as a tool to generate novel hypotheses about TFs in tumor biology.

## Introduction

Pancreatic cancer accounts for 3.2% of new cases but 7.5% of cancer deaths in the United States according to the 2019 Cancer statistics, estimated by the American Cancer Society^1^. The overall five-year survival rate of pancreatic cancer after diagnosis is approximately 9%, making it the cancer type with the worst prognosis^1^. Such a decimal survival rate is caused by many different factors, including a high proportion of late stage tumors at the time of diagnosis, poor resectability, and minimal durable response rates to conventional chemo- and/or radiotherapy^2^.

Recently, T-cell infiltration in the tumor environment has also been identified as a prognostic factor^3–6^. To therapeutically modify the immune milieu of cancer tissues, immune checkpoint blockade therapies have been used. Such therapies have experienced progress in the treatment of melanoma and lung cancer^7–9^, but have had lackluster success in treating pancreatic cancer^7,10,11^. This is likely due to the highly immunosuppressive environment of pancreatic cancer, which is characterized by extensive fibrosis and chronic inflammation^12–14^.

Several components that contribute to this immunosuppressive environment have been identified, including transcription factor (TF) STAT3^15^. STAT3 is activated by a variety of extracellular stimuli, including interleukin (IL) −6^16^, IL-10, and epidermal growth factor (EGF); it can also be activated by intracellular stimuli, such as SRC and ABL^17^. Its role in pancreatic cancer is complex due to the diversity of cells that express STAT3. For example, STAT3 activity inhibits the chemotaxis and activation of cytotoxic CD8 T cells in solid tumors^18,19^, mediates the differentiation of suppressive T regulatory (T_reg_) cells and enhances the expression of immune checkpoints CTLA-4^20^ and PD-L1^21,22^. STAT3 is also active in and required for the presence of suppressive myeloid cells, including the prevalent myeloid-derived suppressor cell (MDSC) population^23^ and profibrotic M2 macrophages^24,25^. In addition, cancer-associated fibroblasts (CAFs) use STAT3 activity to secrete cytokines that recruit additional immune cells and promote STAT3 activity in other cell types in the TME^26–28^. In turn, STAT3 is also active in tumor cells^29,30^. Importantly, STAT3 is required for the evolution of pancreatic neoplasia into pancreatic cancer in the presence of KRAS mutations^31–33^.

The aforementioned insights into the role of STAT3 in pancreatic cancer have mostly come from *in vitro* studies and animal models, which bear a resemblance to patient tumors but cannot fully recapitulate all pancreatic cancer features. In addition, the use of patient-based tissue arrays or immunohistochemistry often preclude the use of large sample sizes. Since TF expression generally does not correlate with activity^34,35^, the use of larger-scale patient-derived gene expression studies to investigate STAT3 has been limited. Models for TF activity inference from gene expression studies have been proposed^36–39^, but current models do not support a distinction between TME-derived and tumor-derived TF activity signals. Since STAT3 is active in several cell types in the TME as well as in tumor cells, being able to make a distinction between TME- and tumor-specific STAT3 activity is crucial. Therefore, we sought to develop a method that can distinguish between TF activities in the tumor and TME compartment to better characterize the multifaceted role of STAT3 in pancreatic cancer using a collection of gene expression datasets.

Our framework relies on the expression pattern of TF target genes to create compartment-specific TF profiles that can be used for TF activity inference. After validating STAT3 as a TME-expressed TF, we show that STAT3 activity is prognostic, whereas STAT3 mRNA is not. We also show that biological insights can be obtained utilizing TME- and tumor-specific STAT3 activity inferences. For example, environmental-STAT3 plays dominant roles in establishing or maintaining an immunosuppressive TME and is associated with tumor intrinsic and extrinsic factors, such as immune infiltration and copy number variation (CNV) burden. In addition, while environmental-STAT3 is most influential at the primary site, tumor-derived STAT3 seems to be dominant at metastatic sites where its activity persists. Thus, using our approach, we can distinguish between tumor- and TME-specific TF activity to obtain more insights into the role of TFs in disease using gene expression datasets.

## Results

### Overview of this study

**Figure 1:**
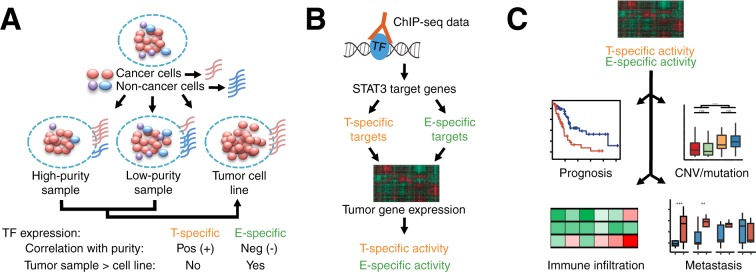
Workflow of analysis. (**A**) Cartoon representing the heterogeneity of tumor samples. Biopsies from different patients are confounded by varying percentages of non-tumor cells, which affects gene expression measurements, whereas tumor cell lines represent pure tumor gene expression. Tumor-specific genes will correlate positively with purity and are lower expressed in tumor samples compared to cell lines. However, environment-specific genes are negatively correlated with purity and will be expressed higher in tumor samples. (**B**) Overview of the identification and generation of STAT3 signatures. STAT3 targets were identified from ChIP-seq data and genes were stratified into tumor- and environmental-specific based on their correlation with tumor purity. Tumor- and environmental-specific weight profiles were used to infer compartment-specific STAT3 activity in gene expression datasets. (**C**) The importance of T- and E-STAT3 activities were evaluated by survival analysis, immune infiltration, genomic characteristics, and the relationship with metastasis.

In this study, we developed a novel method that infers compartment-specific TF activity in gene expression datasets. We first performed a systematic analysis to investigate the differential expression of all human TFs; our analysis included 1164 human TFs expressed in pancreatic cancer and confirmed STAT3 as one of the TFs being more highly expressed in the tumor microenvironment than in cancer cells (Fig. 1A). Given the fact that the  expression level of TFs might not accurately reflect their molecular functions, we applied a computational method to infer the regulatory activity of STAT3 in a sample-specific manner. Specifically, we defined tumor- and environmental-specific STAT3 target genes identified from ChIP-seq experiments, and then calculated compartment-specific STAT3 activities based on the relative expression levels of its target genes (Fig. 1B). Finally, we utilized the compartment-specific STAT3 activities to evaluate the role of STAT3 in prognosis, immune infiltration, and metastasis in pancreatic cancer (Fig. 1C).

### Systematic identification of TME-associated transcription factors

**Figure 2:**
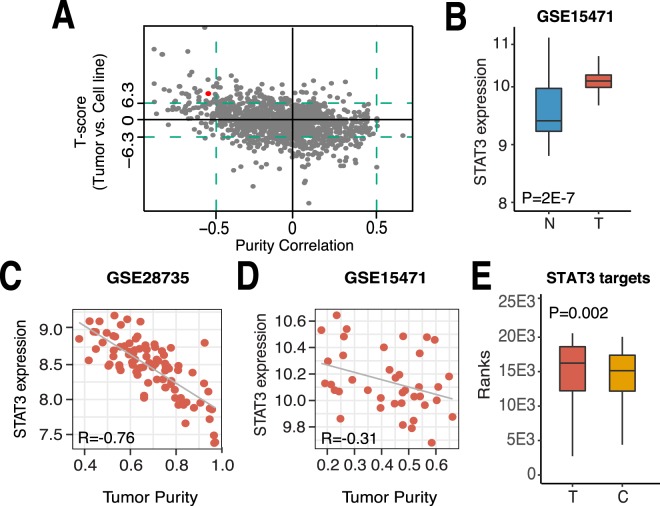
TME STAT3 expression can be detected in pancreatic cancer gene expression studies. (**A**) T-score (tumor vs. cell line) vs. tumor purity correlation (Spearman) for all 1164 TFs expressed in the TCGA PAAD dataset. Red TF indicates STAT3. (**B**) Box plot depicting STAT3 expression in normal pancreatic (**N**) and pancreatic cancer (**T**) tissue in the GSE15471 dataset. STAT3 expression vs. tumor purity in (**C**) the GSE28735 dataset and (**D**) the GSE15471 dataset. (**E**) Box plot of ranked expression of STAT3 targets in pancreatic cancer tissue (**T**) and pancreatic cancer cell lines. (**C**) All p-values were calculated using a two-tailed Wilcoxon test. All correlation coefficients represent the Spearman Correlation Coefficient (SCC).

We systematically investigated the expression patterns of TFs in pancreatic cancer - whether they were more specifically expressed in tumor cells or in microenvironmental non-tumor cells (Fig. 1A). Since no compartment-specific gene expression datasets are available, it is impossible to make direct comparisons. We thus applied indirect comparisons based on the correlation between TF expression levels and tumor purity across pancreatic cancer samples and compared this to pancreatic cancer cell lines, representing pure cancer cells. First, we calculated the correlation between tumor purity and the expression of all 1164 TFs expressed in the TCGA Pancreatic ductal adenocarcinoma (PAAD) dataset. TFs showing positive correlations with purity have higher expression levels in tumor cells and are thus tumor-specific, whereas TFs with negative correlations have higher expression levels in the microenvironment and we thus considered those as environmental-specific. We observed 5 TFs that were positively correlated with tumor purity, exhibiting a Spearman Correlation Coefficient (SCC) greater than 0.5. Meanwhile, 84 TFs were negatively correlated with pancreatic cancer purity, having a SCC less than −0.5 (Fig. 2A).

Second, we compared the expression of TFs between pancreatic tumor tissues and cell lines. Specifically, we compared the expression ranks of TFs in TCGA pancreatic cancer samples with their ranks in pancreatic cell lines using the Student’s t-test. Pancreatic tumor samples are mixed tissues with both tumor and non-tumor cells, whereas cancer cell lines contain merely tumor cells. As such, TFs with a high and low t-statistic (tumor samples versus cell lines) are TME- and tumor-specific, respectively. At the significance level of p < 0.001, we identified zero tumor-specific TFs and 35 TME-specific TFs (Fig. 2A). Combining these two analyses, the expression of 35 TFs could be detected in the TME of pancreatic cancer (Suppl Table 4). In particular, STAT3 was identified as one of the TFs with higher expression levels in TME than in pancreatic cancer cells.

Systematic analysis confirmed the regulatory roles of STAT3 in the TME of pancreatic cancer. We further evaluated STAT3 expression in tumor samples compared to normal pancreatic tissue and observed significant up-regulation of STAT3 expression in pancreatic cancer (p = 2E-7, Fig. 2B), consistent with previous reports^29–31,40^. To corroborate the negative correlation between STAT3 expression and tumor purity observed in the TCGA dataset (Fig. S1A), we further examined three additional datasets and observed identical negative trends (Figs. 2C,D and S1B). Lastly, we observed that STAT3 target genes were more highly expressed in primary pancreatic cancer tissue, compared to pancreatic cancer cell lines (Fig. 2E). This indicated that STAT3 activity, rather than just STAT3 mRNA, might be altered in the TME of pancreatic cancer. Nevertheless, the difference between STAT3 target gene expression between pancreatic tumor tissue and cell lines was not substantial, which suggests that some targets might be mainly regulated by STAT3 in tumor cells, while others are mainly regulated in non-tumor cells. This motivated us to further distinguish the regulatory activity of STAT3 in the tumor and TME compartment.

### Inferring tumor- and environment-specific STAT3 activity

**Figure 3:**
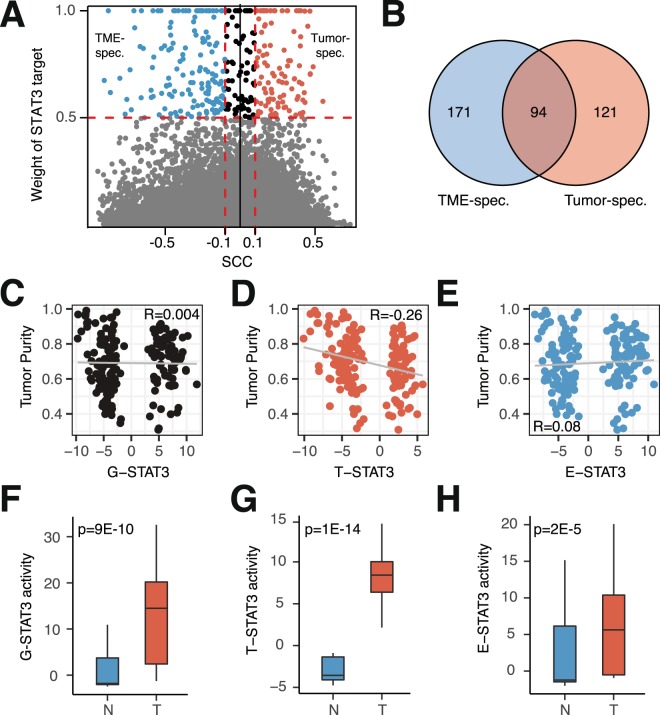
Tumor- and TME-specific STAT3 activities can be inferred by distinct profiles. (**A**) Defining tumor- and environment-specific STAT3 targets by combining ChIP-seq data and correlation of genes with tumor purity. Horizontal dotted line indicates binding affinities smaller than p < 5E-10. (**B**) Venn diagram of STAT3 target genes positively (red) and negatively (blue) correlated with tumor purity. G-STAT3 targets encompass all genes in the Venn diagram. (**C**) G-STAT3 activity vs. tumor purity (SCC = 0.004, P > 0.05). (**D**) T-STAT3 activity vs. tumor purity (SCC = −0.26, P = 5E-4). (E) E-STAT3 activity vs. tumor purity (SCC = 0.08, P > 0.05). Box plots comparing normal pancreatic (**N**) and pancreatic tumor (**T**) tissue in the GSE15471 dataset using (**F**) G-STAT3 activity, (**G**) T-STAT3 activity, and (**H**) E-STAT3 activity (two-sided Wilcoxon test).

To more precisely characterize the regulatory roles of STAT3 in tumor and TME cells, we devised a method to infer compartment-specific STAT3 activity, since previous studies have shown that the regulatory activities of TFs, rather than their mRNA expression levels, more correctly reflect their functions^41^. We extended a previously established algorithm that infers TF activity from high-confidence TF target genes^42^. To this end, we identified a total of 386 STAT3 target genes that were significantly bound by STAT3 (p < 1E-5) according to STAT3 ChIP-seq data (Fig. 3A). Based on their correlation with tumor purity, we divided these targets into a tumor-specific (121 positively correlated genes) and an environmental-specific (171 negatively correlated genes) target gene set, and 94 non-specific target genes (Fig. 3B). Since the STAT family shares a number of target genes, we confirmed that the identified set of STAT3 target genes was almost exclusively specific to STAT3, although some overlap existed between STAT1 and STAT3 in the environmental-specific genes (Fig. S2A). Gene Set Enrichment Analysis (GSEA) of STAT3 target genes showed that tumor-specific genes were enriched in DNA replication and RNA transcription, showing enrichment in for example “packaging of telomers”, “meiotic synapsis” and “RNA pol I promoter opening” (Fig. S2B, Supp Table 5). Environmental-specific genes seemed enriched for immune genes and showed enrichments in “TNF targets”, “IFN gamma response” and “FOXP3 targets” (Fig. S2C, Supp Table 5). We then used these compartment-specific target gene sets to infer the activity of tumor-specific (T-STAT3), environment-specific (E-STAT3), and general (G-STAT3) STAT3 activities in a sample-specific manner utilizing the BASE algorithm^36^. During this calculation, we adjusted STAT3 activity inference scores for tumor purity (see methods). The logic behind this adjustment is that a tumor with high tumor purity does not necessarily have to display high T-STAT3 activity. Without a purity adjustment, this patient would likely receive a high T-STAT3 score just because of high purity, not because of high T-STAT3 activity. Thus, each sample received three scores based on the expression of selected STAT3 target genes.

To show that the inferred compartment-specific STAT3 scores indeed reflected regulatory activities in tumor cells or the TME, rather than capturing tumor purity, we examined their correlation with tumor purity. As shown, correlating the inferred STAT3 activities with tumor purity revealed that STAT3 activity inferences did not reflect tumor purity. On the contrary, although G-STAT3 activity had no correlation with purity (SCC = 0.004, p > 0.05, Fig. 3C), T-STAT3 seemed to be negatively correlated with tumor purity (SCC = −0.26, p = 5E-4, Fig. 3D), whereas E-STAT3 did not show a significant correlation (SCC = 0.08, P > 0.05, Fig. 3E). To further validate our STAT3 inferences, we compared normal pancreatic to pancreatic tumor tissue, expecting that only tumor tissue should show high STAT3 activities. Indeed, we were able to distinguish between normal pancreatic and pancreatic tumor tissue using G-STAT3 activity (Fig. 3F) but were able to more specifically infer STAT3 activity using T- and E-STAT3 (Fig. 3G,H). Assuredly, T-STAT3 activity was not detected in normal pancreatic tissue, whereas tumor tissue, as expected, showed high activity levels (P = 1E-14, Fig. 3G). These findings were also confirmed in an independent dataset (Fig. S2D–F). Thus, these results indicated that we can distinguish between tumor and environmental STAT3 activities using our novel approach.

### Activity but not expression of STAT3 is associated with patient survival

**Figure 4:**
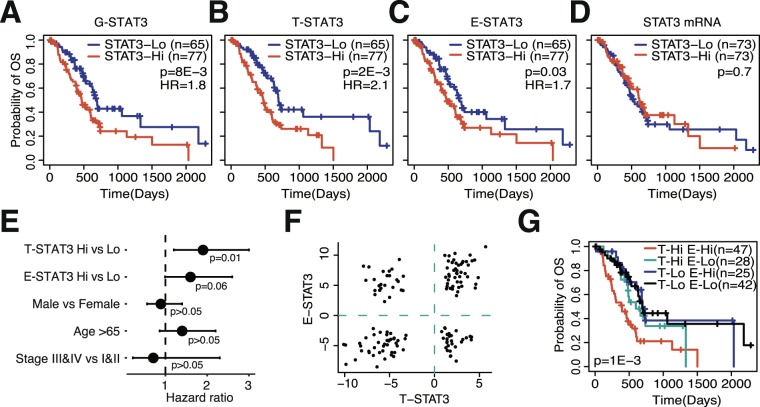
STAT3 activities are prognostic in pancreatic cancer. Kaplan-Meier plot depicting the survival probability over time for samples with high (red) and low (blue) STAT3 activity, (**A**) G-STAT3 activity, (**B**) T-STAT3 activity, (**C**) E-STAT3 activity. (**D**) Kaplan-Meier plot depicting the survival probability over time for samples with high (red) and low (blue) STAT3 expression. (E) Forest plots showing the hazard ratios of STAT3 activities and clinical variables. (**F**) Distribution of T- and E-STAT3 scores. (**G**) Kaplan-Meier plot comparing survival probabilities of T-STAT3-Hi/E-STAT3-Hi (n = 47, T-STAT3-Hi/E-STAT3-Lo (n = 28), T-STAT3-Lo/E-STAT3-Hi (n = 25), and T-STAT3-Lo/E-STAT3-Lo (n = 42). All figures are based on the TCGA PAAD dataset.

After being able to differentiate between tumor- and TME-specific STAT3 activity, we next evaluated if this distinction has prognostic relevance. Previous studies have shown that elevated STAT3 activity is correlated with poor prognosis in pancreatic cancer^29,43^. By stratifying samples into STAT3 activity-high (STAT3 activity score > 0) and -low (STAT3 activity score < 0) groups, we indeed confirmed that high STAT3 activities conferred poor prognosis, irrespective of tumor compartment (Fig. 4A–C). However, no distinction in survival probability was observed using STAT3 mRNA as an indicator of survival (p > 0.05, log-rank test) (Fig. 4D). These results were confirmed in independent datasets (Fig. S3). As pancreatic cancer survival is associated with other attributable risk factors, such as stage, age and gender^44^, we evaluated the prognostic efficacy of STAT3 compared to the prognostic value of these clinical variables. T- and E-STAT3 were the only factors significantly associated with prognosis (Fig. 4E). T-STAT3 was the most significant prognostic factor and conveyed a hazard ratio of 1.9 (p = 0.01, multivariate Cox regression).

We next evaluated if the combination of T- and E-STAT3 activities added prognostic value compared to single STAT3 activities. The distribution of T- and E-STAT3 scores was fairly equal in the TCGA dataset (Fig. 4F), which provided us with enough power to reliably compare the survival probabilities of four groups: T-STAT3-Hi/E-STAT3-Hi (n = 47, T-STAT3-Hi/E-STAT3-Lo (n = 28), T-STAT3-Lo/E-STAT3-Hi (n = 25), and T-STAT3-Lo/E-STAT3-Lo (n = 42). We found that the combination of high T-and E-STAT3 activities was associated with poor survival, whereas the combination of low T- and E-STAT3 activities conferred the best survival (Fig. 4G) (p = 1E-3), which was also confirmed in independent datasets (Fig. S4). Thus, these results indicated that combination of compartment-specific STAT3 activities can serve as prognostic marker in pancreatic cancer.

### E-STAT3 activity is associated with a specific TME composition

Based on the previously recognized coordination between STAT3 activity in cells of the TME and tumor cells, we were curious if we could assess this interaction with our compartment-specific framework. To investigate this, we first we attempted to uncover the source of E-STAT3 activity. Since STAT3 can be active in CAFs^26–28^ and in immune cells^15^, we assessed whether E-STAT3 signals were more associated with the stromal compartment or the immune compartment. We inferred the levels of immune and stromal involvement using the ESTIMATE algorithm^45^ and then used conditional correlations to assess if E-STAT3 activity was more associated with the stromal or the immune compartment. We consistently observed that E-STAT3 was positively correlated with immune scores when adjusted for by stromal scores (GSE15471: SCC = 0.59, p = 9E-5; GSE28735: SCC = 0.40, p = 0.007; TCGA: SCC = 0.15, p = 0.04), whereas stromal scores were negatively correlated with E-STAT3 when adjusted for by immune scores (ICGC: SCC = −0.34, p = 8E-9; GSE57492: SCC = −0.42, p = 0.007; GSE15471: SCC = −0.48,: p = 0.026; GSE28735: SCC = −0.32, p = 0.03; TCGA: SCC = −0.20, p = 0.007). This indicated that E-STAT3 activity likely originated from tumor-infiltrating immune cells.

**Figure 5:**
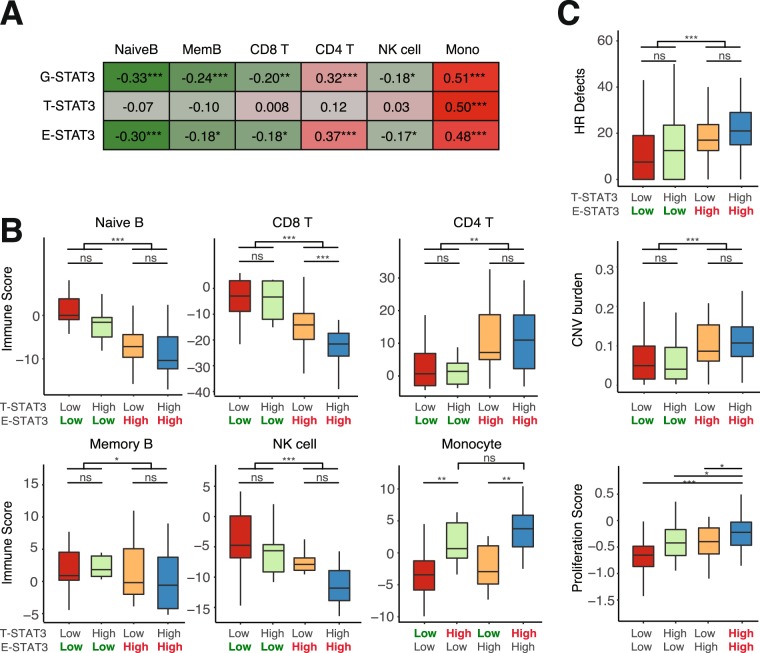
E-STAT3 activity is associated with a specific TME composition. (**A**) SCC of STAT3 activity and immune infiltration scores in TCGA. (**B**) Boxplots showing the infiltration of several immune cell types in four STAT3 groups in the GSE57495 dataset (two-sided Wilcoxon test). (**C**) Boxplots showing HR deficiency, CNV burden and proliferation scores in four STAT3 groups in TCGA, *p ≤ 0.05, **p ≤ 0.01, ***p ≤ 1E-3 (two-sided Wilcoxon test).

To further elucidate which immune cells are associated with E-STAT3 activity, we examined the correlation between immune infiltration and STAT3 activity (Fig. 5A). Specifically, we applied a computational method to calculate the infiltration level of six immune cell subtypes in pancreatic tumor samples^46^. We found that T- and E-STAT3 activity were both most strongly associated with the monocyte profile (T-STAT3: SCC = 0.50, P < 2E-16; E-STAT3: SCC = 0.48, P = 2E-11), whereas E-STAT3 activity was also positively correlated with CD4 T cells (SCC = 0.37, P = 4E-7) and negatively correlated with naïve B cells (SCC = −0.30, p = 7E-5) (Fig. 5A). Since a variety of CD4 T cell subtypes exists, we investigated if we could further narrow down which CD4 T cell subset was associated with E-STAT3. Using CD4 T cell marker genes from a previous publication^47^, we found that both activated CD4 T cells and Th2-polarized CD4 T cells were the only significantly associated CD4 subtypes across independent datasets (Fig. S4E,F). Thus, this indicated a relation between T- and E-STAT3 activities with monocyte infiltration, but only an association with other immune cells and E-STAT3 activity.

To investigate these relations in more detail, we stratified samples into the aforementioned STAT3 groups (T-STAT3-Hi/E-STAT3-Hi, T-STAT3-Hi/E-STAT3-Lo, T-STAT3-Lo/E-STAT3-Hi, and T-STAT3-Lo/E-STAT3-Lo). Three consistent patterns were observed across pancreatic cancer datasets. First, a T-STAT3-dominant pattern was observed for monocyte infiltration, where monocytes were enriched in T-STAT3 high samples, irrespective of E-STAT3 activity (Figs. 5B and S5A). Second, we observed an E-STAT3-dominant pattern in which CD4 T cells were high in E-STAT3 high samples, irrespective of T-STAT3 (Figs. 5B and S5A,B). Lastly, naïve B cells, CD8 T cells, and NK cells were enriched in E-STAT3 low samples, irrespective of T-STAT3 activity (Figs. 5B and S5A,B). The reproducibility of these patterns across datasets suggests that this was not a dataset-specific observation, but a generalizable pancreatic cancer characteristic. In addition, two of the patterns seemed to be dominated by E-STAT3, indicating a dominant role for E-STAT3 in initiating or maintaining an exclusive TME, in which the presence of high E-STAT3 precludes the presence of anti-tumor immune cells, such as CD8 T and NK cells.

We next evaluated whether tumor intrinsic characteristics were associated with T- and/or E-STAT3 activity. Previously, several studies have defined pancreatic cancer subtypes^48,49^. We thus evaluated if T-and E-STAT3 scores were associated with any of the identified subtypes. T-STAT3 activity was highly enriched in the squamous subtype reported by Bailey *et al*.^48^ and in the quasi-mesenchymal subtype reported by Collisson *et al*.^49^(Fig. S6A). E-STAT3 was decreased in the ADEX subtype reported by Bailey *et al*.^48^ and exocrine subtype reported by Collisson *et al*.^49^ (Fig. S6A). Next, tumor mutation burden did not differ between STAT3 groups (data not shown), an E-STAT3-dominant pattern was again observed for CNV burden and homologous recombination (HR) deficiency, which were significantly elevated in E-STAT3 high samples, irrespective of T-STAT3 activity (Fig. 5C). CNV burden is known to be correlated with immune evasion, where high CNV burden is predictive of higher levels of immune evasion^50^, which is in line with our findings of lower CD8 T, and NK cells in high CNV burden groups. In addition, proliferation scores were highest in T-and E-STAT3 high samples (Fig. 5C), implying that the coordination between T- and E-STAT3 provides some growth advantages to tumor cells compared to other STAT3 groups. In conclusion, these results show that E-STAT3 is associated with intrinsic and extrinsic tumor characteristics in pancreatic cancer.

### Differential STAT3 activity between primary and metastatic pancreatic cancer

Several reports have indicated a role for STAT3 in promoting metastasis and invasion^29–31,40,51^. Additionally, since most pancreatic cancer patients are identified at an advanced stage, we were interested in the role of STAT3 during metastasis. We obtained a dataset that included gene expression data from pancreatic cancer lesions at several metastatic sites^52^. Similar to our earlier findings, STAT3 expression was inversely correlated with tumor purity at the primary site (Fig. S6B) and was also negatively correlated at metastatic lesions from liver, whereas it was trending to be significant in lung and lymph node (Fig. S6C–E). This suggested to us that TME-specific STAT3 activity might play a role at metastatic sites as well.

**Figure 6:**
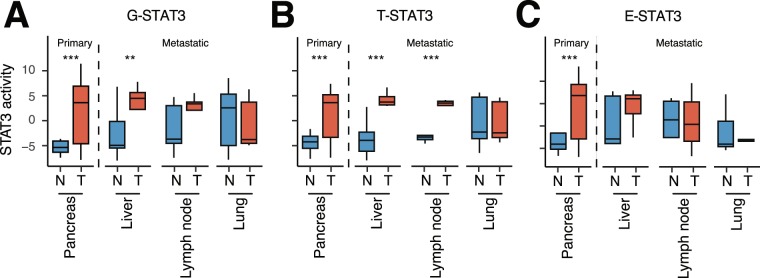
T-STAT3 activity persists, but E-STAT3 activity is unaltered at metastatic sites. (**A**) G-STAT3, (**B**) T-STAT3, (**C**) and E-STAT3 activities in primary pancreatic, metastatic liver, metastatic lymph node, and metastatic lung lesions (**T**) and corresponding normal (N) tissue. *p ≤ 0.05, **p ≤ 0.01, ***p ≤ 1E-3 (two-sided Wilcoxon test).

To follow up, we inferred G-, T-, and E-STAT3 activity in samples of liver, lymph node, and lung metastases. Compared to corresponding normal tissue, G-STAT3 activity was again significantly increased at the primary pancreatic cancer site, but also in liver metastases (Fig. 6A). However, the deconvolution of G-STAT3 into T- and E-STAT3 activities revealed that only T-STAT3 activity, but not E-STAT3 activity, was significantly increased in metastatic liver tissue and also in lymph node metastases (Fig. 6B). Metastatic lung tissue seemed to be an exception, since no difference in T-STAT3 activity was observed between normal and tumor tissue. A potential explanation is the relatively high basal STAT3 expression level in normal lung tissue compared to other tissues (Fig. S7). None of the metastatic sites showed a significant difference between E-STAT3 activity in normal and tumor tissue (Fig. 6C), indicating that E-STAT3 might not be essential at metastatic lesions. However, these results indicated that T-STAT3 seemed to play a major role in pancreatic cancer metastasis as two out of three metastatic sites showed an increase in T-STAT3 activity compared to normal tissue.

## Discussion

STAT3 contributes in several ways to the distinctive immunosuppressive pancreatic TME through its activity in several cell types, including immune and tumors cells. In this study, we developed a framework to assess STAT3 activity in the TME and tumor cell compartment. We found that STAT3 activities have prognostic features, high T- or E-STAT3 activity conferring poor prognosis, but STAT3 mRNA does not. We also observed different requirements for STAT3 in the primary tumor and at metastatic sites, E-STAT3 being more dominant in pancreatic lesions and T-STAT3 seeming to be more important at metastatic lesion. Collectively, we show that our framework can be utilized to obtain biological insights and that the distinction between E- and T-STAT3 is crucial when investigating STAT3 in pancreatic cancer.

We identified four STAT3 groups (T-STAT3-high/E-STAT3-high, T-STAT3-high/E-STAT3-low, T-STAT3-low/E-STAT3-high, and T-STAT3low/E-STAT3-low) with distinct tumor characteristics. By assessing intrinsic and extrinsic tumor characteristics of these groups, we observed three STAT3 patterns: a T-STAT3 dominant pattern, a E-STAT3 dominant pattern, and an E-STAT3 depleted pattern. Whereas the T-STAT3 dominant pattern was only associated with high monocyte infiltration, the E-STAT3 dominant pattern was characterized by high CD4 T cell infiltration, high CNV burden, and high HR deficiency burden. The E-STAT3-depleted pattern was associated with elevated B, CD8 T, and NK cell infiltration. Since these patterns were observed across datasets, this suggests a general pancreatic cancer characteristic which could be utilized to identify high-risk patients, i.e. patients with high T- and E-STAT3 activities.

T-STAT3-high/E-STAT3-high samples had worst prognosis and the highest proliferation scores. It is likely that the combination of high E- and T-STAT3 activities confers tumor growth advantages compared to other STAT3 groups. This group seemed to have an immunosuppressive environment, characterized by the infiltration of monocytes and CD4 T cells, absense of cytotoxic immune cells, and relatively high CNV burden. Intriguingly, a macrophage–tumor cell feedback mechanism has been described in ovarian cancer, in which STAT3 activity in either macrophages or tumor cells can activate STAT3 activity in the other cell type^24^. A similar mechanism might be present in a subset of pancreatic cancer patients with high T- and E-STAT3 activities. Although this hypothesis might not be sufficient to explain the extremely poor prognosis in this group of patients, it does point to a specific tumor composition in which T- and E-STAT3 are coordinated to provide tumor cells with a proliferation advantage compared to patients that do not display high T- and E-STAT3 activity.

The origin of E-STAT3 activity cannot be determined exactly, but we hypothesize that a combination of CD4 T cells, specifically Th2-polarized CD4 T cells, and myeloid cells contributes to the E-STAT3 signal. First, CD4 T cells and monocytes are most highly correlated with E-STAT3 activity. Second, Th2 cells and myeloid cells are abundant in the pancreatic TME in certain patients^14,53–55^. Lastly, both Th2 cells and myeloid cells have been shown to propagate pancreatic cancer growth. Myeloid cells are immunosuppressive and secrete cytokines that prevent the activation of tumor-eliminating immune cells^53,56^, which is consistent with our observations of low CD8 T and NK cell infiltration in E-STAT3 high samples, but not in E-STAT3 low samples. Although CD4 Th2 cells are commonly involved in parasitic responses and allergy, their role in pancreatic cancer seems to be the exacerbation of fibrosis and prevention of collagen clearance^54,55^. However, experimental validation is necessary to confirm E-STAT3 activity in these cell types.

A better understanding of STAT3 activities is essential in identifying new therapeutic avenues. Due to the previous notion that STAT3 is aberrantly activated in tumor-infiltrating immune cells, STAT3 pathway inhibition has been suggested in immunotherapy combinations^57^. Co-targeting IL-6, which is major factor in activating STAT3, and PD-L1 was shown to inhibit growth in a murine model of pancreatic cancer^58^. Identifying which immune cells are major contributors to STAT3 activity might identify additional drugs that inhibit these cell types. In addition, clinical trials have recently been initiated to test the efficacy of STAT3 pathway inhibitors in pancreatic cancer (Clinical Trial Identifiers NCT02767557, NCT02983578). Although no preliminary results are available yet, inhibitors of IL-6 and the IL-6 receptor have been proven to be effective in preclinical models of KRAS-driven pancreatic cancer^59^. Thus, further stratifying the involvement of TFs in pancreatic cancer might reveal new treatment strategies.

Although we believe that our results add valuable insights into the role STAT3 in pancreatic cancer, we note a few limitations present in this study. First, our framework of TF inference relies on TF target genes, which indicates that if two TFs share a large number of common targets, the inferred TF activities will be correlated. The family of STAT TFs has a number of shared target genes and we cannot exclude the possibility that some of these shared target genes might have affected STAT3 activity by being transcribed by another STAT TF. Second, our STAT3 target profiles are in part based on pancreatic cancer cell lines, which might not fully reflect pancreatic cancer cells within a tumor environment. Third, even though we have narrowed down tumor and environment-specific genes using a tumor purity, we cannot exclude the possibility that genes specific to the tumor are expressed in the TME and vice versa.

In conclusion, we have shown that we can distinguish between tumor and environmental STAT3 activity in gene expression studies and that this distinction leads to biological and clinical insights. Our analysis provides a framework by which to study tumor- and TME-specific TF activity levels and can be expanded to other TFs and cancer types.

## Materials and Methods

### Pancreatic cancer datasets

RNAseq data for Pancreatic Ductal Adenocarcinoma (PAAD), generated by The Cancer Genome Atlas (TCGA) were downloaded though FireBrowse in June 2015 (level 3, RNAseqV2). Absolute expression values were log10 transformed. A number of samples was excluded based on previous reports indicating that some samples do not represent pancreatic cancer^60,61^, resulting in the inclusion of 150 patient samples (see Suppl Table 1 for exclusion criteria). HR deficiency and proliferation scores were downloaded as a supplemental file from prior work^62^. Five independent pancreatic cancer datasets were used in this study. Four pancreatic cancer microarray datasets were obtained from Gene Expression Omnibus (GEO) under accession numbers GSE15471^63^, GSE57495^64^, GSE71729^52^, GSE28735^65^. These datasets were provided as normalized expression at the probeset level, in which some genes might be represented by multiple probesets. We converted probeset expression into gene expression values. Specifically, for one-channel arrays, we selected the probeset with the highest hybridization intensity across all samples to represent gene expression. For two-channel arrays, the average expression values of all probesets were calculated to represent gene expression. Datasets from one-channel arrays were further median normalized for each gene to transform intensities into relative expression values. The last RNAseq pancreatic cancer dataset was obtained from the ICGC project at ww.dcc.icgc.org and only primary tumor specimens classified as pancreatic ductal carcinoma were included (Suppl Table 1).

Forty-four pancreatic cancer cell lines were used to compare tumor gene expression to primary pancreatic cancer gene expression (Suppl Table 2). Cell line datasets were obtained from the Broad Institute Cancer Cell Line Encyclopedia (CCLE).

### STAT3 activity profile generation

First, we defined STAT3 consensus target genes based on ChIP-sep data from the Encyclopedia of DNA Elements (ENCODE) project by using a computational method called Target Identification from Profiles (TIP)^42^. This algorithm calculates p-values that indicate the binding probability of genes by STAT3, smaller p-values indicating higher probability of binding. We used log-transformed p-values to represent STAT3-target gene binding affinities. A total of 386 high-affinity STAT3 target genes were identified above an affinity threshold of 0.5 (i.e., p < 10E-5). Second, we divided these targets into a tumor-specific and an environmental-specific target gene set, based on their positive and negative correlation with pancreatic cancer tumor purity, respectively. This resulted in 121 tumor- and 171 environmental-specific genes and 94 genes that did not show compartment-specificity. Third, three STAT3 profiles were created based on these groups; general-STAT3 (G-STAT3) containing all 386 STAT3 target genes, tumor STAT3 (T-STAT3) containing 121 genes, and environmental STAT3 (E-STAT3) containing 171 genes (Suppl Table 3). Target genes for STAT1, STAT2 and STAT5a were calculated in an identical manner. Overlap between the different STAT profiles (Fig. S2A) were displayed using the R “venn” package. The weight of each gene was based on STAT3-target gene binding affinities as calculated by TIP, genes with high affinity receiving higher weights than genes with lower affinity. Last, we defined compartment-specific reference gene sets in order to adjust the inferred STAT3 activities for tumor purity. These reference gene sets included genes correlated with tumor purity (absolute value of SCC > 0.1) but did not reach the affinity threshold of 0.5. We have provided gene-level annotations and additional information for each of the 386 identified STAT3 target genes in Suppl. Table 6.

### Compartment-specific STAT3 activity inference

STAT3 activity scores were calculated by the (Binding Associated with Sorted Expression) BASE algorithm^36,66^. Each of the three STAT3 signatures was inputted into BASE along with a patient gene expression matrix. If the gene expression dataset was a one channel microarray and RNAseq dataset, we median normalized the data before inputted it into BASE. The BASE algorithm calculated STAT3 activity scores for each patient sample by ranking genes in descending order based on gene expression values. Two cumulative distributions were then generated by a foreground (f) and background (b) function. These functions are given by:

$$f(i)\,=\frac{{\sum }_{k=1}^{i}|{g}_{k}{w}_{k}|}{{\sum }_{k=1}^{n}|{g}_{k}{w}_{k}|},1\le i\le {\rm{n}}$$

$$b(i)\,=\frac{{\sum }_{k=1}^{i}|{g}_{k}(1-{w}_{k})|}{{\sum }_{k=1}^{n}|{g}_{k}(1-{w}_{k})|},1\le i\le {\rm{n}}$$

where the weight *w* represents the affinity of STAT3 targets as determined by ChIP-seq, *g* is the expression value of gene *j* in the patient expression profile. The maximal deviation between these two distributions represents the preliminary STAT3 activity score. This score is adjusted for by 1000 iterations of random patient expression profiles. The resulting score constituted the STAT3 activity score, where a higher score represented greater STAT3 activity and lower scores lower activity.

In order to obtain compartment-specific scores, we adjusted T- and E-STAT3 activities for purity. We utilized all genes in the gene expression datasets to calculate G-STAT3 activity since this inference should be minimally affected by tumor purity. To calculate T-STAT3 activity scores, we only selected tumor-specific STAT3 target genes (T-STAT3) and reference genes (SCC > 0.1) from the gene expression dataset and used this as the patient gene expression input for BASE; to calculate E-STAT3 activity scores, we only selected microenvironment-specific STAT3 target genes (E-STAT3) and reference genes (SCC < −0.1). Since neither STAT3 target genes or reference genes are overlapped, the resulting sample-specificT-STAT3 and E-STAT3 activities are inherently independent of each other.

### Gene Set Enrichment Analysis

Gene Set Enrichment Analysis (GSEA) was performed on ranked STAT3 profiles using the GSEA software (version 3.0) provided by the Broad Institute, available at http://software.broadinstitute.org/gsea/index.jsp. All pathways in the C2 database (version 6.2) were used for this analysis.

### Calculation of tumor purity and infiltration scores

Tumor purity scores of pancreatic cancer samples obtained from TCGA were obtained from: http://bioinformatics.mdanderson.org/estimate/ (December, 2017), while tumor purity scores for other datasets were calculated using the R “estimate” package^45^. The infiltration of tumor and stromal cells were also calculated based on the R “estimate” package. Immune infiltration scores of specific immune cell types were calculated using our established framework described in^46^. In short, immune cell-specific weight profiles and a patient gene expression dataset were inputted into BASE to infer the infiltration of selected immune cells; naïve B cells, memory B cells, CD4 T cells, CD8 T cells, NK cells, and monocytes.

### Copy number variation and total mutation burden

Genomic features of the TCGA PAAD dataset were calculated based on MAF files and DNA-sequencing profiles, downloaded from FireBrowse (gdac.broadinstitute.org/). Copy number variation data provided by TCGA was used to calculate total copy number variation (CNV) burden for each sample, which represented the deviation of total copy number compared to normal (no copy number alterations). For each DNA fragment, its copy number was divided by two to account for diploidy, log2 transformed and multiplied by the size of the DNA fragment to take into account the magnitude of copy number alteration. The CNV scores for all fragments were then summed and scaled by the length of the entire genome to generate the final CNV burden score. This can be represented by:

$$CNV\,burden=\frac{{\sum }_{i=1}^{n}|log2({C}_{i}/2)\ast {s}_{i}|}{N}$$

where *C*_*i*_ and *s*_*i*_ represent the copy number and the size DNA segments $i$ in a sample, *n* is the total number of segments in the genome and N is the size of the human genome. Tumor mutation burden (TMB) was represented by the number of non-silent mutations in a given PAAD sample.

### Survival analysis and forest plots

The efficacy of patient-specific STAT3 activity scores in predicting overall survival were verified by Cox proportional hazards models using the “coxph” function from the R “survival” package. Using zero as a cutoff for STAT3 activity, patients were stratified into STAT3-high and STAT3-low groups. Cumulative incidence and the proportion of survival was calculated by log-rank tests using the R “survival” package. Kaplan-Meier curves were generated using the “survfit” function. The statistics calculated to present the difference between survival curves were generated by the “survdiff” function. Forest plots were generated by multivariate Cox regression using the “coxph” function. Variables included in this multivariate analysis were T-STAT3 activity, E-STAT3 activity, gender, age and tumor stage. Unless indicated otherwise, all correlations were performed using Spearman correlation. Conditional correlations were calculated using the “pcor.test” function from the R “ppcor” package. R version 3.4.1 was used for all analyses.

## Supplementary information

Supplementary Figures and Tables

Accompanying code

## Data Availability

All data used in this study are publicly available and sources are provided in Supplementary Table 1. R code to generate figures and results are provided in a supplementary folder for GSE57495. Using this example, figures for the additional datasets used in this study can be obtained in a similar manner.
