# Supplementary material for: Computational STAT3 activity inference reveals its roles in the pancreatic tumor microenvironment
Source: Sci Rep. 2019 Dec 3;9:18257. doi: 10.1038/s41598-019-54791-x (PMC6890662; doi:10.1038/s41598-019-54791-x)
Supplement: Supplementary file 2 — Accompanying code [file 41598_2019_54791_MOESM2_ESM.zip › Code/Plots/Chen_ImmuneInference_boxplots.pdf]

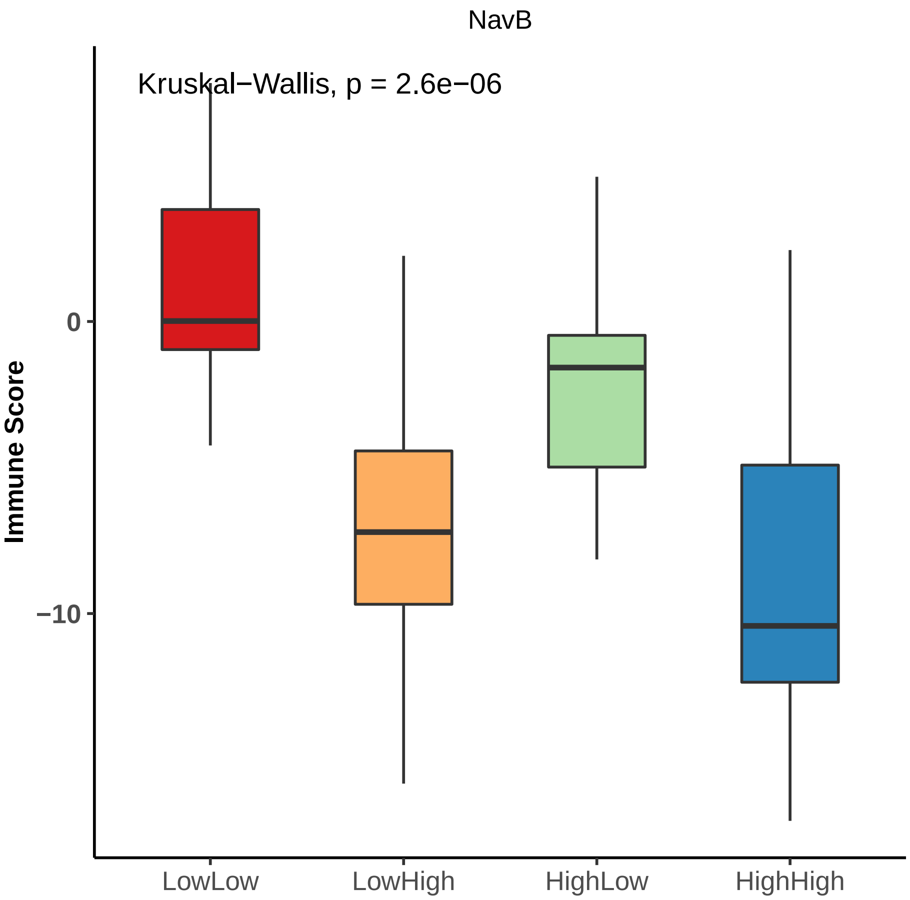
NavB
Kruskal−Wallis, p = 2.6e−06
Immune Score
0
−10
LowLow
LowHigh
HighLow
HighHigh

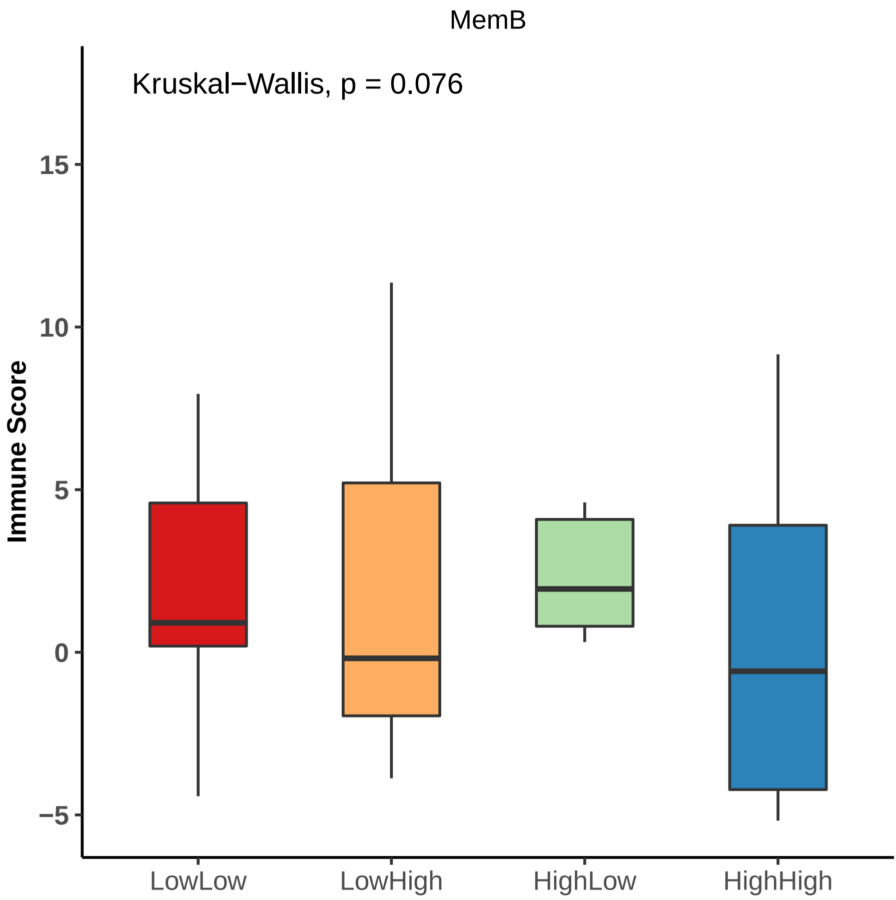
MemB
Kruskal−Wallis, p = 0.076
Immune Score
15
10
5
0
−5
LowLow
LowHigh
HighLow
HighHigh

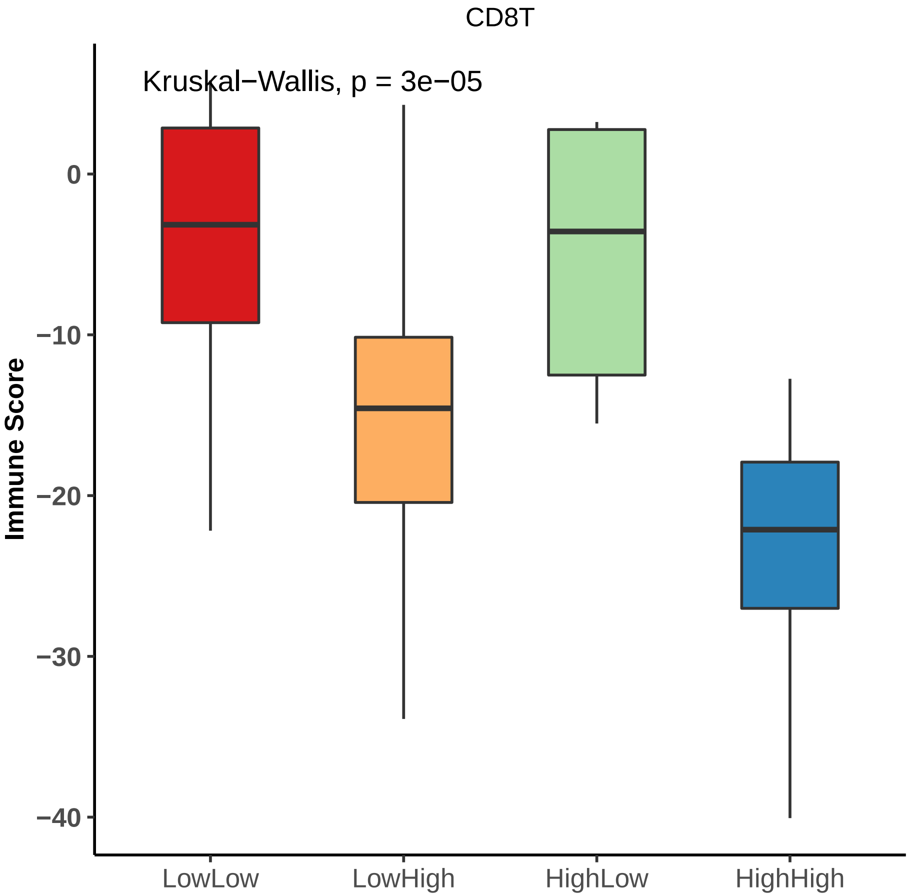
CD8T
Kruskal−Wallis, p = 3e−05
Immune Score
0
−10
−20
−30
−40
LowLow
LowHigh
HighLow
HighHigh

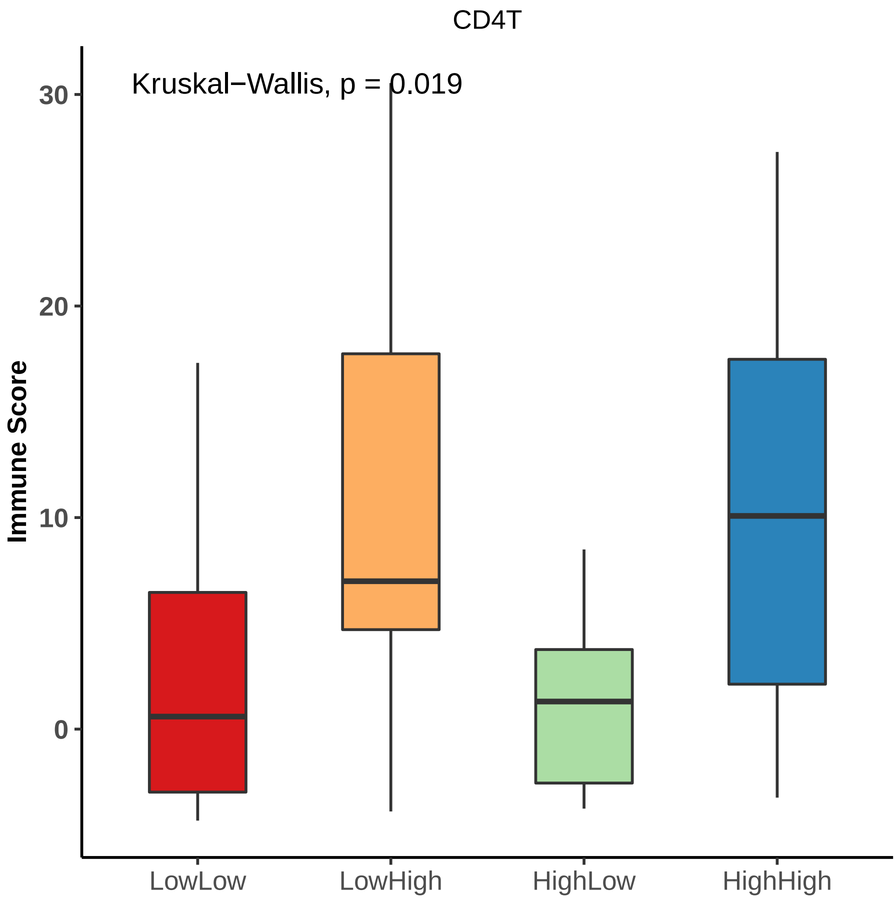
CD4T
Kruskal−Wallis, p = 0.019
Immune Score
30
20
10
0
LowLow
LowHigh
HighLow
HighHigh

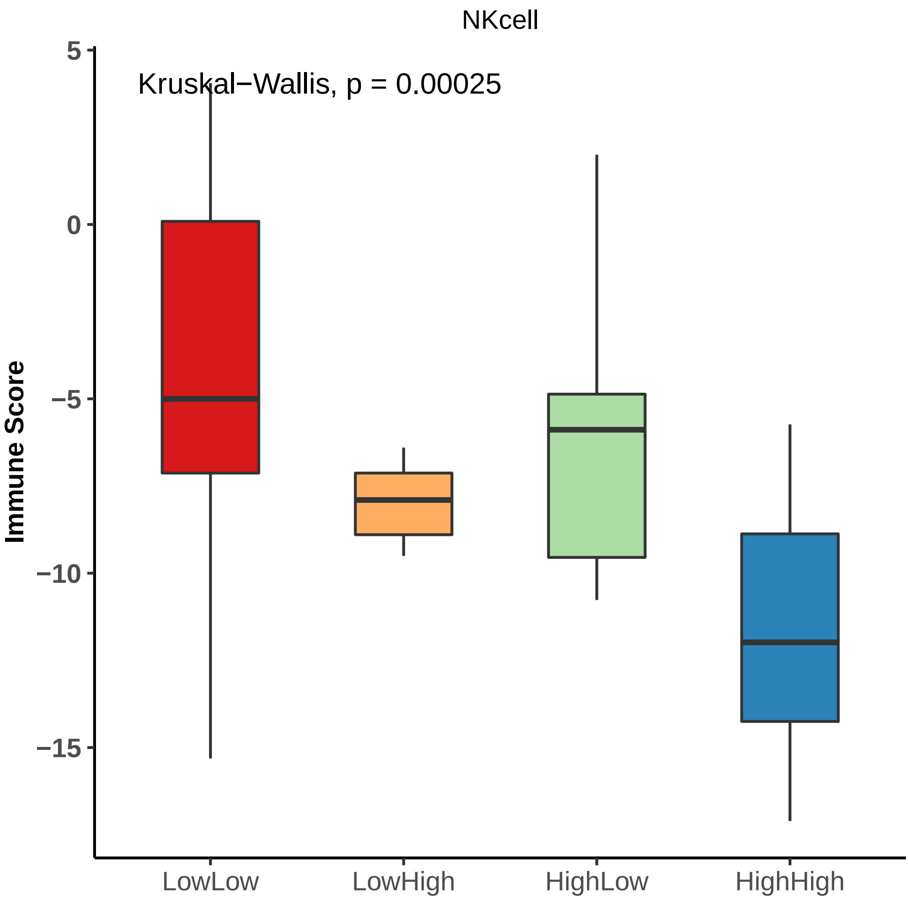
NKcell
Kruskal−Wallis, p = 0.00025
5
0
−5
−10
−15
Immune Score
LowLow
LowHigh
HighLow
HighHigh

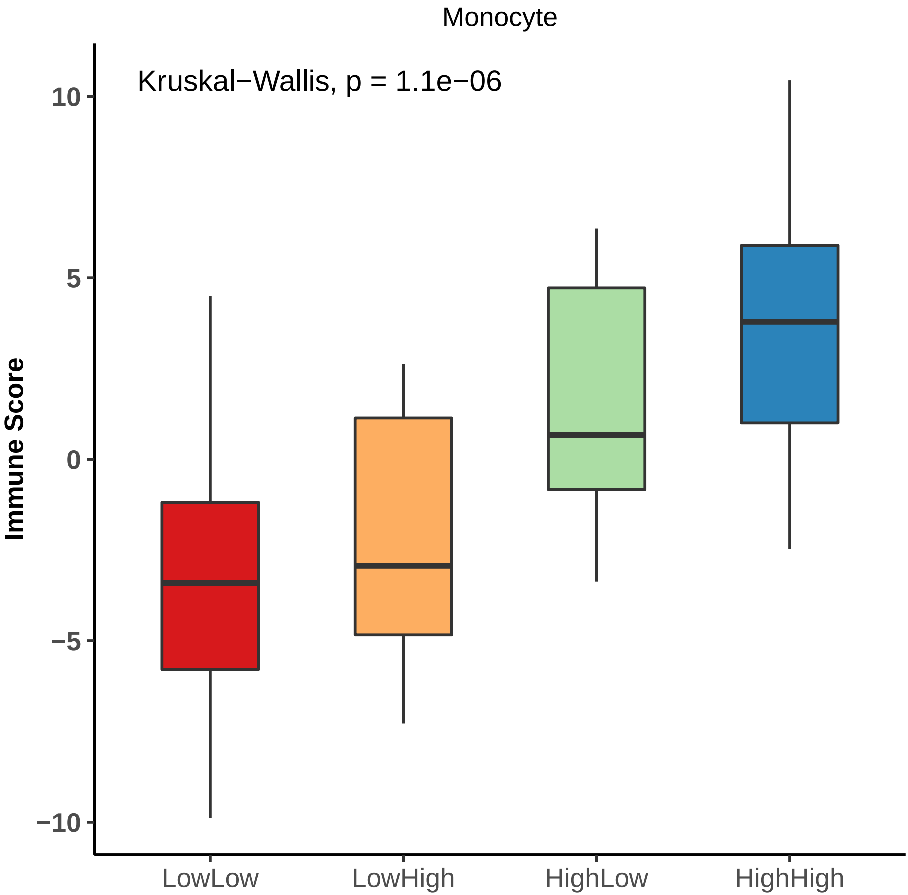
Monocyte
Kruskal−Wallis, p = 1.1e−06
Immune Score
10
5
0
−5
−10
LowLow
LowHigh
HighLow
HighHigh
