# Supplementary figures and images for: Computational STAT3 activity inference reveals its roles in the pancreatic tumor microenvironment
Source: Sci Rep. 2019 Dec 3;9:18257. doi: 10.1038/s41598-019-54791-x (PMC6890662; doi:10.1038/s41598-019-54791-x)

## Chen\_GSE57495 T-STAT3

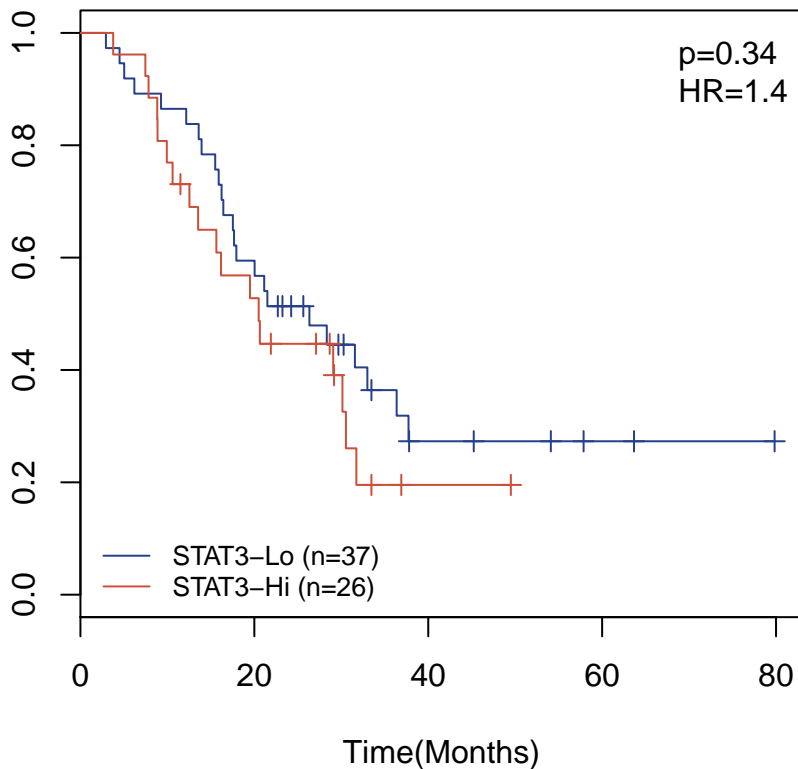

Supplement: Supplementary file 2 — Accompanying code [file 41598_2019_54791_MOESM2_ESM.zip › Code/Plots/SuppFig_3_Chen_E_STAT3_KM_plot.pdf]

# GSE57495 G-STAT3

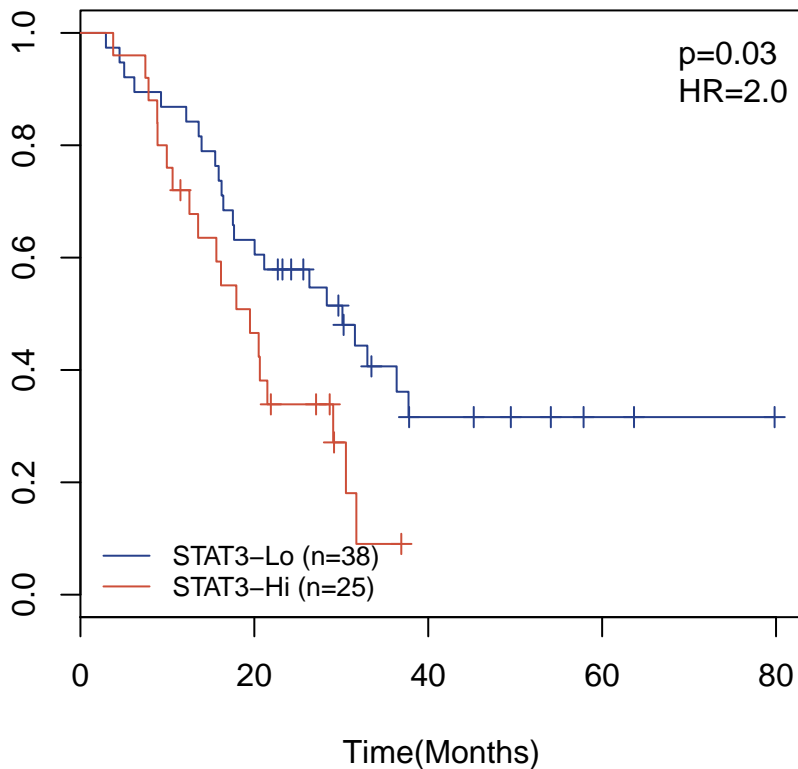

Supplement: Supplementary file 2 — Accompanying code [file 41598_2019_54791_MOESM2_ESM.zip › Code/Plots/SuppFig_3_Chen_G_STAT3_KM_plot.pdf]

## Chen\_GSE57495 T-STAT3

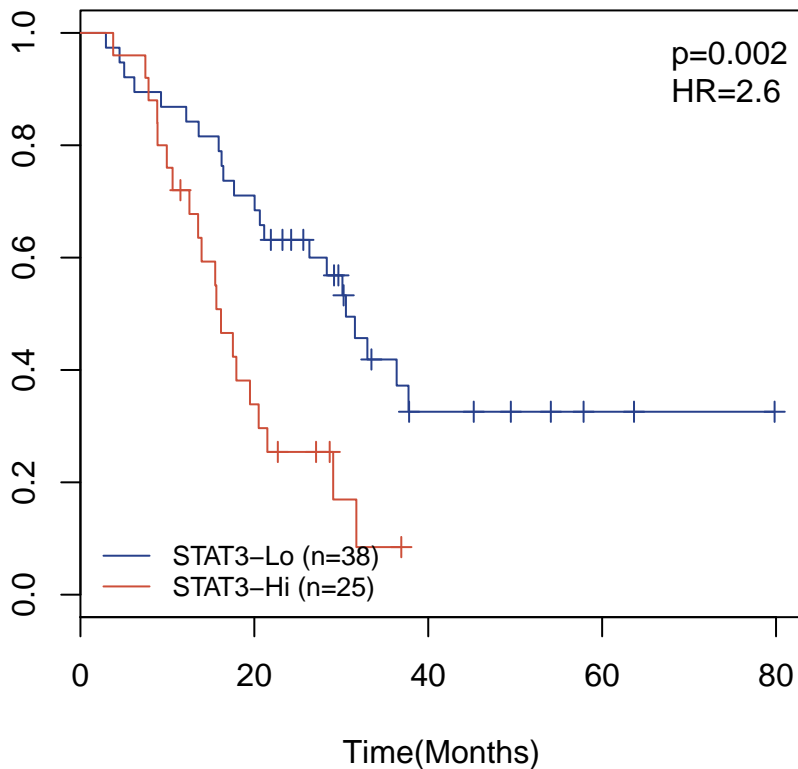

Supplement: Supplementary file 2 — Accompanying code [file 41598_2019_54791_MOESM2_ESM.zip › Code/Plots/SuppFig_3_Chen_T_STAT3_KM_plot.pdf]
