# Supplementary material for: Computational STAT3 activity inference reveals its roles in the pancreatic tumor microenvironment
Source: Sci Rep. 2019 Dec 3;9:18257. doi: 10.1038/s41598-019-54791-x (PMC6890662; doi:10.1038/s41598-019-54791-x)
Supplement: Supplementary file 1 — Supplementary Figures and Tables [file 41598_2019_54791_MOESM1_ESM.pdf]

Supplementary Figures

Computational STAT3 activity inference reveals its roles in the pancreatic tumor microenvironment

Evelien Schaafsma[1], Yiwei Yuan[1], Yanding Zhao[1], Chao Cheng*[2,3,4]

[1] Department of Molecular and Systems Biology, Geisel School of Medicine at Dartmouth, Hanover, NH 03755, USA; [2] Department of Biomedical Data Science, Geisel School of Medicine at Dartmouth, Lebanon, NH 03756, USA; [3] Department of Medicine, Baylor College of Medicine, Houston, TX 77030, USA; and [4] The Institute for Clinical and Translational Research, Baylor College of Medicine, Houston TX 77030, USA.

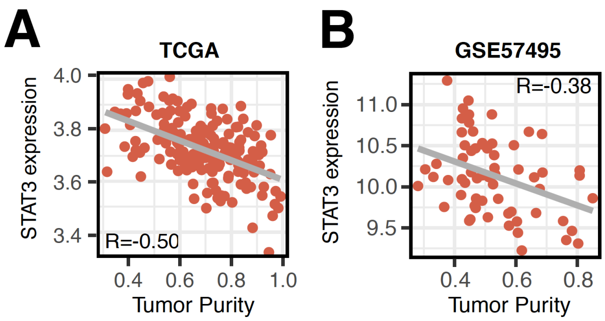

S1. STAT3 expression correlated with tumor purity. Correlation of STAT3 expression and tumor purity in the (A) TCGA dataset (SCC=-0.50, p=2E-16) and (B) GSE57495 dataset (SCC=-0.38, p=2E-3).

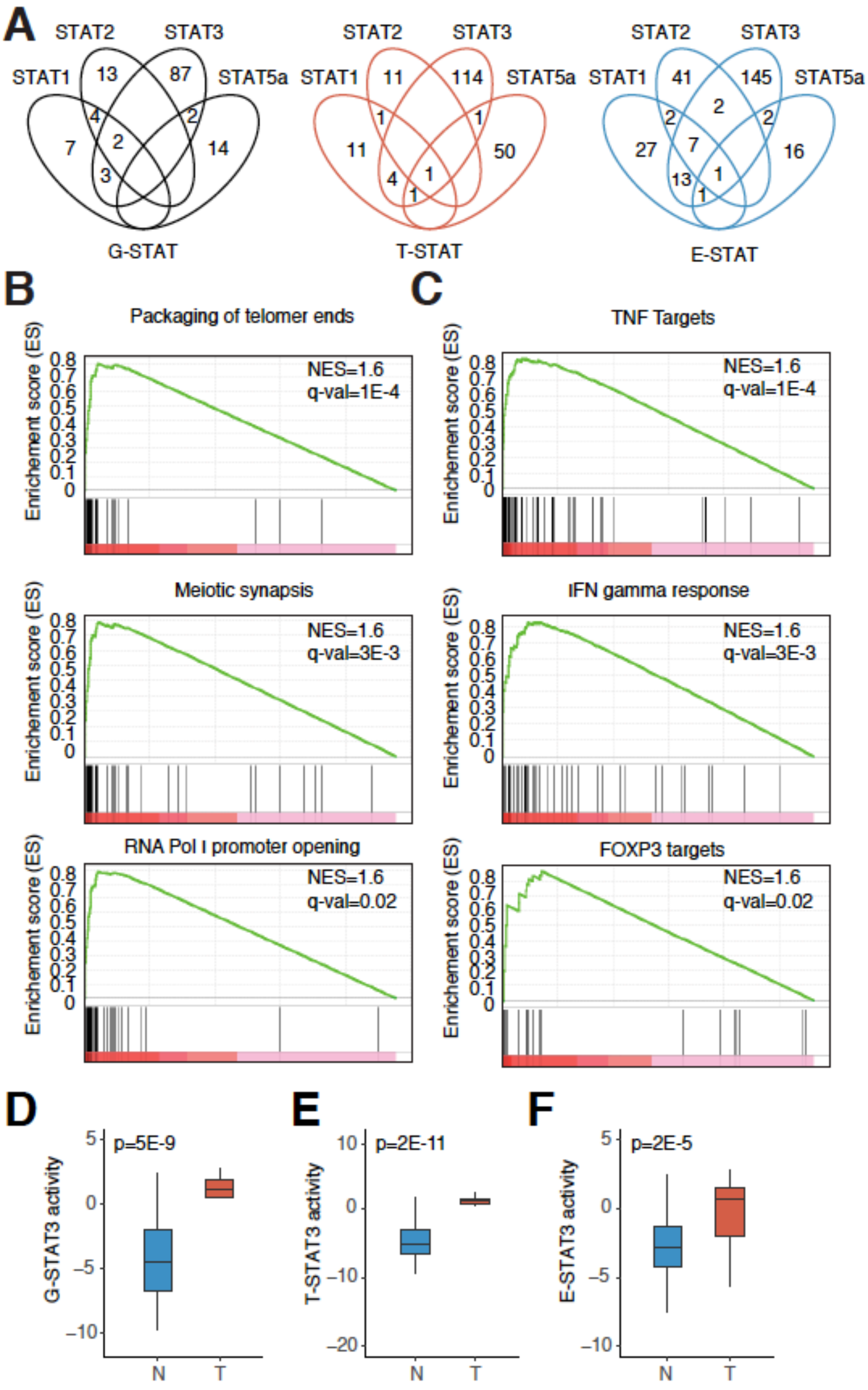
A
STAT1
STAT2
STAT3
STAT5a
G-STAT
T-STAT
E-STAT
B
C
Packaging of telomer ends
Meiotic synapsis
RNA Pol I promoter opening
TNF Targets
IFN gamma response
FOXP3 targets
Enrichement score (ES)
NES=1.6
q-val=1E-4
q-val=3E-3
q-val=0.02
D
E
F
p=5E-9
p=2E-11
p=2E-5
G-STAT3 activity
T-STAT3 activity
E-STAT3 activity
N
T

S2. STAT3 target gene specificity, Gene Set Enrichment Analysis of STAT3 signature and box plots of G-STAT3, T-STAT3, and E-STAT3 activity in dataset GSE28735. (A) Overlap between STAT1, STAT2, STAT3, and STAT5a target genes, stratified by compartment specificity. (B) Gene sets enriched in tumor-specific genes. (C) Gene sets enriched in tumor-specific genes. Comparison of normal pancreatic (N) and tumor (T) tissue for (D) G-STAT3 activity, (E) T-STAT3 activity, and (F) E-STAT3 activity (two-sided Wilcoxon test).

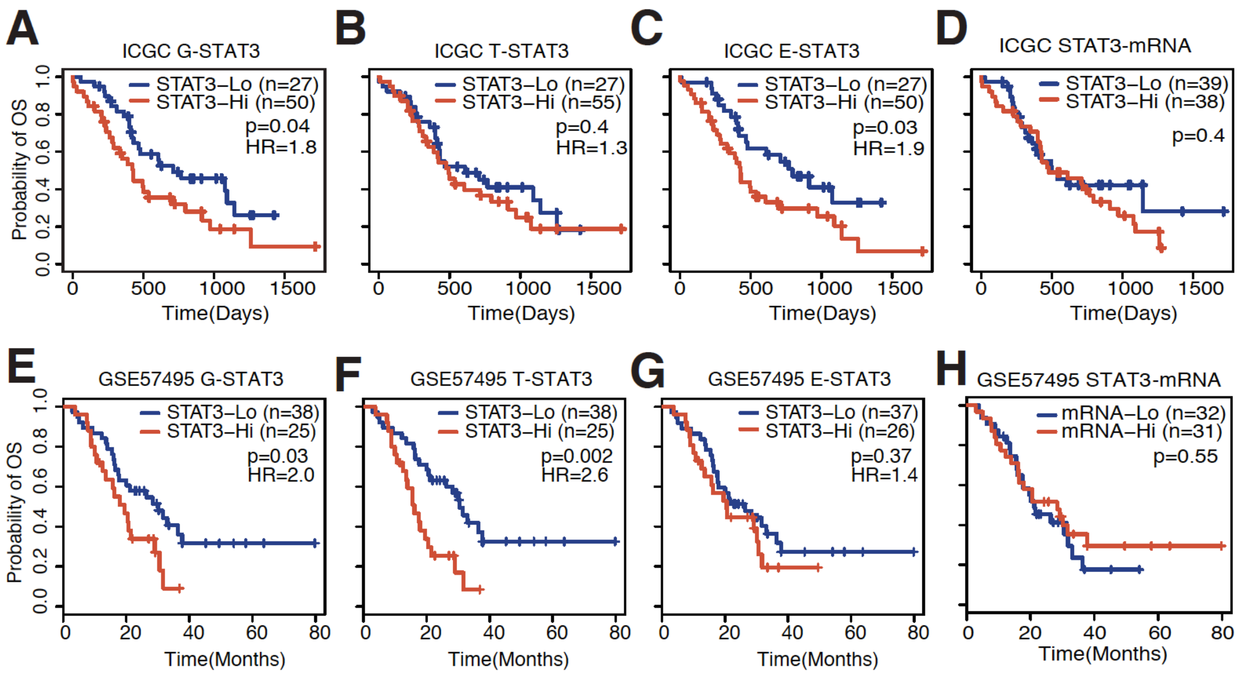

S3. Kaplan-Meier plots of STAT3 activity groups and survival in the ICGC and GSE57495 datasets. Kaplan-Meier plot depicting the survival probability over time for samples with high (red) and low (blue) STAT3 activity, (A) G-STAT3 activity, (B) T-STAT3 activity, (C) E-STAT3 activity in the IGCG dataset. (D) Kaplan-Meier plot depicting the survival probability over time for samples with high (red) and low (blue) STAT3 expression in the ICGC dataset. (E-H) Identical Kaplan-Meier plots for the GSE57495 dataset.

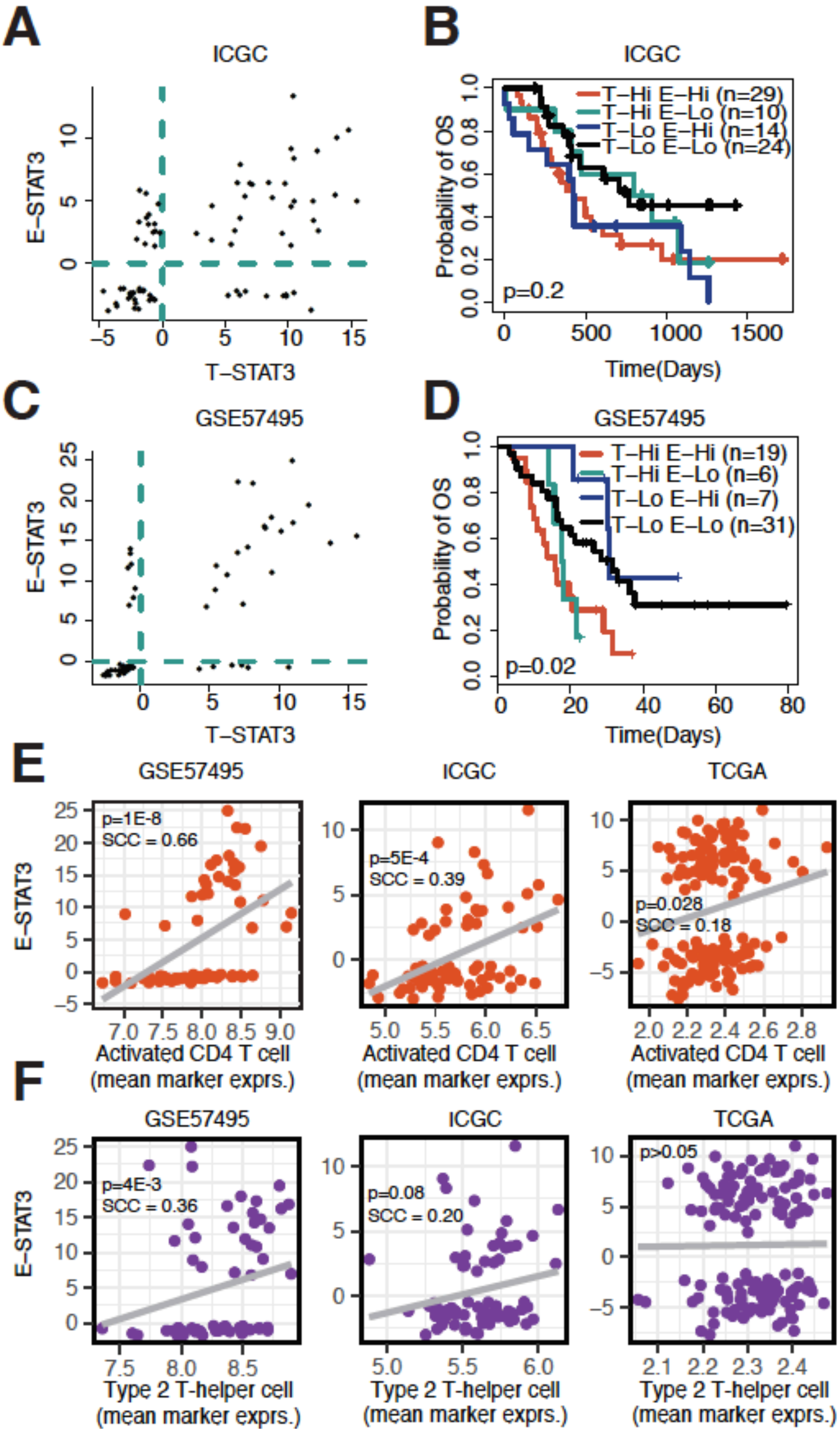

A
ICGC
E−STAT3
T−STAT3
B
ICGC
Probability of OS
T−Hi E−Hi (n=29)
T−Hi E−Lo (n=10)
T−Lo E−Hi (n=14)
T−Lo E−Lo (n=24)
p=0.2
Time(Days)
C
GSE57495
E−STAT3
T−STAT3
D
GSE57495
Probability of OS
T−Hi E−Hi (n=19)
T−Hi E−Lo (n=6)
T−Lo E−Hi (n=7)
T−Lo E−Lo (n=31)
p=0.02
Time(Days)
E
GSE57495
ICGC
TCGA
E−STAT3
p=1E-8
SCC = 0.66
p=5E-4
SCC = 0.39
p=0.028
SCC = 0.18
Activated CD4 T cell
(mean marker exprs.)
F
GSE57495
ICGC
TCGA
E−STAT3
p=4E-3
SCC = 0.36
p=0.08
SCC = 0.20
p>0.05
Type 2 T-helper cell
(mean marker exprs.)

S4. KM plots and CD4 T cell scores. (A) Distribution of T- and E-STAT3 scores in the ICGC dataset. (B) Kaplan-Meier plot comparing survival probabilities of T-STAT3-Hi/E-STAT3-Hi (n=29), T-STAT3-Hi/E-STAT3-Lo (n=10), T-STAT3-Lo/E-STAT3-Hi (n=14), and T-STAT3-Lo/E-STAT3-Lo (n=24) in ICGC dataset. (C) Distribution of T- and E-STAT3 scores in GSE57495 dataset. (D) Kaplan-Meier plot comparing survival probabilities of T-STAT3-Hi/E-STAT3-Hi (n=19), T-STAT3-Hi/E-STAT3-Lo (n=6), T-STAT3-Lo/E-STAT3-Hi (n=7), and T-STAT3-Lo/E-STAT3-Lo (n=31) in GSE57495 dataset. Correlation between E-STAT3 activity and average gene expression of marker genes for (E) activated CD4 T cells and (F) Type 2 T-helper cells.

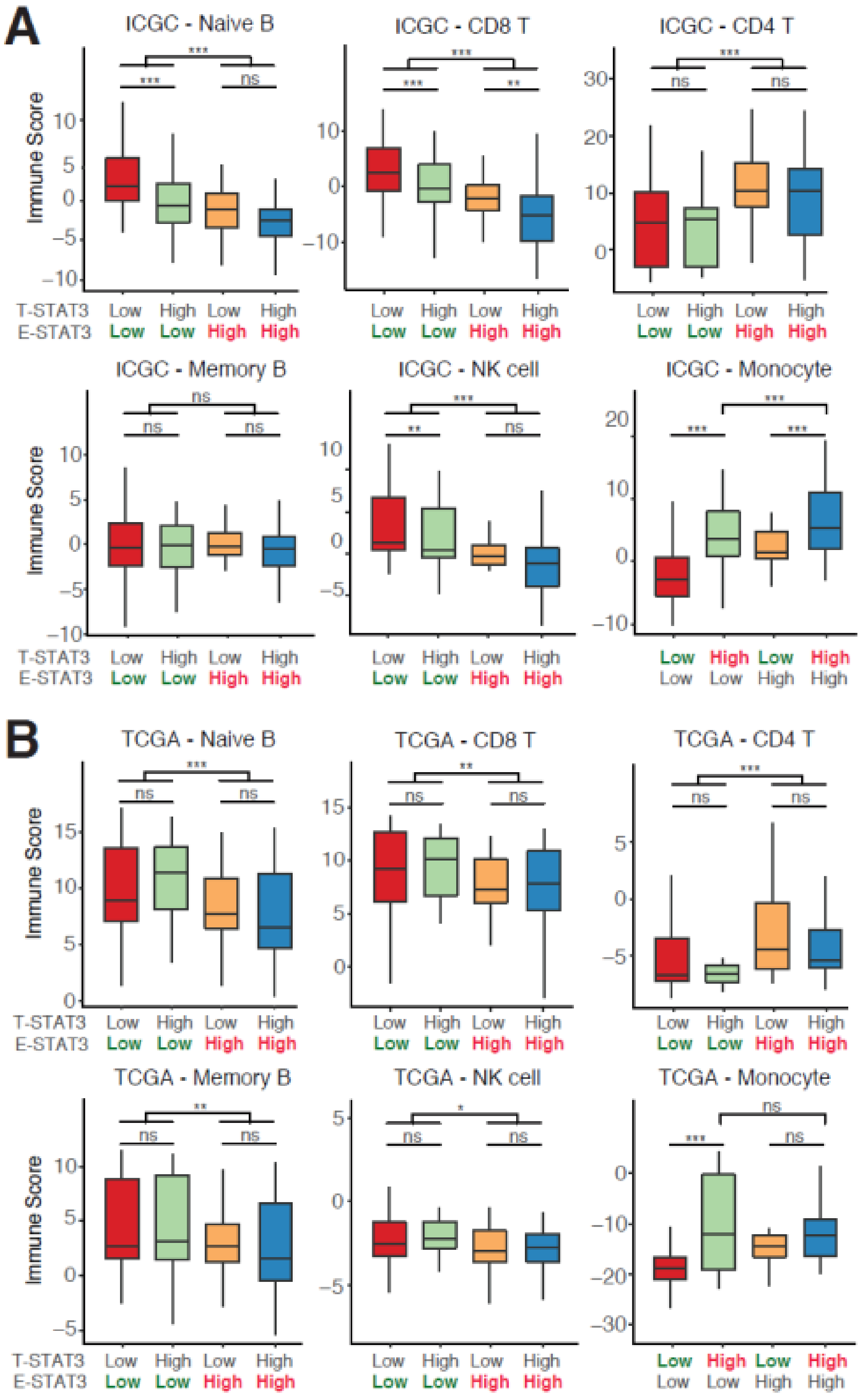

A
ICGC - Naive B
ICGC - CD8 T
ICGC - CD4 T
ICGC - Memory B
ICGC - NK cell
ICGC - Monocyte
B
TCGA - Naive B
TCGA - CD8 T
TCGA - CD4 T
TCGA - Memory B
TCGA - NK cell
TCGA - Monocyte
Immune Score
T-STAT3
E-STAT3
Low
High
ns
*
**
***

S5. STAT3 activity and association with tumor intrinsic characteristics. Boxplots showing (A) total mutation burden and (B) homologous recombination defects in four STAT3 groups in TCGA (two-sided Wilcoxon test).

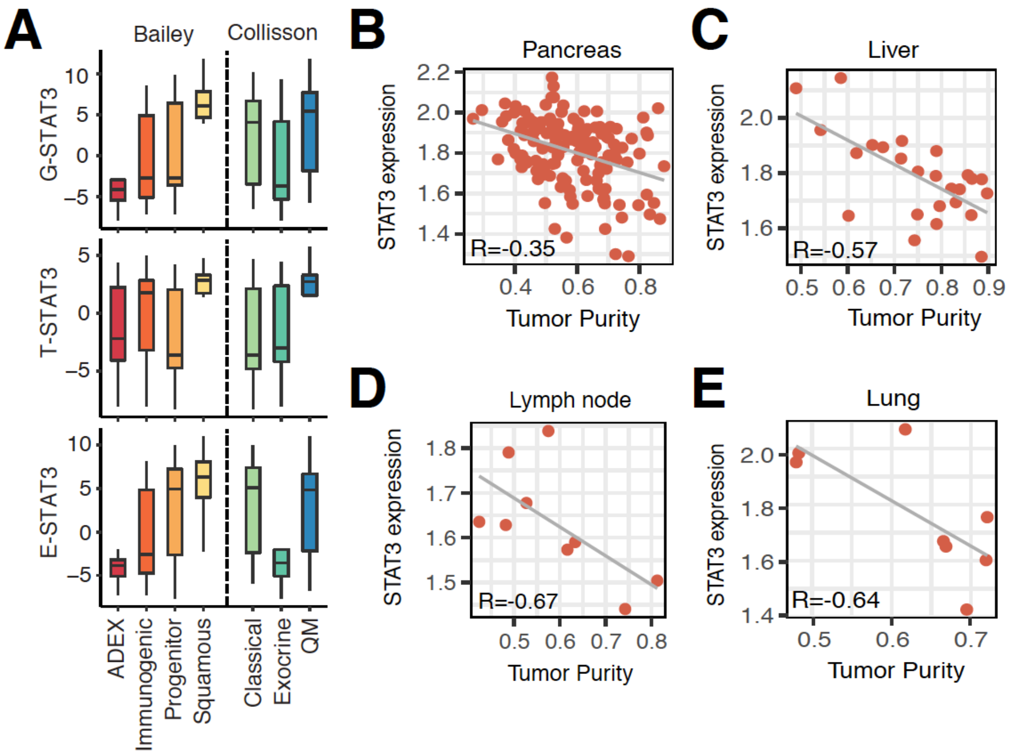

S6. Pancreatic cancer subtypes and STAT3 inference and STAT3 expression correlated with tumor purity. (A) G-,T- and E-STAT3 inference in pancreatic cancer subtypes identified by Bailey et al. [47] and Collisson et al. [48]. Outliers not indicated. STAT3 expression vs. tumor purity in (B) primary pancreatic tumor samples (SCC=-0.35, p=1E-5), (C) pancreatic metastasis to liver (SCC=-0.57, p=3E-3), (D) pancreatic metastasis to lymph node (SCC=-0.67, p=0.059), and (E) pancreatic metastasis to lung (SCC=-0.64, p=0.1).

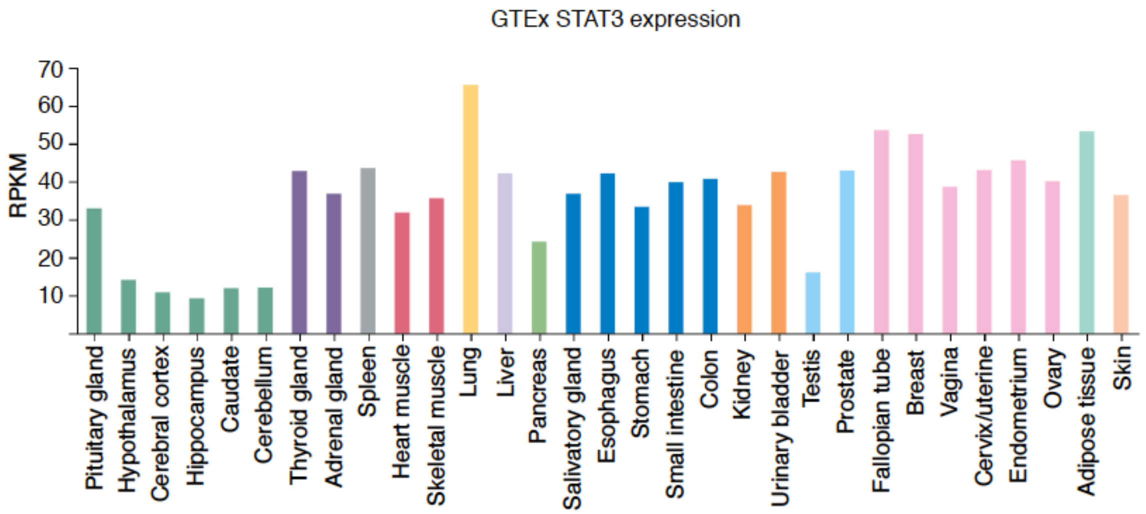

S7. STAT3 expression levels (RPKM) in normal tissues from GTEx. Adapted from proteinatlas.org/ENSG00000168610-STAT3/tissue.

Suppl. Table 1. Overview of pancreatic cancer datasets used.
Suppl. Table 2. Pancreatic cancer cell lines used from CCLE.
Suppl. Table 3. Thirty-five identified TFs in analysis.
Suppl. Table 4. Overview of pancreatic cancer datasets used.
Suppl. Table 5. GSEA results T-STAT3 and E-STAT3 weight profiles.
Suppl. Table 6. Description of 386 utilized STAT3 targets.

Suppl. Table 1. Overview of pancreatic cancer datasets used.

Dataset	GEO_Accession	# Samples	Paired	# Tumor included	# Normal included	Gender
Moffitt	GSE71729	340	Partially	206	134	No
Zhang	GSE28735	90	Yes	45	45	NO
Badea	GSE15471	78	Yes	39	39	No
Chen	GSE57495	63	No	63	0	No
ICGC_AU	NA	91	No	77	14	Yes
TCGA	NA	178	No	150	0	Yes

Suppl. Table 1
Overview of pancreatic cancer datasets used.

Age	Race	TNM (Stage)	OS	PMID
No	No	No	No	26343385
No	No	No	Yes	22363658, 23918603
No	No	No	No	19260470
No	No	Yes	Yes	26247463
Yes	No	Yes	Yes	https://icgc.org/Zuq
Yes	Yes	Yes	Yes	NA

Exluded samples	Exclusion Reason
All samples classified	
TCGA-FB-A7DR-01,	Did not
TCGA-2L-AAQM-01, "T	Neuroendocrine
TCGA-HZ-7918-01	Acinar cell carcinoma
TCGA-FB-AAPP-01, "TC	Intraductal papillary mucinous neoplasm
TCGA-HZ-A9TJ-06	Metastatic
TCGA-2J-AABP-01	Undifferentiated
TCGA-IB-7654-01	Systemic treatment given to the prior/other malignancy
TCGA-H6-8124-11, "TC	Adjacent normal pancreas
TCGA-F2-6880-01, "TC	Pseudonormal
TCGA-H6-A45N-11, "TC	Solid tissue normal

Suppl. Table 2. Pancreatic cancer cell lines used from CCLE.

**Cell lines**
MIAPACA2
CAPAN2
HS766T
PANC1005
ASPC1
L33
KP2
PATU8902
DANG
HUPT3
TCCPAN2
HUPT4
PSN1
PATU8988T
PANC0403
KCIMOH1
PK59
QGP1
PANC0327
HPAFII
CAPAN1
CFPAC1
PANC0213
BXPC3
KP3
KP4
PATU8988S
PL45
SW1990
PANC1
PANC0813
PK1
PANC0504
SUIT2
YAPC
SNU410
T3M4
PK45H
HPAC
SU8686
PANC0203
SNU324
SNU213

KLM1

Suppl. Table 2
Pancreatic cancer cell lines used from CCLE.

Suppl. Table 3. STAT3 Weight matrices

Gene names	G-STAT3	T-STAT3	E-STAT3
A1BG	0.0042101	0	0.0042101
A1CF	0.0106678	0.0106678	0
A2BP1	0.0051881	0	0
A2LD1	0.0038412	0.0038412	0
A2M	0.0373488	0	0.0373488
A2ML1	0.0035767	0	0
A4GALT	0.0241685	0	0.0241685
A4GNT	0.0068514	0	0
AAA1	0.0098406	0.0098406	0
AAAS	0.0834756	0.0834756	0
AACS	0.0074484	0.0074484	0
AACSL	0.0020468	0.0020468	0
AADAC	0.0194708	0	0
AADACL2	0.0153841	0	0
AADACL3	0.0073478	0	0.0073478
AADACL4	0.0039725	0	0.0039725
AADAT	0.0180321	0	0
AAGAB	0.0820218	0.0820218	0
AAK1	0.1062741	0	0.1062741
AAMP	0.0122004	0.0122004	0
AANAT	0.0290622	0	0
AARS	0.0552952	0	0
AARS2	0.0229349	0	0
AARSD1	0.0156876	0.0156876	0
AASDH	0.0721391	0	0.0721391
AASDHPPT	0.0765331	0	0.0765331
AASS	0.0871218	0	0.0871218
AATF	0.0831639	0.0831639	0
AATK	0.0003616	0.0003616	0
ABAT	0.0076963	0	0
ABCA1	0.0097457	0	0.0097457
ABCA10	0.0307123	0	0.0307123
ABCA11P	0.0255736	0	0
ABCA12	0.1499828	0	0
ABCA13	0.0086825	0	0
ABCA17P	0.0123112	0.0123112	0
ABCA2	0.0060026	0.0060026	0
ABCA3	0.0130791	0	0
ABCA4	0.0058137	0	0.0058137
ABCA5	0.0865559	0	0
ABCA6	0.0364939	0	0.0364939
ABCA7	0.012933	0.012933	0
ABCA8	0.0094706	0	0.0094706

ABCA9	0.0257998	0	0.0257998
ABCB1	0.0086382	0	0.0086382
ABCB10	0.0490323	0	0
ABCB11	0.0116124	0	0.0116124
ABCB4	0.0172838	0	0.0172838
ABCB5	0.0134981	0	0.0134981
ABCB6	0.0141393	0.0141393	0
ABCB7	0.0356476	0	0.0356476
ABCB8	0.0295485	0.0295485	0
ABCB9	0.050312	0.050312	0
ABCC1	0.1119515	0	0
ABCC10	0.0196105	0	0
ABCC11	0.0144451	0.0144451	0
ABCC12	0.0051933	0	0
ABCC13	0.0089083	0	0
ABCC2	0.7629071	0	0
ABCC3	0.051484	0.051484	0
ABCC4	0.0184157	0	0.0184157
ABCC5	0.0260225	0	0
ABCC6	3.60E-05	0	0
ABCC6P1	4.17E-05	4.17E-05	0
ABCC6P2	3.21E-05	3.21E-05	0
ABCC8	0.0028283	0	0.0028283
ABCC9	0.0131757	0	0.0131757
ABCD1	0.0039283	0	0
ABCD2	0.1433598	0	0.1433598
ABCD3	0.0721733	0.0721733	0
ABCD4	0.0224508	0.0224508	0
ABCE1	0.0691143	0	0.0691143
ABCF2	0.0195859	0	0
ABCF3	0.0203676	0	0
ABCG1	0.4025851	0	0.4025851
ABCG2	0.0049735	0	0.0049735
ABCG4	0.0019223	0	0.0019223
ABCG5	0.0020637	0.0020637	0
ABCG8	0.0019738	0	0
ABHD1	0.0037128	0	0
ABHD10	0.0898762	0	0
ABHD11	0.0224524	0.0224524	0
ABHD12	0.0137135	0.0137135	0
ABHD12B	0.0134974	0	0
ABHD13	0.0824578	0	0.0824578
ABHD14A	0.0359783	0.0359783	0
ABHD14B	0.0331136	0.0331136	0
ABHD15	0.009176	0	0.009176

ABHD2	0.0818137	0	0
ABHD3	0.0172808	0.0172808	0
ABHD4	0.0477294	0	0
ABHD5	0.2232325	0	0.2232325
ABHD6	0.0193484	0	0.0193484
ABHD8	0.0892501	0	0
ABI1	0.1399533	0	0
ABI2	0.0143275	0	0.0143275
ABI3	0.0269773	0	0.0269773
ABI3BP	0.0256258	0	0.0256258
ABL1	0.1099807	0	0.1099807
ABL2	0.7835745	0	0.7835745
ABLIM1	0.689972	0.689972	0
ABLIM2	0.0026842	0	0
ABLIM3	0.0934573	0.0934573	0
ABO	0.0037728	0.0037728	0
ABP1	0.0030945	0.0030945	0
ABR	0.0308393	0	0
ABRA	0.034197	0	0.034197
ABT1	0.1328287	0	0
ABTB1	0.0041605	0.0041605	0
ABTB2	0.0660537	0.0660537	0
ACAA1	0.0597406	0.0597406	0
ACAA2	0.0727909	0	0.0727909
ACACA	0.0767318	0.0767318	0
ACACB	0.005601	0	0.005601
ACAD10	0.0261233	0.0261233	0
ACAD11	0.0947679	0	0
ACAD8	0.0144869	0.0144869	0
ACAD9	0.0406058	0.0406058	0
ACADL	0.0203565	0	0.0203565
ACADM	0.104267	0	0.104267
ACADS	0.0088932	0.0088932	0
ACADSB	0.0360224	0	0.0360224
ACADVL	0.0343142	0.0343142	0
ACAN	0.0037679	0	0.0037679
ACAP1	0.0165961	0	0.0165961
ACAP2	0.0779499	0	0.0779499
ACAP3	0.0015711	0.0015711	0
ACAT1	0.0259699	0	0.0259699
ACAT2	0.0295897	0	0
ACBD3	0.0788425	0	0
ACBD4	0.0412766	0.0412766	0
ACBD5	0.0964465	0.0964465	0
ACBD6	0.3853059	0.3853059	0

ACBD7	0.0065188	0.0065188	0
ACCN1	0.0105137	0	0.0105137
ACCN2	0.0075873	0	0
ACCN3	0.0052553	0.0052553	0
ACCN4	0.0004378	0.0004378	0
ACCN5	0.0079992	0	0
ACCS	0.0252695	0.0252695	0
ACCSL	0.0057935	0	0.0057935
ACD	0.0061846	0.0061846	0
ACE	0.0029014	0.0029014	0
ACE2	0.0179014	0	0.0179014
ACER1	0.0012851	0	0.0012851
ACER2	0.0299715	0.0299715	0
ACER3	0.0460395	0	0.0460395
ACHE	0.0055128	0.0055128	0
ACIN1	0.1255517	0.1255517	0
ACLY	0.0208038	0	0.0208038
ACMSD	0.0038428	0	0
ACN9	0.0507866	0.0507866	0
ACO1	0.0404686	0	0.0404686
ACO2	0.0442671	0.0442671	0
ACOT1	0.0011844	0	0.0011844
ACOT11	0.0069461	0.0069461	0
ACOT12	0.0075828	0	0
ACOT13	0.1260134	0	0
ACOT2	0.0266397	0	0
ACOT4	0.0054273	0.0054273	0
ACOT6	0.0053991	0	0
ACOT7	0.0273845	0	0
ACOT8	0.054586	0.054586	0
ACOT9	0.0395541	0	0
ACOX1	0.1379602	0.1379602	0
ACOX2	0.013533	0	0.013533
ACOX3	0.0224509	0	0
ACOXL	0.0081083	0	0
ACP1	0.0261995	0.0261995	0
ACP2	0.066799	0	0.066799
ACP5	0.0147703	0	0.0147703
ACP6	0.1701249	0.1701249	0
ACPL2	0.0103552	0	0.0103552
ACPP	0.2244322	0	0.2244322
ACPT	0.0117594	0	0
ACR	0.0006295	0	0.0006295
ACRBP	0.0051923	0	0.0051923
ACRC	0.0058317	0	0

ACRV1	0.0057501	0	0
ACSBG1	0.0081007	0	0.0081007
ACSBG2	0.0075904	0	0
ACSF2	0.0507131	0	0
ACSF3	0.0038273	0.0038273	0
ACSL1	0.0292287	0	0.0292287
ACSL3	0.0691672	0.0691672	0
ACSL4	0.1779009	0	0.1779009
ACSL5	0.045769	0.045769	0
ACSL6	0.0113577	0	0.0113577
ACSM1	0.0075293	0.0075293	0
ACSM2A	5.34E-05	0	5.34E-05
ACSM2B	4.89E-05	0	0
ACSM3	0.0226345	0.0226345	0
ACSM4	0.0075186	0	0
ACSM5	0.0046168	0	0.0046168
ACSS1	0.0232247	0	0
ACSS2	0.0772844	0.0772844	0
ACSS3	0.0189326	0	0.0189326
ACTA1	0.0164062	0	0
ACTA2	0.418159	0	0.418159
ACTB	0.1198875	0	0.1198875
ACTBL2	0.4286499	0	0.4286499
ACTC1	0.0128883	0	0.0128883
ACTG1	0.0532488	0	0
ACTG2	0.0111467	0	0.0111467
ACTL6A	0.0517153	0.0517153	0
ACTL6B	0.0044284	0	0
ACTL7A	0.0115229	0	0
ACTL7B	0.0060783	0.0060783	0
ACTL8	0.0020961	0	0
ACTL9	0.0009597	0	0
ACTN1	0.0207577	0	0.0207577
ACTN2	0.0122744	0	0.0122744
ACTN3	0.0059487	0	0.0059487
ACTN4	0.0887219	0.0887219	0
ACTR10	0.0439077	0	0
ACTR1A	0.0756346	0	0.0756346
ACTR1B	0.0237644	0.0237644	0
ACTR2	0.0748887	0	0.0748887
ACTR3	0.3346185	0	0.3346185
ACTR3B	0.0162043	0.0162043	0
ACTR3C	0.4909498	0.4909498	0
ACTR5	0.0308208	0.0308208	0
ACTR6	0.0449997	0	0.0449997

ACTR8	0.0739196	0	0.0739196
ACTRT1	0.0049803	0	0
ACTRT2	0.0002493	0	0
ACVR1	0.0152076	0	0.0152076
ACVR1B	0.0411719	0.0411719	0
ACVR1C	0.008673	0	0.008673
ACVR2A	0.0209265	0	0.0209265
ACVR2B	0.0152557	0.0152557	0
ACVRL1	0.007783	0	0.007783
ACY1	0.0321573	0.0321573	0
ACY3	0.0260787	0.0260787	0
ACYP1	0.1958398	0.1958398	0
ACYP2	0.0690907	0	0
ADA	0.0805349	0	0.0805349
ADAD1	0.0036643	0.0036643	0
ADAD2	0.0034494	0	0
ADAL	0.0883167	0	0.0883167
ADAM10	0.0987314	0	0.0987314
ADAM11	0.000985	0	0
ADAM12	0.1661438	0	0.1661438
ADAM15	0.0245564	0.0245564	0
ADAM17	0.2393404	0	0.2393404
ADAM18	0.0099341	0	0.0099341
ADAM19	0.0440983	0	0.0440983
ADAM2	0.0129474	0.0129474	0
ADAM20	0.0129729	0	0
ADAM21	0.0114504	0	0
ADAM21P1	0.010251	0	0
ADAM22	0.0373922	0	0.0373922
ADAM23	0.0103579	0	0.0103579
ADAM28	0.0226745	0	0
ADAM29	0.021618	0	0.021618
ADAM30	0.0140157	0	0
ADAM32	0.0170231	0	0.0170231
ADAM33	0.0008177	0	0.0008177
ADAM3A	0.0024515	0	0
ADAM5P	0.0079441	0.0079441	0
ADAM6	0.0004242	0	0.0004242
ADAM7	0.009261	0	0.009261
ADAM8	0.0047733	0	0
ADAM9	0.4221284	0	0
ADAMDEC1	0.0419027	0	0.0419027
ADAMTS1	0.1014401	0	0.1014401
ADAMTS10	0.001241	0	0.001241
ADAMTS12	0.0308106	0	0.0308106

ADAMTS13	0.0361329	0.0361329	0
ADAMTS14	0.0058874	0	0.0058874
ADAMTS15	0.0034285	0	0.0034285
ADAMTS16	0.0102291	0	0.0102291
ADAMTS17	0.0856734	0	0.0856734
ADAMTS18	0.0044144	0	0.0044144
ADAMTS19	0.0096207	0	0
ADAMTS2	0.0004936	0	0.0004936
ADAMTS20	0.00779	0	0
ADAMTS3	0.0384147	0	0.0384147
ADAMTS4	0.224389	0	0.224389
ADAMTS5	0.0123242	0	0.0123242
ADAMTS6	0.1271347	0	0.1271347
ADAMTS7	0.0069851	0	0.0069851
ADAMTS8	0.008914	0	0.008914
ADAMTS9	0.0048742	0	0.0048742
ADAMTSL1	0.0216485	0	0.0216485
ADAMTSL2	0.0013865	0	0.0013865
ADAMTSL3	0.0048345	0	0.0048345
ADAMTSL4	0.0422731	0	0.0422731
ADAMTSL5	0.0019958	0.0019958	0
ADAP1	0.0019284	0.0019284	0
ADAP2	0.0035014	0	0.0035014
ADAR	0.4487858	0	0.4487858
ADARB1	0.0205833	0	0.0205833
ADARB2	0.0579159	0	0
ADAT1	0.0838754	0	0.0838754
ADAT2	0.0706405	0	0
ADAT3	0.1318333	0.1318333	0
ADC	0.0110749	0	0.0110749
ADCK1	0.0313927	0.0313927	0
ADCK2	0.0051696	0.0051696	0
ADCK4	0.5716375	0.5716375	0
ADCK5	0.004696	0.004696	0
ADCY1	0.0013532	0	0.0013532
ADCY10	0.1117075	0	0
ADCY2	0.0057013	0	0.0057013
ADCY3	0.0075222	0	0.0075222
ADCY4	0.0106484	0	0.0106484
ADCY5	0.0016933	0	0.0016933
ADCY6	0.0085072	0.0085072	0
ADCY7	0.004199	0	0.004199
ADCY8	0.0269507	0	0
ADCY9	0.0057179	0	0.0057179
ADCYAP1	0.0038414	0	0.0038414

ADCYAP1R1	0.0009061	0	0.0009061
ADD1	0.0230399	0	0.0230399
ADD2	0.0405479	0	0.0405479
ADD3	0.0451349	0	0.0451349
ADH1A	0.0049574	0	0.0049574
ADH1B	0.0096637	0	0.0096637
ADH1C	0.0024365	0	0
ADH4	0.0037865	0	0.0037865
ADH5	0.0345506	0	0.0345506
ADH6	0.0032052	0.0032052	0
ADH7	0.0058752	0	0
ADHFE1	0.0457287	0	0.0457287
ADI1	0.0175258	0	0
ADIG	0.0095727	0	0
ADIPOQ	0.0071561	0	0.0071561
ADIPOR1	0.2804267	0.2804267	0
ADIPOR2	0.0394592	0.0394592	0
ADK	0.1294857	0	0
ADM	0.2687838	0.2687838	0
ADM2	0.0015671	0.0015671	0
ADNP	0.0914885	0	0
ADNP2	0.0312911	0	0.0312911
ADO	0.0812117	0	0.0812117
ADORA1	0.0158085	0	0.0158085
ADORA2A	0.007059	0	0.007059
ADORA2B	0.0161746	0.0161746	0
ADORA3	0.0500578	0	0.0500578
ADPGK	0.0845382	0	0.0845382
ADPRH	0.0286224	0	0.0286224
ADPRHL1	0.0091174	0.0091174	0
ADPRHL2	0.00953	0	0
ADRA1A	0.0068797	0	0.0068797
ADRA1B	0.0735088	0	0
ADRA1D	0.0016774	0	0.0016774
ADRA2A	0.0022682	0	0.0022682
ADRA2B	0.001274	0	0.001274
ADRA2C	0.0002489	0	0
ADRB1	0.0102979	0	0
ADRB2	0.1706433	0	0.1706433
ADRB3	0.0022027	0	0.0022027
ADRBK1	0.0252735	0.0252735	0
ADRBK2	0.0487491	0	0.0487491
ADRM1	0.0131178	0.0131178	0
ADSL	0.0289376	0	0.0289376
ADSS	0.2810318	0.2810318	0

ADSSL1	0.0021932	0.0021932	0
AEBP1	0.0043155	0	0.0043155
AEBP2	0.1144688	0	0
AEN	0.2255868	0	0
AES	0.0060419	0.0060419	0
AFAP1	0.0048124	0	0.0048124
AFAP1L1	0.0248726	0	0.0248726
AFAP1L2	0.0135963	0	0
AFARP1	0.0353575	0.0353575	0
AFF1	0.5487997	0	0.5487997
AFF2	0.0047976	0	0.0047976
AFF3	0.011642	0	0.011642
AFF4	0.2692211	0	0.2692211
AFG3L1	0.0152165	0.0152165	0
AFG3L2	0.01388	0.01388	0
AFM	0.0063982	0.0063982	0
AFMID	0.0280881	0.0280881	0
AFP	0.0140816	0	0
AFTPH	0.1604341	0.1604341	0
AG2	0.0027113	0	0.0027113
AGA	0.0316478	0.0316478	0
AGAP1	0.0029988	0.0029988	0
AGAP11	0.028009	0	0.028009
AGAP2	0.0461699	0	0.0461699
AGAP3	0.0109579	0.0109579	0
AGAP4	1.53E-05	1.53E-05	0
AGAP5	0.0002071	0.0002071	0
AGAP6	5.85E-05	5.85E-05	0
AGAP7	0.0002907	0	0
AGAP8	1.53E-05	1.53E-05	0
AGBL1	0.0034301	0.0034301	0
AGBL2	0.037989	0.037989	0
AGBL3	0.1257067	0	0
AGBL4	0.0051004	0	0
AGBL5	0.1453223	0.1453223	0
AGFG1	0.1367438	0	0.1367438
AGFG2	0.1838122	0.1838122	0
AGGF1	0.0478565	0	0.0478565
AGK	0.0169764	0	0
AGL	0.0390107	0	0.0390107
AGMAT	0.0043572	0	0
AGPAT2	0.008647	0.008647	0
AGPAT3	0.02089	0	0
AGPAT4	0.0422749	0	0.0422749
AGPAT5	0.0858207	0	0

AGPAT6	0.0329318	0	0
AGPAT9	0.0142264	0.0142264	0
AGPHD1	0.0277307	0.0277307	0
AGPS	0.0685054	0	0.0685054
AGR2	0.070348	0.070348	0
AGR3	0.0150099	0.0150099	0
AGRN	0.006883	0.006883	0
AGRP	0.0341608	0	0.0341608
AGT	0.0274969	0	0.0274969
AGTPBP1	0.6303221	0	0.6303221
AGTR1	0.0463062	0	0.0463062
AGTR2	0.0024757	0	0.0024757
AGTRAP	0.3167806	0	0
AGXT	0.0008497	0.0008497	0
AGXT2	0.0287231	0.0287231	0
AGXT2L1	0.0043701	0.0043701	0
AGXT2L2	0.0534655	0	0
AHCTF1	0.0823748	0	0
AHCY	0.0614727	0.0614727	0
AHCYL1	0.1680285	0	0.1680285
AHCYL2	0.0965901	0	0
AHDC1	0.0181806	0	0
AHI1	0.132641	0	0
AHNAK	0.4515044	0	0.4515044
AHNAK2	0.0836547	0.0836547	0
AHR	0.2046051	0	0
AHRR	0.0037868	0	0.0037868
AHSA1	0.0618163	0.0618163	0
AHSA2	0.0376061	0.0376061	0
AHSG	0.0241835	0.0241835	0
AHSP	0.0015643	0	0.0015643
AICDA	0.0759835	0	0.0759835
AIDA	0.1607221	0	0.1607221
AIF1L	0.0047119	0	0.0047119
AIFM1	0.0646323	0.0646323	0
AIFM2	0.0211778	0.0211778	0
AIFM3	0.0012061	0.0012061	0
AIG1	0.6952557	0.6952557	0
AIM1	0.4010835	0	0.4010835
AIM1L	0.0038916	0.0038916	0
AIM2	1	0	1
AIMP1	0.1777934	0.1777934	0
AIMP2	0.036149	0.036149	0
AIP	0.0289337	0	0
AIPL1	0.004745	0.004745	0

AIRE	0.0043594	0	0.0043594
AJAP1	0.0020168	0	0
AK1	0.006347	0.006347	0
AK2	0.057983	0.057983	0
AK3	0.0619566	0	0.0619566
AK3L1	0.0240309	0.0240309	0
AK5	0.0075384	0	0.0075384
AK7	0.0200968	0.0200968	0
AKAP1	0.0500836	0.0500836	0
AKAP10	0.0271037	0	0.0271037
AKAP11	0.081001	0	0.081001
AKAP12	0.0760249	0	0.0760249
AKAP13	0.1636911	0	0.1636911
AKAP14	0.0013242	0.0013242	0
AKAP2	0.5001009	0	0.5001009
AKAP3	0.0545023	0	0.0545023
AKAP4	0.0262738	0	0
AKAP5	0.0404966	0	0.0404966
AKAP6	0.0205251	0	0.0205251
AKAP7	0.0079374	0	0.0079374
AKAP8	0.0287409	0.0287409	0
AKAP8L	0.0334968	0.0334968	0
AKAP9	0.0561965	0	0
AKD1	0.0737598	0	0
AKIRIN1	0.0391879	0	0.0391879
AKIRIN2	0.2619627	0	0.2619627
AKNA	0.0658119	0	0.0658119
AKNAD1	0.017657	0	0.017657
AKR1A1	0.0536698	0	0
AKR1B1	0.0393906	0	0.0393906
AKR1B10	0.1512778	0	0
AKR1B15	0.0486721	0	0
AKR1C1	0.0071919	0	0.0071919
AKR1C2	0.0350361	0	0.0350361
AKR1C3	0.0339014	0	0
AKR1C4	0.0092509	0	0
AKR1CL1	0.0023329	0	0
AKR1D1	0.020589	0	0
AKR1E2	0.0041264	0.0041264	0
AKR7A2	0.0084062	0.0084062	0
AKR7A3	0.0018673	0.0018673	0
AKR7L	0.0015157	0.0015157	0
AKT1	0.0018257	0.0018257	0
AKT1S1	0.1917429	0.1917429	0
AKT2	0.0519478	0	0.0519478

AKT3	0.1062784	0	0.1062784
AKTIP	0.0433306	0	0.0433306
ALAD	0.0335726	0	0
ALAS1	0.0911057	0.0911057	0
ALAS2	0.0030053	0	0.0030053
ALB	0.0075211	0.0075211	0
ALCAM	0.3531217	0	0.3531217
ALDH16A1	0.0445936	0.0445936	0
ALDH18A1	0.0423634	0.0423634	0
ALDH1A1	0.0134968	0	0
ALDH1A2	0.0192551	0	0.0192551
ALDH1A3	0.0110065	0	0.0110065
ALDH1B1	0.0361048	0	0.0361048
ALDH1L1	0.0061331	0	0
ALDH1L2	0.0161449	0	0.0161449
ALDH2	0.0480732	0	0
ALDH3A1	0.0386831	0.0386831	0
ALDH3A2	0.1067349	0.1067349	0
ALDH3B1	0.0661421	0.0661421	0
ALDH3B2	0.0072022	0.0072022	0
ALDH4A1	0.0120005	0	0
ALDH5A1	0.0149827	0	0
ALDH6A1	0.037942	0	0
ALDH7A1	0.2826038	0	0
ALDH8A1	0.016602	0	0.016602
ALDH9A1	0.0921348	0	0
ALDOA	0.0419637	0.0419637	0
ALDOB	0.0123644	0	0
ALDOC	0.0337048	0.0337048	0
ALG1	0.0115907	0.0115907	0
ALG10	0.0770848	0	0
ALG10B	0.1308797	0	0
ALG11	0.0188333	0	0
ALG12	0.003419	0.003419	0
ALG13	0.0170197	0	0
ALG14	0.0710002	0	0
ALG1L	0.0012892	0.0012892	0
ALG1L2	0.0005437	0.0005437	0
ALG2	0.0603535	0.0603535	0
ALG3	0.0448386	0.0448386	0
ALG5	0.0620953	0.0620953	0
ALG6	0.0400464	0	0
ALG8	0.0658076	0.0658076	0
ALG9	0.0682158	0.0682158	0
ALK	0.0036859	0	0.0036859

ALKBH1	0.0515807	0	0
ALKBH2	0.0741472	0	0
ALKBH3	0.0211068	0	0
ALKBH4	0.0029905	0.0029905	0
ALKBH5	0.0585153	0	0.0585153
ALKBH6	0.0073822	0.0073822	0
ALKBH7	0.0040462	0.0040462	0
ALKBH8	0.0635145	0	0.0635145
ALLC	0.0066626	0	0.0066626
ALMS1	0.0272049	0	0.0272049
ALMS1P	0.0029317	0	0
ALOX12	0.0045539	0.0045539	0
ALOX12B	0.0034099	0.0034099	0
ALOX12P2	0.0200457	0.0200457	0
ALOX15	0.002915	0	0.002915
ALOX15B	0.0115596	0	0.0115596
ALOX5	0.0057214	0	0.0057214
ALOX5AP	0.1467403	0	0.1467403
ALOXE3	0.2399197	0.2399197	0
ALPI	0.0007234	0	0
ALPK1	0.9696298	0	0
ALPK2	0.0303895	0	0.0303895
ALPK3	0.0097118	0	0.0097118
ALPL	0.0040297	0	0.0040297
ALPP	0.0017711	0	0
ALPPL2	0.0008898	0	0
ALS2	0.0284095	0	0
ALS2CL	0.0036267	0.0036267	0
ALS2CR11	0.002232	0	0.002232
ALS2CR12	0.0106006	0	0.0106006
ALS2CR4	0.0326136	0	0.0326136
ALS2CR8	0.0425598	0	0.0425598
ALX1	0.0336504	0	0.0336504
ALX3	0.00411	0	0
ALX4	0.0045393	0	0
AMAC1	0.0049221	0	0.0049221
AMAC1L2	0.0089649	0.0089649	0
AMAC1L3	0.0599559	0	0
AMACR	0.1402712	0	0
AMBN	0.0508372	0	0
AMBP	0.0034974	0	0
AMBRA1	0.0274092	0.0274092	0
AMD1	0.2036451	0	0.2036451
AMDHD1	0.0155501	0	0.0155501
AMDHD2	0.003077	0	0

AMELX	0.0113165	0	0
AMELY	1.53E-05	0	0
AMFR	0.0602955	0	0
AMH	0.0014776	0.0014776	0
AMHR2	0.0844128	0	0.0844128
AMICA1	0.0544268	0	0.0544268
AMIGO1	0.0332857	0	0.0332857
AMIGO2	0.3456462	0	0
AMIGO3	0.0067166	0.0067166	0
AMMECR1	0.0426415	0	0
AMMECR1L	0.0081494	0	0.0081494
AMN	0.0027689	0.0027689	0
AMN1	0.2445267	0.2445267	0
AMOT	0.0099391	0	0
AMOTL1	0.0581918	0	0.0581918
AMOTL2	0.0326418	0	0.0326418
AMPD1	0.0212411	0	0.0212411
AMPD2	0.0407646	0	0.0407646
AMPD3	0.1403416	0	0.1403416
AMPH	0.0045044	0	0.0045044
AMT	0.0307528	0.0307528	0
AMTN	0.4123155	0	0.4123155
AMY2A	0.0005291	0	0
AMY2B	0.0140416	0	0
AMZ1	0.0013016	0	0.0013016
AMZ2	0.0448249	0	0.0448249
ANAPC1	0.0089707	0	0.0089707
ANAPC10	0.0693546	0	0
ANAPC11	0.0063948	0.0063948	0
ANAPC13	0.4867285	0	0
ANAPC16	0.0799707	0.0799707	0
ANAPC2	0.013633	0.013633	0
ANAPC4	0.0298252	0	0
ANAPC5	0.0290765	0	0
ANAPC7	0.0233412	0.0233412	0
ANG	0.2852597	0.2852597	0
ANGEL1	0.0194573	0	0.0194573
ANGEL2	0.1982383	0	0
ANGPT1	0.093807	0	0.093807
ANGPT2	0.0124399	0	0.0124399
ANGPT4	0.0014145	0	0.0014145
ANGPTL1	0.0432867	0	0.0432867
ANGPTL2	0.0783821	0	0.0783821
ANGPTL3	0.0157727	0	0
ANGPTL4	0.2477175	0	0

ANGPTL5	0.0661793	0	0.0661793
ANGPTL6	0.0537968	0	0.0537968
ANGPTL7	0.0060046	0	0.0060046
ANK1	0.0388928	0.0388928	0
ANK2	0.031974	0	0.031974
ANK3	0.0749749	0	0
ANKAR	0.0233072	0	0
ANKDD1A	0.0073518	0	0.0073518
ANKFN1	0.0240535	0	0
ANKFY1	0.0239197	0	0.0239197
ANKH	0.0687274	0	0.0687274
ANKHD1	0.5406255	0	0
ANKHD1-EIF4	0.5406255	0	0
ANKIB1	0.0780734	0	0.0780734
ANKK1	0.0136373	0.0136373	0
ANKLE1	0.0036392	0	0.0036392
ANKLE2	0.0811813	0	0
ANKMY1	0.0167243	0.0167243	0
ANKMY2	0.1143569	0.1143569	0
ANKRA2	0.0598182	0.0598182	0
ANKRD1	0.1431036	0	0.1431036
ANKRD10	0.2641125	0.2641125	0
ANKRD11	0.0686135	0	0
ANKRD12	0.1479183	0	0.1479183
ANKRD13A	0.11823	0	0.11823
ANKRD13B	0.0107469	0.0107469	0
ANKRD13C	0.1230524	0	0.1230524
ANKRD13D	0.0098662	0.0098662	0
ANKRD16	0.0179689	0.0179689	0
ANKRD17	0.1147481	0	0.1147481
ANKRD19	0.0122584	0.0122584	0
ANKRD2	0.016217	0	0.016217
ANKRD20A4	1.77E-05	0	0
ANKRD20B	0.0020577	0.0020577	0
ANKRD22	0.2049727	0.2049727	0
ANKRD23	0.002882	0.002882	0
ANKRD24	0.0070874	0.0070874	0
ANKRD26	0.013611	0	0.013611
ANKRD26P1	0.0049418	0	0.0049418
ANKRD27	0.0061762	0.0061762	0
ANKRD28	0.0900471	0	0.0900471
ANKRD29	0.0189169	0	0.0189169
ANKRD30A	0.0014303	0	0.0014303
ANKRD30B	0.0023691	0	0.0023691
ANKRD31	0.0234174	0	0

ANKRD32	0.1205323	0	0.1205323
ANKRD33	0.0138821	0	0
ANKRD34A	0.1465083	0	0
ANKRD34B	0.006387	0.006387	0
ANKRD34C	0.0053439	0	0.0053439
ANKRD35	0.0481559	0	0.0481559
ANKRD36	0.0012368	0.0012368	0
ANKRD36B	0.0009598	0	0
ANKRD36BP1	0.0329304	0	0.0329304
ANKRD37	0.093129	0.093129	0
ANKRD39	0.0185381	0.0185381	0
ANKRD40	0.0825762	0	0.0825762
ANKRD42	0.1664977	0.1664977	0
ANKRD43	0.011931	0.011931	0
ANKRD44	0.0540593	0	0.0540593
ANKRD45	0.0228618	0	0.0228618
ANKRD46	0.562999	0	0
ANKRD49	0.0810795	0	0
ANKRD5	0.010132	0.010132	0
ANKRD50	0.0326584	0	0.0326584
ANKRD52	0.0113937	0	0
ANKRD53	0.0569475	0	0.0569475
ANKRD54	0.021229	0.021229	0
ANKRD55	0.0044073	0	0.0044073
ANKRD56	0.0070356	0.0070356	0
ANKRD57	0.2257089	0	0
ANKRD58	0.0062945	0	0.0062945
ANKRD6	0.0072409	0	0.0072409
ANKRD7	0.0155043	0	0.0155043
ANKRD9	0.0026825	0.0026825	0
ANKS1A	0.071609	0	0.071609
ANKS1B	0.0195015	0	0.0195015
ANKS3	0.0127501	0.0127501	0
ANKS4B	0.0064496	0.0064496	0
ANKS6	0.021334	0.021334	0
ANKZF1	0.0155161	0.0155161	0
ANLN	0.1621997	0.1621997	0
ANO1	0.0018777	0	0
ANO10	0.0293704	0	0
ANO2	0.0055227	0	0.0055227
ANO3	0.0429009	0	0
ANO4	0.0092407	0	0.0092407
ANO5	0.0095069	0.0095069	0
ANO6	0.1403739	0	0.1403739
ANO7	0.0008082	0.0008082	0

ANO8	0.0511119	0.0511119	0
ANO9	0.0043689	0.0043689	0
ANP32A	0.092814	0.092814	0
ANP32B	0.0514284	0.0514284	0
ANP32C	0.010463	0	0
ANP32D	0.0049581	0	0
ANP32E	0.2439065	0	0.2439065
ANPEP	0.0205608	0	0.0205608
ANTXR1	0.0335642	0	0.0335642
ANTXR2	0.1468315	0	0
ANTXRL	0.0086768	0	0
ANUBL1	0.0075934	0	0.0075934
ANXA1	0.9005784	0	0.9005784
ANXA10	0.0570184	0.0570184	0
ANXA11	0.1282242	0.1282242	0
ANXA13	0.0418698	0	0
ANXA2	0.6163821	0.6163821	0
ANXA2P1	0.0066818	0	0
ANXA2P2	0.0423376	0.0423376	0
ANXA2P3	0.0063419	0	0
ANXA3	0.2231639	0.2231639	0
ANXA4	0.0608045	0	0
ANXA5	0.1718504	0	0.1718504
ANXA6	0.0653808	0	0.0653808
ANXA7	0.1572835	0	0.1572835
ANXA8L2	2.31E-05	0	0
ANXA9	0.0277768	0	0
AOAH	0.006845	0	0.006845
AOC2	0.014587	0	0.014587
AOC3	0.0078251	0	0.0078251
AOX1	0.0181698	0	0.0181698
AOX2P	0.0123161	0.0123161	0
AP1AR	0.0605193	0.0605193	0
AP1B1	0.0146624	0	0.0146624
AP1G1	0.1237945	0	0
AP1G2	0.0150318	0.0150318	0
AP1M1	0.0211502	0	0.0211502
AP1M2	0.0053367	0.0053367	0
AP1S1	0.0085894	0.0085894	0
AP1S2	0.086366	0	0.086366
AP1S3	0.0164699	0.0164699	0
AP2A1	0.4069347	0	0
AP2A2	0.0028568	0	0
AP2B1	0.1958538	0.1958538	0
AP2M1	0.0406333	0	0

AP2S1	0.0516744	0.0516744	0
AP3B1	0.0686993	0	0.0686993
AP3B2	0.0033729	0	0.0033729
AP3D1	0.0331001	0.0331001	0
AP3M1	0.1174657	0	0
AP3M2	0.0781136	0.0781136	0
AP3S1	0.0999674	0	0.0999674
AP3S2	0.077082	0.077082	0
AP4B1	0.0989355	0.0989355	0
AP4E1	0.0960375	0	0.0960375
AP4M1	0.0145283	0.0145283	0
AP4S1	0.2332186	0	0.2332186
APAF1	0.2088676	0	0
APBA1	0.005696	0	0.005696
APBA2	0.0004438	0	0.0004438
APBA3	0.016896	0.016896	0
APBB1	0.0056883	0	0.0056883
APBB1IP	0.0336235	0	0.0336235
APBB2	0.156971	0	0.156971
APBB3	0.1421073	0	0
APC	0.0581447	0	0.0581447
APC2	0.026612	0.026612	0
APCDD1	0.0068723	0	0.0068723
APCDD1L	0.0035001	0	0.0035001
APCS	0.0496874	0	0
APEH	0.0116925	0.0116925	0
APEX1	0.1582334	0	0
APEX2	0.0233183	0	0
APH1A	0.0529066	0.0529066	0
APH1B	0.0215935	0	0.0215935
API5	0.0199659	0	0.0199659
APIP	0.1132704	0.1132704	0
APITD1	0.0173324	0.0173324	0
APLF	0.1003642	0	0.1003642
APLN	0.0029916	0	0
APLNR	0.0019354	0	0.0019354
APLP1	0.0010548	0	0.0010548
APLP2	0.0211345	0.0211345	0
APOA1BP	0.1383816	0.1383816	0
APOA2	0.0363265	0.0363265	0
APOA4	0.0011971	0	0
APOA5	0.04348	0.04348	0
APOB	0.0052819	0	0
APOB48R	0.0103836	0	0.0103836
APOBEC1	0.0053925	0.0053925	0

APOBEC2	0.0058675	0	0.0058675
APOBEC3A	0.0063764	0	0.0063764
APOBEC3B	0.0485696	0	0
APOBEC3C	0.0058422	0	0.0058422
APOBEC3D	0.0017409	0	0.0017409
APOBEC3F	0.0012552	0	0.0012552
APOBEC3G	0.0061153	0	0.0061153
APOBEC3H	0.0167692	0	0.0167692
APOBEC4	0.0296379	0	0
APOC1	0.0017082	0	0.0017082
APOC1P1	0.0015003	0	0.0015003
APOC2	0.0244865	0	0.0244865
APOC3	0.0012765	0.0012765	0
APOC4	0.0125393	0	0.0125393
APOD	0.010026	0	0.010026
APOE	0.0027444	0	0.0027444
APOF	0.298236	0	0
APOH	0.0062558	0	0.0062558
APOL1	0.0434399	0	0
APOL2	0.3121213	0	0.3121213
APOL3	0.0444609	0	0.0444609
APOL4	0.0061057	0	0.0061057
APOL5	0.001568	0	0
APOL6	0.2859343	0	0.2859343
APOLD1	0.3432079	0	0.3432079
APOO	0.1092088	0.1092088	0
APOOL	0.0254898	0	0.0254898
APP	0.0379583	0	0.0379583
APPBP2	0.0487033	0	0.0487033
APPL1	0.1223847	0	0.1223847
APPL2	0.0563044	0	0
APRT	0.0094434	0.0094434	0
APTX	0.0594197	0.0594197	0
AQP1	0.0010712	0	0.0010712
AQP10	0.0163102	0.0163102	0
AQP11	0.0152536	0.0152536	0
AQP12A	6.62E-05	0	0
AQP12B	6.99E-05	0	0
AQP2	0.0175911	0	0
AQP3	0.0282024	0.0282024	0
AQP4	0.0073753	0	0.0073753
AQP5	0.0076578	0	0
AQP6	0.0034026	0.0034026	0
AQP7	0.0053741	0	0
AQP7P1	0.0070597	0	0.0070597

AQP7P3	1.53E-05	0	1.53E-05
AQP8	0.0039723	0	0.0039723
AQP9	0.0169817	0	0.0169817
AQPEP	0.0051489	0	0.0051489
AQR	0.0410781	0	0.0410781
AR	0.0095554	0	0.0095554
ARAF	0.0522149	0.0522149	0
ARAP1	0.0233772	0	0.0233772
ARAP2	0.0776188	0	0
ARAP3	0.0059653	0	0
ARC	0.0008321	0	0.0008321
ARCN1	0.0383675	0	0
ARF1	0.0692838	0.0692838	0
ARF3	0.089371	0	0
ARF4	0.1971644	0	0.1971644
ARF5	0.0110119	0.0110119	0
ARF6	0.20235	0	0
ARFGAP1	0.0032985	0.0032985	0
ARFGAP2	0.0469612	0.0469612	0
ARFGAP3	0.0235567	0	0
ARFGEF1	0.0166125	0.0166125	0
ARFGEF2	0.0553309	0.0553309	0
ARFIP1	0.0566622	0.0566622	0
ARFIP2	0.0543358	0.0543358	0
ARFRP1	0.0526083	0.0526083	0
ARG1	0.0095353	0	0.0095353
ARG2	0.0462273	0	0.0462273
ARGFX	0.0053469	0	0
ARGFXP2	0.0153059	0	0
ARGLU1	0.5556005	0.5556005	0
ARHGAP1	0.0400107	0	0.0400107
ARHGAP10	0.0612088	0	0.0612088
ARHGAP11A	0.0585603	0.0585603	0
ARHGAP11B	0.0719158	0	0
ARHGAP12	0.0495489	0.0495489	0
ARHGAP15	0.0337717	0	0.0337717
ARHGAP17	0.1476464	0	0.1476464
ARHGAP18	0.0460306	0	0.0460306
ARHGAP19	0.0219026	0	0.0219026
ARHGAP20	0.0115746	0	0.0115746
ARHGAP21	0.0912129	0	0
ARHGAP22	0.0130022	0	0.0130022
ARHGAP23	0.01443	0	0.01443
ARHGAP24	0.1147496	0	0.1147496
ARHGAP25	0.0607969	0	0.0607969

ARHGAP26	0.035359	0	0
ARHGAP27	0.0205989	0.0205989	0
ARHGAP28	0.0051004	0	0.0051004
ARHGAP29	0.0397816	0	0.0397816
ARHGAP30	0.0334298	0	0.0334298
ARHGAP31	0.0373896	0	0.0373896
ARHGAP32	0.2385303	0.2385303	0
ARHGAP33	0.0025179	0.0025179	0
ARHGAP36	0.0015209	0	0.0015209
ARHGAP4	0.0044255	0	0.0044255
ARHGAP5	0.1125342	0.1125342	0
ARHGAP6	0.0133129	0	0.0133129
ARHGAP8	0.0044587	0.0044587	0
ARHGAP9	0.0206835	0	0.0206835
ARHGDIA	0.0263663	0.0263663	0
ARHGDIB	0.1671634	0	0.1671634
ARHGDIG	0.0002924	0	0
ARHGEF1	0.0284415	0	0
ARHGEF10	0.0113135	0	0.0113135
ARHGEF10L	0.0035663	0.0035663	0
ARHGEF11	0.0533958	0.0533958	0
ARHGEF12	0.2472329	0	0
ARHGEF15	0.0017093	0	0.0017093
ARHGEF16	0.0028359	0.0028359	0
ARHGEF17	0.0170044	0	0.0170044
ARHGEF18	0.1251133	0.1251133	0
ARHGEF19	0.0072806	0	0
ARHGEF2	0.0996067	0	0
ARHGEF3	0.1127449	0	0.1127449
ARHGEF4	0.0026857	0	0
ARHGEF5	3.09E-05	3.09E-05	0
ARHGEF6	0.0092646	0	0.0092646
ARHGEF7	0.0752174	0	0
ARHGEF9	0.012055	0	0.012055
ARID1A	0.2490233	0	0.2490233
ARID1B	0.0847527	0	0.0847527
ARID2	0.244125	0	0
ARID3A	0.006588	0	0
ARID3B	0.1242005	0	0
ARID3C	0.0119402	0.0119402	0
ARID4A	0.0981277	0	0.0981277
ARID4B	0.7151391	0	0.7151391
ARID5A	0.2808328	0	0.2808328
ARID5B	0.9201251	0	0.9201251
ARIH1	0.3108571	0	0.3108571

ARIH2	0.0354516	0	0
ARL1	0.0308041	0	0
ARL10	0.0136867	0	0.0136867
ARL11	0.0070648	0	0.0070648
ARL13A	0.0049003	0	0
ARL13B	0.074586	0	0.074586
ARL14	0.1646983	0.1646983	0
ARL15	0.0525506	0	0.0525506
ARL16	0.0054287	0.0054287	0
ARL17A	1.65E-05	0	0
ARL17B	1.54E-05	0	0
ARL2	0.0225135	0	0
ARL2BP	0.0769993	0	0.0769993
ARL3	0.0453952	0	0
ARL4A	0.0705664	0	0
ARL4C	0.01068	0	0.01068
ARL4D	0.0374156	0	0.0374156
ARL5A	0.0333075	0	0.0333075
ARL5B	0.3074781	0	0
ARL5C	0.0054612	0	0
ARL6	0.0303619	0	0.0303619
ARL6IP1	0.0838573	0.0838573	0
ARL6IP4	0.0066745	0.0066745	0
ARL6IP5	0.6093635	0	0.6093635
ARL6IP6	0.0662703	0.0662703	0
ARL8A	0.0224998	0.0224998	0
ARL8B	0.0429759	0	0.0429759
ARL9	0.0029468	0	0.0029468
ARMC1	0.1341262	0	0.1341262
ARMC10	0.1113026	0.1113026	0
ARMC2	0.0229135	0	0.0229135
ARMC3	0.0105948	0.0105948	0
ARMC4	0.0033424	0	0
ARMC5	0.1121182	0.1121182	0
ARMC6	0.0134539	0.0134539	0
ARMC7	0.0180126	0.0180126	0
ARMC8	0.0619888	0	0.0619888
ARMC9	0.0149572	0	0.0149572
ARMCX1	0.0006408	0	0.0006408
ARMCX2	0.0053206	0	0.0053206
ARMCX3	0.0061683	0	0.0061683
ARMCX5	0.0140672	0.0140672	0
ARMCX6	0.0042699	0	0
ARMS2	0.0032105	0	0
ARNT	0.3730375	0	0.3730375

ARNT2	0.0043455	0	0.0043455
ARNTL	0.0349713	0	0.0349713
ARNTL2	0.8468637	0	0
ARPC1A	0.0639444	0.0639444	0
ARPC1B	0.0485213	0	0
ARPC2	0.0395169	0	0.0395169
ARPC3	0.0457516	0	0
ARPC4	0.09142	0	0
ARPC5	0.2783431	0	0
ARPC5L	0.6625458	0.6625458	0
ARPM1	0.4214275	0	0
ARPP19	0.1101252	0	0.1101252
ARPP21	0.0174218	0	0.0174218
ARR3	0.0039117	0	0
ARRB1	0.0095451	0	0.0095451
ARRB2	0.0261313	0	0.0261313
ARRDC1	0.0074922	0.0074922	0
ARRDC2	0.0105798	0.0105798	0
ARRDC3	0.4482562	0	0.4482562
ARRDC4	0.0238715	0	0
ARRDC5	0.075392	0	0.075392
ARSA	0.0078984	0.0078984	0
ARSB	0.1037887	0	0.1037887
ARSD	0.0018608	0.0018608	0
ARSE	0.0023191	0.0023191	0
ARSF	0.0010577	0	0
ARSG	0.0510931	0	0.0510931
ARSH	0.0021048	0	0
ARSI	0.0198901	0	0.0198901
ARSJ	0.0276246	0	0.0276246
ARSK	0.112748	0	0.112748
ART1	0.0024491	0	0.0024491
ART3	0.0213797	0	0.0213797
ART4	0.0788033	0	0.0788033
ART5	0.0025721	0	0.0025721
ARTN	0.0131922	0	0
ARV1	0.1259506	0.1259506	0
ARVCF	0.0019761	0.0019761	0
ARX	0.0014427	0	0
AS3MT	0.0059247	0	0.0059247
ASAH1	0.0863268	0	0.0863268
ASAH2	0.007882	0	0
ASAH2B	0.0068706	0	0
ASAM	0.0276564	0	0.0276564
ASAP1	0.0305535	0	0.0305535

ASAP1IT1	0.092504	0	0
ASAP2	0.0140415	0.0140415	0
ASAP3	0.0167126	0	0.0167126
ASB1	0.0107605	0	0.0107605
ASB10	0.0017352	0	0.0017352
ASB11	0.0122694	0	0.0122694
ASB12	0.0032902	0.0032902	0
ASB13	0.0053821	0	0.0053821
ASB14	0.0193639	0	0
ASB15	0.012202	0.012202	0
ASB16	0.0019365	0	0
ASB17	0.0075991	0	0
ASB18	0.0014616	0	0
ASB2	0.051215	0	0.051215
ASB3	0.0580222	0	0.0580222
ASB4	0.077069	0	0
ASB5	0.0766851	0	0.0766851
ASB6	0.0103886	0.0103886	0
ASB7	0.7051862	0	0.7051862
ASB8	0.076462	0	0.076462
ASB9	0.0165577	0	0.0165577
ASCC1	0.077038	0	0.077038
ASCC2	0.0634196	0.0634196	0
ASCC3	0.0740047	0	0.0740047
ASCL1	0.0214089	0	0
ASCL2	0.0006742	0	0.0006742
ASCL3	0.0269037	0	0
ASCL4	0.011522	0	0.011522
ASF1A	0.1011441	0	0.1011441
ASF1B	0.0534168	0.0534168	0
ASFMR1	0.0132485	0.0132485	0
ASGR1	0.0032209	0	0.0032209
ASGR2	0.0047938	0	0.0047938
ASH1L	0.0847754	0	0
ASH2L	0.0286229	0	0.0286229
ASIP	0.0118847	0	0
ASL	0.0027105	0.0027105	0
ASNA1	0.0971897	0	0
ASNS	0.0312098	0.0312098	0
ASNSD1	0.0582563	0	0.0582563
ASPA	0.0099005	0	0.0099005
ASPDH	0.0029359	0	0
ASPG	0.002339	0	0
ASPH	1	1	0
ASPHD1	0.0038529	0.0038529	0

ASPHD2	0.0105599	0.0105599	0
ASPM	0.3229228	0.3229228	0
ASPN	0.0387436	0	0.0387436
ASPRV1	0.0026093	0	0.0026093
ASPSCR1	0.0079218	0.0079218	0
ASRGL1	0.0232111	0	0
ASS1	0.0324289	0.0324289	0
ASTE1	0.0659622	0	0.0659622
ASTL	0.0318256	0	0.0318256
ASTN1	0.0113398	0	0.0113398
ASTN2	0.0381933	0	0.0381933
ASXL1	0.8576248	0	0.8576248
ASXL2	0.0915688	0	0.0915688
ASXL3	0.0045801	0	0.0045801
ASZ1	0.0361441	0	0
ATAD1	0.1306684	0.1306684	0
ATAD2	0.1460019	0	0
ATAD2B	0.0831311	0	0.0831311
ATAD3A	0.0023507	0.0023507	0
ATAD3B	0.0024517	0.0024517	0
ATAD3C	0.0004171	0.0004171	0
ATAD5	0.0733127	0	0
ATCAY	0.0014165	0	0.0014165
ATE1	0.0556434	0	0.0556434
ATF1	0.1261546	0	0.1261546
ATF2	0.1470187	0	0.1470187
ATF3	0.8430128	0	0.8430128
ATF4	0.0891073	0.0891073	0
ATF5	0.0358872	0	0
ATF6	1	0	1
ATF7	0.1727803	0	0.1727803
ATF7IP	0.1389336	0	0.1389336
ATF7IP2	0.0153593	0	0
ATG10	0.0281304	0	0
ATG12	0.0891921	0	0.0891921
ATG16L1	0.1231533	0.1231533	0
ATG16L2	0.0228954	0	0.0228954
ATG2A	0.0286109	0.0286109	0
ATG2B	0.0571144	0	0.0571144
ATG3	0.0736256	0	0.0736256
ATG4A	0.032241	0	0
ATG4C	0.0552655	0	0.0552655
ATG4D	0.0092881	0.0092881	0
ATG5	0.0789381	0	0.0789381
ATG7	0.0353232	0	0.0353232

ATG9A	0.0162083	0.0162083	0
ATG9B	0.0108325	0.0108325	0
ATHL1	0.0005292	0.0005292	0
ATIC	0.0296491	0.0296491	0
ATL1	0.0854361	0	0.0854361
ATL2	0.0476021	0	0
ATL3	0.3468621	0	0.3468621
ATM	0.1195856	0	0.1195856
ATMIN	0.0245461	0	0.0245461
ATN1	0.0464035	0.0464035	0
ATOH1	0.0056519	0	0
ATOH7	0.0190557	0.0190557	0
ATOH8	0.0079337	0	0.0079337
ATOX1	0.0460046	0	0
ATP10A	0.0013708	0	0.0013708
ATP10B	0.0380105	0	0
ATP10D	0.0981071	0	0.0981071
ATP11A	0.013379	0	0
ATP11B	0.1308539	0	0
ATP11C	0.0202675	0	0.0202675
ATP12A	0.0049932	0	0
ATP13A1	0.0265392	0.0265392	0
ATP13A2	0.0085506	0.0085506	0
ATP13A3	0.0803868	0	0.0803868
ATP13A4	0.0126065	0	0
ATP13A5	0.01612	0	0
ATP1A1	0.9618595	0.9618595	0
ATP1A2	0.0156742	0	0.0156742
ATP1A3	0.0004594	0	0.0004594
ATP1A4	0.0221487	0	0.0221487
ATP1B1	0.1299089	0.1299089	0
ATP1B2	0.0012561	0	0.0012561
ATP1B3	0.0626733	0	0.0626733
ATP1B4	0.0041723	0	0
ATP2A1	0.0019563	0.0019563	0
ATP2A2	0.0542693	0	0
ATP2A3	0.0045594	0	0
ATP2B1	0.0679278	0	0.0679278
ATP2B2	0.001878	0	0.001878
ATP2B3	0.0003124	0	0.0003124
ATP2B4	0.6028166	0	0.6028166
ATP2C1	0.0621324	0	0
ATP2C2	0.0045816	0.0045816	0
ATP4A	0.0025085	0	0.0025085
ATP4B	0.0011748	0	0

ATP5A1	0.0308144	0	0.0308144
ATP5B	0.1083685	0.1083685	0
ATP5C1	0.0379757	0.0379757	0
ATP5D	0.0111961	0.0111961	0
ATP5E	0.10672	0.10672	0
ATP5EP2	0.0025024	0	0
ATP5F1	0.0870451	0	0.0870451
ATP5G1	0.0371167	0.0371167	0
ATP5G2	0.0634945	0.0634945	0
ATP5G3	0.0322174	0.0322174	0
ATP5H	0.0325452	0.0325452	0
ATP5I	0.0072056	0.0072056	0
ATP5J	0.0511891	0.0511891	0
ATP5J2	0.0260599	0.0260599	0
ATP5L	0.0769562	0.0769562	0
ATP5L2	0.0125613	0	0.0125613
ATP5O	0.0612689	0.0612689	0
ATP5S	0.2479847	0.2479847	0
ATP5SL	0.0131791	0.0131791	0
ATP6AP1	0.00286	0	0
ATP6AP1L	0.0188288	0.0188288	0
ATP6AP2	0.0319186	0	0.0319186
ATP6V0A1	0.0567628	0	0
ATP6V0A2	0.0177158	0	0.0177158
ATP6V0A4	0.0045244	0	0
ATP6V0B	0.0308911	0.0308911	0
ATP6V0C	0.0058334	0.0058334	0
ATP6V0D1	0.056478	0	0.056478
ATP6V0D2	0.015892	0	0.015892
ATP6V0E1	0.0558872	0	0.0558872
ATP6V0E2	0.003323	0	0
ATP6V1A	0.2994367	0	0.2994367
ATP6V1B1	0.0014233	0.0014233	0
ATP6V1B2	0.1504187	0	0.1504187
ATP6V1C1	0.0723176	0	0.0723176
ATP6V1C2	0.0340465	0.0340465	0
ATP6V1D	0.1925182	0.1925182	0
ATP6V1E1	0.0358486	0	0.0358486
ATP6V1E2	0.0077809	0	0
ATP6V1F	0.0107001	0	0
ATP6V1G1	0.0408748	0	0
ATP6V1G3	0.1964439	0	0.1964439
ATP6V1H	0.0464498	0	0
ATP7A	0.0473081	0	0.0473081
ATP7B	0.0163726	0.0163726	0

ATP8A1	0.0655421	0	0.0655421
ATP8A2	0.001573	0	0.001573
ATP8B1	0.0111494	0.0111494	0
ATP8B2	0.025758	0	0.025758
ATP8B3	0.0043497	0	0
ATP8B4	0.0366749	0	0.0366749
ATP9A	0.0199106	0.0199106	0
ATP9B	0.0208207	0	0.0208207
ATPAF1	0.0197025	0	0
ATPAF2	0.0350922	0	0
ATPBD4	0.0727506	0.0727506	0
ATPIF1	0.0715066	0.0715066	0
ATR	0.1021849	0	0.1021849
ATRIP	0.0230699	0	0
ATRN	0.0070423	0	0.0070423
ATRNL1	0.0057376	0	0.0057376
ATRX	0.0225179	0	0.0225179
ATXN1	0.593501	0	0.593501
ATXN10	0.0472651	0	0
ATXN1L	0.0346241	0	0.0346241
ATXN2	0.0179675	0.0179675	0
ATXN2L	0.0299736	0.0299736	0
ATXN3	0.0312478	0	0.0312478
ATXN3L	0.0182274	0	0.0182274
ATXN7	0.1386921	0	0.1386921
ATXN7L1	0.0153532	0	0
ATXN7L2	0.0150314	0	0
ATXN7L3	0.0399272	0.0399272	0
ATXN8OS	0.0054516	0	0.0054516
AUH	0.0270858	0.0270858	0
AUP1	0.1642289	0.1642289	0
AURKA	0.1122097	0.1122097	0
AURKAIP1	0.0158674	0.0158674	0
AURKAPS1	0.0930719	0.0930719	0
AURKB	0.0233174	0.0233174	0
AURKC	0.003932	0	0
AUTS2	0.0102423	0	0.0102423
AVEN	0.0249885	0	0.0249885
AVIL	0.0115993	0	0.0115993
AVL9	0.0843837	0.0843837	0
AVP	0.0015331	0	0.0015331
AVPI1	0.1721631	0.1721631	0
AVPR1A	0.0134218	0	0.0134218
AVPR1B	0.0231841	0	0.0231841
AVPR2	0.0018106	0	0.0018106

AWAT1	0.0044084	0	0
AWAT2	0.0052823	0	0
AXIN1	0.003096	0.003096	0
AXIN2	0.0134284	0	0.0134284
AXL	0.009967	0	0.009967
AZGP1	0.0045639	0.0045639	0
AZI1	0.0032814	0.0032814	0
AZI2	0.3371861	0	0.3371861
AZIN1	0.2088107	0	0
AZU1	0.0005405	0	0.0005405
B2M	0.404519	0	0.404519
B3GALNT1	0.0261754	0	0.0261754
B3GALNT2	0.1779113	0	0
B3GALT1	0.0036176	0	0.0036176
B3GALT2	0.093382	0	0.093382
B3GALT5	0.0016532	0.0016532	0
B3GALT6	0.001953	0	0
B3GALTL	0.01113	0	0.01113
B3GAT1	0.0007433	0	0.0007433
B3GAT2	0.0039951	0	0
B3GAT3	0.3700198	0.3700198	0
B3GNT1	0.0499778	0	0
B3GNT2	0.0471766	0	0.0471766
B3GNT3	0.0267111	0.0267111	0
B3GNT4	0.0151134	0.0151134	0
B3GNT5	0.0881421	0.0881421	0
B3GNT6	0.017163	0	0
B3GNT7	0.005711	0.005711	0
B3GNT8	0.0014118	0.0014118	0
B3GNT9	0.0111822	0	0.0111822
B3GNTL1	0.0132584	0.0132584	0
B4GALNT1	0.0041905	0	0.0041905
B4GALNT2	0.0040972	0	0
B4GALNT3	0.0066523	0.0066523	0
B4GALNT4	0.0004315	0	0
B4GALT1	1	0	1
B4GALT2	0.0293912	0.0293912	0
B4GALT3	0.0788187	0.0788187	0
B4GALT4	0.0330747	0.0330747	0
B4GALT5	0.1449815	0	0
B4GALT6	0.2029105	0	0.2029105
B4GALT7	0.017619	0.017619	0
B9D1	0.0118724	0.0118724	0
B9D2	0.1031586	0.1031586	0
BAALC	0.011977	0	0.011977

BAAT	0.0142166	0	0.0142166
BACE1	0.0039864	0	0.0039864
BACE2	0.0992253	0.0992253	0
BACH1	0.2631408	0	0.2631408
BACH2	0.009306	0	0.009306
BAD	0.0108776	0.0108776	0
BAG1	0.0710992	0.0710992	0
BAG2	0.1136957	0	0.1136957
BAG3	0.2600651	0	0
BAG4	0.0751344	0	0.0751344
BAG5	0.0200147	0.0200147	0
BAGE	0.3092946	0.3092946	0
BAGE2	0.3095539	0	0
BAHCC1	0.0093254	0	0
BAHD1	0.0048081	0	0
BAI1	0.0008296	0	0
BAI2	0.0031272	0	0.0031272
BAI3	0.0063513	0	0.0063513
BAIAP2	0.0027191	0.0027191	0
BAIAP2L1	0.0202158	0.0202158	0
BAIAP2L2	0.0018867	0.0018867	0
BAIAP3	0.0007632	0	0
BAK1	0.0312238	0.0312238	0
BAMBI	0.0139793	0.0139793	0
BANF1	0.0508589	0.0508589	0
BANF2	0.0044104	0	0.0044104
BANK1	0.0233766	0	0.0233766
BANP	0.043731	0.043731	0
BAP1	0.0110783	0	0.0110783
BARD1	0.0475872	0	0
BARHL1	0.0016983	0	0
BARHL2	0.0035199	0	0
BARX1	0.0104512	0	0
BARX2	0.0042278	0.0042278	0
BASE	0.0035378	0.0035378	0
BASP1	0.2959463	0	0.2959463
BAT2L1	0.0192372	0	0.0192372
BAT2L2	0.271723	0	0.271723
BATF	0.1227767	0	0
BATF2	0.0114682	0	0
BATF3	0.135558	0	0.135558
BAX	0.032023	0.032023	0
BAZ1A	0.1455101	0	0
BAZ1B	0.0398848	0	0
BAZ2A	0.273085	0.273085	0

BAZ2B	0.0884296	0	0
BBC3	0.0706808	0.0706808	0
BBOX1	0.057339	0	0
BBS1	0.0147312	0	0
BBS10	0.0598091	0	0.0598091
BBS12	0.0286383	0	0.0286383
BBS2	0.0315358	0	0
BBS4	0.0660298	0	0
BBS5	0.0140963	0	0.0140963
BBS7	0.0269687	0	0.0269687
BBS9	0.0264982	0	0.0264982
BBX	0.0447779	0	0.0447779
BCAM	0.0019585	0.0019585	0
BCAN	0.0230541	0.0230541	0
BCAP29	0.0730177	0	0.0730177
BCAP31	0.0041915	0.0041915	0
BCAR1	0.0068064	0.0068064	0
BCAR3	0.1633736	0.1633736	0
BCAR4	0.0421845	0.0421845	0
BCAS1	0.0569811	0.0569811	0
BCAS2	0.0755991	0	0.0755991
BCAS3	0.0452292	0	0
BCAS4	0.0341366	0	0.0341366
BCAT1	0.0923378	0	0.0923378
BCAT2	0.1097693	0.1097693	0
BCCIP	0.0532995	0.0532995	0
BCDIN3D	0.0708055	0	0
BCHE	0.018677	0	0.018677
BCKDHA	0.036243	0.036243	0
BCKDHB	0.0248004	0	0
BCKDK	0.006338	0	0
BCL10	0.1941053	0.1941053	0
BCL11A	0.0275705	0	0.0275705
BCL11B	0.0082124	0	0.0082124
BCL2	0.1724122	0	0.1724122
BCL2A1	0.404432	0	0.404432
BCL2L1	0.1840714	0.1840714	0
BCL2L10	0.0808971	0.0808971	0
BCL2L11	0.0470425	0	0.0470425
BCL2L12	0.0179946	0.0179946	0
BCL2L13	0.0517236	0	0
BCL2L14	0.0088663	0.0088663	0
BCL2L15	0.0268635	0.0268635	0
BCL2L2	0.0625017	0.0625017	0
BCL3	0.7596873	0.7596873	0

BCL6	1	0	1
BCL6B	0.0136765	0	0.0136765
BCL7A	0.0058421	0	0
BCL7B	0.0278528	0.0278528	0
BCL7C	0.0154605	0.0154605	0
BCL8	1.83E-05	0	1.83E-05
BCL9	0.0788236	0.0788236	0
BCL9L	0.051377	0.051377	0
BCLAF1	0.2168219	0	0.2168219
BCMO1	0.0088136	0.0088136	0
BCO2	0.0299403	0	0
BCOR	0.0161104	0	0
BCORL1	0.0016554	0	0
BCORL2	1.53E-05	0	0
BCR	0.0345728	0.0345728	0
BCS1L	0.0171415	0.0171415	0
BCYRN1	1.53E-05	0	0
BDH1	0.0293991	0.0293991	0
BDH2	0.1010292	0	0
BDKRB1	0.2248572	0	0.2248572
BDKRB2	0.0068747	0	0.0068747
BDNF	0.0993531	0	0.0993531
BDNFOS	0.2066015	0	0
BEAN	0.005495	0	0
BECN1	0.0736834	0.0736834	0
BEGAIN	0.0008586	0.0008586	0
BEND2	0.0014823	0	0
BEND3	0.0111513	0	0
BEND4	0.0035851	0	0.0035851
BEND5	0.0045777	0	0.0045777
BEND6	0.1506909	0	0.1506909
BEND7	0.0041772	0.0041772	0
BEST1	0.0214192	0	0.0214192
BEST2	0.0017269	0.0017269	0
BEST3	0.2974498	0	0.2974498
BEST4	0.0329635	0.0329635	0
BET1	0.0976558	0.0976558	0
BET1L	0.0068402	0.0068402	0
BET3L	0.0186613	0	0.0186613
BEX1	0.0017728	0	0.0017728
BEX2	0.0050096	0	0
BEX4	0.0042774	0	0.0042774
BEX5	0.0023407	0	0
BEYLA	0.036643	0	0
BFAR	0.0555942	0.0555942	0

BFSP1	0.0517559	0.0517559	0
BFSP2	0.0066396	0	0.0066396
BGLAP	0.0039987	0.0039987	0
BGN	0.0002076	0	0.0002076
BHLHA15	0.0033127	0	0
BHLHB9	0.003479	0	0.003479
BHLHE22	0.0177575	0	0.0177575
BHLHE23	0.007877	0	0
BHLHE40	1	1	0
BHLHE41	0.0445667	0	0.0445667
BHMT	0.0089056	0	0
BHMT2	0.0072554	0	0.0072554
BICC1	0.0222223	0	0.0222223
BICD1	0.0742487	0	0.0742487
BICD2	0.0147458	0	0.0147458
BID	0.0203629	0.0203629	0
BIK	0.002231	0.002231	0
BIN1	0.0060766	0	0
BIN2	0.0412059	0	0.0412059
BIN3	0.1009542	0.1009542	0
BIRC2	0.4434539	0	0.4434539
BIRC3	1	0	1
BIRC5	0.0034034	0.0034034	0
BIRC6	0.046964	0	0.046964
BIRC7	0.0094015	0	0
BIRC8	0.0005891	0	0
BIVM	0.035656	0	0
BLCAP	0.1768569	0.1768569	0
BLID	0.0168255	0	0
BLK	0.121509	0	0.121509
BLM	0.0608077	0	0.0608077
BLMH	0.0295799	0	0.0295799
BLNK	0.1415599	0	0
BLOC1S1	0.0640965	0.0640965	0
BLOC1S2	0.032108	0	0.032108
BLOC1S3	0.0371437	0	0
BLVRA	0.0698776	0	0.0698776
BLVRB	0.0183094	0.0183094	0
BLZF1	0.2611036	0.2611036	0
BMF	0.2650412	0	0.2650412
BMI1	0.0517529	0	0.0517529
BMP1	0.0079063	0	0.0079063
BMP10	0.0177914	0	0
BMP15	0.0118323	0	0
BMP2	0.0225275	0.0225275	0

BMP2K	0.1154268	0	0.1154268
BMP3	0.0064891	0	0.0064891
BMP4	0.0075787	0.0075787	0
BMP5	0.2074541	0	0.2074541
BMP6	0.0611494	0	0.0611494
BMP7	0.0022866	0	0
BMP8A	0.0119924	0	0.0119924
BMP8B	0.0031093	0	0.0031093
BMPER	0.0126703	0	0.0126703
BMPR1A	0.0667245	0	0.0667245
BMPR1B	0.0627043	0	0.0627043
BMPR2	0.0375457	0	0.0375457
BMS1	0.0259339	0.0259339	0
BMS1P4	0.0223747	0	0
BMS1P5	1.54E-05	1.54E-05	0
BMX	0.0329578	0	0.0329578
BNC1	0.0588628	0	0.0588628
BNC2	0.0070608	0	0.0070608
BNIP1	0.070603	0.070603	0
BNIP2	0.1442499	0	0.1442499
BNIP3	0.0083715	0	0
BNIP3L	0.0723822	0	0.0723822
BNIPL	0.0074662	0.0074662	0
BOC	0.0038051	0	0.0038051
BOD1	0.1801693	0	0
BOD1L	0.0416145	0	0.0416145
BOK	0.0017617	0.0017617	0
BOLA1	0.0344206	0.0344206	0
BOLA3	0.0154631	0.0154631	0
BOLL	0.0074057	0	0
BOP1	0.008452	0.008452	0
BPESC1	0.0038273	0	0
BPGM	0.3187746	0	0.3187746
BPHL	0.0326208	0.0326208	0
BPI	0.0054754	0	0.0054754
BPIL1	0.0026031	0	0
BPIL2	0.0043033	0	0.0043033
BPIL3	0.0032235	0.0032235	0
BPNT1	0.2013377	0.2013377	0
BPTF	0.0432037	0	0.0432037
BRAF	0.0449381	0	0.0449381
BRAP	0.0281443	0	0
BRCA1	0.0560205	0	0
BRCA2	0.0927965	0	0.0927965
BRCC3	0.0445048	0.0445048	0

BRD1	0.0059107	0.0059107	0
BRD3	0.0051124	0	0
BRD4	0.0603993	0	0
BRD7	0.0290607	0.0290607	0
BRD7P3	0.0087588	0	0.0087588
BRD8	1	1	0
BRD9	0.0137358	0.0137358	0
BRDT	0.0049882	0	0.0049882
BRE	0.1042172	0	0
BREA2	0.0023322	0	0
BRF1	0.0043124	0.0043124	0
BRF2	0.0188296	0	0
BRI3	0.0105647	0.0105647	0
BRI3BP	0.0576513	0.0576513	0
BRIP1	0.0955435	0	0
BRIX1	0.3007668	0.3007668	0
BRMS1	0.0746951	0.0746951	0
BRMS1L	0.0582565	0	0.0582565
BRP44	0.5047789	0.5047789	0
BRP44L	0.0272209	0	0.0272209
BRPF1	0.0396287	0	0.0396287
BRPF3	0.0191594	0	0.0191594
BRS3	0.0106888	0	0.0106888
BRSK1	0.0090257	0	0
BRSK2	0.0005162	0	0
BRWD1	0.0329363	0	0
BRWD3	0.0559619	0	0.0559619
BSCL2	0.0409857	0.0409857	0
BSDC1	0.046915	0	0
BSG	0.0155146	0.0155146	0
BSN	0.005468	0	0.005468
BSND	0.0030262	0	0
BSPH1	0.0020317	0	0
BSPRY	0.016376	0.016376	0
BST1	0.0183337	0	0.0183337
BST2	0.080201	0	0.080201
BSX	0.0031569	0	0
BTAF1	0.0856754	0	0
BTBD1	0.0996158	0	0.0996158
BTBD10	0.0365992	0.0365992	0
BTBD11	0.0078781	0	0.0078781
BTBD12	0.026953	0	0
BTBD16	0.0097877	0.0097877	0
BTBD17	0.0011256	0	0
BTBD18	0.0183178	0	0

BTBD19	0.154311	0	0.154311
BTBD2	0.0031843	0.0031843	0
BTBD3	0.0548967	0.0548967	0
BTBD6	0.0015271	0	0
BTBD7	0.0736136	0	0.0736136
BTBD8	0.0189808	0	0.0189808
BTBD9	0.0394244	0	0.0394244
BTC	0.0095307	0.0095307	0
BTD	0.0872856	0	0
BTF3	0.0824425	0.0824425	0
BTF3L1	0.1089652	0	0
BTF3L4	0.1392089	0	0.1392089
BTG1	1	0	1
BTG2	0.2643844	0	0.2643844
BTG3	0.0539649	0	0.0539649
BTG4	0.0100752	0	0
BTK	0.0224353	0	0.0224353
BTLA	0.1148962	0	0.1148962
BTN1A1	0.0111223	0	0.0111223
BTN2A1	0.0237249	0	0.0237249
BTN2A2	0.0267566	0	0.0267566
BTN2A3	0.0429288	0	0.0429288
BTN3A1	0.0647622	0	0.0647622
BTN3A2	0.0742714	0	0.0742714
BTN3A3	0.0775135	0	0.0775135
BTNL3	0.0118649	0.0118649	0
BTNL9	0.0054995	0	0
BTRC	0.0556811	0	0
BUB1	0.0510419	0.0510419	0
BUB1B	0.068538	0.068538	0
BUB3	0.1004191	0.1004191	0
BUD13	0.0568549	0	0
BUD31	0.0408974	0.0408974	0
BVES	0.0075672	0	0.0075672
BYSL	0.0697595	0	0
BZRAP1	0.0979163	0	0
BZW1	0.1015668	0	0
BZW2	0.1181498	0.1181498	0
C10orf10	0.4867866	0	0.4867866
C10orf105	0.0381223	0	0.0381223
C10orf107	0.0312817	0	0.0312817
C10orf108	0.0011292	0.0011292	0
C10orf11	0.0073477	0	0.0073477
C10orf110	0.0039875	0	0
C10orf111	0.0102502	0.0102502	0

C10orf113	0.0047156	0.0047156	0
C10orf114	0.0136513	0	0.0136513
C10orf116	0.029724	0	0
C10orf118	0.0913225	0	0
C10orf119	0.0479591	0	0.0479591
C10orf12	0.0178773	0	0
C10orf120	0.0097436	0	0.0097436
C10orf122	0.0220765	0	0
C10orf125	0.0012181	0	0.0012181
C10orf128	0.0447308	0	0.0447308
C10orf129	0.007985	0	0
C10orf131	0.0283078	0	0.0283078
C10orf137	0.0367597	0	0.0367597
C10orf140	0.0769857	0	0.0769857
C10orf18	0.0674112	0	0.0674112
C10orf2	0.0070864	0	0
C10orf25	0.0297564	0	0.0297564
C10orf26	0.5856836	0	0.5856836
C10orf27	0.0068664	0	0
C10orf28	0.1076825	0	0
C10orf32	0.0283218	0	0.0283218
C10orf35	0.0051246	0.0051246	0
C10orf4	0.0898013	0	0
C10orf40	0.0214245	0	0
C10orf41	0.0074098	0	0
C10orf46	0.0450967	0	0
C10orf47	0.0144992	0.0144992	0
C10orf50	0.0045452	0	0.0045452
C10orf53	0.0087045	0	0
C10orf54	0.0377365	0	0.0377365
C10orf55	0.038067	0	0.038067
C10orf57	0.0184422	0.0184422	0
C10orf58	0.0182088	0.0182088	0
C10orf62	0.0066207	0.0066207	0
C10orf67	0.0679255	0	0
C10orf68	0.0017225	0.0017225	0
C10orf71	0.0036555	0	0
C10orf72	0.0053132	0	0.0053132
C10orf75	0.2285893	0.2285893	0
C10orf76	0.0510728	0	0
C10orf78	1	1	0
C10orf79	0.0239329	0.0239329	0
C10orf81	0.0234078	0	0
C10orf82	0.0036336	0	0.0036336
C10orf84	0.0510237	0	0.0510237

C10orf88	0.0357125	0	0
C10orf90	0.0337201	0	0.0337201
C10orf91	0.0038567	0.0038567	0
C10orf93	0.0011247	0	0
C10orf95	0.0274165	0.0274165	0
C10orf96	0.0088942	0	0.0088942
C10orf99	0.0636198	0.0636198	0
C11orf1	0.0761715	0.0761715	0
C11orf10	0.0746567	0.0746567	0
C11orf16	0.0342595	0	0
C11orf17	0.3558222	0	0
C11orf2	0.0440395	0.0440395	0
C11orf20	0.0135825	0.0135825	0
C11orf21	0.00027	0	0.00027
C11orf24	0.0784905	0	0.0784905
C11orf30	0.1262562	0	0.1262562
C11orf31	0.0731518	0.0731518	0
C11orf34	0.0126876	0	0
C11orf35	0.0011261	0.0011261	0
C11orf36	0.0017195	0	0
C11orf40	0.0149457	0	0
C11orf41	0.0186061	0	0.0186061
C11orf42	0.02859	0.02859	0
C11orf45	0.0140436	0	0
C11orf46	0.0985319	0	0.0985319
C11orf48	0.1096492	0.1096492	0
C11orf49	0.0774155	0.0774155	0
C11orf51	0.1051596	0.1051596	0
C11orf52	0.0109289	0.0109289	0
C11orf53	0.0083001	0.0083001	0
C11orf54	0.5739209	0	0
C11orf57	0.0589018	0	0
C11orf58	0.0808433	0	0.0808433
C11orf59	0.0897535	0	0
C11orf61	0.0309797	0.0309797	0
C11orf63	0.0207404	0	0.0207404
C11orf64	0.018601	0.018601	0
C11orf65	0.0273185	0	0
C11orf66	0.0036527	0	0
C11orf67	0.0605446	0.0605446	0
C11orf68	0.0291127	0	0.0291127
C11orf70	0.0100059	0.0100059	0
C11orf71	0.122047	0.122047	0
C11orf73	0.1472608	0	0
C11orf74	0.0556321	0	0

C11orf75	0.1181896	0.1181896	0
C11orf80	0.1827007	0.1827007	0
C11orf82	0.1655432	0	0.1655432
C11orf83	0.115295	0.115295	0
C11orf84	0.0243046	0	0
C11orf85	0.0163976	0	0
C11orf86	0.1958698	0.1958698	0
C11orf87	0.0047455	0	0.0047455
C11orf88	0.0096733	0	0.0096733
C11orf9	0.0053978	0.0053978	0
C11orf90	0.0184199	0	0
C11orf92	0.0045987	0.0045987	0
C11orf93	0.0044388	0.0044388	0
C11orf94	0.0018226	0	0
C11orf95	0.00456	0	0.00456
C12orf10	0.0493798	0.0493798	0
C12orf11	0.1408886	0	0
C12orf12	0.0170738	0	0.0170738
C12orf23	0.0393852	0	0.0393852
C12orf24	0.0503706	0	0.0503706
C12orf26	0.0781983	0	0.0781983
C12orf27	0.0076563	0.0076563	0
C12orf29	0.0685953	0	0
C12orf32	0.023234	0.023234	0
C12orf34	0.0054457	0	0
C12orf35	0.0726366	0	0.0726366
C12orf36	0.0089457	0	0
C12orf39	0.024216	0	0.024216
C12orf4	0.185278	0	0.185278
C12orf40	0.0673474	0	0
C12orf41	0.0740347	0	0.0740347
C12orf42	0.1054876	0	0.1054876
C12orf43	0.0170964	0	0
C12orf44	0.0584841	0.0584841	0
C12orf45	0.0432726	0	0
C12orf47	0.0124043	0.0124043	0
C12orf48	0.0855432	0	0
C12orf49	0.0300937	0	0.0300937
C12orf5	0.031209	0	0.031209
C12orf50	0.0418551	0	0
C12orf51	0.0155434	0	0
C12orf52	0.0952063	0.0952063	0
C12orf53	0.0019928	0	0.0019928
C12orf54	0.0065262	0	0.0065262
C12orf56	0.0091388	0	0

C12orf57	0.3490475	0	0
C12orf59	0.5373215	0	0.5373215
C12orf60	0.1351254	0	0
C12orf61	0.128983	0.128983	0
C12orf62	0.1025118	0.1025118	0
C12orf63	0.0034238	0	0.0034238
C12orf65	0.085678	0	0
C12orf66	0.0458661	0	0
C12orf68	0.0180284	0	0.0180284
C12orf69	0.0230087	0	0.0230087
C12orf70	0.0111695	0.0111695	0
C12orf71	0.3851167	0	0
C12orf72	0.3388338	0	0
C12orf73	0.2084017	0.2084017	0
C12orf74	0.0243133	0	0.0243133
C12orf75	0.0556377	0.0556377	0
C12orf76	0.0063389	0.0063389	0
C12orf77	0.1408989	0	0.1408989
C13orf1	0.0249372	0.0249372	0
C13orf15	0.013011	0	0.013011
C13orf16	0.0012408	0.0012408	0
C13orf18	0.0798787	0	0.0798787
C13orf23	0.136993	0.136993	0
C13orf26	0.0073557	0	0.0073557
C13orf27	0.022839	0	0.022839
C13orf28	0.0028621	0	0
C13orf29	0.2687723	0	0
C13orf30	0.0053466	0	0.0053466
C13orf31	0.0258659	0	0.0258659
C13orf33	0.0043138	0	0.0043138
C13orf34	0.0668317	0.0668317	0
C13orf35	0.0033597	0	0
C13orf36	0.0028907	0	0.0028907
C13orf37	0.0632969	0.0632969	0
C13orf38	0.0167425	0	0
C13orf39	0.0113768	0	0.0113768
C14orf1	0.0907196	0.0907196	0
C14orf101	0.0801395	0.0801395	0
C14orf102	0.0223874	0	0.0223874
C14orf104	0.0605414	0.0605414	0
C14orf105	0.0423173	0	0
C14orf106	0.1317155	0	0.1317155
C14orf109	0.0204983	0	0
C14orf115	0.0025896	0	0
C14orf118	0.0353479	0	0.0353479

C14orf119	0.1020807	0.1020807	0
C14orf126	0.0829927	0	0
C14orf128	0.1094261	0.1094261	0
C14orf129	0.0237094	0.0237094	0
C14orf132	0.0038387	0	0.0038387
C14orf135	0.1389723	0	0.1389723
C14orf138	0.0637947	0.0637947	0
C14orf139	0.0032165	0	0.0032165
C14orf142	0.0562686	0.0562686	0
C14orf143	0.0651601	0.0651601	0
C14orf145	0.1233962	0	0.1233962
C14orf147	0.0595522	0	0
C14orf148	0.0073181	0.0073181	0
C14orf149	0.1978052	0	0.1978052
C14orf153	0.0186278	0.0186278	0
C14orf156	0.0509512	0.0509512	0
C14orf159	0.0806578	0	0
C14orf162	0.0372861	0	0.0372861
C14orf165	0.0065321	0.0065321	0
C14orf166	0.1003054	0.1003054	0
C14orf166B	0.0051272	0	0
C14orf167	0.0045479	0.0045479	0
C14orf169	0.0220822	0	0
C14orf174	0.0486078	0	0
C14orf176	0.0845541	0.0845541	0
C14orf177	0.0104547	0	0.0104547
C14orf178	0.0285074	0	0
C14orf179	0.5246791	0.5246791	0
C14orf180	0.0003057	0	0.0003057
C14orf181	0.5330451	0.5330451	0
C14orf182	0.1326436	0	0
C14orf183	0.0420872	0	0
C14orf184	0.0038895	0.0038895	0
C14orf19	0.0091187	0	0
C14orf2	0.0203265	0.0203265	0
C14orf21	0.0440305	0	0
C14orf23	0.0064977	0	0
C14orf28	0.0283987	0	0
C14orf33	0.0935575	0	0.0935575
C14orf34	0.6165994	0.6165994	0
C14orf37	0.0086812	0	0.0086812
C14orf39	0.016176	0	0
C14orf4	0.3173347	0	0
C14orf43	0.361403	0	0
C14orf45	0.0156145	0	0.0156145

C14orf48	0.0106911	0	0
C14orf49	0.0088432	0	0.0088432
C14orf50	0.0219077	0.0219077	0
C14orf53	0.0375082	0.0375082	0
C14orf64	0.0129972	0	0.0129972
C14orf68	0.001435	0.001435	0
C14orf70	0.0069897	0	0
C14orf72	0.0760767	0.0760767	0
C14orf73	0.0044248	0.0044248	0
C14orf79	0.0193878	0.0193878	0
C14orf80	0.0112525	0.0112525	0
C14orf86	0.0014239	0	0
C14orf93	0.0500932	0.0500932	0
C15orf17	0.0349349	0	0
C15orf2	0.0022231	0	0.0022231
C15orf21	0.0070162	0	0
C15orf23	0.0383579	0.0383579	0
C15orf24	0.0432272	0	0
C15orf26	0.0086403	0	0.0086403
C15orf27	0.0472546	0	0.0472546
C15orf28	0.0269926	0	0
C15orf29	0.0406821	0	0
C15orf32	0.0075526	0	0
C15orf33	0.4096041	0	0.4096041
C15orf34	0.0525893	0	0.0525893
C15orf37	0.0397285	0.0397285	0
C15orf38	0.0865203	0	0.0865203
C15orf39	0.0566467	0	0
C15orf40	0.0574868	0.0574868	0
C15orf41	0.05135	0.05135	0
C15orf42	0.0840755	0.0840755	0
C15orf43	0.0057383	0.0057383	0
C15orf44	0.0582708	0	0
C15orf48	0.0270621	0.0270621	0
C15orf5	0.020754	0	0
C15orf50	0.0060897	0	0.0060897
C15orf51	0.0005313	0	0.0005313
C15orf52	0.0082064	0.0082064	0
C15orf53	0.0530184	0	0.0530184
C15orf54	0.0206098	0	0.0206098
C15orf55	0.0214984	0.0214984	0
C15orf56	0.0051385	0.0051385	0
C15orf57	0.1830321	0	0.1830321
C15orf58	0.0415016	0.0415016	0
C15orf59	0.0021286	0	0.0021286

C15orf60	0.0036488	0	0
C15orf61	0.1037731	0.1037731	0
C15orf62	0.0742519	0.0742519	0
C15orf63	0.1133408	0.1133408	0
C16orf11	0.0003693	0	0
C16orf13	0.0121707	0.0121707	0
C16orf3	0.0118162	0.0118162	0
C16orf42	0.002225	0.002225	0
C16orf45	0.4112074	0	0.4112074
C16orf46	0.0301914	0	0
C16orf48	0.0079773	0.0079773	0
C16orf5	0.0094238	0	0.0094238
C16orf52	0.0206373	0	0.0206373
C16orf53	0.469233	0.469233	0
C16orf55	0.00426	0.00426	0
C16orf57	0.0252892	0	0
C16orf58	0.0214902	0.0214902	0
C16orf59	0.0038465	0.0038465	0
C16orf61	0.1814037	0.1814037	0
C16orf62	0.0523069	0	0.0523069
C16orf63	0.0531371	0	0
C16orf68	0.0121206	0.0121206	0
C16orf7	0.1931725	0.1931725	0
C16orf70	0.0261637	0	0.0261637
C16orf71	0.0114259	0	0
C16orf72	0.0760873	0	0.0760873
C16orf73	0.0087862	0.0087862	0
C16orf74	0.0450599	0	0.0450599
C16orf75	0.0222623	0.0222623	0
C16orf78	0.0029155	0	0
C16orf79	0.0060055	0.0060055	0
C16orf80	0.0543241	0	0
C16orf81	0.0016836	0	0
C16orf82	0.0011745	0	0.0011745
C16orf86	0.0080417	0	0
C16orf87	0.081076	0	0.081076
C16orf88	0.0148668	0.0148668	0
C16orf89	0.0084446	0	0.0084446
C16orf90	0.0044161	0.0044161	0
C16orf91	0.0018873	0.0018873	0
C16orf92	0.0023297	0.0023297	0
C16orf93	0.0143171	0.0143171	0
C17orf100	0.0529005	0.0529005	0
C17orf101	0.0091982	0.0091982	0
C17orf102	0.0008224	0	0.0008224

C17orf103	0.0108133	0	0.0108133
C17orf104	0.0025146	0	0
C17orf105	0.0132608	0	0
C17orf106	0.0866066	0.0866066	0
C17orf107	0.0068727	0	0.0068727
C17orf108	0.0266266	0	0.0266266
C17orf28	0.0012718	0.0012718	0
C17orf37	0.0237246	0.0237246	0
C17orf39	0.0350644	0	0.0350644
C17orf42	0.0276125	0.0276125	0
C17orf44	0.2278508	0	0.2278508
C17orf46	0.004935	0	0
C17orf47	0.0067515	0	0
C17orf48	0.0581194	0	0.0581194
C17orf49	0.0582021	0	0
C17orf50	0.0028905	0.0028905	0
C17orf51	0.0166697	0	0.0166697
C17orf53	0.5089834	0.5089834	0
C17orf54	0.0024145	0	0
C17orf55	0.0027833	0.0027833	0
C17orf56	0.0028319	0.0028319	0
C17orf57	0.1567721	0	0
C17orf58	0.0148798	0.0148798	0
C17orf59	0.2524527	0.2524527	0
C17orf60	0.0005832	0	0.0005832
C17orf61	0.3475316	0.3475316	0
C17orf62	0.6371227	0	0.6371227
C17orf63	0.0084702	0.0084702	0
C17orf64	0.0032195	0	0.0032195
C17orf65	0.0261272	0.0261272	0
C17orf66	0.0069663	0	0.0069663
C17orf67	0.0260223	0	0
C17orf68	0.0266881	0	0
C17orf69	0.009471	0.009471	0
C17orf70	0.0094695	0	0
C17orf71	0.0455099	0	0.0455099
C17orf72	0.0019856	0	0.0019856
C17orf73	0.0106678	0.0106678	0
C17orf74	0.0041253	0	0
C17orf75	0.060247	0.060247	0
C17orf76	0.0028606	0.0028606	0
C17orf77	0.0059779	0.0059779	0
C17orf78	0.010846	0	0
C17orf79	0.0102207	0	0
C17orf80	0.0765736	0	0.0765736

C17orf81	0.0629169	0	0.0629169
C17orf82	0.0021162	0.0021162	0
C17orf85	0.0102321	0	0.0102321
C17orf86	0.0421973	0.0421973	0
C17orf87	0.1103812	0	0.1103812
C17orf88	0.004923	0	0.004923
C17orf89	0.0029049	0.0029049	0
C17orf90	0.0064116	0.0064116	0
C17orf91	0.1581719	0	0.1581719
C17orf93	0.0032562	0.0032562	0
C17orf95	0.1261375	0.1261375	0
C17orf96	0.0210033	0	0
C17orf97	0.0113846	0.0113846	0
C17orf98	0.0044076	0	0
C17orf99	0.0396934	0	0.0396934
C18orf1	0.0649258	0	0.0649258
C18orf10	0.0475608	0	0.0475608
C18orf16	0.0069766	0	0.0069766
C18orf18	0.0225154	0.0225154	0
C18orf19	0.0444083	0	0
C18orf2	0.0068025	0	0
C18orf20	0.0050878	0	0
C18orf21	0.0769696	0	0
C18orf22	0.0162218	0	0
C18orf25	0.028933	0	0.028933
C18orf26	0.0088496	0	0
C18orf32	0.1399811	0	0.1399811
C18orf34	0.0047939	0	0.0047939
C18orf45	0.026665	0.026665	0
C18orf54	0.0305644	0	0.0305644
C18orf55	0.0484798	0	0.0484798
C18orf56	0.027617	0.027617	0
C18orf62	0.0044265	0	0
C18orf8	0.0351881	0.0351881	0
C19orf10	0.0055353	0.0055353	0
C19orf12	0.0151896	0	0.0151896
C19orf18	0.0021273	0.0021273	0
C19orf2	0.018162	0	0
C19orf20	0.2922462	0.2922462	0
C19orf21	0.0042231	0.0042231	0
C19orf22	0.0215812	0.0215812	0
C19orf23	0.1799002	0.1799002	0
C19orf24	0.0455683	0.0455683	0
C19orf25	0.0034408	0.0034408	0
C19orf26	0.0018619	0	0.0018619

C19orf28	0.0355597	0.0355597	0
C19orf29	0.0050202	0.0050202	0
C19orf30	0.0052449	0	0.0052449
C19orf33	0.0091658	0.0091658	0
C19orf34	0.0004314	0	0
C19orf35	0.0039752	0	0.0039752
C19orf36	0.0842182	0.0842182	0
C19orf38	0.0029646	0	0.0029646
C19orf39	0.0498494	0.0498494	0
C19orf40	0.0262749	0	0.0262749
C19orf41	0.0050429	0	0
C19orf42	0.0169541	0.0169541	0
C19orf43	0.059122	0.059122	0
C19orf44	0.0175992	0.0175992	0
C19orf45	0.0079937	0.0079937	0
C19orf46	0.0053317	0.0053317	0
C19orf47	0.0179421	0.0179421	0
C19orf48	0.0631143	0.0631143	0
C19orf50	0.0145225	0.0145225	0
C19orf51	0.0302423	0.0302423	0
C19orf52	0.0100714	0.0100714	0
C19orf53	0.0571908	0.0571908	0
C19orf54	0.09097	0.09097	0
C19orf55	0.0165634	0.0165634	0
C19orf56	0.0136882	0.0136882	0
C19orf57	0.0059838	0.0059838	0
C19orf59	0.0090248	0	0.0090248
C19orf6	0.0923506	0.0923506	0
C19orf60	0.0125479	0.0125479	0
C19orf61	0.8072422	0	0
C19orf62	0.0252579	0.0252579	0
C19orf63	0.0041245	0.0041245	0
C19orf66	0.0053552	0	0
C19orf69	0.0055052	0	0
C19orf70	0.0091862	0.0091862	0
C19orf71	0.0030508	0.0030508	0
C19orf73	0.0141352	0.0141352	0
C19orf75	0.0085822	0	0.0085822
C19orf76	0.0019867	0	0.0019867
C19orf77	0.0017593	0.0017593	0
C1D	0.0437604	0	0
C1GALT1	0.5000891	0.5000891	0
C1GALT1C1	0.0664406	0.0664406	0
C1orf100	0.1162612	0	0
C1orf101	0.0749821	0	0

C1orf103	0.1325752	0	0
C1orf104	0.0281139	0.0281139	0
C1orf105	0.067432	0.067432	0
C1orf106	0.2379417	0.2379417	0
C1orf107	0.2396065	0	0
C1orf109	0.0471412	0	0.0471412
C1orf110	0.0288939	0	0
C1orf111	0.7051635	0.7051635	0
C1orf112	0.1210822	0.1210822	0
C1orf113	0.0346149	0.0346149	0
C1orf114	0.0340128	0	0
C1orf115	0.0194362	0.0194362	0
C1orf116	0.393538	0.393538	0
C1orf122	0.0173715	0.0173715	0
C1orf123	0.0275911	0	0.0275911
C1orf124	0.0978897	0	0.0978897
C1orf125	0.0380765	0.0380765	0
C1orf126	0.0746397	0.0746397	0
C1orf127	0.0101618	0	0.0101618
C1orf128	0.032507	0	0
C1orf129	0.0238754	0	0.0238754
C1orf130	0.0042334	0	0
C1orf131	0.083805	0	0
C1orf133	0.0853935	0.0853935	0
C1orf135	0.0229353	0.0229353	0
C1orf14	0.0388936	0	0.0388936
C1orf141	0.009461	0	0
C1orf144	0.0779612	0	0.0779612
C1orf146	0.0055038	0	0
C1orf150	0.0211555	0	0.0211555
C1orf151	0.0657506	0	0
C1orf152	4.58E-05	0	0
C1orf156	0.1252361	0	0
C1orf157	0.0075599	0	0
C1orf158	0.0170421	0	0
C1orf159	0.0020026	0.0020026	0
C1orf161	0.3412452	0	0
C1orf162	0.0382505	0	0.0382505
C1orf163	0.0484223	0	0.0484223
C1orf168	0.0090468	0	0.0090468
C1orf170	0.0008397	0.0008397	0
C1orf172	0.0064962	0.0064962	0
C1orf173	0.0070564	0	0.0070564
C1orf174	0.0111163	0	0
C1orf175	0.0023906	0.0023906	0

C1orf177	0.0163067	0.0163067	0
C1orf180	0.0495419	0	0
C1orf182	0.106033	0.106033	0
C1orf183	0.0643722	0	0.0643722
C1orf185	0.0082504	0	0
C1orf186	0.1653791	0	0.1653791
C1orf187	0.0028418	0	0.0028418
C1orf189	0.0426062	0	0
C1orf190	0.00695	0	0.00695
C1orf192	0.0371693	0.0371693	0
C1orf194	0.0231407	0	0
C1orf198	0.0587695	0	0.0587695
C1orf200	0.0326254	0	0.0326254
C1orf201	0.0825746	0.0825746	0
C1orf203	0.0279475	0	0
C1orf204	0.0415035	0.0415035	0
C1orf21	0.0436077	0	0.0436077
C1orf210	0.004965	0.004965	0
C1orf212	0.0556037	0	0.0556037
C1orf213	0.0556635	0.0556635	0
C1orf216	0.0134457	0	0.0134457
C1orf220	0.0605592	0.0605592	0
C1orf223	0.0153664	0.0153664	0
C1orf226	1	1	0
C1orf227	0.0126975	0	0.0126975
C1orf228	0.2439115	0	0.2439115
C1orf229	0.0124791	0.0124791	0
C1orf230	0.010304	0	0
C1orf25	0.1922224	0	0
C1orf26	0.1911136	0	0
C1orf27	0.464107	0.464107	0
C1orf31	0.4191424	0.4191424	0
C1orf35	0.0153023	0.0153023	0
C1orf38	0.1086634	0	0.1086634
C1orf43	0.1454427	0.1454427	0
C1orf49	0.0055792	0.0055792	0
C1orf50	0.0220693	0.0220693	0
C1orf51	0.0221395	0.0221395	0
C1orf52	0.0398538	0	0
C1orf53	0.0561583	0.0561583	0
C1orf54	0.0418131	0	0.0418131
C1orf55	0.2174452	0	0.2174452
C1orf56	0.0502026	0.0502026	0
C1orf57	0.1768025	0.1768025	0
C1orf58	0.1438217	0	0

C1orf59	0.0315436	0	0
C1orf61	0.0088979	0	0
C1orf63	0.1023198	0.1023198	0
C1orf64	0.008587	0	0
C1orf65	0.0051381	0	0
C1orf66	0.0906765	0.0906765	0
C1orf68	0.087294	0	0
C1orf69	0.0437644	0	0
C1orf70	0.0003677	0	0.0003677
C1orf74	0.189637	0	0
C1orf77	0.1035953	0.1035953	0
C1orf83	0.1559033	0	0.1559033
C1orf84	0.0487599	0	0.0487599
C1orf85	0.0145932	0	0.0145932
C1orf86	0.0128738	0.0128738	0
C1orf87	0.0081166	0.0081166	0
C1orf88	0.0089377	0	0
C1orf89	0.0214574	0	0
C1orf9	0.2351258	0.2351258	0
C1orf91	0.0395735	0.0395735	0
C1orf92	0.0097606	0	0.0097606
C1orf93	0.0054819	0.0054819	0
C1orf94	0.0022512	0	0.0022512
C1orf95	0.030057	0	0.030057
C1orf96	0.0441505	0	0.0441505
C1orf97	0.2304402	0.2304402	0
C1QA	0.0018918	0	0.0018918
C1QB	0.0020038	0	0.0020038
C1QBP	0.0583999	0	0
C1QC	0.0017506	0	0.0017506
C1QL1	0.0116455	0	0
C1QL2	0.0013438	0	0
C1QL3	0.0075209	0	0
C1QL4	0.0040127	0.0040127	0
C1QTNF1	0.3265268	0	0.3265268
C1QTNF2	0.0114107	0	0.0114107
C1QTNF3	0.0370963	0	0.0370963
C1QTNF4	0.0037344	0	0
C1QTNF6	0.7742627	0	0.7742627
C1QTNF7	0.0225767	0	0.0225767
C1QTNF8	0.0002992	0.0002992	0
C1QTNF9	0.0017833	0	0.0017833
C1QTNF9B	0.0129008	0	0.0129008
C1R	0.0347891	0	0.0347891
C1RL	0.8762958	0	0.8762958

C1S	1	0	1
C20orf103	0.0032108	0	0.0032108
C20orf106	0.0037754	0	0
C20orf107	0.0023821	0	0
C20orf108	0.0117859	0.0117859	0
C20orf11	0.0332903	0.0332903	0
C20orf111	0.3937557	0.3937557	0
C20orf112	0.0086114	0.0086114	0
C20orf114	0.0031803	0	0
C20orf117	0.0227679	0	0.0227679
C20orf118	0.0099005	0.0099005	0
C20orf12	0.0220916	0.0220916	0
C20orf123	0.0369339	0	0.0369339
C20orf132	0.075969	0	0.075969
C20orf134	0.014759	0.014759	0
C20orf135	0.0139571	0.0139571	0
C20orf141	0.230537	0	0.230537
C20orf144	0.0153305	0.0153305	0
C20orf151	0.0004921	0.0004921	0
C20orf152	0.0088139	0	0
C20orf160	0.0044851	0	0.0044851
C20orf165	0.0269943	0.0269943	0
C20orf166	0.0002089	0	0
C20orf173	0.0027688	0	0
C20orf177	0.079712	0	0.079712
C20orf185	0.0023286	0	0.0023286
C20orf186	0.003087	0	0
C20orf191	0.0018903	0	0
C20orf194	0.0105505	0	0.0105505
C20orf195	0.0055463	0.0055463	0
C20orf196	0.0236206	0	0
C20orf197	0.051781	0	0.051781
C20orf199	0.7663207	0.7663207	0
C20orf20	0.0171771	0.0171771	0
C20orf200	0.0002226	0	0.0002226
C20orf201	0.0016169	0.0016169	0
C20orf202	0.0446612	0	0
C20orf203	0.0083839	0	0
C20orf24	0.0609515	0.0609515	0
C20orf26	0.0261277	0	0
C20orf27	0.0038631	0	0
C20orf29	0.1352329	0.1352329	0
C20orf3	0.0454172	0	0.0454172
C20orf30	0.0638995	0	0
C20orf4	0.0457214	0	0

C20orf43	0.0393398	0.0393398	0
C20orf46	0.0020122	0.0020122	0
C20orf54	0.0173893	0.0173893	0
C20orf56	0.0008619	0.0008619	0
C20orf7	0.0673972	0.0673972	0
C20orf70	0.0031392	0.0031392	0
C20orf71	0.0211954	0.0211954	0
C20orf72	0.1612377	0	0
C20orf79	0.0026346	0	0
C20orf85	0.0034454	0	0
C20orf94	0.1825519	0	0.1825519
C20orf96	0.0574583	0.0574583	0
C21orf119	0.0209471	0.0209471	0
C21orf121	0.0047493	0	0
C21orf122	0.0170942	0	0.0170942
C21orf125	0.0372317	0.0372317	0
C21orf128	0.0060393	0	0.0060393
C21orf129	0.0090176	0.0090176	0
C21orf130	0.0025804	0	0
C21orf131	0.0060572	0	0
C21orf15	0.0003428	0	0
C21orf2	0.0059337	0.0059337	0
C21orf29	0.0013561	0	0.0013561
C21orf33	0.0173287	0	0
C21orf34	0.1348807	0	0.1348807
C21orf45	0.0189396	0.0189396	0
C21orf49	0.2194912	0	0
C21orf54	0.004422	0	0
C21orf56	0.0055992	0.0055992	0
C21orf57	0.0179285	0.0179285	0
C21orf58	0.014858	0.014858	0
C21orf59	0.0245524	0.0245524	0
C21orf62	0.0110649	0	0.0110649
C21orf63	0.0455665	0	0.0455665
C21orf67	0.0111615	0	0
C21orf7	0.092024	0	0.092024
C21orf70	0.0118574	0.0118574	0
C21orf71	0.1218157	0	0
C21orf81	0.0010117	0	0
C21orf82	0.005831	0	0.005831
C21orf84	0.3636231	0	0
C21orf88	0.0026667	0	0
C21orf90	0.0017463	0	0
C21orf91	0.0930469	0	0.0930469
C21orf94	0.0031979	0	0.0031979

C21orf96	0.0622275	0	0.0622275
C21orf99	7.89E-05	7.89E-05	0
C22orf13	0.0285804	0	0
C22orf15	0.0053906	0	0.0053906
C22orf23	0.0052476	0	0
C22orf24	0.0530571	0	0
C22orf25	0.0028271	0	0.0028271
C22orf26	0.0171416	0	0.0171416
C22orf27	0.0096468	0.0096468	0
C22orf28	0.0450148	0	0
C22orf29	0.0206559	0.0206559	0
C22orf30	0.0429497	0	0
C22orf31	0.0042983	0	0.0042983
C22orf32	0.0181774	0.0181774	0
C22orf33	0.0037141	0	0
C22orf34	0.0009525	0	0.0009525
C22orf36	0.0017942	0.0017942	0
C22orf39	0.0136192	0	0.0136192
C22orf40	0.0055967	0	0
C22orf41	0.0045834	0.0045834	0
C22orf42	0.0012238	0	0.0012238
C22orf43	0.0022547	0	0
C22orf45	0.0163397	0	0.0163397
C22orf46	0.0128406	0	0.0128406
C22orf9	0.0561778	0	0.0561778
C2CD2	0.0617805	0	0.0617805
C2CD2L	0.0688079	0.0688079	0
C2CD3	0.1580457	0	0
C2CD4A	0.2481186	0.2481186	0
C2CD4B	0.0110929	0.0110929	0
C2CD4C	0.0002748	0.0002748	0
C2CD4D	0.0575408	0.0575408	0
C2orf14	1.77E-05	1.77E-05	0
C2orf15	0.0191635	0.0191635	0
C2orf16	0.0213762	0.0213762	0
C2orf18	1	0	0
C2orf24	0.0137481	0.0137481	0
C2orf27A	0.0043246	0	0
C2orf27B	0.0002301	0	0
C2orf28	0.0556992	0.0556992	0
C2orf29	0.0413612	0.0413612	0
C2orf3	0.0528001	0	0
C2orf34	0.0925978	0.0925978	0
C2orf39	0.0085041	0	0
C2orf40	0.001823	0	0.001823

C2orf42	0.066261	0	0.066261
C2orf43	0.0725202	0	0
C2orf44	0.0270438	0	0.0270438
C2orf47	0.0299919	0.0299919	0
C2orf48	0.0083967	0.0083967	0
C2orf49	0.057046	0	0
C2orf50	0.0214982	0.0214982	0
C2orf51	0.0028554	0	0
C2orf52	0.0155382	0	0
C2orf53	0.0033091	0	0
C2orf54	0.0008483	0.0008483	0
C2orf55	0.0032123	0.0032123	0
C2orf56	0.0718342	0	0
C2orf57	0.0034212	0	0
C2orf58	0.0047124	0	0.0047124
C2orf60	0.0291745	0.0291745	0
C2orf61	0.0273563	0.0273563	0
C2orf62	0.0041233	0	0
C2orf63	0.3850607	0.3850607	0
C2orf64	0.0482088	0.0482088	0
C2orf65	0.0086512	0	0.0086512
C2orf66	0.0127481	0	0
C2orf67	0.242947	0	0
C2orf68	0.0378368	0.0378368	0
C2orf69	0.0282224	0	0.0282224
C2orf7	0.0233841	0	0
C2orf70	0.0017959	0.0017959	0
C2orf71	0.0374322	0	0.0374322
C2orf72	0.0022984	0.0022984	0
C2orf73	0.0440283	0	0
C2orf74	0.02003	0	0
C2orf76	0.0250912	0	0.0250912
C2orf77	0.0162925	0	0
C2orf78	0.0038449	0	0
C2orf79	0.0285942	0.0285942	0
C2orf80	0.0077159	0	0.0077159
C2orf81	0.004811	0.004811	0
C2orf82	0.0034846	0.0034846	0
C2orf83	0.0074155	0	0.0074155
C2orf84	0.0068667	0	0.0068667
C2orf85	0.0033377	0	0.0033377
C2orf86	0.2005647	0	0.2005647
C2orf88	0.0210336	0	0
C2orf89	0.0720154	0	0
C3	0.2001777	0	0.2001777

C3AR1	0.037024	0	0.037024
C3orf1	0.0800493	0	0
C3orf10	0.0183768	0	0
C3orf14	0.0072296	0	0
C3orf15	0.038259	0.038259	0
C3orf16	0.01219	0	0
C3orf17	0.0352911	0	0.0352911
C3orf18	0.0139646	0	0.0139646
C3orf19	0.0888429	0.0888429	0
C3orf20	0.0098643	0	0.0098643
C3orf21	0.0502965	0	0.0502965
C3orf22	0.0027885	0	0
C3orf23	0.0531043	0.0531043	0
C3orf24	0.0083815	0.0083815	0
C3orf26	0.0752418	0	0
C3orf27	0.0034987	0	0
C3orf30	0.0100898	0	0
C3orf31	0.0257937	0	0
C3orf32	0.0114329	0	0.0114329
C3orf33	0.0136715	0.0136715	0
C3orf34	0.0872323	0	0.0872323
C3orf35	0.0101451	0.0101451	0
C3orf36	0.0080018	0	0.0080018
C3orf37	0.0507731	0.0507731	0
C3orf38	0.0713309	0	0.0713309
C3orf39	0.0085531	0	0.0085531
C3orf42	0.0134895	0	0.0134895
C3orf43	0.0149782	0.0149782	0
C3orf45	0.0062952	0	0
C3orf47	0.0542514	0	0
C3orf48	0.0021788	0.0021788	0
C3orf49	0.0104802	0	0
C3orf50	0.0326849	0	0.0326849
C3orf51	0.0306678	0	0
C3orf52	0.0653435	0.0653435	0
C3orf54	0.0058919	0	0.0058919
C3orf55	0.0112462	0	0
C3orf57	0.0147324	0.0147324	0
C3orf58	0.1311409	0	0.1311409
C3orf59	0.0589083	0	0.0589083
C3orf62	0.0152161	0	0.0152161
C3orf63	0.0788618	0	0.0788618
C3orf64	0.0310693	0	0.0310693
C3orf65	0.0166076	0.0166076	0
C3orf66	0.0033066	0	0.0033066

C3orf67	0.0193793	0	0
C3orf70	0.0143761	0	0.0143761
C3orf71	0.0334395	0.0334395	0
C3orf72	0.0041848	0	0.0041848
C3orf74	0.0208132	0.0208132	0
C3orf75	0.0200836	0	0
C3orf77	0.0172754	0	0
C3orf79	0.0106818	0	0
C3P1	0.0023265	0	0
C4BPA	0.1151653	0	0
C4BPB	0.471637	0.471637	0
C4orf10	0.0208681	0.0208681	0
C4orf11	0.0024196	0	0
C4orf12	0.0235769	0	0.0235769
C4orf14	0.0473913	0.0473913	0
C4orf17	0.0059347	0	0
C4orf19	0.0111694	0.0111694	0
C4orf21	0.0643611	0.0643611	0
C4orf22	0.0087899	0	0.0087899
C4orf23	0.0264038	0.0264038	0
C4orf26	0.0101987	0	0
C4orf27	0.1199708	0	0.1199708
C4orf29	0.0247882	0.0247882	0
C4orf3	0.0556783	0	0.0556783
C4orf31	0.0065833	0	0.0065833
C4orf32	0.027344	0	0.027344
C4orf33	0.1325029	0.1325029	0
C4orf34	0.0627552	0	0
C4orf35	0.0030177	0.0030177	0
C4orf36	0.0393777	0	0
C4orf37	0.0347754	0.0347754	0
C4orf38	0.0189364	0	0
C4orf39	0.0255373	0	0.0255373
C4orf40	0.0064173	0	0
C4orf41	0.0207257	0	0.0207257
C4orf42	0.0050439	0	0.0050439
C4orf43	0.0497166	0	0
C4orf44	0.0016654	0.0016654	0
C4orf45	0.0083077	0	0
C4orf46	0.0410624	0	0.0410624
C4orf47	0.025137	0.025137	0
C4orf48	0.0012547	0.0012547	0
C4orf49	0.003948	0	0.003948
C4orf50	0.0014937	0	0.0014937
C4orf51	0.0115333	0	0

C4orf52	0.0187659	0.0187659	0
C4orf6	0.0011376	0	0
C4orf7	0.0191517	0	0.0191517
C5	0.0326363	0	0
C5AR1	0.0484777	0	0.0484777
C5orf13	0.3203309	0	0.3203309
C5orf15	0.0836129	0	0.0836129
C5orf20	0.0180182	0	0.0180182
C5orf22	0.0952333	0	0
C5orf23	0.0456206	0	0.0456206
C5orf24	0.0719209	0	0.0719209
C5orf25	0.0002043	0.0002043	0
C5orf27	0.9359563	0	0
C5orf28	0.1708333	0.1708333	0
C5orf30	0.0529084	0.0529084	0
C5orf32	0.0909989	0.0909989	0
C5orf33	0.1747475	0	0.1747475
C5orf34	0.0857869	0	0
C5orf35	0.0727302	0	0
C5orf36	0.1155125	0	0.1155125
C5orf38	0.0263502	0	0
C5orf39	0.2618721	0	0.2618721
C5orf4	0.0451385	0	0
C5orf40	0.010648	0	0.010648
C5orf41	0.3525979	0	0.3525979
C5orf42	0.0403412	0	0.0403412
C5orf43	0.0601339	0.0601339	0
C5orf44	0.0931811	0	0
C5orf45	0.0186728	0.0186728	0
C5orf46	0.1321272	0	0.1321272
C5orf47	0.0092668	0	0
C5orf48	0.0156781	0.0156781	0
C5orf49	0.0108124	0	0
C5orf51	0.1096124	0	0.1096124
C5orf52	0.008527	0.008527	0
C5orf53	0.0670002	0	0.0670002
C5orf54	0.0582331	0.0582331	0
C5orf55	0.0220887	0.0220887	0
C5orf56	0.2177949	0	0.2177949
C5orf58	0.0101173	0	0.0101173
C5orf60	0.0005943	0.0005943	0
C5orf62	0.1100866	0	0.1100866
C6	0.0296476	0	0.0296476
C6orf1	0.0147881	0	0
C6orf103	0.007785	0	0

C6orf105	0.1884145	0	0.1884145
C6orf106	0.0518893	0	0
C6orf108	0.0144512	0.0144512	0
C6orf114	0.1163765	0	0.1163765
C6orf115	0.0454352	0	0.0454352
C6orf118	0.0004631	0.0004631	0
C6orf120	0.029272	0	0.029272
C6orf122	0.0049797	0	0.0049797
C6orf125	0.0277929	0.0277929	0
C6orf126	0.0071656	0.0071656	0
C6orf127	0.0081565	0	0
C6orf129	0.0694188	0	0
C6orf130	0.1973398	0.1973398	0
C6orf132	0.0627729	0.0627729	0
C6orf138	0.0119378	0	0.0119378
C6orf141	0.0905329	0.0905329	0
C6orf142	0.0209912	0	0.0209912
C6orf145	0.0209029	0	0.0209029
C6orf146	0.0048719	0	0
C6orf147	0.0065942	0.0065942	0
C6orf150	0.4983519	0	0.4983519
C6orf153	0.0116223	0	0
C6orf154	0.0161715	0.0161715	0
C6orf155	0.0439506	0	0
C6orf162	0.0074237	0	0.0074237
C6orf163	0.0129426	0	0
C6orf164	0.0159526	0	0
C6orf165	0.0224117	0	0.0224117
C6orf167	0.0368907	0	0.0368907
C6orf168	0.013516	0	0
C6orf170	0.0325873	0	0.0325873
C6orf174	0.0052266	0	0.0052266
C6orf176	0.0163868	0	0.0163868
C6orf182	0.1218582	0	0.1218582
C6orf186	0.0053935	0	0.0053935
C6orf191	0.0234527	0	0.0234527
C6orf192	0.021622	0	0.021622
C6orf195	0.2532485	0	0
C6orf201	0.0047355	0	0
C6orf203	0.0081121	0	0
C6orf204	0.0139644	0	0.0139644
C6orf208	0.0182063	0	0.0182063
C6orf211	0.0517834	0	0.0517834
C6orf217	0.1263612	0	0
C6orf218	0.4533604	0.4533604	0

C6orf221	0.0016423	0	0
C6orf222	0.0126819	0	0
C6orf223	0.0505696	0.0505696	0
C6orf225	0.0587907	0	0.0587907
C6orf226	0.0295113	0.0295113	0
C6orf227	0.0137897	0.0137897	0
C6orf35	0.0792424	0	0.0792424
C6orf41	0.0010445	0.0010445	0
C6orf52	0.0617185	0.0617185	0
C6orf57	0.0288399	0	0
C6orf58	0.0156202	0	0
C6orf59	0.003672	0	0.003672
C6orf62	0.1307325	0	0.1307325
C6orf64	0.0358823	0	0
C6orf70	0.0259949	0	0
C6orf72	0.0910444	0	0.0910444
C6orf81	0.2196603	0	0.2196603
C6orf89	0.0619613	0	0.0619613
C6orf94	0.0095633	0	0.0095633
C6orf97	0.0100077	0	0.0100077
C7	0.0211181	0	0.0211181
C7orf10	0.0239259	0	0.0239259
C7orf11	0.024696	0.024696	0
C7orf13	0.0244815	0.0244815	0
C7orf16	0.0149198	0	0.0149198
C7orf23	0.1033455	0	0
C7orf25	0.2419852	0	0
C7orf26	0.0089482	0	0
C7orf27	0.0120997	0.0120997	0
C7orf28A	0.0006853	0	0
C7orf28B	0.0006838	0	0
C7orf29	0.0495961	0	0
C7orf30	0.1524604	0.1524604	0
C7orf31	0.0132504	0	0.0132504
C7orf33	0.0042208	0	0.0042208
C7orf34	0.0017966	0	0.0017966
C7orf36	0.0416332	0.0416332	0
C7orf4	0.0086004	0.0086004	0
C7orf40	0.942125	0.942125	0
C7orf41	0.0147146	0	0
C7orf42	0.0390492	0.0390492	0
C7orf43	0.0149567	0.0149567	0
C7orf44	0.1386696	0.1386696	0
C7orf45	0.2513794	0	0
C7orf46	0.011789	0.011789	0

C7orf47	0.124386	0.124386	0
C7orf49	0.3585334	0	0
C7orf50	0.0072168	0.0072168	0
C7orf51	0.0058909	0	0
C7orf52	0.0063281	0	0.0063281
C7orf53	0.1157254	0.1157254	0
C7orf54	0.0125179	0.0125179	0
C7orf55	0.1616973	0.1616973	0
C7orf57	0.0162475	0.0162475	0
C7orf58	0.0625044	0	0.0625044
C7orf59	0.0321834	0.0321834	0
C7orf60	0.0465514	0	0.0465514
C7orf61	0.0047033	0	0
C7orf63	0.0524478	0	0
C7orf64	0.0654163	0	0
C7orf65	0.0201855	0	0
C7orf66	0.0061074	0	0.0061074
C7orf68	0.0695999	0	0
C7orf69	0.0265161	0	0.0265161
C7orf70	0.0097789	0	0.0097789
C7orf71	0.0043454	0	0
C7orf72	0.0124569	0	0.0124569
C8A	0.0057651	0.0057651	0
C8B	0.0064005	0	0
C8G	0.0100911	0.0100911	0
C8orf12	0.0122764	0	0
C8orf22	0.0265529	0	0
C8orf31	0.0048231	0	0
C8orf33	0.0133952	0.0133952	0
C8orf34	0.0065011	0	0.0065011
C8orf37	0.1577337	0	0.1577337
C8orf38	0.0978376	0.0978376	0
C8orf39	0.0676348	0	0
C8orf4	0.2367777	0	0
C8orf40	0.1787661	0.1787661	0
C8orf41	0.140376	0	0
C8orf42	0.0045308	0	0.0045308
C8orf44	0.0763774	0.0763774	0
C8orf45	0.0113708	0.0113708	0
C8orf46	0.0138417	0	0.0138417
C8orf47	0.0123822	0	0
C8orf48	0.0073471	0	0.0073471
C8orf51	0.0062397	0.0062397	0
C8orf55	0.0323101	0.0323101	0
C8orf56	0.0115454	0	0

C8orf58	0.0170944	0	0.0170944
C8orf59	0.0541442	0.0541442	0
C8orf71	0.0061727	0	0
C8orf73	0.017974	0.017974	0
C8orf74	0.0515415	0	0
C8orf75	0.201488	0.201488	0
C8orf76	0.0735819	0.0735819	0
C8orf77	0.0347507	0.0347507	0
C8orf79	0.0410179	0	0
C8orf80	0.0856542	0	0.0856542
C8orf83	0.0435346	0.0435346	0
C8orf84	0.014094	0	0.014094
C8orf85	0.0699293	0	0.0699293
C8orf86	0.0182859	0	0
C8ORFK29	0.0026857	0.0026857	0
C9	0.0422175	0	0
C9orf100	0.1126351	0.1126351	0
C9orf102	0.0907616	0	0.0907616
C9orf103	0.027545	0.027545	0
C9orf106	0.0044307	0.0044307	0
C9orf109	0.0114568	0	0.0114568
C9orf11	0.0125245	0	0
C9orf110	0.0021397	0	0.0021397
C9orf114	0.0061311	0.0061311	0
C9orf116	0.0100936	0.0100936	0
C9orf117	0.012498	0.012498	0
C9orf119	0.0235448	0	0
C9orf122	0.0113313	0.0113313	0
C9orf123	0.05595	0	0
C9orf125	0.0311244	0.0311244	0
C9orf128	0.0161353	0.0161353	0
C9orf129	0.001528	0	0
C9orf130	0.0920214	0	0
C9orf131	0.0150921	0	0
C9orf135	0.0061609	0	0
C9orf139	0.0044848	0	0.0044848
C9orf140	0.0101978	0.0101978	0
C9orf142	0.0107358	0.0107358	0
C9orf144	0.0040258	0	0.0040258
C9orf144B	0.005207	0	0.005207
C9orf150	0.1759383	0.1759383	0
C9orf152	0.0112268	0.0112268	0
C9orf153	0.0097062	0	0
C9orf156	0.1162497	0	0.1162497
C9orf16	0.0161636	0.0161636	0

C9orf163	0.0205821	0.0205821	0
C9orf167	0.0159943	0.0159943	0
C9orf170	0.0097132	0	0.0097132
C9orf171	0.0249843	0	0
C9orf172	0.0075875	0.0075875	0
C9orf173	0.0041112	0.0041112	0
C9orf21	0.0203552	0	0.0203552
C9orf23	0.0197198	0.0197198	0
C9orf24	0.011319	0.011319	0
C9orf25	0.0291013	0	0.0291013
C9orf27	0.1463104	0	0
C9orf3	0.0331093	0.0331093	0
C9orf30	0.5375788	0	0.5375788
C9orf37	0.0326173	0.0326173	0
C9orf4	0.0055863	0	0.0055863
C9orf40	0.0369808	0	0
C9orf41	0.0357797	0	0.0357797
C9orf43	0.0823601	0.0823601	0
C9orf44	0.0041888	0	0.0041888
C9orf45	0.017714	0	0
C9orf46	0.4224523	0	0.4224523
C9orf47	0.009466	0	0.009466
C9orf5	0.1150083	0	0
C9orf50	0.026352	0	0
C9orf53	0.0037333	0	0
C9orf57	0.0080698	0	0
C9orf6	0.048855	0.048855	0
C9orf64	0.0752002	0	0
C9orf66	0.0122226	0	0
C9orf68	0.5194944	0.5194944	0
C9orf69	0.0118814	0.0118814	0
C9orf7	0.0050275	0.0050275	0
C9orf70	0.0151611	0	0
C9orf71	0.0115905	0	0
C9orf72	0.1374121	0	0.1374121
C9orf78	0.0281981	0	0.0281981
C9orf79	0.017094	0	0.017094
C9orf80	0.0469101	0	0.0469101
C9orf82	0.0696797	0	0
C9orf84	0.0581198	0	0.0581198
C9orf85	0.0981233	0	0
C9orf86	0.0111325	0.0111325	0
C9orf89	0.0314063	0.0314063	0
C9orf9	0.0078562	0	0
C9orf91	0.0938749	0	0.0938749

C9orf93	0.0185409	0	0.0185409
C9orf95	0.0649982	0.0649982	0
C9orf96	0.0208358	0.0208358	0
C9orf98	0.0075932	0.0075932	0
CA1	0.012459	0	0.012459
CA10	0.0144427	0	0.0144427
CA11	0.0283994	0	0
CA12	0.1815671	0.1815671	0
CA13	0.1550062	0.1550062	0
CA14	0.0384485	0.0384485	0
CA2	0.0124159	0	0
CA3	0.0060201	0	0
CA4	0.0036078	0	0
CA5A	0.001748	0	0.001748
CA5B	0.0162435	0	0.0162435
CA5BP	0.0618286	0	0
CA6	0.0052861	0	0
CA7	0.0021086	0	0
CA8	0.0037711	0	0
CA9	0.0102794	0.0102794	0
CAB39	0.0499564	0	0.0499564
CAB39L	0.0985971	0	0.0985971
CABC1	0.0867251	0	0
CABIN1	0.0407173	0	0
CABLES1	0.068041	0.068041	0
CABLES2	0.0050941	0.0050941	0
CABP1	0.0120808	0	0
CABP2	0.0045855	0	0
CABP4	0.0052435	0.0052435	0
CABP5	0.0016694	0	0
CABP7	0.0010829	0	0.0010829
CABYR	0.0155512	0	0.0155512
CACHD1	0.0268756	0	0.0268756
CACNA1A	0.0031859	0	0.0031859
CACNA1B	0.0028806	0	0
CACNA1C	0.0048865	0	0
CACNA1D	0.0077111	0	0
CACNA1E	0.0935939	0	0.0935939
CACNA1F	0.0024548	0.0024548	0
CACNA1G	0.0016059	0	0.0016059
CACNA1H	0.0002259	0	0.0002259
CACNA1I	0.0024421	0	0.0024421
CACNA1S	0.0148668	0	0.0148668
CACNA2D1	0.021949	0	0.021949
CACNA2D2	0.000665	0	0.000665

CACNA2D3	0.0043529	0	0.0043529
CACNA2D4	0.0014701	0	0.0014701
CACNB1	0.0242642	0.0242642	0
CACNB2	0.0924045	0	0.0924045
CACNB3	0.0068551	0.0068551	0
CACNB4	0.0108871	0	0.0108871
CACNG1	0.0059563	0	0
CACNG2	0.003099	0	0
CACNG3	0.0138992	0	0.0138992
CACNG4	0.0011263	0	0
CACNG5	0.0033917	0	0.0033917
CACNG6	0.0008878	0.0008878	0
CACNG7	0.0004934	0	0
CACNG8	0.0006752	0.0006752	0
CACYBP	0.492408	0.492408	0
CAD	0.0515568	0	0.0515568
CADM1	0.0037114	0	0.0037114
CADM2	0.0091861	0	0.0091861
CADM3	0.0419883	0	0.0419883
CADM4	0.0021516	0.0021516	0
CADPS	0.0034379	0	0
CADPS2	0.0272341	0	0.0272341
CAGE1	0.0625674	0	0
CALB1	0.0076029	0	0.0076029
CALB2	0.0130742	0	0
CALCA	0.0065757	0	0.0065757
CALCB	0.0051888	0	0
CALCOCO1	0.0586289	0	0.0586289
CALCOCO2	0.0497848	0	0
CALCR	0.0441433	0	0.0441433
CALCRL	0.0479729	0	0.0479729
CALD1	0.1541513	0	0.1541513
CALHM1	0.010398	0	0.010398
CALHM2	0.0094601	0	0.0094601
CALHM3	0.0242168	0.0242168	0
CALM1	0.0433116	0	0.0433116
CALM2	0.1826446	0.1826446	0
CALM3	0.0137301	0	0
CALML3	0.0024005	0	0
CALML4	0.0035212	0.0035212	0
CALML5	0.0018855	0	0
CALML6	0.0016048	0.0016048	0
CALN1	0.0030747	0	0
CALR	0.0816186	0.0816186	0
CALR3	0.016778	0.016778	0

CALU	0.0955487	0	0.0955487
CALY	0.0015304	0	0.0015304
CAMK1	0.0080474	0	0.0080474
CAMK1D	0.0233482	0	0.0233482
CAMK1G	0.0450291	0	0.0450291
CAMK2A	0.0096908	0	0.0096908
CAMK2B	0.0018012	0	0
CAMK2D	0.2251232	0	0.2251232
CAMK2G	0.0176766	0.0176766	0
CAMK2N1	0.0032065	0.0032065	0
CAMK2N2	0.0062582	0	0
CAMK4	0.0446967	0	0.0446967
CAMKK1	0.0136619	0	0.0136619
CAMKK2	0.0168441	0	0.0168441
CAMKV	0.0014733	0	0
CAMLG	0.0543196	0	0
CAMP	0.02088	0	0.02088
CAMSAP1	0.0415962	0	0
CAMSAP1L1	0.2133218	0	0.2133218
CAMTA1	0.0516686	0.0516686	0
CAMTA2	0.012608	0	0.012608
CAND1	0.1146966	0	0.1146966
CAND2	0.0032181	0	0.0032181
CANT1	0.2046489	0.2046489	0
CANX	0.0550887	0	0
CAP1	0.2377712	0	0.2377712
CAP2	0.0379604	0	0.0379604
CAPG	0.0303361	0	0
CAPN1	0.0155643	0.0155643	0
CAPN10	0.0152142	0.0152142	0
CAPN11	0.0036763	0	0.0036763
CAPN12	0.0009127	0.0009127	0
CAPN13	0.00289	0.00289	0
CAPN14	0.0139571	0	0.0139571
CAPN2	0.3720682	0.3720682	0
CAPN3	0.0306905	0	0.0306905
CAPN5	0.0050343	0.0050343	0
CAPN6	0.005726	0	0.005726
CAPN7	0.0551908	0	0.0551908
CAPN8	0.0650168	0.0650168	0
CAPN9	0.0122144	0.0122144	0
CAPNS1	0.1746429	0.1746429	0
CAPNS2	0.1506414	0	0.1506414
CAPRIN1	0.0926106	0	0
CAPRIN2	0.0409599	0.0409599	0

CAPS	0.0016704	0.0016704	0
CAPS2	0.0513229	0	0.0513229
CAPSL	0.025041	0	0.025041
CAPZA1	0.1763481	0	0.1763481
CAPZA2	0.1484424	0	0.1484424
CAPZA3	0.0098194	0	0
CAPZB	0.0124553	0	0.0124553
CARD10	0.0186637	0.0186637	0
CARD11	0.0035703	0.0035703	0
CARD14	0.047423	0.047423	0
CARD16	0.1144612	0	0.1144612
CARD17	0.0523411	0	0.0523411
CARD18	0.0379026	0	0
CARD6	0.3537404	0	0.3537404
CARD8	0.070098	0	0.070098
CARD9	0.0015642	0	0.0015642
CARHSP1	0.2576438	0.2576438	0
CARKD	0.0345113	0.0345113	0
CARM1	0.0589197	0.0589197	0
CARNS1	0.0030141	0	0
CARS	0.1343512	0.1343512	0
CARS2	0.1570484	0.1570484	0
CASC1	0.0586907	0	0
CASC2	0.0708283	0.0708283	0
CASC3	0.0781278	0	0
CASC4	0.2231708	0	0.2231708
CASC5	0.2315906	0	0
CASD1	0.0700117	0	0.0700117
CASK	0.0520749	0	0
CASKIN1	0.0020392	0	0
CASKIN2	0.0096348	0	0
CASP1	0.0523411	0	0.0523411
CASP10	0.0588751	0	0
CASP12	0.0119287	0	0.0119287
CASP14	0.0065922	0	0
CASP2	0.040815	0.040815	0
CASP3	0.0275214	0	0.0275214
CASP4	0.7599548	0	0
CASP5	0.0068195	0	0.0068195
CASP6	0.0356059	0.0356059	0
CASP7	0.1540212	0.1540212	0
CASP8	0.4912137	0	0
CASP8AP2	0.0438248	0	0.0438248
CASP9	0.1223932	0	0
CASQ1	0.0121255	0	0.0121255

CASQ2	0.0229473	0	0.0229473
CASR	0.0133411	0	0
CASS4	0.0112469	0	0.0112469
CAST	0.3024447	0.3024447	0
CASZ1	0.0035456	0.0035456	0
CAT	0.0585681	0	0.0585681
CATSPER1	0.0035227	0	0
CATSPER2	0.0009211	0.0009211	0
CATSPER2P1	0.0288981	0.0288981	0
CATSPER3	0.0339178	0.0339178	0
CATSPER4	0.0040547	0.0040547	0
CATSPERB	0.0112015	0.0112015	0
CATSPERG	0.0087524	0.0087524	0
CAV1	0.168007	0	0.168007
CAV2	0.0834854	0	0
CAV3	0.0126736	0	0.0126736
CBARA1	0.0227391	0	0
CBFA2T2	0.0794473	0	0
CBFA2T3	0.0083726	0	0.0083726
CBFB	0.0332333	0	0.0332333
CBL	0.0260168	0	0.0260168
CBLB	0.1358122	0	0.1358122
CBLC	0.0566098	0.0566098	0
CBLL1	0.0680174	0	0
CBLN1	0.0014637	0	0.0014637
CBLN2	0.0043136	0	0.0043136
CBLN3	0.058972	0	0.058972
CBLN4	0.0031198	0	0.0031198
CBR1	0.0311907	0.0311907	0
CBR3	0.0410805	0	0.0410805
CBR4	0.0475512	0	0
CBS	0.0016895	0	0.0016895
CBWD1	0.0001073	0.0001073	0
CBWD2	6.44E-05	6.44E-05	0
CBWD5	0.0001062	0	0.0001062
CBWD6	1.64E-05	1.64E-05	0
CBX1	0.051278	0	0.051278
CBX2	0.0047335	0	0
CBX3	0.4219061	0.4219061	0
CBX4	0.0072322	0.0072322	0
CBX5	0.6757729	0	0.6757729
CBX6	0.0168735	0	0.0168735
CBX7	0.0170887	0	0.0170887
CBX8	0.0164413	0.0164413	0
CBY1	0.0080101	0	0

CC2D1A	0.0060944	0.0060944	0
CC2D1B	0.0850771	0	0
CC2D2A	0.0133384	0	0.0133384
CC2D2B	0.0111618	0	0.0111618
CCAR1	0.0151138	0	0.0151138
CCBE1	0.011717	0	0.011717
CCBL1	0.0920188	0.0920188	0
CCBL2	0.1556279	0	0
CCBP2	0.0262141	0	0.0262141
CCDC101	0.0181657	0	0
CCDC102A	0.0331922	0	0.0331922
CCDC102B	0.1164108	0	0.1164108
CCDC103	0.1543949	0.1543949	0
CCDC104	0.0632049	0.0632049	0
CCDC105	0.0027501	0	0
CCDC106	0.0350281	0	0
CCDC107	0.376868	0.376868	0
CCDC108	0.0022283	0.0022283	0
CCDC109A	0.1098723	0.1098723	0
CCDC109B	0.0224721	0	0.0224721
CCDC11	0.01728	0.01728	0
CCDC110	0.0087123	0	0.0087123
CCDC111	0.0264485	0	0.0264485
CCDC112	0.0235402	0.0235402	0
CCDC113	0.0275885	0.0275885	0
CCDC114	0.0260846	0.0260846	0
CCDC115	0.041386	0.041386	0
CCDC116	0.0093129	0	0
CCDC117	0.0291984	0	0.0291984
CCDC12	0.0436928	0.0436928	0
CCDC120	0.0023701	0.0023701	0
CCDC121	0.1061617	0	0.1061617
CCDC122	0.0230728	0	0
CCDC123	0.0274107	0	0
CCDC124	0.2668129	0.2668129	0
CCDC126	0.1188674	0	0.1188674
CCDC127	0.0149367	0	0
CCDC129	0.0063267	0	0
CCDC13	0.0595547	0	0.0595547
CCDC130	0.0166343	0.0166343	0
CCDC132	0.1034872	0.1034872	0
CCDC134	0.011775	0	0
CCDC135	0.005353	0	0
CCDC136	0.0046483	0	0
CCDC137	0.0066578	0.0066578	0

CCDC138	0.0230055	0	0.0230055
CCDC14	0.0365638	0.0365638	0
CCDC140	0.001143	0	0
CCDC141	0.0195012	0	0.0195012
CCDC142	0.0162366	0.0162366	0
CCDC144A	0.0002117	0	0.0002117
CCDC144B	0.0006416	0	0
CCDC144C	0.0004627	0	0
CCDC144NL	0.0007459	0	0.0007459
CCDC146	0.0094483	0	0
CCDC147	0.3272265	0	0.3272265
CCDC148	0.0298971	0.0298971	0
CCDC149	0.0183007	0	0
CCDC15	0.021533	0	0
CCDC150	0.0349026	0.0349026	0
CCDC151	0.0169346	0	0
CCDC152	0.0289663	0	0.0289663
CCDC153	0.008214	0.008214	0
CCDC154	0.0002098	0.0002098	0
CCDC155	0.0027718	0	0.0027718
CCDC157	0.0091101	0.0091101	0
CCDC158	0.0059279	0	0
CCDC159	0.0436471	0.0436471	0
CCDC160	0.0033305	0	0.0033305
CCDC163P	0.0958836	0	0
CCDC17	0.0105686	0	0
CCDC18	0.184547	0	0.184547
CCDC19	0.0277324	0.0277324	0
CCDC21	0.0346029	0	0
CCDC22	0.0036573	0.0036573	0
CCDC23	0.0464313	0	0.0464313
CCDC24	0.0123127	0.0123127	0
CCDC25	0.1090308	0	0.1090308
CCDC27	0.0012387	0	0
CCDC28A	0.0870034	0	0
CCDC28B	0.0187564	0	0.0187564
CCDC3	0.0066216	0	0.0066216
CCDC30	0.0147353	0	0
CCDC33	0.0486603	0	0
CCDC34	0.1099596	0.1099596	0
CCDC36	0.0026091	0	0.0026091
CCDC37	0.00209	0	0
CCDC38	0.0158943	0	0
CCDC39	0.0104355	0	0.0104355
CCDC40	0.0103024	0	0

CCDC41	0.0575219	0	0
CCDC42	0.0028855	0	0.0028855
CCDC42B	0.0089716	0.0089716	0
CCDC43	0.0256078	0.0256078	0
CCDC45	0.4479685	0.4479685	0
CCDC46	0.0906715	0	0.0906715
CCDC47	0.2749693	0.2749693	0
CCDC48	0.0023724	0	0.0023724
CCDC50	0.2234568	0	0.2234568
CCDC51	0.0838086	0.0838086	0
CCDC52	0.0509212	0.0509212	0
CCDC53	0.0988497	0	0
CCDC54	0.0105601	0	0.0105601
CCDC55	0.1732891	0	0.1732891
CCDC56	0.0133164	0.0133164	0
CCDC57	0.0293295	0.0293295	0
CCDC58	0.107197	0.107197	0
CCDC59	0.0773866	0	0
CCDC6	0.1567453	0	0
CCDC60	0.0038802	0	0
CCDC61	0.0201532	0.0201532	0
CCDC62	0.0129172	0	0.0129172
CCDC63	0.0039073	0	0
CCDC64	0.0130035	0.0130035	0
CCDC64B	0.0051803	0.0051803	0
CCDC65	0.0106768	0	0.0106768
CCDC66	0.0481763	0	0.0481763
CCDC67	0.0210503	0	0.0210503
CCDC68	0.0436338	0.0436338	0
CCDC69	0.0577657	0	0.0577657
CCDC7	0.03209	0	0
CCDC70	0.0157499	0	0
CCDC71	0.0095451	0	0.0095451
CCDC72	0.0891197	0.0891197	0
CCDC73	0.0174002	0.0174002	0
CCDC74A	0.0004072	0	0
CCDC74B	0.0002277	0	0
CCDC75	0.0906598	0	0.0906598
CCDC76	0.1789371	0	0
CCDC77	0.0685591	0	0
CCDC78	0.0020259	0.0020259	0
CCDC79	0.0112042	0.0112042	0
CCDC8	0.0009408	0	0.0009408
CCDC80	0.0570987	0	0.0570987
CCDC81	0.0132542	0	0.0132542

CCDC82	0.105597	0	0.105597
CCDC83	0.0298016	0	0
CCDC84	0.0226409	0.0226409	0
CCDC85A	0.0061431	0	0.0061431
CCDC85B	0.1071276	0.1071276	0
CCDC85C	0.0132959	0.0132959	0
CCDC86	0.0190518	0.0190518	0
CCDC87	0.0063057	0	0
CCDC88A	0.1684843	0	0.1684843
CCDC88B	0.0372104	0	0.0372104
CCDC88C	0.0418949	0	0
CCDC89	0.0222668	0	0.0222668
CCDC9	0.1221692	0.1221692	0
CCDC90A	0.0246897	0	0.0246897
CCDC90B	0.209366	0	0
CCDC91	0.0319342	0.0319342	0
CCDC92	0.0303834	0	0.0303834
CCDC93	0.0122568	0	0
CCDC94	0.0359972	0.0359972	0
CCDC96	0.0042935	0.0042935	0
CCDC97	0.0273329	0.0273329	0
CCDC99	0.0917667	0	0.0917667
CCIN	0.0039003	0	0.0039003
CCK	0.0424307	0.0424307	0
CCKAR	0.0030186	0	0.0030186
CCKBR	0.016037	0	0
CCL1	0.0056103	0	0.0056103
CCL11	0.0020122	0	0.0020122
CCL13	0.0046792	0	0.0046792
CCL14	0.0047294	0	0.0047294
CCL14-CCL15	0.0120257	0	0
CCL15	0.0120257	0	0
CCL16	0.0063972	0	0.0063972
CCL17	0.0057985	0	0.0057985
CCL18	0.0172292	0	0.0172292
CCL19	0.0037813	0	0.0037813
CCL2	0.0089317	0	0.0089317
CCL20	0.3182846	0	0
CCL21	0.00562	0	0.00562
CCL22	0.0355731	0	0.0355731
CCL23	0.0045371	0	0.0045371
CCL24	0.0027362	0	0
CCL25	0.0020953	0	0.0020953
CCL26	0.0027746	0	0.0027746
CCL27	0.0074169	0	0

CCL28	0.0770072	0	0
CCL3	0.0748465	0	0.0748465
CCL4	0.0090305	0	0.0090305
CCL5	0.0155468	0	0.0155468
CCL7	0.0047515	0	0.0047515
CCL8	0.0039223	0	0.0039223
CCM2	0.1768902	0	0
CCNA1	0.0193037	0	0.0193037
CCNA2	0.0649607	0.0649607	0
CCNB1	0.0266998	0.0266998	0
CCNB1IP1	0.1407882	0.1407882	0
CCNB2	0.0860872	0.0860872	0
CCNB3	0.0011443	0.0011443	0
CCNC	0.0646831	0	0
CCND1	0.0097196	0.0097196	0
CCND2	0.061908	0	0.061908
CCND3	0.1446709	0	0.1446709
CCNDBP1	0.0376382	0	0
CCNE1	0.008263	0.008263	0
CCNE2	0.0258562	0	0
CCNF	0.030201	0.030201	0
CCNG1	0.4267311	0	0
CCNG2	0.097454	0	0
CCNH	0.1098952	0	0
CCNI	0.0691482	0	0.0691482
CCNI2	0.0469707	0.0469707	0
CCNJ	0.0425254	0	0.0425254
CCNJL	0.0208053	0	0
CCNK	0.0600173	0.0600173	0
CCNL1	1	0	0
CCNL2	0.0173655	0.0173655	0
CCNO	0.0244968	0.0244968	0
CCNT1	0.1536109	0	0.1536109
CCNT2	0.062951	0	0
CCNY	0.0702474	0	0.0702474
CCNYL1	0.044369	0	0.044369
CCPG1	0.103239	0	0.103239
CCR1	0.0455405	0	0.0455405
CCR10	0.0577833	0	0.0577833
CCR3	0.0111884	0	0.0111884
CCR4	0.0217147	0	0.0217147
CCR6	0.0574536	0	0.0574536
CCR7	0.1893729	0	0.1893729
CCR8	0.0263102	0	0.0263102
CCR9	0.0347219	0	0.0347219

CCRL1	0.023819	0	0
CCRN4L	0.2801731	0	0.2801731
CCS	0.0064045	0.0064045	0
CCT2	0.0434773	0	0
CCT3	0.1500296	0.1500296	0
CCT4	0.044401	0.044401	0
CCT5	0.229122	0.229122	0
CCT6A	0.0605349	0.0605349	0
CCT6B	0.0283597	0	0
CCT6P1	0.0165427	0.0165427	0
CCT7	0.0339673	0.0339673	0
CCT8	0.1502052	0.1502052	0
CCT8L2	0.0003958	0	0
CD101	0.0499397	0	0.0499397
CD109	0.2763241	0	0.2763241
CD14	0.2132085	0	0.2132085
CD151	0.0042885	0.0042885	0
CD160	0.0169056	0	0.0169056
CD163	0.0329428	0	0.0329428
CD163L1	0.0094003	0	0.0094003
CD164	0.31867	0	0
CD164L2	0.0028821	0.0028821	0
CD177	0.0017372	0	0.0017372
CD180	0.0364203	0	0.0364203
CD19	0.015059	0	0.015059
CD1A	0.0169876	0	0.0169876
CD1B	0.0105622	0	0.0105622
CD1C	0.0342504	0	0.0342504
CD1D	0.0260375	0	0.0260375
CD1E	0.0073106	0	0.0073106
CD2	0.0119629	0	0.0119629
CD200	0.0480203	0	0.0480203
CD200R1	0.0607344	0	0.0607344
CD200R1L	0.0137925	0	0.0137925
CD207	0.0019589	0	0.0019589
CD209	0.0171902	0	0.0171902
CD22	0.0585582	0	0.0585582
CD226	0.0271688	0	0.0271688
CD24	1.53E-05	0	0
CD244	0.0243504	0	0.0243504
CD247	0.0203959	0	0.0203959
CD248	0.0132161	0	0.0132161
CD27	0.0270398	0	0.0270398
CD274	0.8237551	0	0.8237551
CD276	0.0311354	0	0.0311354

CD28	0.0072389	0	0.0072389
CD2AP	0.0991729	0.0991729	0
CD2BP2	0.060669	0	0
CD300A	0.0253937	0	0.0253937
CD300C	0.0022424	0	0.0022424
CD300E	0.0470876	0	0.0470876
CD300LB	0.006604	0	0.006604
CD300LD	0.0097543	0	0.0097543
CD300LF	0.0028629	0	0.0028629
CD300LG	0.0071669	0	0.0071669
CD302	0.0133222	0	0.0133222
CD320	0.0096212	0	0
CD33	0.0082773	0	0.0082773
CD34	0.0280581	0	0.0280581
CD36	0.1348654	0	0.1348654
CD37	0.036343	0	0.036343
CD38	0.129072	0	0.129072
CD3D	0.0056102	0	0.0056102
CD3E	0.0126064	0	0.0126064
CD3EAP	0.1036288	0	0.1036288
CD3G	0.0061213	0	0.0061213
CD4	0.0033297	0	0.0033297
CD40	0.0527784	0	0.0527784
CD40LG	0.0040153	0	0.0040153
CD44	0.3226932	0	0.3226932
CD46	0.9276665	0.9276665	0
CD47	0.0479695	0	0.0479695
CD48	0.1716717	0	0.1716717
CD5	0.0053394	0	0.0053394
CD52	0.0203662	0	0.0203662
CD53	0.249777	0	0.249777
CD55	1	1	0
CD58	0.1107144	0.1107144	0
CD59	1	0	0
CD5L	0.0128735	0	0.0128735
CD6	0.0082282	0	0.0082282
CD63	0.1622165	0.1622165	0
CD68	0.1687369	0	0.1687369
CD69	0.1593412	0	0.1593412
CD7	0.0009621	0	0.0009621
CD70	0.0681463	0	0.0681463
CD72	0.0069944	0	0.0069944
CD74	0.1165807	0	0.1165807
CD79A	0.0423652	0	0.0423652
CD79B	0.0037676	0	0.0037676

CD80	0.0504593	0	0.0504593
CD81	0.0015103	0	0.0015103
CD82	0.1954557	0.1954557	0
CD83	0.0981703	0	0.0981703
CD84	0.0630346	0	0.0630346
CD86	0.1111091	0	0.1111091
CD8A	0.0092142	0	0.0092142
CD8B	0.0001111	0	0.0001111
CD9	0.0294995	0	0
CD93	0.1053916	0	0.1053916
CD96	0.0721782	0	0.0721782
CD97	0.0533716	0.0533716	0
CD99L2	0.0045946	0	0.0045946
CDA	0.0069564	0	0
CDADC1	0.0338272	0	0.0338272
CDAN1	0.0159121	0	0
CDC123	0.0556217	0	0
CDC14A	0.0426355	0	0.0426355
CDC14B	0.0378025	0.0378025	0
CDC14C	0.0008714	0	0
CDC16	0.033492	0.033492	0
CDC20	0.0386977	0.0386977	0
CDC20B	0.0348287	0	0.0348287
CDC23	0.0477179	0	0
CDC25A	0.0357947	0.0357947	0
CDC25B	0.0073618	0	0
CDC25C	0.0697209	0.0697209	0
CDC26	0.0903175	0	0
CDC27	0.1263923	0	0.1263923
CDC34	0.0025201	0.0025201	0
CDC37	0.0305025	0.0305025	0
CDC37L1	0.3478111	0	0.3478111
CDC40	0.1295471	0	0.1295471
CDC42	0.0433638	0	0.0433638
CDC42BPA	0.1720825	0.1720825	0
CDC42BPB	0.0391022	0.0391022	0
CDC42BPG	0.0164303	0.0164303	0
CDC42EP1	0.0151844	0.0151844	0
CDC42EP2	0.0827267	0	0
CDC42EP3	0.047022	0	0.047022
CDC42EP4	0.5847149	0.5847149	0
CDC42EP5	0.093243	0.093243	0
CDC42SE1	0.1184885	0	0.1184885
CDC42SE2	0.180429	0	0.180429
CDC45	0.0614553	0.0614553	0

CDC5L	0.0868181	0	0.0868181
CDC6	0.0919753	0.0919753	0
CDC7	0.7344788	0	0.7344788
CDC73	0.2259505	0	0
CDCA2	0.1061248	0.1061248	0
CDCA3	0.0278151	0.0278151	0
CDCA4	0.0926015	0.0926015	0
CDCA5	0.0419602	0.0419602	0
CDCA7	0.0516314	0.0516314	0
CDCA7L	0.1107179	0.1107179	0
CDCA8	0.0409512	0.0409512	0
CDCP1	0.2830834	0.2830834	0
CDCP2	0.0075588	0	0.0075588
CDH1	0.0136084	0.0136084	0
CDH10	0.0498934	0	0.0498934
CDH11	0.0033719	0	0.0033719
CDH12	0.0261298	0	0
CDH13	0.1124795	0	0.1124795
CDH15	0.0006603	0	0.0006603
CDH16	0.006406	0.006406	0
CDH17	0.0352461	0	0
CDH18	0.0261084	0	0.0261084
CDH19	0.0078494	0	0.0078494
CDH2	0.0108582	0	0.0108582
CDH20	0.0059397	0	0.0059397
CDH22	0.0063947	0	0.0063947
CDH23	0.003865	0	0.003865
CDH24	0.010976	0	0
CDH26	0.0153484	0	0.0153484
CDH3	0.1711134	0	0
CDH4	0.0025619	0	0
CDH5	0.0030445	0	0.0030445
CDH6	0.0291775	0	0.0291775
CDH7	0.0095701	0	0.0095701
CDH8	0.0053392	0	0.0053392
CDH9	0.0210655	0	0
CDHR1	0.0042069	0	0.0042069
CDHR2	0.4901503	0.4901503	0
CDHR3	0.0021683	0.0021683	0
CDHR4	0.0052106	0	0
CDHR5	0.0064922	0.0064922	0
CDIPT	0.0282452	0.0282452	0
CDK1	0.127776	0.127776	0
CDK10	0.0031067	0.0031067	0
CDK11A	0.0086685	0.0086685	0

CDK11B	0.0087416	0.0087416	0
CDK12	0.0810589	0	0.0810589
CDK13	0.0890306	0	0
CDK14	0.082624	0	0.082624
CDK15	0.0127013	0	0.0127013
CDK16	0.1520134	0.1520134	0
CDK17	0.1801647	0	0.1801647
CDK18	0.0302389	0.0302389	0
CDK19	0.0966073	0	0.0966073
CDK2	0.1295722	0	0
CDK20	0.0045234	0	0
CDK2AP1	0.004505	0	0.004505
CDK2AP2	0.0419251	0.0419251	0
CDK3	0.0036095	0.0036095	0
CDK4	0.1268757	0	0
CDK5	0.0203064	0.0203064	0
CDK5R1	0.0094415	0	0.0094415
CDK5R2	0.0052475	0	0
CDK5RAP1	0.0228928	0.0228928	0
CDK5RAP2	0.074468	0	0.074468
CDK5RAP3	0.0303586	0.0303586	0
CDK6	0.1401405	0	0
CDK8	0.0146293	0	0
CDK9	0.0348342	0.0348342	0
CDKAL1	0.1651457	0	0.1651457
CDKL1	0.0426316	0	0.0426316
CDKL2	0.004703	0	0
CDKL3	0.1490108	0	0.1490108
CDKL4	0.0338469	0	0
CDKL5	0.0111533	0	0.0111533
CDKN1A	0.2855869	0	0
CDKN1B	0.3389322	0	0.3389322
CDKN1C	0.0057501	0	0
CDKN2A	0.0408754	0	0
CDKN2AIP	0.0702053	0.0702053	0
CDKN2AIPNL	0.0417201	0.0417201	0
CDKN2B	0.0097494	0	0.0097494
CDKN2BAS	0.0390766	0	0
CDKN2C	0.2200069	0.2200069	0
CDKN2D	0.0420469	0.0420469	0
CDKN3	0.0103531	0.0103531	0
CDNF	0.0408232	0	0
CDO1	0.008752	0	0.008752
CDON	0.0324091	0	0.0324091
CDR1	0.0050888	0	0.0050888

CDR2	0.0389376	0	0.0389376
CDR2L	0.0101893	0.0101893	0
CDRT1	0.0003018	0	0
CDRT15	0.0007007	0	0
CDRT15P	0.0006515	0	0.0006515
CDRT4	0.0065714	0.0065714	0
CDS1	0.0089342	0.0089342	0
CDS2	0.0493538	0	0.0493538
CDT1	0.0100559	0.0100559	0
CDV3	0.2567617	0	0.2567617
CDX1	0.0101819	0	0.0101819
CDX2	0.0004475	0.0004475	0
CDX4	0.0051405	0.0051405	0
CDYL	0.0641142	0	0.0641142
CDYL2	0.0220699	0	0.0220699
CEACAM1	0.0076278	0.0076278	0
CEACAM16	0.0056422	0.0056422	0
CEACAM18	0.0066076	0	0
CEACAM19	0.0080665	0.0080665	0
CEACAM20	0.002992	0	0
CEACAM21	0.0042794	0	0.0042794
CEACAM22P	0.010976	0	0
CEACAM3	0.003466	0	0.003466
CEACAM4	0.0011003	0	0.0011003
CEACAM5	0.0016242	0.0016242	0
CEACAM6	0.0095378	0	0
CEACAM7	0.0017434	0	0.0017434
CEACAM8	0.002543	0	0.002543
CEBPA	0.0087699	0	0.0087699
CEBPB	0.0674335	0	0
CEBPD	0.0969271	0	0
CEBPE	0.0679524	0	0.0679524
CEBPG	0.0909393	0.0909393	0
CEBPZ	0.0730915	0	0
CECR1	0.0055303	0	0.0055303
CECR2	0.0110991	0	0.0110991
CECR4	0.0071607	0.0071607	0
CECR5	0.0085801	0.0085801	0
CECR6	0.0013113	0	0.0013113
CECR7	0.003929	0	0.003929
CEL	0.0006174	0	0.0006174
CELA1	0.0101344	0	0.0101344
CELA2A	0.0684392	0	0.0684392
CELA2B	0.0028863	0	0.0028863
CELA3A	0.0008765	0	0.0008765

CELA3B	0.0004956	0	0.0004956
CELF1	0.2626821	0	0
CELF2	0.0895149	0	0.0895149
CELF3	0.0050711	0	0.0050711
CELF4	0.0026913	0	0.0026913
CELF5	0.001348	0	0
CELF6	0.010987	0	0
CELP	0.0032176	0	0
CELSR1	0.0297038	0.0297038	0
CELSR2	0.0080219	0	0
CELSR3	0.0094398	0.0094398	0
CEMP1	0.0075325	0.0075325	0
CEND1	0.0011602	0	0.0011602
CENPA	0.0410456	0.0410456	0
CENPB	0.0110387	0.0110387	0
CENPBD1	0.0148845	0	0
CENPC1	0.0472762	0	0.0472762
CENPE	0.0240938	0.0240938	0
CENPF	0.0697006	0.0697006	0
CENPI	0.0090355	0	0
CENPJ	0.05747	0	0
CENPK	0.0647984	0.0647984	0
CENPL	0.1398509	0	0
CENPM	0.0656779	0.0656779	0
CENPN	0.1668838	0	0
CENPO	0.0294227	0	0
CENPP	0.2265735	0	0.2265735
CENPQ	0.0775535	0	0.0775535
CENPT	0.0217883	0.0217883	0
CENPV	0.0064324	0.0064324	0
CEP110	0.0365005	0	0.0365005
CEP120	0.3314025	0	0.3314025
CEP135	0.1708985	0	0.1708985
CEP152	0.1599159	0	0
CEP164	0.0269823	0	0
CEP170	0.4202458	0	0.4202458
CEP170L	0.0006011	0	0.0006011
CEP192	0.0815647	0	0
CEP250	0.0951158	0	0
CEP290	0.1106273	0	0.1106273
CEP350	0.1552009	0	0.1552009
CEP55	0.0445722	0.0445722	0
CEP57	0.1724592	0	0
CEP63	0.5258016	0	0.5258016
CEP68	0.058338	0	0.058338

CEP70	0.0671336	0.0671336	0
CEP72	0.0239514	0.0239514	0
CEP76	0.0401487	0	0
CEP78	0.0309265	0	0.0309265
CEP97	0.0619146	0	0.0619146
CEPT1	0.1536045	0	0.1536045
CER1	0.0060504	0	0.0060504
CERCAM	0.0052041	0	0.0052041
CERK	0.0044331	0	0.0044331
CERKL	0.0046888	0	0.0046888
CES1	0.0164594	0	0.0164594
CES2	0.0270376	0.0270376	0
CES3	0.0149488	0.0149488	0
CES4	0.0044536	0	0.0044536
CES7	0.0031895	0	0.0031895
CES8	0.0199276	0	0
CETN1	0.0031209	0	0
CETN2	0.0015891	0.0015891	0
CETN3	0.0765307	0	0
CETN4P	0.0280344	0	0
CETP	0.0182007	0	0.0182007
CFD	0.0133026	0	0.0133026
CFDP1	0.0296673	0.0296673	0
CFH	0.1612003	0	0.1612003
CFHR1	0.0028749	0	0.0028749
CFHR2	0.0027717	0.0027717	0
CFHR3	0.0089148	0	0.0089148
CFHR4	0.021851	0	0.021851
CFHR5	0.0168347	0	0
CFI	0.1467416	0	0.1467416
CFL1	0.1115619	0.1115619	0
CFL2	0.2959196	0	0.2959196
CFLAR	0.4724246	0	0.4724246
CFLP1	0.1253832	0	0
CFP	0.0114677	0	0.0114677
CFTR	0.0296792	0	0.0296792
CG030	0.0134021	0.0134021	0
CGA	0.0270292	0	0.0270292
CGB	0.0006201	0	0
CGB1	0.0001946	0.0001946	0
CGB2	0.0001548	0.0001548	0
CGB5	0.0002387	0	0
CGB7	0.0009606	0.0009606	0
CGB8	0.0002959	0	0
CGGBP1	0.0564702	0	0.0564702

CGN	0.0340068	0.0340068	0
CGNL1	0.006605	0	0.006605
CGREF1	0.0036807	0	0.0036807
CGRRF1	0.0265016	0	0.0265016
CH25H	0.054337	0	0.054337
CHAC1	0.022564	0.022564	0
CHAC2	0.076573	0	0
CHAD	0.0025939	0.0025939	0
CHADL	0.0021856	0.0021856	0
CHAF1A	0.0422648	0.0422648	0
CHAF1B	0.0167398	0	0
CHAT	0.0058335	0	0.0058335
CHCHD1	0.0515453	0.0515453	0
CHCHD10	0.0062998	0.0062998	0
CHCHD2	0.0350045	0.0350045	0
CHCHD3	0.0461694	0.0461694	0
CHCHD4	0.0734507	0.0734507	0
CHCHD5	0.0348074	0.0348074	0
CHCHD6	0.0124401	0.0124401	0
CHCHD7	0.0528727	0.0528727	0
CHCHD8	0.1082822	0.1082822	0
CHD1	0.3911661	0	0.3911661
CHD1L	0.0955652	0	0
CHD2	1	0	0
CHD3	0.0590991	0	0.0590991
CHD4	0.0435522	0	0
CHD5	0.0030167	0	0
CHD6	0.0418082	0	0.0418082
CHD7	0.0430568	0.0430568	0
CHD8	0.0389046	0	0.0389046
CHD9	0.0847121	0	0.0847121
CHDH	0.0143562	0.0143562	0
CHEK1	0.0954215	0.0954215	0
CHEK2	0.5944461	0	0
CHERP	0.0168313	0.0168313	0
CHFR	0.0035916	0	0
CHGA	0.0049582	0	0
CHGB	0.0161569	0	0.0161569
CHI3L1	0.3013758	0	0.3013758
CHI3L2	0.7347585	0	0.7347585
CHIA	0.0081228	0	0
CHIC1	0.0307449	0	0.0307449
CHIC2	0.2966688	0	0.2966688
CHID1	0.0016909	0.0016909	0
CHIT1	0.0099324	0	0.0099324

CHKA	0.0479261	0.0479261	0
CHKB	0.0083475	0	0
CHKB-CPT1B	0.0083475	0.0083475	0
CHL1	0.002987	0	0.002987
CHM	0.0572326	0	0.0572326
CHML	0.5448074	0	0
CHMP1A	0.0045226	0.0045226	0
CHMP1B	0.3156469	0	0
CHMP2A	0.0337824	0.0337824	0
CHMP2B	0.1495706	0	0
CHMP4A	0.0492496	0.0492496	0
CHMP4B	0.1020725	0.1020725	0
CHMP4C	0.0570748	0.0570748	0
CHMP5	0.0654941	0.0654941	0
CHMP6	0.0025225	0	0
CHMP7	0.032735	0	0.032735
CHN1	0.0197323	0	0.0197323
CHN2	0.019156	0	0
CHODL	0.0057412	0	0.0057412
CHORDC1	0.1260182	0	0
CHP	0.0522809	0.0522809	0
CHP2	0.0015012	0	0.0015012
CHPF	0.0024123	0	0
CHPF2	0.0147629	0	0
CHPT1	0.0135446	0	0
CHRAC1	0.0833445	0.0833445	0
CHRD	0.0028637	0	0.0028637
CHRDL1	0.005255	0	0.005255
CHRDL2	0.0153321	0	0.0153321
CHRFAM7A	1.53E-05	0	1.53E-05
CHRM1	0.0031706	0	0
CHRM2	0.003621	0	0.003621
CHRM3	0.0031524	0	0
CHRM4	0.0051955	0	0.0051955
CHRM5	0.0150855	0	0.0150855
CHRNA1	0.0090566	0	0.0090566
CHRNA10	0.0145117	0	0
CHRNA2	0.0050281	0	0
CHRNA3	0.001939	0	0.001939
CHRNA4	0.0003337	0	0
CHRNA5	0.0287128	0.0287128	0
CHRNA6	0.0252055	0	0.0252055
CHRNA7	0.0042283	0	0.0042283
CHRNA9	0.1571811	0	0
CHRNB1	0.0450248	0	0

CHRNB2	0.0378114	0	0.0378114
CHRNB3	0.008289	0	0.008289
CHRNB4	0.0042437	0	0.0042437
CHRND	0.0003516	0	0
CHRNE	0.013534	0	0.013534
CHRNG	0.0012933	0.0012933	0
CHST1	0.002564	0	0.002564
CHST10	0.0133772	0	0.0133772
CHST11	0.1003195	0	0.1003195
CHST12	0.04779	0	0
CHST13	0.0022274	0	0.0022274
CHST14	0.0075744	0	0.0075744
CHST15	0.0127695	0	0.0127695
CHST2	0.024872	0	0.024872
CHST3	0.0489799	0	0.0489799
CHST4	0.0082156	0	0
CHST5	0.0031836	0.0031836	0
CHST6	0.0196773	0.0196773	0
CHST7	0.0257463	0	0.0257463
CHST8	0.0008008	0	0.0008008
CHST9	0.0079167	0	0
CHSY1	0.0613635	0	0.0613635
CHSY3	0.0158862	0	0.0158862
CHTF18	0.0006661	0.0006661	0
CHTF8	0.0414596	0	0
CHUK	0.044742	0	0.044742
CHURC1	0.1891922	0	0.1891922
CIAO1	0.0346285	0	0
CIAPIN1	0.0302508	0	0
CIB1	0.0453847	0.0453847	0
CIB2	0.0085364	0	0
CIB3	0.0122172	0	0
CIB4	0.0033259	0.0033259	0
CIC	0.0168892	0	0
CIDEA	0.0032866	0	0.0032866
CIDEB	0.3068135	0	0
CIDEC	0.0185991	0	0.0185991
CIDECP	0.0296363	0	0
CIITA	0.3562318	0	0.3562318
CILP	0.0046895	0	0.0046895
CILP2	0.001187	0	0.001187
CINP	0.0210135	0.0210135	0
CIR1	0.040988	0	0
CIRBP	0.135669	0	0
CIRH1A	0.0401746	0	0

CISD1	0.3817894	0	0
CISD2	0.0766164	0.0766164	0
CISD3	0.008205	0.008205	0
CISH	0.1369179	0	0.1369179
CIT	0.0566932	0.0566932	0
CITED1	0.0033503	0.0033503	0
CITED2	0.1864002	0	0.1864002
CITED4	0.1410837	0.1410837	0
CIZ1	0.0157898	0.0157898	0
CKAP2	0.0747003	0	0
CKAP2L	0.0628799	0.0628799	0
CKAP4	0.0239974	0	0.0239974
CKAP5	0.0913039	0	0
CKB	0.0042734	0.0042734	0
CKLF	0.0604209	0.0604209	0
CKM	0.0019894	0	0.0019894
CKMT1A	5.18E-05	5.18E-05	0
CKMT1B	0.0001007	0.0001007	0
CKMT2	0.0076403	0	0.0076403
CKS1B	1	1	0
CKS2	0.2756992	0.2756992	0
CLASP1	0.1366156	0	0.1366156
CLASP2	0.0552661	0	0.0552661
CLC	0.0089803	0	0.0089803
CLCA1	0.0113998	0	0
CLCA2	0.3588752	0	0.3588752
CLCA3P	0.0019525	0	0
CLCA4	0.0684877	0	0
CLCC1	0.0505668	0	0
CLCF1	0.045196	0	0
CLCN1	0.0108028	0.0108028	0
CLCN2	0.0237792	0.0237792	0
CLCN3	0.140183	0	0
CLCN4	0.0226815	0	0.0226815
CLCN5	0.0258811	0	0
CLCN6	0.1532634	0	0.1532634
CLCN7	0.0044827	0.0044827	0
CLCNKA	0.0025336	0	0
CLCNKB	0.0009226	0	0
CLDN1	0.1342657	0	0
CLDN10	0.0150086	0	0.0150086
CLDN11	0.0139355	0	0.0139355
CLDN12	0.0812763	0.0812763	0
CLDN14	0.1050668	0	0
CLDN15	0.0046797	0	0.0046797

CLDN16	0.3726567	0	0.3726567
CLDN17	0.0116254	0	0
CLDN18	0.0259415	0.0259415	0
CLDN19	0.0033528	0	0.0033528
CLDN2	0.0110514	0	0
CLDN20	0.0130985	0	0
CLDN22	0.0068562	0	0
CLDN23	0.0170335	0	0
CLDN25	0.014589	0	0
CLDN3	0.00107	0.00107	0
CLDN4	0.0257893	0.0257893	0
CLDN5	0.0007759	0	0.0007759
CLDN6	0.0069429	0	0.0069429
CLDN7	0.0180294	0.0180294	0
CLDN8	0.0074156	0	0
CLDN9	0.0026219	0.0026219	0
CLDND1	0.2260781	0	0.2260781
CLDND2	0.0164589	0.0164589	0
CLEC10A	0.0046657	0	0.0046657
CLEC11A	0.0010291	0	0.0010291
CLEC12A	0.0073984	0	0.0073984
CLEC12B	0.03244	0	0.03244
CLEC14A	0.0117371	0	0.0117371
CLEC16A	0.1052984	0	0
CLEC17A	0.0409514	0	0.0409514
CLEC18A	4.05E-05	4.05E-05	0
CLEC18B	5.85E-05	0	0
CLEC18C	3.02E-05	3.02E-05	0
CLEC1A	0.0161236	0	0.0161236
CLEC1B	0.0116896	0	0
CLEC2A	0.029343	0	0
CLEC2B	0.1781103	0	0.1781103
CLEC2D	0.0885409	0	0.0885409
CLEC2L	0.0012005	0	0.0012005
CLEC3A	0.0074624	0	0
CLEC3B	0.0102195	0	0.0102195
CLEC4A	0.0068277	0	0.0068277
CLEC4C	0.0040679	0	0.0040679
CLEC4D	0.0116913	0	0.0116913
CLEC4E	0.0187706	0	0.0187706
CLEC4F	0.0029664	0	0.0029664
CLEC4G	0.0036157	0	0.0036157
CLEC4GP1	0.0370938	0	0.0370938
CLEC4M	0.0081042	0	0.0081042
CLEC5A	0.0100567	0	0.0100567

CLEC6A	0.0097539	0	0.0097539
CLEC7A	0.0427456	0	0.0427456
CLEC9A	0.3552124	0	0.3552124
CLECL1	0.0831859	0	0.0831859
CLGN	0.019131	0	0.019131
CLIC2	0.0231215	0	0.0231215
CLIC3	0.0084364	0.0084364	0
CLIC4	0.3625674	0	0.3625674
CLIC5	0.053249	0	0.053249
CLIC6	0.012772	0	0.012772
CLINT1	0.0923141	0.0923141	0
CLIP1	0.2521138	0	0.2521138
CLIP2	0.0315442	0.0315442	0
CLIP3	0.0021701	0	0.0021701
CLIP4	0.6475609	0	0.6475609
CLK1	0.071223	0	0
CLK2	0.0532113	0.0532113	0
CLK2P	0.0163647	0.0163647	0
CLK3	0.0129109	0.0129109	0
CLK4	0.0880999	0	0.0880999
CLLU1	0.262711	0	0.262711
CLLU1OS	0.1988921	0	0.1988921
CLMN	0.1016392	0.1016392	0
CLN3	0.0182761	0.0182761	0
CLN5	0.0512743	0	0.0512743
CLN6	0.0156295	0.0156295	0
CLN8	0.0161867	0	0.0161867
CLNK	0.0373335	0	0.0373335
CLNS1A	0.0796152	0.0796152	0
CLOCK	0.0561516	0	0.0561516
CLP1	0.058825	0	0.058825
CLPB	0.0738974	0	0
CLPP	0.0071222	0.0071222	0
CLPS	0.0051282	0	0.0051282
CLPTM1	0.0214476	0.0214476	0
CLPTM1L	0.0099638	0.0099638	0
CLPX	0.2455729	0	0.2455729
CLRN1	0.015035	0	0
CLRN1OS	0.0123215	0.0123215	0
CLRN2	0.0155829	0.0155829	0
CLRN3	0.0090678	0.0090678	0
CLSPN	0.0242417	0	0.0242417
CLSTN1	0.0290691	0.0290691	0
CLSTN2	0.0028307	0	0.0028307
CLSTN3	0.0046233	0	0.0046233

CLTA	0.0371284	0.0371284	0
CLTB	0.0473941	0.0473941	0
CLTC	0.1344808	0	0.1344808
CLTCL1	0.0195703	0	0.0195703
CLU	0.0861199	0	0.0861199
CLUAP1	0.0196739	0	0
CLUL1	0.0164789	0	0
CLVS1	0.007098	0	0
CLVS2	0.0025586	0	0.0025586
CLYBL	0.0228511	0.0228511	0
CMA1	0.0066112	0	0.0066112
CMAH	0.1759493	0	0.1759493
CMAS	0.0322977	0.0322977	0
CMBL	0.2424311	0.2424311	0
CMC1	0.0799681	0.0799681	0
CMIP	0.0233276	0.0233276	0
CMKLR1	0.0237326	0	0.0237326
CMPK1	0.0634154	0.0634154	0
CMPK2	0.0161236	0	0.0161236
CMTM1	0.0326791	0	0
CMTM2	0.0229015	0	0.0229015
CMTM3	0.0049703	0	0.0049703
CMTM4	0.0115502	0.0115502	0
CMTM5	0.0024124	0	0.0024124
CMTM6	0.0847388	0	0
CMTM7	0.011232	0	0
CMTM8	0.0056115	0.0056115	0
CMYA5	0.0160658	0	0.0160658
CN5H6.4	0.0051763	0	0
CNBD1	0.0080953	0	0
CNBP	0.0603532	0	0
CNDP1	0.004658	0	0
CNDP2	0.0473394	0	0.0473394
CNFN	0.0078081	0.0078081	0
CNGA1	0.0160407	0	0
CNGA2	0.0011094	0	0
CNGA3	0.004821	0	0.004821
CNGA4	0.0294416	0.0294416	0
CNGB1	0.0039432	0	0.0039432
CNGB3	0.010463	0	0
CNIH	0.2122028	0	0
CNIH2	0.009365	0	0.009365
CNIH3	0.0061522	0	0
CNIH4	0.1114272	0.1114272	0
CNKSR1	0.0055196	0.0055196	0

CNKSR2	0.007193	0	0.007193
CNKSR3	0.0099041	0	0.0099041
CNN1	0.0124203	0	0.0124203
CNN2	0.0467674	0	0.0467674
CNN3	0.0147906	0	0.0147906
CNNM1	0.0040382	0.0040382	0
CNNM2	0.0383154	0.0383154	0
CNNM3	0.0072396	0.0072396	0
CNNM4	0.0183517	0.0183517	0
CNO	0.0354822	0.0354822	0
CNOT1	0.0687187	0	0
CNOT10	0.0375712	0	0
CNOT2	0.0992066	0	0
CNOT3	0.006228	0.006228	0
CNOT4	0.0660996	0	0
CNOT6	0.060854	0	0
CNOT6L	0.044674	0	0.044674
CNOT7	0.1021731	0	0.1021731
CNOT8	0.1003748	0	0.1003748
CNP	0.025509	0	0.025509
CNPY1	0.0020537	0	0
CNPY2	0.0420499	0.0420499	0
CNPY3	0.0483381	0	0.0483381
CNPY4	0.0430006	0	0.0430006
CNR1	0.0346661	0	0.0346661
CNR2	0.0597322	0	0.0597322
CNRIP1	0.0105967	0	0.0105967
CNST	0.2092124	0	0.2092124
CNTD1	0.0135076	0.0135076	0
CNTD2	0.0114898	0.0114898	0
CNTF	0.0553618	0	0.0553618
CNTFR	0.003128	0	0.003128
CNTLN	0.0205988	0	0.0205988
CNTN1	0.0123113	0	0.0123113
CNTN2	0.0040285	0	0.0040285
CNTN3	0.0062283	0	0
CNTN4	0.0132739	0	0.0132739
CNTN5	0.0195178	0	0.0195178
CNTN6	0.0072119	0	0.0072119
CNTNAP1	0.069216	0	0.069216
CNTNAP2	0.0043689	0	0.0043689
CNTNAP3	1.56E-05	0	0
CNTNAP4	0.0145663	0	0.0145663
CNTNAP5	0.0047489	0	0.0047489
CNTROB	0.0121352	0	0

COASY	0.0613347	0.0613347	0
COBL	0.0988286	0.0988286	0
COBLL1	0.0357482	0	0.0357482
COBRA1	0.0025216	0.0025216	0
COCH	0.009921	0	0
COG1	0.0197944	0.0197944	0
COG2	0.0678658	0.0678658	0
COG3	0.0492591	0.0492591	0
COG4	0.0755234	0.0755234	0
COG5	0.1761722	0	0.1761722
COG6	0.0158331	0	0
COG7	0.017085	0.017085	0
COG8	0.0415352	0	0.0415352
COIL	0.0203564	0.0203564	0
COL10A1	0.0261023	0	0.0261023
COL11A1	0.0114232	0	0.0114232
COL12A1	0.0827012	0	0.0827012
COL13A1	0.0078458	0	0.0078458
COL14A1	0.0154738	0	0.0154738
COL15A1	0.0055169	0	0.0055169
COL16A1	0.0192841	0	0.0192841
COL17A1	0.2175961	0	0
COL18A1	0.0051144	0	0.0051144
COL19A1	0.0405592	0	0.0405592
COL1A1	0.0120313	0	0.0120313
COL1A2	0.0177507	0	0.0177507
COL20A1	0.0007646	0.0007646	0
COL21A1	0.0209212	0	0.0209212
COL22A1	0.0035024	0	0.0035024
COL23A1	0.0059294	0	0.0059294
COL24A1	0.0027807	0	0.0027807
COL25A1	0.0658484	0	0.0658484
COL27A1	0.0025641	0.0025641	0
COL28A1	0.0181265	0	0
COL29A1	0.0486805	0	0.0486805
COL2A1	0.0067834	0	0
COL3A1	0.0222849	0	0.0222849
COL4A1	0.0054986	0	0.0054986
COL4A2	0.0054644	0	0.0054644
COL4A3	0.0085467	0	0.0085467
COL4A3BP	0.1583391	0	0.1583391
COL4A4	0.0085662	0	0.0085662
COL4A5	0.0555705	0	0.0555705
COL4A6	0.0545887	0	0.0545887
COL5A1	0.0110552	0	0.0110552

COL5A2	0.0119431	0	0.0119431
COL5A3	0.0019715	0	0.0019715
COL6A1	0.0011261	0	0.0011261
COL6A2	0.0030689	0	0.0030689
COL6A3	0.0065371	0	0.0065371
COL6A4P2	0.0014663	0	0
COL6A6	0.0083407	0	0.0083407
COL7A1	0.0109289	0	0
COL8A1	0.0676439	0	0.0676439
COL8A2	0.0020063	0	0.0020063
COL9A1	0.0167608	0	0
COL9A2	0.0049319	0.0049319	0
COL9A3	0.0016015	0.0016015	0
COLEC10	0.0787632	0	0
COLEC11	0.0031814	0	0.0031814
COLEC12	0.0074778	0	0.0074778
COLQ	0.0147581	0	0.0147581
COMMD1	0.0296814	0	0
COMMD10	0.0422437	0	0.0422437
COMMD2	0.1017959	0	0.1017959
COMMD3	0.0734289	0	0
COMMD4	0.0141635	0.0141635	0
COMMD5	0.0138947	0.0138947	0
COMMD6	0.0510369	0	0
COMMD7	0.0625658	0.0625658	0
COMMD8	0.0496715	0	0.0496715
COMMD9	0.139258	0	0
COMP	0.0013502	0	0.0013502
COMT	0.0107786	0.0107786	0
COMTD1	0.0322061	0.0322061	0
COPA	0.0867618	0	0
COPB1	0.0641254	0	0
COPB2	0.0845262	0	0
COPE	0.0280236	0.0280236	0
COPG	0.095422	0.095422	0
COPG2	0.0157803	0.0157803	0
COPS2	0.2585293	0	0.2585293
COPS3	0.0223727	0	0.0223727
COPS4	0.1185559	0	0.1185559
COPS5	0.1642907	0.1642907	0
COPS6	0.0339237	0.0339237	0
COPS7A	0.0250145	0.0250145	0
COPS7B	0.051764	0.051764	0
COPS8	0.0513376	0	0.0513376
COPZ1	0.1624398	0.1624398	0

COPZ2	0.0408795	0	0.0408795
COQ10A	0.0224537	0	0
COQ10B	0.1181395	0	0
COQ2	0.0623117	0	0
COQ3	0.0345175	0.0345175	0
COQ4	0.0666782	0.0666782	0
COQ5	0.1047041	0.1047041	0
COQ6	0.0184584	0.0184584	0
COQ7	0.0355838	0.0355838	0
COQ9	0.0303636	0.0303636	0
CORIN	0.0083963	0	0.0083963
CORO1A	0.0032505	0	0.0032505
CORO1B	0.0136444	0.0136444	0
CORO1C	0.1414482	0	0.1414482
CORO2A	0.0098389	0.0098389	0
CORO2B	0.0044165	0	0.0044165
CORO6	0.0113771	0	0.0113771
CORO7	0.0231324	0	0.0231324
CORT	0.0086512	0.0086512	0
COTL1	0.065086	0	0.065086
COX10	0.05912	0	0
COX11	0.0719248	0.0719248	0
COX15	0.0498756	0.0498756	0
COX16	0.1075721	0.1075721	0
COX17	0.0673311	0.0673311	0
COX18	0.131588	0	0
COX19	0.0079447	0.0079447	0
COX4I1	0.0102961	0.0102961	0
COX4I2	0.0031002	0	0.0031002
COX4NB	0.0103697	0.0103697	0
COX5A	0.0379617	0.0379617	0
COX5B	0.0301707	0.0301707	0
COX6A1	0.0468306	0.0468306	0
COX6A2	0.0047096	0	0
COX6B1	0.015131	0.015131	0
COX6B2	0.0085376	0	0
COX6C	0.0550526	0.0550526	0
COX7A1	0.0017275	0	0.0017275
COX7A2	0.0881933	0.0881933	0
COX7A2L	0.1010986	0.1010986	0
COX7B	0.1384854	0.1384854	0
COX7B2	0.0046456	0	0
COX7C	0.0918306	0.0918306	0
COX8A	0.0291182	0.0291182	0
COX8C	0.0089344	0.0089344	0

CP	0.1579115	0	0
CP110	0.1884484	0	0.1884484
CPA1	0.0047419	0	0.0047419
CPA2	0.0295376	0	0.0295376
CPA3	0.0279921	0	0.0279921
CPA4	0.1307428	0	0.1307428
CPA5	0.0130084	0	0.0130084
CPA6	0.0057388	0	0
CPAMD8	0.0054624	0	0.0054624
CPB1	0.0259828	0	0.0259828
CPB2	0.0158292	0.0158292	0
CPD	0.3053315	0.3053315	0
CPE	0.020202	0	0.020202
CPEB1	0.010162	0	0.010162
CPEB2	0.0927244	0	0.0927244
CPEB3	0.0933924	0	0.0933924
CPEB4	0.4958959	0	0.4958959
CPLX1	0.0002534	0.0002534	0
CPLX2	0.0063068	0	0
CPLX3	0.0090516	0	0
CPLX4	0.0134394	0	0.0134394
CPM	0.0502254	0	0.0502254
CPN1	0.0060457	0	0
CPN2	0.0066662	0	0
CPNE1	0.1612439	0.1612439	0
CPNE2	0.0077337	0	0.0077337
CPNE3	0.0670487	0	0.0670487
CPNE4	0.0151086	0	0
CPNE5	0.0016813	0	0.0016813
CPNE6	0.0019459	0	0.0019459
CPNE7	0.0033667	0.0033667	0
CPNE8	0.5765225	0	0.5765225
CPNE9	0.0037584	0	0.0037584
CPO	0.0058499	0	0
CPOX	0.0574872	0	0
CPPED1	0.0386114	0	0.0386114
CPS1	0.1834924	0	0.1834924
CPSF1	0.0212504	0.0212504	0
CPSF2	0.0302612	0	0.0302612
CPSF3	0.04101	0.04101	0
CPSF3L	0.0034383	0.0034383	0
CPSF4	0.0093194	0.0093194	0
CPSF4L	0.0050599	0.0050599	0
CPSF6	0.0606907	0.0606907	0
CPSF7	0.0642476	0	0

CPT1A	0.0215682	0	0
CPT1B	0.0035397	0.0035397	0
CPT1C	0.0014618	0	0.0014618
CPT2	0.0454995	0.0454995	0
CPVL	0.0218981	0	0.0218981
CPXCR1	0.0037477	0	0
CPXM1	0.0013722	0	0.0013722
CPXM2	0.0026645	0	0.0026645
CPZ	0.0008796	0	0.0008796
CR1	0.1219143	0	0.1219143
CR1L	0.0521845	0	0.0521845
CR2	0.1492976	0	0.1492976
CRABP1	0.0022055	0	0.0022055
CRABP2	0.0147956	0	0
CRADD	0.1621791	0	0
CRAMP1L	0.0114332	0	0
CRAT	0.0321816	0.0321816	0
CRB1	0.0204778	0	0.0204778
CRB2	0.0095427	0	0.0095427
CRB3	0.0073896	0.0073896	0
CRBN	0.0304129	0	0.0304129
CRCP	0.0215298	0.0215298	0
CRCT1	0.0479911	0	0
CREB1	0.0710737	0	0.0710737
CREB3	0.0855964	0.0855964	0
CREB3L1	0.0151206	0.0151206	0
CREB3L2	0.1752393	0	0.1752393
CREB3L3	0.0030452	0	0
CREB3L4	0.6172032	0.6172032	0
CREB5	0.1177601	0	0.1177601
CREBBP	0.0117387	0	0.0117387
CREBL2	0.1139278	0	0.1139278
CREBZF	0.1609068	0.1609068	0
CREG1	0.1763212	0	0.1763212
CREG2	0.0359859	0.0359859	0
CRELD1	0.0125011	0.0125011	0
CRELD2	0.0036588	0	0
CREM	0.2505554	0	0.2505554
CRH	0.015195	0	0.015195
CRHBP	0.0087164	0	0.0087164
CRHR1	0.0021837	0	0.0021837
CRHR2	0.0013896	0	0.0013896
CRIM1	0.1647487	0	0.1647487
CRIP1	0.0064214	0.0064214	0
CRIP2	0.0017738	0.0017738	0

CRIP3	0.0121356	0	0.0121356
CRIPAK	0.0009443	0.0009443	0
CRIPT	0.0641108	0.0641108	0
CRISP1	0.0088408	0.0088408	0
CRISP2	0.0080426	0	0
CRISP3	0.0176175	0	0
CRISPLD1	0.0053591	0	0.0053591
CRISPLD2	0.0281482	0	0.0281482
CRK	0.0383512	0	0.0383512
CRKL	0.067716	0	0.067716
CRLF1	0.0008886	0	0.0008886
CRLF3	0.0635439	0	0.0635439
CRLS1	0.1536368	0.1536368	0
CRMP1	0.0009522	0	0.0009522
CRNKL1	0.016645	0.016645	0
CRNN	0.0109866	0	0.0109866
CROCC	0.0059816	0	0
CROCCL1	0.1555633	0.1555633	0
CROCCL2	0.0013498	0.0013498	0
CROT	0.070285	0	0
CRP	0.0116293	0	0
CRTAC1	0.0024499	0	0.0024499
CRTAM	0.0051178	0	0.0051178
CRTAP	0.0331799	0	0.0331799
CRTC1	0.0084269	0	0
CRTC2	0.4338682	0.4338682	0
CRTC3	0.0369968	0	0.0369968
CRX	0.0009752	0	0
CRY1	0.033921	0	0.033921
CRY2	0.0437822	0	0.0437822
CRYAA	0.0658407	0	0
CRYAB	0.0058718	0	0.0058718
CRYBA1	0.0992188	0.0992188	0
CRYBA2	0.0015166	0	0
CRYBA4	0.0027681	0	0
CRYBB1	0.0048829	0	0.0048829
CRYBB2	0.0017025	0	0
CRYBB3	0.003073	0.003073	0
CRYBG3	0.1044666	0	0.1044666
CRYGA	0.0035026	0	0
CRYGB	0.0041462	0	0
CRYGC	0.0081874	0	0
CRYGD	0.0036743	0	0.0036743
CRYGN	0.0029977	0	0.0029977
CRYGS	0.0492153	0.0492153	0

CRYL1	0.0164259	0.0164259	0
CRYM	0.1044737	0.1044737	0
CRYZ	0.0887273	0	0
CRYZL1	0.0848322	0	0.0848322
CS	0.1201511	0.1201511	0
CSAD	0.0517037	0.0517037	0
CSAG1	0.0009757	0	0.0009757
CSDA	0.8487777	0	0.8487777
CSDAP1	0.002479	0	0.002479
CSDC2	0.0018294	0	0.0018294
CSDE1	0.2006018	0	0.2006018
CSE1L	0.0780934	0.0780934	0
CSF1	0.0666131	0	0.0666131
CSF1R	0.0124119	0	0.0124119
CSF2	0.0100386	0	0.0100386
CSF2RB	0.02518	0	0.02518
CSF3	0.2283332	0	0.2283332
CSF3R	0.0333055	0	0.0333055
CSGALNACT1	0.1963498	0	0.1963498
CSGALNACT2	0.0544653	0	0.0544653
CSH1	0.0011095	0	0
CSH2	0.0018065	0	0
CSHL1	0.007392	0	0
CSK	0.1134932	0	0.1134932
CSMD1	0.0023575	0	0.0023575
CSMD2	0.0012181	0	0.0012181
CSMD3	0.0290646	0	0.0290646
CSN1S1	0.0043985	0	0.0043985
CSN1S2A	0.0053951	0	0
CSN2	0.0044522	0.0044522	0
CSN3	0.0131854	0	0.0131854
CSNK1A1	0.1594202	0.1594202	0
CSNK1A1L	0.0064571	0	0.0064571
CSNK1A1P	0.0083269	0	0
CSNK1D	0.0136549	0.0136549	0
CSNK1E	0.1736055	0.1736055	0
CSNK1G1	0.0579887	0	0.0579887
CSNK1G2	0.0127415	0.0127415	0
CSNK1G3	0.1289511	0	0
CSNK2A1	0.0535466	0.0535466	0
CSNK2A1P	0.0133609	0	0
CSNK2A2	0.0183626	0	0
CSPG4	0.0042976	0	0.0042976
CSPG5	0.0037058	0	0
CSPP1	0.1165761	0	0

CSRNP1	0.0707662	0	0.0707662
CSRNP2	0.023164	0	0.023164
CSRNP3	0.0121022	0	0.0121022
CSRP1	0.2546468	0	0.2546468
CSRP2	0.0524554	0	0.0524554
CSRP2BP	0.0125163	0.0125163	0
CSRP3	0.0136497	0	0
CST1	0.0005309	0	0.0005309
CST11	0.0067391	0	0
CST2	0.0009495	0	0.0009495
CST3	0.0281497	0	0
CST4	0.0010435	0	0
CST5	0.0015914	0	0.0015914
CST6	0.021984	0	0.021984
CST7	0.0008206	0	0.0008206
CST8	0.0044393	0.0044393	0
CST9	0.0043034	0.0043034	0
CST9L	0.0025917	0	0
CSTA	0.0693193	0	0.0693193
CSTB	0.0982353	0.0982353	0
CSTF1	0.1154652	0.1154652	0
CSTF2	0.0232564	0	0
CSTF2T	0.4626604	0	0.4626604
CSTF3	0.1638735	0.1638735	0
CSTL1	0.0051092	0.0051092	0
CSTT	0.0032108	0.0032108	0
CT45A1	0.0008374	0	0
CT45A2	1.54E-05	0	0
CT45A3	1.54E-05	0	0
CT45A5	2.51E-05	0	2.51E-05
CT45A6	0.0002226	0.0002226	0
CT47A7	1.53E-05	0	0
CT47B1	0.00013	0	0
CT62	0.013753	0.013753	0
CTAG2	0.0002302	0	0
CTAGE1	0.0183249	0.0183249	0
CTAGE5	0.1873019	0.1873019	0
CTAGE6	1.73E-05	1.73E-05	0
CTAGE9	9.19E-05	9.19E-05	0
CTBP1	0.0050642	0.0050642	0
CTBP2	0.0105074	0.0105074	0
CTBS	0.1169454	0	0.1169454
CTCF	0.0835775	0	0.0835775
CTCFL	0.0057134	0	0
CTDP1	0.0152808	0	0

CTDSP1	0.0209479	0.0209479	0
CTDSP2	0.0344652	0	0.0344652
CTDSPL	0.0134529	0	0
CTDSPL2	0.1250235	0	0.1250235
CTF1	0.0125084	0	0
CTGF	0.051396	0	0.051396
CTH	0.1053804	0	0
CTHRC1	0.016831	0	0.016831
CTLA4	0.0118325	0	0.0118325
CTNNA1	0.079975	0.079975	0
CTNNA2	0.0034768	0	0.0034768
CTNNA3	0.017339	0	0.017339
CTNNAL1	0.1064048	0	0
CTNNB1	0.0675635	0	0.0675635
CTNNBIP1	0.0106769	0.0106769	0
CTNNBL1	0.1315638	0.1315638	0
CTNND1	0.1005773	0.1005773	0
CTNND2	0.0116061	0	0
CTNS	0.0069993	0	0.0069993
CTPS	0.0257512	0	0.0257512
CTPS2	0.0197933	0.0197933	0
CTR9	0.1300979	0	0.1300979
CTRB1	0.0014014	0	0.0014014
CTRB2	0.0025048	0	0.0025048
CTRC	0.0062326	0	0.0062326
CTRL	0.0066762	0	0.0066762
CTSA	0.0299405	0	0
CTSB	0.3033929	0	0.3033929
CTSC	0.2059378	0	0.2059378
CTSD	0.006352	0	0.006352
CTSE	0.3925943	0.3925943	0
CTSF	0.0015124	0	0.0015124
CTSG	0.0033377	0	0.0033377
CTSH	0.0393037	0	0.0393037
CTSK	0.0362617	0	0.0362617
CTSL1	0.0110911	0	0.0110911
CTSL2	0.0157527	0.0157527	0
CTSL3	0.0098291	0	0
CTSO	0.0213586	0	0.0213586
CTSS	0.246794	0	0.246794
CTSW	0.0379094	0	0.0379094
CTSZ	0.0317588	0	0.0317588
CTTN	0.025911	0.025911	0
CTTNBP2	0.0088569	0	0.0088569
CTTNBP2NL	0.371274	0	0.371274

CTU1	0.0185377	0.0185377	0
CTU2	0.0027703	0.0027703	0
CTXN1	0.004172	0.004172	0
CTXN2	0.0137864	0	0.0137864
CTXN3	0.0233996	0	0
CUBN	0.0061402	0	0.0061402
CUEDC1	0.1001978	0.1001978	0
CUEDC2	0.0272731	0	0
CUL1	0.0545979	0	0.0545979
CUL2	0.0412158	0	0.0412158
CUL3	0.0467609	0	0
CUL4A	0.052785	0.052785	0
CUL4B	0.0449877	0	0.0449877
CUL5	0.0348777	0	0.0348777
CUL7	0.0188664	0.0188664	0
CUL9	0.0789551	0	0
CUTA	0.0982772	0.0982772	0
CUTC	0.0485274	0	0.0485274
CUX1	0.0559207	0.0559207	0
CUX2	0.0020896	0	0.0020896
CUZD1	0.0330551	0	0
CWC15	0.0599115	0.0599115	0
CWC22	0.0921008	0	0.0921008
CWC25	0.0264556	0	0
CWC27	0.1917516	0	0
CWF19L1	0.0574492	0	0
CWF19L2	0.0203649	0	0.0203649
CWH43	0.0058563	0	0
CX3CL1	0.018957	0	0.018957
CX3CR1	0.0160776	0	0.0160776
CXADR	0.0332552	0.0332552	0
CXADRP2	2.28E-05	0	2.28E-05
CXADRP3	5.88E-05	0	5.88E-05
CXCL1	0.0706981	0	0.0706981
CXCL10	0.0204104	0	0.0204104
CXCL11	0.0128419	0	0.0128419
CXCL12	0.0012609	0	0.0012609
CXCL13	0.0010784	0	0.0010784
CXCL14	0.0076676	0	0.0076676
CXCL16	0.012495	0	0
CXCL17	0.0798869	0	0
CXCL2	1	0	1
CXCL3	0.1175882	0	0.1175882
CXCL5	0.0525005	0	0.0525005
CXCL6	0.082723	0	0.082723

CXCL9	0.0220252	0	0.0220252
CXCR1	0.0059168	0	0.0059168
CXCR2	0.0118092	0	0.0118092
CXCR3	0.0140711	0	0.0140711
CXCR4	0.0720231	0	0.0720231
CXCR5	0.0213615	0	0.0213615
CXCR6	0.0096231	0	0.0096231
CXCR7	0.0358172	0	0.0358172
CXorf1	0.00398	0	0.00398
CXorf21	0.0463779	0	0.0463779
CXorf22	0.0048023	0.0048023	0
CXorf23	0.010918	0	0
CXorf26	0.0098928	0.0098928	0
CXorf27	0.0282081	0.0282081	0
CXorf30	0.002731	0.002731	0
CXorf36	0.0049548	0	0.0049548
CXorf38	0.0387504	0	0.0387504
CXorf40A	0.0015269	0	0
CXorf40B	0.001643	0	0
CXorf41	0.0118764	0	0
CXorf42	0.0367399	0	0
CXorf48	0.0051016	0	0
CXorf56	0.0078431	0	0
CXorf57	0.0116427	0	0.0116427
CXorf58	0.1040793	0	0
CXorf59	0.0047137	0	0
CXorf61	0.0283991	0	0
CXorf64	0.0035281	0	0.0035281
CXorf65	0.0180471	0	0.0180471
CXorf66	0.0026063	0	0
CXXC1	0.0522354	0	0
CXXC4	0.0478607	0	0
CXXC5	0.0265415	0.0265415	0
CYB561	0.0534223	0.0534223	0
CYB561D1	0.0192302	0	0.0192302
CYB561D2	0.0240531	0.0240531	0
CYB5A	0.0214329	0	0
CYB5B	0.093278	0.093278	0
CYB5D1	0.0732403	0	0
CYB5D2	0.0508414	0	0
CYB5R1	0.1110189	0	0
CYB5R2	0.0397822	0	0
CYB5R3	0.0155884	0	0.0155884
CYB5R4	0.179479	0	0.179479
CYB5RL	0.0148072	0	0.0148072

CYBA	0.0025293	0	0
CYBASC3	0.1288965	0	0.1288965
CYBB	0.0187076	0	0.0187076
CYBRD1	0.0365293	0	0.0365293
CYC1	0.021008	0.021008	0
CYCS	0.1579524	0.1579524	0
CYCSP52	0.0433444	0.0433444	0
CYFIP1	0.0167076	0	0.0167076
CYFIP2	0.0824029	0	0.0824029
CYGB	0.0102103	0	0.0102103
CYHR1	0.0045413	0.0045413	0
CYLC1	0.0042599	0	0
CYLC2	0.0083118	0.0083118	0
CYLD	0.0625742	0	0.0625742
CYMP	0.0083795	0	0
CYorf15A	1.53E-05	0	0
CYorf15B	1.53E-05	0	0
CYP11A1	0.0496934	0	0.0496934
CYP11B1	0.0012689	0	0
CYP11B2	0.0022657	0	0
CYP17A1	0.0054565	0	0
CYP19A1	0.0131539	0	0.0131539
CYP1A1	0.0160501	0	0.0160501
CYP1A2	0.004115	0	0.004115
CYP1B1	0.1040925	0	0.1040925
CYP20A1	0.0141755	0	0.0141755
CYP24A1	0.1316071	0	0.1316071
CYP26A1	0.0027735	0	0.0027735
CYP26B1	0.0033613	0	0.0033613
CYP26C1	0.0036352	0	0.0036352
CYP27A1	0.0041825	0	0.0041825
CYP27B1	0.0312791	0.0312791	0
CYP27C1	0.002887	0	0.002887
CYP2A13	0.0016357	0.0016357	0
CYP2A6	0.000496	0.000496	0
CYP2A7	0.0005627	0.0005627	0
CYP2B6	0.0052165	0	0
CYP2B7P1	0.006717	0	0
CYP2C18	0.0136809	0.0136809	0
CYP2C19	0.0022028	0	0
CYP2C8	0.0034227	0.0034227	0
CYP2C9	0.0047182	0	0
CYP2D6	0.0004518	0.0004518	0
CYP2D7P1	0.0005035	0.0005035	0
CYP2E1	0.0032835	0	0

CYP2F1	0.0010948	0	0
CYP2J2	0.0089544	0.0089544	0
CYP2R1	0.0280131	0.0280131	0
CYP2S1	0.0024601	0.0024601	0
CYP2U1	0.0106539	0	0.0106539
CYP2W1	0.0023801	0.0023801	0
CYP39A1	0.0837063	0	0.0837063
CYP3A4	0.0104752	0	0
CYP3A43	0.039479	0	0
CYP3A5	0.0092598	0.0092598	0
CYP3A7	0.2489898	0.2489898	0
CYP46A1	0.0063726	0	0.0063726
CYP4A11	0.0033383	0	0
CYP4A22	0.0020329	0	0
CYP4B1	0.0039668	0	0.0039668
CYP4F11	0.0095838	0.0095838	0
CYP4F12	0.0056769	0.0056769	0
CYP4F2	0.0043824	0	0
CYP4F22	0.0020956	0	0.0020956
CYP4F3	0.0093328	0.0093328	0
CYP4F8	0.0029086	0	0
CYP4V2	0.006087	0	0
CYP4X1	0.0235334	0	0.0235334
CYP4Z1	0.0014604	0	0
CYP4Z2P	0.0008075	0	0
CYP51A1	0.0432413	0.0432413	0
CYP7A1	0.007521	0	0.007521
CYP7B1	0.0340461	0	0.0340461
CYP8B1	0.0071513	0	0.0071513
CYR61	0.1352089	0	0.1352089
CYS1	0.0244333	0	0.0244333
CYSLTR1	0.0299599	0	0.0299599
CYSLTR2	0.0043013	0	0.0043013
CYTH1	0.0405986	0	0.0405986
CYTH2	0.1125776	0.1125776	0
CYTH3	0.0165819	0	0.0165819
CYTH4	0.0277198	0	0.0277198
CYTIP	0.1996283	0	0.1996283
CYTL1	0.0046542	0	0.0046542
CYTSA	0.0421912	0	0.0421912
CYTSB	0.04265	0	0.04265
CYYR1	0.0082341	0	0.0082341
D4S234E	0.0023263	0	0
DAAM1	0.0412035	0	0.0412035
DAAM2	0.0037436	0	0.0037436

DAB1	0.0036291	0	0.0036291
DAB2	0.1051917	0	0.1051917
DAB2IP	0.0299116	0	0
DACH1	0.0058048	0	0
DACH2	0.0079477	0	0.0079477
DACT1	0.0229931	0	0.0229931
DACT2	0.0090157	0.0090157	0
DACT3	0.0064539	0	0.0064539
DAD1	0.0695299	0.0695299	0
DAD1L	0.0122209	0	0
DAG1	0.0422829	0.0422829	0
DAGLA	0.0057168	0.0057168	0
DAGLB	0.1390495	0	0
DAK	0.0369975	0.0369975	0
DALRD3	0.0538141	0.0538141	0
DAND5	0.0041683	0.0041683	0
DAO	0.0097221	0	0.0097221
DAOA	0.0168476	0	0
DAP	0.0696757	0.0696757	0
DAP3	0.0567683	0.0567683	0
DAPK1	0.0422095	0	0
DAPK2	0.0181327	0	0
DAPK3	0.0216588	0.0216588	0
DAPL1	0.0101792	0	0.0101792
DAPP1	0.1826266	0	0.1826266
DARC	0.034781	0	0.034781
DARS	0.2057175	0	0
DARS2	0.1328595	0.1328595	0
DAZ1	1.53E-05	1.53E-05	0
DAZ2	1.53E-05	1.53E-05	0
DAZAP1	0.0059087	0.0059087	0
DAZAP2	0.041822	0	0.041822
DAZL	0.0290137	0	0.0290137
DBC1	0.0082322	0	0.0082322
DBF4	0.1243481	0	0
DBF4B	0.0314606	0.0314606	0
DBH	0.0012523	0	0.0012523
DBI	0.0248017	0	0
DBN1	0.002993	0	0
DBNDD1	0.0092203	0.0092203	0
DBNDD2	0.0252443	0.0252443	0
DBNL	0.0074438	0	0
DBP	0.0611883	0.0611883	0
DBR1	0.0263012	0	0.0263012
DBT	0.0359245	0	0.0359245

DBX1	0.0036906	0	0
DBX2	0.0117773	0	0.0117773
DCAF10	0.0354254	0	0
DCAF11	0.146364	0.146364	0
DCAF12	0.0336091	0	0
DCAF12L1	0.0019409	0	0
DCAF12L2	0.0018034	0	0.0018034
DCAF13	0.182891	0.182891	0
DCAF15	0.0073027	0.0073027	0
DCAF16	0.1093657	0.1093657	0
DCAF17	0.0549961	0	0.0549961
DCAF4	0.0151158	0.0151158	0
DCAF4L1	0.0124516	0.0124516	0
DCAF4L2	0.0049203	0.0049203	0
DCAF5	0.0592286	0	0
DCAF6	0.4840013	0	0
DCAF7	0.0251021	0	0.0251021
DCAF8	0.1627197	0.1627197	0
DCAF8L1	0.0045351	0.0045351	0
DCAF8L2	0.0036244	0.0036244	0
DCAKD	0.0789609	0	0
DCBLD1	0.0280421	0	0.0280421
DCBLD2	0.0302173	0	0.0302173
DCC	0.0074295	0	0.0074295
DCD	0.013237	0	0
DCDC1	0.0636556	0	0
DCDC2	0.0098061	0	0.0098061
DCDC2B	0.0151034	0.0151034	0
DCHS1	0.0069724	0	0.0069724
DCHS2	0.0093368	0	0.0093368
DCI	0.0088373	0.0088373	0
DCK	0.0301341	0	0.0301341
DCLK1	0.0037455	0	0.0037455
DCLK2	0.0284598	0	0.0284598
DCLK3	0.0150697	0	0.0150697
DCLRE1A	0.1539883	0.1539883	0
DCLRE1B	0.1019963	0	0.1019963
DCLRE1C	0.0591798	0	0
DCN	0.0328113	0	0.0328113
DCP1A	0.0523105	0	0.0523105
DCP1B	0.0420185	0.0420185	0
DCP2	0.1501403	0	0.1501403
DCPS	0.0241637	0	0
DCST1	0.0062871	0.0062871	0
DCST2	0.0061402	0.0061402	0

DCT	0.0066842	0	0
DCTD	0.0271274	0	0
DCTN1	0.011211	0	0
DCTN2	0.0317534	0.0317534	0
DCTN3	0.0153872	0	0
DCTN4	0.1842964	0	0
DCTN5	0.0329697	0	0
DCTN6	0.0643584	0	0.0643584
DCTPP1	0.0275046	0.0275046	0
DCUN1D1	0.0199979	0	0.0199979
DCUN1D2	0.049119	0.049119	0
DCUN1D3	0.6237352	0	0.6237352
DCUN1D4	0.0217579	0	0
DCUN1D5	0.0831337	0	0
DCX	0.0042953	0	0.0042953
DCXR	0.0029977	0.0029977	0
DDA1	0.0895449	0.0895449	0
DDAH1	0.1394626	0.1394626	0
DDB1	0.0411651	0	0
DDB2	0.7566892	0	0
DDC	0.0035878	0.0035878	0
DDHD1	0.0670396	0	0.0670396
DDHD2	0.0209617	0	0.0209617
DDI1	0.0080824	0	0
DDI2	0.0505181	0	0.0505181
DDIT3	0.0794488	0.0794488	0
DDIT4	0.315915	0.315915	0
DDIT4L	0.0048126	0	0.0048126
DDN	0.0221009	0	0
DDO	0.0086387	0	0
DDOST	0.0075361	0	0
DDR2	0.1281665	0	0.1281665
DDRGK1	0.0076476	0.0076476	0
DDT	8.36E-05	8.36E-05	0
DDTL	6.47E-05	6.47E-05	0
DDX1	0.0319353	0.0319353	0
DDX10	0.0379113	0	0
DDX11	0.0518891	0.0518891	0
DDX12	0.0083562	0.0083562	0
DDX17	0.0546141	0	0
DDX18	0.0288655	0	0.0288655
DDX19A	0.0270068	0	0.0270068
DDX19B	0.0739889	0	0
DDX20	0.0665837	0	0.0665837
DDX21	0.2567279	0	0.2567279

DDX23	0.5862333	0.5862333	0
DDX24	0.0586327	0	0.0586327
DDX25	0.0606432	0	0.0606432
DDX26B	0.011319	0	0.011319
DDX27	0.0339727	0.0339727	0
DDX28	0.0246085	0	0
DDX31	0.064364	0.064364	0
DDX39	0.0319158	0	0
DDX3X	0.3242108	0	0.3242108
DDX3Y	1.53E-05	0	0
DDX4	0.0251415	0	0
DDX41	0.0163234	0.0163234	0
DDX42	0.2543104	0	0
DDX43	0.0140002	0	0.0140002
DDX46	0.0785532	0	0
DDX47	0.3568653	0	0
DDX49	0.0274503	0.0274503	0
DDX5	0.582726	0	0.582726
DDX50	0.0470992	0	0.0470992
DDX51	0.0013354	0.0013354	0
DDX52	0.04404	0	0
DDX53	0.0079787	0	0
DDX54	0.1000657	0.1000657	0
DDX55	0.0281191	0.0281191	0
DDX56	0.0409883	0.0409883	0
DDX58	0.1516494	0	0.1516494
DDX59	0.6609722	0	0.6609722
DDX6	0.031277	0	0.031277
DDX60	0.0295305	0	0.0295305
DDX60L	0.1154178	0	0
DEAF1	0.0080165	0	0
1-Dec	0.020539	0	0
DECR1	0.0830966	0	0
DECR2	0.0133807	0.0133807	0
DEDD	0.2084414	0.2084414	0
DEDD2	0.0137714	0.0137714	0
DEF6	0.0259083	0	0.0259083
DEF8	0.0033581	0	0
DEFA4	0.0024728	0	0.0024728
DEFA5	0.0058468	0	0
DEFA6	0.005497	0	0.005497
DEFB1	0.0210827	0	0
DEFB108B	0.0028995	0	0
DEFB109P1	2.15E-05	0	0
DEFB110	0.0090843	0	0

DEFB112	0.0143747	0	0
DEFB113	0.0144393	0	0
DEFB114	0.0137216	0	0
DEFB115	0.0055442	0	0
DEFB116	0.0119632	0	0
DEFB118	0.0067627	0	0
DEFB119	0.0065359	0	0.0065359
DEFB121	0.0076951	0	0
DEFB122	0.0045681	0	0
DEFB123	0.006148	0	0
DEFB124	0.002627	0	0.002627
DEFB125	0.0041146	0	0.0041146
DEFB126	0.0042394	0.0042394	0
DEFB127	0.0044852	0	0
DEFB128	0.0092689	0	0
DEFB129	0.0090278	0	0
DEFB131	0.0010237	0	0
DEFB132	0.0071724	0	0.0071724
DEFB134	0.0096868	0	0
DEFB135	0.0050594	0	0
DEFB136	0.0035235	0	0
DEFB4A	2.64E-05	0	0
DEGS1	0.0904708	0	0.0904708
DEGS2	0.000983	0.000983	0
DEK	0.1519276	0	0.1519276
DEM1	0.0085304	0	0.0085304
DENND1A	0.7572863	0	0
DENND1B	0.1748843	0	0
DENND1C	0.0085902	0	0.0085902
DENND2A	0.0052205	0	0.0052205
DENND2C	0.0601606	0	0.0601606
DENND2D	0.1565345	0	0
DENND3	0.219926	0	0.219926
DENND4A	0.763589	0	0.763589
DENND4B	0.023387	0	0
DENND4C	0.0465344	0	0.0465344
DENND5A	0.0318973	0	0.0318973
DENND5B	0.0340764	0	0.0340764
DENR	0.0461139	0	0
DEPDC1	0.0483649	0.0483649	0
DEPDC1B	0.0409583	0.0409583	0
DEPDC4	0.0586523	0.0586523	0
DEPDC5	0.0448231	0	0.0448231
DEPDC6	0.0456855	0	0.0456855
DEPDC7	0.0895918	0	0.0895918

DERA	0.095299	0.095299	0
DERL1	0.183183	0	0
DERL2	0.0581456	0	0.0581456
DERL3	0.0068414	0	0.0068414
DES	0.0011433	0	0.0011433
DET1	0.0026708	0	0
DEXI	0.082373	0	0
DFFA	0.0599904	0	0.0599904
DFFB	0.0067093	0	0
DFNA5	0.0558982	0	0.0558982
DFNB31	0.0300161	0.0300161	0
DFNB59	0.0885788	0	0
DGAT2	0.0898343	0	0
DGAT2L6	0.0114135	0	0.0114135
DGCR10	0.0006433	0.0006433	0
DGCR11	0.0020636	0	0
DGCR14	0.0077871	0.0077871	0
DGCR2	0.0045255	0.0045255	0
DGCR5	0.0003191	0	0
DGCR6	0.0027721	0	0
DGCR6L	0.0013902	0.0013902	0
DGCR8	0.0109787	0.0109787	0
DGCR9	0.0020223	0	0
DGKA	0.3272323	0	0
DGKB	0.0286239	0	0.0286239
DGKD	0.027974	0	0.027974
DGKE	0.0419227	0	0
DGKG	0.0065178	0	0.0065178
DGKH	0.0597245	0	0
DGKI	0.0157042	0	0.0157042
DGKK	0.0032871	0	0
DGKQ	0.0003281	0.0003281	0
DGKZ	0.0175484	0.0175484	0
DGUOK	0.3939232	0.3939232	0
DHCR24	0.1341057	0.1341057	0
DHCR7	0.025519	0.025519	0
DHDDS	0.0119555	0	0.0119555
DHDH	0.0066967	0.0066967	0
DHDPSL	0.0099774	0	0
DHFR	0.0418311	0	0
DHFRL1	0.1538	0	0.1538
DHH	0.0134745	0	0.0134745
DHODH	0.0360276	0.0360276	0
DHPS	0.0345948	0.0345948	0
DHRS1	0.0484648	0.0484648	0

DHRS11	0.0158444	0.0158444	0
DHRS12	0.0357219	0.0357219	0
DHRS13	0.5768423	0.5768423	0
DHRS2	0.0078004	0	0
DHRS3	0.0252507	0.0252507	0
DHRS4	0.0040405	0.0040405	0
DHRS4L1	0.0101656	0	0
DHRS4L2	0.0017236	0.0017236	0
DHRS7	0.0829623	0.0829623	0
DHRS7B	0.0764112	0	0
DHRS7C	0.0279516	0	0
DHRS9	0.0228997	0	0
DHTKD1	0.0490553	0.0490553	0
DHX15	0.0600096	0	0
DHX29	0.1088983	0	0.1088983
DHX30	0.0496987	0.0496987	0
DHX32	0.4226159	0.4226159	0
DHX33	0.0510906	0	0.0510906
DHX34	0.0110527	0.0110527	0
DHX35	0.0705399	0	0
DHX36	0.1037908	0	0.1037908
DHX37	0.0396889	0.0396889	0
DHX38	0.1139134	0	0
DHX40	0.0397041	0	0.0397041
DHX40P1	1.81E-05	0	0
DHX57	0.0245398	0	0.0245398
DHX58	0.0064635	0	0.0064635
DHX8	0.2782553	0	0
DHX9	0.124002	0	0
DIABLO	0.0106944	0.0106944	0
DIAPH1	0.1305928	0.1305928	0
DIAPH2	0.0307385	0	0
DIAPH3	0.1230481	0.1230481	0
DICER1	0.5226627	0	0.5226627
DIDO1	0.0860455	0.0860455	0
DIMT1L	0.0600651	0	0.0600651
DIO1	0.1140406	0	0.1140406
DIO2	0.076942	0	0.076942
DIO3	0.0012287	0	0.0012287
DIO3OS	0.0010119	0	0.0010119
DIP2A	0.0176054	0.0176054	0
DIP2B	0.0405268	0	0
DIP2C	0.0015212	0	0.0015212
DIRAS1	0.0005688	0	0.0005688
DIRAS2	0.0066629	0	0

DIRAS3	0.0049842	0	0.0049842
DIRC1	0.0775966	0	0.0775966
DIRC2	0.7091	0	0
DIRC3	0.0028614	0	0.0028614
DIS3	0.1627153	0	0.1627153
DIS3L	0.0669466	0	0.0669466
DIS3L2	0.0213193	0.0213193	0
DISC1	0.049769	0	0.049769
DISC2	0.421284	0	0
DISP1	0.0684036	0	0
DISP2	0.0025488	0.0025488	0
DIXDC1	0.0584041	0	0.0584041
DKC1	0.0158501	0.0158501	0
DKFZP434H16	0.0099367	0	0
DKFZp434J02	0.0699029	0	0
DKFZP434K02	0.0049015	0.0049015	0
DKFZP434L18	1.56E-05	0	1.56E-05
DKFZp434L19	0.0017852	0	0.0017852
DKFZp566F09	0.0020762	0	0
DKFZP586I142	0.0303647	0	0
DKFZp686A16	0.0008764	0.0008764	0
DKFZP686I152	0.7914096	0.7914096	0
DKFZp686O24	0.0264989	0	0
DKFZp761E19	0.0068806	0	0.0068806
DKFZp779M0	0.0171209	0	0.0171209
DKK1	0.072621	0	0
DKK2	0.0072114	0	0.0072114
DKK3	0.0259292	0	0.0259292
DKK4	0.0219059	0.0219059	0
DKKL1	0.0227393	0	0.0227393
DLAT	0.1091416	0	0.1091416
DLC1	0.0132826	0	0.0132826
DLD	0.0444915	0	0
DLEC1	0.0121132	0	0
DLEU1	0.15029	0.15029	0
DLEU2	0.036395	0.036395	0
DLEU2L	0.0231225	0	0
DLEU7	0.0031022	0	0.0031022
DLG1	0.186798	0	0
DLG2	0.2149516	0	0.2149516
DLG3	0.0103995	0.0103995	0
DLG4	0.0394631	0	0.0394631
DLG5	0.0077618	0.0077618	0
DLGAP1	0.0335615	0	0
DLGAP2	0.0014587	0	0.0014587

DLGAP3	0.004134	0	0.004134
DLGAP4	0.0776143	0.0776143	0
DLGAP5	0.0498861	0.0498861	0
DLK1	0.0005071	0	0.0005071
DLK2	0.0049196	0	0
DLL1	0.0013779	0	0.0013779
DLL3	0.0037428	0	0.0037428
DLL4	0.0085624	0	0.0085624
DLST	0.0325677	0.0325677	0
DLX1	0.0074894	0	0.0074894
DLX2	0.0173682	0	0
DLX3	0.0060958	0	0
DLX4	0.0044316	0.0044316	0
DLX5	0.0028674	0	0.0028674
DLX6	0.0017977	0	0
DLX6AS	0.0021145	0	0
DMAP1	0.0344503	0.0344503	0
DMBT1	0.0164798	0	0
DMBX1	0.0041056	0	0
DMC1	0.0104343	0	0.0104343
DMD	0.0853666	0	0.0853666
DMGDH	0.0068275	0	0.0068275
DMKN	0.048426	0.048426	0
DMP1	0.0237546	0	0
DMPK	0.0037427	0.0037427	0
DMRT1	0.002461	0	0
DMRT2	0.0040775	0	0.0040775
DMRT3	0.0021913	0	0.0021913
DMRTA1	0.0083838	0	0
DMRTA2	0.0014473	0	0
DMRTB1	0.0042094	0	0
DMRTC2	0.0024118	0	0.0024118
DMTF1	0.2280861	0.2280861	0
DMWD	0.0085912	0.0085912	0
DMXL1	0.1722769	0	0.1722769
DMXL2	0.0737759	0	0.0737759
DNA2	0.0146012	0.0146012	0
DNAH1	0.003876	0	0
DNAH10	0.0110604	0	0.0110604
DNAH11	0.0097575	0	0
DNAH12	0.0099384	0	0
DNAH14	0.1704215	0.1704215	0
DNAH17	0.003529	0	0
DNAH2	0.0045693	0.0045693	0
DNAH3	0.0220013	0.0220013	0

DNAH5	0.0250323	0.0250323	0
DNAH6	0.0071057	0.0071057	0
DNAH7	0.0461098	0	0
DNAH8	0.0104093	0	0.0104093
DNAH9	0.0031701	0	0.0031701
DNAI1	0.0289043	0	0
DNAI2	0.0091441	0	0.0091441
DNAJA1	0.1619032	0	0.1619032
DNAJA2	0.0644542	0	0
DNAJA3	0.0372006	0.0372006	0
DNAJA4	0.0341696	0.0341696	0
DNAJB1	0.2278802	0.2278802	0
DNAJB11	0.1033113	0.1033113	0
DNAJB12	0.0500772	0.0500772	0
DNAJB13	0.0108746	0.0108746	0
DNAJB14	0.1199956	0	0.1199956
DNAJB2	0.0654389	0.0654389	0
DNAJB3	0.0024705	0.0024705	0
DNAJB4	0.2885602	0	0.2885602
DNAJB5	0.045908	0	0.045908
DNAJB6	0.0310065	0	0.0310065
DNAJB7	0.0323584	0	0
DNAJB8	0.0012807	0	0
DNAJB9	0.3762404	0	0.3762404
DNAJC1	0.062433	0.062433	0
DNAJC10	0.0422752	0	0
DNAJC11	0.0126049	0.0126049	0
DNAJC12	0.0275694	0	0
DNAJC13	0.0475063	0	0.0475063
DNAJC14	0.1530802	0	0
DNAJC15	0.0288667	0	0
DNAJC16	0.1826143	0	0.1826143
DNAJC17	0.0249526	0.0249526	0
DNAJC18	0.0934511	0	0.0934511
DNAJC19	0.0562613	0.0562613	0
DNAJC2	0.2148476	0.2148476	0
DNAJC21	0.1717018	0	0
DNAJC22	0.0101448	0.0101448	0
DNAJC24	0.0579084	0	0.0579084
DNAJC25	0.0951853	0	0
DNAJC25-GN(	0.0945536	0	0
DNAJC27	0.1349345	0	0.1349345
DNAJC28	0.0140446	0	0
DNAJC3	0.1361707	0	0
DNAJC30	0.011095	0.011095	0

DNAJC4	0.0194035	0.0194035	0
DNAJC5	0.0386378	0.0386378	0
DNAJC5B	0.0473478	0	0.0473478
DNAJC5G	0.0121503	0	0.0121503
DNAJC6	0.0154766	0	0.0154766
DNAJC7	0.0525995	0.0525995	0
DNAJC8	0.0457297	0	0.0457297
DNAJC9	0.1844449	0.1844449	0
DNAL1	0.0255255	0	0.0255255
DNAL4	0.0170592	0.0170592	0
DNALI1	0.0157525	0.0157525	0
DNASE1	0.0042648	0.0042648	0
DNASE1L1	0.0062949	0.0062949	0
DNASE1L2	0.0009276	0.0009276	0
DNASE1L3	0.4975743	0	0.4975743
DNASE2	0.0312631	0	0.0312631
DNASE2B	0.0413919	0	0.0413919
DND1	0.0198084	0.0198084	0
DNER	0.0102621	0	0
DNHD1	0.0206391	0	0
DNLZ	0.0039687	0.0039687	0
DNM1	0.0060864	0	0.0060864
DNM1L	0.0949968	0	0
DNM1P35	0.0054968	0.0054968	0
DNM2	0.0387854	0.0387854	0
DNM3	0.0396106	0	0.0396106
DNMBP	0.1181012	0.1181012	0
DNMT1	0.0259601	0	0.0259601
DNMT3A	0.0038429	0	0
DNMT3B	0.0382652	0.0382652	0
DNMT3L	0.0012731	0	0
DNPEP	0.0039616	0.0039616	0
DNTT	0.0121302	0	0.0121302
DNTTIP1	0.0187001	0.0187001	0
DNTTIP2	0.0898728	0	0.0898728
DOC2A	0.0013974	0.0013974	0
DOC2B	0.0090318	0	0.0090318
DOCK1	0.004247	0	0.004247
DOCK10	0.0416632	0	0.0416632
DOCK11	0.003572	0	0.003572
DOCK2	0.0557727	0	0.0557727
DOCK3	0.010153	0	0
DOCK4	0.1597883	0	0.1597883
DOCK5	0.3362961	0	0
DOCK6	0.0029422	0.0029422	0

DOCK7	0.0647235	0	0.0647235
DOCK8	0.0095312	0	0.0095312
DOCK9	0.0155353	0	0.0155353
DOHH	0.0261179	0.0261179	0
DOK1	0.0240083	0	0.0240083
DOK2	0.0273833	0	0.0273833
DOK3	0.018772	0	0.018772
DOK4	0.0049363	0.0049363	0
DOK5	0.0051103	0	0.0051103
DOK6	0.0051212	0	0.0051212
DOK7	0.0011723	0.0011723	0
DOLK	0.0217374	0.0217374	0
DOLPP1	0.013085	0.013085	0
DONSON	0.0385503	0	0
DOPEY1	0.1141763	0	0.1141763
DOPEY2	0.0076036	0.0076036	0
DOT1L	0.0961745	0.0961745	0
DPAGT1	0.3069148	0.3069148	0
DPCD	0.0119845	0.0119845	0
DPEP1	0.0007601	0	0.0007601
DPEP2	0.0068278	0	0.0068278
DPEP3	0.0065979	0	0.0065979
DPF1	0.0031276	0	0
DPF2	0.0235814	0	0.0235814
DPF3	0.0254515	0	0.0254515
DPH1	0.0060244	0	0
DPH2	0.0232524	0	0
DPH3	0.076352	0	0.076352
DPH3B	0.0077097	0	0.0077097
DPH5	0.1386232	0	0.1386232
DPM1	0.0682722	0	0
DPM2	0.0283431	0.0283431	0
DPM3	0.2227598	0.2227598	0
DPP10	0.0046909	0	0
DPP3	0.0491506	0.0491506	0
DPP4	0.0184022	0	0.0184022
DPP6	0.0022589	0	0.0022589
DPP7	0.0046332	0.0046332	0
DPP8	0.059207	0	0.059207
DPP9	0.1061064	0.1061064	0
DPPA2	0.0013989	0	0
DPPA3	0.002041	0	0
DPPA4	0.0031091	0	0.0031091
DPPA5	0.0019933	0.0019933	0
DPRX	0.0006607	0	0

DPRXP4	0.0259457	0	0.0259457
DPT	0.0401175	0	0.0401175
DPY19L1	0.0189285	0	0
DPY19L2	0.0015452	0	0
DPY19L2P1	0.0014597	0	0
DPY19L2P2	0.0017402	0	0
DPY19L2P4	0.0002887	0	0
DPY19L3	0.0349698	0	0.0349698
DPY19L4	0.0428477	0	0
DPY30	0.0258158	0.0258158	0
DPYD	0.1005836	0	0.1005836
DPYS	0.0143036	0	0.0143036
DPYSL2	0.178594	0	0.178594
DPYSL3	0.0130245	0	0.0130245
DPYSL4	0.0005034	0	0.0005034
DPYSL5	0.0042626	0	0.0042626
DQX1	0.0674546	0.0674546	0
DR1	0.2474161	0	0.2474161
DRAM1	0.0402707	0	0.0402707
DRAM2	0.1416634	0	0.1416634
DRAP1	0.0276363	0	0
DRD1	0.0082109	0	0
DRD2	0.0032055	0	0
DRD3	0.0101603	0	0.0101603
DRD4	0.0014823	0.0014823	0
DRD5	0.0021332	0	0.0021332
DRG1	0.0138453	0	0
DRG2	0.0087239	0	0
DRGX	0.0047967	0	0.0047967
DRP2	0.0053298	0	0.0053298
DSC1	0.009863	0	0.009863
DSC2	0.0240219	0	0
DSC3	0.0779926	0	0.0779926
DSCAM	0.0076421	0	0.0076421
DSCAML1	0.0020001	0	0
DSCC1	0.0461313	0	0
DSCR10	0.0059788	0.0059788	0
DSCR3	0.1080777	0	0.1080777
DSCR4	0.0136213	0	0.0136213
DSCR6	0.0046031	0.0046031	0
DSCR8	0.0142119	0	0.0142119
DSCR9	0.0176543	0	0
DSE	0.2135501	0	0.2135501
DSEL	0.0513238	0	0.0513238
DSG1	0.0351857	0	0

DSG2	0.0849405	0.0849405	0
DSG3	0.4863317	0	0
DSG4	0.0107846	0	0
DSN1	0.0267593	0.0267593	0
DSP	0.0688925	0.0688925	0
DSPP	0.0076098	0	0
DST	0.1996461	0	0.1996461
DSTN	0.0303235	0.0303235	0
DSTYK	0.0974495	0	0.0974495
DTD1	0.0141129	0.0141129	0
DTHD1	0.0800829	0	0.0800829
DTL	1	1	0
DTNA	0.2107458	0	0.2107458
DTNB	0.0491587	0.0491587	0
DTNBP1	0.0238126	0	0.0238126
DTWD1	0.4437208	0	0.4437208
DTWD2	0.0613347	0	0.0613347
DTX1	0.0109078	0	0.0109078
DTX2	0.0001052	0.0001052	0
DTX3	0.004309	0	0
DTX3L	0.7176282	0	0
DTX4	0.1938096	0.1938096	0
DULLARD	0.0706799	0	0.0706799
DUOX1	0.0049029	0	0
DUOX2	0.0027162	0	0
DUOXA1	0.0049235	0	0
DUOXA2	0.0028297	0	0
DUPD1	0.005833	0	0.005833
DUS1L	0.0031035	0.0031035	0
DUS2L	0.0269328	0	0
DUS3L	0.0152244	0.0152244	0
DUS4L	0.1536873	0.1536873	0
DUSP1	0.8358839	0	0.8358839
DUSP10	0.3618026	0	0
DUSP11	0.0244473	0	0.0244473
DUSP12	0.3712958	0.3712958	0
DUSP13	0.0290031	0	0
DUSP14	0.0949348	0	0.0949348
DUSP15	0.0142937	0	0
DUSP16	0.0986135	0	0
DUSP18	0.0061935	0	0
DUSP19	0.0828943	0	0.0828943
DUSP2	0.0549832	0	0.0549832
DUSP21	0.0059101	0	0
DUSP22	1	0	1

DUSP23	0.3905467	0.3905467	0
DUSP26	0.0061225	0	0.0061225
DUSP27	0.058271	0	0.058271
DUSP28	0.0301389	0.0301389	0
DUSP3	0.0129148	0	0.0129148
DUSP4	0.1041499	0.1041499	0
DUSP5	0.5052358	0	0.5052358
DUSP5P	0.0028623	0	0
DUSP6	0.1151287	0.1151287	0
DUSP7	0.049227	0	0.049227
DUSP8	0.000683	0.000683	0
DUSP9	0.0005558	0.0005558	0
DUT	0.1596299	0	0
DUXA	0.0015368	0	0
DVL1	0.0009679	0.0009679	0
DVL2	0.0649867	0	0.0649867
DVL3	0.0150923	0	0.0150923
DVWA	0.0538383	0.0538383	0
DYDC1	0.0035542	0	0
DYDC2	0.0036041	0	0.0036041
DYM	0.0435763	0	0.0435763
DYNC1H1	0.0443966	0	0.0443966
DYNC1I1	0.0082367	0	0.0082367
DYNC1I2	0.0461945	0	0.0461945
DYNC1LI1	0.0340243	0	0.0340243
DYNC1LI2	0.0251446	0	0
DYNC2H1	0.0318641	0	0
DYNC2LI1	0.0319913	0.0319913	0
DYNLL1	0.0258635	0.0258635	0
DYNLL2	0.0096158	0	0
DYNLRB1	0.1393805	0.1393805	0
DYNLRB2	0.0234006	0	0
DYNLT1	0.1505754	0	0
DYNLT3	0.0396415	0	0
DYRK1A	0.2057687	0	0.2057687
DYRK1B	0.0200538	0.0200538	0
DYRK2	0.0416921	0	0.0416921
DYRK3	0.4783746	0	0.4783746
DYRK4	0.0040802	0	0
DYSF	0.0579064	0	0.0579064
DYSFIP1	0.0024997	0	0
DYTN	0.0084264	0	0
DYX1C1	0.0065818	0	0
DZIP1	0.0144796	0	0.0144796
DZIP1L	0.0453784	0	0.0453784

DZIP3	0.1037056	0	0.1037056
E2F1	0.0089726	0.0089726	0
E2F2	0.0423707	0	0
E2F3	0.1607964	0	0.1607964
E2F4	0.0114214	0.0114214	0
E2F5	0.0331812	0.0331812	0
E2F6	0.0329654	0	0
E2F7	0.1495479	0.1495479	0
E2F8	0.283634	0.283634	0
E4F1	0.0118386	0.0118386	0
EAF1	0.0352668	0	0
EAF2	0.0793185	0	0.0793185
EAPP	0.0067328	0.0067328	0
EARS2	0.0329273	0.0329273	0
EBAG9	0.1105343	0.1105343	0
EBF1	0.0791635	0	0.0791635
EBF2	0.0014807	0	0.0014807
EBF3	0.000615	0	0.000615
EBF4	0.0004207	0	0
EBI3	0.0193106	0	0.0193106
EBNA1BP2	0.0192998	0.0192998	0
EBP	0.012146	0.012146	0
EBPL	0.0780308	0.0780308	0
ECD	0.0638965	0	0
ECE1	0.0383026	0	0
ECE2	0.0452242	0.0452242	0
ECEL1	0.0002005	0	0.0002005
ECH1	0.0384655	0.0384655	0
ECHDC1	0.1181003	0	0.1181003
ECHDC2	0.0170097	0.0170097	0
ECHDC3	0.0014193	0	0.0014193
ECHS1	0.0023435	0.0023435	0
ECM1	0.0194728	0	0
ECM2	0.0131573	0	0.0131573
ECSCR	0.0332992	0	0.0332992
ECSIT	0.0329987	0.0329987	0
ECT2	0.2623124	0.2623124	0
ECT2L	0.0183244	0	0
EDA	0.0087891	0	0.0087891
EDA2R	0.0074153	0	0.0074153
EDAR	0.0051672	0	0
EDARADD	0.0138823	0	0.0138823
EDC3	0.0362183	0	0
EDC4	0.0215417	0.0215417	0
EDDM3A	0.006367	0	0.006367

EDDM3B	0.0114557	0	0
EDEM1	0.4529017	0	0.4529017
EDEM2	0.513247	0	0
EDEM3	0.1363846	0	0
EDF1	0.004763	0.004763	0
EDIL3	0.1029515	0	0.1029515
EDN1	0.1798591	0	0.1798591
EDN2	0.0248429	0.0248429	0
EDN3	0.0028052	0	0
EDNRA	0.0351258	0	0.0351258
EDNRB	0.0158158	0	0.0158158
EEA1	0.0855424	0	0.0855424
EED	0.2529384	0	0
EEF1A1	0.3778601	0	0.3778601
EEF1A1P9	0.005256	0	0.005256
EEF1A2	0.0011205	0.0011205	0
EEF1B2	0.0851322	0	0
EEF1D	0.0232958	0.0232958	0
EEF1DP3	0.0069734	0.0069734	0
EEF1E1	0.0516949	0	0
EEF1G	0.0520214	0	0
EEF2	0.0549443	0	0
EEF2K	0.134195	0	0
EEFSEC	0.0132763	0.0132763	0
EEPD1	0.0265823	0.0265823	0
EFCAB1	0.0131472	0	0.0131472
EFCAB10	0.0113938	0.0113938	0
EFCAB2	0.3815957	0	0
EFCAB3	0.0050608	0	0
EFCAB4A	0.0047655	0.0047655	0
EFCAB4B	0.0084008	0	0.0084008
EFCAB5	0.1009356	0	0
EFCAB6	0.0180473	0	0
EFCAB7	0.0836609	0	0
EFEMP1	0.0378288	0	0.0378288
EFEMP2	0.0215396	0	0.0215396
EFHA1	0.0238183	0	0
EFHA2	0.0056409	0	0.0056409
EFHB	0.0133625	0	0.0133625
EFHC1	0.1080919	0.1080919	0
EFHC2	0.0060684	0	0
EFHD1	0.0060353	0	0.0060353
EFHD2	0.0725987	0.0725987	0
EFNA1	0.7380529	0.7380529	0
EFNA2	0.0008804	0.0008804	0

EFNA3	0.0208934	0.0208934	0
EFNA4	0.019253	0.019253	0
EFNA5	0.1136779	0.1136779	0
EFNB1	0.0044056	0.0044056	0
EFNB2	0.0289756	0.0289756	0
EFNB3	0.0013906	0	0.0013906
EFR3A	0.091015	0	0.091015
EFR3B	0.0063282	0	0.0063282
EFS	0.0017088	0	0.0017088
EFTUD1	0.0668526	0	0
EFTUD2	0.1424143	0	0
EGF	0.0096966	0	0.0096966
EGFL6	0.0083077	0	0.0083077
EGFL7	0.0007495	0	0.0007495
EGFLAM	0.0404934	0	0.0404934
EGFR	0.0207163	0	0
EGLN1	0.1426775	0	0
EGLN2	0.0181363	0	0
EGLN3	0.0611619	0.0611619	0
EGOT	0.6725122	0	0
EGR1	0.0750714	0	0.0750714
EGR2	0.0772927	0	0.0772927
EGR3	0.0303834	0	0.0303834
EGR4	0.0190244	0.0190244	0
EHBP1	0.2152548	0	0
EHBP1L1	0.0976053	0	0
EHD1	0.1413952	0	0
EHD2	0.0049671	0	0.0049671
EHD3	0.0124843	0	0.0124843
EHD4	0.1869768	0	0.1869768
EHF	0.0263747	0.0263747	0
EHHADH	0.0176968	0.0176968	0
EHMT1	0.038328	0.038328	0
EI24	0.0276414	0.0276414	0
EID1	0.2426243	0	0.2426243
EID2	0.0712174	0	0.0712174
EID2B	0.04638	0	0.04638
EID3	0.0584685	0	0.0584685
EIF1	1	1	0
EIF1AD	0.0608691	0	0
EIF1AX	0.0453435	0	0
EIF1AY	1.53E-05	0	0
EIF1B	0.2003688	0	0.2003688
EIF2A	0.2082289	0.2082289	0
EIF2AK1	0.0098259	0.0098259	0

EIF2AK2	0.0314771	0	0.0314771
EIF2AK3	0.1786036	0	0.1786036
EIF2AK4	0.0970105	0	0.0970105
EIF2B1	0.0193218	0	0
EIF2B2	0.0421114	0.0421114	0
EIF2B3	0.0327893	0	0.0327893
EIF2B4	0.0886709	0.0886709	0
EIF2B5	0.0969472	0.0969472	0
EIF2C1	0.0106704	0	0.0106704
EIF2C2	0.0361211	0	0
EIF2C3	0.0565631	0	0.0565631
EIF2C4	0.0100691	0	0.0100691
EIF2S1	0.1830648	0	0.1830648
EIF2S2	0.1382188	0.1382188	0
EIF2S3	0.1301438	0	0
EIF3A	0.065846	0	0
EIF3B	0.0539991	0.0539991	0
EIF3D	0.0274269	0	0
EIF3E	0.2875388	0	0
EIF3F	0.2204642	0.2204642	0
EIF3G	0.022529	0.022529	0
EIF3H	0.1591371	0.1591371	0
EIF3I	0.0385178	0	0
EIF3IP1	0.0084024	0	0
EIF3J	0.079552	0	0
EIF3K	0.039472	0.039472	0
EIF3L	0.0344697	0	0.0344697
EIF3M	0.0620734	0.0620734	0
EIF4A1	0.0815466	0	0.0815466
EIF4A2	0.315824	0.315824	0
EIF4A3	0.0564208	0	0
EIF4B	0.0941901	0	0.0941901
EIF4E	0.1736838	0	0
EIF4E1B	0.0031428	0	0
EIF4E2	0.0178833	0	0
EIF4E3	0.0078678	0	0.0078678
EIF4EBP1	0.0142813	0.0142813	0
EIF4EBP2	0.0239516	0	0.0239516
EIF4EBP3	0.111113	0.111113	0
EIF4ENIF1	0.0688374	0	0
EIF4G1	0.0295945	0	0
EIF4G2	0.1460852	0	0.1460852
EIF4G3	0.0335148	0	0.0335148
EIF4H	0.0531598	0.0531598	0
EIF5	0.1764202	0.1764202	0

EIF5A	0.0522555	0.0522555	0
EIF5A2	0.0966787	0	0.0966787
EIF5AL1	0.0001939	0	0
EIF5B	0.0928039	0	0.0928039
EIF6	0.0193177	0.0193177	0
ELAC1	0.0300409	0	0.0300409
ELAC2	0.0942376	0	0
ELANE	0.0065658	0	0.0065658
ELAVL1	0.0119238	0.0119238	0
ELAVL2	0.0082252	0	0.0082252
ELAVL3	0.0020397	0	0.0020397
ELAVL4	0.0093711	0	0.0093711
ELF1	0.1370348	0	0.1370348
ELF2	0.0524723	0	0.0524723
ELF3	0.2033578	0.2033578	0
ELF4	0.0152569	0	0
ELF5	0.0141168	0	0
ELFN1	0.0012746	0	0.0012746
ELFN2	0.0165171	0	0
ELK1	0.0407564	0	0.0407564
ELK3	0.2640501	0	0.2640501
ELK4	0.266463	0	0.266463
ELL	0.0759623	0	0.0759623
ELL2	0.1709488	0	0.1709488
ELL3	0.0843622	0.0843622	0
ELMO1	0.0139144	0	0.0139144
ELMO2	0.0751585	0	0.0751585
ELMO3	0.0090226	0.0090226	0
ELMOD1	0.0074876	0	0.0074876
ELMOD2	0.0176607	0	0.0176607
ELMOD3	0.0387781	0.0387781	0
ELN	0.0062553	0	0.0062553
ELOF1	0.0273003	0.0273003	0
ELOVL1	0.0615624	0.0615624	0
ELOVL2	0.0090384	0	0.0090384
ELOVL3	0.0182558	0	0.0182558
ELOVL4	0.0106616	0	0.0106616
ELOVL5	0.1316041	0	0.1316041
ELOVL6	0.0430284	0.0430284	0
ELOVL7	0.0148157	0	0
ELP2	0.0876749	0	0.0876749
ELP2P	0.0115014	0	0
ELP3	0.1390981	0	0.1390981
ELP4	0.0715109	0	0.0715109
ELSPBP1	0.0015833	0	0

ELTD1	0.0120432	0	0.0120432
EMB	0.0371311	0	0.0371311
EMCN	0.0094426	0	0.0094426
EMD	0.0033278	0.0033278	0
EME1	0.0296807	0.0296807	0
EME2	0.0067772	0.0067772	0
EMG1	0.1613187	0.1613187	0
EMID1	0.0230748	0	0.0230748
EMID2	0.001502	0	0.001502
EMILIN1	0.0178091	0	0.0178091
EMILIN2	0.0050504	0	0.0050504
EMILIN3	0.0007937	0	0.0007937
EML1	0.0097055	0	0.0097055
EML2	0.1299599	0.1299599	0
EML3	0.1621426	0	0.1621426
EML4	0.0484372	0	0
EML5	0.0103164	0	0
EML6	0.0370794	0	0.0370794
EMP1	0.3445901	0	0.3445901
EMP2	0.0124992	0.0124992	0
EMP3	0.0949304	0	0.0949304
EMR1	0.008501	0	0.008501
EMR2	0.0744151	0	0.0744151
EMR3	0.0126211	0	0.0126211
EMR4P	0.0128698	0	0.0128698
EMX1	0.0080187	0.0080187	0
EMX2	0.0021198	0	0.0021198
EMX2OS	0.001872	0	0.001872
EN1	0.0008267	0	0.0008267
EN2	0.000429	0	0.000429
ENAH	0.5564536	0	0.5564536
ENAM	0.0074325	0	0
ENC1	0.374588	0	0.374588
ENDOD1	0.1589527	0	0
ENDOG	0.0174208	0.0174208	0
ENDOU	0.0304789	0	0.0304789
ENG	0.0100538	0	0.0100538
ENGASE	0.00951	0.00951	0
ENHO	0.0011114	0	0.0011114
ENKUR	0.0330416	0.0330416	0
ENO1	0.4165578	0	0
ENO2	0.0133282	0.0133282	0
ENO3	0.045885	0	0
ENOPH1	0.0802988	0.0802988	0
ENOSF1	0.0226205	0.0226205	0

ENOX1	0.0164178	0	0.0164178
ENOX2	0.0212271	0	0.0212271
ENPEP	0.0111425	0	0.0111425
ENPP1	0.0065877	0	0.0065877
ENPP2	0.1204162	0	0.1204162
ENPP3	0.0192506	0	0.0192506
ENPP4	0.0076454	0	0
ENPP5	0.019891	0	0
ENPP6	0.0010837	0	0.0010837
ENPP7	0.0021195	0	0
ENSA	0.1850904	0.1850904	0
ENTHD1	0.0269292	0	0.0269292
ENTPD1	0.1081624	0	0.1081624
ENTPD2	0.0049837	0.0049837	0
ENTPD3	0.0080577	0	0
ENTPD4	0.0772957	0	0.0772957
ENTPD5	0.0159201	0	0
ENTPD6	0.0122418	0.0122418	0
ENTPD7	0.1725201	0	0.1725201
ENTPD8	0.0022785	0.0022785	0
ENY2	0.2493601	0	0
EOMES	0.0223679	0	0.0223679
EP300	0.0274518	0	0.0274518
EP400	0.0111991	0	0
EP400NL	0.0106662	0	0
EPAS1	0.9221569	0	0.9221569
EPB41	0.045601	0	0.045601
EPB41L1	0.0120291	0.0120291	0
EPB41L2	0.0534936	0	0.0534936
EPB41L3	0.0031262	0	0.0031262
EPB41L4A	0.0325979	0	0
EPB41L4B	0.0560731	0.0560731	0
EPB41L5	0.0194781	0	0
EPB42	0.0213775	0	0.0213775
EPB49	0.0102622	0.0102622	0
EPC1	0.0787174	0	0.0787174
EPC2	0.0577893	0	0.0577893
EPCAM	0.0070119	0.0070119	0
EPDR1	0.0228302	0	0.0228302
EPGN	0.2600406	0	0
EPHA1	0.007847	0.007847	0
EPHA10	0.0126686	0.0126686	0
EPHA2	0.2691412	0.2691412	0
EPHA3	0.0088214	0	0.0088214
EPHA4	0.0059912	0	0

EPHA5	0.0082664	0	0.0082664
EPHA6	0.0089034	0	0.0089034
EPHA7	0.0080968	0	0
EPHA8	0.0006549	0	0.0006549
EPHB1	0.0138722	0	0.0138722
EPHB2	0.0027867	0	0.0027867
EPHB3	0.0028682	0.0028682	0
EPHB4	0.0017765	0.0017765	0
EPHB6	0.0120273	0	0
EPHX1	0.0337654	0	0
EPHX2	0.0307971	0	0
EPHX3	0.0024262	0	0.0024262
EPHX4	0.0058167	0.0058167	0
EPM2A	0.0497753	0	0.0497753
EPM2AIP1	0.1319302	0	0
EPN1	0.009815	0.009815	0
EPN2	0.0007015	0.0007015	0
EPN3	0.0071589	0.0071589	0
EPO	0.0030511	0	0
EPOR	0.0237515	0.0237515	0
EPPK1	0.0078935	0.0078935	0
EPR1	0.002494	0.002494	0
EPRS	0.0696107	0	0
EPS15	0.1102052	0	0.1102052
EPS15L1	0.0094973	0	0
EPS8	0.1180231	0	0
EPS8L1	0.0074924	0.0074924	0
EPS8L2	0.0033143	0.0033143	0
EPS8L3	0.0089194	0.0089194	0
EPSTI1	0.0861487	0	0.0861487
EPT1	0.1712728	0.1712728	0
EPX	0.0065584	0	0
EPYC	0.0326065	0	0.0326065
ERAL1	0.0159286	0.0159286	0
ERAP1	0.0496184	0	0.0496184
ERAP2	0.1582759	0	0.1582759
ERAS	0.0013969	0.0013969	0
ERBB2	0.0168073	0.0168073	0
ERBB2IP	0.1112386	0	0.1112386
ERBB3	0.0069317	0.0069317	0
ERBB4	0.0064576	0	0.0064576
ERC1	0.0612565	0	0.0612565
ERC2	0.0090964	0	0.0090964
ERCC1	0.0405758	0	0
ERCC2	0.0089822	0.0089822	0

ERCC3	0.0191874	0	0
ERCC4	0.0454871	0	0.0454871
ERCC5	0.0913377	0.0913377	0
ERCC6	0.0488651	0	0.0488651
ERCC6L	0.0070955	0.0070955	0
ERCC8	0.0502043	0	0.0502043
EREG	0.1005021	0	0
ERF	0.005877	0.005877	0
ERG	0.0251512	0	0.0251512
ERGIC1	0.0692005	0.0692005	0
ERGIC2	0.5911614	0	0.5911614
ERGIC3	0.0151356	0.0151356	0
ERH	0.1406575	0.1406575	0
ERI1	0.9702619	0	0.9702619
ERI2	0.0504424	0.0504424	0
ERI3	0.0157657	0.0157657	0
ERICH1	0.0046139	0	0.0046139
ERLEC1	0.0588865	0	0
ERLIN1	0.12135	0	0
ERLIN2	0.034112	0	0
ERMAP	0.0364947	0	0.0364947
ERMN	0.0223593	0	0.0223593
ERMP1	0.096092	0.096092	0
ERN1	0.0701363	0	0.0701363
ERN2	0.0021705	0.0021705	0
ERO1L	0.3594184	0.3594184	0
ERO1LB	0.1146013	0	0.1146013
ERP27	0.0214882	0	0.0214882
ERP29	0.1223328	0.1223328	0
ERP44	0.0879138	0.0879138	0
ERRFI1	0.2095164	0	0
ERVFRDE1	0.020303	0	0.020303
ESAM	0.0107138	0	0.0107138
ESCO1	0.030972	0	0
ESCO2	0.1094779	0	0
ESD	0.050323	0	0
ESF1	0.0661782	0	0.0661782
ESM1	0.0664474	0	0
ESPL1	0.0201649	0.0201649	0
ESPN	0.0093696	0.0093696	0
ESPNL	0.0015355	0	0.0015355
ESPNP	0.0616498	0.0616498	0
ESR1	0.0138278	0	0.0138278
ESR2	0.0515865	0	0.0515865
ESRP1	0.0114233	0.0114233	0

ESRP2	0.0307539	0.0307539	0
ESRRA	0.0230736	0.0230736	0
ESRRB	0.0033097	0	0.0033097
ESRRG	0.0257352	0	0
ESX1	0.0014519	0	0
ESYT1	0.1872945	0	0.1872945
ESYT2	0.0548338	0	0
ESYT3	0.0046302	0.0046302	0
ETAA1	0.1210975	0	0
ETF1	0.1124996	0	0.1124996
ETFA	0.1264031	0.1264031	0
ETFB	0.0204071	0.0204071	0
ETFDH	0.0372464	0	0
ETHE1	0.155936	0.155936	0
ETNK1	0.0735194	0.0735194	0
ETNK2	0.0085186	0	0
ETS1	0.1120325	0	0.1120325
ETS2	0.5989716	0	0.5989716
ETV1	0.1247772	0	0.1247772
ETV2	0.0096333	0.0096333	0
ETV3	0.1365346	0	0.1365346
ETV3L	0.0070113	0	0.0070113
ETV4	0.0143508	0.0143508	0
ETV5	0.0567939	0	0.0567939
ETV6	1	0	1
ETV7	0.0190565	0	0
EVC	0.0098283	0	0.0098283
EVC2	0.007623	0	0.007623
EVI2A	0.1194978	0	0.1194978
EVI2B	0.2336371	0	0.2336371
EVI5	0.0220524	0	0.0220524
EVI5L	0.025209	0.025209	0
EVL	0.1197368	0	0.1197368
EVPL	0.0018794	0.0018794	0
EVPLL	0.0051733	0.0051733	0
EVX1	0.0013039	0.0013039	0
EVX2	0.0018112	0	0
EWSR1	0.033464	0.033464	0
EXD1	0.0482296	0	0
EXD2	0.0060427	0	0
EXD3	0.0146173	0.0146173	0
EXO1	0.1465181	0.1465181	0
EXOC1	0.0153386	0	0
EXOC2	0.0207511	0	0.0207511
EXOC3	0.0233427	0.0233427	0

EXOC3L	0.0089456	0	0
EXOC3L2	0.0014095	0	0.0014095
EXOC4	0.0448248	0.0448248	0
EXOC5	0.0780266	0	0.0780266
EXOC6	0.0657814	0	0
EXOC6B	0.0743036	0	0.0743036
EXOC7	0.0098291	0	0
EXOC8	0.1068291	0	0
EXOG	0.0296446	0	0.0296446
EXOSC1	0.036239	0.036239	0
EXOSC10	0.0080588	0	0
EXOSC2	0.0787392	0.0787392	0
EXOSC3	0.0332632	0.0332632	0
EXOSC4	0.3677176	0.3677176	0
EXOSC5	0.036859	0.036859	0
EXOSC6	0.0104708	0	0
EXOSC7	0.0537362	0.0537362	0
EXOSC8	0.069855	0.069855	0
EXOSC9	0.0479962	0	0
EXPH5	0.064869	0.064869	0
EXT1	0.5215337	0	0.5215337
EXT2	0.0148496	0	0.0148496
EXTL1	0.0025735	0	0.0025735
EXTL2	0.1876476	0	0.1876476
EXTL3	0.0440098	0	0.0440098
EYA1	0.0320046	0	0.0320046
EYA2	0.0309136	0	0.0309136
EYA3	0.07362	0	0.07362
EYA4	0.2460654	0	0.2460654
EYS	0.0058817	0	0
EZH1	0.013832	0	0
EZH2	0.0592589	0.0592589	0
EZR	0.1066788	0.1066788	0
F10	0.0011412	0	0.0011412
F11	0.0038033	0	0
F11R	0.1558809	0.1558809	0
F12	0.0218511	0.0218511	0
F13A1	0.0135608	0	0.0135608
F13B	0.0261701	0.0261701	0
F2	0.0046395	0.0046395	0
F2R	0.3993231	0	0.3993231
F2RL1	0.1062764	0.1062764	0
F2RL2	0.026132	0	0.026132
F2RL3	0.0079772	0	0.0079772
F3	0.1406004	0	0

F5	0.2320306	0.2320306	0
F7	0.0005049	0	0.0005049
F8	0.0204057	0	0.0204057
F9	0.0015848	0.0015848	0
FA2H	0.0071325	0.0071325	0
FAAH	0.0030205	0.0030205	0
FAAH2	0.998115	0.998115	0
FABP1	0.0045437	0.0045437	0
FABP12	0.0312053	0	0
FABP2	0.0066365	0	0
FABP3	0.0058386	0	0.0058386
FABP4	0.015438	0	0.015438
FABP5	0.0794593	0	0.0794593
FABP5L3	0.0183515	0	0.0183515
FABP6	0.0179201	0.0179201	0
FABP7	0.0132102	0	0
FABP9	0.0121934	0	0.0121934
FADD	0.0345131	0.0345131	0
FADS1	0.0699307	0	0.0699307
FADS2	0.0573901	0	0.0573901
FADS3	0.0141916	0	0.0141916
FADS6	0.0012661	0	0
FAF1	0.0838867	0.0838867	0
FAF2	0.0489906	0	0
FAH	0.0256981	0	0
FAHD1	0.0099329	0.0099329	0
FAHD2A	0.00671	0	0
FAHD2B	0.0008658	0	0
FAIM	0.0199206	0	0
FAIM2	0.0042395	0	0.0042395
FAIM3	0.3008759	0	0.3008759
FAM100A	0.0339128	0.0339128	0
FAM100B	0.1987224	0.1987224	0
FAM101A	0.0030682	0.0030682	0
FAM101B	0.0005045	0	0.0005045
FAM102A	0.0469705	0	0
FAM102B	0.2931753	0	0.2931753
FAM103A1	0.0432546	0.0432546	0
FAM104A	0.0771843	0.0771843	0
FAM104B	0.035459	0	0
FAM105A	0.050932	0	0.050932
FAM105B	0.2504901	0	0.2504901
FAM106A	2.31E-05	0	0
FAM107A	0.006839	0	0.006839
FAM107B	0.0093642	0	0

FAM108A1	0.0022119	0.0022119	0
FAM108B1	0.1048057	0	0.1048057
FAM108C1	0.055766	0.055766	0
FAM109A	0.0039052	0.0039052	0
FAM109B	0.010935	0	0.010935
FAM10A4	0.0039459	0	0.0039459
FAM110A	0.0167407	0.0167407	0
FAM110B	0.052181	0	0.052181
FAM110C	0.0266269	0.0266269	0
FAM111A	0.2897769	0	0
FAM111B	0.1044728	0	0
FAM113A	0.0187716	0.0187716	0
FAM113B	0.1695638	0	0.1695638
FAM114A1	0.0322078	0	0.0322078
FAM114A2	0.0853759	0	0.0853759
FAM115A	0.0378655	0	0.0378655
FAM115C	1.68E-05	0	1.68E-05
FAM116A	0.0797373	0	0.0797373
FAM116B	0.0029511	0.0029511	0
FAM117A	0.0142659	0	0.0142659
FAM117B	0.0337155	0	0
FAM118A	0.0129882	0	0
FAM118B	0.0193134	0	0.0193134
FAM119A	0.0612372	0	0
FAM119B	0.0368192	0	0
FAM120A	0.0527106	0.0527106	0
FAM120AOS	0.0726163	0	0
FAM120B	0.0115895	0	0.0115895
FAM120C	0.0587088	0	0.0587088
FAM122A	0.0366595	0	0.0366595
FAM122B	0.0139586	0.0139586	0
FAM122C	0.0127993	0	0
FAM123A	0.0110485	0	0
FAM123B	0.0090704	0	0.0090704
FAM123C	0.0011425	0	0.0011425
FAM124A	0.0055294	0	0.0055294
FAM124B	0.0035073	0	0.0035073
FAM125A	0.034537	0.034537	0
FAM125B	0.005359	0	0.005359
FAM126A	0.1205906	0	0.1205906
FAM126B	0.0623951	0	0
FAM127A	0.0135301	0	0.0135301
FAM127B	0.0077033	0.0077033	0
FAM127C	0.0133959	0	0.0133959
FAM128A	0.0164327	0.0164327	0

FAM128B	0.0047253	0.0047253	0
FAM129A	0.1418367	0	0.1418367
FAM129B	1	1	0
FAM129C	0.0154428	0	0.0154428
FAM131A	0.0173774	0.0173774	0
FAM131B	0.0054664	0	0.0054664
FAM131C	0.0134526	0	0
FAM132A	0.0007552	0.0007552	0
FAM133A	0.0061151	0	0.0061151
FAM133B	0.046868	0	0
FAM134A	0.015589	0.015589	0
FAM134B	0.0595229	0	0
FAM134C	0.0347648	0.0347648	0
FAM135A	0.0543494	0.0543494	0
FAM135B	0.0028977	0	0.0028977
FAM136A	0.0387651	0.0387651	0
FAM136B	0.0272435	0	0
FAM138B	0.0003012	0	0.0003012
FAM138D	0.0001253	0	0
FAM138E	3.53E-05	0	0
FAM13A	0.0867594	0	0
FAM13AOS	0.0157125	0	0
FAM13B	0.2020343	0	0.2020343
FAM13C	0.0066976	0	0.0066976
FAM149A	0.0053932	0	0
FAM149B1	0.0666618	0	0
FAM150A	0.0014448	0.0014448	0
FAM150B	0.0044395	0	0.0044395
FAM151A	0.0027333	0	0
FAM151B	0.055777	0	0.055777
FAM153A	1.58E-05	0	0
FAM153B	2.46E-05	2.46E-05	0
FAM153C	0.0002758	0	0
FAM154A	0.0111623	0	0
FAM154B	0.068191	0	0
FAM155A	0.0043561	0	0.0043561
FAM155B	0.0018195	0.0018195	0
FAM157A	5.68E-05	5.68E-05	0
FAM157B	3.94E-05	0	0
FAM158A	0.0613488	0.0613488	0
FAM159A	0.0102879	0	0.0102879
FAM160A1	0.1452789	0.1452789	0
FAM160A2	0.0639435	0.0639435	0
FAM160B1	0.1275952	0	0.1275952
FAM160B2	0.0149396	0.0149396	0

FAM161A	0.0473239	0	0
FAM161B	0.018021	0	0
FAM162A	0.1199371	0.1199371	0
FAM162B	0.0034956	0	0.0034956
FAM163A	0.0101217	0	0.0101217
FAM163B	0.0010528	0	0
FAM164A	0.0497822	0	0.0497822
FAM164C	0.1980204	0.1980204	0
FAM165B	0.0352582	0	0
FAM166A	0.00866	0.00866	0
FAM166B	0.0059756	0	0
FAM167A	0.0190376	0	0
FAM167B	0.1040592	0	0.1040592
FAM168A	0.0644014	0	0.0644014
FAM168B	0.0114651	0	0.0114651
FAM169A	0.0062586	0	0
FAM169B	0.0077412	0	0
FAM170A	0.0105536	0	0
FAM170B	0.0103248	0	0.0103248
FAM171A1	0.0049683	0	0.0049683
FAM171A2	0.0018102	0	0.0018102
FAM171B	0.0206447	0	0.0206447
FAM172A	0.0726248	0	0.0726248
FAM173A	0.0052042	0.0052042	0
FAM173B	0.2246587	0	0
FAM174A	0.0743534	0.0743534	0
FAM174B	0.0482968	0.0482968	0
FAM175A	0.0153142	0	0.0153142
FAM175B	0.0121301	0	0.0121301
FAM176A	0.012381	0	0.012381
FAM176B	0.0029078	0	0
FAM177A1	0.1231607	0.1231607	0
FAM177B	0.0829711	0	0
FAM178A	0.1231036	0	0.1231036
FAM178B	0.0074897	0	0.0074897
FAM179A	0.0109777	0	0.0109777
FAM179B	0.1839719	0	0.1839719
FAM180A	0.0105046	0	0.0105046
FAM180B	0.0306067	0	0.0306067
FAM181A	0.0027865	0	0
FAM181B	0.0077035	0	0
FAM182A	1.53E-05	0	0
FAM182B	1.53E-05	0	0
FAM183A	0.0158016	0	0
FAM183B	0.0108813	0	0

FAM184A	0.0228615	0	0.0228615
FAM184B	0.0065927	0	0
FAM185A	0.0169183	0.0169183	0
FAM186A	0.6711623	0.6711623	0
FAM186B	0.0061789	0	0
FAM187B	0.0064349	0	0.0064349
FAM188A	0.0651635	0	0
FAM188B	0.0199404	0.0199404	0
FAM189A1	0.0016489	0	0.0016489
FAM189A2	0.013221	0.013221	0
FAM189B	0.0533145	0.0533145	0
FAM18A	0.0228164	0	0.0228164
FAM18B	0.0017767	0.0017767	0
FAM18B2	0.0005539	0.0005539	0
FAM190A	0.0273783	0.0273783	0
FAM190B	0.0374138	0	0.0374138
FAM192A	0.1043248	0.1043248	0
FAM193A	0.003099	0	0.003099
FAM193B	0.1064375	0.1064375	0
FAM194A	0.0232499	0	0
FAM194B	0.0029565	0	0
FAM195A	0.0055501	0.0055501	0
FAM195B	0.0034658	0	0
FAM196A	0.0024648	0	0.0024648
FAM196B	0.0686484	0	0.0686484
FAM198A	0.020705	0	0.020705
FAM198B	0.0122622	0	0.0122622
FAM199X	0.0194635	0.0194635	0
FAM19A1	0.0055745	0	0.0055745
FAM19A2	0.0232924	0	0.0232924
FAM19A3	0.019749	0	0.019749
FAM19A4	0.0037271	0	0.0037271
FAM19A5	0.0006521	0	0.0006521
FAM200A	0.0333482	0	0
FAM200B	0.2601132	0	0
FAM20A	0.0075674	0	0.0075674
FAM20B	0.1444806	0	0
FAM20C	0.0006535	0	0.0006535
FAM21A	0.0008697	0	0.0008697
FAM21C	0.000719	0	0.000719
FAM22A	3.24E-05	0	0
FAM22D	3.51E-05	0	0
FAM22F	4.67E-05	4.67E-05	0
FAM22G	6.58E-05	6.58E-05	0
FAM24A	0.0031104	0	0

FAM24B	0.0274181	0.0274181	0
FAM25A	0.0006871	0.0006871	0
FAM26D	0.0153394	0	0
FAM26E	0.0097971	0	0.0097971
FAM26F	0.0118839	0	0.0118839
FAM27A	2.85E-05	2.85E-05	0
FAM27B	1.53E-05	0	0
FAM27C	1.53E-05	1.53E-05	0
FAM27L	0.0009434	0	0
FAM32A	0.0353441	0.0353441	0
FAM35A	0.0857694	0	0.0857694
FAM35B	1.53E-05	0	1.53E-05
FAM35B2	3.38E-05	0	3.38E-05
FAM36A	0.3836664	0.3836664	0
FAM38A	0.0082464	0	0
FAM38B	0.0220535	0	0.0220535
FAM3A	0.0019156	0.0019156	0
FAM3B	0.0029393	0.0029393	0
FAM3C	0.0926622	0	0
FAM3D	0.0017163	0.0017163	0
FAM40A	0.0797168	0	0.0797168
FAM40B	0.3382595	0.3382595	0
FAM41C	0.0240836	0.0240836	0
FAM43A	0.0494205	0	0.0494205
FAM43B	0.0012337	0	0.0012337
FAM45A	0.0236153	0	0
FAM45B	0.0240124	0	0.0240124
FAM46A	0.1530614	0	0.1530614
FAM46B	0.0187038	0	0.0187038
FAM46C	0.1073817	0	0.1073817
FAM46D	0.0041349	0	0.0041349
FAM47A	0.0080795	0	0
FAM47B	0.0033344	0	0
FAM47C	0.0024191	0	0
FAM47E	0.007205	0.007205	0
FAM48A	0.0102664	0.0102664	0
FAM48B1	0.0023149	0	0.0023149
FAM48B2	0.0024783	0	0.0024783
FAM49A	0.0224055	0	0.0224055
FAM49B	0.3487776	0	0.3487776
FAM50A	0.0007325	0.0007325	0
FAM50B	0.0741242	0	0
FAM53A	0.0053857	0.0053857	0
FAM53B	0.0402363	0	0.0402363
FAM53C	0.0874888	0	0.0874888

FAM54A	0.0724604	0.0724604	0
FAM54B	0.0164494	0	0
FAM55A	0.062691	0	0
FAM55B	0.0079291	0	0
FAM55C	0.0216415	0	0.0216415
FAM55D	0.0189084	0	0.0189084
FAM57A	0.0165634	0.0165634	0
FAM57B	0.0023663	0	0
FAM58A	0.0023042	0.0023042	0
FAM58B	0.0773669	0	0
FAM59A	0.0162502	0	0
FAM5B	0.0093354	0	0
FAM5C	0.0167887	0	0.0167887
FAM60A	0.1424413	0.1424413	0
FAM63A	0.0765929	0.0765929	0
FAM63B	0.0868951	0	0.0868951
FAM64A	0.0087165	0.0087165	0
FAM65A	0.0123964	0	0.0123964
FAM65B	0.1307578	0	0.1307578
FAM65C	0.0164143	0	0.0164143
FAM66A	1.75E-05	0	0
FAM66C	0.0002475	0	0
FAM66D	1.79E-05	1.79E-05	0
FAM66E	1.54E-05	0	0
FAM69A	0.0884073	0	0.0884073
FAM69B	0.0100782	0	0.0100782
FAM69C	0.0018158	0	0.0018158
FAM70A	0.0147391	0	0.0147391
FAM70B	0.0037985	0	0.0037985
FAM71A	0.0545621	0	0
FAM71B	0.0153089	0	0.0153089
FAM71C	0.0051314	0	0
FAM71D	0.0643668	0.0643668	0
FAM71E1	0.0045948	0.0045948	0
FAM71E2	0.0033633	0	0
FAM71F1	0.0076098	0	0
FAM71F2	0.0064129	0.0064129	0
FAM72A	8.51E-05	8.51E-05	0
FAM72B	0.0001286	0.0001286	0
FAM72D	0.0001439	0.0001439	0
FAM73A	0.0322619	0	0.0322619
FAM73B	0.0129586	0.0129586	0
FAM74A1	1.53E-05	0	0
FAM74A3	1.98E-05	0	1.98E-05
FAM74A4	1.53E-05	0	0

FAM75A3	1.54E-05	0	0
FAM75A6	9.29E-05	0	0
FAM75C1	8.01E-05	0	8.01E-05
FAM76A	0.0277681	0	0.0277681
FAM76B	0.1720664	0	0.1720664
FAM78A	0.0152645	0	0.0152645
FAM78B	0.0829025	0	0.0829025
FAM7A3	1.66E-05	0	1.66E-05
FAM81A	0.0246775	0	0
FAM81B	0.012942	0.012942	0
FAM82A1	0.1110109	0	0.1110109
FAM82A2	0.069472	0	0
FAM82B	0.0664757	0	0
FAM83A	0.4788975	0	0
FAM83B	0.4144729	0.4144729	0
FAM83C	0.0062934	0	0
FAM83D	0.029198	0.029198	0
FAM83E	0.0685144	0.0685144	0
FAM83F	0.0214892	0.0214892	0
FAM83G	0.0139437	0.0139437	0
FAM83H	0.0027791	0.0027791	0
FAM84A	0.0171194	0.0171194	0
FAM84B	0.0362153	0.0362153	0
FAM86A	0.0017854	0.0017854	0
FAM86B1	1.98E-05	1.98E-05	0
FAM86B2	1.65E-05	1.65E-05	0
FAM86C	0.015204	0.015204	0
FAM86D	0.0031482	0.0031482	0
FAM89A	0.0592612	0	0.0592612
FAM89B	0.1037813	0	0
FAM8A1	0.0407487	0	0.0407487
FAM90A1	0.0008355	0	0
FAM91A1	0.1994164	0	0
FAM92A1	0.0128331	0	0.0128331
FAM92A3	0.0017251	0	0.0017251
FAM92B	0.003489	0	0.003489
FAM96A	0.3054758	0	0
FAM96B	0.0263929	0.0263929	0
FAM98A	0.0430738	0	0.0430738
FAM98B	0.0705053	0	0.0705053
FAM98C	0.0032724	0.0032724	0
FAM99A	0.0004337	0.0004337	0
FAM99B	0.0006751	0	0
FAM9A	0.0122891	0	0
FAM9B	0.0081199	0	0

FAM9C	0.0410297	0	0.0410297
FANCA	0.0072715	0.0072715	0
FANCB	0.0277296	0	0.0277296
FANCC	0.0476725	0.0476725	0
FANCD2	0.0287939	0.0287939	0
FANCE	0.0205598	0.0205598	0
FANCF	0.0486159	0.0486159	0
FANCG	0.1328667	0.1328667	0
FANCI	0.0717532	0.0717532	0
FANCL	0.0935585	0.0935585	0
FANCM	0.1866448	0	0.1866448
FANK1	1	1	0
FAP	0.3360067	0	0.3360067
FAR1	0.0728054	0.0728054	0
FAR2	0.007701	0	0
FARP1	0.0098809	0	0.0098809
FARP2	0.0282894	0.0282894	0
FARS2	0.0556309	0	0
FARSA	0.0379221	0.0379221	0
FARSB	0.0629549	0.0629549	0
FAS	0.4613608	0	0.4613608
FASLG	0.0410344	0	0.0410344
FASN	0.0025481	0.0025481	0
FASTK	0.0067904	0.0067904	0
FASTKD1	0.0289314	0.0289314	0
FASTKD2	0.0523027	0	0
FASTKD3	0.4778454	0	0
FASTKD5	0.0128337	0	0.0128337
FAT1	0.0365349	0	0
FAT2	0.2626157	0	0.2626157
FAT3	0.0066386	0	0.0066386
FAT4	0.0289181	0	0.0289181
FATE1	0.0012869	0	0
FAU	0.071888	0.071888	0
FBF1	0.0469655	0	0
FBL	0.0208322	0.0208322	0
FBLIM1	0.0069639	0	0.0069639
FBLL1	0.0066746	0	0
FBLN1	0.0024687	0	0.0024687
FBLN2	0.0501832	0	0.0501832
FBLN5	0.0063638	0	0.0063638
FBLN7	0.001807	0	0.001807
FBN1	0.0364145	0	0.0364145
FBN2	0.0271821	0	0.0271821
FBN3	0.0008532	0	0.0008532

FBP1	0.0039012	0	0
FBP2	0.0041591	0	0
FBRS	0.0152594	0.0152594	0
FBRSL1	0.001107	0.001107	0
FBXL12	0.0067913	0.0067913	0
FBXL13	0.1110258	0.1110258	0
FBXL14	0.0328576	0.0328576	0
FBXL15	0.0071798	0.0071798	0
FBXL16	0.0025599	0	0.0025599
FBXL17	0.0516163	0	0.0516163
FBXL18	0.023342	0.023342	0
FBXL19	0.0201501	0.0201501	0
FBXL2	0.0023875	0	0
FBXL20	0.054538	0	0
FBXL21	0.0159742	0	0.0159742
FBXL22	0.0844613	0	0.0844613
FBXL3	0.054908	0	0.054908
FBXL4	0.0469135	0	0.0469135
FBXL5	0.2054764	0	0.2054764
FBXL6	0.0043806	0.0043806	0
FBXL7	0.009965	0	0.009965
FBXL8	0.0254092	0.0254092	0
FBXO10	0.0353615	0	0.0353615
FBXO11	0.1028898	0	0.1028898
FBXO15	0.0441701	0	0
FBXO16	0.0579393	0.0579393	0
FBXO17	0.0198868	0	0.0198868
FBXO18	0.0208615	0	0.0208615
FBXO2	0.0027945	0	0
FBXO21	0.0118043	0	0
FBXO22	0.0509212	0.0509212	0
FBXO22OS	0.0624026	0.0624026	0
FBXO24	0.0140085	0.0140085	0
FBXO25	0.0084539	0	0
FBXO27	0.0105496	0	0.0105496
FBXO28	0.1158873	0	0
FBXO3	0.0423597	0	0
FBXO30	0.0693442	0	0.0693442
FBXO31	0.2756712	0	0.2756712
FBXO32	0.0912314	0	0.0912314
FBXO33	0.1240173	0	0.1240173
FBXO34	0.0853026	0.0853026	0
FBXO36	0.0299728	0	0.0299728
FBXO38	0.1930506	0	0.1930506
FBXO39	0.0084589	0	0.0084589

FBXO4	0.1245934	0	0
FBXO40	0.0077104	0	0.0077104
FBXO41	0.0048425	0.0048425	0
FBXO42	0.0345054	0	0.0345054
FBXO43	0.1593711	0.1593711	0
FBXO44	0.0029445	0.0029445	0
FBXO45	0.1042683	0	0
FBXO46	0.092108	0.092108	0
FBXO47	0.0127547	0	0
FBXO48	0.0920652	0	0.0920652
FBXO5	0.2067021	0	0.2067021
FBXO6	0.0030825	0	0.0030825
FBXO7	0.0412134	0	0
FBXO8	0.0906861	0	0
FBXO9	0.6732824	0	0
FBXW10	0.0001913	0.0001913	0
FBXW11	0.1322262	0	0
FBXW12	0.006984	0	0
FBXW2	0.1680672	0	0.1680672
FBXW4	0.044464	0.044464	0
FBXW5	0.0102165	0.0102165	0
FBXW7	0.1261552	0	0.1261552
FBXW8	0.0271892	0	0
FBXW9	0.0084386	0.0084386	0
FCAMR	0.0683221	0	0.0683221
FCAR	0.0160617	0	0.0160617
FCER1A	0.0404526	0	0.0404526
FCER1G	0.0305939	0	0.0305939
FCER2	0.0483688	0	0.0483688
FCF1	0.0406317	0	0.0406317
FCGBP	0.0098157	0	0
FCGR1A	0.0001901	0	0.0001901
FCGR1B	2.13E-05	0	2.13E-05
FCGR1C	8.97E-05	0	8.97E-05
FCGR2A	0.3826367	0	0.3826367
FCGR2B	3.34E-05	0	3.34E-05
FCGR2C	3.43E-05	0	3.43E-05
FCGR3A	0.0154341	0	0.0154341
FCGR3B	0.002394	0	0.002394
FCGRT	0.003063	0	0.003063
FCHO1	0.1525569	0	0
FCHO2	0.0798616	0	0.0798616
FCHSD1	0.0148961	0.0148961	0
FCHSD2	0.0890309	0	0.0890309
FCN1	0.0042125	0	0.0042125

FCN2	0.005094	0	0.005094
FCN3	0.0216297	0	0.0216297
FCRL1	0.0250833	0	0.0250833
FCRL2	0.0205029	0	0.0205029
FCRL3	0.0222774	0	0.0222774
FCRL4	0.0466232	0	0.0466232
FCRL5	0.0309298	0	0.0309298
FCRL6	0.0283708	0	0.0283708
FCRLA	0.0924926	0	0.0924926
FCRLB	0.1046048	0	0
FDFT1	0.0384703	0.0384703	0
FDPS	0.1152435	0.1152435	0
FDPSL2A	0.0001923	0.0001923	0
FDX1	0.0616406	0.0616406	0
FDX1L	0.002835	0.002835	0
FDXACB1	0.0699559	0	0.0699559
FDXR	0.0487134	0.0487134	0
FECH	0.0151726	0	0.0151726
FEM1A	0.0234828	0	0
FEM1B	0.3232672	0	0.3232672
FEM1C	0.1297747	0	0
FEN1	0.0796935	0.0796935	0
FER	0.1823564	0	0.1823564
FER1L4	0.0344374	0.0344374	0
FER1L5	0.0142698	0	0
FER1L6	0.0439475	0	0
FERD3L	0.005505	0	0
FERMT1	0.020261	0.020261	0
FERMT2	0.1436666	0	0.1436666
FERMT3	0.0147257	0	0.0147257
FES	0.0027964	0	0.0027964
FETUB	0.0556957	0.0556957	0
FEV	0.0007977	0	0.0007977
FEZ1	0.0119291	0	0.0119291
FEZ2	0.0078795	0	0.0078795
FEZF1	0.0036996	0.0036996	0
FEZF2	0.0007818	0.0007818	0
FFAR1	0.0245509	0	0.0245509
FFAR2	0.0424182	0	0.0424182
FFAR3	0.0093593	0	0.0093593
FGA	0.006091	0	0
FGB	0.0261179	0	0
FGD1	0.0021796	0	0.0021796
FGD2	0.0082141	0	0.0082141
FGD3	0.0159795	0	0.0159795

FGD4	0.0175904	0.0175904	0
FGD5	0.0024151	0	0.0024151
FGD6	0.1475101	0	0
FGF1	0.0502222	0	0.0502222
FGF10	0.0253475	0	0.0253475
FGF11	0.0281808	0	0.0281808
FGF12	0.0149371	0	0.0149371
FGF13	0.0125459	0	0.0125459
FGF14	0.0114757	0	0.0114757
FGF16	0.0024694	0	0.0024694
FGF17	0.0064616	0.0064616	0
FGF18	0.0119453	0	0.0119453
FGF19	0.0032342	0	0.0032342
FGF2	0.0859479	0	0.0859479
FGF20	0.0054884	0	0
FGF21	0.0018797	0.0018797	0
FGF22	0.0022172	0.0022172	0
FGF23	0.003164	0.003164	0
FGF3	0.0005087	0	0
FGF4	0.0010364	0.0010364	0
FGF5	0.0133603	0	0.0133603
FGF6	0.0040178	0	0
FGF7	0.104565	0	0.104565
FGF8	0.0042149	0.0042149	0
FGF9	0.0112729	0	0.0112729
FGFBP1	0.0208499	0	0
FGFBP2	0.0106871	0	0.0106871
FGFBP3	0.0759178	0.0759178	0
FGFR1	0.0046618	0	0.0046618
FGFR1OP	0.03319	0	0
FGFR1OP2	0.1505155	0	0.1505155
FGFR2	0.0046791	0	0
FGFR3	0.0003306	0.0003306	0
FGFR4	0.002816	0.002816	0
FGFRL1	0.0018835	0.0018835	0
FGG	0.0092007	0.0092007	0
FGGY	0.0852244	0	0
FGL1	0.5795984	0	0
FGL2	0.0378927	0	0.0378927
FGR	0.1395906	0	0.1395906
FH	0.2579361	0.2579361	0
FHAD1	0.0201538	0	0
FHDC1	0.0092147	0.0092147	0
FHIT	0.024869	0	0
FHL1	0.0461372	0	0.0461372

FHL2	0.5504291	0.5504291	0
FHL3	0.0436456	0	0.0436456
FHL5	0.0089284	0	0.0089284
FHOD1	0.0263137	0	0.0263137
FHOD3	0.017886	0	0.017886
FIBCD1	0.0246458	0.0246458	0
FIBIN	0.0538756	0	0.0538756
FIBP	0.082134	0.082134	0
FICD	0.0237759	0	0.0237759
FIG4	0.080127	0	0.080127
FIGF	0.017582	0	0.017582
FIGLA	0.0053935	0	0.0053935
FIGN	0.0306871	0	0.0306871
FIGNL1	0.1701338	0	0
FIGNL2	0.0011784	0	0.0011784
FILIP1	0.0843462	0	0.0843462
FILIP1L	0.078214	0	0.078214
FIP1L1	0.033162	0.033162	0
FIS1	0.0185084	0.0185084	0
FITM1	0.0135639	0	0
FITM2	0.026316	0	0.026316
FIZ1	0.1058022	0.1058022	0
FJX1	0.0546732	0	0.0546732
FKBP10	0.0058267	0	0.0058267
FKBP11	0.0117849	0	0
FKBP14	0.1491995	0	0.1491995
FKBP15	0.0913866	0	0.0913866
FKBP1A	0.0496193	0	0.0496193
FKBP1AP1	0.0035448	0	0.0035448
FKBP1B	0.0153782	0.0153782	0
FKBP2	0.0282988	0.0282988	0
FKBP3	0.150105	0.150105	0
FKBP4	0.0197492	0.0197492	0
FKBP5	0.5313415	0	0.5313415
FKBP6	0.0016925	0	0
FKBP7	0.0716974	0	0.0716974
FKBP8	0.0053933	0.0053933	0
FKBP9	0.0039058	0	0.0039058
FKBP9L	0.022596	0	0
FKRP	0.004708	0	0
FKSG29	0.0397821	0	0.0397821
FKSG73	0.0003016	0	0
FKSG83	0.0257138	0	0
FKTN	0.0223374	0	0
FLAD1	0.353267	0.353267	0

FLCN	0.019551	0	0.019551
FLG	0.030307	0	0.030307
FLG2	0.019085	0	0
FLI1	0.027777	0	0.027777
FLII	0.005693	0	0
FLJ10038	0.2586172	0	0.2586172
FLJ10213	0.015404	0	0
FLJ10357	0.0067819	0	0.0067819
FLJ10661	0.0021262	0.0021262	0
FLJ11235	0.0293338	0	0
FLJ12825	0.0068685	0.0068685	0
FLJ13197	0.0907902	0.0907902	0
FLJ13224	0.1603334	0.1603334	0
FLJ14107	0.0064107	0.0064107	0
FLJ16779	0.0004966	0	0.0004966
FLJ22536	0.0053811	0	0.0053811
FLJ23867	0.0042743	0.0042743	0
FLJ25328	0.003205	0	0
FLJ25363	0.0012378	0	0
FLJ25758	0.024969	0.024969	0
FLJ26850	0.0067866	0	0
FLJ30679	0.0141111	0	0
FLJ32063	0.0045149	0.0045149	0
FLJ33360	0.0064108	0	0
FLJ33630	0.1643219	0.1643219	0
FLJ34503	0.0059544	0	0
FLJ35024	0.0174257	0	0
FLJ35220	0.0058679	0.0058679	0
FLJ35390	0.0016387	0	0
FLJ35776	1	0	0
FLJ36000	0.0004271	0	0
FLJ36031	0.084073	0	0.084073
FLJ36777	0.002382	0	0
FLJ37201	0.0789408	0.0789408	0
FLJ37307	0.0116784	0	0
FLJ37453	0.061802	0	0
FLJ37543	0.0070515	0	0.0070515
FLJ39582	0.0076275	0.0076275	0
FLJ39609	0.0007869	0	0.0007869
FLJ39653	0.0258745	0.0258745	0
FLJ40292	0.0497958	0.0497958	0
FLJ40330	0.0076021	0	0.0076021
FLJ40434	0.0050491	0	0
FLJ40504	0.0075642	0	0
FLJ40852	0.0816508	0	0.0816508

FLJ41941	0.0717874	0	0.0717874
FLJ42289	0.3389132	0	0.3389132
FLJ42393	0.0447191	0	0
FLJ42627	1.99E-05	0	0
FLJ42709	0.0298772	0	0.0298772
FLJ42875	0.0002187	0.0002187	0
FLJ43390	0.0253218	0	0.0253218
FLJ43663	0.3367337	0	0
FLJ43859	1.54E-05	0	0
FLJ43860	0.0028787	0	0
FLJ43950	0.0033597	0	0.0033597
FLJ44054	0.0029005	0	0.0029005
FLJ44082	6.46E-05	0	0
FLJ44606	0.0580363	0.0580363	0
FLJ44635	0.0059913	0	0.0059913
FLJ45079	0.0113564	0	0.0113564
FLJ45244	0.0710359	0.0710359	0
FLJ45340	0.0001698	0.0001698	0
FLJ45445	1.53E-05	1.53E-05	0
FLJ45983	0.0058446	0	0
FLJ46111	0.0068323	0.0068323	0
FLJ46321	0.0053693	0	0
FLJ46361	0.0054984	0	0
FLJ90757	0.0025754	0.0025754	0
FLNA	0.0051666	0	0.0051666
FLNB	0.1224854	0.1224854	0
FLNC	0.0007821	0	0.0007821
FLOT2	0.594713	0	0
FLRT1	0.0021116	0	0
FLRT2	0.0735139	0	0.0735139
FLRT3	0.0464753	0	0
FLT1	0.003114	0	0.003114
FLT3	0.0027455	0	0.0027455
FLT3LG	0.0083626	0	0.0083626
FLT4	0.0010985	0	0.0010985
FLVCR1	0.098432	0.098432	0
FLVCR2	0.0258464	0	0.0258464
FLYWCH1	0.0527168	0	0
FLYWCH2	0.024443	0.024443	0
FMN1	0.0188857	0	0
FMN2	0.0077754	0	0.0077754
FMNL1	0.0550627	0	0.0550627
FMNL2	0.029055	0	0.029055
FMNL3	0.0521836	0	0.0521836
FMO1	0.0266671	0	0.0266671

FMO2	0.0287315	0	0.0287315
FMO3	0.0101268	0	0.0101268
FMO4	0.0980334	0	0.0980334
FMO5	0.0067173	0.0067173	0
FMO6P	0.0391054	0	0
FMO9P	0.0171092	0.0171092	0
FMOD	0.0238714	0	0.0238714
FMR1	0.0154095	0	0.0154095
FMR1NB	0.0014634	0	0
FN1	0.0273393	0	0.0273393
FN3K	0.001168	0.001168	0
FN3KRP	0.0035974	0.0035974	0
FNBP1	0.1515479	0	0.1515479
FNBP1L	0.048736	0.048736	0
FNBP4	0.2228328	0	0
FNDC1	0.0029943	0	0.0029943
FNDC3A	0.1288261	0	0
FNDC3B	0.2590332	0	0.2590332
FNDC4	0.0191807	0	0.0191807
FNDC5	0.0040222	0	0.0040222
FNDC7	0.0423763	0	0
FNDC8	0.0079213	0	0
FNIP1	0.0762962	0	0.0762962
FNIP2	0.0141921	0	0.0141921
FNTA	0.0405487	0	0
FNTB	0.0725399	0	0
FOLH1	0.0053699	0	0.0053699
FOLH1B	0.0047592	0	0
FOLR1	0.0115859	0	0
FOLR2	0.0163936	0	0.0163936
FOLR3	0.0282897	0	0.0282897
FOLR4	0.0062638	0	0.0062638
FOS	0.8803395	0	0
FOSB	0.0215347	0	0.0215347
FOSL1	0.0738616	0	0
FOSL2	0.9063535	0	0.9063535
FOXA1	0.0151669	0.0151669	0
FOXA2	0.0010052	0.0010052	0
FOXA3	0.0076979	0.0076979	0
FOXB1	0.0140325	0	0
FOXB2	0.0012565	0	0.0012565
FOXC1	0.0121015	0	0
FOXC2	0.014783	0	0.014783
FOXD1	0.0125629	0	0
FOXD2	0.0087097	0.0087097	0

FOXD3	0.0036387	0	0.0036387
FOXD4	0.0001434	0.0001434	0
FOXD4L1	5.20E-05	5.20E-05	0
FOXD4L3	1.55E-05	0	0
FOXD4L5	1.70E-05	0	0
FOXD4L6	1.55E-05	1.55E-05	0
FOXE1	0.0018161	0	0.0018161
FOXE3	0.0062364	0	0
FOXF1	0.0016408	0	0.0016408
FOXF2	0.0052132	0	0.0052132
FOXG1	0.0035452	0	0.0035452
FOXH1	0.0038355	0.0038355	0
FOXI1	0.0046438	0	0
FOXI2	0.001696	0	0.001696
FOXI3	0.0054466	0.0054466	0
FOXJ1	0.0054494	0.0054494	0
FOXJ2	0.072989	0	0.072989
FOXJ3	0.0778939	0	0.0778939
FOXK1	0.0123019	0	0
FOXK2	0.0300684	0.0300684	0
FOXL1	0.02487	0.02487	0
FOXL2	0.0040771	0	0.0040771
FOXM1	0.0248154	0.0248154	0
FOXN1	0.0057613	0	0
FOXN2	0.3249672	0	0.3249672
FOXN3	0.1198786	0	0.1198786
FOXN4	0.0100885	0	0.0100885
FOXO1	0.1051797	0	0.1051797
FOXO3	0.1427803	0	0.1427803
FOXO3B	0.0012645	0	0.0012645
FOXO4	0.0120741	0.0120741	0
FOXP1	0.0456648	0	0.0456648
FOXP2	0.0344576	0	0.0344576
FOXP3	0.0065358	0	0.0065358
FOXP4	0.0167903	0.0167903	0
FOXQ1	0.0099511	0.0099511	0
FOXR1	0.0021863	0	0
FOXR2	0.0030391	0	0
FOXRED1	0.0175682	0.0175682	0
FOXRED2	0.0086784	0	0.0086784
FOXS1	0.0062004	0	0.0062004
FPGS	0.2183985	0.2183985	0
FPGT	0.0356609	0	0.0356609
FPR1	0.002066	0	0.002066
FPR2	0.0024509	0	0.0024509

FPR3	0.0035664	0	0.0035664
FRAS1	0.0199927	0	0
FRAT1	0.0129078	0	0
FRAT2	0.0424611	0.0424611	0
FREM1	0.0085481	0	0.0085481
FREM2	0.0051664	0	0
FRG1	0.2727288	0	0
FRG1B	0.2413464	0	0
FRG2	0.0005779	0	0
FRG2B	0.0001985	0	0
FRG2C	0.2116012	0	0
FRK	0.1055361	0	0
FRMD1	0.0005928	0	0.0005928
FRMD3	0.0104304	0	0.0104304
FRMD4A	0.0098998	0	0.0098998
FRMD4B	0.0084488	0	0.0084488
FRMD5	0.0157801	0.0157801	0
FRMD6	0.1448287	0	0.1448287
FRMD7	0.0015886	0	0.0015886
FRMD8	0.0559355	0	0
FRMPD1	0.0059858	0	0.0059858
FRMPD2	0.0550436	0	0
FRMPD2L1	8.18E-05	8.18E-05	0
FRMPD4	0.004161	0	0.004161
FRRS1	0.0811035	0	0.0811035
FRS2	0.0352654	0	0.0352654
FRS3	0.0167384	0.0167384	0
FRY	0.0257952	0	0
FRYL	0.0579369	0	0
FRZB	0.0035306	0	0.0035306
FSCB	0.0040981	0	0
FSCN1	0.0299035	0	0
FSCN2	0.0019038	0.0019038	0
FSCN3	0.0075028	0	0
FSD1	0.0008199	0	0.0008199
FSD1L	0.0299051	0	0.0299051
FSD2	0.0925206	0	0
FSHB	0.0049101	0.0049101	0
FSHR	0.0045302	0	0
FSIP1	0.0903739	0	0.0903739
FST	0.181503	0	0.181503
FSTL1	0.0113206	0	0.0113206
FSTL3	0.0149292	0	0.0149292
FSTL4	0.0084413	0	0
FSTL5	0.0109382	0	0.0109382

FTCD	0.0007137	0	0
FTH1	1	0	1
FTHL17	0.0050331	0.0050331	0
FTHL3	0.0073866	0	0.0073866
FTL	0.0883212	0	0.0883212
FTLP10	0.0238346	0	0
FTMT	0.0074639	0	0
FTO	0.0434318	0	0.0434318
FTSJ1	0.0279746	0	0
FTSJ2	0.0271247	0.0271247	0
FTSJ3	0.0353139	0	0.0353139
FTSJD1	0.0331048	0	0.0331048
FTSJD2	0.0384635	0	0.0384635
FUBP1	0.2713791	0	0
FUBP3	0.0730161	0	0
FUCA1	0.0362979	0	0.0362979
FUCA2	0.0373918	0	0
FUK	0.0090468	0.0090468	0
FUNDC1	0.0141542	0.0141542	0
FUNDC2	0.0370191	0	0.0370191
FUNDC2P2	0.0072999	0	0
FURIN	0.0847241	0.0847241	0
FUS	0.0520656	0	0
FUT1	0.0022826	0.0022826	0
FUT10	0.0714011	0	0.0714011
FUT11	0.0575906	0	0.0575906
FUT2	0.0031469	0.0031469	0
FUT3	0.0017466	0.0017466	0
FUT4	0.0303658	0.0303658	0
FUT5	0.0011944	0	0
FUT6	0.0009134	0.0009134	0
FUT7	0.0046581	0	0.0046581
FUT8	0.052161	0	0.052161
FUT9	0.0038929	0	0
FUZ	0.0759506	0.0759506	0
FXC1	0.0553495	0.0553495	0
FXN	0.0196469	0	0
FXR1	0.0398974	0	0.0398974
FXR2	0.0199189	0	0.0199189
FXYD1	0.0040696	0	0.0040696
FXYD2	0.0244114	0	0
FXYD3	0.1989283	0.1989283	0
FXYD4	0.0020506	0.0020506	0
FXYD5	0.0262398	0.0262398	0
FXYD6	0.0015197	0	0.0015197

FXYD7	0.0045625	0	0.0045625
FYB	0.0751911	0	0.0751911
FYCO1	0.0295293	0	0.0295293
FYN	0.1944759	0	0.1944759
FYTTD1	0.0454836	0	0.0454836
FZD1	0.0184944	0	0.0184944
FZD10	0.0081534	0	0.0081534
FZD2	0.0098296	0	0.0098296
FZD3	0.0487897	0	0
FZD4	0.012689	0	0.012689
FZD5	0.0465431	0.0465431	0
FZD6	0.0720584	0	0
FZD7	0.0087224	0	0.0087224
FZD8	0.0069008	0	0.0069008
FZD9	0.0020955	0	0
FZR1	0.0216433	0.0216433	0
G0S2	0.6327381	0	0
G2E3	0.0707267	0	0
G3BP1	0.28136	0	0.28136
G3BP2	0.0734178	0	0
G6PC	0.0086544	0	0
G6PC2	0.0056902	0	0.0056902
G6PC3	0.0281381	0.0281381	0
G6PD	0.0120128	0	0.0120128
GAA	0.0034852	0	0.0034852
GAB1	0.0973341	0	0.0973341
GAB2	0.1560473	0	0.1560473
GAB3	0.0099051	0	0.0099051
GAB4	0.0008293	0	0.0008293
GABARAP	0.1234412	0	0.1234412
GABARAPL1	0.1687612	0	0.1687612
GABARAPL2	0.080539	0	0
GABARAPL3	0.0257683	0	0.0257683
GABBR2	0.0025506	0.0025506	0
GABPA	0.0605666	0	0.0605666
GABPB1	0.245365	0	0.245365
GABPB2	0.0960752	0	0
GABRA1	0.0150613	0	0.0150613
GABRA2	0.010977	0	0.010977
GABRA3	0.0039777	0	0.0039777
GABRA4	0.0146682	0	0.0146682
GABRA5	0.0011568	0	0.0011568
GABRA6	0.0157682	0	0
GABRB1	0.0134069	0	0
GABRB2	0.014931	0	0.014931

GABRB3	0.0013786	0	0
GABRD	0.0006253	0	0
GABRE	0.1212304	0.1212304	0
GABRG1	0.0051999	0	0.0051999
GABRG2	0.0107785	0	0.0107785
GABRG3	0.0014045	0	0.0014045
GABRP	0.0088642	0	0.0088642
GABRQ	0.0041354	0	0.0041354
GABRR1	0.0047158	0	0.0047158
GABRR2	0.0165387	0	0.0165387
GABRR3	0.0295453	0	0
GAD1	0.0110139	0.0110139	0
GAD2	0.0018418	0	0.0018418
GADD45A	0.5243354	0	0.5243354
GADD45B	0.2476401	0	0.2476401
GADD45G	0.0396373	0.0396373	0
GADD45GIP1	0.0392689	0.0392689	0
GADL1	0.0066438	0	0
GAGE1	8.83E-05	0	0
GAGE10	0.0032612	0	0
GAGE12J	0.0001233	0	0
GAGE13	1.57E-05	1.57E-05	0
GAGE2B	1.54E-05	0	0
GAGE4	1.68E-05	0	0
GAK	0.0218783	0.0218783	0
GAL	0.0023653	0	0.0023653
GAL3ST1	0.0014301	0.0014301	0
GAL3ST2	0.0017072	0	0
GAL3ST3	0.0142205	0	0
GAL3ST4	0.0038737	0	0.0038737
GALC	0.0540013	0	0.0540013
GALE	0.0509573	0.0509573	0
GALK1	0.0052497	0	0
GALK2	0.2502546	0.2502546	0
GALM	0.0354112	0	0
GALNS	0.0021768	0	0.0021768
GALNT1	0.0145344	0	0.0145344
GALNT10	0.0392911	0	0.0392911
GALNT11	0.0109987	0	0.0109987
GALNT12	0.0178418	0.0178418	0
GALNT13	0.0030208	0	0.0030208
GALNT14	0.0061872	0	0
GALNT2	0.0878568	0	0.0878568
GALNT3	0.0172313	0.0172313	0
GALNT4	0.0512815	0.0512815	0

GALNT5	0.0138113	0.0138113	0
GALNT6	0.0742831	0.0742831	0
GALNT7	0.0305452	0.0305452	0
GALNT8	0.004082	0	0
GALNT9	0.006747	0	0.006747
GALNTL1	0.0036453	0	0.0036453
GALNTL2	0.046702	0	0.046702
GALNTL4	0.0550932	0	0
GALNTL5	0.0037565	0	0
GALNTL6	0.0048011	0	0
GALP	0.0021436	0	0.0021436
GALR1	0.0031839	0	0.0031839
GALR2	0.0056882	0	0
GALR3	0.0005927	0.0005927	0
GALT	0.0298002	0.0298002	0
GAMT	0.0028702	0	0
GAN	0.0444395	0	0
GANAB	0.0363322	0	0
GANC	0.3479155	0	0.3479155
GAP43	0.0110581	0	0.0110581
GAPDH	0.1251702	0.1251702	0
GAPDHS	0.0024446	0.0024446	0
GAPT	0.0223177	0	0.0223177
GAPVD1	0.043969	0	0.043969
GAR1	0.0397985	0.0397985	0
GARNL3	0.0267332	0	0.0267332
GARS	0.0738056	0.0738056	0
GART	0.1281131	0	0
GAS1	0.0349516	0	0.0349516
GAS2	0.0402779	0	0
GAS2L1	0.005832	0	0
GAS2L2	0.00425	0	0
GAS2L3	0.2252936	0	0.2252936
GAS5	0.5828033	0.5828033	0
GAS6	0.0136894	0	0.0136894
GAS7	0.0105455	0	0.0105455
GAS8	0.0244142	0.0244142	0
GAST	0.0034359	0.0034359	0
GATA1	0.0023646	0	0.0023646
GATA2	0.002803	0	0.002803
GATA3	0.0061409	0	0.0061409
GATA4	0.0014907	0.0014907	0
GATA5	0.0004514	0	0
GATA6	0.0080216	0.0080216	0
GATAD1	0.0593952	0.0593952	0

GATAD2A	0.0134599	0.0134599	0
GATAD2B	0.0935018	0	0
GATC	0.0476819	0	0
GATM	0.0370993	0	0
GATS	0.0047757	0	0
GATSL1	1.79E-05	0	1.79E-05
GATSL2	1.53E-05	0	1.53E-05
GATSL3	0.0094954	0	0.0094954
GBA	0.1409731	0	0
GBA2	0.0270012	0.0270012	0
GBA3	0.0093334	0	0.0093334
GBAP1	0.7408101	0.7408101	0
GBAS	0.0300707	0	0
GBE1	0.1687065	0	0.1687065
GBF1	0.0965576	0.0965576	0
GBGT1	0.0060486	0	0.0060486
GBP1	0.0862515	0	0.0862515
GBP2	0.6154466	0	0.6154466
GBP3	0.0610292	0	0
GBP4	0.0312695	0	0.0312695
GBP5	0.196883	0	0.196883
GBP6	0.0198351	0	0.0198351
GBP7	0.0195811	0	0.0195811
GBX1	0.0039709	0	0
GBX2	0.0004595	0	0
GC	0.0036876	0	0
GCA	0.0180422	0	0.0180422
GCAT	0.0078278	0.0078278	0
GCC1	0.0131291	0	0
GCC2	0.0256973	0	0
GCDH	0.0338663	0	0
GCET2	0.5783546	0	0.5783546
GCFC1	0.2419614	0	0
GCG	0.011955	0	0.011955
GCGR	0.0001132	0	0.0001132
GCH1	0.0432705	0	0.0432705
GCHFR	0.0970744	0	0
GCK	0.0267643	0	0.0267643
GCKR	0.0158328	0.0158328	0
GCLC	0.0580774	0	0.0580774
GCLM	0.0950743	0	0.0950743
GCM1	0.0145059	0	0.0145059
GCM2	0.0074666	0.0074666	0
GCN1L1	0.1596181	0.1596181	0
GCNT1	0.0390223	0	0

GCNT2	0.0604769	0	0
GCNT3	0.1020309	0	0
GCNT4	0.0140708	0	0.0140708
GCNT7	0.0034493	0	0
GCOM1	0.0227391	0	0
GCSH	0.0389514	0.0389514	0
GDA	0.1604253	0	0
GDAP1	0.0247287	0	0
GDAP1L1	0.0037624	0	0.0037624
GDAP2	0.0900438	0	0.0900438
GDE1	0.1783183	0.1783183	0
GDEP	0.0044946	0	0
GDF1	0.0005462	0	0
GDF10	0.0025306	0	0.0025306
GDF11	0.0265482	0	0.0265482
GDF15	0.0585107	0.0585107	0
GDF2	0.0020614	0	0
GDF3	0.0025991	0	0.0025991
GDF5	0.0083249	0	0.0083249
GDF6	0.0047101	0	0.0047101
GDF7	0.0025203	0	0
GDF9	0.0576111	0.0576111	0
GDI1	0.0015219	0.0015219	0
GDI2	0.0807557	0	0.0807557
GDNF	0.0170547	0	0.0170547
GDPD1	0.0253903	0.0253903	0
GDPD2	0.0027515	0.0027515	0
GDPD3	0.0039322	0.0039322	0
GDPD4	0.0267365	0	0
GDPD5	0.0138317	0	0.0138317
GEFT	0.0025108	0	0.0025108
GEM	0.0469512	0	0.0469512
GEMIN4	0.0126998	0	0
GEMIN5	0.0938479	0	0.0938479
GEMIN6	0.0139481	0.0139481	0
GEMIN7	0.0117503	0.0117503	0
GEMIN8	0.1609346	0.1609346	0
GEMIN8P4	0.2021377	0.2021377	0
GEN1	0.2226182	0.2226182	0
GET4	0.0143648	0.0143648	0
GFAP	0.0634084	0	0.0634084
GFER	0.0050591	0.0050591	0
GFI1	0.0096687	0	0.0096687
GFI1B	0.0041258	0	0
GFM1	0.0483653	0	0

GFM2	0.117304	0.117304	0
GFOD1	0.0858202	0	0.0858202
GFOD2	0.0230785	0	0
GFPT1	0.0484285	0.0484285	0
GFPT2	0.0034973	0	0.0034973
GFRA1	0.0039622	0	0.0039622
GFRA2	0.0009185	0	0.0009185
GFRA3	0.005134	0	0.005134
GFRA4	0.002058	0	0.002058
GFRAL	0.0110289	0	0
GGA1	0.0284512	0.0284512	0
GGA2	0.0484919	0	0.0484919
GGA3	0.0283735	0	0
GGCT	0.1004325	0.1004325	0
GGCX	0.0250038	0	0
GGH	0.0259777	0.0259777	0
GGN	0.0071415	0.0071415	0
GGNBP1	0.0173314	0	0.0173314
GGNBP2	0.1084688	0	0
GGPS1	0.7072869	0.7072869	0
GGT1	0.0030865	0	0
GGT3P	1.63E-05	0	0
GGT5	0.032587	0	0.032587
GGT6	0.0214432	0	0
GGT7	0.0380457	0.0380457	0
GGT8P	0.0009491	0.0009491	0
GGTA1	0.0075228	0	0.0075228
GGTLC1	0.0001781	0	0
GGTLC2	4.36E-05	0	0
GH1	0.0110638	0	0.0110638
GH2	0.0012024	0	0
GHDC	0.0091757	0.0091757	0
GHITM	0.0481723	0	0
GHR	0.0101309	0	0.0101309
GHRH	0.0054745	0	0
GHRHR	0.0039269	0.0039269	0
GHRL	0.0142654	0	0.0142654
GHRLOS	0.0139975	0	0
GHSR	0.0130662	0	0.0130662
GIF	0.0082205	0	0
GIGYF1	0.0063497	0.0063497	0
GIGYF2	0.0279758	0	0.0279758
GIMAP1	0.0027151	0	0.0027151
GIMAP2	0.013741	0	0.013741
GIMAP4	0.0053297	0	0.0053297

GIMAP5	0.020211	0	0.020211
GIMAP6	0.0108689	0	0.0108689
GIMAP7	0.0169262	0	0.0169262
GIMAP8	0.0502242	0	0.0502242
GIN1	0.2322329	0	0
GINS1	0.0224596	0.0224596	0
GINS2	0.008818	0.008818	0
GINS3	0.048206	0	0.048206
GINS4	0.0147976	0	0.0147976
GIP	0.0025713	0	0
GIPC1	0.0411076	0.0411076	0
GIPC2	0.0606693	0.0606693	0
GIPC3	0.0012895	0	0.0012895
GIPR	0.0039952	0.0039952	0
GIT1	0.0141946	0.0141946	0
GIT2	0.0935929	0	0.0935929
GJA1	0.0091337	0	0.0091337
GJA10	0.0048719	0	0
GJA3	0.0133967	0	0.0133967
GJA4	0.0132596	0	0.0132596
GJA5	0.029734	0	0.029734
GJA8	0.0233864	0.0233864	0
GJA9	0.0078631	0.0078631	0
GJB1	0.0016863	0.0016863	0
GJB2	0.0147535	0	0.0147535
GJB3	0.0334279	0.0334279	0
GJB4	0.0173881	0	0
GJB5	0.0349359	0	0
GJB6	0.0024715	0.0024715	0
GJB7	0.0070947	0	0
GJC1	0.0127372	0	0.0127372
GJC2	0.0082525	0.0082525	0
GJC3	0.0119923	0.0119923	0
GJD2	0.0104592	0	0.0104592
GJD3	0.0042708	0	0.0042708
GJD4	0.0041149	0	0
GK	0.1281193	0	0.1281193
GK2	0.0055508	0.0055508	0
GK3P	0.0057258	0	0.0057258
GK5	0.0160117	0.0160117	0
GKAP1	0.0078584	0	0
GKN1	0.0166028	0.0166028	0
GKN2	0.0111587	0.0111587	0
GLA	0.1410225	0	0.1410225
GLB1	0.0295621	0	0

GLB1L	0.0200051	0	0.0200051
GLB1L2	0.0023924	0.0023924	0
GLB1L3	0.0070334	0	0.0070334
GLCCI1	0.168487	0	0.168487
GLCE	0.0575549	0	0
GLDC	0.0727611	0	0.0727611
GLDN	0.0157081	0	0.0157081
GLE1	0.0388422	0	0
GLG1	0.0360351	0	0.0360351
GLI1	0.0369531	0	0.0369531
GLI2	0.001377	0	0.001377
GLI3	0.022309	0	0.022309
GLI4	0.0043292	0.0043292	0
GLIPR1	0.1280226	0	0.1280226
GLIPR1L1	0.0365051	0	0
GLIPR1L2	0.1098586	0	0.1098586
GLIPR2	0.0145429	0	0.0145429
GLIS1	0.0091993	0	0.0091993
GLIS2	0.0011581	0	0.0011581
GLIS3	0.0968262	0	0.0968262
GLMN	0.0646023	0	0.0646023
GLO1	0.0410405	0	0
GLOD4	0.0220871	0.0220871	0
GLOD5	0.0073178	0.0073178	0
GLP1R	0.0017214	0	0.0017214
GLP2R	0.0129577	0	0.0129577
GLRA1	0.0099311	0	0.0099311
GLRA2	0.0107706	0	0
GLRA3	0.0157626	0	0.0157626
GLRA4	0.0038778	0	0
GLRB	0.0072037	0	0.0072037
GLRX	0.2229813	0	0.2229813
GLRX2	0.1222192	0.1222192	0
GLRX3	0.0191898	0.0191898	0
GLRX5	0.1288699	0.1288699	0
GLS	0.0844956	0	0.0844956
GLS2	0.0038965	0	0
GLT1D1	0.0032134	0	0.0032134
GLT25D1	0.0221656	0	0.0221656
GLT25D2	0.0423644	0	0
GLT6D1	0.0067947	0	0
GLT8D1	0.0664522	0	0.0664522
GLT8D2	0.0301801	0	0.0301801
GLTP	0.054827	0	0.054827
GLTPD1	0.0033566	0.0033566	0

GLTPD2	0.1328825	0.1328825	0
GLTSCR1	0.0208423	0.0208423	0
GLTSCR2	0.023118	0.023118	0
GLUD1	0.0889844	0.0889844	0
GLUD2	0.0041039	0	0
GLUL	0.0695937	0	0.0695937
GLYAT	0.0125243	0	0.0125243
GLYATL1	0.006227	0	0
GLYATL2	0.0106756	0	0.0106756
GLYATL3	0.0096831	0	0
GLYCAM1	0.0123289	0	0
GLYCTK	0.0512079	0.0512079	0
GLYR1	0.0250379	0.0250379	0
GM2A	0.2854743	0	0.2854743
GMCL1	0.0480124	0	0
GMCL1L	0.0057697	0	0
GMDS	0.5735433	0.5735433	0
GMEB1	0.0157368	0	0.0157368
GMEB2	0.0137068	0.0137068	0
GMFB	0.0676067	0	0.0676067
GMFG	0.0143551	0	0.0143551
GMIP	0.0039078	0	0.0039078
GML	0.0039895	0	0.0039895
GMNN	0.0863676	0	0
GMPPA	0.0180336	0.0180336	0
GMPPB	0.0103357	0.0103357	0
GMPR	0.00759	0	0
GMPR2	0.2472172	0.2472172	0
GMPS	0.0347228	0.0347228	0
GNA11	0.0009589	0.0009589	0
GNA12	0.0404023	0	0.0404023
GNA13	0.0952445	0	0.0952445
GNA14	0.0034264	0	0.0034264
GNA15	0.0551555	0.0551555	0
GNAI1	0.0421306	0	0
GNAI2	0.0988091	0	0.0988091
GNAI3	0.1652678	0	0.1652678
GNAL	0.0185635	0	0.0185635
GNAO1	0.0072826	0	0.0072826
GNAQ	0.0298848	0	0
GNAS	0.0414589	0	0
GNASAS	0.0051587	0	0.0051587
GNAT1	0.0024121	0	0
GNAT2	0.0488688	0	0
GNAT3	0.0153321	0	0

GNAZ	0.0023169	0	0
GNB1	0.0073874	0	0.0073874
GNB1L	0.0203122	0	0
GNB2	0.33381	0.33381	0
GNB2L1	0.2817496	0.2817496	0
GNB3	0.003505	0	0.003505
GNB4	0.0383517	0	0.0383517
GNB5	0.0361154	0	0
GNE	0.0549837	0	0
GNG10	0.0156782	0	0
GNG11	0.0690589	0	0.0690589
GNG12	0.0630027	0	0.0630027
GNG13	0.0007686	0	0
GNG2	0.0761779	0	0.0761779
GNG3	0.0297844	0	0.0297844
GNG4	0.0119156	0	0.0119156
GNG5	0.1024292	0.1024292	0
GNG7	0.0079925	0	0.0079925
GNG8	0.0026424	0	0.0026424
GNGT1	0.0929903	0	0
GNGT2	0.0236796	0	0.0236796
GNL2	0.0307926	0	0
GNL3	0.1528729	0	0
GNL3L	0.0144597	0	0.0144597
GNLY	0.044329	0	0.044329
GNMT	0.0017299	0	0.0017299
GNPAT	0.0836834	0.0836834	0
GNPDA1	0.4433356	0	0
GNPDA2	0.0418203	0	0.0418203
GNPNAT1	0.0637549	0.0637549	0
GNPTAB	0.0510968	0	0.0510968
GNPTG	0.0021112	0.0021112	0
GNRH1	0.0198293	0.0198293	0
GNRH2	0.0041191	0	0
GNRHR	0.014654	0	0
GNRHR2	0.1779265	0.1779265	0
GNS	0.0753284	0	0.0753284
GOLGA1	0.1095377	0	0
GOLGA2	0.0260861	0.0260861	0
GOLGA2B	0.007783	0.007783	0
GOLGA3	0.0091809	0.0091809	0
GOLGA4	0.0729662	0.0729662	0
GOLGA5	0.1057003	0.1057003	0
GOLGA6A	1.53E-05	0	0
GOLGA6B	1.55E-05	0	0

GOLGA6C	0.0001832	0	0
GOLGA6D	2.06E-05	0	0
GOLGA6L1	1.53E-05	0	0
GOLGA6L5	2.51E-05	0	0
GOLGA6L6	1.59E-05	0	0
GOLGA7	0.0212087	0	0.0212087
GOLGA7B	0.0094675	0.0094675	0
GOLGA8A	0.0167973	0.0167973	0
GOLGA8B	0.0127284	0.0127284	0
GOLGA8C	1.55E-05	0	1.55E-05
GOLGA8DP	1.58E-05	0	0
GOLGA8E	1.53E-05	0	0
GOLGA9P	4.68E-05	0	0
GOLGB1	0.1027852	0	0
GOLIM4	0.1575666	0	0.1575666
GOLM1	0.0201374	0.0201374	0
GOLPH3	0.1424638	0	0
GOLPH3L	0.1941391	0.1941391	0
GOLT1A	0.042617	0.042617	0
GOLT1B	0.1102955	0	0.1102955
GON4L	0.133675	0	0
GOPC	0.0702143	0	0.0702143
GORAB	0.387198	0	0
GORASP1	0.0133153	0.0133153	0
GORASP2	0.13784	0.13784	0
GOSR1	0.0680563	0	0
GOSR2	0.0409471	0	0.0409471
GOT1	0.0990334	0.0990334	0
GOT1L1	0.002833	0	0
GOT2	0.0303647	0.0303647	0
GP1BA	0.0121746	0	0.0121746
GP2	0.0029653	0	0
GP5	0.0190131	0	0.0190131
GP6	0.0023777	0.0023777	0
GP9	0.0061787	0.0061787	0
GPA33	0.0398228	0	0
GPAA1	0.2936721	0.2936721	0
GPAM	0.0491659	0	0.0491659
GPAT2	1.70E-05	0	1.70E-05
GPATCH1	0.0270642	0.0270642	0
GPATCH2	0.161821	0.161821	0
GPATCH3	0.0211545	0	0
GPATCH4	0.2661514	0	0
GPATCH8	0.0549977	0	0
GPBAR1	0.0074212	0	0.0074212

GPBP1	0.1616708	0	0
GPBP1L1	0.1684871	0	0
GPC1	0.0029755	0	0
GPC2	0.0051308	0	0
GPC3	0.0027322	0	0.0027322
GPC4	0.0027233	0	0
GPC5	0.0381317	0	0.0381317
GPC6	0.0483747	0	0.0483747
GPCPD1	0.1991745	0	0.1991745
GPD1	0.0447544	0	0.0447544
GPD1L	0.0182021	0.0182021	0
GPD2	0.1001338	0.1001338	0
GPER	0.003539	0.003539	0
GPHA2	0.0045745	0	0.0045745
GPHB5	0.0061503	0	0
GPHN	0.149903	0.149903	0
GPI	0.0318979	0.0318979	0
GPIHBP1	0.0024069	0	0.0024069
GPKOW	0.0042238	0.0042238	0
GPLD1	0.0138986	0	0.0138986
GPM6A	0.0095844	0	0.0095844
GPM6B	0.0211857	0	0.0211857
GPN1	0.1183457	0	0
GPN2	0.0141521	0	0
GPN3	0.0496342	0	0.0496342
GPNMB	0.024716	0	0.024716
GPR1	0.0059949	0	0.0059949
GPR101	0.003721	0	0
GPR107	0.050813	0	0.050813
GPR108	0.1708294	0.1708294	0
GPR109A	0.2475967	0	0.2475967
GPR109B	0.4670878	0	0.4670878
GPR110	0.1434989	0.1434989	0
GPR111	0.0325605	0	0.0325605
GPR112	0.0024857	0	0.0024857
GPR113	0.1579963	0	0
GPR114	0.0446322	0	0.0446322
GPR115	0.176521	0	0
GPR116	0.0167958	0	0.0167958
GPR119	0.0039254	0	0.0039254
GPR12	0.0019013	0	0.0019013
GPR120	0.0032645	0	0
GPR123	0.0002329	0	0.0002329
GPR124	0.0027457	0	0.0027457
GPR125	0.0195473	0.0195473	0

GPR126	0.1202819	0	0
GPR128	0.1624129	0	0.1624129
GPR132	0.0278947	0	0.0278947
GPR133	0.0090787	0	0.0090787
GPR135	0.0187297	0	0.0187297
GPR137	0.0110265	0	0
GPR137B	0.0202744	0	0.0202744
GPR137C	0.0412582	0	0.0412582
GPR139	0.0020139	0	0
GPR141	0.0368257	0	0.0368257
GPR142	0.0022005	0	0.0022005
GPR143	0.0025378	0	0
GPR144	0.0043162	0	0
GPR146	0.003138	0	0.003138
GPR148	0.0019639	0	0.0019639
GPR149	0.0022271	0.0022271	0
GPR15	0.2260842	0	0.2260842
GPR150	0.034364	0	0
GPR151	0.0320917	0	0
GPR152	0.0044755	0.0044755	0
GPR153	0.0020076	0	0
GPR155	0.0503816	0	0.0503816
GPR156	0.0136131	0	0.0136131
GPR157	0.0224903	0	0.0224903
GPR158	0.0049902	0	0
GPR160	0.0244277	0.0244277	0
GPR161	0.0397097	0	0.0397097
GPR162	0.0036112	0	0.0036112
GPR17	0.0006692	0	0.0006692
GPR171	0.030208	0	0.030208
GPR172A	0.0041735	0.0041735	0
GPR172B	0.0044268	0	0.0044268
GPR173	0.0039043	0	0.0039043
GPR174	0.0203033	0	0.0203033
GPR176	0.0342212	0	0.0342212
GPR179	0.0088621	0	0
GPR18	0.0471778	0	0.0471778
GPR180	0.0966395	0	0
GPR182	0.0103777	0	0.0103777
GPR183	0.2466468	0	0.2466468
GPR19	0.1288622	0	0
GPR20	0.0023899	0.0023899	0
GPR21	0.0448748	0	0.0448748
GPR22	0.0252656	0	0.0252656
GPR25	0.0838477	0	0.0838477

GPR26	0.0013385	0	0.0013385
GPR27	0.0027679	0	0
GPR3	0.0101972	0	0.0101972
GPR31	0.0239067	0	0.0239067
GPR32	0.0021814	0	0
GPR34	0.0124049	0	0.0124049
GPR35	0.0008331	0.0008331	0
GPR37	0.0921656	0	0
GPR37L1	0.0311367	0.0311367	0
GPR39	0.0212406	0.0212406	0
GPR4	0.0033877	0	0.0033877
GPR44	0.0027079	0	0
GPR45	0.0043403	0	0
GPR50	0.0012091	0	0
GPR52	0.0159553	0	0
GPR55	0.0261171	0	0.0261171
GPR56	0.1128858	0.1128858	0
GPR6	0.0040839	0	0
GPR61	0.0552154	0	0
GPR62	0.0024209	0	0
GPR63	0.0119774	0	0.0119774
GPR64	0.0061483	0	0.0061483
GPR65	0.0676405	0	0.0676405
GPR68	0.1015519	0	0.1015519
GPR75	0.0125466	0	0.0125466
GPR77	0.006081	0	0.006081
GPR78	0.0007304	0.0007304	0
GPR81	0.0718391	0	0
GPR82	0.0208027	0	0.0208027
GPR83	0.0064504	0	0
GPR84	0.0600272	0	0.0600272
GPR85	0.0139635	0	0.0139635
GPR87	0.2995423	0	0
GPR88	0.0184139	0	0.0184139
GPR89A	0.0009808	0.0009808	0
GPR89B	0.0066382	0.0066382	0
GPR97	0.0126513	0	0.0126513
GPR98	0.0089942	0	0
GPRASP1	0.003531	0	0.003531
GPRASP2	0.0032013	0	0.0032013
GPRC5A	0.8380255	0.8380255	0
GPRC5B	0.0043642	0	0.0043642
GPRC5C	0.028639	0.028639	0
GPRC5D	0.007244	0.007244	0
GPRC6A	0.0040387	0	0

GPRIN1	0.008005	0.008005	0
GPRIN2	0.0203652	0	0
GPRIN3	0.0269568	0	0.0269568
GPS1	0.0006403	0.0006403	0
GPS2	0.0282139	0.0282139	0
GPSM1	0.0009134	0	0
GPSM2	0.063141	0.063141	0
GPT	0.0018089	0.0018089	0
GPT2	0.0112484	0.0112484	0
GPX1	0.1757964	0	0
GPX2	0.0130958	0.0130958	0
GPX3	0.029144	0	0.029144
GPX4	0.0887238	0.0887238	0
GPX5	0.0070301	0	0
GPX6	0.006286	0.006286	0
GPX7	0.0094506	0	0.0094506
GPX8	0.0576277	0	0.0576277
GRAMD1A	0.4572921	0	0
GRAMD1B	0.0110404	0	0
GRAMD1C	0.1552858	0.1552858	0
GRAMD2	0.0414314	0.0414314	0
GRAMD3	0.7384435	0	0
GRAMD4	0.0003147	0.0003147	0
GRAP	1.53E-05	0	1.53E-05
GRAP2	0.0851417	0	0.0851417
GRAPL	1.53E-05	0	1.53E-05
GRASP	0.0038304	0	0.0038304
GRB10	0.0238215	0	0.0238215
GRB14	0.0044131	0	0
GRB2	0.05324	0	0.05324
GRB7	0.0436779	0.0436779	0
GREB1	0.0522304	0	0.0522304
GREB1L	0.0092268	0.0092268	0
GREM1	0.0045146	0	0.0045146
GREM2	0.0326117	0	0.0326117
GRHL1	0.0165921	0.0165921	0
GRHL2	0.0129216	0.0129216	0
GRHL3	0.0601131	0.0601131	0
GRHPR	0.0188912	0.0188912	0
GRIA1	0.0145337	0	0.0145337
GRIA2	0.0051042	0	0.0051042
GRIA3	0.0056223	0	0.0056223
GRIA4	0.0070657	0	0.0070657
GRID1	0.0021762	0	0.0021762
GRID2	0.0057962	0	0.0057962

GRID2IP	0.0014115	0.0014115	0
GRIK1	0.0078794	0	0.0078794
GRIK2	0.0091092	0	0.0091092
GRIK3	0.0009414	0	0.0009414
GRIK4	0.0084213	0	0.0084213
GRIK5	0.0018593	0	0.0018593
GRIN1	0.0026581	0.0026581	0
GRIN2A	0.0066283	0	0.0066283
GRIN2B	0.0050178	0	0.0050178
GRIN2C	0.0009605	0	0.0009605
GRIN2D	0.0060817	0	0
GRIN3A	0.0054883	0	0.0054883
GRIN3B	0.0008483	0	0
GRINA	0.0676163	0	0
GRINL1A	0.0779143	0	0.0779143
GRIP1	0.011514	0	0.011514
GRIP2	0.0020103	0	0.0020103
GRIPAP1	0.0114189	0.0114189	0
GRK1	0.000383	0	0
GRK4	0.0203324	0	0.0203324
GRK5	0.0583044	0	0.0583044
GRK6	0.0392853	0	0
GRK7	0.0039065	0	0
GRLF1	0.035222	0	0
GRM1	0.0040233	0	0.0040233
GRM2	0.0011495	0	0.0011495
GRM3	0.0047448	0.0047448	0
GRM4	0.0011842	0	0
GRM5	0.0098466	0	0.0098466
GRM6	0.000697	0	0.000697
GRM7	0.0049285	0	0.0049285
GRM8	0.0086606	0	0
GRN	0.0270875	0	0.0270875
GRP	0.0072153	0	0.0072153
GRPEL1	0.0271253	0.0271253	0
GRPEL2	0.0915337	0	0
GRPR	0.0316273	0	0.0316273
GRRP1	0.0032971	0	0.0032971
GRSF1	0.0102749	0.0102749	0
GRTP1	0.0014632	0.0014632	0
GRWD1	0.0296437	0.0296437	0
GRXCR1	0.0077393	0	0
GRXCR2	0.0066626	0	0
GSC	0.0039358	0	0.0039358
GSC2	0.0021762	0	0

GSDMA	0.0128208	0	0.0128208
GSDMB	0.0117153	0.0117153	0
GSDMC	1	0	1
GSDMD	0.5878097	0.5878097	0
GSG1	0.3791066	0	0.3791066
GSG1L	0.002035	0	0.002035
GSG2	0.02447	0	0
GSK3A	0.1237935	0.1237935	0
GSK3B	0.2611275	0	0
GSN	0.0487945	0	0.0487945
GSPT1	0.0702333	0	0
GSPT2	0.0091745	0	0
GSR	0.0930648	0	0
GSS	0.0640321	0.0640321	0
GSTA1	0.0043172	0	0
GSTA2	0.0067468	0	0
GSTA3	0.0569162	0	0
GSTA4	0.1611034	0	0.1611034
GSTA5	0.007121	0	0
GSTCD	0.1181717	0	0
GSTK1	0.0750614	0.0750614	0
GSTM1	0.0010417	0	0.0010417
GSTM2	0.0054909	0	0.0054909
GSTM2P1	0.0082514	0	0.0082514
GSTM3	0.0049409	0	0.0049409
GSTM4	0.0611224	0	0.0611224
GSTM5	0.0118043	0	0.0118043
GSTO1	0.0769935	0	0
GSTO2	0.0512042	0.0512042	0
GSTP1	0.0538493	0.0538493	0
GSTT1	0.0035459	0.0035459	0
GSTTP1	0.0004324	0	0
GSTTP2	0.0061361	0.0061361	0
GSTZ1	0.0420374	0.0420374	0
GSX1	0.0021923	0	0
GSX2	0.0029633	0	0.0029633
GTDC1	0.0489292	0	0.0489292
GTF2A1	0.0838274	0	0.0838274
GTF2A1L	0.00327	0	0.00327
GTF2A2	0.0752288	0	0
GTF2B	0.1035972	0	0
GTF2E1	0.0313742	0	0.0313742
GTF2E2	0.084737	0	0
GTF2F1	0.0054293	0.0054293	0
GTF2F2	0.0281308	0.0281308	0

GTF2H1	0.3784544	0	0.3784544
GTF2H3	0.0189844	0	0.0189844
GTF2H5	0.0206333	0	0.0206333
GTF2I	0.0968364	0	0
GTF2IRD1	0.0146264	0.0146264	0
GTF2IRD2	1.57E-05	1.57E-05	0
GTF2IRD2B	2.47E-05	2.47E-05	0
GTF2IRD2P1	2.09E-05	0	0
GTF3A	0.0358495	0.0358495	0
GTF3C1	0.0263967	0	0.0263967
GTF3C2	0.0368948	0.0368948	0
GTF3C3	0.0461208	0.0461208	0
GTF3C4	0.0720123	0	0.0720123
GTF3C5	0.0401977	0.0401977	0
GTF3C6	0.0508177	0	0
GTPBP1	0.2215194	0.2215194	0
GTPBP10	0.0588617	0.0588617	0
GTPBP2	0.1289587	0	0
GTPBP3	0.0925518	0.0925518	0
GTPBP4	0.0480576	0	0
GTPBP5	0.0351034	0.0351034	0
GTPBP8	0.0937465	0	0.0937465
GTSE1	0.0054436	0.0054436	0
GTSF1	0.0183988	0	0.0183988
GTSF1L	0.017542	0	0.017542
GUCA1A	0.0045567	0	0.0045567
GUCA1B	0.0164243	0	0
GUCA1C	0.0208239	0	0.0208239
GUCA2A	0.005316	0	0
GUCA2B	0.0065127	0	0
GUCY1A2	0.010696	0	0.010696
GUCY1A3	0.0126946	0	0.0126946
GUCY1B2	0.0058915	0.0058915	0
GUCY1B3	0.0089273	0	0.0089273
GUCY2C	0.0069912	0	0
GUCY2D	0.0315732	0	0
GUCY2E	0.0066585	0	0
GUCY2F	0.0085315	0	0
GUCY2GP	0.0125775	0.0125775	0
GUF1	0.0466095	0.0466095	0
GUK1	0.0864918	0.0864918	0
GULP1	0.0116114	0	0.0116114
GUSB	0.0127996	0	0
GUSBL1	0.0009971	0	0
GUSBL2	0.000883	0	0.000883

GUSBP1	0.0022272	0	0
GVIN1	0.0103416	0	0.0103416
GXYLT1	0.0751862	0	0.0751862
GXYLT2	0.0411725	0	0.0411725
GYG1	0.0710249	0	0.0710249
GYG2	0.0024394	0	0.0024394
GYLTL1B	0.0158416	0	0
GYPA	0.0012276	0	0
GYPB	0.000624	0	0.000624
GYPC	0.0459064	0	0.0459064
GYPE	0.0007206	0	0.0007206
GYS1	0.0272569	0.0272569	0
GYS2	0.0282864	0	0
GZF1	0.0462111	0	0.0462111
GZMA	0.00625	0	0.00625
GZMB	0.0065464	0	0.0065464
GZMH	0.004784	0	0.004784
GZMK	0.0050134	0	0.0050134
GZMM	0.0004466	0	0.0004466
H19	0.0004944	0	0.0004944
H1F0	0.0061163	0.0061163	0
H1FNT	0.0023206	0	0.0023206
H1FOO	0.0020351	0	0
H1FX	0.0521542	0.0521542	0
H2AFJ	0.2720252	0.2720252	0
H2AFV	0.1333473	0	0
H2AFX	0.1896996	0.1896996	0
H2AFY	0.0247249	0.0247249	0
H2AFY2	0.0080763	0	0
H2AFZ	0.1392828	0.1392828	0
H2BFM	0.0030584	0	0
H2BFWT	0.0049768	0	0
H3F3A	0.1243607	0.1243607	0
H3F3B	0.6610116	0	0
H3F3C	0.0062452	0	0
H6PD	0.2195582	0	0.2195582
HAAO	0.0201563	0	0.0201563
HABP2	0.0740445	0	0
HABP4	0.0181244	0	0.0181244
HACE1	0.0796416	0	0.0796416
HACL1	0.0912037	0	0
HADH	0.0367319	0	0
HADHA	0.2590906	0	0
HADHB	0.2305992	0	0
HAGH	0.0103304	0.0103304	0

HAGHL	0.0021173	0.0021173	0
HAL	0.0215712	0	0
HAMP	0.003039	0	0.003039
HAND1	0.0130552	0	0.0130552
HAND2	0.0007697	0	0.0007697
HAO1	0.0124873	0	0
HAO2	0.0776779	0	0.0776779
HAP1	0.0033926	0	0
HAPLN1	0.0278819	0.0278819	0
HAPLN2	0.0259496	0.0259496	0
HAPLN3	0.0046202	0	0.0046202
HAPLN4	0.0009879	0	0.0009879
HAR1A	0.0014263	0	0.0014263
HAR1B	0.0015664	0	0.0015664
HARBI1	0.0427982	0	0
HARS	0.0890146	0.0890146	0
HARS2	0.0808786	0.0808786	0
HAS1	0.0029829	0	0.0029829
HAS2	0.1567839	0	0.1567839
HAS2AS	0.1912439	0	0.1912439
HAS3	0.0066583	0.0066583	0
HAT1	0.0424678	0	0.0424678
HAUS1	0.0246669	0	0.0246669
HAUS2	0.0436322	0	0.0436322
HAUS3	0.0142714	0	0.0142714
HAUS4	0.0224665	0	0.0224665
HAUS5	0.0082029	0.0082029	0
HAUS6	0.0605252	0	0.0605252
HAUS7	0.0012688	0	0
HAUS8	0.0471008	0	0
HAVCR1	0.0186857	0	0.0186857
HAVCR2	0.0480228	0	0.0480228
HAX1	0.0793821	0.0793821	0
HBA1	0.0081524	0	0
HBA2	0.0065492	0	0.0065492
HBB	0.0039291	0	0.0039291
HBBP1	0.0097001	0	0
HBD	0.0131563	0	0.0131563
HBE1	0.0113513	0	0.0113513
HBEGF	0.1665105	0	0.1665105
HBG1	0.005777	0	0.005777
HBG2	0.0046966	0	0.0046966
HBII-52-24	0.0003047	0	0
HBII-52-27	0.0002903	0	0
HBII-52-28	0.0002553	0	0

HBII-52-45	0.0018771	0	0
HBII-52-46	0.0031366	0	0
HBM	0.0044539	0	0
HBP1	0.2722892	0	0.2722892
HBQ1	0.003803	0.003803	0
HBS1L	0.052679	0	0.052679
HBXIP	0.1729371	0.1729371	0
HBZ	0.0011041	0	0
HCCS	0.0270379	0.0270379	0
HCFC1	0.0100526	0.0100526	0
HCFC1R1	0.0200178	0	0
HCFC2	0.0356847	0	0.0356847
HCG11	0.0829676	0	0.0829676
HCK	0.0104455	0	0.0104455
HCLS1	0.0444113	0	0.0444113
HCN1	0.0055335	0	0.0055335
HCN2	0.0015969	0	0.0015969
HCN3	0.0305155	0.0305155	0
HCN4	0.000933	0	0
HCRT	0.0070058	0.0070058	0
HCRTR1	0.0029004	0	0.0029004
HCRTR2	0.0111966	0	0.0111966
HCST	0.056887	0	0.056887
HDAC1	0.017369	0.017369	0
HDAC10	0.0024388	0.0024388	0
HDAC11	0.0136261	0.0136261	0
HDAC2	0.068467	0	0
HDAC3	0.0267299	0	0
HDAC4	0.0342628	0	0.0342628
HDAC5	0.0077809	0	0
HDAC6	0.010712	0	0
HDAC7	0.628044	0	0.628044
HDAC8	0.0527901	0.0527901	0
HDAC9	0.0312743	0	0.0312743
HDC	0.0876695	0	0.0876695
HDDC2	0.0369251	0	0
HDDC3	0.0782769	0.0782769	0
HDGF	0.1949882	0.1949882	0
HDGFL1	0.0092497	0	0
HDGFRP2	0.0425343	0.0425343	0
HDGFRP3	0.075314	0	0.075314
HDHD1A	0.0676949	0	0
HDHD2	0.0598571	0	0.0598571
HDHD3	0.0121924	0.0121924	0
HDLBP	0.0961506	0	0

HDX	0.0077099	0	0.0077099
HEATR1	0.8569387	0	0
HEATR2	0.0069334	0.0069334	0
HEATR3	0.0348352	0	0.0348352
HEATR4	0.0022427	0.0022427	0
HEATR5A	0.023059	0	0.023059
HEATR5B	0.0912406	0	0.0912406
HEATR6	0.2941269	0	0.2941269
HEATR7A	1.53E-05	1.53E-05	0
HEATR7B2	0.0123876	0	0.0123876
HEBP1	0.0152933	0	0.0152933
HEBP2	0.0483381	0.0483381	0
HECA	0.0261325	0	0.0261325
HECTD1	0.0505778	0	0
HECTD2	0.0466146	0	0.0466146
HECTD3	0.038483	0	0
HECW1	0.0252201	0	0.0252201
HECW2	0.0094363	0	0.0094363
HEG1	0.0922449	0	0.0922449
HELB	0.6108083	0	0.6108083
HELLS	0.0536573	0.0536573	0
HELQ	0.0660052	0	0.0660052
HELT	0.001818	0	0
HELZ	0.0577307	0	0.0577307
HEMGN	0.0078057	0	0.0078057
HEMK1	0.0251267	0.0251267	0
HEPACAM	0.0050581	0	0.0050581
HEPACAM2	0.0550617	0	0
HEPH	0.015242	0	0.015242
HEPHL1	0.0397555	0.0397555	0
HEPN1	0.0013572	0	0.0013572
HERC1	0.0859041	0	0.0859041
HERC2	0.0001029	0	0.0001029
HERC2P2	1.99E-05	1.99E-05	0
HERC2P4	1.74E-05	0	0
HERC3	0.0401159	0	0
HERC4	0.1077898	0	0
HERC5	0.0661768	0	0.0661768
HERC6	0.0292741	0	0.0292741
HERPUD1	0.101973	0	0.101973
HERPUD2	0.1449342	0	0
HES1	0.3657184	0.3657184	0
HES2	0.01859	0	0
HES3	0.0064354	0	0
HES4	0.0037136	0.0037136	0

HES5	0.0026769	0.0026769	0
HES6	0.0025417	0.0025417	0
HES7	0.1795751	0	0
HESRG	0.0027543	0	0
HESX1	0.0165521	0	0
HEXA	0.0509604	0	0.0509604
HEXB	0.051468	0	0.051468
HEXDC	0.0095601	0.0095601	0
HEXIM1	0.1311073	0.1311073	0
HEXIM2	0.0775528	0.0775528	0
HEY1	0.0358185	0	0.0358185
HEY2	0.0228145	0	0.0228145
HEYL	0.002511	0	0.002511
HFE	0.0248463	0	0.0248463
HFE2	0.0490179	0.0490179	0
HFM1	0.014012	0	0.014012
HGC6.3	0.0020851	0	0.0020851
HGF	0.0286287	0	0.0286287
HGFAC	0.0005247	0.0005247	0
HGS	0.0056517	0.0056517	0
HGSNAT	0.0313324	0	0.0313324
HHAT	0.2272358	0	0
HHATL	0.0013051	0	0.0013051
HHEX	0.0096357	0	0.0096357
HHIP	0.0051806	0	0.0051806
HHIPL1	0.0015055	0	0.0015055
HHIPL2	0.0375625	0	0
HHLA1	0.0145003	0	0
HHLA2	0.0202298	0	0
HHLA3	0.1081296	0	0
HIAT1	0.1537509	0	0.1537509
HIATL1	0.000521	0	0.000521
HIATL2	0.0004798	0.0004798	0
HIBADH	0.0414546	0.0414546	0
HIBCH	0.0838308	0.0838308	0
HIC1	0.0060969	0	0.0060969
HIC2	1.53E-05	0	0
HIF1A	0.1601037	0	0.1601037
HIF1AN	0.0762141	0	0
HIF3A	0.0037831	0	0
HIGD1A	0.0283285	0.0283285	0
HIGD1B	0.005116	0	0.005116
HIGD1C	0.0073088	0	0
HIGD2A	0.0640351	0.0640351	0
HIGD2B	0.079853	0.079853	0

HILS1	0.0129231	0	0.0129231
HINFP	0.0282552	0.0282552	0
HINT1	0.1493553	0.1493553	0
HINT2	0.0375972	0.0375972	0
HINT3	0.1588856	0	0.1588856
HIP1	0.0147149	0	0.0147149
HIP1R	0.0373804	0.0373804	0
HIPK1	0.1601573	0	0.1601573
HIPK2	0.1167752	0	0
HIPK3	0.4995176	0	0.4995176
HIPK4	0.0009614	0	0.0009614
HIRA	0.0389381	0	0
HIRIP3	0.0076347	0	0
HIST1H1A	0.3903739	0	0
HIST1H1B	0.1015099	0.1015099	0
HIST1H1C	0.3309965	0.3309965	0
HIST1H1D	0.1782519	0	0
HIST1H1E	0.3950269	0.3950269	0
HIST1H1T	0.1090413	0	0
HIST1H2AA	0.0114519	0	0
HIST1H2AB	1	0	0
HIST1H2AC	1	1	0
HIST1H2AD	0.8939745	0	0
HIST1H2AE	0.4748963	0.4748963	0
HIST1H2AG	0.579028	0	0
HIST1H2AH	0.4056626	0	0
HIST1H2AJ	0.4731148	0	0
HIST1H2AK	0.473826	0.473826	0
HIST1H2AL	0.1193469	0	0
HIST1H2AM	0.4669631	0.4669631	0
HIST1H2BA	0.0118116	0	0
HIST1H2BB	0.4131315	0.4131315	0
HIST1H2BC	1	1	0
HIST1H2BD	0.5207949	0.5207949	0
HIST1H2BE	0.1098126	0.1098126	0
HIST1H2BF	0.8497776	0	0
HIST1H2BG	0.4681881	0	0
HIST1H2BH	0.1570673	0	0
HIST1H2BI	0.3367935	0	0
HIST1H2BJ	0.5751239	0.5751239	0
HIST1H2BK	0.4264552	0.4264552	0
HIST1H2BL	0.297814	0	0
HIST1H2BM	0.4868773	0	0
HIST1H2BN	0.4735849	0.4735849	0
HIST1H2BO	0.492333	0.492333	0

HIST1H3A	0.5836746	0	0
HIST1H3B	1	1	0
HIST1H3C	0.3435408	0.3435408	0
HIST1H3D	0.8964085	0	0
HIST1H3E	0.4898387	0	0
HIST1H3F	0.1662937	0	0
HIST1H3G	0.3099598	0	0
HIST1H3H	0.3326801	0.3326801	0
HIST1H3I	0.0799786	0	0
HIST1H3J	0.2204541	0.2204541	0
HIST1H4A	0.6977296	0	0
HIST1H4B	1	1	0
HIST1H4C	0.2787524	0	0
HIST1H4D	0.3323696	0.3323696	0
HIST1H4E	1	1	0
HIST1H4F	0.1508805	0	0
HIST1H4G	0.1059371	0.1059371	0
HIST1H4H	0.7420656	0	0
HIST1H4I	0.7269369	0	0
HIST1H4J	0.2980087	0	0
HIST1H4K	0.5486605	0	0
HIST1H4L	0.0483651	0	0.0483651
HIST2H2AB	0.6329023	0.6329023	0
HIST2H2AC	0.6056853	0.6056853	0
HIST2H2BA	2.32E-05	2.32E-05	0
HIST2H2BE	0.5863007	0.5863007	0
HIST2H2BF	0.002664	0.002664	0
HIST2H3D	0.0011683	0.0011683	0
HIST3H2A	0.1682375	0.1682375	0
HIST3H2BB	0.1588702	0.1588702	0
HIST3H3	0.0164298	0.0164298	0
HIST4H4	0.3100828	0.3100828	0
HIVEP1	0.2891173	0	0.2891173
HIVEP2	0.6140994	0	0.6140994
HIVEP3	0.0363304	0	0.0363304
HJURP	0.0158114	0.0158114	0
HK1	0.0559105	0	0
HK2	0.0416674	0.0416674	0
HK3	0.0133962	0	0.0133962
HKDC1	0.1049948	0.1049948	0
HKR1	0.0080594	0.0080594	0
HLA-DQA1	0.03478	0	0.03478
HLA-DQB1	0.014767	0	0.014767
HLA-DRB5	0.0015829	0	0.0015829
HLCS	0.0108828	0.0108828	0

HLF	0.0097341	0	0.0097341
HLTF	0.0655011	0	0
HLX	0.0300473	0	0.0300473
HM13	0.0195662	0.0195662	0
HMBOX1	0.1385318	0	0.1385318
HMBS	0.03106	0.03106	0
HMCN1	0.0471962	0	0.0471962
HMG20A	0.1539848	0	0.1539848
HMG20B	0.026843	0.026843	0
HMGA1	0.0201242	0.0201242	0
HMGA2	0.0791554	0	0
HMGB1	0.0368792	0	0
HMGB2	0.0883562	0	0
HMGB3	0.0029414	0.0029414	0
HMGB3L1	0.0190713	0	0
HMGB4	0.0033535	0	0
HMGCL	0.0289553	0.0289553	0
HMGCLL1	0.006385	0	0.006385
HMGCR	0.131796	0.131796	0
HMGCS1	0.2397557	0.2397557	0
HMGCS2	0.0167264	0.0167264	0
HMGN1	0.0379172	0.0379172	0
HMGN2	0.0438039	0	0
HMGN3	0.1441113	0	0
HMGN4	0.2457388	0	0.2457388
HMGN5	0.0293288	0	0
HMGXB3	0.0990867	0	0
HMGXB4	0.0230288	0	0.0230288
HMHA1	0.0084901	0	0.0084901
HMHB1	0.0361298	0	0.0361298
HMMR	0.1914688	0.1914688	0
HMOX1	0.0403324	0	0.0403324
HMOX2	0.0547043	0	0
HMP19	0.0202017	0	0.0202017
HMSD	0.0488199	0	0.0488199
HMX1	0.0002747	0	0.0002747
HMX2	0.022406	0	0
HMX3	0.0073082	0	0
HN1	0.4167647	0.4167647	0
HN1L	0.0052587	0.0052587	0
HNF1A	0.0037284	0.0037284	0
HNF1B	0.0526122	0.0526122	0
HNF4A	0.0412987	0.0412987	0
HNF4G	0.0197442	0.0197442	0
HNMT	0.0632294	0	0.0632294

HNRNPA0	0.2372988	0	0
HNRNPA1	0.5824197	0	0
HNRNPA1L2	0.0319755	0	0
HNRNPA2B1	0.4752354	0.4752354	0
HNRNPA3	0.0889623	0	0
HNRNPA3P1	0.0050592	0	0.0050592
HNRNPAB	0.0267598	0.0267598	0
HNRNPC	0.2817907	0	0
HNRNPCL1	0.0023326	0	0
HNRNPD	0.1104412	0.1104412	0
HNRNPF	0.2789369	0.2789369	0
HNRNPH1	0.1223411	0	0
HNRNPH2	0.1240208	0	0.1240208
HNRNPH3	0.1734655	0	0.1734655
HNRNPK	0.2210088	0	0
HNRNPL	0.0933767	0.0933767	0
HNRNPM	0.020551	0.020551	0
HNRNPR	0.1003623	0	0.1003623
HNRNPU	0.8161941	0	0
HNRNPUL1	0.106875	0	0
HNRNPUL2	0.2074477	0	0.2074477
HNRPDL	0.0833297	0	0
HNRPLL	0.1183637	0	0.1183637
HOMER1	0.0495655	0	0.0495655
HOMER2	0.0089516	0	0
HOMER3	0.00238	0	0.00238
HOMEZ	0.0318911	0.0318911	0
HOOK1	0.0274187	0.0274187	0
HOOK2	0.2399695	0.2399695	0
HOOK3	0.0498253	0	0.0498253
HOPX	0.1687604	0	0.1687604
HORMAD1	0.0112841	0	0.0112841
HORMAD2	0.0161893	0	0.0161893
HOTAIR	0.01419	0	0
HOXA1	0.0248151	0	0.0248151
HOXA10	0.005096	0.005096	0
HOXA11	0.00506	0	0
HOXA11AS	0.0054275	0.0054275	0
HOXA13	0.00784	0	0
HOXA2	0.0559355	0	0.0559355
HOXA3	0.0772894	0	0
HOXA4	0.0355922	0	0.0355922
HOXA5	0.0737789	0	0.0737789
HOXA6	0.0146602	0	0
HOXA7	0.0067707	0	0.0067707

HOXA9	0.0051889	0	0
HOXB1	0.0026131	0.0026131	0
HOXB13	0.0021218	0.0021218	0
HOXB2	0.0070742	0	0
HOXB3	0.0104027	0.0104027	0
HOXB4	0.0178501	0	0.0178501
HOXB5	0.0075829	0.0075829	0
HOXB6	0.0242697	0.0242697	0
HOXB7	0.0215536	0.0215536	0
HOXB8	0.0090757	0.0090757	0
HOXB9	0.0041134	0.0041134	0
HOXC10	0.0811097	0.0811097	0
HOXC11	0.035311	0.035311	0
HOXC12	0.0048286	0	0
HOXC13	0.0130291	0	0
HOXC4	0.0589377	0	0
HOXC5	0.0584061	0	0
HOXC6	0.0604475	0	0
HOXC8	0.0225945	0	0.0225945
HOXC9	0.0140908	0.0140908	0
HOXD1	0.0033263	0	0.0033263
HOXD10	0.0016138	0	0
HOXD11	0.0016421	0	0
HOXD12	0.0018117	0.0018117	0
HOXD13	0.0019715	0.0019715	0
HOXD3	0.0011705	0	0.0011705
HOXD4	0.0010877	0	0.0010877
HOXD8	0.0023617	0	0.0023617
HOXD9	0.0016665	0	0.0016665
HP	0.0125733	0	0.0125733
HP1BP3	0.034226	0	0.034226
HPCA	0.0014807	0	0
HPCAL1	0.0343495	0.0343495	0
HPCAL4	0.0032879	0	0.0032879
HPD	0.0589814	0	0.0589814
HPDL	0.0131518	0.0131518	0
HPGD	0.015976	0	0
HPGDS	0.0132201	0	0.0132201
HPN	0.0019163	0	0
HPR	0.0056596	0	0.0056596
HPRT1	0.015506	0.015506	0
HPS1	0.0206595	0	0
HPS3	0.1110269	0	0
HPS4	0.0498262	0.0498262	0
HPS5	0.3660751	0	0.3660751

HPS6	0.0284715	0	0
HPSE	0.0148593	0	0
HPSE2	0.0058416	0	0
HPVC1	0.0038422	0	0.0038422
HPX	0.012943	0.012943	0
HPYR1	0.0079503	0.0079503	0
HR	0.018322	0.018322	0
HRAS	0.0019685	0.0019685	0
HRASLS	0.0109038	0.0109038	0
HRASLS2	0.285085	0	0
HRASLS5	0.0178845	0	0.0178845
HRC	0.0033305	0	0.0033305
HRCT1	0.0665371	0.0665371	0
HRG	0.0173156	0	0
HRH1	0.8686086	0	0
HRH2	0.2450901	0	0.2450901
HRH3	0.0005596	0.0005596	0
HRH4	0.0127578	0	0.0127578
HRK	0.0077515	0	0
HRNBP3	0.0010252	0	0.0010252
HRNR	0.0202664	0	0.0202664
HRSP12	0.0313177	0.0313177	0
HS1BP3	0.0187687	0.0187687	0
HS2ST1	0.1441452	0	0.1441452
HS3ST1	0.0187145	0.0187145	0
HS3ST2	0.0033257	0	0.0033257
HS3ST3A1	0.0113799	0	0.0113799
HS3ST3B1	0.0064561	0	0.0064561
HS3ST4	0.0030477	0	0.0030477
HS3ST5	0.0118705	0.0118705	0
HS3ST6	0.0005241	0.0005241	0
HS6ST1	0.0016342	0	0.0016342
HS6ST2	0.0031361	0	0.0031361
HS6ST3	0.0092891	0	0.0092891
HSBP1	0.0296833	0.0296833	0
HSBP1L1	0.0329148	0.0329148	0
HSCB	0.6038918	0	0
HSD11B1	0.5167308	0	0.5167308
HSD11B1L	0.0109729	0.0109729	0
HSD11B2	0.0030328	0.0030328	0
HSD17B1	0.0077457	0.0077457	0
HSD17B10	0.0317902	0.0317902	0
HSD17B11	0.0277447	0	0
HSD17B12	0.0349565	0.0349565	0
HSD17B13	0.0110495	0	0.0110495

HSD17B14	0.0083105	0	0.0083105
HSD17B2	0.0190225	0	0
HSD17B3	0.0080763	0.0080763	0
HSD17B4	0.023817	0	0
HSD17B6	0.0166163	0	0.0166163
HSD17B7	0.185873	0.185873	0
HSD17B7P2	0.0053068	0.0053068	0
HSD3B1	0.0037878	0	0
HSD3B2	0.0172277	0	0
HSD3B7	0.0342259	0	0
HSDL1	0.0129817	0	0.0129817
HSDL2	0.0968397	0.0968397	0
HSF1	0.0084633	0.0084633	0
HSF2	0.0328519	0	0.0328519
HSF2BP	0.0177673	0	0
HSF4	0.0264757	0.0264757	0
HSF5	0.0053315	0	0.0053315
HSFYL1	0.019517	0	0
HSH2D	0.0144243	0	0
HSN2	0.2311491	0	0
HSP90AA1	0.0649427	0.0649427	0
HSP90AB1	0.1999205	0.1999205	0
HSP90AB2P	0.003798	0	0
HSP90AB4P	0.0265922	0	0
HSP90B1	0.2143882	0	0
HSP90B3P	0.0072559	0	0
HSPA12A	0.006387	0	0.006387
HSPA12B	0.0014399	0	0.0014399
HSPA13	0.0727052	0	0.0727052
HSPA14	0.0422521	0	0
HSPA2	0.0278218	0	0.0278218
HSPA4	0.0967845	0	0
HSPA4L	0.2475492	0	0
HSPA5	0.2602261	0.2602261	0
HSPA6	0.1716908	0	0.1716908
HSPA7	0.008902	0	0.008902
HSPA8	0.1860656	0	0.1860656
HSPA9	0.1605074	0.1605074	0
HSPB1	0.1396436	0.1396436	0
HSPB11	0.0431513	0	0
HSPB2	0.0054272	0	0.0054272
HSPB3	0.0076528	0	0
HSPB6	0.0175216	0	0.0175216
HSPB7	0.0051063	0	0.0051063
HSPB8	0.0191664	0	0.0191664

HSPB9	0.0055684	0.0055684	0
HSPBAP1	0.9700916	0	0.9700916
HSPBP1	0.0099665	0.0099665	0
HSPC072	0.0212573	0	0.0212573
HSPC157	0.0742171	0	0.0742171
HSPC159	0.0627652	0	0
HSPD1	0.6824606	0.6824606	0
HSPE1	0.6660638	0.6660638	0
HSPG2	0.0107175	0	0.0107175
HSPH1	0.081528	0.081528	0
HTA	0.5541215	0	0.5541215
HTATIP2	0.1051848	0.1051848	0
HTATSF1	0.0238684	0.0238684	0
HTN1	0.0050895	0	0
HTN3	0.0028153	0	0
HTR1A	0.0045672	0	0
HTR1B	0.0603133	0	0
HTR1D	0.0711009	0	0
HTR1E	0.0050136	0	0.0050136
HTR1F	0.0035719	0	0.0035719
HTR2A	0.008562	0	0.008562
HTR2B	0.04068	0	0.04068
HTR2C	0.004288	0	0
HTR3A	0.018179	0	0
HTR3B	0.0060727	0	0
HTR3C	0.0146212	0	0
HTR3D	0.0221354	0	0.0221354
HTR3E	0.006186	0	0
HTR4	0.0213458	0	0
HTR5A	0.0101578	0	0
HTR6	0.0030598	0	0.0030598
HTR7	0.0569058	0	0.0569058
HTR7P1	0.0147648	0	0.0147648
HTRA1	0.0271485	0	0.0271485
HTRA2	0.0809855	0	0.0809855
HTRA3	0.0017761	0	0.0017761
HTRA4	0.0149122	0	0.0149122
HTT	0.0254718	0	0
HULC	0.0096445	0.0096445	0
HUNK	0.0027891	0	0.0027891
HUS1	0.028069	0	0
HUS1B	0.0132901	0	0
HUWE1	0.0776093	0	0.0776093
HVCN1	0.0240589	0	0.0240589
HYAL1	0.0048032	0.0048032	0

HYAL2	0.0106694	0	0.0106694
HYAL3	0.0190605	0.0190605	0
HYAL4	0.0042253	0	0
HYALP1	0.0457624	0.0457624	0
HYDIN	0.0040182	0	0
HYI	0.0172777	0.0172777	0
HYLS1	0.0200914	0.0200914	0
HYMAI	0.0282647	0	0.0282647
HYOU1	0.0092962	0.0092962	0
IAH1	0.0183605	0	0
IAPP	0.0046622	0	0.0046622
IARS	0.110332	0	0
IARS2	0.1323845	0.1323845	0
IBSP	0.0063386	0	0.0063386
IBTK	0.0794804	0	0
ICA1	0.0155966	0.0155966	0
ICA1L	0.010441	0	0.010441
ICAM1	0.5949436	0	0.5949436
ICAM2	0.0359987	0	0.0359987
ICAM3	0.0115622	0	0.0115622
ICAM4	0.023082	0	0.023082
ICAM5	0.0150357	0	0
ICK	0.6779558	0	0.6779558
ICMT	0.0135232	0	0
ICOS	0.0054982	0	0.0054982
ICOSLG	0.0208062	0	0.0208062
ICT1	0.0082103	0.0082103	0
ID1	0.7430077	0.7430077	0
ID2	0.1022197	0	0.1022197
ID2B	0.011647	0	0.011647
ID3	0.2655415	0	0.2655415
ID4	0.0066827	0	0.0066827
IDE	0.0766801	0	0.0766801
IDH1	0.0914473	0.0914473	0
IDH2	0.0351684	0	0
IDH3A	0.4278746	0	0.4278746
IDH3B	0.025071	0.025071	0
IDH3G	0.0016795	0.0016795	0
IDI1	0.0829344	0	0
IDI2	0.0046433	0.0046433	0
IDO1	0.0328863	0	0.0328863
IDO2	0.170869	0	0.170869
IDS	0.0154235	0	0.0154235
IDUA	0.0016366	0.0016366	0
IER2	0.2199593	0.2199593	0

IER3IP1	0.0281732	0	0
IER5	0.5387305	0	0
IER5L	0.0459023	0.0459023	0
IFFO1	0.0185684	0	0.0185684
IFFO2	0.0777692	0	0.0777692
IFI16	1	0	1
IFI27	0.0050331	0	0
IFI27L1	0.0539104	0.0539104	0
IFI27L2	0.0169187	0.0169187	0
IFI30	0.0334987	0	0.0334987
IFI35	0.1516047	0	0.1516047
IFI44	0.0675217	0	0.0675217
IFI44L	0.2135427	0	0.2135427
IFI6	0.0463923	0	0
IFIH1	0.0844874	0	0.0844874
IFIT1	0.097656	0	0.097656
IFIT1B	0.0423678	0	0.0423678
IFIT2	0.2023437	0	0.2023437
IFIT3	0.2699717	0	0.2699717
IFIT5	0.0324134	0	0.0324134
IFITM1	0.183682	0	0.183682
IFITM2	0.1425433	0	0.1425433
IFITM3	0.3479059	0	0.3479059
IFITM5	0.0112993	0	0
IFLTD1	0.0363512	0	0
IFNA1	0.0066857	0.0066857	0
IFNA10	0.0017934	0	0
IFNA13	0.0054748	0.0054748	0
IFNA14	0.0103446	0	0
IFNA16	0.0011178	0	0
IFNA17	0.0027762	0.0027762	0
IFNA2	0.0275483	0	0
IFNA21	0.0059304	0	0
IFNA4	0.0047879	0	0
IFNA5	0.0058899	0	0
IFNA6	0.0154884	0	0
IFNA7	0.0027374	0	0
IFNA8	0.0093966	0.0093966	0
IFNAR1	0.0492493	0	0.0492493
IFNAR2	0.0799408	0	0.0799408
IFNB1	0.0126184	0	0.0126184
IFNE	0.0046043	0.0046043	0
IFNG	0.0218119	0	0.0218119
IFNGR1	0.8326528	0	0.8326528
IFNGR2	0.1008739	0	0

IFNK	0.0857767	0	0.0857767
IFNW1	0.0096595	0	0
IFRD1	0.0892766	0.0892766	0
IFRD2	0.0174237	0.0174237	0
IFT122	0.1170514	0.1170514	0
IFT140	0.0153576	0.0153576	0
IFT172	0.0272111	0.0272111	0
IFT20	0.7566819	0.7566819	0
IFT52	0.0199218	0	0.0199218
IFT57	0.0464194	0	0.0464194
IFT74	0.2114798	0	0
IFT80	0.1859612	0	0
IFT81	0.0194735	0	0
IFT88	0.0225198	0.0225198	0
IGBP1	0.02515	0.02515	0
IGDCC3	0.003444	0	0
IGDCC4	0.005844	0	0.005844
IGF1	0.156951	0	0.156951
IGF1R	0.1627921	0	0.1627921
IGF2	0.0021022	0	0.0021022
IGF2AS	0.000664	0	0.000664
IGF2BP1	0.0158864	0	0.0158864
IGF2BP2	0.0445055	0.0445055	0
IGF2BP3	0.2337097	0	0
IGF2R	0.1035266	0	0.1035266
IGFALS	0.0010105	0.0010105	0
IGFBP1	0.0521146	0	0
IGFBP2	0.0028091	0.0028091	0
IGFBP3	0.0241919	0	0.0241919
IGFBP4	0.0103601	0	0.0103601
IGFBP5	0.0094093	0	0.0094093
IGFBP6	0.0216662	0	0.0216662
IGFBP7	0.0359491	0	0.0359491
IGFBPL1	0.0048038	0	0.0048038
IGFL1	0.0088974	0	0
IGFL2	0.0101505	0	0.0101505
IGFL3	0.0211705	0	0
IGFL4	0.0099326	0	0.0099326
IGFN1	0.049189	0	0.049189
IGHMBP2	0.0190204	0.0190204	0
IGJ	0.0239455	0	0.0239455
IGLL1	0.0007347	0	0.0007347
IGLL3	0.0008251	0	0.0008251
IGLON5	0.0018273	0	0
IGSF1	0.0031621	0	0.0031621

IGSF10	0.0191457	0	0.0191457
IGSF11	0.0098571	0	0.0098571
IGSF21	0.0015959	0	0.0015959
IGSF22	0.0064833	0.0064833	0
IGSF3	0.01405	0	0
IGSF5	0.0016031	0.0016031	0
IGSF6	0.0385437	0	0.0385437
IGSF8	0.0285707	0.0285707	0
IGSF9	0.013489	0.013489	0
IGSF9B	0.0021123	0.0021123	0
IHH	0.0014211	0.0014211	0
IK	0.1046587	0	0
IKBIP	0.2029833	0	0.2029833
IKBKAP	0.0500164	0	0
IKBKB	0.0456447	0	0
IKBKE	0.1042889	0	0.1042889
IKBKG	0.0209482	0	0
IKZF1	0.0354419	0	0.0354419
IKZF2	0.1427992	0	0.1427992
IKZF3	0.0867439	0	0.0867439
IKZF4	0.006577	0	0.006577
IKZF5	0.0369382	0	0.0369382
IL10	0.15757	0	0.15757
IL10RA	0.0127314	0	0.0127314
IL10RB	0.0703447	0	0
IL11	0.001047	0	0.001047
IL11RA	0.0146325	0	0.0146325
IL12A	0.0269443	0	0.0269443
IL12B	0.2614752	0	0.2614752
IL12RB1	0.0115408	0	0.0115408
IL12RB2	0.0081424	0	0.0081424
IL13	0.039735	0	0.039735
IL13RA1	0.2126881	0	0.2126881
IL13RA2	0.2953287	0	0.2953287
IL15	0.1177028	0	0.1177028
IL15RA	0.264971	0	0
IL16	0.1444371	0	0.1444371
IL17A	0.016008	0	0.016008
IL17B	0.0040427	0	0.0040427
IL17C	0.0021826	0	0.0021826
IL17D	0.0050378	0	0.0050378
IL17F	0.0112657	0	0.0112657
IL17RA	0.0089654	0	0.0089654
IL17RB	0.0147822	0.0147822	0
IL17RC	0.0165514	0.0165514	0

IL17RD	0.0084039	0	0.0084039
IL17RE	0.0518754	0.0518754	0
IL17REL	0.0052804	0	0.0052804
IL18	0.142061	0	0
IL18BP	0.0624916	0	0.0624916
IL18R1	0.0487919	0	0.0487919
IL18RAP	0.0327011	0	0.0327011
IL19	0.1584168	0	0
IL1A	0.2858802	0	0
IL1B	0.1415408	0	0.1415408
IL1F10	0.0473331	0.0473331	0
IL1F5	0.0020717	0.0020717	0
IL1F6	0.0079996	0	0
IL1F7	0.0045434	0	0
IL1F8	0.0029577	0	0
IL1F9	0.0096676	0	0
IL1R1	0.0106478	0	0.0106478
IL1R2	0.0670194	0	0
IL1RAP	0.2277353	0	0.2277353
IL1RAPL1	0.0053895	0	0.0053895
IL1RAPL2	0.0017482	0	0
IL1RL1	0.131419	0	0.131419
IL1RL2	0.0133235	0	0.0133235
IL1RN	0.1002081	0	0
IL2	0.0034767	0	0.0034767
IL20	0.5555918	0	0
IL20RA	0.0170027	0.0170027	0
IL20RB	0.2021097	0	0
IL21	0.0087609	0	0.0087609
IL21R	0.1417374	0	0.1417374
IL22	0.0243427	0	0.0243427
IL22RA1	0.0150229	0.0150229	0
IL22RA2	0.0676142	0	0.0676142
IL23A	0.034886	0	0.034886
IL23R	0.0987763	0	0
IL24	1	0	1
IL25	0.0020958	0	0
IL26	0.0259132	0	0.0259132
IL27	0.0047201	0	0.0047201
IL27RA	0.0163017	0	0.0163017
IL28A	0.0011716	0	0
IL28B	0.001141	0	0
IL28RA	0.018848	0	0
IL29	0.00364	0	0.00364
IL2RA	0.0364204	0	0.0364204

IL2RB	0.0150661	0	0.0150661
IL2RG	0.0288653	0	0.0288653
IL3	0.0058076	0	0
IL31	0.0034417	0	0
IL31RA	0.042151	0	0.042151
IL32	0.0204924	0	0.0204924
IL33	0.0432213	0	0.0432213
IL34	0.0175735	0	0.0175735
IL4	0.0690184	0	0
IL4I1	0.0650357	0	0.0650357
IL4R	0.8077636	0	0.8077636
IL5	0.0155737	0	0
IL5RA	0.0202694	0	0.0202694
IL6	0.3288074	0	0.3288074
IL6R	0.2087096	0	0.2087096
IL6ST	0.9458219	0	0.9458219
IL7	0.2301205	0	0
IL7R	0.4065732	0	0.4065732
IL8	0.1369191	0	0.1369191
IL9	0.0214286	0	0
ILDR1	0.0852792	0.0852792	0
ILDR2	0.0177287	0	0.0177287
ILF2	0.3242124	0	0
ILF3	0.0897168	0.0897168	0
ILK	0.0922242	0	0.0922242
ILKAP	0.007968	0.007968	0
ILVBL	0.0082709	0.0082709	0
IMMP1L	0.0729282	0.0729282	0
IMMP2L	0.0570401	0.0570401	0
IMMT	0.1056866	0.1056866	0
IMP3	0.0941071	0.0941071	0
IMP4	0.0358052	0.0358052	0
IMP5	0.0012051	0.0012051	0
IMPA1	0.025909	0	0
IMPA2	0.0647853	0.0647853	0
IMPACT	0.0126542	0	0
IMPAD1	0.3545729	0	0
IMPDH1	0.0133247	0	0
IMPDH2	0.0492587	0.0492587	0
IMPG1	0.0089252	0	0
IMPG2	0.0159888	0	0.0159888
INA	0.0318795	0	0.0318795
INADL	0.0192806	0.0192806	0
INCA1	0.0054238	0	0
INCENP	0.0627155	0	0

INE1	0.0109726	0.0109726	0
INE2	0.023901	0.023901	0
INF2	0.0074657	0.0074657	0
ING1	0.1883265	0	0
ING2	0.0329446	0.0329446	0
ING3	0.135577	0	0.135577
ING4	0.0480289	0.0480289	0
ING5	0.0062707	0.0062707	0
INGX	0.0027934	0	0.0027934
INHA	0.0031049	0.0031049	0
INHBA	0.0713288	0	0.0713288
INHBB	0.0008682	0	0.0008682
INHBC	0.0146485	0	0
INHBE	0.0353331	0	0.0353331
INMT	0.0023949	0	0.0023949
INO80	0.0869216	0	0.0869216
INO80B	0.1925192	0.1925192	0
INO80C	0.1289103	0.1289103	0
INO80D	0.035056	0	0
INO80E	0.008245	0.008245	0
INPP1	0.0469782	0.0469782	0
INPP4A	0.0280881	0	0.0280881
INPP4B	0.0105228	0.0105228	0
INPP5A	0.0207846	0.0207846	0
INPP5B	0.0103828	0	0.0103828
INPP5D	0.0436538	0	0.0436538
INPP5E	0.0037177	0.0037177	0
INPP5F	0.0324882	0	0.0324882
INPP5J	0.0019084	0.0019084	0
INPP5K	0.0319224	0	0
INPPL1	0.0133677	0	0
INS	0.0004263	0	0.0004263
INSC	0.0321707	0	0
INSIG1	0.1120195	0	0
INSIG2	0.0420013	0	0
INS-IGF2	0.0004263	0	0.0004263
INSL3	0.0016926	0	0
INSL4	0.0115647	0.0115647	0
INSL5	0.0079028	0.0079028	0
INSL6	0.0159304	0	0
INSM1	0.0021211	0	0
INSM2	0.0104116	0	0
INSR	0.0080033	0.0080033	0
INSRR	0.0053577	0	0.0053577
INTS1	0.002925	0.002925	0

INTS10	0.5626456	0	0.5626456
INTS12	0.1150393	0.1150393	0
INTS2	0.0310456	0	0.0310456
INTS3	0.092723	0.092723	0
INTS4	0.1666092	0	0
INTS4L1	2.78E-05	0	0
INTS4L2	1.71E-05	0	0
INTS5	0.0583225	0	0
INTS6	0.0883918	0	0
INTS7	1	0	0
INTS8	0.0454445	0	0
INTS9	0.1372141	0	0.1372141
INTU	0.1498935	0.1498935	0
INVS	0.0829888	0	0.0829888
IP6K1	0.0143413	0	0.0143413
IP6K2	0.0377421	0.0377421	0
IP6K3	0.0331942	0	0.0331942
IPCEF1	0.0165868	0	0.0165868
IPMK	0.3156374	0	0.3156374
IPO11	0.0456291	0	0.0456291
IPO13	0.019908	0	0.019908
IPO4	0.0529255	0.0529255	0
IPO5	0.006621	0	0.006621
IPO7	0.0669699	0	0
IPO8	0.1559368	0	0.1559368
IPO9	0.0829451	0	0
IPP	0.0329681	0	0.0329681
IPPK	0.1205013	0	0
IPW	0.0061513	0	0.0061513
IQCA1	0.0042027	0	0.0042027
IQCB1	0.0790573	0	0
IQCC	0.0143279	0.0143279	0
IQCD	0.1490264	0.1490264	0
IQCE	0.022118	0.022118	0
IQCF1	0.0153738	0.0153738	0
IQCF2	0.0025546	0	0
IQCF3	0.0053466	0	0
IQCF5	0.0053323	0	0
IQCF6	0.0064547	0	0
IQCG	0.2383462	0	0
IQCH	0.0842339	0.0842339	0
IQCJ	0.0081824	0	0
IQCK	0.0107437	0	0
IQGAP1	0.6272492	0	0.6272492
IQGAP2	0.0359493	0	0.0359493

IQGAP3	0.1524184	0.1524184	0
IQSEC1	0.0065618	0	0.0065618
IQSEC2	0.0197066	0	0
IQSEC3	0.005704	0	0.005704
IQUB	0.020556	0	0.020556
IRAK1	0.0030428	0.0030428	0
IRAK1BP1	0.0289797	0	0.0289797
IRAK2	0.0209555	0	0
IRAK3	0.0200135	0	0.0200135
IRAK4	0.6096601	0	0.6096601
IREB2	0.0950869	0	0.0950869
IRF1	0.7933226	0	0.7933226
IRF2	0.2461371	0	0.2461371
IRF2BP1	0.0119409	0.0119409	0
IRF2BP2	1	1	0
IRF3	0.0174165	0.0174165	0
IRF4	0.0700536	0	0.0700536
IRF5	0.0339639	0	0.0339639
IRF6	0.3314553	0.3314553	0
IRF7	0.0322443	0	0
IRF8	0.0508635	0	0.0508635
IRF9	1	0	1
IRGC	0.0054351	0	0
IRGM	0.0177466	0	0.0177466
IRGQ	0.0157391	0.0157391	0
IRS1	0.0214469	0	0
IRS2	0.0872575	0	0
IRS4	0.003029	0	0
IRX1	0.0012424	0	0.0012424
IRX2	0.0265742	0	0.0265742
IRX3	0.0030766	0.0030766	0
IRX4	0.0033486	0	0.0033486
IRX5	0.0180565	0.0180565	0
IRX6	0.003132	0	0.003132
ISCA1	0.0436976	0	0.0436976
ISCA1P1	0.0102613	0	0.0102613
ISCA2	0.0547205	0.0547205	0
ISCU	0.361605	0	0.361605
ISG15	0.0144275	0	0.0144275
ISG20	0.1154056	0	0
ISG20L2	0.0924413	0	0
ISL1	0.0097838	0	0
ISL2	0.0224621	0	0.0224621
ISLR	0.0148756	0	0.0148756
ISLR2	0.0055302	0	0.0055302

ISM1	0.0053303	0	0.0053303
ISM2	0.0030535	0	0
ISOC1	0.0684067	0	0
ISOC2	0.0130985	0.0130985	0
ISPD	0.011271	0	0
ISX	0.0090553	0	0
ISY1	0.0804647	0	0.0804647
ISYNA1	0.0026827	0.0026827	0
ITCH	0.0391208	0	0
ITFG1	0.0571008	0	0
ITFG2	0.030498	0	0
ITFG3	0.0301722	0.0301722	0
ITGA1	0.1227441	0	0.1227441
ITGA10	0.0455908	0	0.0455908
ITGA11	0.0049028	0	0.0049028
ITGA2	0.2407026	0	0
ITGA2B	0.0040307	0	0
ITGA3	0.452304	0.452304	0
ITGA4	0.0288996	0	0.0288996
ITGA5	0.1167959	0	0.1167959
ITGA6	0.1229981	0.1229981	0
ITGA7	0.0371683	0	0.0371683
ITGA8	0.0039733	0	0.0039733
ITGA9	0.0058375	0	0.0058375
ITGAD	0.0077198	0	0.0077198
ITGAE	0.008153	0	0.008153
ITGAL	0.081694	0	0.081694
ITGAM	0.0330043	0	0.0330043
ITGAV	0.0469641	0	0.0469641
ITGAX	0.0106262	0	0.0106262
ITGB1	0.2047065	0	0.2047065
ITGB1BP1	0.0395772	0	0.0395772
ITGB1BP2	0.0081718	0	0.0081718
ITGB1BP3	0.0007013	0	0.0007013
ITGB2	0.0963914	0	0.0963914
ITGB3	0.0230962	0	0.0230962
ITGB3BP	0.0874044	0	0
ITGB4	0.0061393	0.0061393	0
ITGB5	0.0505386	0	0.0505386
ITGB6	0.387462	0.387462	0
ITGB7	0.046452	0	0.046452
ITGB8	0.2011625	0	0
ITGBL1	0.1299345	0	0.1299345
ITIH1	0.0088525	0	0
ITIH2	0.0052103	0.0052103	0

ITIH3	0.0011257	0	0.0011257
ITIH4	0.1150597	0	0
ITIH5	0.0146965	0	0.0146965
ITIH5L	0.0031186	0	0
ITK	0.022666	0	0.022666
ITLN1	0.013339	0	0
ITLN2	0.0196022	0.0196022	0
ITM2A	0.0090625	0	0.0090625
ITM2B	0.0542782	0	0.0542782
ITM2C	0.0033072	0	0
ITPA	0.0109754	0.0109754	0
ITPK1	0.0629252	0.0629252	0
ITPKA	0.0231622	0.0231622	0
ITPKB	0.0765529	0	0.0765529
ITPKC	0.5572178	0	0
ITPR1	0.1215973	0	0.1215973
ITPR2	0.0488054	0	0.0488054
ITPR3	0.0425647	0.0425647	0
ITPRIP	0.244606	0	0.244606
ITPRIPL1	0.0809325	0	0.0809325
ITPRIPL2	0.1376853	0	0.1376853
ITSN1	0.0845038	0	0.0845038
ITSN2	0.0770141	0	0.0770141
IVD	0.0190711	0.0190711	0
IVL	0.0317017	0	0
IVNS1ABP	0.36949	0.36949	0
IWS1	0.2867442	0	0
IYD	0.0052622	0	0
IZUMO1	0.0044505	0	0
JAG1	0.1340978	0	0.1340978
JAG2	0.0004775	0.0004775	0
JAGN1	0.1272232	0.1272232	0
JAK1	0.0312383	0	0.0312383
JAK2	0.1561433	0	0.1561433
JAK3	0.0263754	0	0.0263754
JAKMIP1	0.0023638	0	0.0023638
JAKMIP2	0.0163516	0	0.0163516
JAKMIP3	0.0020607	0	0
JAM2	0.0121271	0	0.0121271
JAM3	0.0042913	0	0.0042913
JARID2	0.1172456	0	0.1172456
JAZF1	0.018114	0	0.018114
JDP2	0.0707622	0	0.0707622
JHDM1D	0.0275727	0	0
JKAMP	0.1877195	0.1877195	0

JMJD1C	0.6645045	0	0.6645045
JMJD4	0.0312812	0.0312812	0
JMJD5	0.0282264	0	0.0282264
JMJD6	0.1230439	0	0
JMJD7-PLA2G	0.0149187	0.0149187	0
JMJD8	0.0073435	0.0073435	0
JMY	0.1232891	0	0.1232891
JOSD1	0.190552	0	0.190552
JOSD2	0.007971	0.007971	0
JPH1	0.0106897	0.0106897	0
JPH2	0.0647135	0	0.0647135
JPH3	0.0009901	0	0.0009901
JPH4	0.0013387	0	0.0013387
JRK	0.0361475	0.0361475	0
JRKL	0.1029931	0.1029931	0
JSRP1	0.0078287	0	0
JTB	0.20603	0.20603	0
JUB	0.0814695	0	0
JUN	0.195994	0	0.195994
JUNB	1	0	0
JUND	0.0637391	0.0637391	0
JUP	0.0407578	0.0407578	0
KAAG1	0.0111444	0.0111444	0
KAL1	0.0077317	0	0.0077317
KALRN	0.0416439	0	0
KANK1	0.0135958	0	0.0135958
KANK2	0.0170388	0	0.0170388
KANK3	0.0013832	0	0.0013832
KANK4	0.0179024	0	0.0179024
KARS	0.0539573	0.0539573	0
KAT2A	0.0067951	0.0067951	0
KAT2B	0.0394157	0	0.0394157
KAT5	0.0559079	0.0559079	0
KATNA1	0.0198977	0	0.0198977
KATNAL1	0.0426112	0	0.0426112
KATNAL2	0.0122701	0	0.0122701
KATNB1	0.027747	0.027747	0
KAZ	0.0164805	0	0.0164805
KAZALD1	0.0582424	0	0
KBTBD10	0.0174562	0	0.0174562
KBTBD11	0.0022865	0.0022865	0
KBTBD12	0.0218852	0.0218852	0
KBTBD13	0.0026955	0	0
KBTBD2	0.2534575	0	0.2534575
KBTBD3	0.0765846	0	0

KBTBD4	0.1112688	0	0.1112688
KBTBD5	0.0065754	0	0.0065754
KBTBD6	0.2373104	0	0.2373104
KBTBD7	0.0278006	0	0
KBTBD8	0.0909408	0	0.0909408
KC6	0.0122741	0	0
KCMF1	0.0726082	0.0726082	0
KCNA1	0.004631	0	0.004631
KCNA10	0.0060787	0	0
KCNA2	0.0050585	0	0.0050585
KCNA3	0.0168533	0	0.0168533
KCNA4	0.0082504	0	0.0082504
KCNA5	0.0043372	0	0.0043372
KCNA6	0.0063712	0	0.0063712
KCNA7	0.0016206	0	0
KCNAB1	0.0314485	0	0.0314485
KCNAB2	0.0183747	0	0.0183747
KCNAB3	0.0069025	0	0.0069025
KCNB1	0.0067329	0	0.0067329
KCNB2	0.0058868	0	0.0058868
KCNC1	0.0018417	0	0.0018417
KCNC2	0.0065027	0	0.0065027
KCNC3	0.0043055	0	0
KCNC4	0.013056	0.013056	0
KCND1	0.0020631	0	0.0020631
KCND2	0.0194509	0	0.0194509
KCND3	0.0082222	0	0.0082222
KCNE1	0.0169775	0	0.0169775
KCNE1L	0.0071518	0	0.0071518
KCNE2	0.0139345	0	0
KCNE3	0.0077179	0	0
KCNE4	0.0082349	0	0.0082349
KCNF1	0.0012931	0	0
KCNG1	0.0037766	0	0.0037766
KCNG2	0.0018323	0	0.0018323
KCNG3	0.0113439	0	0.0113439
KCNG4	0.0036543	0	0
KCNH1	0.0438597	0	0.0438597
KCNH2	0.0012636	0	0
KCNH3	0.0026754	0	0
KCNH4	0.0058098	0	0.0058098
KCNH5	0.1572937	0	0
KCNH6	0.0012769	0	0.0012769
KCNH7	0.0040262	0	0.0040262
KCNH8	0.0071683	0.0071683	0

KCNIP1	0.0093224	0	0.0093224
KCNIP2	0.0145172	0	0.0145172
KCNIP3	0.0031793	0	0.0031793
KCNIP4	0.0080799	0.0080799	0
KCNJ1	0.0160132	0	0.0160132
KCNJ10	0.006922	0	0.006922
KCNJ11	0.0033485	0	0
KCNJ12	0.0010304	0	0.0010304
KCNJ13	0.0261358	0	0
KCNJ14	0.0348289	0.0348289	0
KCNJ15	0.657322	0	0.657322
KCNJ16	0.2265562	0	0
KCNJ2	0.0302572	0	0.0302572
KCNJ3	0.0057355	0	0
KCNJ4	0.0010228	0	0
KCNJ5	0.0137778	0	0.0137778
KCNJ6	0.0066486	0	0.0066486
KCNJ8	0.0122022	0	0.0122022
KCNJ9	0.0090619	0	0
KCNK1	0.4913784	0.4913784	0
KCNK10	0.0051609	0.0051609	0
KCNK12	0.0033616	0	0.0033616
KCNK13	0.0042125	0	0.0042125
KCNK15	0.0666115	0.0666115	0
KCNK16	0.0019156	0	0.0019156
KCNK17	0.0013898	0	0.0013898
KCNK18	0.0024103	0	0
KCNK2	0.0566063	0	0.0566063
KCNK3	0.0043197	0	0
KCNK4	0.008701	0	0.008701
KCNK5	0.0149	0.0149	0
KCNK6	0.0074619	0.0074619	0
KCNK7	0.0046836	0.0046836	0
KCNK9	0.0024672	0	0
KCNMA1	0.0109114	0	0.0109114
KCNMB1	0.0143587	0	0.0143587
KCNMB2	0.0528717	0	0.0528717
KCNMB3	0.0152369	0.0152369	0
KCNMB4	0.02867	0	0.02867
KCNN1	0.004962	0	0.004962
KCNN2	0.0313216	0	0.0313216
KCNN3	0.0678623	0	0.0678623
KCNN4	0.0794961	0.0794961	0
KCNQ1	0.0009083	0.0009083	0
KCNQ1DN	0.0009842	0	0.0009842

KCNQ1OT1	0.0046772	0.0046772	0
KCNQ2	0.022155	0	0
KCNQ3	0.0101528	0	0.0101528
KCNQ4	0.0022286	0	0
KCNQ5	0.0487135	0	0.0487135
KCNRG	0.0112196	0	0
KCNS1	0.0134066	0	0.0134066
KCNS2	0.002261	0	0.002261
KCNS3	0.010295	0	0.010295
KCNT1	0.0008136	0	0.0008136
KCNT2	0.0184257	0	0.0184257
KCNU1	0.0072015	0	0
KCNV1	0.0094481	0	0
KCNV2	0.0134452	0.0134452	0
KCP	0.0017355	0.0017355	0
KCTD1	0.1482047	0.1482047	0
KCTD10	0.0258474	0	0.0258474
KCTD11	0.0137633	0	0
KCTD12	0.0344385	0	0.0344385
KCTD13	0.0157791	0.0157791	0
KCTD14	0.0244964	0.0244964	0
KCTD15	0.0043945	0	0.0043945
KCTD16	0.1046985	0	0.1046985
KCTD17	0.0022849	0	0.0022849
KCTD18	0.0142891	0	0
KCTD19	0.0095654	0	0
KCTD2	0.0366622	0	0.0366622
KCTD20	0.0971782	0	0.0971782
KCTD21	0.0381065	0	0.0381065
KCTD3	0.196344	0.196344	0
KCTD4	0.1284216	0	0
KCTD5	0.0078342	0.0078342	0
KCTD6	0.0566282	0	0
KCTD7	0.0160744	0	0.0160744
KCTD8	0.0049314	0	0.0049314
KCTD9	0.1034603	0	0.1034603
KDELC1	0.0346878	0	0.0346878
KDELC2	0.0257872	0	0.0257872
KDELR1	0.0072691	0.0072691	0
KDELR2	0.0206181	0.0206181	0
KDELR3	0.0014113	0.0014113	0
KDM1A	0.0798942	0.0798942	0
KDM1B	0.089951	0	0.089951
KDM2A	0.0367736	0	0.0367736
KDM2B	0.0222077	0	0.0222077

KDM3A	0.126039	0.126039	0
KDM3B	0.0528844	0.0528844	0
KDM4A	0.0968556	0	0.0968556
KDM4B	0.013162	0.013162	0
KDM4C	0.1612268	0	0
KDM4D	0.0568949	0	0.0568949
KDM4DL	0.0044737	0	0.0044737
KDM5A	0.059229	0	0.059229
KDM5B	0.1254393	0.1254393	0
KDM5C	0.0359941	0	0
KDM5D	1.53E-05	0	0
KDM6A	0.0401086	0	0.0401086
KDM6B	0.146082	0	0
KDR	0.0032855	0	0.0032855
KDSR	0.1105899	0	0.1105899
KEAP1	0.0101801	0.0101801	0
KEL	0.0206745	0	0.0206745
KERA	0.0212279	0	0.0212279
KGFLP2	1.56E-05	0	0
KHDC1	0.0117861	0	0
KHDC1L	0.0169094	0	0
KHDRBS1	0.0985532	0	0
KHDRBS2	0.0058167	0	0.0058167
KHDRBS3	0.0233041	0	0
KHK	0.0078706	0	0
KHNYN	0.0657373	0.0657373	0
KHSRP	0.009105	0.009105	0
KIAA0020	0.1045712	0	0.1045712
KIAA0040	0.2386137	0	0.2386137
KIAA0087	0.0153889	0	0.0153889
KIAA0090	0.0127442	0	0.0127442
KIAA0100	0.0368963	0	0.0368963
KIAA0101	0.0589164	0.0589164	0
KIAA0114	0.0374157	0.0374157	0
KIAA0125	0.0001491	0	0.0001491
KIAA0141	0.0222776	0.0222776	0
KIAA0146	0.0193416	0	0
KIAA0174	0.0636839	0	0
KIAA0182	0.029816	0.029816	0
KIAA0195	0.030489	0.030489	0
KIAA0196	0.0701114	0	0.0701114
KIAA0226	0.0636398	0	0.0636398
KIAA0232	0.0141093	0	0
KIAA0240	0.0073522	0	0.0073522
KIAA0247	0.1632765	0	0.1632765

KIAA0284	0.0011512	0.0011512	0
KIAA0317	0.043554	0	0
KIAA0319	0.0333249	0	0.0333249
KIAA0319L	0.0253267	0.0253267	0
KIAA0355	0.0155364	0	0.0155364
KIAA0368	0.0443487	0	0
KIAA0391	0.1111289	0	0.1111289
KIAA0406	0.0983485	0.0983485	0
KIAA0408	0.0146513	0	0.0146513
KIAA0415	0.0097258	0.0097258	0
KIAA0427	0.0192539	0	0.0192539
KIAA0430	0.4422896	0	0.4422896
KIAA0467	0.0100316	0	0
KIAA0494	0.0724454	0	0.0724454
KIAA0495	0.0016441	0	0.0016441
KIAA0513	0.0438631	0	0
KIAA0528	0.0536724	0	0
KIAA0556	0.0239623	0.0239623	0
KIAA0562	0.0077529	0	0.0077529
KIAA0564	0.0581504	0	0
KIAA0586	0.7190598	0	0.7190598
KIAA0649	0.0086978	0.0086978	0
KIAA0652	0.0421425	0.0421425	0
KIAA0664	0.0080777	0.0080777	0
KIAA0748	0.0693286	0	0.0693286
KIAA0753	0.0787389	0.0787389	0
KIAA0754	0.0375566	0	0.0375566
KIAA0776	0.0423983	0	0.0423983
KIAA0802	0.007374	0	0
KIAA0831	0.0527707	0	0
KIAA0892	0.0162769	0	0
KIAA0895	0.1540188	0.1540188	0
KIAA0895L	0.0138742	0.0138742	0
KIAA0907	0.2580306	0.2580306	0
KIAA0913	0.0224643	0	0
KIAA0922	0.0618385	0	0.0618385
KIAA0947	0.1555079	0	0.1555079
KIAA1009	0.1861797	0	0.1861797
KIAA1012	0.035234	0	0.035234
KIAA1024	0.003434	0	0.003434
KIAA1033	0.1455151	0	0.1455151
KIAA1045	0.022805	0	0.022805
KIAA1107	0.0080527	0.0080527	0
KIAA1109	0.0088319	0	0.0088319
KIAA1143	0.0440668	0	0.0440668

KIAA1147	0.0195781	0	0
KIAA1161	0.0100128	0.0100128	0
KIAA1191	0.0203303	0.0203303	0
KIAA1199	0.0251487	0	0.0251487
KIAA1210	0.0030376	0	0.0030376
KIAA1211	0.0074719	0	0
KIAA1217	0.0220597	0	0.0220597
KIAA1239	0.00988	0	0.00988
KIAA1244	0.0067938	0.0067938	0
KIAA1257	0.0048273	0.0048273	0
KIAA1267	0.3174399	0	0
KIAA1274	0.0089754	0	0.0089754
KIAA1279	0.0748909	0	0.0748909
KIAA1310	0.0563045	0	0.0563045
KIAA1324	0.0225895	0	0
KIAA1324L	0.0175507	0	0.0175507
KIAA1328	0.0471027	0	0.0471027
KIAA1370	0.0790653	0	0.0790653
KIAA1377	0.0389048	0	0
KIAA1383	0.0289331	0.0289331	0
KIAA1407	0.0633855	0.0633855	0
KIAA1409	0.0655587	0	0.0655587
KIAA1429	0.0829899	0	0.0829899
KIAA1430	0.0932747	0	0.0932747
KIAA1432	0.3589564	0	0.3589564
KIAA1462	0.0052807	0	0.0052807
KIAA1467	0.0298071	0	0.0298071
KIAA1468	0.0909649	0	0
KIAA1486	0.0049995	0	0.0049995
KIAA1522	0.0034433	0.0034433	0
KIAA1524	0.1229745	0	0
KIAA1529	0.0106712	0	0.0106712
KIAA1530	0.0184604	0.0184604	0
KIAA1539	0.0218893	0	0
KIAA1543	0.001725	0.001725	0
KIAA1549	0.0103934	0.0103934	0
KIAA1586	0.050831	0	0.050831
KIAA1598	0.0157031	0.0157031	0
KIAA1609	0.0657416	0.0657416	0
KIAA1614	0.0111525	0	0.0111525
KIAA1632	0.3784642	0	0.3784642
KIAA1644	0.0015337	0	0.0015337
KIAA1671	0.004489	0.004489	0
KIAA1683	0.0286841	0	0
KIAA1704	0.0158136	0	0

KIAA1712	0.1030815	0.1030815	0
KIAA1715	0.0197466	0	0
KIAA1731	0.1552719	0	0
KIAA1737	0.093922	0.093922	0
KIAA1751	0.0011133	0	0
KIAA1755	0.0045141	0	0.0045141
KIAA1797	0.0253234	0	0
KIAA1804	0.0391723	0.0391723	0
KIAA1826	0.0622023	0	0.0622023
KIAA1841	0.0263945	0.0263945	0
KIAA1875	0.0028771	0.0028771	0
KIAA1908	0.031919	0	0.031919
KIAA1919	0.1119441	0	0.1119441
KIAA1958	0.0368055	0	0
KIAA1967	0.0347448	0	0
KIAA1984	0.0129018	0.0129018	0
KIAA2013	0.0121568	0.0121568	0
KIAA2018	0.0435308	0	0.0435308
KIAA2022	0.0098601	0	0.0098601
KIAA2026	0.2668224	0	0.2668224
KIDINS220	0.0116388	0	0.0116388
KIF11	0.119213	0.119213	0
KIF12	0.0022007	0.0022007	0
KIF13A	0.0740391	0	0.0740391
KIF13B	0.0352805	0.0352805	0
KIF14	0.290808	0.290808	0
KIF15	0.0429592	0.0429592	0
KIF16B	0.0284101	0	0
KIF17	0.00077	0	0.00077
KIF18A	0.2097737	0.2097737	0
KIF18B	0.0394961	0.0394961	0
KIF19	0.0016383	0	0.0016383
KIF1A	0.0007075	0	0.0007075
KIF1B	0.0276964	0	0.0276964
KIF1C	0.0050585	0	0
KIF20A	0.9281095	0.9281095	0
KIF20B	0.1340423	0	0
KIF21A	0.0647468	0.0647468	0
KIF21B	0.0920957	0	0.0920957
KIF22	0.0273443	0.0273443	0
KIF23	0.1055146	0.1055146	0
KIF24	0.1158098	0.1158098	0
KIF25	0.0072027	0	0
KIF26A	0.0005353	0	0.0005353
KIF26B	0.0240351	0	0.0240351

KIF27	0.0221827	0	0
KIF2A	0.0853111	0	0.0853111
KIF2B	0.0033272	0.0033272	0
KIF2C	0.2433602	0.2433602	0
KIF3A	0.0516483	0	0.0516483
KIF3B	0.0615517	0.0615517	0
KIF3C	0.0154902	0	0.0154902
KIF4A	0.0306431	0.0306431	0
KIF4B	0.0108465	0	0
KIF5A	0.0182985	0	0.0182985
KIF5B	0.0704558	0	0
KIF5C	0.0051919	0	0.0051919
KIF6	0.0123373	0	0.0123373
KIF7	0.0032069	0	0.0032069
KIF9	0.0439133	0.0439133	0
KIFAP3	0.1827408	0	0.1827408
KIFC2	0.0038214	0.0038214	0
KIFC3	0.0797712	0	0
KILLIN	0.2904681	0	0.2904681
KIN	0.036458	0	0
KIR2DL1	0.0051023	0	0.0051023
KIR2DL3	0.00432	0	0.00432
KIR2DS4	0.0010397	0	0.0010397
KIR3DL1	0.0026762	0	0.0026762
KIR3DL2	0.0053477	0	0.0053477
KIR3DL3	0.004223	0	0.004223
KIR3DX1	0.0049397	0	0.0049397
KIRREL	0.0165248	0	0.0165248
KIRREL2	0.0012671	0	0
KIRREL3	0.0017528	0	0.0017528
KISS1	0.0281887	0.0281887	0
KISS1R	0.0200093	0.0200093	0
KIT	0.0115303	0	0.0115303
KITLG	0.0271671	0	0.0271671
KL	0.00992	0	0.00992
KLB	0.0065409	0	0.0065409
KLC1	0.0280804	0	0
KLC2	0.0075792	0.0075792	0
KLC3	0.0022294	0.0022294	0
KLC4	0.0197371	0.0197371	0
KLF1	0.0167372	0	0.0167372
KLF10	0.3036474	0	0.3036474
KLF11	0.0240271	0	0.0240271
KLF12	0.0590878	0	0.0590878
KLF13	0.0227344	0	0.0227344

KLF14	0.0128412	0	0.0128412
KLF15	0.0023201	0	0.0023201
KLF16	0.015273	0.015273	0
KLF17	0.005679	0	0.005679
KLF2	0.0335468	0	0
KLF3	0.0839568	0.0839568	0
KLF4	0.0245977	0.0245977	0
KLF5	0.9981589	0.9981589	0
KLF6	0.8528442	0	0.8528442
KLF7	0.1195607	0	0.1195607
KLF8	0.0060289	0	0.0060289
KLF9	0.6477364	0	0.6477364
KLHDC1	0.0440296	0	0.0440296
KLHDC10	0.0645055	0	0
KLHDC2	0.0235721	0.0235721	0
KLHDC3	0.0164242	0	0
KLHDC4	0.0321236	0.0321236	0
KLHDC5	0.0237001	0	0.0237001
KLHDC7A	0.004594	0.004594	0
KLHDC7B	1	0	1
KLHDC8A	0.0265588	0	0.0265588
KLHDC8B	0.0046051	0	0.0046051
KLHDC9	0.1324955	0.1324955	0
KLHL1	0.0051338	0	0.0051338
KLHL10	0.0060034	0	0.0060034
KLHL11	0.0078087	0	0.0078087
KLHL12	0.1366837	0.1366837	0
KLHL13	0.0232215	0	0.0232215
KLHL14	0.005329	0	0.005329
KLHL15	0.0268908	0	0.0268908
KLHL17	0.0026785	0.0026785	0
KLHL18	0.0506775	0	0.0506775
KLHL2	0.0783812	0	0.0783812
KLHL20	0.1529837	0	0.1529837
KLHL21	0.0560646	0	0
KLHL22	0.0056909	0	0.0056909
KLHL23	0.0162514	0.0162514	0
KLHL24	0.0419482	0	0.0419482
KLHL25	0.0145686	0.0145686	0
KLHL26	0.0043367	0	0
KLHL28	0.1727337	0	0.1727337
KLHL29	0.1514898	0	0.1514898
KLHL3	0.0498323	0	0
KLHL30	0.0012421	0.0012421	0
KLHL31	0.0189061	0.0189061	0

KLHL32	0.0234111	0	0.0234111
KLHL33	0.0092859	0	0.0092859
KLHL34	0.00339	0	0.00339
KLHL35	0.0047224	0.0047224	0
KLHL36	0.0088258	0	0
KLHL38	0.098219	0	0.098219
KLHL4	0.0341625	0	0.0341625
KLHL5	0.4458948	0	0.4458948
KLHL6	0.064628	0	0.064628
KLHL7	0.0578735	0	0.0578735
KLHL8	0.0447117	0	0.0447117
KLHL9	0.0526348	0	0.0526348
KLK1	0.0050929	0	0
KLK10	0.0012398	0	0
KLK11	0.0008767	0.0008767	0
KLK12	0.0008983	0.0008983	0
KLK13	0.0017956	0	0
KLK14	0.0009525	0	0.0009525
KLK15	0.0025014	0.0025014	0
KLK2	0.0052947	0	0.0052947
KLK3	0.0032301	0.0032301	0
KLK4	0.0033839	0	0.0033839
KLK5	0.0011806	0	0.0011806
KLK6	0.0013878	0	0
KLK7	0.0018035	0	0
KLK8	0.002096	0	0
KLK9	0.0010891	0.0010891	0
KLKB1	0.0049309	0	0
KLKP1	0.0410359	0	0.0410359
KLRA1	0.0216799	0	0
KLRAQ1	0.0823797	0	0
KLRB1	0.0045724	0	0.0045724
KLRC1	0.2221674	0	0.2221674
KLRC2	0.0185751	0	0.0185751
KLRC3	0.0106896	0	0.0106896
KLRC4	0.0511554	0	0.0511554
KLRD1	0.1219696	0	0.1219696
KLRF1	0.0165131	0	0.0165131
KLRG1	0.0151169	0	0.0151169
KLRG2	0.002167	0	0
KLRK1	0.4966205	0	0.4966205
KMO	0.2218368	0	0.2218368
KNCN	0.0035818	0	0
KNDC1	0.0016893	0	0
KNG1	0.0070899	0	0

KNTC1	0.2254447	0	0
KPNA1	0.0768857	0	0.0768857
KPNA3	0.0358374	0	0.0358374
KPNA4	0.122019	0	0.122019
KPNA5	0.0241604	0	0.0241604
KPNA6	0.0560281	0	0.0560281
KPNA7	0.0382516	0.0382516	0
KPNB1	0.0774275	0	0
KPRP	0.0296332	0	0
KPTN	0.0066969	0.0066969	0
KRAS	0.1017473	0.1017473	0
KRBA1	0.00621	0	0.00621
KRBA2	0.3805883	0	0.3805883
KRCC1	0.0624237	0	0
KREMEN1	0.0127883	0	0.0127883
KREMEN2	0.0027014	0.0027014	0
KRI1	0.0771102	0.0771102	0
KRIT1	0.0769559	0	0
KRR1	0.0889113	0	0
KRT1	0.0177778	0	0.0177778
KRT10	0.1306568	0.1306568	0
KRT12	0.013376	0	0
KRT13	0.0060073	0	0
KRT14	0.0617715	0	0
KRT15	0.0344968	0.0344968	0
KRT16	0.0413484	0	0
KRT17	0.3322491	0	0
KRT18	0.0477177	0.0477177	0
KRT19	0.0391738	0.0391738	0
KRT2	0.0049192	0	0.0049192
KRT20	0.009117	0	0
KRT222	0.0146144	0	0.0146144
KRT23	0.0112887	0	0.0112887
KRT24	0.0254467	0	0.0254467
KRT25	0.0131388	0	0.0131388
KRT26	0.013886	0.013886	0
KRT27	0.0131859	0	0.0131859
KRT28	0.0197213	0	0
KRT3	0.0042289	0	0
KRT31	0.0248134	0	0
KRT32	0.0086143	0	0
KRT33A	0.009739	0	0
KRT33B	0.0072319	0	0
KRT34	0.0523198	0	0.0523198
KRT35	0.0039296	0	0

KRT36	0.0079763	0	0.0079763
KRT37	0.01211	0	0
KRT38	0.0072467	0	0.0072467
KRT4	0.0035389	0	0
KRT5	0.3011634	0	0.3011634
KRT6A	0.3437763	0	0
KRT6B	0.1692482	0	0
KRT6C	0.0206099	0	0
KRT7	0.2844533	0.2844533	0
KRT71	0.002998	0	0.002998
KRT72	0.0079181	0	0.0079181
KRT73	0.0047575	0	0.0047575
KRT74	0.0121936	0	0
KRT75	0.1519312	0	0.1519312
KRT76	0.0060252	0	0
KRT77	0.0043344	0	0
KRT78	0.0043801	0	0
KRT79	0.0035427	0	0
KRT8	0.0445648	0.0445648	0
KRT80	0.1534128	0.1534128	0
KRT81	0.0176018	0	0.0176018
KRT82	0.0034478	0	0.0034478
KRT83	0.0162789	0	0
KRT84	0.0090637	0	0
KRT85	0.011946	0	0
KRT86	0.0072816	0	0
KRT9	0.0085875	0	0
KRTAP10-1	0.0018374	0.0018374	0
KRTAP10-10	0.0012216	0	0
KRTAP10-11	0.001013	0	0
KRTAP10-12	0.0018865	0	0
KRTAP10-2	0.0022919	0	0
KRTAP10-3	0.0034383	0.0034383	0
KRTAP10-4	0.0026391	0.0026391	0
KRTAP10-5	0.0019922	0	0
KRTAP10-6	0.0015409	0	0
KRTAP10-7	0.0022308	0	0
KRTAP10-8	0.0025951	0	0
KRTAP10-9	0.0023246	0.0023246	0
KRTAP11-1	0.0076826	0	0
KRTAP12-1	0.0043656	0	0
KRTAP12-2	0.0059879	0	0
KRTAP12-3	0.0033497	0	0
KRTAP12-4	0.0022964	0	0
KRTAP13-1	0.004897	0	0

KRTAP13-2	0.0034906	0	0
KRTAP13-3	0.006968	0	0
KRTAP13-4	0.0064299	0	0
KRTAP15-1	0.005655	0	0
KRTAP17-1	0.007707	0	0
KRTAP19-1	0.0080114	0	0
KRTAP19-2	0.006971	0	0
KRTAP19-3	0.0085835	0	0
KRTAP19-4	0.005407	0	0
KRTAP19-5	0.0053424	0	0
KRTAP19-6	0.0060494	0	0
KRTAP19-7	0.0051171	0	0
KRTAP19-8	0.0120071	0.0120071	0
KRTAP20-1	0.0078927	0	0
KRTAP20-2	0.0039842	0	0
KRTAP20-3	0.0047054	0	0
KRTAP20-4	0.0082617	0	0
KRTAP21-1	0.0084571	0	0
KRTAP21-2	0.0078009	0	0
KRTAP2-2	0.0071479	0	0.0071479
KRTAP22-1	0.0052921	0	0
KRTAP23-1	0.0092418	0	0
KRTAP24-1	0.0061998	0	0
KRTAP25-1	0.008308	0	0
KRTAP26-1	0.0082618	0	0
KRTAP27-1	0.0087532	0	0
KRTAP4-1	0.0061107	0	0
KRTAP4-2	0.0093311	0	0.0093311
KRTAP4-3	0.0069896	0	0
KRTAP4-4	0.0092565	0	0.0092565
KRTAP4-5	0.0128061	0	0
KRTAP4-7	0.0059746	0.0059746	0
KRTAP4-8	0.0053499	0.0053499	0
KRTAP4-9	0.0054878	0	0
KRTAP5-1	0.0013885	0.0013885	0
KRTAP5-10	0.0059703	0.0059703	0
KRTAP5-11	0.0030266	0.0030266	0
KRTAP5-2	0.0031749	0	0
KRTAP5-3	0.0023728	0	0
KRTAP5-4	0.0011326	0.0011326	0
KRTAP5-5	0.0010407	0.0010407	0
KRTAP5-6	0.0027439	0.0027439	0
KRTAP5-7	0.0037688	0	0
KRTAP5-8	0.0087654	0	0
KRTAP5-9	0.0078841	0.0078841	0

KRTAP6-1	0.0082266	0	0
KRTAP6-2	0.0060843	0	0
KRTAP6-3	0.006924	0.006924	0
KRTAP7-1	0.0049302	0	0
KRTAP8-1	0.0038615	0	0
KRTAP9-2	0.0028788	0	0
KRTAP9-4	0.0040857	0	0
KRTAP9-8	0.0018928	0.0018928	0
KRTAP9-9	0.0060658	0	0
KRTCAP2	0.0510618	0.0510618	0
KRTCAP3	0.0063125	0.0063125	0
KRTDAP	0.0085057	0.0085057	0
KSR1	0.7949605	0	0.7949605
KSR2	0.0045147	0	0
KTELC1	0.065217	0	0.065217
KTI12	0.0595849	0	0.0595849
KTN1	0.1047127	0	0
KY	0.0029909	0.0029909	0
KYNU	1	0	1
L1CAM	0.0006907	0	0.0006907
L1TD1	0.0027466	0	0.0027466
L2HGDH	0.2432751	0.2432751	0
L3MBTL	0.0690789	0.0690789	0
L3MBTL2	0.008456	0	0
L3MBTL3	0.0241187	0	0.0241187
L3MBTL4	0.0057738	0	0.0057738
LACE1	0.0469619	0	0.0469619
LACRT	0.0046644	0.0046644	0
LACTB	0.036969	0	0.036969
LACTB2	0.0829679	0.0829679	0
LAD1	0.0322556	0.0322556	0
LAG3	0.0291627	0	0.0291627
LAGE3	0.0024033	0.0024033	0
LAIR1	0.0026586	0	0.0026586
LAIR2	0.0011934	0	0.0011934
LALBA	0.0057074	0	0
LAMA1	0.0033474	0	0.0033474
LAMA2	0.0061817	0	0.0061817
LAMA3	0.0960127	0	0
LAMA4	0.0335916	0	0.0335916
LAMA5	0.1185043	0.1185043	0
LAMB1	0.1161553	0	0.1161553
LAMB2	0.0020302	0	0.0020302
LAMB2L	0.0026053	0	0
LAMB3	0.595945	0.595945	0

LAMB4	0.0354874	0	0.0354874
LAMC1	0.4995604	0	0.4995604
LAMC2	0.5019001	0	0
LAMC3	0.0038517	0	0.0038517
LAMP1	0.0173787	0	0
LAMP2	0.022438	0	0.022438
LAMP3	0.0280677	0	0.0280677
LANCL1	0.0328418	0	0.0328418
LANCL2	0.0306862	0	0
LANCL3	0.0037247	0.0037247	0
LAP3	0.0323455	0	0.0323455
LAPTM4A	0.6641194	0	0.6641194
LAPTM4B	0.0143403	0.0143403	0
LAPTM5	0.1040771	0	0.1040771
LARGE	0.0064187	0	0.0064187
LARP1	0.0266679	0.0266679	0
LARP1B	0.0067909	0.0067909	0
LARP4	0.1471108	0	0
LARP4B	0.0091022	0.0091022	0
LARP6	0.0472063	0	0.0472063
LARP7	0.0649043	0	0
LARS	0.0735491	0	0
LARS2	0.026295	0	0.026295
LAS1L	0.0263353	0.0263353	0
LASP1	0.1244288	0.1244288	0
LASS1	0.0005462	0	0.0005462
LASS2	0.0374658	0.0374658	0
LASS3	0.0039515	0	0.0039515
LASS4	0.003654	0	0.003654
LASS5	0.0784475	0	0
LASS6	0.0214972	0	0
LAT	0.0021533	0	0.0021533
LAT2	0.0407265	0	0.0407265
LATS1	0.0394395	0	0.0394395
LATS2	0.1433606	0	0.1433606
LAX1	0.105335	0	0.105335
LAYN	0.0082328	0	0.0082328
LBH	0.0262133	0	0.0262133
LBP	0.005793	0	0.005793
LBR	0.1164788	0	0.1164788
LBX1	0.0009202	0.0009202	0
LBX2	0.0095049	0.0095049	0
LBXCOR1	0.0016902	0.0016902	0
LCA5	0.1490095	0	0.1490095
LCA5L	0.0392219	0.0392219	0

LCAT	0.0037734	0	0
LCE1A	0.0044927	0	0
LCE1B	0.0375006	0.0375006	0
LCE1C	0.0395493	0	0
LCE1D	0.003596	0	0
LCE1E	0.0057736	0.0057736	0
LCE1F	0.0204553	0	0
LCE2A	0.0098276	0	0
LCE2B	0.0056811	0.0056811	0
LCE2C	0.0134817	0	0
LCE2D	0.0232917	0	0
LCE3A	0.0088727	0	0
LCE3B	0.0016433	0	0
LCE3C	0.0008929	0	0
LCE3D	0.0614276	0	0
LCE3E	0.0336108	0	0
LCE4A	0.0664748	0	0
LCE5A	0.0636847	0	0
LCE6A	0.0070865	0	0
LCK	0.0387866	0	0.0387866
LCLAT1	0.0242216	0.0242216	0
LCMT1	0.058421	0.058421	0
LCMT2	0.0934763	0	0.0934763
LCN1	0.0024041	0	0
LCN10	0.0014474	0	0.0014474
LCN12	0.0035579	0.0035579	0
LCN15	0.0032436	0	0
LCN2	0.3099553	0.3099553	0
LCN6	0.0014647	0	0.0014647
LCN8	0.0041782	0	0.0041782
LCN9	0.0010289	0	0
LCNL1	0.004822	0	0.004822
LCOR	0.0585734	0	0
LCORL	0.0420391	0	0
LCP1	0.0860567	0	0.0860567
LCP2	0.0472641	0	0.0472641
LCT	0.0054565	0	0.0054565
LCTL	0.0044516	0	0.0044516
LDB1	0.0196024	0	0.0196024
LDB2	0.003136	0	0.003136
LDB3	0.0019948	0	0.0019948
LDHA	0.5668541	0.5668541	0
LDHAL6A	0.0089963	0	0
LDHAL6B	0.043726	0	0
LDHB	0.0438877	0	0.0438877

LDHC	0.0156801	0	0
LDHD	0.0087523	0.0087523	0
LDLR	1	1	0
LDLRAD1	0.0062614	0.0062614	0
LDLRAD2	0.004118	0	0.004118
LDLRAD3	0.0196361	0	0.0196361
LDLRAP1	0.0113009	0	0.0113009
LDOC1	0.0031597	0	0
LDOC1L	0.0040665	0	0.0040665
LEAP2	0.0845781	0.0845781	0
LECT1	0.0033165	0	0.0033165
LECT2	0.032646	0.032646	0
LEF1	0.0113155	0	0.0113155
LEFTY1	0.0193497	0	0
LEFTY2	0.0144287	0	0.0144287
LEKR1	0.3168446	0.3168446	0
LELP1	0.0086294	0	0
LEMD1	0.0359039	0.0359039	0
LEMD2	0.0174448	0.0174448	0
LEMD3	0.149283	0	0.149283
LENEP	0.0504445	0.0504445	0
LENG1	0.0070569	0.0070569	0
LENG8	0.0177175	0.0177175	0
LENG9	0.2980414	0.2980414	0
LEO1	0.048733	0.048733	0
LEP	0.0062825	0	0.0062825
LEPR	0.5997096	0	0.5997096
LEPRE1	0.0241129	0	0.0241129
LEPREL1	0.0822194	0	0
LEPREL2	0.0042412	0	0.0042412
LEPROT	0.5997096	0	0.5997096
LEPROTL1	0.0512906	0	0.0512906
LETM1	0.0045786	0.0045786	0
LETM2	0.1035106	0.1035106	0
LETMD1	0.1065666	0.1065666	0
LEUTX	0.0109527	0	0
LFNG	0.0034223	0.0034223	0
LGALS1	0.0120309	0	0.0120309
LGALS12	0.0054525	0	0.0054525
LGALS13	0.0086642	0	0.0086642
LGALS14	0.0684996	0	0.0684996
LGALS2	0.003645	0	0.003645
LGALS3	0.1006908	0.1006908	0
LGALS3BP	0.0224166	0.0224166	0
LGALS4	0.0006682	0.0006682	0

LGALS7	0.0097073	0.0097073	0
LGALS7B	0.0024527	0.0024527	0
LGALS8	0.1650245	0	0
LGALS9	0.0079187	0	0
LGALS9B	2.16E-05	2.16E-05	0
LGALS9C	2.89E-05	0	0
LGI1	0.0167644	0	0.0167644
LGI2	0.002611	0	0.002611
LGI3	0.0038411	0	0.0038411
LGI4	0.0041917	0	0.0041917
LGMN	0.043218	0	0.043218
LGR4	0.295047	0.295047	0
LGR5	0.0080248	0	0
LGR6	0.0195275	0	0
LGSN	0.0096099	0	0.0096099
LGTN	0.1388842	0	0
LHB	0.001316	0.001316	0
LHCGR	0.0049444	0	0.0049444
LHFP	0.0101663	0	0.0101663
LHFPL1	0.0197755	0	0.0197755
LHFPL2	0.0571123	0	0.0571123
LHFPL3	0.005979	0.005979	0
LHFPL4	0.0018003	0	0
LHFPL5	0.0617512	0.0617512	0
LHPP	0.0075787	0	0
LHX1	0.0021448	0	0
LHX2	0.0558343	0	0.0558343
LHX3	0.0009951	0	0
LHX4	0.0125364	0	0
LHX5	0.0009593	0.0009593	0
LHX6	0.0037555	0	0.0037555
LHX8	0.0020261	0	0
LHX9	0.0116024	0	0
LIAS	0.1251402	0.1251402	0
LIF	0.0075409	0	0
LIFR	0.133152	0	0.133152
LIG1	0.0131494	0.0131494	0
LIG3	0.0336286	0.0336286	0
LIG4	0.0788923	0	0.0788923
LILRA1	0.0016246	0	0.0016246
LILRA2	0.0009008	0	0.0009008
LILRA3	0.0009809	0	0.0009809
LILRA4	0.0031474	0	0.0031474
LILRA5	0.0017397	0	0.0017397
LILRA6	0.000746	0	0.000746

LILRB1	0.0606595	0	0.0606595
LILRB2	0.0009114	0	0.0009114
LILRB3	0.0016914	0	0.0016914
LILRB4	0.0090106	0	0.0090106
LILRB5	0.001493	0	0.001493
LILRP2	0.0019098	0	0
LIM2	0.007173	0	0.007173
LIMA1	1	0	0
LIMCH1	0.0275308	0	0
LIMD1	0.019416	0	0
LIMD2	0.0053947	0	0.0053947
LIME1	0.0107573	0.0107573	0
LIMK1	0.0065284	0	0.0065284
LIMK2	0.1114673	0	0.1114673
LIMS1	0.0875432	0	0.0875432
LIMS2	0.0017812	0	0.0017812
LIMS3-LOC44(	1.53E-05	0	1.53E-05
LIN28A	0.0066884	0	0
LIN28B	0.009231	0	0
LIN37	0.243156	0.243156	0
LIN52	0.0387529	0	0.0387529
LIN54	0.0939517	0	0
LIN7A	0.0116925	0	0.0116925
LIN7B	0.0094443	0.0094443	0
LIN7C	0.2044724	0.2044724	0
LIN9	0.0931717	0.0931717	0
LINGO1	0.0027417	0	0.0027417
LINGO2	0.0106009	0	0.0106009
LINGO3	0.0007995	0	0.0007995
LINGO4	0.0078884	0	0.0078884
LINS1	0.7091558	0	0.7091558
LIPA	0.0902601	0	0.0902601
LIPC	0.044143	0	0.044143
LIPE	0.0140481	0	0
LIPF	0.0222869	0	0
LIPG	0.0154258	0	0.0154258
LIPH	0.1658876	0.1658876	0
LIPI	0.0059272	0	0
LIPJ	0.0242712	0	0
LIPK	0.0137948	0	0
LIPM	0.038342	0.038342	0
LIPN	0.0487698	0	0.0487698
LIPT1	0.0809414	0	0
LIPT2	0.0368349	0.0368349	0
LITAF	0.0662818	0	0.0662818

LIX1	0.0231676	0	0.0231676
LIX1L	0.1653521	0	0.1653521
LLGL1	0.0022757	0	0.0022757
LLGL2	0.0092574	0.0092574	0
LLPH	0.0511036	0	0
LMAN1	0.070062	0	0.070062
LMAN1L	0.0129602	0	0
LMAN2	0.0361452	0.0361452	0
LMAN2L	0.0250518	0.0250518	0
LMBR1	0.0528377	0	0
LMBR1L	0.0165716	0	0
LMBRD1	0.0515296	0	0.0515296
LMBRD2	0.1279501	0	0.1279501
LMCD1	0.0261723	0	0.0261723
LMF1	0.0028728	0.0028728	0
LMF2	0.0024537	0.0024537	0
LMLN	0.1401802	0	0
LMNA	0.7974085	0.7974085	0
LMNB1	0.5999644	0	0
LMNB2	0.0072602	0.0072602	0
LMO1	0.0107064	0	0.0107064
LMO2	0.0327755	0	0.0327755
LMO3	0.0172349	0	0.0172349
LMO4	0.0653429	0	0
LMO7	0.2585347	0.2585347	0
LMOD1	0.0508029	0	0.0508029
LMOD2	0.0114629	0	0
LMOD3	0.0176496	0	0.0176496
LMTK2	0.0235651	0.0235651	0
LMTK3	0.0060317	0.0060317	0
LMX1A	0.1179946	0	0.1179946
LMX1B	0.0014203	0	0
LNP1	0.093621	0	0.093621
LNPEP	0.2184472	0	0.2184472
LNX1	0.0437602	0.0437602	0
LNX2	0.0455603	0.0455603	0
LOC10000967	0.0931631	0.0931631	0
LOC10010126	0.0049352	0	0
LOC10010193	0.0019144	0	0.0019144
LOC10012469	0.0062989	0	0
LOC10012555	0.0128552	0.0128552	0
LOC10012678	0.0207006	0	0
LOC10012788	0.0032428	0.0032428	0
LOC10012802	0.0388835	0	0
LOC10012807	0.0013526	0.0013526	0

LOC10012816	0.1073058	0	0.1073058
LOC10012819	0.0791721	0.0791721	0
LOC10012823	0.0373291	0	0
LOC10012828	0.2858576	0	0
LOC10012829	0.0075376	0.0075376	0
LOC10012854	0.0193467	0	0
LOC10012855	0.0031408	0	0
LOC10012857	0.0023107	0.0023107	0
LOC10012864	0.0164343	0.0164343	0
LOC10012867	0.0676488	0.0676488	0
LOC10012878	0.0261903	0.0261903	0
LOC10012881	0.0045236	0	0
LOC10012882	0.0157557	0.0157557	0
LOC10012884	0.0021223	0.0021223	0
LOC10012897	0.0043474	0.0043474	0
LOC10012903	0.0183366	0	0.0183366
LOC10012905	0.0149783	0	0
LOC10012906	0.0245961	0	0
LOC10012938	0.2614234	0	0
LOC10012953	0.0005471	0.0005471	0
LOC10012955	0.0216343	0	0
LOC10012963	0.0015307	0.0015307	0
LOC10012971	0.4440039	0	0
LOC10012972	0.2973045	0	0
LOC10012993	0.0078548	0	0
LOC10013001	0.0012913	0.0012913	0
LOC10013009	0.0507385	0	0
LOC10013014	0.0041262	0	0
LOC10013023	0.0019052	0	0
LOC10013026	0.0022935	0	0.0022935
LOC10013027	0.0019737	0.0019737	0
LOC10013033	0.0404416	0	0.0404416
LOC10013052	0.0304652	0	0
LOC10013055	0.0940866	0.0940866	0
LOC10013058	0.3417289	0.3417289	0
LOC10013069	0.0642583	0	0
LOC10013077	0.0059307	0.0059307	0
LOC10013087	0.002672	0	0
LOC10013093	0.007314	0.007314	0
LOC10013093	0.0049589	0.0049589	0
LOC10013098	0.0273547	0.0273547	0
LOC10013119	0.0131347	0	0
LOC10013143	0.0012333	0.0012333	0
LOC10013149	0.4815879	0.4815879	0
LOC10013155	0.0483809	0.0483809	0

LOC10013169	0.0245147	0.0245147	0
LOC10013172	0.0077488	0.0077488	0
LOC10013211	0.0526583	0	0.0526583
LOC10013221	0.0097224	0.0097224	0
LOC10013224	1.53E-05	0	0
LOC10013235	0.0033865	0	0
LOC10013270	0.0667268	0	0.0667268
LOC10013272	0.0341064	0	0
LOC10013283	0.0050946	0	0
LOC10013283	1.73E-05	0	0
LOC10013305	3.02E-05	0	0
LOC10013316	1.74E-05	1.74E-05	0
LOC10013330	0.0124798	0	0
LOC10013346	0.0048122	0	0
LOC10013354	0.0001716	0	0
LOC10013361	0.0092162	0.0092162	0
LOC10013366	0.0696106	0	0
LOC10013389	0.01953	0	0
LOC10013392	1.56E-05	0	0
LOC10013395	0.0468006	0.0468006	0
LOC10013398	0.1745812	0.1745812	0
LOC10013399	0.0068227	0	0
LOC10013422	0.0251367	0.0251367	0
LOC10013425	0.0090368	0	0.0090368
LOC10013436	0.0028423	0.0028423	0
LOC10013471	0.0131484	0.0131484	0
LOC10013486	0.0015929	0	0
LOC10014460	0.008027	0	0
LOC10014460	0.0031851	0.0031851	0
LOC10016975	0.0066732	0	0
LOC10018894	0.1898824	0	0.1898824
LOC10018894	0.0448257	0	0.0448257
LOC10018958	0.0271056	0.0271056	0
LOC10019093	0.0056449	0	0.0056449
LOC10019093	0.2720011	0.2720011	0
LOC10019094	0.0049274	0	0
LOC10019237	0.0136304	0	0.0136304
LOC10019237	0.0129733	0	0.0129733
LOC10019242	0.0061052	0.0061052	0
LOC10021600	0.5169864	0	0
LOC10021654	0.4319227	0.4319227	0
LOC10023320	0.1823307	0	0.1823307
LOC10024072	0.0075631	0	0
LOC10024073	0.00134	0	0.00134
LOC10024073	0.0028282	0	0.0028282

LOC10026816	0.0838157	0	0
LOC10027071	0.446786	0	0.446786
LOC10027074	0.0672025	0.0672025	0
LOC10027080	0.0213349	0.0213349	0
LOC10027172	0.010955	0	0
LOC10027183	0.0047761	0.0047761	0
LOC10027183	0.0055654	0	0
LOC10027183	0.2565805	0	0.2565805
LOC10027214	0.0111593	0.0111593	0
LOC10027221	0.0614956	0.0614956	0
LOC10027222	0.0014928	0.0014928	0
LOC10028679	3.19E-05	3.19E-05	0
LOC10028684	0.0315705	0	0
LOC10028722	0.6928748	0.6928748	0
LOC10028770	1.54E-05	0	0
LOC10028771	0.0074416	0	0
LOC10028934	0.0363644	0	0
LOC10030240	0.1291758	0	0.1291758
LOC10030264	0.0741548	0	0.0741548
LOC10030265	0.0977161	0	0.0977161
LOC10030372	0.0179645	0	0
LOC113230	0.0166755	0.0166755	0
LOC115110	0.0055258	0.0055258	0
LOC116437	0.015419	0	0.015419
LOC121838	0.0133382	0	0.0133382
LOC121952	0.0208788	0.0208788	0
LOC126536	0.0010946	0	0
LOC127841	0.0342011	0.0342011	0
LOC134466	0.012792	0	0.012792
LOC143188	0.0059239	0	0
LOC143666	0.0030829	0.0030829	0
LOC144438	0.0461422	0	0
LOC144486	0.0589904	0	0
LOC144571	0.0121709	0	0.0121709
LOC144742	0.0020701	0	0
LOC144776	0.0094618	0	0
LOC145474	0.049233	0	0.049233
LOC145783	0.0861882	0	0
LOC145820	0.013771	0	0.013771
LOC145837	0.0701495	0	0
LOC145845	0.0169881	0	0
LOC146336	0.005255	0	0
LOC146481	0.0027022	0	0
LOC146880	0.001977	0.001977	0
LOC147727	0.0867192	0	0

LOC147804	0.0043412	0.0043412	0
LOC148145	0.002488	0	0.002488
LOC148189	0.0336994	0	0
LOC148413	0.017131	0.017131	0
LOC148696	0.2606329	0	0
LOC148709	0.0123378	0	0
LOC148824	0.0140171	0	0.0140171
LOC149134	0.0028996	0	0
LOC149620	0.0297694	0	0.0297694
LOC149837	0.0014362	0	0.0014362
LOC150185	0.0126893	0	0
LOC150197	0.0055308	0.0055308	0
LOC150381	0.0184777	0.0184777	0
LOC150568	0.0097427	0	0
LOC150622	0.002632	0	0
LOC150776	0.0173681	0.0173681	0
LOC150786	0.0007964	0	0
LOC151009	1.59E-05	1.59E-05	0
LOC151162	0.003339	0	0.003339
LOC151174	0.0017564	0	0.0017564
LOC151300	0.0029509	0	0
LOC151534	0.0095216	0.0095216	0
LOC151658	0.0278721	0	0
LOC152024	0.014157	0.014157	0
LOC152217	0.0618334	0.0618334	0
LOC152225	0.3596127	0	0.3596127
LOC153328	0.0066357	0	0
LOC153684	0.235636	0	0.235636
LOC153910	0.0081225	0	0
LOC154449	0.0015665	0	0
LOC154761	2.38E-05	0	2.38E-05
LOC154822	0.008492	0	0.008492
LOC157381	0.0183126	0	0
LOC157627	0.0016772	0	0.0016772
LOC158376	0.0565597	0	0.0565597
LOC158572	0.0144867	0.0144867	0
LOC158696	0.0031093	0	0
LOC162632	6.75E-05	0	0
LOC168474	0.0033203	0	0
LOC200726	0.0107254	0	0
LOC201651	0.1133208	0	0
LOC202181	0.0001949	0	0
LOC202781	0.0283112	0.0283112	0
LOC219347	0.0219483	0	0.0219483
LOC220429	0.0019485	0.0019485	0

LOC220594	2.39E-05	0	0
LOC220729	0.0001407	0.0001407	0
LOC220930	0.0405339	0.0405339	0
LOC221122	0.0030964	0	0.0030964
LOC221442	0.0157497	0	0
LOC221710	0.0549871	0	0.0549871
LOC222699	0.1335724	0.1335724	0
LOC253039	0.0809616	0	0
LOC253724	0.20565	0	0
LOC254312	0.002514	0	0
LOC254559	0.0041901	0	0.0041901
LOC255025	0.0213426	0	0
LOC255167	0.0087681	0	0.0087681
LOC256880	0.1356213	0.1356213	0
LOC257358	0.2061516	0	0.2061516
LOC25845	0.0041552	0.0041552	0
LOC26102	0.0003053	0	0
LOC282997	0.3715271	0	0.3715271
LOC283050	0.0193549	0.0193549	0
LOC283070	0.0024648	0	0.0024648
LOC283174	0.0023936	0.0023936	0
LOC283267	0.0118662	0	0.0118662
LOC283314	0.7602905	0	0
LOC283332	0.0102091	0	0
LOC283392	0.0117432	0	0
LOC283404	0.780158	0.780158	0
LOC283663	0.0226972	0	0.0226972
LOC283731	0.0054378	0	0.0054378
LOC283761	0.0381508	0	0
LOC283856	0.0072477	0	0.0072477
LOC283867	0.0130157	0	0.0130157
LOC283914	0.0259446	0	0
LOC283922	0.0008329	0	0
LOC283999	0.0011805	0	0.0011805
LOC284009	0.03179	0	0
LOC284023	0.0232123	0	0
LOC284100	0.0032926	0	0
LOC284232	0.0006894	0	0
LOC284233	0.000633	0.000633	0
LOC284276	0.005091	0	0.005091
LOC284379	0.0007378	0	0
LOC284440	0.0506059	0	0
LOC284441	0.0018904	0	0.0018904
LOC284551	0.0190395	0	0
LOC284578	0.0311892	0	0

LOC284632	0.0641281	0	0
LOC284661	0.0024073	0	0
LOC284688	0.0097074	0	0.0097074
LOC284749	0.0123628	0	0.0123628
LOC284788	0.0248508	0.0248508	0
LOC284798	0.003015	0	0
LOC284837	0.0070386	0	0
LOC284900	0.0585749	0.0585749	0
LOC285033	0.0171516	0	0
LOC285045	0.0165685	0.0165685	0
LOC285074	0.0001078	0.0001078	0
LOC285194	0.0106161	0	0
LOC285205	0.0064734	0	0.0064734
LOC285359	0.0272805	0.0272805	0
LOC285370	0.0060989	0	0
LOC285375	0.0071372	0	0
LOC285401	0.0031545	0	0
LOC285419	0.023679	0.023679	0
LOC285456	0.0737303	0	0.0737303
LOC285501	0.0161776	0.0161776	0
LOC285548	0.0051173	0	0.0051173
LOC285593	0.0098617	0	0.0098617
LOC285627	0.1448342	0.1448342	0
LOC285629	0.0445482	0	0
LOC285692	0.0157238	0	0
LOC285696	0.2972553	0	0.2972553
LOC285733	0.0060736	0	0
LOC285735	0.0078805	0	0
LOC285740	0.0168863	0	0
LOC285768	0.0212055	0	0.0212055
LOC285780	0.0156792	0	0.0156792
LOC285796	0.0014308	0	0
LOC285847	0.218867	0	0
LOC285954	0.0524745	0	0.0524745
LOC286002	0.0964596	0	0.0964596
LOC286094	0.0157973	0	0
LOC286135	0.0882974	0	0.0882974
LOC286238	0.0645356	0	0
LOC286359	0.0102522	0.0102522	0
LOC286367	0.2150221	0	0.2150221
LOC286467	0.0668985	0.0668985	0
LOC29034	0.0205358	0	0
LOC338588	0.0840576	0.0840576	0
LOC338651	0.0007336	0.0007336	0
LOC338758	0.1268435	0	0.1268435

LOC338799	0.0811615	0.0811615	0
LOC339240	3.23E-05	3.23E-05	0
LOC339290	0.0244959	0	0
LOC339524	0.0072419	0	0.0072419
LOC339535	0.0331213	0	0.0331213
LOC339568	0.0064386	0	0
LOC339674	0.0111323	0.0111323	0
LOC339788	0.0175135	0.0175135	0
LOC340017	0.0062503	0	0
LOC340074	0.0384533	0	0
LOC340094	0.0145467	0	0
LOC340357	0.0041564	0.0041564	0
LOC340508	0.0045343	0	0
LOC341056	0.0036609	0	0
LOC342346	0.0021651	0	0.0021651
LOC344595	0.0726556	0	0.0726556
LOC344967	0.1691221	0	0
LOC347376	0.0105411	0	0
LOC348021	0.0020983	0	0
LOC348840	0.0221261	0	0.0221261
LOC348926	0.024341	0.024341	0
LOC349114	0.0561995	0.0561995	0
LOC360030	0.0024236	0	0
LOC374443	0.1855983	0	0.1855983
LOC374491	0.0018266	0	0.0018266
LOC375190	0.0248402	0	0.0248402
LOC387646	0.0116874	0	0.0116874
LOC387647	0.0125446	0	0.0125446
LOC388152	1.53E-05	1.53E-05	0
LOC388387	0.0082083	0	0
LOC388428	0.0003389	0.0003389	0
LOC388588	0.0012618	0.0012618	0
LOC388692	0.0014117	0	0
LOC388789	0.0253383	0.0253383	0
LOC388796	0.0516533	0.0516533	0
LOC388946	0.4101174	0	0.4101174
LOC388955	0.006171	0	0
LOC389033	0.0010751	0	0
LOC389332	0.0057504	0.0057504	0
LOC389333	0.1085505	0.1085505	0
LOC389458	0.0087422	0.0087422	0
LOC389493	0.0036382	0	0
LOC389634	0.000152	0	0.000152
LOC389705	0.0069703	0	0.0069703
LOC389791	0.0467684	0.0467684	0

LOC390595	0.0087507	0	0
LOC390858	0.0121666	0	0
LOC391322	0.012746	0.012746	0
LOC392196	0.0002136	0	0
LOC399744	3.98E-05	3.98E-05	0
LOC399815	0.0285582	0.0285582	0
LOC399959	0.2109271	0	0.2109271
LOC400027	0.3718863	0.3718863	0
LOC400043	0.0048703	0	0
LOC400657	0.0430174	0	0.0430174
LOC400696	0.0905307	0	0.0905307
LOC400752	0.0467172	0.0467172	0
LOC400759	0.0033882	0	0.0033882
LOC400794	0.0220253	0	0
LOC400804	0.1244685	0	0.1244685
LOC400891	0.0061618	0.0061618	0
LOC400927	0.0313103	0	0
LOC400931	0.3088898	0.3088898	0
LOC400940	0.0024291	0	0.0024291
LOC401010	0.0006535	0.0006535	0
LOC401052	0.005992	0.005992	0
LOC401093	1	0	1
LOC401127	0.0034802	0	0
LOC401387	0.0242882	0	0
LOC401397	0.0681878	0	0
LOC401431	0.0031462	0	0.0031462
LOC401463	0.0252676	0	0.0252676
LOC401588	0.0238547	0	0
LOC402377	0.1589765	0	0.1589765
LOC402644	0.0082748	0	0
LOC407835	0.0248997	0.0248997	0
LOC415056	0.0017158	0	0
LOC440040	0.0089089	0	0
LOC440173	0.0025986	0	0.0025986
LOC440356	0.0255341	0.0255341	0
LOC440461	0.0153246	0	0.0153246
LOC440563	0.0012561	0	0
LOC440896	1.73E-05	0	1.73E-05
LOC440905	2.94E-05	0	0
LOC440925	0.0027965	0.0027965	0
LOC440944	0.2757916	0.2757916	0
LOC440957	0.022475	0.022475	0
LOC441046	0.0191851	0	0.0191851
LOC441089	0.0045731	0	0.0045731
LOC441177	0.0147849	0	0.0147849

LOC441204	0.0187189	0	0.0187189
LOC441208	0.006964	0.006964	0
LOC441294	1.62E-05	1.62E-05	0
LOC441454	4.16E-05	0	0
LOC441455	0.0032789	0	0
LOC441601	0.0009423	0	0.0009423
LOC441666	0.0029806	0	0.0029806
LOC441869	0.0022883	0	0.0022883
LOC442308	0.0102689	0	0.0102689
LOC442421	0.0003232	0	0.0003232
LOC442454	0.0513385	0	0
LOC442459	0.0033802	0	0
LOC493754	0.0135513	0.0135513	0
LOC494141	0.0066969	0	0
LOC541471	0.0002473	0	0.0002473
LOC541473	1.68E-05	1.68E-05	0
LOC550112	0.065943	0.065943	0
LOC550643	0.044933	0.044933	0
LOC554202	0.0652844	0	0
LOC55908	0.0026945	0	0
LOC572558	7.96E-05	0	7.96E-05
LOC595101	0.0021235	0	0.0021235
LOC613037	1.53E-05	0	0
LOC619207	0.0013643	0.0013643	0
LOC641298	0.0001553	0	0.0001553
LOC641367	0.0055339	0	0.0055339
LOC642587	0.1989397	0	0.1989397
LOC642597	0.0038267	0	0.0038267
LOC642846	0.0090527	0.0090527	0
LOC642852	0.0031626	0	0.0031626
LOC642929	2.46E-05	0	0
LOC643008	0.002144	0.002144	0
LOC643387	0.0019165	0	0
LOC643486	0.003173	0	0
LOC643677	0.0103349	0	0.0103349
LOC643719	0.0017165	0.0017165	0
LOC643763	0.0070164	0	0.0070164
LOC643837	0.0027622	0	0.0027622
LOC643923	0.0071492	0	0
LOC643955	1.53E-05	0	0
LOC644145	0.0056999	0	0
LOC644165	0.0001391	0.0001391	0
LOC644172	0.0002038	0	0
LOC644538	0.0166044	0	0.0166044
LOC644669	0.0001318	0	0.0001318

LOC644936	0.0024389	0	0
LOC645166	0.0037363	0	0.0037363
LOC645323	0.0107617	0	0.0107617
LOC645332	0.0041413	0.0041413	0
LOC645431	0.0453848	0.0453848	0
LOC645676	0.0729608	0.0729608	0
LOC645752	0.0006708	0	0
LOC646214	0.0095678	0	0.0095678
LOC646471	0.0174937	0	0
LOC646498	0.0024797	0	0.0024797
LOC646627	0.0047044	0	0
LOC646762	0.0026647	0.0026647	0
LOC646813	0.0137816	0	0
LOC646851	0.0075669	0.0075669	0
LOC646982	0.0108897	0	0.0108897
LOC646999	0.0415167	0	0.0415167
LOC647121	0.0207777	0	0
LOC647288	0.002249	0.002249	0
LOC647309	0.0295629	0	0.0295629
LOC647946	0.0113394	0.0113394	0
LOC647979	0.0421094	0	0.0421094
LOC648691	0.0024892	0	0
LOC648740	0.0109559	0	0
LOC649330	0.0021289	0	0
LOC650293	0.0004039	0	0
LOC650368	0.0255449	0	0.0255449
LOC651250	0.0098452	0	0.0098452
LOC652276	0.0044305	0.0044305	0
LOC653113	0.0007218	0.0007218	0
LOC653566	0.0251772	0	0
LOC653653	0.0089119	0	0.0089119
LOC653786	0.0001247	0	0.0001247
LOC654342	0.0010624	0	0.0010624
LOC654433	0.0173093	0	0
LOC678655	0.0341721	0.0341721	0
LOC723809	0.022999	0	0
LOC723972	0.0026365	0	0
LOC727677	0.120374	0	0
LOC727896	0.0196576	0	0.0196576
LOC727924	1.53E-05	0	1.53E-05
LOC728024	0.0195756	0	0.0195756
LOC728190	0.0010266	0.0010266	0
LOC728264	0.0056387	0	0.0056387
LOC728276	0.0163336	0.0163336	0
LOC728323	0.0113841	0	0.0113841

LOC728392	0.0040669	0	0.0040669
LOC728554	3.16E-05	3.16E-05	0
LOC728606	0.0266781	0.0266781	0
LOC728613	0.0045535	0.0045535	0
LOC728640	0.0127228	0	0
LOC728643	0.093921	0	0
LOC728723	0.0434673	0	0.0434673
LOC728743	0.0146751	0	0
LOC728758	0.0135338	0.0135338	0
LOC728819	0.01176	0	0.01176
LOC728855	1.54E-05	0	0
LOC728989	0.0219344	0	0.0219344
LOC729020	0.0465436	0	0
LOC729082	0.1318859	0	0.1318859
LOC729121	0.0003942	0	0
LOC729156	0.0168938	0	0
LOC729176	0.0106756	0	0.0106756
LOC729234	0.0012492	0.0012492	0
LOC729375	0.0008967	0.0008967	0
LOC729467	0.2132509	0	0.2132509
LOC729603	0.0029324	0	0.0029324
LOC729609	0.0139054	0.0139054	0
LOC729668	0.0032464	0	0.0032464
LOC729678	0.037275	0	0.037275
LOC729799	0.0123645	0	0
LOC729991	0.010372	0.010372	0
LOC729991-M	0.010372	0	0
LOC730101	0.0576804	0	0
LOC730668	0.0863782	0.0863782	0
LOC730811	0.0048161	0	0.0048161
LOC731779	0.0166802	0.0166802	0
LOC731789	0.0034599	0	0.0034599
LOC732275	0.0389296	0	0
LOC80054	0.0077645	0	0
LOC81691	0.0464961	0.0464961	0
LOC84740	0.002079	0.002079	0
LOC84856	0.0029587	0	0.0029587
LOC84931	0.0118695	0	0
LOC84989	0.0605819	0.0605819	0
LOC90110	0.0080927	0.0080927	0
LOC90246	0.0076936	0	0
LOC90586	0.0032287	0	0.0032287
LOC90784	0.0104976	0	0
LOC90834	0.0005129	0	0
LOC91149	0.0033763	0	0

LOC91316	0.0076598	0	0
LOC91450	0.0130093	0	0
LOC91948	0.0038222	0	0
LOC92249	0.0243776	0.0243776	0
LOC92973	0.0032696	0.0032696	0
LOC93432	0.0066203	0	0
LOC93622	0.0090963	0.0090963	0
LOC96610	0.0001345	0	0.0001345
LOH12CR1	0.1502715	0	0
LOH12CR2	0.1308434	0	0.1308434
LOH3CR2A	0.0103009	0	0.0103009
LONP1	0.0204496	0.0204496	0
LONP2	0.0306397	0	0
LONRF1	0.0842497	0	0.0842497
LONRF2	0.0078272	0	0
LONRF3	0.0036474	0.0036474	0
LOR	0.0232767	0	0.0232767
LOX	0.1350248	0	0.1350248
LOXHD1	0.0046837	0	0.0046837
LOXL1	0.0068007	0	0.0068007
LOXL2	0.1078154	0	0.1078154
LOXL3	0.024164	0	0.024164
LOXL4	0.002376	0	0
LPA	0.004428	0	0
LPAL2	0.0055013	0	0.0055013
LPAR1	0.0115622	0	0.0115622
LPAR2	0.0044856	0.0044856	0
LPAR3	0.0220743	0	0.0220743
LPAR4	0.0137074	0	0.0137074
LPAR5	0.0367536	0.0367536	0
LPAR6	0.0381356	0	0.0381356
LPCAT1	0.0314566	0	0
LPCAT2	0.0068644	0	0
LPCAT3	0.1020958	0.1020958	0
LPCAT4	0.0133684	0.0133684	0
LPGAT1	0.1120048	0.1120048	0
LPHN1	0.0062789	0	0
LPHN2	0.0098203	0	0.0098203
LPHN3	0.0056316	0	0.0056316
LPIN1	0.2332396	0	0.2332396
LPIN2	0.0473112	0	0
LPIN3	0.0109235	0.0109235	0
LPL	0.004376	0	0.004376
LPO	0.0118213	0.0118213	0
LPP	0.1287598	0	0.1287598

LPPR1	0.0073681	0	0
LPPR2	0.004665	0	0.004665
LPPR3	0.0004891	0	0.0004891
LPPR4	0.0101675	0	0.0101675
LPPR5	0.0055044	0	0.0055044
LPXN	0.724141	0	0.724141
LQK1	0.0834774	0	0
LRAT	0.0049235	0.0049235	0
LRBA	0.0378939	0.0378939	0
LRCH1	0.0569171	0	0.0569171
LRCH2	0.0084488	0	0.0084488
LRCH3	0.1549161	0	0.1549161
LRCH4	0.0145706	0.0145706	0
LRDD	0.0070268	0.0070268	0
LRFN1	0.001069	0	0.001069
LRFN2	0.001739	0	0.001739
LRFN3	0.0071162	0.0071162	0
LRFN4	0.0024831	0.0024831	0
LRFN5	0.0039141	0	0.0039141
LRG1	0.2565279	0.2565279	0
LRGUK	0.010868	0.010868	0
LRIG1	0.0134148	0	0.0134148
LRIG2	0.0190621	0	0.0190621
LRIG3	0.0614974	0	0
LRIT1	0.0020018	0	0
LRIT2	0.0047458	0	0
LRIT3	0.0028284	0.0028284	0
LRMP	0.1458151	0	0.1458151
LRP1	0.0116457	0	0.0116457
LRP10	0.2424194	0.2424194	0
LRP11	0.1869236	0.1869236	0
LRP12	0.1170505	0	0.1170505
LRP1B	0.0172337	0	0.0172337
LRP2	0.0044313	0	0
LRP2BP	0.0303623	0	0
LRP3	0.0015962	0	0.0015962
LRP4	0.0166634	0	0.0166634
LRP5	0.0259898	0.0259898	0
LRP5L	0.0015515	0.0015515	0
LRP6	0.0200334	0	0
LRP8	0.0178438	0	0
LRPAP1	0.0006571	0	0
LRPPRC	0.0528042	0.0528042	0
LRRC1	0.0638676	0.0638676	0
LRRC10	0.0074328	0.0074328	0

LRRC10B	0.0019109	0	0.0019109
LRRC14	0.00837	0.00837	0
LRRC14B	0.0030382	0	0.0030382
LRRC15	0.0039413	0	0.0039413
LRRC16A	0.05943	0	0
LRRC16B	0.0033371	0.0033371	0
LRRC17	0.0125859	0	0.0125859
LRRC18	0.0106825	0	0.0106825
LRRC19	0.0061776	0	0
LRRC2	0.0084122	0	0.0084122
LRRC20	0.0715875	0.0715875	0
LRRC23	0.0213172	0.0213172	0
LRRC24	0.0037357	0.0037357	0
LRRC25	0.0625069	0	0.0625069
LRRC26	0.0012711	0.0012711	0
LRRC27	0.0571491	0	0
LRRC28	0.2271316	0	0.2271316
LRRC29	0.0124697	0.0124697	0
LRRC3	0.0011227	0	0.0011227
LRRC30	0.0043999	0	0
LRRC31	0.0178483	0.0178483	0
LRRC32	0.0165421	0	0.0165421
LRRC33	0.0583951	0	0.0583951
LRRC34	0.0249935	0	0.0249935
LRRC36	0.0273883	0.0273883	0
LRRC37A	0.0012586	0	0
LRRC37A2	1.75E-05	0	0
LRRC37A3	0.0076603	0	0.0076603
LRRC37A4	2.20E-05	0	2.20E-05
LRRC37B	0.0023188	0	0.0023188
LRRC37B2	0.001684	0	0
LRRC39	0.0125954	0	0.0125954
LRRC3B	0.0033185	0	0.0033185
LRRC4	0.0204543	0	0.0204543
LRRC40	0.0588978	0	0.0588978
LRRC41	0.055828	0	0.055828
LRRC42	0.0460336	0	0
LRRC43	0.0052759	0	0.0052759
LRRC45	0.0017485	0.0017485	0
LRRC46	0.0212128	0.0212128	0
LRRC47	0.0115971	0.0115971	0
LRRC48	0.0372308	0	0
LRRC49	0.0691194	0	0.0691194
LRRC4B	0.0011469	0	0.0011469
LRRC4C	0.0194503	0	0.0194503

LRRC50	0.0127695	0	0.0127695
LRRC52	0.0176649	0	0
LRRC55	0.0032441	0	0.0032441
LRRC56	0.0018866	0.0018866	0
LRRC57	0.0472516	0.0472516	0
LRRC58	0.0728483	0	0.0728483
LRRC59	0.0257533	0	0
LRRC6	0.0195758	0	0
LRRC61	0.1942533	0.1942533	0
LRRC66	0.0125148	0.0125148	0
LRRC67	0.0079304	0	0
LRRC69	0.0133495	0.0133495	0
LRRC7	0.0150502	0	0.0150502
LRRC70	0.0234874	0	0.0234874
LRRC8A	0.0959805	0	0
LRRC8B	0.0673256	0	0
LRRC8C	0.054511	0	0.054511
LRRC8D	0.0506282	0	0.0506282
LRRC8E	0.0059164	0.0059164	0
LRRCC1	0.0280834	0	0.0280834
LRRFIP1	0.1603704	0.1603704	0
LRRFIP2	0.1315426	0	0
LRRIQ1	0.0380961	0	0.0380961
LRRIQ3	0.0366868	0	0
LRRIQ4	0.0182889	0	0
LRRK1	0.0462616	0	0.0462616
LRRK2	0.1339997	0	0.1339997
LRRN1	0.003629	0	0
LRRN2	0.0097861	0	0.0097861
LRRN3	0.0562696	0	0.0562696
LRRN4	0.0062263	0	0
LRRN4CL	0.0129793	0	0.0129793
LRRTM1	0.0037515	0	0
LRRTM2	0.0292825	0	0.0292825
LRRTM3	0.0195022	0	0.0195022
LRRTM4	0.0114664	0	0.0114664
LRSAM1	0.1773414	0.1773414	0
LRTM1	0.0050694	0	0
LRTM2	0.0012012	0.0012012	0
LRTOMT	0.1403196	0.1403196	0
LRWD1	0.0031197	0.0031197	0
LSAMP	0.0074956	0	0.0074956
LSG1	0.161066	0.161066	0
LSM1	0.0751064	0	0
LSM10	0.0145539	0	0.0145539

LSM11	0.0594149	0	0.0594149
LSM12	0.0230078	0.0230078	0
LSM14A	0.0706274	0	0.0706274
LSM14B	0.0079433	0.0079433	0
LSM3	0.152121	0.152121	0
LSM4	0.0485413	0.0485413	0
LSM5	0.0966241	0.0966241	0
LSM6	0.0463796	0	0
LSM7	0.0173572	0.0173572	0
LSMD1	0.0729103	0.0729103	0
LSP1	0.0096198	0	0.0096198
LSR	0.0907264	0.0907264	0
LSS	0.0239508	0	0
LST-3TM12	0.0053302	0	0
LTA4H	0.1177024	0	0.1177024
LTB4R	0.2677391	0	0
LTB4R2	0.4111142	0.4111142	0
LTBP1	0.0446523	0	0.0446523
LTBP2	0.0380855	0	0.0380855
LTBP3	0.0117266	0	0.0117266
LTBP4	0.0352039	0	0.0352039
LTBR	0.0830343	0.0830343	0
LTC4S	0.0037852	0	0.0037852
LTF	0.004974	0	0.004974
LTK	0.0066228	0	0
LTV1	0.0231259	0	0.0231259
LUC7L	0.0241705	0.0241705	0
LUC7L2	0.0801165	0.0801165	0
LUC7L3	0.0424704	0.0424704	0
LUM	0.0333492	0	0.0333492
LUZP1	0.0492828	0	0.0492828
LUZP2	0.0049094	0	0.0049094
LUZP4	0.0040882	0	0
LUZP6	0.3534494	0	0.3534494
LXN	0.0326467	0	0.0326467
LY6D	0.0014883	0	0
LY6E	0.0668124	0	0
LY6H	0.0011193	0	0
LY6K	0.1331435	0	0.1331435
LY75	0.0775527	0	0
LY86	0.1203166	0	0.1203166
LY9	0.0935365	0	0.0935365
LY96	0.0448921	0	0.0448921
LYAR	0.0122084	0	0.0122084
LYG1	0.0023817	0.0023817	0

LYG2	0.00724	0	0
LYL1	0.0063542	0	0.0063542
LYN	0.060861	0	0.060861
LYNX1	0.0016941	0	0.0016941
LYPD1	0.3108967	0	0.3108967
LYPD2	0.001095	0	0
LYPD3	0.0287891	0	0
LYPD4	0.0026101	0	0
LYPD5	0.020988	0	0.020988
LYPD6	0.0098092	0	0
LYPD6B	0.0111815	0	0
LYPLA1	0.0350335	0.0350335	0
LYPLA2	0.0401956	0.0401956	0
LYPLAL1	0.3695655	0	0
LYRM1	0.6757061	0	0
LYRM2	0.0599008	0	0.0599008
LYRM4	0.0547673	0	0.0547673
LYRM5	0.0562232	0	0
LYRM7	0.1512279	0	0.1512279
LYSMD1	0.1702566	0.1702566	0
LYSMD2	0.1009331	0	0
LYSMD3	0.0593541	0	0.0593541
LYSMD4	0.0167281	0.0167281	0
LYST	0.1402185	0	0.1402185
LYVE1	0.0116667	0	0.0116667
LYZ	0.0131355	0	0
LYZL1	0.0091656	0.0091656	0
LYZL2	0.2475995	0	0
LYZL4	0.006729	0.006729	0
LYZL6	0.0057892	0	0
LZIC	0.0656085	0	0.0656085
LZTFL1	0.0437692	0	0
LZTR1	0.004	0	0.004
LZTS1	0.0010214	0	0.0010214
LZTS2	0.0099936	0	0
M6PR	0.0666743	0	0.0666743
MAB21L1	0.0042638	0	0.0042638
MAB21L2	0.008623	0	0.008623
MACC1	0.1904734	0.1904734	0
MACF1	0.0627848	0	0.0627848
MACROD1	0.0047726	0.0047726	0
MACROD2	0.0142323	0	0
MAD1L1	0.0296662	0.0296662	0
MAD2L1	0.072778	0.072778	0
MAD2L1BP	0.1196855	0.1196855	0

MAD2L2	0.0246605	0	0.0246605
MADCAM1	0.0014178	0	0.0014178
MADD	0.153256	0	0
MAEA	0.0052964	0.0052964	0
MAEL	0.0176677	0	0.0176677
MAF	0.0135818	0	0.0135818
MAF1	0.0103023	0.0103023	0
MAFA	0.0008163	0	0.0008163
MAFB	0.0064202	0	0.0064202
MAFF	0.0386169	0.0386169	0
MAFG	0.0032189	0	0.0032189
MAFK	0.069691	0.069691	0
MAG	0.0037728	0	0.0037728
MAGEA1	0.0017885	0	0
MAGEA10	0.0017403	0.0017403	0
MAGEA11	0.00316	0	0
MAGEA12	0.001016	0	0
MAGEA3	1.72E-05	0	0
MAGEA4	0.0008561	0	0.0008561
MAGEA5	0.0015271	0.0015271	0
MAGEA6	1.83E-05	1.83E-05	0
MAGEA8	0.0023004	0	0
MAGEB1	0.004345	0	0
MAGEB10	0.0029961	0	0
MAGEB16	0.0055545	0	0
MAGEB18	0.0043459	0	0
MAGEB2	0.0027586	0	0.0027586
MAGEB3	0.0026402	0	0
MAGEB4	0.0026453	0	0
MAGEB6	0.0015868	0	0
MAGEC1	0.0011684	0	0
MAGEC2	0.001936	0	0
MAGEC3	0.0063631	0	0
MAGED1	0.0146319	0	0
MAGED2	0.0079637	0	0.0079637
MAGED4	3.61E-05	0	0
MAGED4B	3.74E-05	0	3.74E-05
MAGEE1	0.0031346	0	0.0031346
MAGEE2	0.0051451	0	0.0051451
MAGEF1	0.0609205	0.0609205	0
MAGEH1	0.0005221	0	0.0005221
MAGEL2	0.0017146	0	0.0017146
MAGI1	0.0052428	0.0052428	0
MAGI2	0.0055446	0	0.0055446
MAGI3	0.1270334	0	0

MAGIX	0.0406932	0.0406932	0
MAGOH	0.0320592	0	0
MAGOHB	0.0981883	0.0981883	0
MAGT1	0.1071408	0	0
MAK	0.0404137	0	0.0404137
MAK16	0.0714653	0	0.0714653
MAL	0.0061049	0	0.0061049
MAL2	0.368931	0.368931	0
MALAT1	1	0	0
MALL	0.0111242	0.0111242	0
MALT1	0.3621403	0	0.3621403
MAMDC2	0.0270353	0	0.0270353
MAMDC4	0.0062135	0.0062135	0
MAML1	0.136201	0	0.136201
MAML2	0.1601639	0	0.1601639
MAML3	0.0175644	0	0
MAMLD1	0.0081011	0	0.0081011
MAMSTR	0.0181029	0	0
MAN1A1	0.1007457	0	0.1007457
MAN1A2	0.0831508	0	0.0831508
MAN1B1	0.0371187	0	0
MAN1C1	0.0139445	0	0.0139445
MAN2A1	0.2338493	0	0.2338493
MAN2A2	0.0149373	0	0
MAN2B1	0.0110798	0	0.0110798
MAN2B2	0.0019561	0	0
MAN2C1	0.0598529	0.0598529	0
MANBA	0.0180817	0	0.0180817
MANBAL	0.1708915	0.1708915	0
MANEA	0.0687193	0	0.0687193
MANEAL	0.0051805	0.0051805	0
MANF	0.0226919	0.0226919	0
MANSC1	0.0545087	0.0545087	0
MAOA	0.0149406	0.0149406	0
MAOB	0.0132778	0	0
MAP1A	0.0129404	0	0.0129404
MAP1B	0.0125483	0	0.0125483
MAP1D	0.0241992	0	0
MAP1LC3A	0.0071358	0	0
MAP1LC3B	0.2623316	0	0.2623316
MAP1LC3B2	0.0508336	0	0.0508336
MAP1LC3C	0.082301	0	0.082301
MAP1S	0.0075641	0.0075641	0
MAP2	0.0217228	0	0.0217228
MAP2K1	0.1026368	0	0.1026368

MAP2K2	0.011019	0.011019	0
MAP2K3	0.1503745	0.1503745	0
MAP2K4	0.0275439	0	0.0275439
MAP2K5	0.0850068	0	0
MAP2K6	0.1212851	0	0
MAP2K7	0.0327113	0.0327113	0
MAP3K1	0.0734739	0	0.0734739
MAP3K10	0.0085232	0.0085232	0
MAP3K11	0.0292574	0.0292574	0
MAP3K12	0.0475326	0	0.0475326
MAP3K13	0.0866471	0.0866471	0
MAP3K14	0.5072312	0	0.5072312
MAP3K15	0.0015806	0	0.0015806
MAP3K2	0.0165084	0	0.0165084
MAP3K3	0.0323556	0	0.0323556
MAP3K4	0.0265454	0	0.0265454
MAP3K5	0.2701857	0	0
MAP3K6	0.1380447	0	0
MAP3K7	0.05817	0	0.05817
MAP3K8	0.3755293	0	0.3755293
MAP3K9	0.0547408	0.0547408	0
MAP4	0.0488374	0	0.0488374
MAP4K1	0.0197365	0	0.0197365
MAP4K2	0.0083064	0	0.0083064
MAP4K3	0.3666717	0	0
MAP4K4	0.0492965	0	0.0492965
MAP4K5	0.0862659	0	0.0862659
MAP6	0.024398	0	0.024398
MAP6D1	0.0063161	0	0
MAP7	0.0268547	0.0268547	0
MAP7D1	0.0681766	0	0.0681766
MAP7D2	0.0071614	0	0
MAP7D3	0.0202255	0	0.0202255
MAP9	0.0141476	0	0.0141476
MAPK1	0.0492266	0	0.0492266
MAPK10	0.0186628	0	0.0186628
MAPK11	0.001855	0	0.001855
MAPK12	0.0009485	0	0.0009485
MAPK13	0.0176498	0.0176498	0
MAPK14	0.1717456	0	0.1717456
MAPK15	0.0011899	0.0011899	0
MAPK1IP1L	0.1130446	0	0
MAPK3	0.0867758	0.0867758	0
MAPK4	0.0035586	0	0.0035586
MAPK6	0.3027989	0.3027989	0

MAPK7	0.0224475	0	0.0224475
MAPK8	0.0278951	0	0.0278951
MAPK8IP1	0.0073792	0	0.0073792
MAPK8IP2	0.0008886	0	0
MAPK8IP3	0.0181533	0.0181533	0
MAPK9	0.0321513	0	0
MAPKAP1	0.1670655	0	0.1670655
MAPKAPK2	0.1927541	0	0.1927541
MAPKAPK3	0.0692614	0	0
MAPKAPK5	0.0111468	0.0111468	0
MAPKBP1	0.2268365	0	0.2268365
MAPKSP1	0.0363774	0.0363774	0
MAPRE1	0.0181621	0	0.0181621
MAPRE2	0.1791721	0	0.1791721
MAPRE3	0.0148533	0	0
MAPT	0.0050025	0	0.0050025
1-Mar	0.0167415	0	0.0167415
10-Mar	0.0092467	0.0092467	0
11-Mar	0.0084747	0	0
2-Mar	0.0045772	0	0.0045772
3-Mar	0.052374	0	0
4-Mar	0.0101113	0	0.0101113
5-Mar	0.0983557	0	0
6-Mar	0.2059863	0.2059863	0
7-Mar	0.0684343	0	0.0684343
8-Mar	0.0251674	0	0.0251674
9-Mar	0.0643198	0.0643198	0
MARCKS	0.1116862	0	0.1116862
MARCKSL1	0.0095318	0.0095318	0
MARCO	0.0056851	0	0.0056851
MARK1	0.0649839	0	0.0649839
MARK2	0.0595792	0.0595792	0
MARK3	0.0406083	0.0406083	0
MARK4	0.0022713	0.0022713	0
MARS	0.0329479	0.0329479	0
MARS2	0.0396017	0	0
MARVELD1	0.1191087	0	0.1191087
MARVELD3	0.0049945	0.0049945	0
MAS1	0.0107802	0	0.0107802
MASP1	0.0214682	0	0.0214682
MASP2	0.0036032	0	0.0036032
MAST1	0.01929	0.01929	0
MAST2	0.0386859	0.0386859	0
MAST3	0.0183744	0	0
MAST4	0.113038	0	0.113038

MASTL	0.0572563	0	0.0572563
MAT1A	0.0094432	0	0
MAT2A	0.3408317	0.3408317	0
MAT2B	0.4439981	0	0.4439981
MATK	0.001249	0	0.001249
MATN1	0.0079795	0	0.0079795
MATN2	0.0044681	0	0.0044681
MATN3	0.0062588	0	0.0062588
MATN4	0.0032204	0	0
MATR3	0.2800829	0	0.2800829
MAVS	0.010684	0	0
MAX	0.038716	0	0.038716
MAZ	0.0339142	0.0339142	0
MB	0.0283769	0.0283769	0
MBD1	0.0399883	0	0.0399883
MBD2	0.1202788	0	0.1202788
MBD3	0.0074348	0	0
MBD3L1	0.052667	0	0
MBD3L2	1.91E-05	0	0
MBD3L5	1.64E-05	0	0
MBD4	0.1270053	0	0.1270053
MBD5	0.1760374	0	0.1760374
MBD6	0.0803204	0.0803204	0
MBIP	0.0998789	0.0998789	0
MBL1P	0.0101788	0.0101788	0
MBL2	0.0097339	0	0.0097339
MBLAC1	0.0398609	0.0398609	0
MBLAC2	0.06118	0	0.06118
MBNL1	1	0	1
MBNL2	0.3024304	0	0.3024304
MBNL3	0.0533221	0	0.0533221
MBOAT1	0.0967191	0.0967191	0
MBOAT2	0.0753353	0.0753353	0
MBOAT4	0.0257105	0	0.0257105
MBOAT7	0.037807	0.037807	0
MBP	0.0971555	0	0.0971555
MBTD1	0.1136052	0	0
MBTPS1	0.0214082	0	0.0214082
MBTPS2	0.0282837	0	0.0282837
MC1R	0.0818936	0.0818936	0
MC2R	0.0031158	0	0.0031158
MC3R	0.0102031	0	0.0102031
MC4R	0.0072114	0	0.0072114
MC5R	0.0073938	0	0.0073938
MCAM	0.0099362	0	0.0099362

MCART1	0.0452465	0	0.0452465
MCART2	0.01053	0	0.01053
MCART3P	0.0044951	0	0.0044951
MCART6	0.0169201	0	0.0169201
MCAT	0.0077288	0.0077288	0
MCC	0.0391819	0	0.0391819
MCCC1	0.0438807	0	0
MCEE	0.0760451	0.0760451	0
MCF2	0.0094579	0	0.0094579
MCF2L	0.0010351	0	0
MCF2L2	0.0085953	0	0.0085953
MCFD2	0.0929363	0	0.0929363
MCHR1	0.0567104	0	0.0567104
MCHR2	0.0091634	0	0
MCL1	0.3949358	0	0.3949358
MCM10	0.0097399	0.0097399	0
MCM2	0.0542888	0	0
MCM3	0.2757524	0	0
MCM3AP	0.0183825	0	0
MCM3APAS	0.0240016	0.0240016	0
MCM4	0.0498265	0	0
MCM5	0.0151151	0	0
MCM6	0.0216841	0	0.0216841
MCM7	0.0165179	0.0165179	0
MCM8	0.2010792	0	0
MCM9	0.0232531	0	0.0232531
MCOLN1	0.0067475	0	0.0067475
MCOLN2	0.0799341	0	0.0799341
MCOLN3	0.0136639	0	0
MCPH1	0.0355249	0	0.0355249
MCRS1	0.0236946	0.0236946	0
MCTP1	0.3648065	0	0.3648065
MCTP2	0.2084714	0	0.2084714
MCTS1	0.0371824	0.0371824	0
MDFI	0.0077024	0.0077024	0
MDFIC	0.2030827	0	0.2030827
MDGA1	0.0069538	0	0.0069538
MDGA2	0.0064762	0	0.0064762
MDH1	0.1976323	0	0
MDH1B	0.0553179	0	0
MDH2	0.0256683	0.0256683	0
MDK	0.0029023	0.0029023	0
MDM1	0.0461121	0	0.0461121
MDM2	0.1513598	0	0
MDM4	0.8106988	0.8106988	0

MDN1	0.0307778	0	0.0307778
MDP1	0.0819461	0.0819461	0
MDS2	0.024443	0	0.024443
ME1	0.0462329	0	0.0462329
ME2	0.1263033	0	0.1263033
ME3	0.0300629	0	0
MEA1	0.0159079	0	0
MEAF6	0.0236567	0	0
MECOM	0.1310376	0.1310376	0
MECP2	0.0059196	0	0.0059196
MECR	0.0250741	0.0250741	0
MED1	0.0304136	0	0.0304136
MED10	0.0637193	0.0637193	0
MED11	0.0520663	0	0
MED12	0.0161582	0	0
MED12L	0.0057305	0	0.0057305
MED13	0.0671225	0	0.0671225
MED13L	0.0502777	0	0.0502777
MED14	0.0421525	0	0
MED15	0.0366151	0.0366151	0
MED16	1	1	0
MED17	0.0894873	0	0.0894873
MED18	0.0484891	0	0
MED19	0.0246566	0.0246566	0
MED20	0.06736	0	0
MED21	0.1152786	0.1152786	0
MED22	0.0497181	0	0
MED23	0.0382701	0	0.0382701
MED24	0.0130375	0.0130375	0
MED25	0.0695496	0.0695496	0
MED26	0.0313612	0.0313612	0
MED27	0.0670562	0	0
MED28	0.0232406	0	0.0232406
MED29	0.1035715	0.1035715	0
MED30	0.1222223	0.1222223	0
MED31	0.0514781	0	0.0514781
MED4	0.0203133	0	0
MED6	0.0459792	0	0
MED7	0.1539906	0.1539906	0
MED8	0.0468151	0	0
MED9	0.0175331	0	0
MEF2A	0.114802	0	0.114802
MEF2B	0.0050897	0	0
MEF2C	0.0916966	0	0.0916966
MEF2D	0.3090512	0	0.3090512

MEFV	0.0958097	0	0.0958097
MEG3	0.0007335	0	0
MEG8	0.0018624	0	0
MEGF10	0.0200189	0	0
MEGF11	0.00724	0	0
MEGF6	0.0022303	0	0.0022303
MEGF8	0.0075764	0	0.0075764
MEGF9	0.1024314	0	0.1024314
MEI1	0.0044868	0	0.0044868
MEIG1	0.0323793	0	0
MEIS1	0.0361688	0	0
MEIS2	0.0439311	0	0.0439311
MEIS3	0.0023252	0	0.0023252
MEIS3P1	0.0008361	0	0.0008361
MELK	0.0282836	0.0282836	0
MEMO1	0.0927113	0	0
MEN1	0.0142484	0.0142484	0
MEOX1	0.0215355	0	0.0215355
MEOX2	0.0038252	0	0.0038252
MEP1A	0.0144642	0	0.0144642
MEP1B	0.0080925	0	0
MEPCE	0.0820211	0.0820211	0
MEPE	0.0156322	0	0.0156322
MERTK	0.0113035	0	0.0113035
MESDC1	0.0415233	0	0
MESDC2	0.0571576	0	0
MESP1	0.0042729	0.0042729	0
MESP2	0.0224098	0	0
MEST	0.0240831	0	0.0240831
MESTIT1	0.0176112	0	0.0176112
MET	0.391801	0.391801	0
METAP1	0.0293751	0.0293751	0
METAP2	0.0579335	0	0.0579335
METRN	0.0041954	0.0041954	0
METRNL	0.0026998	0	0
METT10D	0.0281177	0	0.0281177
METT11D1	0.0602296	0.0602296	0
METT5D1	0.2125735	0.2125735	0
METTL1	0.0332376	0	0
METTL10	0.0137132	0.0137132	0
METTL11A	0.0446763	0.0446763	0
METTL11B	0.0233326	0	0
METTL12	0.0726717	0.0726717	0
METTL13	0.1294929	0	0
METTL14	0.0362015	0	0.0362015

METTL2A	0.0111292	0.0111292	0
METTL2B	0.0165767	0.0165767	0
METTL3	0.0688406	0.0688406	0
METTL4	0.0865204	0	0
METTL5	0.0369575	0.0369575	0
METTL6	0.0338027	0	0
METTL7A	0.071352	0	0.071352
METTL7B	0.3503909	0.3503909	0
METTL8	0.0526231	0	0.0526231
METTL9	0.2382593	0	0
MEX3A	0.0170731	0	0.0170731
MEX3B	0.006112	0	0.006112
MEX3C	0.083493	0	0.083493
MEX3D	0.0006993	0.0006993	0
MFAP1	0.1622284	0	0.1622284
MFAP2	0.0034264	0	0.0034264
MFAP3	0.0880734	0	0.0880734
MFAP3L	0.0109234	0	0.0109234
MFAP4	0.0081909	0	0.0081909
MFAP5	0.0284505	0	0.0284505
MFF	0.049945	0.049945	0
MFGE8	0.0480429	0	0.0480429
MFHAS1	0.085014	0	0.085014
MFI2	0.0347752	0	0
MFN1	0.0594704	0	0
MFN2	0.0270748	0	0.0270748
MFNG	0.0044426	0	0.0044426
MFRP	0.0019013	0	0.0019013
MFSD1	0.1088182	0	0.1088182
MFSD10	0.0191752	0.0191752	0
MFSD11	0.174406	0	0
MFSD2A	0.0232714	0.0232714	0
MFSD2B	0.0102428	0.0102428	0
MFSD3	0.0047318	0.0047318	0
MFSD4	0.0131971	0.0131971	0
MFSD5	0.0218744	0.0218744	0
MFSD6	0.0305134	0.0305134	0
MFSD6L	0.015312	0	0
MFSD7	0.0024823	0	0
MFSD8	0.0289963	0.0289963	0
MFSD9	0.0430268	0.0430268	0
MGA	0.0608201	0	0.0608201
MGAM	0.00572	0	0.00572
MGAT1	0.1479561	0	0.1479561
MGAT2	0.1398472	0	0.1398472

MGAT3	0.0167507	0	0
MGAT4A	0.0319197	0	0.0319197
MGAT4B	0.0051861	0.0051861	0
MGAT4C	0.0077202	0	0.0077202
MGAT5	0.0530719	0	0.0530719
MGAT5B	0.0039174	0	0.0039174
MGC12916	0.0057111	0	0.0057111
MGC12982	0.0096598	0.0096598	0
MGC14436	0.0084636	0.0084636	0
MGC15885	0.0178232	0	0.0178232
MGC16025	0.0010242	0	0
MGC16121	0.0167778	0	0.0167778
MGC16142	0.0195501	0	0
MGC16275	0.0334006	0	0.0334006
MGC16384	0.1561406	0	0
MGC16703	0.0019755	0	0.0019755
MGC23270	0.0043429	0	0
MGC23284	0.0040049	0	0.0040049
MGC26647	0.0164913	0	0
MGC27382	0.2737128	0	0
MGC2752	0.0485574	0	0
MGC2889	0.0108955	0.0108955	0
MGC29506	0.0636087	0	0.0636087
MGC34034	0.0114388	0	0.0114388
MGC3771	0.0084225	0	0
MGC42105	0.0347675	0	0.0347675
MGC4473	0.064918	0	0.064918
MGC45800	0.0126666	0	0.0126666
MGC57346	0.0002433	0.0002433	0
MGC70857	0.004444	0.004444	0
MGC72080	0.0280785	0.0280785	0
MGC87042	0.0054536	0	0.0054536
MGEA5	0.1089225	0	0
MGLL	0.1016448	0.1016448	0
MGMT	0.0040281	0.0040281	0
MGP	0.0186019	0	0.0186019
MGRN1	0.0422082	0.0422082	0
MGST1	0.063715	0.063715	0
MGST2	0.0313382	0.0313382	0
MGST3	0.178112	0.178112	0
MIA	0.0253116	0	0
MIA2	0.0182611	0	0
MIA3	0.0655447	0	0
MIAT	0.1149014	0	0.1149014
MIB1	0.0309323	0	0.0309323

MIB2	0.0036019	0.0036019	0
MICAL1	0.0263397	0	0.0263397
MICAL2	0.0139966	0	0.0139966
MICAL3	0.0490452	0.0490452	0
MICALCL	0.0804017	0	0
MICALL1	0.0154384	0	0
MICALL2	0.0020807	0.0020807	0
MID1	0.072826	0	0.072826
MID1IP1	0.2350806	0.2350806	0
MID2	0.005853	0	0.005853
MIDN	0.2437505	0.2437505	0
MIER1	0.2211605	0	0.2211605
MIER2	0.0053412	0.0053412	0
MIER3	0.0161871	0	0
MIF	0.0300455	0.0300455	0
MIF4GD	0.0181841	0	0.0181841
MIIP	0.3891535	0	0
MIMT1	0.0020095	0	0
MINA	0.086814	0	0.086814
MINK1	0.0074568	0.0074568	0
MINPP1	0.0749854	0	0
MIOS	0.1508064	0	0.1508064
MIOX	0.000796	0	0
MIP	0.0214025	0	0
MIPEP	0.0184924	0.0184924	0
MIPOL1	0.0150074	0.0150074	0
MIR155HG	0.1427844	0	0.1427844
MIR17HG	0.1115065	0.1115065	0
MIS12	0.0542935	0	0.0542935
MITD1	0.1788917	0	0
MITF	0.0522471	0	0.0522471
MIXL1	0.0163561	0	0.0163561
MKI67	0.0477901	0.0477901	0
MKI67IP	0.0094647	0	0
MKKS	0.1654562	0.1654562	0
MKL1	0.1490973	0	0.1490973
MKL2	0.0159803	0	0.0159803
MKLN1	0.3307319	0	0.3307319
MKNK1	0.0257113	0	0.0257113
MKNK2	0.1033801	0.1033801	0
MKRN1	0.0301613	0.0301613	0
MKRN2	0.0971622	0	0.0971622
MKRN3	0.0029705	0	0.0029705
MKS1	0.0357562	0.0357562	0
MKX	0.0028772	0	0.0028772

MLANA	0.0115695	0	0
MLC1	0.0019748	0	0
MLEC	0.1265306	0.1265306	0
MLF1	0.0140046	0	0
MLF1IP	0.1295587	0.1295587	0
MLF2	0.0507257	0.0507257	0
MLH1	0.1261887	0	0.1261887
MLH3	0.1278517	0.1278517	0
MLKL	0.1920923	0	0
MLL	0.0398007	0	0.0398007
MLL2	0.0364246	0	0
MLL3	0.0202403	0	0
MLL4	0.0212034	0.0212034	0
MLL5	0.445468	0	0.445468
MLLT1	0.0188953	0	0
MLLT10	0.0570834	0	0.0570834
MLLT11	0.1183914	0	0.1183914
MLLT3	0.1281798	0	0
MLLT4	0.0377773	0.0377773	0
MLLT6	0.0616014	0	0
MLN	0.0018869	0	0
MLNR	0.0069416	0	0.0069416
MLPH	0.0055726	0.0055726	0
MLST8	0.007171	0.007171	0
MLX	0.0326161	0.0326161	0
MLXIP	0.0633905	0	0.0633905
MLXIPL	0.0074059	0.0074059	0
MLYCD	0.0045861	0.0045861	0
MMAA	0.0703992	0	0
MMAB	0.0309902	0.0309902	0
MMACHC	0.0933261	0.0933261	0
MMADHC	0.0592808	0	0
MMD	0.0987218	0	0.0987218
MMD2	0.0004928	0	0
MME	0.018851	0	0.018851
MMEL1	0.000742	0.000742	0
MMGT1	0.012514	0	0.012514
MMP1	0.0202326	0	0
MMP10	0.0325164	0	0.0325164
MMP11	0.00721	0	0.00721
MMP12	0.0437313	0	0.0437313
MMP13	0.0207804	0	0.0207804
MMP14	0.0130183	0	0.0130183
MMP15	0.0055241	0.0055241	0
MMP16	0.0139108	0	0.0139108

MMP17	0.0012487	0	0.0012487
MMP19	0.1567198	0	0.1567198
MMP2	0.0162148	0	0.0162148
MMP20	0.0080729	0	0.0080729
MMP21	0.0042808	0	0.0042808
MMP23B	0.0001039	0	0.0001039
MMP24	0.0104664	0.0104664	0
MMP25	0.004463	0	0.004463
MMP26	0.0111299	0.0111299	0
MMP27	0.008618	0	0.008618
MMP28	0.0330037	0.0330037	0
MMP3	0.1143272	0	0.1143272
MMP7	0.0361671	0	0.0361671
MMP8	0.014173	0	0.014173
MMP9	0.0113972	0	0.0113972
MMRN1	0.0181962	0	0.0181962
MMRN2	0.0071906	0	0.0071906
MMS19	0.0558027	0.0558027	0
MN1	0.0020799	0	0.0020799
MNAT1	0.0570777	0.0570777	0
MND1	0.0570911	0.0570911	0
MNDA	0.0559363	0	0.0559363
MNS1	0.0289093	0	0
MNT	0.2409716	0.2409716	0
MNX1	0.0033309	0.0033309	0
MOAP1	0.0217408	0	0
MOB2	0.0069188	0.0069188	0
MOBKL1A	0.006705	0	0.006705
MOBKL1B	0.0861923	0	0.0861923
MOBKL2A	0.1031922	0	0.1031922
MOBKL2B	0.1350045	0	0.1350045
MOBKL2C	0.0197584	0	0.0197584
MOBKL3	0.0268252	0	0
MOBP	0.0056338	0	0.0056338
MOCOS	0.139573	0.139573	0
MOCS1	0.0087467	0	0.0087467
MOCS2	0.0424399	0	0.0424399
MOCS3	0.0650775	0.0650775	0
MOGAT1	0.0055961	0	0
MOGAT2	0.0096944	0	0
MOGAT3	0.001073	0.001073	0
MOGS	0.0306273	0.0306273	0
MON1A	0.0096752	0.0096752	0
MON1B	0.5881786	0	0
MON2	0.0383438	0	0

MORC1	0.004868	0	0
MORC2	0.0347758	0.0347758	0
MORC3	0.0873942	0	0.0873942
MORC4	0.0122865	0	0
MORF4	0.0007245	0	0.0007245
MORF4L1	0.0812076	0	0.0812076
MORF4L2	0.0836547	0	0
MORN1	0.0227172	0.0227172	0
MORN2	0.0241616	0.0241616	0
MORN3	0.0642061	0	0
MORN4	0.0266736	0	0
MORN5	0.0369137	0	0
MOS	0.0033013	0.0033013	0
MOSC1	0.0735634	0.0735634	0
MOSC2	0.0203587	0	0
MOSPD1	0.0120264	0	0.0120264
MOSPD2	0.027872	0	0.027872
MOSPD3	0.0141417	0.0141417	0
MOV10	0.1577512	0	0
MOV10L1	0.0030808	0	0.0030808
MOXD1	0.0216996	0	0.0216996
MPDU1	0.0973238	0.0973238	0
MPDZ	0.0075798	0	0.0075798
MPEG1	0.1800092	0	0.1800092
MPG	0.0051176	0.0051176	0
MPHOSPH10	0.0721953	0	0.0721953
MPHOSPH6	0.0280211	0	0.0280211
MPHOSPH8	0.0272911	0	0.0272911
MPHOSPH9	0.0134835	0	0.0134835
MPI	0.0197287	0	0
MPL	0.006877	0	0
MPND	0.0138078	0.0138078	0
MPO	0.0029887	0	0.0029887
MPP1	0.0271694	0	0.0271694
MPP2	0.0039053	0	0.0039053
MPP3	0.007921	0.007921	0
MPP4	0.0088831	0	0
MPP5	0.068316	0.068316	0
MPP6	0.0702678	0	0.0702678
MPP7	0.0091945	0	0
MPPE1	0.0171581	0.0171581	0
MPPED1	0.0010526	0	0.0010526
MPPED2	0.0103501	0	0.0103501
MPRIP	0.0341946	0	0.0341946
MPST	0.0022131	0.0022131	0

MPV17	0.5601042	0	0.5601042
MPV17L	0.0015281	0	0
MPV17L2	0.0575617	0.0575617	0
MPZ	0.0317898	0	0.0317898
MPZL1	0.8368692	0	0.8368692
MPZL2	0.1181325	0.1181325	0
MPZL3	0.1167995	0	0
MR1	0.3288697	0	0.3288697
MRAP	0.0057152	0	0.0057152
MRAP2	0.0057139	0.0057139	0
MRAS	0.0061224	0	0.0061224
MRC2	0.0041638	0	0.0041638
MRE11A	0.077664	0	0.077664
MREG	0.5157486	0.5157486	0
MRFAP1	0.0700513	0	0
MRFAP1L1	0.0461257	0	0
MRGPRD	0.0036002	0	0
MRGPRE	0.0020194	0	0.0020194
MRGPRF	0.0015276	0	0.0015276
MRGPRG	0.0015366	0	0
MRGPRX1	0.009649	0	0
MRGPRX2	0.0046995	0	0.0046995
MRGPRX3	0.0015502	0	0
MRGPRX4	0.008559	0	0
MRI1	0.0065726	0.0065726	0
MRM1	0.0161201	0.0161201	0
MRO	0.0183443	0	0.0183443
MRP63	0.0172252	0.0172252	0
MRPL1	0.0938882	0	0
MRPL10	0.021064	0.021064	0
MRPL11	0.0671411	0.0671411	0
MRPL12	0.0119846	0.0119846	0
MRPL13	0.1552669	0.1552669	0
MRPL14	0.0275855	0.0275855	0
MRPL15	0.0449672	0.0449672	0
MRPL16	0.0490935	0.0490935	0
MRPL17	0.0677849	0.0677849	0
MRPL18	0.1097292	0	0
MRPL19	0.0624067	0	0
MRPL2	0.0194849	0.0194849	0
MRPL20	0.019195	0.019195	0
MRPL21	0.0191498	0.0191498	0
MRPL22	0.1045995	0.1045995	0
MRPL23	0.0010103	0.0010103	0
MRPL24	0.5125212	0.5125212	0

MRPL27	0.0276748	0.0276748	0
MRPL28	0.003454	0.003454	0
MRPL3	0.0610048	0.0610048	0
MRPL30	0.1664366	0.1664366	0
MRPL32	0.1190433	0.1190433	0
MRPL33	0.043861	0	0
MRPL34	0.1363467	0.1363467	0
MRPL35	0.0834137	0	0
MRPL36	0.1556987	0.1556987	0
MRPL37	0.0160308	0.0160308	0
MRPL38	0.009332	0.009332	0
MRPL39	0.0324294	0	0
MRPL4	0.0256252	0.0256252	0
MRPL40	0.0444633	0.0444633	0
MRPL41	0.0066032	0.0066032	0
MRPL42	0.0476052	0	0
MRPL42P5	0.0066593	0	0
MRPL43	0.0073458	0.0073458	0
MRPL44	0.0850487	0.0850487	0
MRPL45	0.0018526	0.0018526	0
MRPL46	0.1826745	0.1826745	0
MRPL47	0.0959766	0.0959766	0
MRPL48	0.3995368	0.3995368	0
MRPL49	0.0647235	0.0647235	0
MRPL50	0.0812185	0	0
MRPL51	0.0519407	0.0519407	0
MRPL52	0.0437936	0.0437936	0
MRPL53	0.0221991	0.0221991	0
MRPL54	0.0223008	0.0223008	0
MRPL55	0.027428	0.027428	0
MRPL9	0.1092555	0.1092555	0
MRPS10	0.2009537	0	0
MRPS11	0.1709865	0.1709865	0
MRPS12	0.0251955	0.0251955	0
MRPS14	0.1462541	0.1462541	0
MRPS15	0.0364169	0.0364169	0
MRPS16	0.2287691	0.2287691	0
MRPS17	0.0078719	0.0078719	0
MRPS18A	0.0580351	0.0580351	0
MRPS18C	0.0625983	0	0
MRPS2	0.0111951	0.0111951	0
MRPS21	0.0409741	0.0409741	0
MRPS22	0.032097	0	0
MRPS23	0.0553887	0.0553887	0
MRPS24	0.122466	0.122466	0

MRPS25	0.019695	0.019695	0
MRPS26	0.0064032	0.0064032	0
MRPS27	0.077284	0.077284	0
MRPS28	0.1091587	0.1091587	0
MRPS30	0.4440191	0.4440191	0
MRPS31	0.0632077	0	0
MRPS33	0.1178684	0.1178684	0
MRPS34	0.006956	0.006956	0
MRPS35	0.0637467	0.0637467	0
MRPS5	0.0352491	0.0352491	0
MRPS6	0.0501102	0.0501102	0
MRPS7	0.0260046	0.0260046	0
MRPS9	0.0329123	0.0329123	0
MRRF	0.1492055	0.1492055	0
MRS2	0.0444531	0	0
MRS2P2	0.0213296	0.0213296	0
MRTO4	0.0133622	0	0
MRVI1	0.0380442	0	0.0380442
MS4A1	0.0996271	0	0.0996271
MS4A10	0.005888	0	0
MS4A12	0.0144671	0	0
MS4A13	0.0108514	0	0
MS4A14	0.0239781	0	0.0239781
MS4A15	0.0491184	0.0491184	0
MS4A2	0.0153042	0	0.0153042
MS4A3	0.0077186	0	0.0077186
MS4A4A	0.0436521	0	0.0436521
MS4A5	0.0083911	0	0
MS4A6A	0.0272592	0	0.0272592
MS4A6E	0.0283247	0	0.0283247
MS4A7	0.0478766	0	0.0478766
MS4A8B	0.0114594	0.0114594	0
MSC	0.1793776	0	0.1793776
MSGN1	0.0406364	0	0
MSH2	0.0151743	0	0
MSH3	0.0357719	0	0
MSH4	0.063522	0	0
MSH6	0.0289406	0	0.0289406
MSI1	0.0018549	0	0.0018549
MSI2	0.0421557	0	0
MSL1	0.0427514	0.0427514	0
MSL2	0.1349616	0	0.1349616
MSL3	0.099054	0	0.099054
MSL3L2	0.0123916	0.0123916	0
MSLN	0.0049665	0.0049665	0

MSLNL	0.0008036	0.0008036	0
MSMB	0.0043753	0.0043753	0
MSMP	0.0235658	0	0
MSN	0.0426593	0	0.0426593
MSR1	0.0065082	0	0.0065082
MSRA	0.0393877	0	0
MSRB2	0.024742	0.024742	0
MSRB3	0.0149571	0	0.0149571
MST1	0.0047494	0.0047494	0
MST1P2	0.0732253	0.0732253	0
MST1P9	0.0035519	0.0035519	0
MST1R	0.0174792	0.0174792	0
MST4	0.0320442	0	0
MSTN	0.0136967	0	0
MSTO1	0.0075232	0.0075232	0
MSTO2P	0.0135831	0.0135831	0
MSX1	0.0009652	0	0.0009652
MSX2	0.0074508	0	0
MSX2P1	0.0064235	0	0.0064235
MT1A	0.043534	0	0.043534
MT1B	0.1127484	0	0.1127484
MT1DP	0.0427479	0	0
MT1E	0.0734334	0	0
MT1F	0.1168474	0	0
MT1G	0.0083119	0	0
MT1H	0.006842	0	0.006842
MT1IP	0.0573888	0	0
MT1L	0.5340684	0	0.5340684
MT1M	0.0725982	0	0.0725982
MT1X	0.3496987	0	0.3496987
MT2A	0.8263032	0	0.8263032
MT3	0.004293	0.004293	0
MT4	0.0060561	0.0060561	0
MTA1	0.0012273	0.0012273	0
MTA2	0.0704487	0	0
MTA3	0.0223522	0.0223522	0
MTAP	0.0126492	0	0.0126492
MTBP	0.1655579	0	0
MTCH1	0.0149256	0	0
MTCH2	0.0552763	0.0552763	0
MTCP1	0.0446523	0.0446523	0
MTCP1NB	0.0446523	0	0
MTDH	0.1306189	0	0.1306189
MTERF	0.0361076	0.0361076	0
MTERFD1	0.2215331	0	0

MTERFD2	0.0393116	0	0.0393116
MTERFD3	0.0492936	0.0492936	0
MTF1	0.1023728	0	0.1023728
MTF2	0.0971113	0	0.0971113
MTFMT	0.0559753	0.0559753	0
MTFR1	0.0545897	0.0545897	0
MTG1	0.0160086	0.0160086	0
MTHFD1	0.0448144	0.0448144	0
MTHFD1L	0.4704695	0	0.4704695
MTHFD2	0.1864968	0	0.1864968
MTHFD2L	0.0770974	0.0770974	0
MTHFR	0.1380064	0	0.1380064
MTHFS	0.6649738	0.6649738	0
MTHFSD	0.0148288	0.0148288	0
MTIF2	0.0326181	0.0326181	0
MTIF3	0.360153	0.360153	0
MTL5	0.0055151	0.0055151	0
MTM1	0.0444754	0	0.0444754
MTMR1	0.0302857	0	0
MTMR10	0.0206984	0	0.0206984
MTMR11	0.3904911	0.3904911	0
MTMR12	0.0642502	0	0.0642502
MTMR14	0.0174095	0	0
MTMR15	0.0286965	0.0286965	0
MTMR2	0.1887252	0	0.1887252
MTMR3	0.0153706	0	0
MTMR4	0.0901562	0	0
MTMR6	0.0227515	0	0.0227515
MTMR7	0.0098132	0	0.0098132
MTMR8	0.0026388	0	0
MTMR9	0.0823696	0	0.0823696
MTMR9L	0.0447789	0	0
MTNR1A	0.0024947	0	0.0024947
MTNR1B	0.0086443	0	0.0086443
MTO1	0.3369276	0	0.3369276
MTOR	0.0149865	0	0.0149865
MTP18	0.069428	0.069428	0
MTPAP	0.0290092	0	0
MTR	0.1635326	0	0.1635326
MTRF1	0.0327048	0.0327048	0
MTRF1L	0.2051191	0	0.2051191
MTRR	0.4705884	0	0.4705884
MTSS1	0.0162546	0	0
MTSS1L	0.0009608	0	0
MTTP	0.0801197	0	0

MTUS1	0.4239519	0	0
MTUS2	0.0568491	0	0
MTVR2	0.0147079	0	0
MTX1	0.0257674	0.0257674	0
MTX2	0.0172407	0.0172407	0
MTX3	0.0463578	0	0.0463578
MUC1	0.8782961	0.8782961	0
MUC12	0.0015097	0	0
MUC13	0.02454	0.02454	0
MUC15	0.1034409	0	0
MUC16	0.0017184	0	0.0017184
MUC17	0.0024543	0	0
MUC2	0.0010649	0	0
MUC20	0.0066586	0.0066586	0
MUC4	0.0016953	0	0
MUC5B	0.0003599	0	0
MUC6	0.0003372	0	0
MUC7	0.003452	0	0
MUCL1	0.0290194	0	0
MUDENG	0.0689444	0	0
MUL1	0.0104421	0	0
MUM1	0.0120176	0.0120176	0
MUM1L1	0.0062364	0	0.0062364
MURC	0.0146132	0	0.0146132
MUS81	0.1240182	0.1240182	0
MUSK	0.0203431	0	0.0203431
MUSTN1	0.0228897	0	0.0228897
MUT	0.0762495	0	0
MUTED	0.0676592	0	0.0676592
MUTYH	0.0465416	0.0465416	0
MVD	0.0040269	0.0040269	0
MVK	0.0320679	0.0320679	0
MVP	0.9028528	0.9028528	0
MX1	0.1020857	0	0.1020857
MX2	0.0480779	0	0.0480779
MXD1	0.0879657	0	0
MXD3	0.0580358	0.0580358	0
MXD4	0.0012668	0	0
MXI1	0.1934434	0.1934434	0
MXRA5	0.0014978	0	0.0014978
MXRA7	0.0304456	0	0.0304456
MXRA8	0.0010728	0	0.0010728
MYADM	0.00574	0	0.00574
MYADML	0.0045011	0	0
MYADML2	0.0010511	0	0

MYB	0.054647	0	0.054647
MYBBP1A	0.0099541	0	0
MYBL1	0.0541725	0	0.0541725
MYBL2	0.0295903	0.0295903	0
MYBPC1	0.0320672	0	0
MYBPC2	0.0112048	0	0.0112048
MYBPC3	0.0060298	0	0.0060298
MYBPH	0.0094883	0	0.0094883
MYBPHL	0.0081926	0	0
MYC	1	0	1
MYCBP	0.017166	0.017166	0
MYCBP2	0.1976531	0	0.1976531
MYCBPAP	0.0287129	0	0.0287129
MYCL1	0.0561843	0	0.0561843
MYCN	0.0023965	0.0023965	0
MYCNOS	0.0020607	0.0020607	0
MYCT1	0.0073988	0	0.0073988
MYD88	0.0680587	0	0
MYEF2	0.0300965	0	0
MYEOV	0.0941615	0.0941615	0
MYEOV2	0.0016569	0.0016569	0
MYF5	0.0085174	0	0.0085174
MYF6	0.0899099	0	0
MYH1	0.0065669	0	0.0065669
MYH10	0.0067192	0	0.0067192
MYH11	0.0058998	0	0.0058998
MYH13	0.0048472	0	0.0048472
MYH14	0.0019104	0.0019104	0
MYH15	0.0266376	0	0.0266376
MYH16	0.0115242	0.0115242	0
MYH2	0.0055484	0	0.0055484
MYH3	0.0020019	0	0
MYH4	0.0051114	0	0
MYH6	0.0013455	0	0.0013455
MYH7	0.0023121	0	0
MYH7B	0.0662314	0.0662314	0
MYH8	0.0075172	0	0.0075172
MYH9	0.0957744	0	0.0957744
MYL1	0.0088005	0	0
MYL10	0.0019324	0	0.0019324
MYL12A	0.3120871	0	0
MYL12B	0.185014	0.185014	0
MYL2	0.0171316	0	0.0171316
MYL3	0.0017507	0	0.0017507
MYL4	0.0159147	0	0.0159147

MYL5	0.0067267	0.0067267	0
MYL6	0.0853917	0	0
MYL6B	0.0950369	0	0
MYL7	0.0012577	0	0
MYL9	0.0020053	0	0.0020053
MYLIP	0.1093783	0	0.1093783
MYLK	0.0146955	0	0.0146955
MYLK2	0.0033838	0	0.0033838
MYLK3	0.0032056	0	0.0032056
MYLK4	0.0245686	0	0.0245686
MYLPF	0.0170961	0	0
MYNN	0.4258674	0	0.4258674
MYO10	0.116788	0.116788	0
MYO15A	0.0012597	0.0012597	0
MYO15B	0.0012883	0.0012883	0
MYO16	0.0121318	0	0.0121318
MYO18A	0.1476425	0.1476425	0
MYO18B	0.0151237	0	0.0151237
MYO19	0.0665748	0.0665748	0
MYO1A	0.0120694	0.0120694	0
MYO1B	0.0282824	0	0.0282824
MYO1C	0.0086207	0	0
MYO1D	0.0265466	0	0
MYO1E	0.1638607	0	0.1638607
MYO1F	0.014093	0	0.014093
MYO1G	0.6845688	0	0.6845688
MYO1H	0.0208564	0	0.0208564
MYO3A	0.002696	0	0
MYO3B	0.0397935	0	0.0397935
MYO5A	0.0301323	0	0.0301323
MYO5B	0.0310525	0.0310525	0
MYO5C	0.0295071	0.0295071	0
MYO6	0.0487767	0.0487767	0
MYO7A	0.003189	0	0
MYO7B	0.0354428	0	0
MYO9A	0.0587777	0	0.0587777
MYO9B	0.0506878	0	0.0506878
MYOC	0.0192293	0	0.0192293
MYOCD	0.0234812	0	0.0234812
MYOD1	0.0023217	0	0
MYOF	0.2258909	0	0.2258909
MYOG	0.0107472	0	0
MYOM1	0.0307439	0	0
MYOM2	0.0064903	0	0
MYOM3	0.0047307	0	0

MYOT	0.0248854	0	0.0248854
MYOZ1	0.0092975	0	0.0092975
MYOZ2	0.0122951	0	0.0122951
MYOZ3	0.0089013	0	0.0089013
MYPN	0.1416173	0.1416173	0
MYPOP	0.003727	0.003727	0
MYRIP	0.0142469	0	0.0142469
MYSM1	0.0187455	0	0.0187455
MYST1	0.0360689	0.0360689	0
MYST2	0.0530111	0	0
MYST3	0.1343694	0	0.1343694
MYST4	0.0475541	0	0.0475541
MYT1	0.0013994	0	0.0013994
MYT1L	0.0033067	0	0.0033067
MZF1	0.0591933	0.0591933	0
N4BP1	0.0877007	0	0
N4BP2	0.0871253	0	0.0871253
N4BP2L1	0.0256657	0	0.0256657
N4BP2L2	0.0926474	0	0
N4BP3	0.0136517	0.0136517	0
N6AMT1	0.2777356	0.2777356	0
N6AMT2	0.0246519	0	0
NAA10	0.0023029	0.0023029	0
NAA11	0.0062172	0.0062172	0
NAA15	0.4072758	0	0
NAA16	0.0389938	0	0
NAA20	0.0182932	0.0182932	0
NAA25	0.0775667	0	0
NAA30	0.0640971	0	0
NAA35	0.0870403	0.0870403	0
NAA38	0.1063561	0.1063561	0
NAA40	0.0206947	0.0206947	0
NAA50	0.3402009	0	0.3402009
NAAA	0.0085543	0	0.0085543
NAALAD2	0.0164095	0	0.0164095
NAALADL1	0.0060585	0	0.0060585
NAALADL2	0.046212	0	0
NAB1	0.0907057	0.0907057	0
NAB2	0.1188358	0	0.1188358
NACA	0.0783109	0.0783109	0
NACA2	0.0156458	0.0156458	0
NACAD	0.0029034	0	0.0029034
NACAP1	0.05307	0.05307	0
NACC1	0.024948	0.024948	0
NACC2	0.0030804	0	0.0030804

NADK	0.0260858	0	0.0260858
NADSYN1	0.0277956	0.0277956	0
NAE1	0.0280802	0	0
NAF1	0.0965363	0	0.0965363
NAGA	0.0155793	0	0.0155793
NAGK	0.0477289	0	0.0477289
NAGLU	0.0495767	0	0
NAGPA	0.0082202	0.0082202	0
NAGS	0.0088328	0.0088328	0
NAIF1	0.0942768	0.0942768	0
NALCN	0.0089351	0	0.0089351
NAMPT	0.8864841	0	0.8864841
NANOG	0.0030773	0.0030773	0
NANOS1	0.0316884	0	0
NANOS2	0.0021073	0.0021073	0
NANOS3	0.0153717	0.0153717	0
NANP	0.0453184	0	0
NANS	0.0807043	0.0807043	0
NAP1L1	0.0743415	0	0.0743415
NAP1L2	0.0063541	0	0.0063541
NAP1L3	0.0062112	0	0.0062112
NAP1L4	0.0885656	0.0885656	0
NAP1L5	0.0144882	0	0.0144882
NAP1L6	0.0017049	0	0.0017049
NAPA	0.0277898	0.0277898	0
NAPB	0.0100751	0	0
NAPEPLD	0.0753651	0.0753651	0
NAPG	0.1120229	0.1120229	0
NAPRT1	0.0223107	0.0223107	0
NAPSA	0.0783748	0	0
NAPSB	0.0601789	0	0.0601789
NARF	0.4463255	0.4463255	0
NARFL	0.0041304	0.0041304	0
NARG2	0.1844379	0	0.1844379
NARS	0.0375669	0	0.0375669
NARS2	0.058363	0	0
NASP	0.0377144	0	0.0377144
NAT1	0.0241838	0	0
NAT10	0.1991444	0.1991444	0
NAT14	0.003828	0.003828	0
NAT15	0.0154106	0.0154106	0
NAT2	0.0099342	0	0
NAT6	0.0177927	0.0177927	0
NAT8	0.0031827	0	0
NAT8B	0.0043346	0	0.0043346

NAT8L	0.0004195	0	0.0004195
NAT9	0.0149267	0.0149267	0
NAV1	0.293441	0	0.293441
NAV2	0.0412185	0	0
NAV3	0.0184267	0	0.0184267
NBAS	0.0489719	0	0
NBEA	0.0090644	0	0.0090644
NBEAL1	0.0463328	0	0.0463328
NBEAL2	0.0313113	0.0313113	0
NBL1	0.239483	0	0.239483
NBLA00301	0.0007614	0	0.0007614
NBN	0.2246917	0	0.2246917
NBPF1	0.8707465	0	0.8707465
NBPF10	0.0723604	0	0.0723604
NBPF14	2.00E-05	0	2.00E-05
NBPF15	0.0575029	0	0
NBPF22P	0.0005533	0.0005533	0
NBPF3	0.0035321	0	0
NBPF4	1.53E-05	0	0
NBPF6	1.79E-05	1.79E-05	0
NBPF7	0.0171939	0.0171939	0
NBPF9	4.00E-05	0	4.00E-05
NBR1	0.2013452	0	0
NBR2	0.0548456	0.0548456	0
NCALD	0.0636327	0	0.0636327
NCAM1	0.0041796	0	0.0041796
NCAM2	0.0078509	0	0.0078509
NCAN	0.0026558	0	0
NCAPD2	0.0490742	0	0
NCAPD3	0.0206108	0	0
NCAPG	0.1070465	0.1070465	0
NCAPG2	0.0205674	0	0
NCAPH	0.1122971	0	0
NCAPH2	0.0026303	0.0026303	0
NCBP1	0.2536104	0	0
NCBP2	0.0609856	0	0
NCCRP1	0.0020884	0	0.0020884
NCDN	0.0226074	0	0.0226074
NCEH1	0.1238502	0	0.1238502
NCF1	1.73E-05	0	1.73E-05
NCF1B	1.53E-05	0	1.53E-05
NCF1C	1.53E-05	0	1.53E-05
NCF2	0.1363582	0	0.1363582
NCF4	0.0355819	0	0.0355819
NCK1	0.0826329	0	0.0826329

NCK2	0.0307942	0	0
NCKAP1	0.0363658	0	0
NCKAP1L	0.0248783	0	0.0248783
NCKAP5	0.0104094	0	0.0104094
NCKAP5L	0.035099	0	0.035099
NCKIPSD	0.0097061	0.0097061	0
NCL	0.0292364	0	0
NCLN	0.0239628	0.0239628	0
NCOA1	0.0521833	0	0.0521833
NCOA2	0.036312	0	0.036312
NCOA3	0.2595272	0	0.2595272
NCOA4	0.1812385	0	0.1812385
NCOA5	0.0791797	0	0
NCOA6	0.0320512	0.0320512	0
NCOA7	0.1920061	0	0.1920061
NCOR1	0.058509	0	0.058509
NCOR2	0.0087594	0.0087594	0
NCR1	0.0021963	0	0.0021963
NCR2	0.0089332	0	0
NCRNA00028	0.0050426	0	0.0050426
NCRNA00029	0.0033514	0	0
NCRNA00032	0.0193753	0	0
NCRNA00051	0.0010665	0	0
NCRNA00052	0.0156458	0	0
NCRNA00081	0.2885211	0	0
NCRNA00085	0.0020196	0.0020196	0
NCRNA00086	0.0025689	0	0
NCRNA00087	0.0027759	0	0.0027759
NCRNA00092	0.0060363	0	0.0060363
NCRNA00093	0.0241313	0	0
NCRNA00094	0.0081293	0	0.0081293
NCRNA00095	0.0440737	0.0440737	0
NCRNA00099	0.0047291	0	0
NCRNA00107	0.0001244	0	0
NCRNA00110	0.0058659	0	0.0058659
NCRNA00111	0.0502555	0.0502555	0
NCRNA00112	0.0076955	0	0
NCRNA00113	0.0076606	0	0
NCRNA00114	0.3095344	0	0
NCRNA00115	0.0027166	0.0027166	0
NCRNA00116	0.002193	0.002193	0
NCRNA00119	0.0446635	0	0
NCRNA00120	0.2328256	0	0.2328256
NCRNA00152	0.000358	0	0.000358
NCRNA00157	0.0095678	0	0

NCRNA00158	0.0395605	0	0.0395605
NCRNA00159	0.0060922	0	0
NCRNA00160	0.0134027	0	0
NCRNA00161	0.0244242	0	0.0244242
NCRNA00162	0.0091871	0	0
NCRNA00164	1	0	0
NCRNA00167	0.0412327	0.0412327	0
NCRNA00169	0.1477687	0	0
NCRNA00173	0.0840448	0	0.0840448
NCRNA00174	0.0075122	0.0075122	0
NCRNA00175	0.0008338	0	0
NCRNA00176	0.0064604	0.0064604	0
NCRNA00181	0.0018467	0	0.0018467
NCRNA00182	0.0471103	0	0
NCRNA00183	0.0587499	0	0
NCRNA00185	1.53E-05	1.53E-05	0
NCRNA00188	0.1061397	0.1061397	0
NCRNA00189	0.1410548	0	0.1410548
NCRNA00200	0.0012905	0	0.0012905
NCRNA00201	0.3040474	0.3040474	0
NCRNA00202	0.0056919	0	0
NCRNA00203	0.0235977	0	0
NCRNA00207	0.0206398	0	0
NCRNA00219	0.0605955	0.0605955	0
NCRNA00235	0.0067987	0.0067987	0
NCS1	0.0028409	0	0.0028409
NCSTN	0.098916	0.098916	0
NDC80	0.0906808	0	0
NDE1	0.4303129	0	0
NDEL1	0.0770975	0	0.0770975
NDFIP1	0.0972303	0.0972303	0
NDFIP2	0.2982169	0.2982169	0
NDN	0.0013237	0	0.0013237
NDNL2	0.0560673	0	0.0560673
NDOR1	0.016947	0.016947	0
NDP	0.0116247	0	0.0116247
NDRG1	0.5383979	0.5383979	0
NDRG2	0.0021051	0	0
NDRG3	0.0262527	0	0
NDRG4	0.0039569	0	0.0039569
NDST1	0.0158709	0	0.0158709
NDST2	0.0185443	0	0.0185443
NDST3	0.0056942	0.0056942	0
NDST4	0.0246608	0	0
NDUFA1	0.013565	0.013565	0

NDUFA10	0.0078432	0.0078432	0
NDUFA11	0.0068736	0.0068736	0
NDUFA12	0.0437696	0.0437696	0
NDUFA13	0.0092438	0.0092438	0
NDUFA2	0.1102813	0.1102813	0
NDUFA3	0.0038444	0.0038444	0
NDUFA4	0.0763841	0	0
NDUFA4L2	0.0080851	0	0.0080851
NDUFA5	0.0625356	0.0625356	0
NDUFA6	0.0169985	0	0
NDUFA7	0.016	0.016	0
NDUFA8	0.0384071	0.0384071	0
NDUFA9	0.1276802	0.1276802	0
NDUFAB1	0.0173002	0.0173002	0
NDUFAF1	0.0374362	0	0.0374362
NDUFAF2	0.0511874	0.0511874	0
NDUFAF3	0.0578387	0.0578387	0
NDUFAF4	0.0657077	0	0.0657077
NDUFB1	0.0291719	0.0291719	0
NDUFB10	0.0335422	0.0335422	0
NDUFB11	0.0213356	0.0213356	0
NDUFB2	0.0143744	0.0143744	0
NDUFB3	0.052658	0.052658	0
NDUFB5	0.1020835	0	0
NDUFB6	0.0779388	0.0779388	0
NDUFB7	0.0535331	0.0535331	0
NDUFB8	0.0689287	0.0689287	0
NDUFB9	0.1823668	0.1823668	0
NDUFC1	0.3900901	0.3900901	0
NDUFC2	0.0420315	0.0420315	0
NDUFS1	0.0870161	0	0
NDUFS2	0.3285225	0.3285225	0
NDUFS3	0.1261851	0.1261851	0
NDUFS4	0.0806851	0.0806851	0
NDUFS5	0.0232379	0.0232379	0
NDUFS6	0.1406625	0.1406625	0
NDUFS7	0.0317021	0.0317021	0
NDUFS8	0.0193772	0.0193772	0
NDUFV1	0.011325	0.011325	0
NDUFV2	0.3719227	0.3719227	0
NDUFV3	0.0146856	0.0146856	0
NEAT1	1	1	0
NEB	0.0090569	0	0.0090569
NEBL	0.029518	0.029518	0
NECAB1	0.0081623	0	0.0081623

NECAB2	0.0007492	0	0.0007492
NECAB3	0.012551	0.012551	0
NECAP1	0.1302892	0	0
NECAP2	0.0156112	0	0.0156112
NEDD1	0.0369962	0	0.0369962
NEDD4	0.6849988	0	0.6849988
NEDD4L	0.7669293	0.7669293	0
NEDD8	0.1932075	0.1932075	0
NEDD9	0.1446397	0	0.1446397
NEFH	0.0014907	0	0.0014907
NEFL	0.0070279	0	0.0070279
NEFM	0.0069105	0	0.0069105
NEGR1	0.0526304	0	0.0526304
NEIL1	0.0069503	0.0069503	0
NEIL2	0.0491291	0	0
NEIL3	0.1139582	0.1139582	0
NEK1	0.1219797	0	0.1219797
NEK10	0.0468092	0	0.0468092
NEK11	0.0641877	0	0
NEK2	0.1455611	0.1455611	0
NEK3	0.0158716	0.0158716	0
NEK4	0.036586	0	0
NEK5	0.0166228	0	0
NEK6	0.6655142	0	0.6655142
NEK7	0.3432594	0	0.3432594
NEK8	0.3912466	0.3912466	0
NEK9	0.1026358	0	0.1026358
NELF	0.0014931	0	0
NELL1	0.0050515	0.0050515	0
NELL2	0.0174301	0	0.0174301
NENF	0.0634145	0.0634145	0
NEO1	0.0235175	0	0
NES	0.1294665	0	0.1294665
NET1	0.0940946	0.0940946	0
NETO1	0.018138	0	0.018138
NETO2	0.0921108	0	0.0921108
NEU2	0.0088618	0	0
NEU3	0.0598718	0	0.0598718
NEU4	0.0002362	0	0
NEURL	0.0063349	0	0
NEURL1B	0.0132962	0	0.0132962
NEURL2	0.0335416	0.0335416	0
NEURL3	0.0109482	0	0.0109482
NEURL4	0.0163082	0	0
NEUROD1	0.0067059	0	0.0067059

NEUROD2	0.0017198	0.0017198	0
NEUROD4	0.0121233	0.0121233	0
NEUROD6	0.0041811	0.0041811	0
NEUROG1	0.0039201	0.0039201	0
NEUROG2	0.0176358	0	0
NEUROG3	0.0024498	0	0
NEXN	0.0073029	0	0.0073029
NF1	0.0483721	0	0.0483721
NF1P1	3.53E-05	0	0
NF2	0.402074	0	0.402074
NFAM1	0.0148169	0	0.0148169
NFASC	0.0161355	0	0.0161355
NFAT5	0.110569	0	0.110569
NFATC1	0.0032226	0	0.0032226
NFATC2	0.0317552	0	0.0317552
NFATC2IP	0.0378428	0.0378428	0
NFATC3	0.073303	0	0.073303
NFATC4	0.0183361	0	0.0183361
NFE2	0.6703504	0	0.6703504
NFE2L1	0.2320088	0	0.2320088
NFE2L2	0.4259241	0	0.4259241
NFE2L3	0.0318195	0	0
NFIA	0.136172	0	0.136172
NFIB	0.2597324	0	0.2597324
NFIC	0.0174715	0	0.0174715
NFIL3	0.824355	0	0.824355
NFIX	0.001475	0	0.001475
NFKB1	0.0895365	0	0.0895365
NFKB2	0.2282702	0	0
NFKBIA	0.2878078	0	0.2878078
NFKBIB	0.0193752	0.0193752	0
NFKBID	0.0191779	0	0
NFKBIE	0.0791823	0	0
NFKBIL2	0.002776	0.002776	0
NFKBIZ	0.7730901	0	0.7730901
NFRKB	0.0151117	0.0151117	0
NFS1	0.0735988	0.0735988	0
NFU1	0.0274966	0	0
NFX1	0.2130168	0	0
NFXL1	0.0428501	0.0428501	0
NFYA	0.2149387	0	0
NFYB	0.0330197	0	0.0330197
NFYC	0.0949619	0	0
NGB	0.0013498	0	0
NGDN	0.0463258	0.0463258	0

NGEF	0.0020723	0.0020723	0
NGF	0.1664578	0	0.1664578
NGFR	0.002535	0	0.002535
NGFRAP1	0.0038972	0	0
NGLY1	0.1463793	0	0
NGRN	0.1225762	0.1225762	0
NHEDC1	0.1187133	0	0.1187133
NHEDC2	0.4178492	0	0
NHEG1	0.0192939	0.0192939	0
NHEJ1	0.0080964	0.0080964	0
NHLH1	0.0091699	0	0
NHLH2	0.0136739	0	0
NHLRC1	0.0068831	0.0068831	0
NHLRC2	0.1603453	0	0
NHLRC3	0.1379955	0	0.1379955
NHLRC4	0.0009624	0	0.0009624
NHP2	0.0409454	0.0409454	0
NHP2L1	0.0198067	0.0198067	0
NHS	0.0135585	0	0
NHSL1	0.0307316	0	0.0307316
NHSL2	0.0025629	0	0.0025629
NICN1	0.029737	0	0
NID1	0.0495697	0	0.0495697
NID2	0.0472619	0	0.0472619
NIF3L1	0.0967037	0.0967037	0
NIN	0.0709686	0	0.0709686
NINJ1	0.0257353	0	0.0257353
NINJ2	0.0403603	0	0.0403603
NINL	0.002031	0.002031	0
NIP7	0.0411721	0	0
NIPA1	0.028489	0.028489	0
NIPA2	0.0383599	0	0
NIPAL1	0.0217196	0	0
NIPAL2	0.0273417	0	0
NIPAL3	0.091063	0	0
NIPAL4	0.4244716	0	0.4244716
NIPBL	0.310922	0	0.310922
NIPSNAP1	0.0064671	0.0064671	0
NIPSNAP3A	0.0692796	0	0
NIPSNAP3B	0.034623	0	0.034623
NISCH	0.008157	0	0.008157
NIT1	0.6587121	0.6587121	0
NIT2	0.0317768	0.0317768	0
NKAIN1	0.0028254	0	0.0028254
NKAIN2	0.0052021	0	0.0052021

NKAIN3	0.013363	0	0.013363
NKAIN4	0.0004856	0	0.0004856
NKAP	0.0140705	0.0140705	0
NKAPL	0.076041	0	0.076041
NKD1	0.0030517	0	0.0030517
NKD2	0.0096084	0	0.0096084
NKG7	0.0167478	0	0.0167478
NKIRAS1	0.0916299	0	0.0916299
NKIRAS2	0.0527417	0	0
NKPD1	0.0013748	0.0013748	0
NKRF	0.0188198	0	0
NKTR	0.1542112	0	0
NKX1-2	0.0029788	0.0029788	0
NKX2-1	0.0010379	0	0
NKX2-2	0.0003165	0	0
NKX2-3	0.0017047	0	0.0017047
NKX2-4	0.0058042	0.0058042	0
NKX2-5	0.0021712	0	0
NKX2-6	0.0030521	0	0
NKX2-8	0.004961	0.004961	0
NKX3-1	0.0104652	0	0
NKX3-2	0.0037279	0	0.0037279
NKX6-1	0.0032404	0	0
NKX6-2	0.0001953	0	0
NKX6-3	0.0015536	0	0
NLE1	0.1616974	0.1616974	0
NLGN1	0.0143997	0	0.0143997
NLGN2	0.0557057	0	0.0557057
NLGN3	0.0011483	0	0.0011483
NLGN4X	0.0030683	0	0.0030683
NLGN4Y	1.53E-05	0	0
NLK	0.124541	0.124541	0
NLN	0.0401117	0	0
NLRC3	0.0030925	0	0.0030925
NLRC4	0.0114274	0	0.0114274
NLRC5	0.1681746	0	0.1681746
NLRP1	0.0334597	0	0.0334597
NLRP10	0.0104313	0	0
NLRP11	0.0032288	0	0.0032288
NLRP12	0.0006804	0	0.0006804
NLRP13	0.0020375	0	0
NLRP14	0.0102395	0	0.0102395
NLRP2	0.0011821	0	0.0011821
NLRP3	0.0641999	0	0.0641999
NLRP4	0.0030046	0	0.0030046

NLRP5	0.0016674	0	0
NLRP6	0.0218829	0	0.0218829
NLRP7	0.0023834	0	0.0023834
NLRP8	0.0024389	0.0024389	0
NLRP9	0.0033653	0	0
NLRX1	0.0050686	0	0
NMB	0.0444971	0.0444971	0
NMBR	0.0032074	0	0.0032074
NMD3	0.0937576	0	0.0937576
NME1	0.174647	0.174647	0
NME1-NME2	0.174647	0.174647	0
NME2	0.0330374	0.0330374	0
NME2P1	0.048749	0.048749	0
NME3	0.0024058	0.0024058	0
NME4	0.0127456	0	0
NME5	0.0284191	0	0
NME6	0.0501905	0.0501905	0
NME7	0.2813806	0	0
NMI	0.203477	0	0
NMNAT1	0.0650581	0	0
NMNAT2	0.0421588	0	0
NMNAT3	0.0055599	0	0
NMRAL1	0.0198474	0.0198474	0
NMS	0.0041538	0	0
NMT1	0.0679035	0	0
NMT2	0.0098882	0	0.0098882
NMU	0.0056407	0.0056407	0
NMUR1	0.0010361	0	0.0010361
NMUR2	0.0120292	0	0
NNAT	0.1000517	0	0
NNMT	1	0	1
NNT	0.1134928	0	0
NOB1	0.045358	0.045358	0
NOBOX	0.0101567	0	0
NOC2L	0.0026262	0.0026262	0
NOC3L	0.0601458	0	0.0601458
NOC4L	0.0012959	0.0012959	0
NOD1	0.1235204	0	0.1235204
NOD2	0.216119	0	0.216119
NODAL	0.001606	0	0
NOG	0.0075604	0	0
NOL10	0.1009855	0.1009855	0
NOL11	0.0669219	0	0
NOL12	0.0201394	0.0201394	0
NOL3	0.0248286	0.0248286	0

NOL4	0.0106953	0	0.0106953
NOL6	0.0179384	0.0179384	0
NOL7	0.0579198	0.0579198	0
NOL8	0.1614912	0	0.1614912
NOL9	0.0064011	0	0.0064011
NOLC1	0.3814833	0	0
NOM1	0.0216878	0	0
NOMO1	2.68E-05	2.68E-05	0
NOMO2	4.03E-05	0	0
NOMO3	3.01E-05	0	0
NONO	0.0250044	0.0250044	0
NOP10	0.0995883	0.0995883	0
NOP14	0.0212142	0.0212142	0
NOP16	0.0685379	0.0685379	0
NOP2	0.0206022	0.0206022	0
NOP56	0.0212191	0	0
NOP58	0.1309973	0.1309973	0
NOS1	0.0049794	0	0.0049794
NOS1AP	0.0889399	0.0889399	0
NOS2	0.0168667	0	0.0168667
NOS3	0.0016174	0	0.0016174
NOSIP	0.0495413	0.0495413	0
NOSTRIN	0.0108029	0.0108029	0
NOTCH1	0.036313	0	0.036313
NOTCH2	0.0027441	0	0.0027441
NOTCH2NL	0.0328088	0	0
NOTCH3	0.0029425	0	0.0029425
NOTO	0.0025568	0	0
NOTUM	0.0006337	0	0.0006337
NOV	0.0547292	0	0.0547292
NOVA1	0.0037917	0	0.0037917
NOVA2	0.0020018	0	0.0020018
NOX1	0.0075918	0	0
NOX3	0.0119035	0	0
NOX4	0.0129036	0	0.0129036
NOX5	0.0122362	0	0
NOXA1	0.0145029	0.0145029	0
NOXO1	0.0052579	0.0052579	0
NPAS1	0.001269	0.001269	0
NPAS2	0.0315035	0.0315035	0
NPAS3	0.0038164	0	0.0038164
NPAS4	0.0131873	0	0.0131873
NPAT	0.1186244	0	0.1186244
NPBWR1	0.0055232	0	0
NPBWR2	0.0008287	0	0

NPC1	0.6382124	0	0
NPC1L1	0.0040499	0.0040499	0
NPC2	0.0606259	0	0.0606259
NPDC1	0.0055928	0.0055928	0
NPEPL1	0.0034358	0.0034358	0
NPEPPS	0.0038375	0	0
NPFF	0.0314103	0.0314103	0
NPFFR1	0.0090624	0	0
NPFFR2	0.0043686	0	0.0043686
NPHP1	0.0188823	0	0
NPHP3	0.0439265	0	0.0439265
NPHP4	0.0078959	0	0
NPHS1	0.0011939	0	0.0011939
NPHS2	0.0068231	0.0068231	0
NPIP	1.71E-05	0	0
NPIPL3	1.53E-05	1.53E-05	0
NPL	0.1109416	0	0.1109416
NPLOC4	0.0093454	0.0093454	0
NPM1	0.1277604	0	0
NPM2	0.0089135	0.0089135	0
NPM3	0.0174275	0.0174275	0
NPNT	0.008017	0	0
NPPA	0.003516	0	0.003516
NPPB	0.0029146	0	0
NPPC	0.0008821	0	0.0008821
NPR1	0.0823358	0	0.0823358
NPR2	0.0091759	0	0.0091759
NPR3	0.0137266	0	0.0137266
NPRL2	0.0276555	0.0276555	0
NPRL3	0.0084708	0.0084708	0
NPS	0.011573	0	0
NPSR1	0.0100669	0	0
NPTN	0.4587034	0	0
NPTX1	0.0009939	0	0.0009939
NPTX2	0.0044475	0	0.0044475
NPTXR	0.001572	0	0.001572
NPVF	0.0081246	0	0
NPW	0.0055213	0.0055213	0
NPY	0.0072029	0	0.0072029
NPY1R	0.007913	0	0.007913
NPY2R	0.0050046	0	0.0050046
NPY5R	0.0031816	0	0.0031816
NPY6R	0.0260647	0	0.0260647
NQO1	0.0394828	0.0394828	0
NQO2	0.0650024	0	0.0650024

NR0B1	0.0075266	0	0
NR0B2	0.0071795	0.0071795	0
NR1D1	0.5989676	0.5989676	0
NR1D2	0.2274345	0	0
NR1H2	0.0574753	0.0574753	0
NR1H3	0.0757973	0	0.0757973
NR1H4	0.0084767	0	0.0084767
NR1I2	0.0167794	0.0167794	0
NR1I3	0.0130556	0	0
NR2C1	0.0244361	0.0244361	0
NR2C2	0.0397527	0	0.0397527
NR2C2AP	0.0042947	0.0042947	0
NR2E1	0.0033081	0	0
NR2E3	0.0042216	0	0
NR2F1	0.0356247	0	0.0356247
NR2F2	0.119911	0	0.119911
NR2F6	0.0107097	0.0107097	0
NR3C1	0.6285754	0	0.6285754
NR3C2	0.0091092	0	0
NR4A1	0.0244726	0	0.0244726
NR4A2	0.1955649	0	0.1955649
NR4A3	0.1196008	0	0.1196008
NR5A1	0.0019039	0	0
NR5A2	0.0224183	0	0.0224183
NR6A1	0.1957483	0.1957483	0
NRADDP	0.0080215	0.0080215	0
NRAP	0.0107815	0.0107815	0
NRARP	0.0145359	0.0145359	0
NRAS	0.08602	0	0.08602
NRBF2	0.0461062	0	0.0461062
NRBP1	0.0201212	0	0.0201212
NRBP2	0.0131351	0.0131351	0
NRCAM	0.0338865	0	0.0338865
NRD1	0.6118007	0	0.6118007
NRF1	0.0610644	0	0
NRG1	0.0323125	0	0.0323125
NRG2	0.1077651	0	0.1077651
NRG3	0.0049806	0	0
NRG4	0.04859	0.04859	0
NRGN	0.0123587	0	0.0123587
NRIP1	0.120355	0	0.120355
NRIP2	0.0032605	0	0.0032605
NRIP3	0.0157948	0	0.0157948
NRK	0.0045912	0	0.0045912
NRL	0.0102636	0.0102636	0

NRN1	0.00611	0	0.00611
NRN1L	0.0048355	0.0048355	0
NRP1	0.1029124	0	0.1029124
NRP2	0.1605611	0	0.1605611
NRSN1	0.0095775	0	0.0095775
NRSN2	0.0132308	0	0.0132308
NRTN	0.0020364	0.0020364	0
NRXN1	0.0073664	0	0.0073664
NRXN2	0.0023783	0	0.0023783
NRXN3	0.0574283	0	0.0574283
NSA2	0.1240429	0	0
NSD1	0.0151806	0	0.0151806
NSDHL	0.0015357	0.0015357	0
NSF	1.53E-05	0	0
NSFL1C	0.116462	0.116462	0
NSL1	0.2659795	0	0.2659795
NSMAF	0.0476175	0	0.0476175
NSMCE1	0.0938991	0.0938991	0
NSMCE2	0.0705263	0	0
NSMCE4A	0.0093621	0.0093621	0
NSUN2	0.1335575	0.1335575	0
NSUN3	0.1525625	0	0.1525625
NSUN4	0.0780147	0	0.0780147
NSUN5	0.0028574	0.0028574	0
NSUN5P1	4.68E-05	4.68E-05	0
NSUN5P2	2.26E-05	2.26E-05	0
NSUN6	0.1686048	0.1686048	0
NSUN7	0.0071845	0.0071845	0
NT5C	0.0273345	0.0273345	0
NT5C1A	0.0116711	0	0.0116711
NT5C1B	0.0074685	0	0
NT5C2	0.0854125	0.0854125	0
NT5C3	0.0902345	0.0902345	0
NT5C3L	0.0066575	0.0066575	0
NT5DC1	0.0626427	0	0
NT5DC2	0.0128528	0	0.0128528
NT5DC3	0.02685	0	0
NT5E	0.0689339	0	0
NT5M	0.0114992	0	0
NTAN1	0.022706	0	0.022706
NTF3	0.0026858	0	0.0026858
NTF4	0.0017162	0.0017162	0
NTHL1	0.0020584	0.0020584	0
NTM	0.004487	0	0.004487
NTN1	0.0040714	0	0

NTN3	0.0028012	0	0.0028012
NTN4	0.023078	0	0
NTN5	0.0014816	0	0
NTNG1	0.02833	0	0.02833
NTNG2	0.0071229	0	0.0071229
NTRK1	0.0725972	0	0.0725972
NTRK2	0.0077411	0	0.0077411
NTRK3	0.0018467	0	0.0018467
NTS	0.0196244	0	0.0196244
NTSR1	0.0055538	0.0055538	0
NTSR2	0.036379	0	0.036379
NUAK1	0.0255693	0	0.0255693
NUAK2	0.6630258	0	0.6630258
NUB1	0.0639741	0	0
NUBP1	0.1257789	0	0.1257789
NUBP2	0.0172161	0.0172161	0
NUBPL	0.2066159	0.2066159	0
NUCB1	0.3194374	0	0
NUCB2	0.0616339	0	0
NUCKS1	0.4252369	0	0.4252369
NUDC	0.0125266	0.0125266	0
NUDCD1	0.2596004	0	0.2596004
NUDCD2	0.1867395	0	0
NUDCD3	0.1896911	0.1896911	0
NUDT1	0.0264819	0.0264819	0
NUDT10	0.0029173	0	0.0029173
NUDT11	0.0032832	0	0.0032832
NUDT12	0.0211049	0.0211049	0
NUDT13	0.1354503	0.1354503	0
NUDT14	0.0007852	0.0007852	0
NUDT15	0.0399981	0.0399981	0
NUDT16	0.0329002	0	0
NUDT16L1	0.00184	0.00184	0
NUDT16P1	0.0049254	0.0049254	0
NUDT17	0.0515712	0.0515712	0
NUDT18	0.0056953	0	0
NUDT19	0.0311835	0.0311835	0
NUDT2	0.118734	0.118734	0
NUDT21	0.0644995	0	0
NUDT22	0.0250182	0.0250182	0
NUDT3	0.0678994	0	0.0678994
NUDT4	0.0740195	0	0
NUDT5	0.052581	0.052581	0
NUDT6	0.0649392	0.0649392	0
NUDT7	0.0207197	0	0

NUDT8	0.0049152	0.0049152	0
NUDT9	0.0520031	0	0
NUDT9P1	0.0163912	0	0.0163912
NUF2	0.8159263	0.8159263	0
NUFIP1	0.0156644	0	0
NUFIP2	0.0703108	0	0
NUMA1	0.1528499	0.1528499	0
NUMB	0.2928714	0.2928714	0
NUMBL	0.0121854	0	0.0121854
NUP107	0.7145484	0	0
NUP133	0.083356	0	0
NUP153	0.1714698	0	0.1714698
NUP155	0.087432	0	0
NUP160	0.4153304	0	0.4153304
NUP188	0.0202678	0	0
NUP205	0.0770405	0	0
NUP210	0.0459143	0	0.0459143
NUP210L	0.0653318	0	0
NUP214	0.0531592	0	0
NUP35	0.054617	0.054617	0
NUP37	0.078888	0	0
NUP43	0.0409952	0	0.0409952
NUP50	0.0882299	0	0.0882299
NUP54	0.0345677	0	0
NUP62	0.0346585	0	0
NUP62CL	0.0120608	0.0120608	0
NUP85	0.0343963	0	0
NUP88	0.1684133	0	0
NUP93	0.0238593	0	0
NUP98	0.3391435	0	0.3391435
NUPL1	0.0377864	0	0
NUPL2	0.0630112	0.0630112	0
NUPR1	0.0487772	0	0.0487772
NUS1	0.0408478	0	0.0408478
NUSAP1	0.1038764	0.1038764	0
NUTF2	0.0190921	0.0190921	0
NVL	0.1266135	0.1266135	0
NWD1	0.0199505	0	0
NXF1	0.0514955	0	0.0514955
NXF3	0.0037951	0	0
NXF4	0.0021767	0	0
NXF5	0.001339	0	0
NXN	0.004135	0	0.004135
NXNL1	0.0118899	0	0.0118899
NXNL2	0.0203313	0.0203313	0

NXPH1	0.00347	0.00347	0
NXPH2	0.0105203	0	0.0105203
NXPH3	0.0070313	0	0.0070313
NXPH4	0.0027257	0	0
NXT1	0.1620721	0	0
NXT2	0.060572	0	0.060572
NYNRIN	0.0062122	0.0062122	0
NYX	0.0723303	0	0
OAF	0.0062538	0	0.0062538
OAS1	0.2250785	0	0
OAS2	0.0444886	0	0.0444886
OAS3	0.0635598	0	0.0635598
OASL	0.0315681	0	0
OAT	0.008818	0	0
OAZ1	0.0374242	0.0374242	0
OAZ2	0.0388865	0	0.0388865
OAZ3	0.0978622	0.0978622	0
OBFC1	0.2727742	0.2727742	0
OBFC2A	0.2017176	0	0.2017176
OBFC2B	0.0434955	0.0434955	0
OBP2A	0.0030275	0	0
OBP2B	0.0017636	0	0
OBSCN	0.0049782	0	0
OBSL1	0.0028518	0.0028518	0
OC90	0.0142165	0.0142165	0
OCA2	0.001459	0	0.001459
OCEL1	0.0145841	0.0145841	0
OCIAD1	0.0304903	0.0304903	0
OCIAD2	0.0407753	0.0407753	0
OCLM	0.053174	0.053174	0
OCM	0.0014309	0	0
OCM2	0.0010585	0	0.0010585
OCRL	0.0079588	0.0079588	0
ODAM	0.0160012	0	0.0160012
ODC1	0.5704894	0	0.5704894
ODF1	0.0420877	0	0.0420877
ODF2	0.0105127	0	0
ODF2L	0.0408123	0	0.0408123
ODF3	0.0172951	0	0
ODF3B	1	1	0
ODF3L1	0.0183685	0	0.0183685
ODF3L2	0.00056	0	0
ODF4	0.0039084	0	0
ODZ1	0.002529	0	0.002529
ODZ2	0.1005623	0	0.1005623

ODZ3	0.0040537	0	0.0040537
ODZ4	0.0190303	0	0.0190303
OFD1	0.056231	0.056231	0
OGDH	0.0370326	0	0
OGDHL	0.0022376	0	0
OGFOD1	0.0641535	0	0.0641535
OGFOD2	0.0303901	0.0303901	0
OGFR	0.0050845	0.0050845	0
OGFRL1	0.0325795	0	0.0325795
OGG1	0.0257986	0	0
OGN	0.016084	0	0.016084
OGT	0.1795764	0	0
OIP5	0.1056733	0.1056733	0
OIT3	0.0180438	0	0.0180438
OLA1	0.0201129	0.0201129	0
OLAH	0.0128342	0	0.0128342
OLFM1	0.002061	0	0.002061
OLFM2	0.004994	0	0.004994
OLFM3	0.0086219	0	0.0086219
OLFM4	0.0440776	0	0
OLFML1	0.0137819	0	0.0137819
OLFML2A	0.0667732	0	0.0667732
OLFML2B	0.0563697	0	0.0563697
OLFML3	0.0197092	0	0.0197092
OLIG1	0.0038495	0	0.0038495
OLIG2	0.0011775	0	0.0011775
OLIG3	0.0023829	0	0.0023829
OLR1	0.8239325	0	0.8239325
OMA1	0.069615	0	0
OMD	0.0116979	0	0.0116979
OMG	0.0336871	0	0.0336871
OMP	0.0015693	0.0015693	0
ONECUT1	0.0043031	0	0
ONECUT2	0.0163344	0.0163344	0
ONECUT3	0.0011522	0.0011522	0
OOEP	0.0020712	0	0
OPA1	0.0946157	0	0
OPA3	0.0386211	0	0
OPALIN	0.0044429	0	0
OPCML	0.0036162	0	0.0036162
OPHN1	0.0249124	0	0.0249124
OPLAH	0.0089978	0.0089978	0
OPN1LW	0.0002186	0	0.0002186
OPN1SW	0.0079442	0	0.0079442
OPN3	0.5729986	0	0.5729986

OPN4	0.0056913	0	0
OPN5	0.0384558	0	0.0384558
OPRD1	0.0017521	0	0.0017521
OPRK1	0.0056372	0	0
OPRL1	0.0022155	0	0.0022155
OPRM1	0.0066491	0	0.0066491
OPTC	0.0131334	0	0
OPTN	0.0165214	0	0
OR10A2	0.0057501	0	0
OR10A3	0.0121564	0	0
OR10A4	0.0079988	0.0079988	0
OR10A5	0.0089189	0	0
OR10A6	0.00692	0	0
OR10A7	0.004454	0.004454	0
OR10AD1	0.0242118	0	0
OR10AG1	0.0044035	0	0
OR10G2	0.0048641	0	0.0048641
OR10G3	0.0032381	0	0
OR10G4	0.0045923	0	0.0045923
OR10G7	0.0053636	0	0
OR10G8	0.0040443	0	0.0040443
OR10G9	0.0033052	0	0
OR10H1	0.0052798	0	0
OR10H2	0.0056379	0.0056379	0
OR10H3	0.007186	0.007186	0
OR10H4	0.0093009	0	0
OR10H5	0.0018259	0	0
OR10J1	0.0185487	0	0
OR10J3	0.0223598	0.0223598	0
OR10J5	0.0044163	0	0.0044163
OR10K1	0.0163186	0	0
OR10K2	0.0165094	0	0
OR10P1	0.0142556	0	0
OR10Q1	0.0098147	0	0
OR10R2	0.0228593	0	0
OR10S1	0.0386656	0	0
OR10T2	0.0170918	0	0
OR10V1	0.0138518	0	0.0138518
OR10W1	0.007622	0	0
OR10X1	0.016156	0	0
OR10Z1	0.0187813	0	0
OR11G2	0.0081756	0	0
OR11H1	1.56E-05	0	0
OR11H12	1.61E-05	0	0
OR11H4	0.0022219	0	0

OR11H6	0.0034207	0.0034207	0
OR11L1	0.0104908	0	0
OR13A1	0.0079865	0	0
OR13C2	0.0168934	0.0168934	0
OR13C3	0.011196	0	0
OR13C4	0.0098877	0.0098877	0
OR13C5	0.0124837	0	0
OR13C8	0.0063817	0	0
OR13C9	0.0078313	0	0
OR13D1	0.0055658	0	0
OR13F1	0.0073387	0	0
OR13G1	0.0244946	0	0
OR13H1	0.008086	0	0
OR13J1	0.0071748	0.0071748	0
OR14A16	0.0144891	0	0
OR14C36	0.0200223	0	0
OR14I1	0.0408189	0	0
OR1A1	0.0049873	0	0
OR1A2	0.0078284	0	0
OR1B1	0.0107811	0	0
OR1C1	0.0173073	0	0
OR1D2	0.0057905	0	0
OR1D4	2.77E-05	0	0
OR1E1	0.0038588	0	0.0038588
OR1E2	0.0082358	0	0
OR1F1	0.002687	0	0.002687
OR1F2P	0.0068633	0	0.0068633
OR1G1	0.0054281	0	0
OR1I1	0.0065835	0.0065835	0
OR1J1	0.0172097	0.0172097	0
OR1J2	0.0164144	0	0
OR1J4	0.0160027	0.0160027	0
OR1K1	0.0080036	0	0
OR1L1	0.0213134	0.0213134	0
OR1L3	0.0144104	0.0144104	0
OR1L4	0.0090059	0	0.0090059
OR1L6	0.016407	0	0
OR1L8	0.012285	0	0.012285
OR1M1	0.0009569	0	0
OR1N1	0.0092799	0	0
OR1N2	0.0152791	0	0
OR1Q1	0.0135782	0	0
OR1S1	0.00965	0	0
OR1S2	0.0086786	0	0
OR2A12	0.0080643	0	0

OR2A14	0.0086845	0.0086845	0
OR2A2	0.0124921	0	0
OR2A25	0.0081343	0	0.0081343
OR2A4	0.0003168	0.0003168	0
OR2A5	0.0055288	0.0055288	0
OR2A7	0.0001243	0.0001243	0
OR2AE1	0.0067954	0	0
OR2AG1	0.0112165	0	0
OR2AG2	0.0083017	0	0
OR2AK2	0.0059073	0	0
OR2AT4	0.009442	0	0
OR2B11	0.0228784	0	0.0228784
OR2B2	0.0561412	0.0561412	0
OR2B6	0.0160989	0.0160989	0
OR2C1	0.0047262	0	0.0047262
OR2C3	0.019182	0	0
OR2D2	0.0163255	0.0163255	0
OR2D3	0.0137065	0	0
OR2F1	0.0158486	0	0.0158486
OR2F2	0.0109096	0	0
OR2G2	0.0135773	0	0
OR2G3	0.0195616	0	0
OR2G6	0.0013074	0	0
OR2K2	0.0058626	0.0058626	0
OR2L13	0.0259905	0	0.0259905
OR2L1P	0.0140481	0	0.0140481
OR2L2	0.0213514	0	0
OR2L3	0.0137098	0	0
OR2L8	0.0152468	0	0
OR2M1P	0.0055425	0	0
OR2M2	0.0114324	0.0114324	0
OR2M3	0.0163713	0.0163713	0
OR2M4	0.0307953	0	0
OR2M5	0.0193089	0.0193089	0
OR2M7	0.0138574	0.0138574	0
OR2S2	0.0076678	0	0
OR2T1	0.0571131	0	0
OR2T10	0.0054522	0	0.0054522
OR2T11	0.0033016	0	0.0033016
OR2T12	0.0071209	0	0
OR2T2	0.0050129	0	0.0050129
OR2T27	0.0007655	0	0
OR2T29	5.66E-05	0	0
OR2T3	0.0253601	0	0
OR2T33	0.015369	0	0

OR2T34	0.0004085	0	0
OR2T35	0.0002306	0	0
OR2T4	0.0223205	0.0223205	0
OR2T5	0.0002077	0	0.0002077
OR2T6	0.0129141	0	0
OR2T8	0.0181314	0	0.0181314
OR2V2	0.0196054	0	0
OR2W3	0.0015716	0	0.0015716
OR2W5	0.0846778	0	0
OR2Y1	0.0089132	0	0
OR2Z1	0.0012993	0	0
OR3A1	0.0028454	0	0.0028454
OR3A2	0.0016241	0	0.0016241
OR3A3	0.0057949	0	0.0057949
OR3A4	0.0029395	0	0
OR4A15	0.0029348	0	0
OR4A16	0.0031386	0	0
OR4A47	0.0094837	0	0
OR4A5	0.0016025	0	0
OR4B1	0.0158689	0.0158689	0
OR4C11	0.001014	0	0
OR4C12	0.0045896	0	0
OR4C13	0.0014221	0	0
OR4C15	0.0040025	0	0
OR4C16	0.0021118	0	0
OR4C3	0.0606722	0	0
OR4C45	0.0293375	0	0
OR4C46	0.0020954	0	0
OR4C6	0.0018928	0	0
OR4D1	0.0071471	0	0
OR4D10	0.0092844	0.0092844	0
OR4D11	0.0077656	0	0
OR4D2	0.0076799	0	0
OR4D5	0.0116423	0	0
OR4D6	0.0081417	0	0
OR4D9	0.0065834	0	0
OR4E2	0.0069146	0	0.0069146
OR4F15	0.0187521	0	0
OR4F17	1.64E-05	0	0
OR4F21	1.73E-05	0	1.73E-05
OR4F4	2.63E-05	0	0
OR4F5	1.84E-05	0	1.84E-05
OR4F6	0.024582	0	0
OR4K1	0.0071533	0	0
OR4K13	0.0022041	0	0

OR4K14	0.0069803	0	0
OR4K15	0.0042351	0	0
OR4K17	0.0068397	0	0
OR4K2	0.0064257	0	0
OR4K5	0.0050765	0	0
OR4L1	0.0094417	0	0
OR4M1	0.0067856	0.0067856	0
OR4M2	0.0150648	0	0
OR4N2	0.0018499	0	0.0018499
OR4N3P	0.0258318	0	0
OR4N4	0.0138046	0.0138046	0
OR4N5	0.0077041	0	0
OR4P4	0.00067	0	0
OR4Q3	0.0044642	0	0
OR4S1	0.0182676	0	0
OR4S2	0.0011546	0	0
OR4X1	0.0278667	0	0
OR4X2	0.0251743	0	0
OR51A2	0.0031588	0	0
OR51A4	0.0043081	0	0
OR51A7	0.0113841	0.0113841	0
OR51B2	0.0094025	0.0094025	0
OR51B4	0.0102618	0	0
OR51B5	0.0344538	0	0
OR51B6	0.0092014	0	0
OR51D1	0.0062963	0	0
OR51E1	0.0091672	0	0.0091672
OR51E2	0.0237656	0	0.0237656
OR51F1	0.0078394	0.0078394	0
OR51F2	0.0132901	0.0132901	0
OR51G1	0.0255317	0	0
OR51G2	0.0270221	0	0
OR51I1	0.0133344	0	0
OR51I2	0.0104612	0.0104612	0
OR51L1	0.0139857	0	0
OR51M1	0.0100288	0	0
OR51Q1	0.0035094	0	0
OR51S1	0.0033209	0	0
OR51T1	0.0097486	0	0.0097486
OR51V1	0.0089781	0	0
OR52A1	0.0050867	0	0
OR52A4	0.0164975	0	0
OR52A5	0.0167818	0	0
OR52B2	0.0072872	0	0
OR52B4	0.0050627	0	0

OR52B6	0.0044134	0	0
OR52D1	0.0063823	0	0
OR52E2	0.0119105	0	0
OR52E4	0.0159501	0	0
OR52E6	0.0090472	0	0
OR52E8	0.006454	0	0
OR52H1	0.0295436	0	0
OR52I1	0.0098623	0.0098623	0
OR52I2	0.0130979	0	0
OR52J3	0.0160471	0	0
OR52K1	0.0257872	0	0
OR52K2	0.0107596	0	0.0107596
OR52L1	0.0101361	0	0
OR52M1	0.0141486	0	0
OR52N1	0.012107	0	0.012107
OR52N2	0.0127004	0	0.0127004
OR52N4	0.0127628	0	0.0127628
OR52N5	0.0101599	0	0
OR52R1	0.0051299	0	0
OR52W1	0.0203355	0	0
OR56A1	0.0105123	0	0
OR56A3	0.0086635	0	0
OR56A4	0.0055393	0.0055393	0
OR56A5	0.0080834	0	0
OR56B1	0.0109668	0	0.0109668
OR56B4	0.0081062	0.0081062	0
OR5A1	0.0102748	0	0
OR5A2	0.0180054	0	0
OR5AC2	0.0027103	0	0
OR5AK2	0.0058213	0	0
OR5AN1	0.0080147	0	0
OR5AP2	0.0037238	0.0037238	0
OR5AR1	0.0045031	0	0
OR5AS1	0.0036782	0	0
OR5AU1	0.0062028	0	0.0062028
OR5B12	0.0096845	0	0
OR5B17	0.0096889	0	0
OR5B2	0.0054663	0	0
OR5B21	0.0258309	0	0
OR5B3	0.0140271	0.0140271	0
OR5C1	0.0051783	0.0051783	0
OR5D13	0.0047432	0	0
OR5D14	0.0050019	0	0
OR5D16	0.0047527	0	0.0047527
OR5D18	0.0047091	0	0

OR5E1P	0.0182319	0.0182319	0
OR5F1	0.0016246	0	0
OR5H1	0.0096636	0	0
OR5H14	0.0081812	0	0
OR5H15	0.0040447	0	0
OR5H2	0.0085885	0	0.0085885
OR5H6	0.007194	0	0
OR5I1	0.0061412	0	0
OR5J2	0.0047631	0	0
OR5K1	0.0056981	0	0
OR5K2	0.0087138	0	0.0087138
OR5K3	0.0065457	0	0
OR5K4	0.0127268	0	0
OR5L1	0.0050608	0	0
OR5L2	0.0048424	0	0
OR5M1	0.0072389	0	0
OR5M10	0.0041084	0	0
OR5M11	0.0026398	0	0
OR5M3	0.0051629	0	0
OR5M8	0.0039065	0	0
OR5M9	0.0065109	0	0
OR5P2	0.0024187	0	0
OR5P3	0.0183505	0	0
OR5R1	0.0047983	0	0
OR5T1	0.0045122	0	0
OR5T2	0.003273	0.003273	0
OR5T3	0.0044453	0	0
OR5W2	0.0054336	0	0
OR6A2	0.0116645	0.0116645	0
OR6B1	0.0068292	0	0
OR6B2	0.0077936	0	0
OR6B3	0.0026923	0	0
OR6C1	0.0157673	0	0
OR6C2	0.0081998	0.0081998	0
OR6C3	0.0120223	0	0
OR6C4	0.0060786	0	0
OR6C6	0.0065751	0	0
OR6C65	0.0118719	0	0
OR6C68	0.0052332	0	0
OR6C70	0.0094172	0	0
OR6C74	0.0136398	0	0
OR6C75	0.0056973	0	0
OR6C76	0.0110335	0	0
OR6F1	0.0128796	0	0.0128796
OR6K2	0.0146288	0.0146288	0

OR6K3	0.0315607	0	0.0315607
OR6K6	0.0100716	0	0
OR6M1	0.00911	0	0
OR6N1	0.0083605	0	0
OR6N2	0.0230851	0	0
OR6P1	0.0180158	0	0
OR6Q1	0.018913	0	0.018913
OR6S1	0.013522	0	0.013522
OR6T1	0.0118574	0	0
OR6V1	0.0124698	0	0
OR6W1P	0.0053131	0	0
OR6X1	0.0118979	0	0
OR6Y1	0.0166017	0	0
OR7A10	0.0167227	0	0
OR7A17	0.0174559	0	0
OR7A5	0.0248946	0	0
OR7C1	0.01555	0	0
OR7C2	0.004257	0	0
OR7D2	0.0019629	0	0
OR7D4	0.0041829	0	0
OR7E156P	0.0018562	0	0
OR7E24	0.0028123	0	0
OR7E5P	0.0011796	0	0
OR7E91P	0.0110082	0.0110082	0
OR7G1	0.0021315	0	0
OR7G2	0.001509	0	0
OR7G3	0.0036948	0	0
OR8A1	0.0046665	0	0
OR8B12	0.0057087	0	0
OR8B2	0.0039148	0	0
OR8B3	0.0025341	0.0025341	0
OR8B4	0.0052451	0	0
OR8B8	0.0064286	0	0
OR8D1	0.0242045	0	0
OR8D2	0.0151523	0	0
OR8D4	0.0098213	0	0.0098213
OR8G2	0.0106544	0	0
OR8G5	0.0102867	0	0
OR8H1	0.006093	0	0
OR8H2	0.0042829	0	0
OR8H3	0.0044339	0	0
OR8I2	0.0030269	0	0
OR8J1	0.0035421	0	0
OR8J3	0.0039407	0	0
OR8K1	0.0044753	0	0

OR8K3	0.0038375	0	0
OR8K5	0.005181	0	0
OR8S1	0.010327	0	0
OR8U1	0.0059491	0	0
OR9A2	0.0045726	0	0
OR9A4	0.0022484	0	0
OR9G4	0.0058941	0	0
OR9G9	0.0129919	0	0
OR9I1	0.0162813	0	0
OR9K2	0.0138714	0	0
OR9Q1	0.0210088	0	0
OR9Q2	0.0086529	0	0
ORAI1	0.0419866	0	0
ORAI2	0.0383943	0	0.0383943
ORAI3	0.026074	0.026074	0
ORAOV1	0.0161565	0.0161565	0
ORC1L	0.0779034	0	0
ORC2L	0.069954	0.069954	0
ORC3L	0.0605977	0	0.0605977
ORC4L	0.1690496	0.1690496	0
ORC5L	0.0209036	0.0209036	0
ORC6L	0.0691113	0.0691113	0
ORM1	0.0092271	0	0
ORM2	0.001066	0.001066	0
ORMDL1	0.0545835	0	0
ORMDL2	0.1377004	0.1377004	0
ORMDL3	0.0678142	0.0678142	0
OS9	0.0485941	0	0
OSBP	0.0673535	0.0673535	0
OSBP2	0.003329	0.003329	0
OSBPL10	0.0532282	0	0
OSBPL11	0.0556766	0	0.0556766
OSBPL1A	0.0320422	0	0.0320422
OSBPL2	0.00607	0.00607	0
OSBPL3	0.0821859	0.0821859	0
OSBPL5	0.0018476	0	0.0018476
OSBPL6	0.0171925	0	0.0171925
OSBPL7	0.0076573	0.0076573	0
OSBPL8	0.0682265	0	0.0682265
OSBPL9	0.122325	0	0.122325
OSCAR	0.0028143	0	0.0028143
OSCP1	0.0176831	0	0
OSGEP	0.1615768	0.1615768	0
OSGEPL1	0.0549573	0	0
OSGIN1	0.0192178	0.0192178	0

OSGIN2	0.1753541	0	0.1753541
OSM	0.0031238	0	0.0031238
OSMR	0.8482848	0	0.8482848
OSR1	0.0052594	0	0.0052594
OSR2	0.0391552	0	0.0391552
OST4	0.025468	0.025468	0
OSTalpha	0.0039524	0	0.0039524
OSTBETA	0.0150723	0	0.0150723
OSTC	0.0613976	0	0.0613976
OSTCL	0.0951119	0	0.0951119
OSTF1	0.0650628	0	0.0650628
OSTM1	0.0153781	0	0.0153781
OSTN	0.0128971	0	0.0128971
OTC	0.0082749	0	0
OTOA	0.0067188	0	0.0067188
OTOF	0.001996	0	0.001996
OTOL1	0.0601364	0	0
OTOP1	0.0029095	0	0
OTOP2	0.0009387	0.0009387	0
OTOP3	0.0066529	0	0
OTOR	0.0070573	0	0.0070573
OTOS	0.0014246	0	0
OTP	0.0110192	0.0110192	0
OTUB1	0.008286	0.008286	0
OTUB2	0.0080871	0.0080871	0
OTUD1	0.1948281	0	0.1948281
OTUD3	0.0330207	0	0
OTUD4	0.0685953	0	0.0685953
OTUD5	0.0173033	0	0
OTUD6A	0.0024548	0.0024548	0
OTUD6B	0.0972516	0	0
OTUD7A	0.000784	0	0.000784
OTUD7B	0.1369107	0	0
OTX1	0.0087897	0.0087897	0
OTX2	0.0028096	0.0028096	0
OVCA2	0.0093321	0.0093321	0
OVCH1	0.0096641	0	0.0096641
OVCH2	0.012963	0	0
OVGP1	0.0214291	0.0214291	0
OVOL1	0.0029456	0.0029456	0
OVOL2	0.0033674	0.0033674	0
OXA1L	0.0461232	0	0
OXCT1	0.0779934	0	0.0779934
OXCT2	0.0002008	0	0.0002008
OXER1	0.0032488	0	0.0032488

OXGR1	0.0211238	0	0.0211238
OXNAD1	0.0775003	0	0.0775003
OXR1	0.0837074	0	0
OXSM	0.1438212	0.1438212	0
OXSR1	0.128727	0	0
OXT	0.0095621	0	0
OXTR	0.0114126	0	0.0114126
P2RX1	0.007533	0	0.007533
P2RX2	0.0022344	0	0.0022344
P2RX3	0.0115023	0	0
P2RX4	0.0321045	0.0321045	0
P2RX5	0.0068463	0	0.0068463
P2RX6	0.0020867	0.0020867	0
P2RX6P	0.007446	0	0
P2RX7	0.0375479	0	0.0375479
P2RY1	0.0245974	0	0.0245974
P2RY10	0.0420949	0	0.0420949
P2RY11	0.0491774	0.0491774	0
P2RY12	0.06461	0	0.06461
P2RY13	0.1094416	0	0.1094416
P2RY14	0.0346525	0	0.0346525
P2RY2	0.0091039	0.0091039	0
P2RY4	0.0059933	0	0
P2RY6	0.0352294	0	0.0352294
P4HA1	0.0855095	0	0
P4HA2	0.0315453	0.0315453	0
P4HA3	0.0314827	0	0.0314827
P4HTM	0.0172519	0.0172519	0
P704P	1.53E-05	0	1.53E-05
PA2G4	0.0742983	0.0742983	0
PA2G4P4	0.3914836	0.3914836	0
PAAF1	0.1018891	0	0
PABPC1	0.3405461	0.3405461	0
PABPC1L	0.0516156	0.0516156	0
PABPC1L2A	0.0007248	0	0
PABPC1L2B	0.0002652	0	0
PABPC1P2	0.0203557	0	0
PABPC3	0.0028819	0	0
PABPC4	0.0186796	0	0.0186796
PABPC4L	0.0134991	0	0.0134991
PABPC5	0.0052539	0	0.0052539
PABPN1	0.049269	0.049269	0
PABPN1L	0.0010745	0	0.0010745
PACRG	0.0128441	0	0
PACRGL	0.0253679	0.0253679	0

PACS1	0.1081191	0	0.1081191
PACS2	0.0016061	0.0016061	0
PACSIN1	0.0032877	0	0.0032877
PACSIN2	0.0654627	0	0
PACSIN3	0.2634875	0	0
PADI1	0.025082	0	0
PADI2	0.0191757	0	0.0191757
PADI3	0.006165	0	0
PADI4	0.0068409	0	0.0068409
PADI6	0.0032423	0	0.0032423
PAEP	0.0279391	0	0
PAF1	0.1043139	0.1043139	0
PAFAH1B1	0.0200785	0	0.0200785
PAFAH1B2	0.008371	0	0.008371
PAFAH1B3	0.0170395	0.0170395	0
PAFAH2	0.0688891	0.0688891	0
PAG1	0.0491737	0	0.0491737
PAGE1	0.0059743	0	0.0059743
PAGE2	0.0011389	0	0
PAGE2B	0.0007038	0.0007038	0
PAGE3	0.0040149	0	0
PAGE4	0.006508	0	0
PAGE5	0.0045307	0	0
PAH	0.0111555	0	0
PAICS	0.0964211	0.0964211	0
PAIP1	0.1363777	0.1363777	0
PAIP2	0.0815852	0	0.0815852
PAIP2B	0.0191769	0	0
PAK1	0.0685045	0.0685045	0
PAK1IP1	0.0610962	0	0.0610962
PAK2	0.0688096	0	0.0688096
PAK3	0.0179204	0	0.0179204
PAK4	0.0137761	0.0137761	0
PAK6	0.0411258	0.0411258	0
PAK7	0.002375	0	0
PALB2	0.0316894	0.0316894	0
PALLD	0.1313528	0	0.1313528
PALM	0.0009895	0	0.0009895
PALM2	0.0382156	0	0.0382156
PALM2-AKAP2	0.0371998	0	0.0371998
PALM3	0.0017796	0.0017796	0
PALMD	0.1483213	0	0.1483213
PAM	0.0537483	0	0.0537483
PAMR1	0.0129173	0	0.0129173
PAN2	0.0587606	0.0587606	0

PAN3	0.0465267	0	0
PANK1	0.1084712	0.1084712	0
PANK2	0.0520469	0	0.0520469
PANK3	0.2051888	0	0
PANK4	0.0032727	0	0
PANX1	0.5175381	0	0.5175381
PANX2	0.0004296	0	0
PANX3	0.0040923	0	0
PAOX	0.003861	0	0
PAPD4	0.0843454	0	0.0843454
PAPD5	0.0253471	0	0.0253471
PAPD7	0.0879375	0	0
PAPL	0.0741028	0	0.0741028
PAPLN	0.0114084	0	0.0114084
PAPOLA	0.0866469	0	0
PAPOLB	0.0050241	0	0.0050241
PAPOLG	0.0233957	0	0.0233957
PAPPA	0.0677151	0	0.0677151
PAPPA2	0.0126906	0	0.0126906
PAPSS1	0.0183075	0	0.0183075
PAPSS2	0.0384936	0	0.0384936
PAQR3	0.0357264	0	0.0357264
PAQR4	0.0034889	0.0034889	0
PAQR5	0.0192539	0	0
PAQR6	0.0057213	0.0057213	0
PAQR7	0.0073093	0	0.0073093
PAQR8	0.0688111	0	0
PAQR9	0.0177766	0	0
PAR1	0.0062223	0	0.0062223
PAR4	0.0003175	0	0
PAR5	0.0057434	0	0.0057434
PARD3	0.0089955	0.0089955	0
PARD3B	0.0092179	0	0.0092179
PARD6A	0.0069799	0.0069799	0
PARD6B	0.1803124	0.1803124	0
PARD6G	0.0030397	0	0.0030397
PARG	1.57E-05	0	0
PARK2	0.0130326	0	0.0130326
PARK7	0.0426809	0	0
PARL	0.0273763	0.0273763	0
PARM1	0.0261489	0	0
PARN	0.0563844	0	0
PARP1	0.204122	0	0
PARP10	0.0697988	0.0697988	0
PARP11	0.0334491	0	0.0334491

PARP12	0.0210917	0	0
PARP14	0.2425357	0	0.2425357
PARP15	0.1710495	0	0.1710495
PARP16	0.1640213	0	0
PARP2	0.1037835	0.1037835	0
PARP3	0.0096561	0.0096561	0
PARP4	0.1201119	0	0
PARP6	0.0636851	0.0636851	0
PARP8	0.0519279	0	0.0519279
PARP9	0.725062	0	0.725062
PARS2	0.025362	0	0
PAR-SN	0.0047161	0	0.0047161
PART1	0.0301429	0	0
PARVA	0.0157304	0	0.0157304
PARVB	0.0283059	0	0.0283059
PARVG	0.0094811	0	0.0094811
PASD1	0.0005204	0	0.0005204
PASK	0.0189452	0	0.0189452
PATE1	0.0049962	0.0049962	0
PATE2	0.0034068	0	0
PATE3	0.0022824	0	0
PATE4	0.0033611	0	0
PATL1	0.0769711	0	0
PATL2	0.0133793	0	0.0133793
PATZ1	0.0084898	0.0084898	0
PAWR	0.2531721	0.2531721	0
PAX1	0.0007687	0	0.0007687
PAX2	0.0008796	0.0008796	0
PAX3	0.0009981	0	0
PAX4	0.0030706	0	0
PAX5	0.0274368	0	0.0274368
PAX6	0.0046097	0	0.0046097
PAX7	0.001253	0	0
PAX8	0.0095124	0	0
PAX9	0.0086386	0.0086386	0
PAXIP1	0.0272729	0	0
PBK	0.063611	0.063611	0
PBLD	0.156049	0.156049	0
PBOV1	0.0066738	0.0066738	0
PBRM1	0.1889062	0	0.1889062
PBX1	0.1238302	0	0.1238302
PBX3	0.0863043	0	0.0863043
PBX4	0.007935	0	0
PBXIP1	0.0402007	0	0.0402007
PC	0.0445653	0	0.0445653

PCA3	0.0081478	0	0.0081478
PCBD1	0.0225688	0.0225688	0
PCBD2	0.0711982	0.0711982	0
PCBP1	0.2045325	0.2045325	0
PCBP2	0.1031101	0	0
PCBP3	0.006377	0	0.006377
PCBP4	0.0068286	0.0068286	0
PCCA	0.0332552	0.0332552	0
PCCB	0.0243062	0.0243062	0
PCDH1	0.0184584	0.0184584	0
PCDH10	0.0078669	0	0.0078669
PCDH11X	0.0066109	0	0.0066109
PCDH11Y	1.53E-05	0	1.53E-05
PCDH12	0.0303811	0	0.0303811
PCDH15	0.0128405	0	0.0128405
PCDH17	0.0080631	0	0.0080631
PCDH18	0.0143587	0	0.0143587
PCDH19	0.003194	0	0.003194
PCDH20	0.0063	0	0
PCDH7	0.0408077	0	0.0408077
PCDH8	0.002705	0	0.002705
PCDH9	0.052398	0	0.052398
PCDHA1	0.0238145	0	0
PCDHA10	0.0074052	0	0.0074052
PCDHA11	0.0116057	0	0
PCDHA12	0.0157032	0	0.0157032
PCDHA13	0.0119746	0	0.0119746
PCDHA2	0.0230745	0	0
PCDHA3	0.0220965	0	0.0220965
PCDHA4	0.0125824	0	0.0125824
PCDHA5	0.0129522	0	0
PCDHA6	0.0088283	0	0.0088283
PCDHA7	0.0089239	0	0.0089239
PCDHA8	0.0083035	0	0
PCDHA9	0.0099161	0	0
PCDHAC1	0.0267947	0	0.0267947
PCDHAC2	0.0155694	0	0.0155694
PCDHB1	0.009905	0	0
PCDHB10	0.0047472	0	0
PCDHB11	0.0057787	0	0
PCDHB12	0.0057453	0	0.0057453
PCDHB13	0.0104662	0	0
PCDHB14	0.0126678	0.0126678	0
PCDHB15	0.0081345	0	0.0081345
PCDHB16	0.0056818	0	0.0056818

PCDHB17	0.0079454	0.0079454	0
PCDHB18	0.0067752	0	0.0067752
PCDHB19P	0.0071335	0	0
PCDHB2	0.0126102	0	0.0126102
PCDHB3	0.0068035	0	0.0068035
PCDHB4	0.0070073	0	0.0070073
PCDHB5	0.0108215	0	0.0108215
PCDHB6	0.007193	0	0.007193
PCDHB7	0.010351	0	0.010351
PCDHB8	0.0071606	0	0.0071606
PCDHB9	0.0041226	0.0041226	0
PCDHGA1	0.02433	0	0
PCDHGA10	0.0300451	0	0.0300451
PCDHGA11	0.0346145	0	0.0346145
PCDHGA12	0.0175808	0	0.0175808
PCDHGA2	0.0091343	0	0.0091343
PCDHGA3	0.0077969	0	0.0077969
PCDHGA4	0.0064285	0	0.0064285
PCDHGA5	0.0076949	0	0.0076949
PCDHGA6	0.0067794	0	0.0067794
PCDHGA7	0.006631	0	0
PCDHGA8	0.0065586	0	0
PCDHGA9	0.0082132	0	0.0082132
PCDHGB1	0.0071378	0	0
PCDHGB2	0.0088173	0	0
PCDHGB3	0.0072083	0	0
PCDHGB4	0.0070281	0	0.0070281
PCDHGB5	0.0063077	0	0.0063077
PCDHGB6	0.0138175	0	0.0138175
PCDHGB7	0.0296489	0	0.0296489
PCDHGB8P	0.019087	0	0
PCDHGC3	0.0179281	0	0.0179281
PCDHGC4	0.0105153	0.0105153	0
PCDHGC5	0.006026	0	0
PCDP1	0.0040646	0	0
PCF11	0.4897918	0	0.4897918
PCGEM1	0.0082661	0	0.0082661
PCGF1	0.0117987	0.0117987	0
PCGF2	0.084652	0.084652	0
PCGF3	0.0054505	0.0054505	0
PCGF5	0.0431445	0	0.0431445
PCGF6	0.0261135	0	0.0261135
PCID2	0.0493746	0.0493746	0
PCIF1	0.0262092	0.0262092	0
PCK1	0.0046776	0	0

PCK2	0.0252206	0.0252206	0
PCLO	0.0071837	0.0071837	0
PCM1	0.3046584	0	0.3046584
PCMT1	0.0328133	0	0.0328133
PCMTD1	0.1055986	0	0
PCMTD2	0.0179111	0	0
PCNA	0.0903837	0	0
PCNAP1	0.0053885	0	0
PCNP	0.1006967	0	0.1006967
PCNT	0.0143018	0	0
PCNX	0.0801411	0	0.0801411
PCNXL2	0.0962624	0	0
PCNXL3	0.0241315	0.0241315	0
PCOLCE	0.0034465	0	0.0034465
PCOLCE2	0.077346	0	0.077346
PCOTH	0.0173328	0	0
PCP2	0.0029617	0.0029617	0
PCP4	0.0020889	0	0
PCP4L1	0.0156238	0	0.0156238
PCSK1	0.0434931	0	0.0434931
PCSK1N	0.0011169	0.0011169	0
PCSK2	0.0030689	0	0.0030689
PCSK4	0.0051159	0.0051159	0
PCSK5	0.0058487	0	0.0058487
PCSK6	0.0026019	0.0026019	0
PCSK7	0.0189724	0.0189724	0
PCSK9	0.0068652	0.0068652	0
PCTP	0.0389646	0.0389646	0
PCYOX1	0.0246368	0	0.0246368
PCYOX1L	0.0381483	0	0
PCYT1A	0.0767171	0	0.0767171
PCYT1B	0.0151846	0	0.0151846
PDAP1	0.0414896	0.0414896	0
PDC	0.0998652	0	0
PDCD1	0.0030848	0	0.0030848
PDCD10	0.262718	0	0
PDCD11	0.0441876	0	0
PDCD1LG2	0.6179053	0	0.6179053
PDCD2	0.0219846	0	0
PDCD2L	0.0088122	0.0088122	0
PDCD4	0.4282642	0	0.4282642
PDCD5	0.0114354	0.0114354	0
PDCD6	0.0624441	0.0624441	0
PDCD6IP	0.0677913	0	0
PDCD7	0.0233087	0	0.0233087

PDCL	0.066632	0	0.066632
PDCL2	0.0041218	0	0
PDCL3	0.0321972	0	0.0321972
PDDC1	0.0016301	0.0016301	0
PDE10A	0.0023433	0	0.0023433
PDE11A	0.0308229	0.0308229	0
PDE12	0.0604227	0	0.0604227
PDE1A	0.0103044	0	0.0103044
PDE1B	0.0102405	0	0.0102405
PDE1C	0.0060508	0	0.0060508
PDE2A	0.0117864	0	0.0117864
PDE3A	0.0960108	0	0.0960108
PDE3B	0.0299261	0	0.0299261
PDE4A	0.0339123	0	0
PDE4B	0.1592884	0	0.1592884
PDE4C	0.0039713	0.0039713	0
PDE4D	0.1243243	0	0.1243243
PDE4DIP	1	0	1
PDE5A	0.0121256	0	0.0121256
PDE6A	0.5396727	0	0.5396727
PDE6B	0.0006172	0	0.0006172
PDE6C	0.0070518	0	0
PDE6D	0.0500672	0	0
PDE6G	0.0019084	0	0.0019084
PDE6H	0.0188502	0	0
PDE7A	0.0880707	0	0.0880707
PDE7B	0.0435154	0	0.0435154
PDE8A	0.112222	0	0
PDE8B	0.0113215	0	0.0113215
PDE9A	0.0011685	0.0011685	0
PDF	0.025867	0.025867	0
PDGFA	0.0025132	0	0
PDGFB	0.0048225	0	0.0048225
PDGFC	0.0125503	0	0.0125503
PDGFD	0.0080924	0	0.0080924
PDGFRA	0.0054838	0	0.0054838
PDGFRB	0.0124598	0	0.0124598
PDGFRL	0.0175353	0	0.0175353
PDHA1	0.0222962	0.0222962	0
PDHA2	0.0082653	0	0
PDHB	0.0680016	0	0.0680016
PDHX	0.092346	0.092346	0
PDIA2	0.0002922	0	0
PDIA3	0.0297358	0.0297358	0
PDIA3P	0.096189	0.096189	0

PDIA4	0.0244096	0.0244096	0
PDIA5	0.0264076	0	0.0264076
PDIA6	0.0147377	0	0
PDIK1L	0.0180618	0	0
PDILT	0.0036111	0	0
PDK1	0.0624889	0	0
PDK2	0.0129575	0.0129575	0
PDK3	0.0141975	0	0.0141975
PDK4	0.0584712	0	0.0584712
PDLIM1	0.0409996	0	0.0409996
PDLIM2	0.0067605	0	0.0067605
PDLIM3	0.0050655	0	0.0050655
PDLIM4	0.1015965	0	0.1015965
PDLIM5	0.2294989	0	0.2294989
PDLIM7	0.0249326	0	0
PDP1	0.0547692	0	0.0547692
PDP2	0.0137396	0	0
PDPK1	0.0067084	0	0
PDPN	0.0829137	0	0.0829137
PDPR	0.0003437	0	0.0003437
PDRG1	0.019288	0.019288	0
PDS5A	0.0251229	0	0
PDS5B	0.0377457	0	0.0377457
PDSS1	0.0405394	0.0405394	0
PDSS2	0.0337361	0	0
PDX1	0.0268324	0.0268324	0
PDXDC1	0.0407297	0.0407297	0
PDXDC2	0.0021856	0.0021856	0
PDXK	0.0713723	0.0713723	0
PDXP	0.0073616	0	0.0073616
PDYN	0.0022996	0	0.0022996
PDZD11	0.0307335	0.0307335	0
PDZD2	0.0107512	0	0.0107512
PDZD3	0.0015094	0.0015094	0
PDZD4	0.000619	0	0.000619
PDZD7	0.004677	0.004677	0
PDZD8	0.0593251	0	0
PDZK1	0.0255609	0	0
PDZK1IP1	0.2502105	0.2502105	0
PDZRN3	0.0021053	0	0.0021053
PDZRN4	0.0101654	0	0.0101654
PEA15	0.0379059	0	0.0379059
PEAR1	0.0081459	0	0.0081459
PEBP1	0.0120754	0	0
PEBP4	0.0095289	0	0.0095289

PECAM1	0.4585376	0	0.4585376
PECI	0.096989	0	0
PECR	0.0138272	0.0138272	0
PEF1	0.0940451	0	0
PEG10	0.0423069	0	0.0423069
PEG3	0.0024378	0	0.0024378
PEG3AS	0.0015604	0	0
PELI1	0.1673416	0	0
PELI2	0.0075434	0	0.0075434
PELI3	0.0105315	0	0.0105315
PELO	0.1246659	0	0.1246659
PELP1	0.0340486	0.0340486	0
PEMT	0.0071452	0.0071452	0
PENK	0.003736	0	0.003736
PEPD	0.0034637	0	0.0034637
PER1	0.087632	0	0.087632
PER2	0.0554452	0.0554452	0
PER3	0.0103126	0	0
PER4	0.0068409	0	0
PERP	0.10892	0.10892	0
PES1	0.0263686	0.0263686	0
PET112L	0.0354913	0	0
PEX1	0.0695534	0.0695534	0
PEX10	0.0020724	0.0020724	0
PEX11A	0.0696866	0.0696866	0
PEX11B	0.1789229	0.1789229	0
PEX11G	0.011302	0.011302	0
PEX12	0.1167731	0	0.1167731
PEX13	0.2239291	0.2239291	0
PEX14	0.0421231	0.0421231	0
PEX16	0.0129608	0.0129608	0
PEX19	0.0818299	0	0
PEX2	0.0692816	0.0692816	0
PEX26	0.0591092	0	0
PEX3	0.0693046	0	0.0693046
PEX5	0.3863457	0	0
PEX5L	0.0117571	0	0.0117571
PEX6	0.0263389	0.0263389	0
PEX7	0.0180003	0.0180003	0
PF4	0.0099729	0.0099729	0
PF4V1	0.1191656	0	0.1191656
PFAS	0.0299707	0	0.0299707
PFDN1	0.3818454	0	0
PFDN2	0.6814329	0.6814329	0
PFDN4	0.0783646	0.0783646	0

PFDN5	0.0737298	0	0
PFKFB1	0.010574	0	0.010574
PFKFB2	0.4668904	0	0
PFKFB3	0.1138859	0	0.1138859
PFKFB4	0.0224906	0.0224906	0
PFKL	0.012059	0.012059	0
PFKM	0.0995364	0	0
PFKP	0.0089327	0.0089327	0
PFN1	0.1282959	0	0.1282959
PFN2	0.0848663	0	0.0848663
PFN3	0.0085335	0.0085335	0
PFN4	0.0234623	0.0234623	0
PGA3	0.0003133	0	0.0003133
PGA4	1.60E-05	0	1.60E-05
PGA5	3.89E-05	0	3.89E-05
PGAM1	0.0425638	0	0.0425638
PGAM2	0.0175037	0.0175037	0
PGAM5	0.0056013	0.0056013	0
PGAP1	0.0339816	0	0.0339816
PGAP2	0.3215525	0.3215525	0
PGAP3	0.0162564	0.0162564	0
PGBD1	0.0240989	0	0.0240989
PGBD2	0.0680115	0	0
PGBD3	0.020746	0	0.020746
PGBD4	0.0408433	0.0408433	0
PGBD5	0.0784664	0	0.0784664
PGC	0.0065739	0	0
PGCP	0.0044886	0	0.0044886
PGD	0.0228416	0	0.0228416
PGF	0.0072644	0	0.0072644
PGGT1B	0.0548826	0	0
PGK1	0.1602713	0.1602713	0
PGK2	0.0038376	0	0
PGLS	0.0475453	0.0475453	0
PGLYRP1	0.0035269	0	0.0035269
PGLYRP2	0.0025945	0	0
PGLYRP3	0.0055067	0	0
PGLYRP4	0.5299442	0	0.5299442
PGM1	0.0918374	0	0
PGM2	0.0823936	0	0.0823936
PGM2L1	0.0813511	0	0
PGM3	0.1496004	0	0.1496004
PGM5	8.42E-05	0	8.42E-05
PGM5P2	7.49E-05	0	7.49E-05
PGP	0.0141259	0.0141259	0

PGPEP1	0.0167486	0.0167486	0
PGPEP1L	0.0214809	0	0.0214809
PGR	0.0099257	0	0.0099257
PGRMC1	0.0163269	0.0163269	0
PGRMC2	0.0511747	0.0511747	0
PGS1	0.5732495	0	0.5732495
PHACTR1	0.0072801	0	0.0072801
PHACTR2	0.0836446	0	0.0836446
PHACTR3	0.0043703	0.0043703	0
PHACTR4	0.3061889	0	0.3061889
PHAX	0.238454	0	0.238454
PHB	0.0370816	0.0370816	0
PHB2	0.1756359	0	0
PHC1	0.0140028	0	0.0140028
PHC2	0.0830549	0	0.0830549
PHC3	0.0723261	0	0.0723261
PHEX	0.003858	0	0.003858
PHF1	0.0396437	0.0396437	0
PHF10	0.0200271	0	0.0200271
PHF11	0.0540333	0	0.0540333
PHF12	0.0969042	0	0
PHF13	0.0270552	0	0.0270552
PHF14	0.0374428	0	0
PHF15	0.060201	0	0
PHF16	0.0250038	0	0.0250038
PHF17	0.0440191	0	0
PHF19	0.062647	0	0.062647
PHF2	0.0068536	0	0
PHF20	0.0746081	0	0.0746081
PHF20L1	0.0796848	0	0.0796848
PHF21A	0.3251734	0	0.3251734
PHF21B	0.0008885	0	0.0008885
PHF23	0.1240093	0	0.1240093
PHF3	0.1979006	0	0.1979006
PHF5A	0.039909	0	0
PHF6	0.037475	0	0
PHF7	0.012663	0	0.012663
PHF8	0.0220751	0	0
PHGDH	0.0254795	0	0
PHGR1	0.0036157	0.0036157	0
PHIP	0.1386018	0	0.1386018
PHKA1	0.012127	0.012127	0
PHKA2	0.0118896	0.0118896	0
PHKB	0.0594385	0.0594385	0
PHKG1	0.0029382	0	0.0029382

PHKG2	0.0560906	0.0560906	0
PHLDA1	0.1853524	0	0
PHLDA2	0.0093809	0.0093809	0
PHLDA3	0.2225735	0	0
PHLDB1	0.0050125	0	0.0050125
PHLDB2	0.6535949	0	0.6535949
PHLDB3	0.0028763	0.0028763	0
PHLPP1	0.1194532	0.1194532	0
PHLPP2	0.0411555	0	0.0411555
PHOSPHO1	0.0079602	0	0.0079602
PHOSPHO2	0.0161339	0.0161339	0
PHOX2A	0.0026132	0	0.0026132
PHOX2B	0.0044016	0	0.0044016
PHPT1	0.0114101	0.0114101	0
PHRF1	0.0038318	0.0038318	0
PHTF1	0.0727717	0	0.0727717
PHTF2	0.0910886	0	0.0910886
PHYH	0.0109238	0.0109238	0
PHYHD1	0.0090063	0	0.0090063
PHYHIP	0.0074841	0	0.0074841
PHYHIPL	0.0161233	0	0.0161233
PI15	0.0090826	0	0.0090826
PI16	0.0082717	0	0.0082717
PI3	0.9290585	0	0
PI4K2A	0.0729895	0	0.0729895
PI4K2B	0.0463973	0.0463973	0
PI4KA	0.0164725	0	0.0164725
PI4KAP1	1.69E-05	0	0
PI4KAP2	1.55E-05	1.55E-05	0
PI4KB	0.0503028	0	0
PIAS1	0.0683362	0	0.0683362
PIAS2	0.0105019	0	0.0105019
PIAS3	0.0350531	0.0350531	0
PIAS4	0.0046559	0.0046559	0
PIBF1	0.2145664	0	0
PICALM	0.5184703	0	0.5184703
PICK1	0.0042916	0.0042916	0
PID1	0.0102291	0	0.0102291
PIF1	0.0306425	0	0
PIGA	0.0675509	0	0
PIGB	0.0450343	0	0
PIGC	0.1244088	0.1244088	0
PIGF	0.0668968	0.0668968	0
PIGG	0.0147262	0.0147262	0
PIGH	0.0287663	0.0287663	0

PIGK	0.1145326	0	0.1145326
PIGL	0.0867771	0.0867771	0
PIGM	0.1311396	0.1311396	0
PIGN	0.0877081	0	0.0877081
PIGO	0.031098	0.031098	0
PIGP	0.0507714	0.0507714	0
PIGQ	0.0011247	0.0011247	0
PIGR	0.0314259	0	0
PIGS	0.0343631	0.0343631	0
PIGT	0.0687236	0.0687236	0
PIGU	0.0363686	0.0363686	0
PIGV	0.0731794	0	0
PIGW	0.0661148	0	0
PIGX	0.0944919	0	0
PIGY	0.0930909	0	0.0930909
PIGZ	0.008221	0.008221	0
PIH1D1	0.0660452	0.0660452	0
PIH1D2	0.0518253	0.0518253	0
PIK3AP1	0.0203703	0	0.0203703
PIK3C2A	0.0294585	0	0.0294585
PIK3C2B	0.1493852	0.1493852	0
PIK3C2G	0.0224017	0	0
PIK3C3	0.0473017	0	0.0473017
PIK3CA	0.1238446	0	0.1238446
PIK3CB	0.0101942	0.0101942	0
PIK3CD	0.0264484	0	0.0264484
PIK3CG	0.0349678	0	0.0349678
PIK3IP1	0.0666245	0	0.0666245
PIK3R1	0.1339739	0	0.1339739
PIK3R2	0.0178119	0.0178119	0
PIK3R3	0.2334877	0	0.2334877
PIK3R4	0.0496043	0	0.0496043
PIK3R5	0.0409974	0	0.0409974
PIK3R6	0.0516869	0	0.0516869
PIKFYVE	0.1062319	0	0.1062319
PILRA	0.0223459	0	0.0223459
PILRB	0.0140876	0.0140876	0
PIM1	0.0584194	0	0.0584194
PIM2	0.0689958	0	0.0689958
PIM3	0.0199606	0.0199606	0
PIN1	0.0079101	0.0079101	0
PIN1L	0.002861	0	0
PIN4	0.0133223	0.0133223	0
PINK1	0.0131331	0	0
PINX1	0.0485933	0	0.0485933

PION	0.1512646	0.1512646	0
PIP	0.0064065	0.0064065	0
PIP4K2A	0.0472048	0	0.0472048
PIP4K2B	0.0221507	0	0.0221507
PIP4K2C	0.0134356	0.0134356	0
PIP5K1A	0.3783422	0	0
PIP5K1B	0.0133166	0.0133166	0
PIP5K1C	0.01588	0	0
PIP5KL1	0.1038352	0.1038352	0
PIPOX	0.0063894	0	0.0063894
PIPSL	0.0051416	0.0051416	0
PIR	0.0250797	0	0
PIRT	0.0018376	0	0.0018376
PISD	0.0141931	0	0
PISRT1	0.0054143	0	0
PITPNA	0.0064506	0	0.0064506
PITPNB	0.0617801	0	0.0617801
PITPNC1	0.0399286	0	0.0399286
PITPNM1	0.0397971	0.0397971	0
PITPNM2	0.0149017	0	0.0149017
PITPNM3	0.0099837	0.0099837	0
PITRM1	0.0276984	0	0
PITX1	0.0090476	0.0090476	0
PITX2	0.0034777	0	0.0034777
PITX3	0.040564	0	0.040564
PIWIL1	0.0048787	0.0048787	0
PIWIL2	0.1377703	0	0.1377703
PIWIL3	0.0025944	0	0
PIWIL4	0.0170939	0.0170939	0
PJA1	0.0104051	0.0104051	0
PJA2	0.2711029	0	0.2711029
PKD1	0.0012172	0	0
PKD1L1	0.1625116	0	0.1625116
PKD1L2	0.0026331	0	0
PKD1L3	0.0102303	0	0.0102303
PKD2	0.0200427	0	0.0200427
PKD2L1	0.0315664	0	0.0315664
PKD2L2	0.0605593	0	0
PKDCC	0.0017463	0	0
PKDREJ	0.0046711	0	0
PKHD1	0.0031908	0	0.0031908
PKHD1L1	0.0091432	0	0.0091432
PKIA	0.0605731	0	0.0605731
PKIB	0.0336245	0	0
PKIG	0.0538656	0	0.0538656

PKLR	0.0342535	0.0342535	0
PKM2	0.9347314	0.9347314	0
PKMYT1	0.0029761	0.0029761	0
PKN1	0.0067428	0.0067428	0
PKN2	0.0687586	0.0687586	0
PKN3	0.0237265	0	0
PKNOX1	0.0327307	0	0.0327307
PKNOX2	0.0017287	0	0.0017287
PKP1	0.0534835	0	0
PKP2	0.1167936	0.1167936	0
PKP3	0.0009217	0.0009217	0
PKP4	0.0314349	0.0314349	0
PL-5283	0.0740171	0.0740171	0
PLA1A	0.102559	0	0.102559
PLA2G10	4.58E-05	4.58E-05	0
PLA2G12A	0.026462	0.026462	0
PLA2G12B	0.0080282	0	0
PLA2G15	0.0121159	0	0.0121159
PLA2G16	0.0156929	0.0156929	0
PLA2G1B	0.0131248	0	0
PLA2G2A	0.0052019	0	0.0052019
PLA2G2C	0.0059152	0	0
PLA2G2D	0.0128665	0	0.0128665
PLA2G2E	0.0170371	0	0
PLA2G2F	0.0028314	0	0
PLA2G3	0.0052373	0	0
PLA2G4A	0.1380795	0	0
PLA2G4C	0.0183108	0	0.0183108
PLA2G4D	0.2736387	0.2736387	0
PLA2G4E	0.5299045	0	0
PLA2G4F	0.0038269	0.0038269	0
PLA2G5	0.0093464	0	0.0093464
PLA2G6	0.1178482	0.1178482	0
PLA2G7	0.0055464	0	0.0055464
PLA2R1	0.0069792	0	0.0069792
PLAA	0.2098223	0	0.2098223
PLAC1	0.012059	0	0
PLAC1L	0.0176964	0	0
PLAC2	0.0014597	0	0
PLAC4	0.0033995	0.0033995	0
PLAC8	0.0874126	0	0
PLAC8L1	0.0224759	0.0224759	0
PLAC9	0.0026558	0	0.0026558
PLAG1	0.0530174	0	0.0530174
PLAGL1	0.0759728	0	0.0759728

PLAGL2	0.1056624	0	0
PLAT	0.0479903	0	0.0479903
PLAU	0.1543505	0	0.1543505
PLAUR	0.0606001	0	0
PLB1	0.0098337	0	0.0098337
PLBD1	0.0067816	0	0
PLBD2	0.0135299	0	0.0135299
PLCB1	0.0102166	0	0.0102166
PLCB2	0.008451	0	0.008451
PLCB3	0.0078973	0.0078973	0
PLCB4	0.0219313	0.0219313	0
PLCD1	0.0174416	0	0.0174416
PLCD3	0.0317725	0.0317725	0
PLCD4	0.0022332	0	0
PLCE1	0.0105248	0	0
PLCG1	0.0290104	0	0.0290104
PLCG2	0.0184306	0	0.0184306
PLCH1	0.0303204	0.0303204	0
PLCH2	0.0001579	0	0
PLCL1	0.0279088	0	0.0279088
PLCL2	0.0480206	0	0.0480206
PLCXD2	0.1318042	0	0
PLCXD3	0.015606	0	0.015606
PLCZ1	0.0098344	0	0
PLD1	1	0	0
PLD2	0.0045317	0	0
PLD3	0.0175114	0	0.0175114
PLD4	0.0026474	0	0.0026474
PLD5	0.0270937	0	0.0270937
PLD6	0.0060298	0	0
PLDN	0.3927103	0	0.3927103
PLEC	0.2155973	0.2155973	0
PLEK	0.0899533	0	0.0899533
PLEK2	0.0535578	0.0535578	0
PLEKHA1	0.031978	0.031978	0
PLEKHA2	0.091594	0	0.091594
PLEKHA3	0.0737627	0	0.0737627
PLEKHA4	0.1679167	0	0.1679167
PLEKHA5	0.0610159	0.0610159	0
PLEKHA6	0.0223788	0.0223788	0
PLEKHA7	0.0433507	0.0433507	0
PLEKHA8	0.1207642	0	0
PLEKHA9	0.1035528	0	0
PLEKHB1	0.0050558	0.0050558	0
PLEKHB2	0.061959	0	0.061959

PLEKHF1	0.0147893	0	0.0147893
PLEKHF2	0.11432	0	0
PLEKHG1	0.0137758	0	0.0137758
PLEKHG2	0.6992655	0	0.6992655
PLEKHG3	0.0487655	0.0487655	0
PLEKHG4	0.0447373	0.0447373	0
PLEKHG4B	0.0275045	0	0
PLEKHG5	0.008698	0	0
PLEKHG6	0.0112381	0.0112381	0
PLEKHG7	0.0966908	0	0.0966908
PLEKHH1	0.0135766	0.0135766	0
PLEKHH2	0.0128483	0	0
PLEKHH3	0.0856029	0.0856029	0
PLEKHJ1	0.0129675	0.0129675	0
PLEKHM1	0.0005839	0.0005839	0
PLEKHM1P	0.0004539	0.0004539	0
PLEKHM2	0.0413203	0	0.0413203
PLEKHM3	0.0217218	0	0.0217218
PLEKHN1	0.0040537	0.0040537	0
PLEKHO1	0.0523915	0	0.0523915
PLEKHO2	0.0314974	0	0.0314974
PLG	0.0021624	0	0
PLGLA	0.0001046	0	0
PLIN1	0.0209343	0	0.0209343
PLIN2	0.0556705	0	0.0556705
PLIN3	0.2791574	0	0
PLIN4	0.0008325	0	0.0008325
PLIN5	0.0647489	0	0
PLK1	0.0242125	0.0242125	0
PLK1S1	0.0174316	0	0
PLK2	0.1349253	0	0.1349253
PLK3	0.2459881	0	0.2459881
PLK4	0.0473946	0.0473946	0
PLK5P	0.0006776	0.0006776	0
PLLP	0.0323543	0.0323543	0
PLN	0.0165076	0	0.0165076
PLOD1	0.0099253	0	0.0099253
PLOD2	0.4040773	0	0.4040773
PLOD3	0.0085769	0.0085769	0
PLP1	0.0019244	0	0.0019244
PLP2	0.0332989	0.0332989	0
PLRG1	0.0414259	0	0.0414259
PLS1	0.0467546	0.0467546	0
PLS3	0.0701273	0	0.0701273
PLSCR1	0.9484376	0	0.9484376

PLSCR2	0.1526847	0.1526847	0
PLSCR3	0.0317253	0	0.0317253
PLSCR4	0.1038191	0	0.1038191
PLSCR5	0.0149822	0	0
PLTP	0.0170169	0	0.0170169
PLUNC	0.0042758	0.0042758	0
PLVAP	0.0141854	0	0.0141854
PLXDC1	0.068699	0	0.068699
PLXDC2	0.0247251	0	0.0247251
PLXNA1	0.0015326	0	0.0015326
PLXNA2	0.065592	0.065592	0
PLXNA3	0.0006765	0.0006765	0
PLXNA4	0.0113801	0	0.0113801
PLXNB1	0.0409211	0.0409211	0
PLXNB2	0.0033833	0.0033833	0
PLXNB3	0.0009551	0.0009551	0
PLXNC1	0.0179915	0	0.0179915
PLXND1	0.0011809	0	0.0011809
PM20D1	0.0293184	0	0.0293184
PM20D2	0.0627102	0	0
PMAIP1	0.4908528	0	0
PMCH	0.0476344	0	0.0476344
PMCHL1	0.0035785	0	0
PMEPA1	0.0256035	0	0
PMF1	1	1	0
PMFBP1	0.0181784	0	0.0181784
PML	0.1789411	0	0
PMM1	0.0064934	0	0
PMM2	0.0420823	0.0420823	0
PMP2	0.0093423	0	0.0093423
PMP22	0.0124269	0	0.0124269
PMPCA	0.0168488	0.0168488	0
PMPCB	0.0369138	0.0369138	0
PMS1	0.0526663	0	0.0526663
PMS2	0.035598	0.035598	0
PMS2CL	0.0036219	0	0
PMS2L1	0.0055773	0.0055773	0
PMS2L11	4.34E-05	4.34E-05	0
PMS2L3	0.0160704	0.0160704	0
PMS2L4	0.0100585	0.0100585	0
PMS2L5	3.12E-05	3.12E-05	0
PMVK	0.7054117	0.7054117	0
PNCK	0.0007996	0.0007996	0
PNKD	0.0658704	0.0658704	0
PNKP	0.1914196	0.1914196	0

PNLDC1	0.0156961	0	0.0156961
PNLIP	0.0071038	0	0.0071038
PNLIPRP1	0.00434	0	0.00434
PNLIPRP2	0.0018798	0	0.0018798
PNLIPRP3	0.1188986	0	0
PNMA1	0.0291967	0	0
PNMA2	0.004239	0	0
PNMA3	0.000575	0	0.000575
PNMA5	0.0006041	0	0.0006041
PNMAL1	0.0045135	0	0.0045135
PNMAL2	0.0009804	0	0.0009804
PNMT	0.0012877	0.0012877	0
PNN	0.1912199	0.1912199	0
PNO1	0.0936722	0	0.0936722
PNOC	0.0122189	0	0.0122189
PNP	0.0954567	0	0
PNPLA1	0.0113901	0	0.0113901
PNPLA2	0.0097223	0.0097223	0
PNPLA3	0.0082536	0	0
PNPLA4	0.0471821	0.0471821	0
PNPLA5	0.0012757	0	0
PNPLA6	0.0183816	0	0
PNPLA7	0.0071527	0.0071527	0
PNPLA8	0.2455465	0	0.2455465
PNPO	0.0635359	0.0635359	0
PNPT1	0.042535	0.042535	0
PNRC1	0.3866206	0	0.3866206
PNRC2	0.2248262	0	0.2248262
POC1A	0.430215	0.430215	0
POC1B	0.0595912	0.0595912	0
POC5	0.0899211	0	0
PODN	0.0019304	0	0.0019304
PODNL1	0.0081263	0	0.0081263
PODXL	0.0080441	0	0.0080441
PODXL2	0.0070908	0.0070908	0
POF1B	0.0143914	0.0143914	0
POFUT1	0.0999127	0	0.0999127
POFUT2	0.0031357	0.0031357	0
POGK	0.037256	0.037256	0
POGZ	0.0976153	0.0976153	0
POLA1	0.0416123	0	0
POLA2	0.0461887	0.0461887	0
POLB	0.0248019	0.0248019	0
POLD1	0.0366857	0.0366857	0
POLD2	0.0055458	0.0055458	0

POLD3	0.0865957	0	0
POLD4	0.2749443	0.2749443	0
POLDIP2	0.0201768	0.0201768	0
POLDIP3	0.1398763	0	0
POLE	0.0025047	0.0025047	0
POLE2	0.1192559	0.1192559	0
POLE3	0.0846433	0	0.0846433
POLE4	0.0867495	0	0
POLG	0.0879439	0	0
POLG2	0.353545	0.353545	0
POLH	0.0362219	0	0.0362219
POLI	0.0981906	0	0.0981906
POLK	0.1490512	0	0.1490512
POLL	0.0123561	0.0123561	0
POLM	0.0158082	0.0158082	0
POLN	0.0118891	0	0.0118891
POLQ	0.104592	0.104592	0
POLR1A	0.1305966	0	0.1305966
POLR1B	0.0433941	0	0.0433941
POLR1C	0.0513364	0	0
POLR1D	0.042552	0.042552	0
POLR1E	0.0549444	0	0.0549444
POLR2A	0.1640229	0	0.1640229
POLR2B	0.0568904	0	0.0568904
POLR2C	0.019245	0.019245	0
POLR2D	0.0128862	0	0
POLR2E	0.06821	0.06821	0
POLR2F	0.0052672	0.0052672	0
POLR2G	0.0304734	0	0
POLR2H	0.0437461	0.0437461	0
POLR2I	0.0206848	0.0206848	0
POLR2J	5.73E-05	5.73E-05	0
POLR2J2	1.62E-05	0	0
POLR2J3	1.93E-05	1.93E-05	0
POLR2J4	0.0016101	0.0016101	0
POLR2K	0.2494578	0.2494578	0
POLR2L	0.0076347	0.0076347	0
POLR3A	0.3650072	0	0
POLR3B	0.0515513	0	0.0515513
POLR3C	0.0157711	0	0.0157711
POLR3D	0.0374554	0	0
POLR3E	0.0940995	0.0940995	0
POLR3F	0.0223723	0	0.0223723
POLR3G	0.0590878	0	0
POLR3GL	0.141752	0	0

POLR3H	0.00488	0	0.00488
POLR3K	0.0054936	0.0054936	0
POLRMT	0.0056829	0.0056829	0
POM121	0.000377	0.000377	0
POM121C	0.0001631	0.0001631	0
POM121L10P	6.63E-05	6.63E-05	0
POM121L12	0.0007394	0	0
POM121L1P	2.59E-05	0	2.59E-05
POM121L2	0.0265563	0	0
POM121L4P	0.0002285	0	0.0002285
POM121L8P	1.82E-05	0	1.82E-05
POM121L9P	0.0071121	0	0.0071121
POMC	0.0049691	0	0.0049691
POMGNT1	0.0111596	0.0111596	0
POMP	0.0708096	0.0708096	0
POMT1	0.0163769	0.0163769	0
POMT2	0.0423918	0.0423918	0
POMZP3	7.19E-05	7.19E-05	0
PON1	0.0241507	0	0
PON2	0.1407818	0.1407818	0
PON3	0.0145663	0	0
POP1	0.0329299	0	0.0329299
POP4	0.0183199	0.0183199	0
POP5	0.0377704	0.0377704	0
POP7	0.012691	0.012691	0
POPDC2	0.0235007	0	0.0235007
POPDC3	0.066802	0	0.066802
POR	0.0357301	0.0357301	0
PORCN	0.0042658	0.0042658	0
POSTN	0.0073489	0	0.0073489
POT1	0.1545926	0	0
POTEA	0.0060529	0	0
POTEB	1.54E-05	0	0
POTEC	0.0001367	0.0001367	0
POTED	7.92E-05	0	0
POTEE	2.33E-05	0	2.33E-05
POTEF	8.22E-05	0	0
POTEG	1.60E-05	0	0
POTEH	2.32E-05	0	0
POU1F1	0.0075798	0	0
POU2AF1	0.0615693	0	0.0615693
POU2F1	0.2258391	0	0.2258391
POU2F2	0.0194117	0	0.0194117
POU2F3	0.0519989	0	0
POU3F1	0.0052249	0	0.0052249

POU3F2	0.0025314	0	0.0025314
POU3F3	0.003163	0.003163	0
POU3F4	0.0024857	0	0
POU4F1	0.0059251	0	0.0059251
POU4F2	0.0037914	0	0
POU4F3	0.0026614	0	0
POU5F1B	0.0386921	0.0386921	0
POU5F2	0.0104445	0	0
POU6F1	0.0184463	0	0.0184463
POU6F2	0.0100814	0	0
PP14571	0.0002891	0	0
PPA1	0.0754222	0.0754222	0
PPA2	0.0462426	0.0462426	0
PPAN	0.0544389	0.0544389	0
PPAN-P2RY11	0.0533401	0	0
PPAP2A	0.0288627	0	0.0288627
PPAP2B	0.0639848	0	0.0639848
PPAP2C	0.0018259	0.0018259	0
PPAPDC1A	0.0024316	0	0.0024316
PPAPDC1B	0.0582547	0.0582547	0
PPAPDC2	0.1652853	0	0
PPAPDC3	0.0025223	0	0.0025223
PPARA	0.0038937	0	0
PPARD	0.0043137	0	0.0043137
PPARG	0.3497234	0.3497234	0
PPARGC1A	0.0245317	0	0
PPARGC1B	0.0410875	0	0.0410875
PPAT	0.0963419	0	0
PPBP	0.0081842	0	0.0081842
PPBPL2	0.0684089	0	0
PPCDC	0.0436497	0.0436497	0
PPCS	0.0643796	0.0643796	0
PPDPF	0.0049638	0.0049638	0
PPEF1	0.0267672	0	0.0267672
PPEF2	0.0042796	0	0
PPFIA1	0.0108824	0	0
PPFIA2	0.0061881	0	0.0061881
PPFIA3	0.0143348	0.0143348	0
PPFIA4	0.0086532	0.0086532	0
PPFIBP1	0.1169833	0	0.1169833
PPFIBP2	0.0728622	0	0
PPHLN1	0.0886075	0	0.0886075
PPIA	0.0569722	0.0569722	0
PPIAL4B	1.78E-05	0	0
PPIAL4D	2.49E-05	0	0

PPIAL4G	3.10E-05	3.10E-05	0
PPIB	0.0572617	0	0
PPIC	0.0622683	0	0.0622683
PPID	0.0342757	0.0342757	0
PPIE	0.0268917	0.0268917	0
PPIF	0.0217114	0.0217114	0
PPIG	0.0337926	0	0.0337926
PPIH	0.0346004	0	0
PPIL1	0.0908403	0	0
PPIL2	0.0102271	0.0102271	0
PPIL3	0.0935726	0	0
PPIL4	0.0378086	0	0.0378086
PPIL5	0.0572367	0	0
PPIL6	0.0127545	0.0127545	0
PPIP5K1	2.87E-05	2.87E-05	0
PPIP5K2	0.0830921	0	0.0830921
PPL	0.0251835	0.0251835	0
PPM1A	0.0855708	0	0
PPM1B	0.1068346	0	0
PPM1D	0.0977967	0	0.0977967
PPM1E	0.0096623	0	0.0096623
PPM1F	0.0049346	0	0.0049346
PPM1G	0.027951	0.027951	0
PPM1H	0.0131297	0.0131297	0
PPM1J	0.0980447	0	0
PPM1K	0.1138413	0	0.1138413
PPM1L	0.0497144	0	0
PPM1M	0.0508439	0	0.0508439
PPM1N	0.0109449	0.0109449	0
PPME1	0.1569136	0.1569136	0
PPOX	0.0546296	0.0546296	0
PPP1CA	0.0076032	0.0076032	0
PPP1CB	0.1045794	0	0.1045794
PPP1CC	0.0515145	0	0.0515145
PPP1R12A	0.082384	0	0.082384
PPP1R12B	0.284949	0	0.284949
PPP1R12C	0.0138085	0.0138085	0
PPP1R13B	0.0090819	0.0090819	0
PPP1R13L	0.0917399	0.0917399	0
PPP1R14A	0.0026538	0	0.0026538
PPP1R14B	0.019184	0.019184	0
PPP1R14C	0.0116629	0.0116629	0
PPP1R14D	0.016037	0.016037	0
PPP1R15A	0.2648645	0	0
PPP1R15B	1	0	0

PPP1R16A	0.0008533	0.0008533	0
PPP1R16B	0.0179053	0	0.0179053
PPP1R1A	0.0052859	0	0.0052859
PPP1R1B	0.0210504	0.0210504	0
PPP1R1C	0.0419962	0.0419962	0
PPP1R2	0.2417626	0	0.2417626
PPP1R2P3	0.0095651	0	0.0095651
PPP1R2P9	0.0081384	0	0.0081384
PPP1R3A	0.0114161	0	0
PPP1R3B	0.0969567	0	0.0969567
PPP1R3C	0.0484773	0	0
PPP1R3D	0.0849681	0	0
PPP1R3E	0.0621428	0	0
PPP1R3F	0.0125317	0	0
PPP1R3G	0.0254362	0	0
PPP1R7	0.0280771	0.0280771	0
PPP1R8	0.0356145	0	0.0356145
PPP1R9A	0.0240586	0.0240586	0
PPP1R9B	0.0739062	0	0.0739062
PPP2CA	0.2180638	0	0
PPP2CB	0.0632161	0	0.0632161
PPP2R1A	0.0246106	0.0246106	0
PPP2R1B	0.0280027	0	0
PPP2R2A	0.0963163	0	0
PPP2R2B	0.0222054	0	0.0222054
PPP2R2C	0.0020381	0.0020381	0
PPP2R2D	0.0074288	0	0
PPP2R3A	0.0148538	0	0.0148538
PPP2R3C	0.102262	0	0.102262
PPP2R4	0.0365681	0.0365681	0
PPP2R5A	0.5500099	0.5500099	0
PPP2R5B	0.0247853	0.0247853	0
PPP2R5C	0.1152351	0	0.1152351
PPP2R5D	0.0230663	0	0.0230663
PPP2R5E	0.0402905	0	0.0402905
PPP3CA	0.1577021	0	0
PPP3CB	0.0587279	0	0.0587279
PPP3CC	0.0709129	0	0.0709129
PPP3R1	0.0974699	0	0.0974699
PPP3R2	0.0096178	0	0.0096178
PPP4C	0.0163658	0.0163658	0
PPP4R1	0.0576066	0.0576066	0
PPP4R1L	0.1259149	0	0
PPP4R2	0.0351923	0	0.0351923
PPP4R4	0.0344198	0	0.0344198

PPP5C	0.0187964	0.0187964	0
PPP6C	0.0554807	0	0.0554807
PPPDE1	0.1421266	0	0.1421266
PPPDE2	0.0541583	0	0
PPRC1	0.035352	0	0
PPT1	0.0217213	0	0.0217213
PPTC7	0.2763913	0	0.2763913
PPWD1	0.0632965	0	0.0632965
PPY	0.0071543	0	0.0071543
PPY2	0.0028497	0	0.0028497
PPYR1	0.0754438	0	0.0754438
PQBP1	0.0188709	0.0188709	0
PQLC1	0.0055955	0	0.0055955
PQLC2	0.0091714	0.0091714	0
PQLC3	0.0043671	0	0.0043671
PRAC	0.0031465	0	0
PRAF2	0.003826	0	0.003826
PRAM1	0.0006972	0	0.0006972
PRAME	0.0025379	0	0.0025379
PRAMEF1	0.0017962	0.0017962	0
PRAMEF10	2.02E-05	0	0
PRAMEF11	0.0009843	0	0
PRAMEF12	0.0042712	0.0042712	0
PRAMEF13	1.53E-05	0	0
PRAMEF16	1.53E-05	0	0
PRAMEF17	1.53E-05	0	0
PRAMEF2	0.0019092	0	0
PRAMEF3	1.53E-05	0	0
PRAMEF4	3.21E-05	0	0
PRAP1	0.0011619	0	0
PRB1	0.0042893	0	0
PRB2	0.0034483	0	0
PRB3	0.0062064	0	0
PRB4	0.001393	0	0.001393
PRC1	0.1224354	0.1224354	0
PRCC	0.2158999	0.2158999	0
PRCD	0.0238449	0	0.0238449
PRCP	0.1568119	0	0.1568119
PRDM1	0.4784858	0	0.4784858
PRDM10	0.0498224	0	0
PRDM11	0.020245	0	0.020245
PRDM12	0.0007524	0.0007524	0
PRDM13	0.0093028	0	0
PRDM14	0.0025914	0	0
PRDM15	0.0259142	0	0.0259142

PRDM16	0.000171	0.000171	0
PRDM2	0.0668232	0	0.0668232
PRDM4	0.0476762	0	0.0476762
PRDM5	0.0076925	0	0.0076925
PRDM6	0.0048994	0	0.0048994
PRDM7	0.0119231	0.0119231	0
PRDM8	0.0093363	0	0
PRDM9	0.0124776	0	0
PRDX1	0.072829	0	0
PRDX2	0.503852	0.503852	0
PRDX3	0.0324442	0.0324442	0
PRDX4	0.0218325	0	0
PRDX5	0.0647457	0.0647457	0
PRDX6	0.2802487	0	0
PRDXDD1P	0.0141235	0.0141235	0
PREB	0.0063314	0	0
PRELID1	0.2527417	0.2527417	0
PRELID2	0.065527	0.065527	0
PRELP	0.0386599	0	0.0386599
PREP	0.0279581	0	0
PREPL	0.0915759	0	0
PREX1	0.0649355	0	0.0649355
PREX2	0.0074982	0	0.0074982
PRF1	0.0032058	0	0.0032058
PRG1	0.0559306	0	0
PRG2	0.0084019	0	0.0084019
PRG3	0.0034625	0	0
PRG4	0.2943401	0	0.2943401
PRH1	0.1046977	0	0
PRH2	0.016121	0.016121	0
PRHOXNB	0.0054072	0	0
PRIC285	0.0094867	0.0094867	0
PRICKLE1	0.0294306	0	0.0294306
PRICKLE2	0.0209806	0	0.0209806
PRICKLE3	0.014443	0.014443	0
PRICKLE4	0.0184301	0.0184301	0
PRIM1	0.0401143	0	0
PRIM2	0.1138363	0	0
PRIMA1	0.0035917	0	0.0035917
PRINS	0.2115361	0	0
PRKAA1	0.0779875	0	0
PRKAA2	0.0216886	0.0216886	0
PRKAB1	0.0829995	0.0829995	0
PRKAB2	0.2369436	0	0
PRKACA	0.0073584	0	0.0073584

PRKACB	0.0296554	0	0.0296554
PRKACG	0.0036179	0	0
PRKAG1	0.0366449	0.0366449	0
PRKAG2	0.121613	0	0
PRKAG3	0.0175263	0	0
PRKAR1A	0.0664584	0	0.0664584
PRKAR1B	0.0066097	0.0066097	0
PRKAR2A	0.0059787	0	0.0059787
PRKAR2B	0.0082466	0	0.0082466
PRKCA	0.0193464	0	0
PRKCB	0.0343351	0	0.0343351
PRKCD	0.013328	0.013328	0
PRKCDBP	0.0566076	0	0
PRKCE	0.0602768	0	0.0602768
PRKCG	0.0021234	0.0021234	0
PRKCH	0.0733413	0	0.0733413
PRKCI	0.1414861	0.1414861	0
PRKCQ	0.0100959	0	0.0100959
PRKCSH	0.0167473	0.0167473	0
PRKCZ	0.0009332	0.0009332	0
PRKD1	0.1157145	0	0.1157145
PRKD2	0.0129963	0.0129963	0
PRKD3	0.1086193	0	0.1086193
PRKDC	0.0457394	0	0.0457394
PRKG1	0.0093773	0	0.0093773
PRKG2	0.0144172	0.0144172	0
PRKRA	0.0903371	0	0
PRKRIP1	0.0522492	0.0522492	0
PRKRIR	0.075883	0	0.075883
PRKX	0.006621	0	0.006621
PRKY	1.53E-05	0	0
PRL	0.0275421	0	0.0275421
PRLH	0.004952	0	0
PRLHR	0.0073989	0	0.0073989
PRLR	0.0421416	0	0
PRM1	0.0028242	0	0
PRM2	0.0025572	0	0
PRM3	0.0025835	0	0
PRMT1	0.049296	0.049296	0
PRMT10	0.017924	0	0.017924
PRMT2	0.0948428	0	0.0948428
PRMT3	0.0557907	0	0
PRMT5	0.1564254	0.1564254	0
PRMT6	0.0313009	0	0
PRMT7	0.0223954	0.0223954	0

PRMT8	0.0024763	0	0
PRND	0.0320452	0	0.0320452
PRNP	0.4139979	0	0.4139979
PRNT	0.0074983	0	0
PRO0611	0.0574881	0	0
PRO0628	0.294026	0	0
PRO1768	0.0046462	0	0.0046462
PROC	0.0223784	0.0223784	0
PROCA1	0.045516	0	0
PROCR	0.0290827	0	0.0290827
PRODH	0.0009269	0	0
PRODH2	0.0026475	0	0
PROK1	0.007408	0	0.007408
PROK2	0.0027311	0	0.0027311
PROKR1	0.005726	0	0
PROKR2	0.0018674	0	0.0018674
PROL1	0.0202671	0.0202671	0
PROM1	0.004189	0	0
PROM2	0.0049547	0.0049547	0
PROP1	0.0029905	0	0
PROS1	0.1278772	0	0.1278772
ProSAPiP1	0.0040599	0.0040599	0
PROSC	0.0272288	0	0.0272288
PROX1	0.0134749	0	0
PROX2	0.0161688	0	0.0161688
PROZ	0.003407	0.003407	0
PRPF18	0.0198897	0	0
PRPF19	0.1822258	0.1822258	0
PRPF3	0.0490502	0.0490502	0
PRPF31	0.0078192	0.0078192	0
PRPF38A	0.0746617	0	0.0746617
PRPF38B	0.1113902	0	0.1113902
PRPF39	0.0576616	0.0576616	0
PRPF4	0.0983224	0	0.0983224
PRPF40A	0.0635089	0	0
PRPF40B	0.0406372	0.0406372	0
PRPF4B	0.1958954	0	0.1958954
PRPF6	0.0421621	0.0421621	0
PRPF8	0.0137083	0	0.0137083
PRPH	0.0053871	0	0.0053871
PRPH2	0.0092821	0	0.0092821
PRPS1	0.0363714	0	0.0363714
PRPS1L1	0.0170421	0	0
PRPS2	0.0143762	0	0
PRPSAP1	0.0104067	0.0104067	0

PRPSAP2	0.0569568	0	0
PRR11	0.2726251	0	0
PRR12	0.0068952	0.0068952	0
PRR13	0.0954182	0.0954182	0
PRR14	0.0362871	0.0362871	0
PRR15	0.0375894	0.0375894	0
PRR15L	0.0164713	0.0164713	0
PRR16	0.0349488	0	0.0349488
PRR18	0.0044179	0	0
PRR19	0.0075914	0.0075914	0
PRR21	0.0032639	0	0
PRR22	0.005811	0.005811	0
PRR23A	0.0036203	0.0036203	0
PRR23B	0.0030822	0	0
PRR23C	0.0035572	0	0
PRR24	0.0087187	0	0
PRR25	0.001528	0.001528	0
PRR4	0.1047499	0.1047499	0
PRR5	0.0032973	0	0
PRR5-ARHGA	0.0018847	0.0018847	0
PRR5L	0.1458243	0	0
PRR7	0.0392588	0.0392588	0
PRRC1	0.0398852	0	0
PRRG1	0.0281311	0	0
PRRG2	0.0361464	0.0361464	0
PRRG3	0.001507	0	0.001507
PRRG4	0.1595294	0.1595294	0
PRRT2	0.3477627	0	0
PRRT3	0.0133127	0	0
PRRT4	0.0024663	0	0.0024663
PRRX1	0.0144334	0	0.0144334
PRRX2	0.0021943	0	0.0021943
PRSS1	0.0100703	0	0
PRSS12	0.0071513	0.0071513	0
PRSS16	0.0134716	0.0134716	0
PRSS21	0.0045517	0	0
PRSS22	0.0427096	0.0427096	0
PRSS23	0.1644816	0	0.1644816
PRSS27	0.0052206	0.0052206	0
PRSS3	0.0457428	0	0
PRSS33	0.0371737	0	0
PRSS35	0.0178763	0	0.0178763
PRSS36	0.0110007	0	0.0110007
PRSS37	0.0052706	0	0
PRSS38	0.0895508	0	0

PRSS42	0.0095275	0.0095275	0
PRSS45	0.0033945	0.0033945	0
PRSS48	0.0050459	0	0
PRSS50	0.0029	0.0029	0
PRSS8	0.013258	0.013258	0
PRSSL1	0.0006561	0	0.0006561
PRTFDC1	0.1106509	0	0
PRTG	0.0165234	0	0.0165234
PRTN3	0.0027253	0	0
PRUNE	0.0811418	0.0811418	0
PRUNE2	0.0113236	0	0.0113236
PRX	0.0466058	0	0.0466058
PSAP	0.0809223	0	0.0809223
PSAPL1	0.0010637	0	0
PSAT1	0.0383285	0	0
PSCA	0.0684993	0	0
PSD	0.0054593	0	0.0054593
PSD2	0.003579	0	0.003579
PSD3	0.0298251	0	0.0298251
PSD4	0.0103552	0.0103552	0
PSEN1	0.0253786	0.0253786	0
PSEN2	0.0459936	0.0459936	0
PSENEN	0.2243028	0.2243028	0
PSG1	0.0066641	0	0
PSG10	0.004868	0	0
PSG11	0.0048047	0	0
PSG2	0.0014454	0	0
PSG3	0.0003567	0	0.0003567
PSG4	0.0014594	0	0.0014594
PSG5	0.0032875	0	0.0032875
PSG6	0.001818	0	0
PSG7	0.0018671	0	0.0018671
PSG8	0.0004325	0	0
PSG9	0.0041554	0	0
PSIMCT-1	0.0327932	0	0
PSIP1	0.0958187	0	0.0958187
psiTPTE22	0.0010134	0	0.0010134
PSKH1	0.0100602	0	0.0100602
PSKH2	0.0021664	0	0
PSMA1	0.1041297	0	0
PSMA2	0.1180608	0.1180608	0
PSMA3	0.1418438	0	0
PSMA4	0.0802149	0	0
PSMA5	0.1374555	0	0
PSMA6	0.1997412	0	0

PSMA7	0.0137064	0.0137064	0
PSMA8	0.007206	0	0.007206
PSMB1	0.0664335	0	0
PSMB10	0.0088622	0.0088622	0
PSMB11	0.0079725	0	0
PSMB2	0.0594897	0	0
PSMB3	0.2898207	0.2898207	0
PSMB4	0.0847832	0.0847832	0
PSMB5	0.0308984	0.0308984	0
PSMB6	0.1253111	0.1253111	0
PSMB7	0.1198555	0.1198555	0
PSMC1	0.0245961	0.0245961	0
PSMC2	0.154757	0	0
PSMC3	0.0590708	0.0590708	0
PSMC3IP	0.0565082	0.0565082	0
PSMC4	0.0272632	0.0272632	0
PSMC5	0.042421	0.042421	0
PSMC6	0.5685526	0.5685526	0
PSMD1	0.0254149	0	0
PSMD10	0.0321865	0	0
PSMD11	0.0297623	0	0
PSMD12	0.0923163	0	0
PSMD13	0.0175884	0.0175884	0
PSMD14	0.0483878	0	0
PSMD2	0.0260177	0	0
PSMD3	0.1924324	0.1924324	0
PSMD4	0.06881	0.06881	0
PSMD5	0.0862423	0	0.0862423
PSMD6	0.0343174	0	0
PSMD7	0.0986903	0.0986903	0
PSMD8	0.0451641	0.0451641	0
PSMD9	0.0648314	0.0648314	0
PSME1	0.0285866	0.0285866	0
PSME2	0.0492954	0.0492954	0
PSME3	0.0651563	0.0651563	0
PSME4	0.0679991	0	0.0679991
PSMF1	0.0281364	0.0281364	0
PSMG1	0.0731126	0	0
PSMG2	0.0387902	0.0387902	0
PSMG3	0.0317017	0.0317017	0
PSMG4	0.0156368	0.0156368	0
PSPC1	0.0075073	0.0075073	0
PSPH	0.0558375	0.0558375	0
PSPN	0.0018629	0.0018629	0
PSRC1	0.0527523	0.0527523	0

PSTK	0.0316275	0	0
PSTPIP1	0.0117429	0	0.0117429
PSTPIP2	0.0292648	0	0.0292648
PTAFR	0.1165618	0	0.1165618
PTAR1	0.0275259	0	0.0275259
PTBP1	0.0122649	0.0122649	0
PTBP2	0.0282075	0	0
PTCD1	0.0089276	0.0089276	0
PTCD2	0.0763848	0	0.0763848
PTCD3	0.1426558	0.1426558	0
PTCH1	0.0726791	0	0.0726791
PTCH2	0.0050939	0	0.0050939
PTCHD1	0.0036449	0	0.0036449
PTCHD2	0.003343	0	0
PTCHD3	0.0015716	0	0.0015716
PTCRA	0.0027236	0	0.0027236
PTDSS1	0.232757	0	0.232757
PTDSS2	0.0166967	0.0166967	0
PTEN	0.2957508	0	0.2957508
PTENP1	0.0037222	0	0.0037222
PTER	0.0359489	0.0359489	0
PTF1A	0.0012114	0	0
PTGDR	0.0082356	0	0.0082356
PTGDS	0.0014552	0	0.0014552
PTGER1	0.0016953	0.0016953	0
PTGER2	0.015965	0	0.015965
PTGER3	0.0085236	0	0.0085236
PTGER4	0.5420785	0	0.5420785
PTGES	0.1625439	0	0
PTGES2	0.0489277	0.0489277	0
PTGES3	0.0688491	0.0688491	0
PTGFR	0.0115136	0	0.0115136
PTGFRN	0.1899452	0	0.1899452
PTGIR	0.0079112	0	0.0079112
PTGIS	0.0085347	0	0.0085347
PTGR1	0.0268779	0	0.0268779
PTGR2	0.139672	0.139672	0
PTGS1	0.019137	0	0.019137
PTGS2	0.3959309	0	0
PTH	0.0109904	0	0
PTH1R	0.0011883	0	0.0011883
PTH2	0.0024859	0	0
PTH2R	0.0062889	0	0.0062889
PTHLH	0.0463722	0	0.0463722
PTK2	0.1530029	0.1530029	0

PTK2B	0.2087501	0	0.2087501
PTK6	0.0325362	0.0325362	0
PTK7	0.004343	0	0.004343
PTMA	0.0954143	0.0954143	0
PTMS	0.0354077	0	0
PTN	0.020587	0	0.020587
PTOV1	0.0351346	0.0351346	0
PTP4A1	0.7535688	0	0
PTP4A2	0.6425084	0	0.6425084
PTP4A3	0.0013739	0	0.0013739
PTPDC1	0.0108822	0	0.0108822
PTPLA	0.0122565	0	0
PTPLAD1	0.031774	0.031774	0
PTPLAD2	0.0110249	0	0.0110249
PTPLB	0.1835119	0	0.1835119
PTPMT1	0.0496737	0.0496737	0
PTPN1	0.337021	0	0.337021
PTPN11	0.0626147	0	0.0626147
PTPN12	0.0906902	0	0
PTPN13	0.0857903	0	0.0857903
PTPN14	0.1266472	0	0.1266472
PTPN18	0.0056806	0.0056806	0
PTPN2	0.3280014	0	0
PTPN21	0.0193581	0	0.0193581
PTPN22	0.1676427	0	0.1676427
PTPN23	0.0336474	0.0336474	0
PTPN3	0.3001316	0.3001316	0
PTPN4	0.1682877	0	0.1682877
PTPN5	0.0044939	0	0.0044939
PTPN6	0.2587544	0	0.2587544
PTPN7	0.0285717	0	0.0285717
PTPN9	0.0239702	0	0.0239702
PTPRA	0.0784614	0	0.0784614
PTPRB	0.1071221	0	0.1071221
PTPRC	0.2042566	0	0.2042566
PTPRCAP	0.0295521	0	0.0295521
PTPRD	0.0072548	0	0.0072548
PTPRE	0.0476962	0	0.0476962
PTPRF	0.0196267	0.0196267	0
PTPRG	0.083605	0	0.083605
PTPRH	0.088798	0.088798	0
PTPRJ	0.048906	0	0
PTPRK	0.059571	0	0
PTPRM	0.0145978	0	0.0145978
PTPRN	0.0023537	0	0.0023537

PTPRN2	0.0048299	0.0048299	0
PTPRO	0.0377339	0	0.0377339
PTPRQ	0.0054118	0	0.0054118
PTPRR	0.0543701	0.0543701	0
PTPRS	0.0016111	0	0.0016111
PTPRT	0.0031354	0	0.0031354
PTPRU	0.0187238	0.0187238	0
PTPRVP	0.0080078	0	0
PTPRZ1	0.0148799	0	0
PTRF	0.0485589	0	0.0485589
PTRH1	0.0364744	0.0364744	0
PTRH2	0.0988538	0	0
PTS	0.040735	0.040735	0
PTTG1	0.2050354	0.2050354	0
PTTG1IP	0.6529297	0	0
PTTG2	0.0055649	0	0
PTTG3P	0.0156517	0.0156517	0
PTX3	0.0480097	0	0.0480097
PTX4	0.0016465	0	0.0016465
PUF60	0.0737401	0.0737401	0
PUM1	0.031047	0	0
PUM2	0.0581238	0	0
PURA	0.1066063	0	0.1066063
PURB	0.0685203	0	0
PURG	0.047665	0	0.047665
PUS1	0.0066355	0.0066355	0
PUS10	0.2133887	0	0.2133887
PUS3	0.157081	0	0
PUS7	0.014607	0.014607	0
PUS7L	0.6387476	0	0.6387476
PUSL1	0.0026132	0.0026132	0
PVALB	0.0019471	0	0.0019471
PVR	0.1918625	0.1918625	0
PVRIG	0.0117029	0	0.0117029
PVRL1	0.0087395	0.0087395	0
PVRL2	0.002564	0.002564	0
PVRL3	0.0308707	0	0
PVRL4	0.1320785	0.1320785	0
PVT1	0.2004817	0.2004817	0
PWP1	0.0413228	0	0
PWP2	0.0105686	0	0
PWRN1	0.0014457	0	0
PWRN2	0.0009094	0	0
PWWP2A	0.0359969	0	0
PWWP2B	0.0006776	0.0006776	0

PXDN	0.0026572	0	0.0026572
PXDNL	0.022458	0	0.022458
PXK	0.0852743	0	0.0852743
PXMP2	0.0024826	0.0024826	0
PXMP4	0.0185477	0.0185477	0
PXN	0.0835168	0.0835168	0
PXT1	0.0912044	0	0
PYCARD	0.0210404	0.0210404	0
PYCR2	0.1623589	0.1623589	0
PYCRL	0.0530949	0.0530949	0
PYDC1	0.0028376	0	0
PYDC2	0.0087867	0	0
PYGB	0.0311529	0.0311529	0
PYGL	0.0415295	0	0.0415295
PYGM	0.0068737	0	0.0068737
PYGO1	0.0073898	0	0.0073898
PYGO2	0.0703299	0.0703299	0
PYHIN1	0.0238742	0	0.0238742
PYROXD1	0.0699992	0.0699992	0
PYROXD2	0.0051351	0.0051351	0
PYY	0.0080611	0	0
PYY2	0.0035072	0	0
PZP	0.0302162	0	0.0302162
QARS	0.0067098	0.0067098	0
QDPR	0.0260659	0	0
QKI	0.0629737	0	0.0629737
QPCT	0.0189841	0	0.0189841
QPCTL	0.0143007	0.0143007	0
QPRT	0.0036823	0	0.0036823
QRFP	0.0026921	0	0
QRFPR	0.0278661	0	0
QRICH1	0.0205107	0	0.0205107
QRICH2	0.0026958	0	0
QRSL1	0.0629243	0	0.0629243
QSER1	0.0908188	0	0.0908188
QSOX1	0.0918905	0.0918905	0
QSOX2	0.0101627	0	0
QTRT1	0.0100928	0.0100928	0
QTRTD1	0.0641188	0	0
R3HCC1	0.0159649	0	0
R3HDM1	0.0845131	0	0.0845131
R3HDM2	0.0128916	0	0
R3HDML	0.0074592	0.0074592	0
RAB10	0.0440597	0.0440597	0
RAB11A	0.1066343	0.1066343	0

RAB11B	0.0316571	0.0316571	0
RAB11FIP1	0.0775701	0.0775701	0
RAB11FIP2	0.0723989	0	0.0723989
RAB11FIP3	0.0137181	0	0.0137181
RAB11FIP4	0.0023031	0.0023031	0
RAB11FIP5	0.0153291	0	0
RAB12	0.0798593	0	0.0798593
RAB13	0.4274132	0	0
RAB14	0.3243545	0	0.3243545
RAB15	0.0036242	0.0036242	0
RAB17	0.0185183	0.0185183	0
RAB18	0.0563045	0	0.0563045
RAB19	0.0079927	0.0079927	0
RAB1A	0.043023	0	0
RAB1B	0.0208595	0.0208595	0
RAB20	0.0222262	0.0222262	0
RAB21	0.0851036	0	0.0851036
RAB22A	0.1274746	0.1274746	0
RAB23	0.0749345	0	0.0749345
RAB24	0.2652414	0.2652414	0
RAB25	0.0401465	0.0401465	0
RAB26	0.0268337	0.0268337	0
RAB27A	0.5171346	0	0.5171346
RAB27B	0.0985799	0.0985799	0
RAB28	0.0368905	0	0.0368905
RAB2A	0.0505654	0.0505654	0
RAB2B	0.0879938	0	0.0879938
RAB30	0.4499582	0	0.4499582
RAB31	0.0570501	0	0.0570501
RAB32	0.0327886	0	0.0327886
RAB33A	0.0393745	0	0.0393745
RAB33B	0.0594986	0	0.0594986
RAB34	0.2589981	0	0.2589981
RAB35	0.1090521	0	0.1090521
RAB36	0.0156284	0	0
RAB37	0.013083	0	0.013083
RAB38	0.3235357	0	0
RAB39	0.008444	0	0.008444
RAB39B	0.0080348	0	0.0080348
RAB3A	0.0120282	0	0
RAB3B	0.004582	0	0
RAB3C	0.0079036	0	0.0079036
RAB3D	0.0372199	0.0372199	0
RAB3GAP1	0.0386575	0	0.0386575
RAB3GAP2	0.1428239	0	0

RAB3IL1	0.0055206	0	0.0055206
RAB3IP	0.0539506	0.0539506	0
RAB40A	0.0096396	0	0
RAB40AL	0.0064168	0	0
RAB40B	0.0027273	0.0027273	0
RAB40C	0.0132534	0.0132534	0
RAB41	0.0108895	0	0
RAB42	0.0224125	0	0.0224125
RAB43	0.0285822	0	0
RAB4A	0.0715106	0.0715106	0
RAB4B	0.029192	0.029192	0
RAB5A	0.1763651	0	0
RAB5B	0.2041154	0	0
RAB5C	0.0065621	0	0.0065621
RAB6A	0.0690516	0	0
RAB6B	0.0035433	0	0.0035433
RAB6C	0.0011784	0.0011784	0
RAB7A	0.0735337	0	0.0735337
RAB7L1	0.3292651	0	0.3292651
RAB8A	0.0441655	0	0
RAB8B	0.2044655	0	0.2044655
RAB9A	0.1770314	0	0.1770314
RAB9B	0.0067307	0	0.0067307
RABAC1	0.0042689	0.0042689	0
RABEP1	0.0168072	0	0.0168072
RABEP2	0.0250046	0.0250046	0
RABEPK	0.0795243	0	0
RABGAP1	0.1500393	0	0
RABGAP1L	0.1952916	0	0.1952916
RABGEF1	0.0331455	0	0
RABGGTA	0.3269373	0.3269373	0
RABGGTB	0.2576159	0.2576159	0
RABIF	0.132437	0	0
RABL2A	0.0001588	0.0001588	0
RABL2B	0.0001373	0	0
RABL3	0.0302959	0	0.0302959
RABL5	0.0070249	0.0070249	0
RAC1	0.0861952	0.0861952	0
RAC2	0.0132599	0	0.0132599
RAC3	0.0025715	0.0025715	0
RACGAP1	0.1141164	0.1141164	0
RACGAP1P	0.0110242	0.0110242	0
RAD1	0.2955576	0	0.2955576
RAD18	0.1015096	0	0
RAD21	0.3071393	0	0

RAD21L1	0.0014588	0	0
RAD23A	0.1099273	0.1099273	0
RAD23B	0.7081408	0	0
RAD50	0.1036685	0	0
RAD51	0.0523825	0.0523825	0
RAD51AP1	0.1876894	0.1876894	0
RAD51AP2	0.0249004	0	0.0249004
RAD51C	0.042295	0	0.042295
RAD51L1	0.0761497	0	0.0761497
RAD51L3	0.0094657	0	0.0094657
RAD52	0.1636754	0.1636754	0
RAD54B	0.047153	0.047153	0
RAD54L	0.0154568	0.0154568	0
RAD54L2	0.0205428	0	0.0205428
RAD9A	0.0088621	0.0088621	0
RAD9B	0.0958867	0	0
RADIL	0.0008726	0	0.0008726
RAE1	0.0897189	0.0897189	0
RAET1E	0.1580693	0	0
RAET1G	0.0049391	0	0
RAET1K	0.0075051	0	0
RAET1L	0.0127868	0	0.0127868
RAF1	0.0525621	0	0
RAG1	0.016314	0	0
RAG1AP1	0.703047	0.703047	0
RAG2	0.0358237	0	0
RAGE	0.0111634	0.0111634	0
RAI1	0.0387739	0.0387739	0
RAI14	0.0516033	0	0.0516033
RAI2	0.0028182	0	0.0028182
RALA	0.1463348	0	0
RALB	0.015356	0	0
RALBP1	0.1237253	0.1237253	0
RALGAPA1	0.0493561	0	0
RALGAPA2	0.1102145	0	0
RALGAPB	0.0716407	0	0
RALGDS	0.1329854	0	0.1329854
RALGPS1	0.0125195	0.0125195	0
RALGPS2	0.1141367	0	0
RALY	0.094023	0.094023	0
RALYL	0.0080648	0	0
RAMP1	0.0070875	0	0
RAMP2	0.0058168	0	0.0058168
RAMP3	0.0027052	0	0.0027052
RAN	0.0452956	0	0

RANBP1	0.0107731	0.0107731	0
RANBP10	0.0243572	0	0.0243572
RANBP17	0.0183538	0	0
RANBP2	0.0044554	0	0.0044554
RANBP3	0.0192326	0.0192326	0
RANBP3L	0.0487918	0	0.0487918
RANBP6	0.3476572	0	0.3476572
RANBP9	0.0421588	0	0.0421588
RANGAP1	0.1687336	0.1687336	0
RANGRF	0.0103232	0	0.0103232
RAP1A	0.2857935	0	0.2857935
RAP1B	0.0947168	0	0.0947168
RAP1GAP	0.0045004	0.0045004	0
RAP1GAP2	0.0011837	0.0011837	0
RAP1GDS1	0.0396713	0	0.0396713
RAP2A	0.1657534	0	0.1657534
RAP2B	0.1509954	0	0
RAP2C	0.0344899	0	0.0344899
RAPGEF1	0.2289705	0	0.2289705
RAPGEF2	0.0124948	0	0.0124948
RAPGEF3	0.0818176	0.0818176	0
RAPGEF4	0.0133984	0	0.0133984
RAPGEF5	0.0602642	0	0.0602642
RAPGEF6	0.2873372	0	0.2873372
RAPGEFL1	0.0119276	0.0119276	0
RAPH1	0.0502871	0	0
RAPSN	0.0116162	0	0
RARA	0.1776418	0	0.1776418
RARB	0.0101878	0	0.0101878
RARG	0.037441	0.037441	0
RARRES1	0.0852042	0	0.0852042
RARRES2	0.0022454	0	0.0022454
RARRES3	0.0161308	0	0.0161308
RARS	0.0912277	0.0912277	0
RARS2	0.0597632	0	0.0597632
RASA1	0.0625465	0	0
RASA2	0.1994717	0	0.1994717
RASA3	0.0057529	0	0.0057529
RASA4	1.77E-05	0	1.77E-05
RASA4P	0.0019467	0	0
RASAL1	0.0044131	0.0044131	0
RASAL2	0.2782164	0	0.2782164
RASAL3	0.0080185	0	0.0080185
RASD1	0.0021738	0	0.0021738
RASD2	0.0026118	0	0.0026118

RASEF	0.0307002	0	0
RASGEF1A	0.0011823	0	0.0011823
RASGEF1B	0.0136361	0	0.0136361
RASGEF1C	0.00826	0	0.00826
RASGRF1	0.0079331	0	0.0079331
RASGRF2	0.0197283	0	0.0197283
RASGRP1	0.0229418	0	0.0229418
RASGRP2	0.0025366	0	0.0025366
RASGRP3	0.0846379	0	0.0846379
RASGRP4	0.0058601	0	0.0058601
RASIP1	0.0046546	0	0.0046546
RASL10A	0.0028336	0	0
RASL10B	0.00392	0	0.00392
RASL11A	0.0318988	0	0.0318988
RASL11B	0.0048219	0	0.0048219
RASL12	0.0032218	0	0.0032218
RASSF1	0.0199804	0	0.0199804
RASSF10	0.039597	0.039597	0
RASSF2	0.0266491	0	0.0266491
RASSF3	0.1784079	0	0.1784079
RASSF4	0.0382306	0	0.0382306
RASSF5	0.8164214	0	0.8164214
RASSF6	0.0366176	0.0366176	0
RASSF7	0.0012059	0.0012059	0
RASSF8	0.0402535	0	0.0402535
RASSF9	0.0895819	0	0.0895819
RAVER1	0.0089682	0.0089682	0
RAVER2	0.0084419	0.0084419	0
RAX	0.0028741	0	0
RAX2	0.0024029	0.0024029	0
RB1	0.0745511	0	0.0745511
RB1CC1	0.4983333	0	0.4983333
RBAK	0.006009	0	0
RBBP4	0.0498721	0	0.0498721
RBBP5	0.2614495	0	0.2614495
RBBP6	0.0995634	0	0.0995634
RBBP7	0.0209718	0.0209718	0
RBBP8	0.0542974	0	0
RBBP9	0.021356	0	0.021356
RBCK1	0.0621795	0.0621795	0
RBKS	0.1029372	0.1029372	0
RBL1	0.1088405	0	0.1088405
RBL2	0.0519307	0	0.0519307
RBM10	0.020514	0.020514	0
RBM11	0.0086604	0	0.0086604

RBM12	0.1425267	0	0.1425267
RBM12B	0.0648496	0	0
RBM14	0.0881959	0.0881959	0
RBM15	0.2390605	0	0.2390605
RBM15B	0.0271677	0	0
RBM16	0.0191305	0	0.0191305
RBM17	0.0311494	0	0.0311494
RBM18	0.1376764	0	0.1376764
RBM19	0.0294026	0.0294026	0
RBM20	0.018094	0	0.018094
RBM22	0.105277	0	0
RBM23	0.0966232	0	0.0966232
RBM24	0.0092129	0	0.0092129
RBM25	0.0670222	0	0
RBM26	0.1096149	0	0.1096149
RBM27	0.1393356	0	0
RBM28	0.0407558	0	0
RBM3	0.0463164	0	0.0463164
RBM33	0.063542	0.063542	0
RBM34	0.1024705	0	0
RBM38	0.0279785	0	0
RBM39	0.2852558	0.2852558	0
RBM4	0.1301999	0	0
RBM41	0.0340879	0.0340879	0
RBM42	0.0152966	0.0152966	0
RBM43	0.0277818	0	0.0277818
RBM44	0.0066203	0	0
RBM45	0.0434449	0	0
RBM46	0.0017384	0	0.0017384
RBM47	0.3619908	0.3619908	0
RBM4B	0.0575638	0	0
RBM5	0.0284548	0	0
RBM6	0.0243248	0.0243248	0
RBM7	0.1143232	0	0.1143232
RBM8A	0.3250595	0.3250595	0
RBM9	0.0574588	0	0.0574588
RBMS1	0.0145229	0	0.0145229
RBMS2	0.259149	0	0.259149
RBMS3	0.2437551	0	0.2437551
RBMX	0.0097563	0	0
RBMX2	0.0110296	0	0
RBMXL1	0.1556279	0	0.1556279
RBMXL2	0.0087786	0.0087786	0
RBMXL3	0.0030535	0	0
RBMY1A3P	1.53E-05	0	0

RBMY2EP	1.53E-05	0	0
RBMY2FP	1.53E-05	0	0
RBMY3AP	1.53E-05	0	0
RBP1	0.0127118	0	0.0127118
RBP2	0.0064723	0	0.0064723
RBP3	0.0029118	0.0029118	0
RBP4	0.0037699	0	0.0037699
RBP5	0.0042216	0	0.0042216
RBP7	0.0017682	0	0.0017682
RBPJ	0.0988927	0	0.0988927
RBPJL	0.0034762	0	0.0034762
RBPMS	0.007273	0	0
RBPMS2	0.0022566	0	0.0022566
RBX1	0.0229449	0	0
RC3H1	0.0409229	0	0.0409229
RC3H2	0.2056934	0	0.2056934
RCAN1	0.2172594	0	0.2172594
RCAN2	0.3000696	0	0.3000696
RCAN3	0.0110414	0	0
RCBTB1	0.0275601	0	0.0275601
RCBTB2	0.0220999	0	0.0220999
RCC1	0.1009026	0.1009026	0
RCC2	0.0416616	0	0
RCCD1	0.0131162	0.0131162	0
RCE1	0.0228105	0.0228105	0
RCHY1	0.0841347	0	0.0841347
RCL1	0.0469323	0	0.0469323
RCN1	0.1749653	0	0
RCN2	0.0541215	0	0.0541215
RCN3	0.0012362	0	0.0012362
RCOR1	0.0277691	0.0277691	0
RCOR2	0.0015365	0.0015365	0
RCOR3	0.0663675	0.0663675	0
RCSD1	0.0625122	0	0.0625122
RCVRN	0.0042917	0	0.0042917
RD3	0.0065497	0	0.0065497
RDH10	0.0903926	0	0.0903926
RDH11	0.1186063	0.1186063	0
RDH12	0.0914642	0.0914642	0
RDH13	0.024819	0.024819	0
RDH14	0.0575081	0.0575081	0
RDH16	0.0048058	0	0
RDH5	0.1116547	0.1116547	0
RDH8	0.0017035	0	0.0017035
RDM1	0.0088065	0.0088065	0

RDX	0.0850911	0	0.0850911
REC8	0.0082579	0	0
RECK	0.0301724	0	0.0301724
RECQL	0.1036432	0	0.1036432
RECQL4	0.0087348	0.0087348	0
RECQL5	0.047745	0.047745	0
REEP1	0.0091904	0	0
REEP2	0.0055345	0	0.0055345
REEP3	0.0845074	0	0
REEP4	0.0106012	0.0106012	0
REEP5	0.0975864	0.0975864	0
REEP6	0.0045827	0.0045827	0
REG1A	0.0042212	0	0
REG1B	0.0018903	0	0.0018903
REG1P	0.0045734	0	0.0045734
REG3A	0.003567	0	0.003567
REG3G	0.0039117	0	0.0039117
REG4	0.0199702	0.0199702	0
REL	0.1437239	0	0.1437239
RELA	0.0491725	0.0491725	0
RELB	0.0662593	0	0.0662593
RELL1	0.0377332	0	0.0377332
RELL2	0.0262792	0.0262792	0
RELN	0.0063557	0	0.0063557
RELT	0.0301629	0	0.0301629
REM1	0.0024602	0	0.0024602
REM2	0.0193396	0.0193396	0
REN	0.0857806	0	0
RENBP	0.0016092	0	0.0016092
REP15	0.0297359	0	0
REPIN1	0.0804786	0.0804786	0
REPS1	0.0362672	0	0.0362672
REPS2	0.0143649	0.0143649	0
RER1	0.0223124	0.0223124	0
RERE	0.1638708	0	0.1638708
RERG	0.0201808	0	0.0201808
RERGL	0.0036869	0	0.0036869
RESP18	0.0038936	0.0038936	0
REST	0.0098631	0	0.0098631
RET	0.0003158	0	0.0003158
RETN	0.003221	0	0.003221
RETNLB	0.0012477	0	0
RETSAT	0.0346502	0.0346502	0
REV1	0.09035	0	0
REV3L	0.4244621	0	0.4244621

REXO1	0.0094692	0.0094692	0
REXO1L1	3.61E-05	0	0
REXO2	0.0264314	0	0
REXO4	0.0375466	0.0375466	0
RFC1	0.0292438	0	0.0292438
RFC2	0.0961687	0.0961687	0
RFC3	0.0477039	0	0
RFC4	0.0463505	0.0463505	0
RFC5	0.0102896	0.0102896	0
RFESD	0.0730947	0	0.0730947
RFFL	0.3546254	0	0
RFK	0.0495315	0	0
RFNG	0.0006677	0.0006677	0
RFPL1	0.0045503	0	0
RFPL1S	0.0045495	0	0.0045495
RFPL2	0.0022285	0	0.0022285
RFPL3	0.0022267	0	0
RFPL3S	0.0029743	0	0
RFPL4A	0.0028531	0	0.0028531
RFPL4B	0.0093402	0	0.0093402
RFT1	0.0659229	0	0
RFTN1	0.2838059	0	0.2838059
RFTN2	0.0087273	0	0.0087273
RFWD2	0.1165864	0.1165864	0
RFWD3	0.0518789	0	0
RFX1	0.0763511	0	0
RFX2	0.046976	0	0.046976
RFX3	0.1632097	0	0.1632097
RFX4	0.0089604	0	0.0089604
RFX5	0.1395978	0	0.1395978
RFX6	0.006523	0	0.006523
RFX7	0.1632549	0	0.1632549
RFX8	0.0060591	0	0
RFXANK	0.0103998	0.0103998	0
RFXAP	0.0376639	0	0.0376639
RG9MTD1	0.0394676	0	0.0394676
RG9MTD2	0.0809403	0.0809403	0
RG9MTD3	0.0224211	0.0224211	0
RGAG1	0.0193834	0	0.0193834
RGAG4	0.0055027	0	0.0055027
RGL1	0.2644186	0	0.2644186
RGL3	0.0014856	0.0014856	0
RGL4	0.0023029	0	0.0023029
RGMA	0.0122866	0	0.0122866
RGMB	0.1041514	0	0.1041514

RGN	0.0028774	0	0
RGNEF	0.0241597	0.0241597	0
RGP1	0.0264026	0	0
RGPD3	3.28E-05	0	3.28E-05
RGPD4	0.0002728	0	0
RGR	0.0023473	0	0
RGS1	0.3601383	0	0.3601383
RGS10	0.0252201	0	0.0252201
RGS11	0.0003681	0	0
RGS12	0.0089609	0.0089609	0
RGS13	0.0469509	0	0.0469509
RGS14	0.049383	0.049383	0
RGS16	0.3059646	0	0.3059646
RGS17	0.0079914	0	0.0079914
RGS18	0.0163824	0	0.0163824
RGS19	0.002645	0	0.002645
RGS2	0.72789	0	0.72789
RGS20	0.0793952	0	0.0793952
RGS21	0.02086	0	0
RGS22	0.0072651	0	0.0072651
RGS3	0.1186242	0	0
RGS4	0.0553533	0	0.0553533
RGS5	0.0300549	0	0.0300549
RGS6	0.0166968	0	0
RGS7	0.014177	0	0
RGS7BP	0.0060963	0	0.0060963
RGS8	0.1074781	0	0.1074781
RGS9	0.0117517	0	0.0117517
RGS9BP	0.0058961	0.0058961	0
RGSL1	0.0234359	0	0
RHAG	0.0167391	0	0.0167391
RHBDD1	0.0501619	0	0.0501619
RHBDD2	0.0589285	0.0589285	0
RHBDD3	0.0332593	0.0332593	0
RHBDF1	0.0036174	0.0036174	0
RHBDF2	0.0262237	0	0
RHBDL1	0.0032036	0.0032036	0
RHBDL2	0.0043798	0.0043798	0
RHBDL3	0.0031379	0	0.0031379
RHBG	0.0071282	0.0071282	0
RHCE	0.0039451	0	0
RHCG	0.0550627	0	0
RHD	0.0001137	0	0
RHEB	0.0343357	0.0343357	0
RHEBL1	0.0488311	0	0.0488311

RHO	0.0015217	0	0
RHOA	0.0792044	0	0.0792044
RHOB	0.2923064	0	0
RHOBTB1	0.0244606	0	0.0244606
RHOBTB2	0.258934	0	0.258934
RHOBTB3	0.2039583	0	0.2039583
RHOC	0.1401051	0	0
RHOD	0.028972	0.028972	0
RHOF	0.3022873	0	0
RHOG	0.0858255	0	0.0858255
RHOH	0.2552309	0	0.2552309
RHOJ	0.0109804	0	0.0109804
RHOQ	0.1006537	0	0.1006537
RHOT1	0.054417	0	0.054417
RHOT2	0.0020109	0.0020109	0
RHOU	0.0289913	0	0.0289913
RHOV	0.835892	0.835892	0
RHOXF1	0.0033422	0	0
RHPN1	0.0063798	0.0063798	0
RHPN2	0.0064503	0.0064503	0
RIBC1	0.0344607	0.0344607	0
RIBC2	0.0047962	0.0047962	0
RIC3	0.0394445	0	0.0394445
RIC8A	0.0069358	0	0
RIC8B	0.084802	0	0.084802
RICH2	0.0035097	0	0
RICTOR	0.2723158	0	0
RIF1	0.0247372	0	0.0247372
RILP	0.0090012	0	0
RILPL1	0.0028331	0	0.0028331
RILPL2	0.0743898	0	0.0743898
RIMBP2	0.0014341	0	0.0014341
RIMBP3	1.62E-05	0	1.62E-05
RIMKLA	0.0056794	0.0056794	0
RIMKLB	0.0405223	0	0.0405223
RIMS1	0.0174088	0	0
RIMS2	0.0176401	0	0.0176401
RIMS3	0.0128998	0	0
RIMS4	0.0045731	0	0.0045731
RIN1	0.0718629	0.0718629	0
RIN2	0.0207708	0	0.0207708
RIN3	0.0157335	0	0.0157335
RINL	0.0052742	0.0052742	0
RINT1	0.0264848	0.0264848	0
RIOK1	0.0640226	0	0.0640226

RIOK2	0.1462483	0	0
RIOK3	0.0895471	0.0895471	0
RIPK1	0.0338802	0	0.0338802
RIPK2	0.1611534	0	0.1611534
RIPK3	0.0130013	0.0130013	0
RIPK4	0.0899898	0.0899898	0
RIPPLY1	0.0055581	0.0055581	0
RIPPLY2	0.0400794	0	0.0400794
RIT1	0.0976699	0	0.0976699
RIT2	0.0056918	0	0
RLBP1	0.0044208	0	0
RLF	0.0458168	0	0.0458168
RLIM	0.1664602	0	0.1664602
RLN1	0.0071816	0	0
RLN2	0.0047847	0	0
RLN3	0.0125752	0	0.0125752
RLTPR	0.0016069	0	0.0016069
RMI1	0.2125271	0.2125271	0
RMND1	0.0509393	0	0
RMND5A	0.0001094	0.0001094	0
RMND5B	0.0072631	0.0072631	0
RMRP	0.3510931	0	0.3510931
RMST	0.0116982	0	0
RNASE1	0.0062138	0	0.0062138
RNASE10	0.058013	0	0
RNASE11	0.0095665	0	0
RNASE12	0.0091718	0	0
RNASE13	0.0087584	0	0
RNASE2	0.007504	0	0.007504
RNASE3	0.0028179	0	0.0028179
RNASE4	0.2838846	0.2838846	0
RNASE6	0.0262965	0	0.0262965
RNASE7	0.0248534	0	0
RNASE8	0.0093441	0	0
RNASE9	0.0091974	0	0
RNASEH1	0.0338578	0	0
RNASEH2A	0.0152776	0.0152776	0
RNASEH2B	0.0457657	0	0.0457657
RNASEH2C	0.03345	0.03345	0
RNASEK	0.0600483	0	0
RNASEL	0.0879789	0	0.0879789
RNASEN	0.0965968	0.0965968	0
RNASET2	0.0286055	0.0286055	0
RND1	0.0155285	0	0
RND2	0.0046247	0	0

RND3	0.3226948	0	0.3226948
RNF10	0.1174559	0.1174559	0
RNF103	0.0527041	0.0527041	0
RNF11	0.0527003	0	0.0527003
RNF111	0.1132691	0	0.1132691
RNF112	0.0018011	0	0.0018011
RNF113A	0.0135272	0.0135272	0
RNF113B	0.0076271	0.0076271	0
RNF114	0.0560949	0.0560949	0
RNF115	0.0151145	0	0.0151145
RNF121	0.1282605	0	0
RNF122	0.0865037	0	0.0865037
RNF123	0.0049575	0.0049575	0
RNF125	0.0347269	0	0.0347269
RNF126	0.007535	0.007535	0
RNF126P1	0.0059117	0	0
RNF128	0.0089475	0.0089475	0
RNF13	0.1904026	0	0.1904026
RNF130	0.0171152	0	0.0171152
RNF133	0.006993	0	0
RNF135	0.0366951	0	0.0366951
RNF138	0.0477244	0	0.0477244
RNF138P1	0.0281526	0	0.0281526
RNF139	0.2251377	0	0.2251377
RNF14	0.0829051	0	0
RNF141	0.0495995	0.0495995	0
RNF144A	0.0161767	0	0.0161767
RNF144B	0.1683265	0	0.1683265
RNF145	0.5609395	0	0
RNF146	0.0416833	0	0.0416833
RNF148	0.0095231	0	0
RNF149	0.0685412	0	0
RNF150	0.0094726	0	0.0094726
RNF151	0.0362202	0	0
RNF152	0.0150597	0	0.0150597
RNF157	0.0284742	0	0.0284742
RNF160	0.0630211	0	0.0630211
RNF165	0.0201905	0	0.0201905
RNF166	0.0030908	0	0.0030908
RNF167	0.1841032	0	0
RNF168	0.1084227	0	0.1084227
RNF169	0.1444506	0	0.1444506
RNF17	0.0050336	0	0
RNF170	0.0486532	0	0
RNF175	0.004839	0	0.004839

RNF180	0.0200326	0	0.0200326
RNF181	0.0396617	0.0396617	0
RNF182	0.030937	0	0.030937
RNF183	0.0056763	0.0056763	0
RNF185	0.0155506	0	0.0155506
RNF186	0.0062776	0.0062776	0
RNF187	0.0470709	0.0470709	0
RNF19A	0.2779272	0	0.2779272
RNF19B	0.0572537	0	0.0572537
RNF2	0.0517785	0	0
RNF20	0.0445443	0	0
RNF207	0.0169755	0.0169755	0
RNF212	0.0084918	0	0.0084918
RNF213	1	0	1
RNF214	0.0171989	0	0
RNF215	0.002836	0	0
RNF216	0.0201845	0	0.0201845
RNF216L	0.0040045	0	0
RNF217	0.0310188	0	0.0310188
RNF219	0.0453003	0	0
RNF220	0.0124711	0	0
RNF222	0.0028641	0	0
RNF24	0.0579942	0	0.0579942
RNF25	0.0092388	0.0092388	0
RNF26	0.0065949	0.0065949	0
RNF31	0.057501	0.057501	0
RNF32	0.0240086	0.0240086	0
RNF34	0.0491103	0	0
RNF38	0.0920376	0	0.0920376
RNF4	0.0175871	0	0.0175871
RNF40	0.0149963	0.0149963	0
RNF41	0.0381328	0	0.0381328
RNF43	0.1263765	0.1263765	0
RNF44	0.0780852	0.0780852	0
RNF6	0.0482946	0	0
RNF7	0.0711647	0.0711647	0
RNF8	0.0234246	0	0
RNFT1	0.0210802	0	0.0210802
RNFT2	0.0270221	0.0270221	0
RNGTT	0.0289812	0	0.0289812
RNH1	0.0170232	0	0
RNLS	0.0287255	0.0287255	0
RNMT	0.0436134	0	0
RNMTL1	0.0227156	0	0
RNPC3	0.0953322	0	0

RNPEP	0.0552705	0.0552705	0
RNPEPL1	0.0118575	0.0118575	0
RNPS1	0.0463969	0.0463969	0
RNU11	0.3785594	0	0
RNU4ATAC	0.3471337	0	0
RNU6ATAC	0.1227349	0	0
ROBLD3	0.1159003	0.1159003	0
ROBO1	0.0648906	0	0.0648906
ROBO2	0.0079504	0	0.0079504
ROBO3	0.0018011	0	0.0018011
ROBO4	0.0242193	0	0.0242193
ROCK1	0.0553893	0	0.0553893
ROCK2	0.1724204	0	0.1724204
ROD1	0.1621584	0	0
ROGDI	0.0040069	0.0040069	0
ROM1	0.1159473	0	0
ROMO1	0.073884	0.073884	0
ROPN1	0.003575	0.003575	0
ROPN1B	0.0020948	0.0020948	0
ROPN1L	0.0505117	0	0.0505117
ROR1	0.007769	0	0.007769
ROR2	0.1532214	0	0.1532214
RORA	0.0808974	0	0.0808974
RORB	0.0069168	0	0.0069168
RORC	0.2937384	0.2937384	0
ROS1	0.0336935	0	0.0336935
RP1	0.0518182	0	0
RP1-177G6.2	0.0058944	0	0
RP1L1	0.0246408	0.0246408	0
RP2	0.0314805	0	0.0314805
RP9	0.0279251	0.0279251	0
RP9P	0.0128416	0.0128416	0
RPA1	0.0307817	0	0.0307817
RPA2	0.0282706	0	0.0282706
RPA3	0.0816031	0.0816031	0
RPA4	0.006194	0	0
RPAIN	0.1597618	0.1597618	0
RPAP1	0.0147057	0.0147057	0
RPAP2	0.0673897	0	0.0673897
RPAP3	0.1937404	0	0.1937404
RPE	0.0350718	0	0
RPE65	0.0146624	0	0.0146624
RPF1	0.0555518	0	0
RPF2	0.0455845	0	0.0455845
RPGR	0.0304653	0	0.0304653

RPGRIP1	0.0052026	0	0.0052026
RPGRIP1L	0.0435747	0	0.0435747
RPH3A	0.0034316	0	0.0034316
RPH3AL	0.0068505	0.0068505	0
RPIA	0.0382409	0.0382409	0
RPL10	0.0100689	0	0
RPL10A	0.0709217	0	0.0709217
RPL10L	0.0037926	0.0037926	0
RPL11	0.0482611	0	0
RPL12	0.1807364	0.1807364	0
RPL13	0.0364787	0.0364787	0
RPL13A	0.0946701	0	0
RPL13AP17	0.0099205	0	0
RPL13AP20	0.6636931	0.6636931	0
RPL13AP3	0.0147065	0	0
RPL13AP6	0.2232891	0	0
RPL13P5	0.0087867	0.0087867	0
RPL14	0.1065561	0.1065561	0
RPL15	0.0903043	0	0
RPL17	0.1367467	0	0.1367467
RPL18	0.1122825	0.1122825	0
RPL18A	0.2990357	0.2990357	0
RPL19	0.0901845	0.0901845	0
RPL19P12	0.0424497	0	0
RPL21	0.1218272	0.1218272	0
RPL21P44	0.0207146	0	0
RPL22	0.0398896	0	0
RPL22L1	0.1430263	0	0
RPL23	0.0969602	0	0
RPL23A	0.4458804	0.4458804	0
RPL23AP32	0.1206405	0	0
RPL23AP53	0.0120756	0	0.0120756
RPL23AP64	0.0194628	0	0
RPL23AP7	0.0001623	0	0.0001623
RPL23AP82	0.0002689	0	0.0002689
RPL23P8	0.0057293	0	0
RPL24	0.1574992	0.1574992	0
RPL26	0.0884504	0	0
RPL26L1	0.0827552	0.0827552	0
RPL27	0.2266767	0	0
RPL27A	0.1657209	0.1657209	0
RPL28	0.5475254	0.5475254	0
RPL29	0.0245602	0.0245602	0
RPL29P2	0.0799086	0	0
RPL3	0.0884682	0	0

RPL30	0.0504865	0.0504865	0
RPL31	0.0599324	0.0599324	0
RPL31P11	0.0292455	0	0.0292455
RPL32	0.077795	0	0
RPL32P3	0.0473763	0.0473763	0
RPL34	0.0760903	0.0760903	0
RPL35	0.6360445	0.6360445	0
RPL35A	0.2512772	0	0
RPL36	0.0194954	0.0194954	0
RPL36A	0.0348702	0.0348702	0
RPL36AL	0.1321888	0.1321888	0
RPL37	0.5296238	0.5296238	0
RPL37A	0.1292249	0.1292249	0
RPL38	0.0600492	0.0600492	0
RPL39	0.0228105	0	0
RPL39L	0.0379395	0	0
RPL3L	0.0056634	0	0
RPL4	0.2186804	0	0
RPL41	0.4122483	0.4122483	0
RPL5	0.0724362	0	0.0724362
RPL6	0.0845232	0	0
RPL7	0.1216115	0	0
RPL7A	0.049925	0	0
RPL7L1	0.0393723	0	0.0393723
RPL8	0.0469691	0.0469691	0
RPL9	0.1304406	0.1304406	0
RPLP0	0.2015914	0.2015914	0
RPLP0P2	0.0098252	0	0.0098252
RPLP1	0.1643728	0.1643728	0
RPLP2	0.0119337	0.0119337	0
RPN1	0.0202039	0.0202039	0
RPN2	0.0810482	0.0810482	0
RPP14	0.0276667	0	0
RPP25	0.0140909	0.0140909	0
RPP30	0.0907491	0	0.0907491
RPP38	0.0119605	0.0119605	0
RPP40	0.0155562	0	0.0155562
RPPH1	0.1087275	0	0
RPRD1A	0.0758784	0	0
RPRD1B	0.1007477	0	0
RPRD2	0.2488702	0	0
RPRM	0.0148109	0	0
RPRML	0.0020026	0	0
RPS10	0.0245566	0	0
RPS10P7	0.0621277	0.0621277	0

RPS11	0.1799735	0	0
RPS12	0.3504316	0	0.3504316
RPS13	0.0812548	0	0
RPS14	0.0940139	0.0940139	0
RPS15	0.0389259	0.0389259	0
RPS15A	0.0872621	0	0
RPS15AP10	0.025723	0	0
RPS16	0.5255169	0.5255169	0
RPS19	0.0790986	0.0790986	0
RPS19BP1	0.0423759	0.0423759	0
RPS2	0.1081761	0.1081761	0
RPS20	0.0822058	0	0
RPS21	0.0068578	0.0068578	0
RPS23	0.0608465	0	0
RPS24	0.3593225	0.3593225	0
RPS25	0.0550937	0	0
RPS26	0.1021109	0	0
RPS26P11	0.0032906	0	0
RPS27	0.3292509	0	0
RPS27A	0.399987	0	0
RPS27L	0.3205954	0.3205954	0
RPS28	0.0162368	0.0162368	0
RPS29	0.1467451	0	0
RPS2P32	0.0057708	0	0.0057708
RPS3	0.0861717	0.0861717	0
RPS3A	0.0692611	0	0.0692611
RPS4X	0.0667176	0	0
RPS4Y1	1.53E-05	0	0
RPS4Y2	1.53E-05	0	0
RPS5	0.0460523	0.0460523	0
RPS6	0.1762859	0	0
RPS6KA1	0.0345039	0	0
RPS6KA2	0.016276	0	0.016276
RPS6KA3	0.0782485	0	0.0782485
RPS6KA4	0.0103725	0.0103725	0
RPS6KA5	0.0580562	0	0.0580562
RPS6KA6	0.0025098	0	0
RPS6KB1	0.0407186	0	0.0407186
RPS6KB2	0.0301923	0.0301923	0
RPS6KC1	0.0974565	0	0.0974565
RPS6KL1	0.0057361	0.0057361	0
RPS7	0.1019555	0	0
RPS8	0.0429642	0	0
RPS9	0.3386427	0.3386427	0
RPSA	0.0870239	0	0

RPSAP52	0.0918069	0	0
RPSAP58	0.0020281	0.0020281	0
RPSAP9	0.0328477	0.0328477	0
RPTN	0.237825	0	0
RPTOR	0.021081	0	0
RPUSD1	0.0006645	0.0006645	0
RPUSD2	0.1139975	0	0
RPUSD3	0.0328824	0.0328824	0
RPUSD4	0.0188519	0	0
RQCD1	0.0864234	0	0.0864234
RRAD	0.0185695	0	0.0185695
RRAGA	0.0917493	0	0.0917493
RRAGB	0.0443461	0	0.0443461
RRAGC	0.3401937	0	0.3401937
RRAGD	0.0071455	0	0.0071455
RRAS	0.0301933	0.0301933	0
RRAS2	0.0381382	0	0
RRBP1	0.0164071	0.0164071	0
RREB1	0.0509029	0	0
RRH	0.0121113	0	0
RRM1	0.0646849	0.0646849	0
RRM2	0.2591068	0.2591068	0
RRM2B	0.659525	0	0.659525
RRN3	0.006147	0	0
RRN3P1	0.0004194	0	0
RRN3P2	8.33E-05	0	8.33E-05
RRN3P3	8.44E-05	0	0
RRP1	0.0909727	0.0909727	0
RRP12	0.0374739	0.0374739	0
RRP15	0.0863282	0	0
RRP1B	0.018588	0	0.018588
RRP7A	0.0012006	0.0012006	0
RRP7B	0.0014539	0.0014539	0
RRP8	0.0860926	0.0860926	0
RRP9	0.0103819	0.0103819	0
RRS1	0.0529452	0.0529452	0
RS1	0.0108064	0.0108064	0
RSAD1	0.0510172	0	0
RSAD2	0.0182717	0	0.0182717
RSBN1	0.151166	0	0.151166
RSBN1L	0.0588696	0	0
RSC1A1	0.0161517	0	0.0161517
RSF1	0.0644671	0	0.0644671
RSL1D1	0.03346	0	0
RSL24D1	0.1732325	0	0

RSPH1	0.0291571	0.0291571	0
RSPH3	0.0696687	0	0
RSPH4A	0.009849	0	0.009849
RSPH6A	0.0099406	0.0099406	0
RSPH9	0.0086512	0	0.0086512
RSPO1	0.001812	0	0.001812
RSPO2	0.015704	0	0.015704
RSPO3	0.0076436	0	0.0076436
RSPO4	0.0033419	0	0.0033419
RSPRY1	0.1059694	0	0.1059694
RSRC1	0.0968818	0	0
RSRC2	0.2255325	0	0
RSU1	0.0320082	0	0.0320082
RTBDN	0.0132175	0.0132175	0
RTCD1	0.0217118	0	0
RTDR1	0.0191558	0.0191558	0
RTEL1	0.0045367	0.0045367	0
RTF1	0.0824223	0	0.0824223
RTKN	0.0312284	0.0312284	0
RTKN2	0.0406776	0	0
RTL1	0.0010638	0	0.0010638
RTN1	0.0232372	0	0.0232372
RTN2	0.0103167	0.0103167	0
RTN3	0.2130247	0.2130247	0
RTN4	0.4579494	0	0
RTN4IP1	0.0595879	0	0
RTN4R	0.000334	0.000334	0
RTN4RL1	0.0038398	0	0.0038398
RTN4RL2	0.0080076	0	0.0080076
RTP1	0.0195998	0	0
RTP2	0.0098658	0.0098658	0
RTP3	0.0108172	0	0
RTP4	0.0210433	0	0
RTTN	0.076406	0	0.076406
RUFY1	0.1089713	0	0
RUFY2	0.0123214	0	0.0123214
RUFY3	0.2039058	0	0.2039058
RUFY4	0.0019893	0	0.0019893
RUNDC1	0.0180996	0.0180996	0
RUNDC2A	0.0338632	0	0.0338632
RUNDC2C	0.0027504	0	0.0027504
RUNDC3A	0.0052487	0	0.0052487
RUNDC3B	0.0043711	0	0.0043711
RUNX1	0.8860828	0	0.8860828
RUNX1T1	0.0169297	0	0.0169297

RUNX2	0.0465258	0	0.0465258
RUNX3	0.0429782	0	0.0429782
RUSC1	0.0286293	0.0286293	0
RUSC2	0.0586566	0	0.0586566
RUVBL1	0.0739521	0.0739521	0
RUVBL2	0.0244035	0.0244035	0
RWDD1	0.0566156	0	0.0566156
RWDD2A	0.145084	0	0.145084
RWDD2B	0.0478208	0	0
RWDD3	0.0194963	0	0
RWDD4A	0.0212066	0.0212066	0
RXFP1	0.0069074	0	0.0069074
RXFP2	0.0092572	0	0.0092572
RXFP3	0.0523936	0	0.0523936
RXFP4	0.189155	0.189155	0
RXRA	0.0039312	0	0
RXRG	0.0119165	0	0.0119165
RYBP	0.0761739	0	0.0761739
RYK	0.0346944	0	0
RYR1	0.0029425	0	0.0029425
RYR2	0.017936	0	0.017936
RYR3	0.0053495	0	0.0053495
S100A1	0.0433023	0	0
S100A10	1	1	0
S100A11	0.7420562	0.7420562	0
S100A12	0.3184263	0	0.3184263
S100A13	0.1006314	0.1006314	0
S100A14	0.1272648	0.1272648	0
S100A16	0.1211867	0.1211867	0
S100A2	0.5344537	0	0
S100A3	0.3787412	0	0.3787412
S100A4	0.1432779	0	0.1432779
S100A5	0.2698764	0.2698764	0
S100A6	0.2501937	0.2501937	0
S100A7	0.0122681	0.0122681	0
S100A7A	0.0097832	0	0
S100A7L2	0.0066923	0	0
S100A8	0.3980885	0	0.3980885
S100A9	0.4656921	0	0.4656921
S100B	0.0139898	0	0.0139898
S100G	0.0259945	0	0
S100P	0.0185078	0.0185078	0
S100PBP	0.2292007	0	0.2292007
S100Z	0.0055904	0	0.0055904
S1PR1	0.0888003	0	0.0888003

S1PR2	0.0299675	0	0.0299675
S1PR3	0.0099622	0	0.0099622
S1PR4	0.0160476	0	0.0160476
S1PR5	0.0056598	0	0.0056598
SAA1	0.3170575	0	0.3170575
SAA2	0.2749094	0	0.2749094
SAA3P	0.0077669	0	0.0077669
SAA4	0.0242558	0	0.0242558
SAAL1	0.0384237	0.0384237	0
SAC3D1	0.0075559	0.0075559	0
SACM1L	0.2156722	0	0.2156722
SACS	0.0177759	0	0.0177759
SAE1	0.0149652	0	0
SAFB	0.0395192	0.0395192	0
SAFB2	0.0388848	0.0388848	0
SAG	0.0031298	0	0
SAGE1	0.0008096	0	0.0008096
SALL1	0.0032283	0	0
SALL2	0.0049924	0	0.0049924
SALL3	0.0013965	0	0
SALL4	0.1025821	0	0
SAMD1	0.0189019	0.0189019	0
SAMD10	0.0382738	0.0382738	0
SAMD11	0.0005656	0	0.0005656
SAMD12	0.0644982	0.0644982	0
SAMD13	0.0404995	0.0404995	0
SAMD14	0.0039227	0	0.0039227
SAMD3	0.0069556	0	0.0069556
SAMD4A	0.0568729	0	0.0568729
SAMD4B	0.0201139	0	0
SAMD5	0.0036349	0	0
SAMD7	0.011865	0	0.011865
SAMD8	0.0401655	0	0.0401655
SAMD9	0.0534486	0	0
SAMD9L	0.0552942	0	0.0552942
SAMHD1	0.0409432	0	0.0409432
SAMM50	0.0124817	0	0
SAMSN1	0.6741183	0	0.6741183
SAP130	0.0075645	0	0
SAP18	0.0452859	0.0452859	0
SAP30	0.2536844	0	0.2536844
SAP30BP	0.0464889	0.0464889	0
SAP30L	0.0837254	0	0.0837254
SAPS1	0.0593897	0.0593897	0
SAPS2	0.0043489	0.0043489	0

SAPS3	0.0533115	0.0533115	0
SAR1A	0.0828735	0	0.0828735
SAR1B	0.0448008	0.0448008	0
SARDH	0.0038253	0	0.0038253
SARM1	0.116963	0	0
SARNP	0.131645	0.131645	0
SARS	0.1114813	0	0
SARS2	0.0206387	0.0206387	0
SART1	0.0442826	0.0442826	0
SART3	0.3590928	0	0
SASH1	0.0151655	0	0.0151655
SASH3	0.0168852	0	0.0168852
SASS6	0.1841943	0	0
SAT1	0.5620346	0	0.5620346
SAT2	0.0048213	0	0
SATB1	0.0128846	0	0.0128846
SATB2	0.0144466	0	0
SATL1	0.00795	0.00795	0
SAV1	0.3925728	0	0.3925728
SBDS	0.0317812	0.0317812	0
SBDSP1	0.0657097	0.0657097	0
SBF1	0.0017765	0.0017765	0
SBF1P1	0.0007051	0	0
SBF2	0.1414787	0	0.1414787
SBK1	0.0015191	0	0
SBK2	0.0010332	0	0.0010332
SBNO1	0.0132226	0	0.0132226
SBNO2	0.5027485	0	0
SBSN	0.0061096	0	0
SC4MOL	0.0848037	0.0848037	0
SC5DL	0.0506559	0	0
SC65	0.0057313	0	0
SCAF1	0.0324568	0.0324568	0
SCAI	0.0841564	0	0
SCAMP1	0.0828467	0	0
SCAMP2	0.0306721	0.0306721	0
SCAMP3	0.1192092	0.1192092	0
SCAMP4	0.1332787	0.1332787	0
SCAMP5	0.0081234	0	0
SCAND1	0.0976917	0.0976917	0
SCAND2	0.0411148	0.0411148	0
SCAP	0.0102629	0.0102629	0
SCAPER	0.029354	0	0.029354
SCARA3	0.004754	0	0.004754
SCARA5	0.0248826	0	0.0248826

SCARB1	0.0160276	0.0160276	0
SCARB2	0.037927	0	0.037927
SCARF1	0.0069433	0	0.0069433
SCARF2	0.0007558	0	0.0007558
SCARNA1	0.0286171	0	0
SCARNA10	0.0107248	0	0
SCARNA11	0.0046323	0	0
SCARNA12	0.1008968	0.1008968	0
SCARNA14	0.0212313	0	0
SCARNA15	0.0807632	0	0
SCARNA16	0.0481721	0.0481721	0
SCARNA17	0.0623915	0	0.0623915
SCARNA18	0.0425049	0	0
SCARNA2	0.1025898	0	0
SCARNA20	0.0403992	0	0.0403992
SCARNA21	0.0114785	0	0
SCARNA22	0.0018271	0.0018271	0
SCARNA23	0.0126089	0.0126089	0
SCARNA27	0.1269665	0	0
SCARNA3	0.0525614	0	0
SCARNA4	0.1203189	0	0.1203189
SCARNA5	0.0128536	0	0
SCARNA6	0.0041603	0.0041603	0
SCARNA7	0.0167958	0.0167958	0
SCARNA8	0.0198696	0	0
SCARNA9	0.0879418	0	0
SCARNA9L	0.0329531	0.0329531	0
SCCPDH	0.0142065	0.0142065	0
SCD	0.0692011	0.0692011	0
SCD5	0.0096792	0	0.0096792
SCEL	0.0386707	0	0
SCFD1	0.0934634	0.0934634	0
SCFD2	0.0586583	0	0.0586583
SCG2	0.0094168	0	0.0094168
SCG3	0.0321125	0	0.0321125
SCG5	0.0228523	0	0.0228523
SCGB1A1	0.0074323	0	0
SCGB1C1	0.0230406	0	0
SCGB1D1	0.0064004	0	0
SCGB1D2	0.0120876	0	0
SCGB1D4	0.0032569	0	0
SCGB2A1	0.0116584	0	0
SCGB2A2	0.0087304	0	0
SCGB3A1	0.003103	0	0
SCGB3A2	0.0116413	0	0.0116413

SCGBL	0.002471	0	0
SCGN	0.0250697	0	0.0250697
SCHIP1	0.0167244	0	0.0167244
SCIN	0.0258267	0.0258267	0
SCLT1	0.1281282	0	0.1281282
SCLY	0.0218007	0.0218007	0
SCMH1	0.339141	0.339141	0
SCML1	0.0377162	0	0
SCML2	0.0089698	0	0.0089698
SCML4	0.0128466	0	0.0128466
SCN10A	0.0029974	0	0.0029974
SCN11A	0.0049847	0	0.0049847
SCN1A	0.0094299	0	0
SCN1B	0.0011547	0	0.0011547
SCN2A	0.0157286	0	0.0157286
SCN2B	0.0058974	0	0.0058974
SCN3A	0.0107907	0	0.0107907
SCN3B	0.0062224	0	0.0062224
SCN4A	0.0020523	0	0.0020523
SCN4B	0.0481004	0	0.0481004
SCN5A	0.0032201	0	0
SCN7A	0.0050845	0	0.0050845
SCN8A	0.011503	0	0
SCN9A	0.0065506	0	0.0065506
SCNM1	0.153018	0	0
SCNN1A	0.1673605	0.1673605	0
SCNN1B	0.0073746	0	0.0073746
SCNN1D	0.0015561	0	0
SCNN1G	0.006438	0	0
SCO1	0.0591592	0	0
SCO2	0.025847	0	0
SCOC	0.0378549	0.0378549	0
SCP2	0.0331677	0.0331677	0
SCPEP1	0.0242878	0	0.0242878
SCRG1	0.0092522	0	0.0092522
SCRIB	0.0015183	0.0015183	0
SCRN1	0.0274023	0	0
SCRN2	0.0144275	0.0144275	0
SCRN3	0.0443484	0	0
SCRT2	0.0015633	0	0
SCT	0.0010786	0	0
SCTR	0.0029997	0	0
SCUBE1	0.0011582	0	0.0011582
SCUBE2	0.0074057	0	0.0074057
SCUBE3	0.0060007	0	0.0060007

SCYL1	0.0065036	0.0065036	0
SCYL2	0.0592343	0	0.0592343
SCYL3	0.8991314	0.8991314	0
SDAD1	0.0389319	0	0
SDC1	0.0811402	0.0811402	0
SDC2	0.0043829	0	0.0043829
SDC3	0.0033318	0	0.0033318
SDC4	0.1710988	0.1710988	0
SDC4P	0.0032328	0	0
SDCBP	0.1312468	0	0.1312468
SDCBP2	0.1224842	0.1224842	0
SDCCAG1	0.1111234	0	0.1111234
SDCCAG3	0.0171747	0.0171747	0
SDCCAG8	0.4523783	0	0
SDF2	0.0425326	0.0425326	0
SDF2L1	0.008308	0.008308	0
SDF4	0.0019486	0	0
SDHA	0.0177546	0.0177546	0
SDHAF1	0.0071214	0.0071214	0
SDHAF2	0.0589475	0	0
SDHAP1	0.000145	0.000145	0
SDHAP2	7.64E-05	7.64E-05	0
SDHAP3	0.0041695	0.0041695	0
SDHB	0.0597556	0	0
SDHC	0.0568794	0.0568794	0
SDHD	0.043834	0	0.043834
SDK1	0.0080852	0	0.0080852
SDK2	0.0017315	0	0.0017315
SDPR	0.0377806	0	0.0377806
SDR16C5	0.4525865	0.4525865	0
SDR39U1	0.063348	0.063348	0
SDR42E1	0.0085159	0.0085159	0
SDR9C7	0.0047483	0	0
SDS	0.0051183	0	0.0051183
SDSL	0.1001098	0	0
SEBOX	0.0328656	0.0328656	0
SEC1	0.0635995	0	0
SEC11A	0.0581411	0	0
SEC11C	0.0534843	0	0
SEC13	0.0157404	0.0157404	0
SEC14L1	0.0966181	0	0.0966181
SEC14L2	0.0080485	0	0
SEC14L3	0.0021813	0	0.0021813
SEC14L4	0.0016906	0	0
SEC14L5	0.0050885	0	0

SEC16A	0.0216891	0.0216891	0
SEC16B	0.021629	0.021629	0
SEC22A	0.0285955	0	0.0285955
SEC22B	1	0	1
SEC22C	0.0433908	0	0.0433908
SEC23A	0.0386948	0	0.0386948
SEC23B	0.0459189	0	0
SEC23IP	0.0603307	0	0.0603307
SEC24A	0.1113681	0	0.1113681
SEC24B	0.0750723	0	0.0750723
SEC24C	0.0377105	0.0377105	0
SEC24D	0.0411423	0	0.0411423
SEC31A	0.1776338	0	0.1776338
SEC31B	0.0233202	0	0
SEC61A1	0.0506094	0.0506094	0
SEC61A2	0.0211303	0	0
SEC61B	0.0639447	0.0639447	0
SEC61G	0.1802743	0.1802743	0
SEC62	0.1170772	0	0.1170772
SEC63	0.0745024	0	0.0745024
SECISBP2	0.2886439	0.2886439	0
SECISBP2L	0.1435787	0	0.1435787
SECTM1	0.029377	0	0.029377
SEH1L	0.0390824	0	0
SEL1L	0.0833575	0	0.0833575
SEL1L2	0.0091322	0	0.0091322
SEL1L3	0.0457851	0	0
SELE	0.0211246	0	0.0211246
SELENBP1	0.0171145	0.0171145	0
SELK	0.0925433	0	0
SELL	0.0665466	0	0.0665466
SELM	0.0042115	0	0.0042115
SELO	0.0044839	0.0044839	0
SELP	0.0141614	0	0.0141614
SELPLG	1	0	1
SELS	0.0401796	0.0401796	0
SELT	0.1498784	0	0
SELV	0.0024574	0	0
SEMA3A	0.0612863	0	0.0612863
SEMA3B	0.0136257	0.0136257	0
SEMA3C	0.0522136	0	0.0522136
SEMA3D	0.0075585	0	0.0075585
SEMA3E	0.0125338	0	0.0125338
SEMA3F	0.0074024	0	0
SEMA3G	0.001464	0	0.001464

SEMA4A	0.0616258	0	0.0616258
SEMA4B	0.8354841	0.8354841	0
SEMA4C	0.0062749	0	0.0062749
SEMA4D	0.0130843	0	0.0130843
SEMA4F	0.016761	0.016761	0
SEMA4G	0.0052409	0.0052409	0
SEMA5A	0.0119547	0	0.0119547
SEMA5B	0.0167943	0	0.0167943
SEMA6A	0.1426255	0	0.1426255
SEMA6B	0.0016232	0	0.0016232
SEMA6C	0.0113649	0	0.0113649
SEMA6D	0.0377439	0	0.0377439
SEMA7A	0.0209073	0	0.0209073
SEMG1	0.0239372	0	0
SEMG2	0.024841	0	0
SENP1	0.1009393	0	0.1009393
SENP2	0.0195184	0	0
SENP3	0.0724813	0	0
SENP5	0.0189041	0	0
SENP6	0.0508827	0	0.0508827
SENP7	0.1809981	0	0.1809981
SENP8	0.0570908	0	0
15-Sep	0.1370472	0	0.1370472
SEPHS1	0.0240543	0	0
SEPHS2	0.121712	0.121712	0
SEPN1	0.0094554	0	0.0094554
SEPP1	0.0180368	0	0.0180368
SEPSECS	0.0769249	0.0769249	0
1-Sep	0.0051435	0	0.0051435
10-Sep	0.217857	0	0
11-Sep	0.0340608	0	0.0340608
12-Sep	0.0012543	0.0012543	0
14-Sep	0.0025503	0.0025503	0
2-Sep	0.1090574	0	0.1090574
3-Sep	0.0023346	0	0.0023346
4-Sep	0.0192469	0	0.0192469
5-Sep	0.0005323	0	0.0005323
6-Sep	0.0246638	0	0.0246638
7-Sep	0.0960816	0	0.0960816
SEPT7L	0.0001035	0.0001035	0
SEPT7P2	0.0026687	0	0
8-Sep	0.0236134	0	0.0236134
9-Sep	1	0	0
SEPW1	0.0099857	0.0099857	0
SEPX1	0.0248745	0.0248745	0

SERAC1	0.0215099	0	0.0215099
SERBP1	0.1254206	0	0.1254206
SERF2	0.0780371	0.0780371	0
SERGEF	0.04379	0.04379	0
SERHL	0.0006945	0.0006945	0
SERHL2	0.0015614	0	0
SERINC1	0.0339411	0	0.0339411
SERINC2	0.0373021	0.0373021	0
SERINC3	0.0957618	0	0
SERINC4	0.1076252	0.1076252	0
SERINC5	0.0516723	0	0.0516723
SERP1	0.2295226	0	0
SERP2	0.0130936	0.0130936	0
SERPINA1	0.3010293	0.3010293	0
SERPINA10	0.0025797	0	0.0025797
SERPINA11	0.0047879	0	0
SERPINA12	0.0037105	0	0
SERPINA13	0.0028473	0	0
SERPINA3	0.016795	0	0.016795
SERPINA4	0.0028823	0	0
SERPINA5	0.0064381	0	0
SERPINA6	0.0123202	0	0
SERPINA7	0.0063118	0	0
SERPINA9	0.0066015	0	0.0066015
SERPINB1	0.7313887	0.7313887	0
SERPINB10	0.0395764	0	0.0395764
SERPINB11	0.0104489	0	0
SERPINB12	0.0129083	0.0129083	0
SERPINB13	0.067739	0	0
SERPINB2	0.2994043	0	0.2994043
SERPINB3	0.4884904	0	0
SERPINB4	0.4872475	0	0
SERPINB5	0.1482208	0.1482208	0
SERPINB6	0.2539653	0.2539653	0
SERPINB7	0.0408563	0	0.0408563
SERPINB8	0.1290921	0	0.1290921
SERPINB9	0.0203156	0	0.0203156
SERPINC1	0.0110185	0	0
SERPIND1	0.0122933	0	0
SERPINE1	0.0586123	0	0.0586123
SERPINE2	0.012767	0	0.012767
SERPINE3	0.010359	0	0
SERPINF1	0.0038806	0	0.0038806
SERPINF2	0.0077299	0	0.0077299
SERPING1	0.0044117	0	0.0044117

SERPINH1	0.0253606	0	0.0253606
SERPINI1	0.2070897	0	0.2070897
SERPINI2	0.0141985	0	0.0141985
SERTAD1	0.15294	0	0
SERTAD2	0.1274698	0	0.1274698
SERTAD3	0.054461	0	0
SERTAD4	0.0798632	0	0
SESN1	0.1130428	0	0.1130428
SESN2	0.0550723	0	0
SESN3	0.0428639	0	0.0428639
SESTD1	0.3111452	0	0
SET	0.1641847	0	0
SETBP1	0.0240275	0	0.0240275
SETD1A	0.0305839	0.0305839	0
SETD1B	0.0553302	0	0
SETD2	0.0496279	0	0.0496279
SETD3	0.054811	0.054811	0
SETD4	0.050381	0.050381	0
SETD5	0.2836678	0	0
SETD6	0.0305929	0.0305929	0
SETD7	0.0199312	0	0.0199312
SETD8	0.0964625	0.0964625	0
SETDB1	0.1421258	0.1421258	0
SETDB2	0.1006198	0	0.1006198
SETMAR	0.0469826	0	0.0469826
SETX	0.0425791	0	0.0425791
SEZ6	0.003241	0	0.003241
SEZ6L	0.0025148	0	0.0025148
SEZ6L2	0.0032002	0.0032002	0
SF1	0.1455741	0	0
SF3A1	0.0092748	0	0.0092748
SF3A2	0.0135765	0.0135765	0
SF3A3	0.0697459	0	0.0697459
SF3B1	0.0524957	0	0.0524957
SF3B14	0.4611329	0.4611329	0
SF3B2	0.0247201	0.0247201	0
SF3B3	0.0743463	0	0
SF3B4	0.189236	0.189236	0
SF3B5	0.0571001	0	0
SF4	0.0157506	0.0157506	0
SFI1	0.0565513	0.0565513	0
SFMBT1	0.0175833	0	0
SFMBT2	0.0253139	0	0.0253139
SFN	0.4508308	0.4508308	0
SFPQ	0.0909411	0	0.0909411

SFRP1	0.0075214	0	0.0075214
SFRP2	0.0074495	0	0.0074495
SFRP4	0.0232705	0	0.0232705
SFRP5	0.001335	0	0
SFRS1	0.2577677	0	0
SFRS11	0.057166	0	0
SFRS12	0.1343867	0	0
SFRS12IP1	0.1955126	0	0
SFRS13B	0.0090731	0	0.0090731
SFRS14	0.0153306	0	0
SFRS15	0.0307358	0	0
SFRS16	0.0480421	0.0480421	0
SFRS18	0.1990377	0	0
SFRS2	0.1977768	0.1977768	0
SFRS2B	0.298869	0	0
SFRS2IP	1	0	1
SFRS3	0.1815675	0	0
SFRS4	0.1035185	0	0.1035185
SFRS5	0.0897471	0	0
SFRS6	0.0854126	0.0854126	0
SFRS7	0.162856	0	0.162856
SFRS8	0.0258217	0.0258217	0
SFRS9	0.026092	0.026092	0
SFT2D1	0.0635203	0.0635203	0
SFT2D2	0.1104599	0	0.1104599
SFT2D3	0.0449271	0.0449271	0
SFTA1P	0.0589671	0	0.0589671
SFTA3	0.0009457	0	0
SFTPA1	0.005688	0	0.005688
SFTPB	0.0063734	0	0.0063734
SFTPC	0.0066296	0	0
SFTPD	0.0064207	0.0064207	0
SFXN1	0.0189609	0.0189609	0
SFXN2	0.0441276	0.0441276	0
SFXN3	0.0049201	0	0
SFXN4	0.0295404	0.0295404	0
SFXN5	0.170099	0	0.170099
SGCA	0.0094734	0	0.0094734
SGCB	0.0114172	0	0.0114172
SGCD	0.0108968	0	0.0108968
SGCE	0.0429511	0	0.0429511
SGCG	0.0031805	0	0.0031805
SGCZ	0.0028178	0	0.0028178
SGEF	0.0074869	0	0.0074869
SGIP1	0.0197888	0	0.0197888

SGK1	0.1959382	0	0.1959382
SGK196	0.032761	0	0.032761
SGK2	0.004358	0.004358	0
SGK223	0.0470922	0	0.0470922
SGK269	0.0221701	0	0.0221701
SGK3	0.0392021	0	0.0392021
SGK494	0.0068797	0.0068797	0
SGMS1	0.0915506	0	0.0915506
SGMS2	0.3807903	0	0.3807903
SGOL1	0.0875998	0.0875998	0
SGOL2	0.0384671	0	0
SGPL1	0.0211466	0	0
SGPP1	0.0784293	0	0.0784293
SGPP2	0.1928135	0.1928135	0
SGSH	0.0025319	0	0
SGSM1	0.0021564	0	0
SGSM2	0.0189394	0	0
SGSM3	0.0188071	0.0188071	0
SGTA	0.0364043	0.0364043	0
SGTB	0.0413726	0	0.0413726
SH2B1	0.0283144	0.0283144	0
SH2B2	0.0089033	0	0
SH2B3	0.0544178	0	0.0544178
SH2D1A	0.0039799	0	0.0039799
SH2D1B	0.060766	0	0.060766
SH2D2A	0.0887509	0	0.0887509
SH2D3A	0.0355387	0.0355387	0
SH2D3C	0.2269089	0	0.2269089
SH2D4A	0.0343941	0.0343941	0
SH2D4B	0.0142364	0	0.0142364
SH2D5	0.0301707	0.0301707	0
SH2D6	0.0201328	0.0201328	0
SH2D7	0.0114413	0	0
SH3BGR	0.0401116	0	0.0401116
SH3BGRL	0.0293687	0	0.0293687
SH3BGRL2	0.0132354	0.0132354	0
SH3BGRL3	0.0388051	0	0
SH3BP1	0.0057785	0	0
SH3BP2	0.029353	0	0
SH3BP4	0.0123707	0	0
SH3BP5	0.0416461	0	0.0416461
SH3BP5L	0.176453	0.176453	0
SH3D19	0.0230241	0	0.0230241
SH3D20	0.0081562	0.0081562	0
SH3GL1	0.0382702	0	0

SH3GL2	0.0072275	0	0.0072275
SH3GL3	0.0028642	0	0
SH3GLB1	0.1157685	0	0.1157685
SH3GLB2	0.0185782	0.0185782	0
SH3KBP1	0.0266952	0	0.0266952
SH3PXD2A	0.0092649	0	0.0092649
SH3PXD2B	0.0263827	0	0.0263827
SH3RF1	0.0358037	0.0358037	0
SH3RF2	0.2462893	0.2462893	0
SH3RF3	0.0207466	0	0.0207466
SH3TC1	0.0311147	0	0
SH3TC2	0.0515132	0.0515132	0
SH3YL1	0.0229525	0.0229525	0
SHANK1	0.0009028	0	0.0009028
SHANK2	0.0023501	0.0023501	0
SHANK3	0.0005859	0	0.0005859
SHARPIN	0.0109744	0.0109744	0
SHB	0.0172572	0.0172572	0
SHBG	0.0197618	0	0
SHC1	1	0	1
SHC2	0.0003301	0.0003301	0
SHC3	0.0102668	0	0
SHC4	0.0328267	0	0.0328267
SHCBP1	0.0163649	0	0
SHD	0.000913	0	0.000913
SHE	0.0150229	0	0.0150229
SHF	0.3856509	0	0
SHFM1	0.0522177	0.0522177	0
SHH	0.0013128	0.0013128	0
SHISA2	0.0031903	0	0.0031903
SHISA3	0.004825	0	0.004825
SHISA4	0.0407299	0	0.0407299
SHISA5	0.0179337	0	0
SHISA6	0.0008875	0	0.0008875
SHISA7	0.0023367	0	0.0023367
SHISA9	0.0037244	0	0.0037244
SHKBP1	0.0360618	0	0
SHMT1	0.0142912	0	0
SHMT2	0.0254835	0	0
SHOC2	0.2866535	0	0.2866535
SHOX2	0.0539795	0	0.0539795
SHPK	0.0073566	0	0
SHPRH	0.0296466	0	0.0296466
SHQ1	0.0384656	0	0.0384656
SHROOM1	0.0215865	0.0215865	0

SHROOM2	0.0023833	0	0
SHROOM3	0.0231753	0.0231753	0
SHROOM4	0.0033364	0	0.0033364
SI	0.0132926	0	0.0132926
SIAE	0.1925466	0.1925466	0
SIAH1	0.2000308	0	0
SIAH2	0.1141979	0	0
SIAH3	0.004776	0	0.004776
SIDT1	0.0798686	0	0.0798686
SIDT2	0.020961	0	0.020961
SIGIRR	0.0009104	0.0009104	0
SIGLEC1	0.0013527	0	0.0013527
SIGLEC10	0.0047273	0	0.0047273
SIGLEC11	0.0011753	0	0.0011753
SIGLEC12	0.0022093	0	0.0022093
SIGLEC14	0.0026753	0	0.0026753
SIGLEC15	0.0096012	0	0
SIGLEC16	0.0006619	0	0.0006619
SIGLEC5	0.0010703	0	0.0010703
SIGLEC6	0.0048133	0	0.0048133
SIGLEC7	0.0022599	0	0.0022599
SIGLEC8	0.0013741	0	0.0013741
SIGLEC9	0.001436	0	0.001436
SIGLECP3	0.0102701	0	0.0102701
SIGMAR1	0.030128	0.030128	0
SIK1	0.0436782	0	0.0436782
SIK2	0.0518414	0	0.0518414
SIK3	0.0985609	0	0.0985609
SIKE1	0.0350951	0	0.0350951
SIL1	0.0413321	0.0413321	0
SILV	0.1308834	0.1308834	0
SIM1	0.0084282	0	0
SIM2	0.0019455	0.0019455	0
SIN3A	0.1797841	0	0.1797841
SIN3B	0.0396756	0	0
SIP1	0.0757874	0	0
SIPA1	0.0115054	0	0.0115054
SIPA1L1	0.0517826	0	0
SIPA1L2	0.0637844	0	0.0637844
SIPA1L3	0.0415948	0.0415948	0
SIRPA	0.0074716	0	0.0074716
SIRPB1	0.024911	0	0.024911
SIRPB2	0.0315056	0	0.0315056
SIRPD	0.0079322	0	0.0079322
SIRPG	0.0133021	0	0.0133021

SIRT1	0.028613	0	0.028613
SIRT2	0.0193188	0	0.0193188
SIRT3	0.015714	0.015714	0
SIRT4	0.0618861	0	0
SIRT5	0.0325424	0.0325424	0
SIRT6	0.0091702	0.0091702	0
SIT1	0.1923042	0	0.1923042
SIVA1	0.0060519	0.0060519	0
SIX1	0.0049601	0	0
SIX2	0.0059654	0	0
SIX3	0.0009804	0	0
SIX4	0.0241144	0	0
SIX5	0.0114556	0.0114556	0
SIX6	0.0040205	0	0
SKA1	0.0242245	0.0242245	0
SKA2	0.2628195	0	0
SKA3	0.0160292	0.0160292	0
SKAP1	0.0263977	0.0263977	0
SKAP2	0.5881944	0	0.5881944
SKI	0.0058913	0	0.0058913
SKIL	0.1812469	0	0.1812469
SKINTL	0.0109344	0.0109344	0
SKIV2L2	0.1021331	0	0
SKP1	0.0676446	0	0
SKP2	0.1265319	0	0.1265319
SLA	0.1342843	0	0.1342843
SLA2	0.0056165	0	0.0056165
SLAIN1	0.0308423	0.0308423	0
SLAIN2	0.0735029	0	0.0735029
SLAMF1	0.1810824	0	0.1810824
SLAMF6	0.0367155	0	0.0367155
SLAMF7	0.1737872	0	0.1737872
SLAMF8	0.1136488	0	0.1136488
SLAMF9	0.0273028	0	0.0273028
SLBP	0.0174257	0.0174257	0
SLC10A1	0.0092613	0.0092613	0
SLC10A2	0.0178724	0	0.0178724
SLC10A3	0.0043191	0.0043191	0
SLC10A4	0.0050259	0	0
SLC10A5	0.0249329	0.0249329	0
SLC10A6	0.2543223	0	0.2543223
SLC10A7	0.0301377	0	0
SLC11A1	0.0038295	0	0.0038295
SLC11A2	0.4781556	0	0
SLC12A1	0.0136777	0	0.0136777

SLC12A2	0.1545649	0	0
SLC12A3	0.0198231	0	0.0198231
SLC12A4	0.0745421	0	0.0745421
SLC12A5	0.0077726	0	0.0077726
SLC12A6	0.158259	0	0.158259
SLC12A7	0.0081169	0.0081169	0
SLC12A8	0.0101986	0	0.0101986
SLC12A9	0.0033996	0.0033996	0
SLC13A1	0.0312047	0	0.0312047
SLC13A2	0.0151435	0	0
SLC13A3	0.0337118	0	0.0337118
SLC13A4	0.0045612	0	0
SLC13A5	0.0061014	0	0
SLC14A1	0.0087542	0	0.0087542
SLC14A2	0.0055117	0	0.0055117
SLC15A1	0.0038415	0	0
SLC15A2	0.0073526	0	0.0073526
SLC15A3	0.0141196	0	0.0141196
SLC15A4	0.1296003	0	0.1296003
SLC16A1	0.164301	0	0
SLC16A10	0.014997	0	0.014997
SLC16A11	0.0348497	0.0348497	0
SLC16A12	0.0065476	0	0.0065476
SLC16A13	0.0705508	0.0705508	0
SLC16A14	0.008335	0	0.008335
SLC16A2	0.0037487	0	0.0037487
SLC16A3	0.0061064	0.0061064	0
SLC16A4	0.0241467	0	0.0241467
SLC16A5	0.1003453	0.1003453	0
SLC16A6	0.0477635	0	0.0477635
SLC16A7	0.0232499	0	0.0232499
SLC16A8	0.0007339	0.0007339	0
SLC16A9	0.0059601	0	0
SLC17A1	0.0126436	0	0
SLC17A2	0.0120409	0	0
SLC17A3	0.0208225	0	0.0208225
SLC17A4	0.0101317	0	0
SLC17A5	0.0584399	0	0
SLC17A6	0.0139244	0	0.0139244
SLC17A7	0.0036035	0	0.0036035
SLC17A8	0.0075129	0	0
SLC17A9	0.0054769	0.0054769	0
SLC18A1	0.0427195	0	0.0427195
SLC18A2	0.0030253	0	0.0030253
SLC18A3	0.0048596	0	0.0048596

SLC19A1	0.0103062	0.0103062	0
SLC19A2	0.3733723	0	0.3733723
SLC19A3	0.0039272	0	0.0039272
SLC1A1	0.0304069	0	0
SLC1A2	0.0117128	0	0.0117128
SLC1A3	0.2457325	0	0.2457325
SLC1A4	0.0992774	0	0.0992774
SLC1A5	0.1097806	0.1097806	0
SLC1A6	0.0036711	0	0
SLC1A7	0.0159712	0	0.0159712
SLC20A1	0.1095629	0	0.1095629
SLC20A2	0.1718415	0	0.1718415
SLC22A1	0.0010912	0	0
SLC22A10	0.0121611	0	0
SLC22A11	0.003451	0	0.003451
SLC22A12	0.0020999	0	0.0020999
SLC22A13	0.011033	0.011033	0
SLC22A14	0.003162	0.003162	0
SLC22A15	0.065127	0	0
SLC22A16	0.0077463	0	0.0077463
SLC22A17	0.0022335	0	0.0022335
SLC22A18	0.0211893	0.0211893	0
SLC22A18AS	0.0121547	0.0121547	0
SLC22A2	0.0090028	0	0.0090028
SLC22A20	0.0019887	0.0019887	0
SLC22A23	0.0223344	0.0223344	0
SLC22A24	0.0082715	0	0.0082715
SLC22A25	0.0064442	0	0
SLC22A3	0.0147652	0	0
SLC22A4	0.4138012	0	0.4138012
SLC22A5	0.4934498	0.4934498	0
SLC22A6	0.0031686	0	0
SLC22A7	0.0032967	0	0
SLC22A8	0.0049935	0	0
SLC22A9	0.005585	0	0
SLC23A1	0.0835094	0	0
SLC23A2	0.0163837	0	0.0163837
SLC23A3	0.0109832	0.0109832	0
SLC24A1	0.0134385	0	0.0134385
SLC24A2	0.0060371	0	0.0060371
SLC24A3	0.0046222	0	0.0046222
SLC24A4	0.0048296	0	0.0048296
SLC24A5	0.0127639	0	0
SLC24A6	0.0593761	0	0.0593761
SLC25A1	0.0040521	0.0040521	0

SLC25A10	0.0057323	0.0057323	0
SLC25A11	0.1714882	0	0
SLC25A12	0.049233	0	0.049233
SLC25A13	0.0569237	0.0569237	0
SLC25A14	0.0110916	0	0
SLC25A15	0.0084929	0.0084929	0
SLC25A16	0.0036158	0.0036158	0
SLC25A17	0.0293092	0	0
SLC25A18	0.0051352	0	0.0051352
SLC25A19	0.0855739	0	0.0855739
SLC25A2	0.005742	0.005742	0
SLC25A20	0.0082697	0	0
SLC25A21	0.0200101	0	0
SLC25A22	0.0013217	0.0013217	0
SLC25A23	0.0142778	0.0142778	0
SLC25A24	0.0718211	0	0
SLC25A25	0.3010573	0.3010573	0
SLC25A26	0.0261706	0.0261706	0
SLC25A27	0.0832478	0	0
SLC25A28	0.2324016	0	0
SLC25A29	0.0056654	0.0056654	0
SLC25A3	0.0757302	0.0757302	0
SLC25A30	0.0118436	0	0.0118436
SLC25A31	0.0083259	0	0
SLC25A32	0.2210466	0	0.2210466
SLC25A33	0.0057098	0	0
SLC25A34	0.0009116	0	0.0009116
SLC25A35	0.0128841	0.0128841	0
SLC25A36	0.0472381	0	0
SLC25A37	0.0574802	0.0574802	0
SLC25A38	0.0394859	0.0394859	0
SLC25A39	0.0235971	0.0235971	0
SLC25A4	0.0173749	0.0173749	0
SLC25A40	0.126055	0	0
SLC25A41	0.0028735	0.0028735	0
SLC25A42	0.0080616	0	0.0080616
SLC25A43	0.0074281	0	0
SLC25A44	1	0	0
SLC25A45	0.1036621	0	0.1036621
SLC25A46	0.0484471	0	0.0484471
SLC25A5	0.0175089	0.0175089	0
SLC26A1	0.0008592	0.0008592	0
SLC26A10	0.0020165	0	0.0020165
SLC26A11	0.0028114	0	0.0028114
SLC26A2	0.0981396	0	0.0981396

SLC26A3	0.0084185	0	0
SLC26A4	0.1480483	0	0.1480483
SLC26A5	0.0025136	0.0025136	0
SLC26A6	0.0030833	0.0030833	0
SLC26A7	0.0124365	0	0.0124365
SLC26A8	0.0931712	0.0931712	0
SLC26A9	0.292772	0.292772	0
SLC27A1	0.0156585	0.0156585	0
SLC27A2	0.0357731	0.0357731	0
SLC27A3	0.0344931	0	0
SLC27A4	0.0305624	0.0305624	0
SLC27A5	0.0122114	0.0122114	0
SLC27A6	0.0015226	0	0.0015226
SLC28A1	0.0037301	0	0
SLC28A2	0.0108591	0.0108591	0
SLC28A3	0.0154524	0	0
SLC29A1	0.0198753	0	0.0198753
SLC29A2	0.0159631	0.0159631	0
SLC29A3	0.009274	0	0.009274
SLC29A4	0.0006967	0	0
SLC2A1	0.0731972	0.0731972	0
SLC2A10	0.017351	0	0
SLC2A11	0.0046604	0	0
SLC2A12	0.0396067	0	0
SLC2A13	0.0558658	0	0.0558658
SLC2A14	0.0049385	0	0.0049385
SLC2A2	0.0180206	0	0.0180206
SLC2A3	0.1050051	0	0.1050051
SLC2A4	0.024124	0	0.024124
SLC2A4RG	0.0037968	0.0037968	0
SLC2A5	0.0915359	0	0.0915359
SLC2A6	0.0051718	0	0
SLC2A7	0.0024226	0	0
SLC2A8	0.0631989	0.0631989	0
SLC2A9	0.0436459	0	0.0436459
SLC30A1	0.3671102	0	0
SLC30A10	0.0192329	0	0
SLC30A2	0.001675	0	0.001675
SLC30A3	0.0046059	0	0.0046059
SLC30A4	0.0096575	0	0.0096575
SLC30A5	0.037503	0	0
SLC30A6	0.2685098	0	0.2685098
SLC30A7	0.2208902	0	0.2208902
SLC30A8	0.029995	0	0.029995
SLC30A9	0.0290854	0.0290854	0

SLC31A1	0.0932396	0	0.0932396
SLC31A2	0.0175507	0	0.0175507
SLC32A1	0.0025327	0	0.0025327
SLC33A1	0.1485901	0	0
SLC34A1	0.013986	0	0
SLC34A2	0.0046134	0	0
SLC34A3	0.0121888	0.0121888	0
SLC35A1	0.0428645	0.0428645	0
SLC35A2	0.0532553	0.0532553	0
SLC35A3	0.0280561	0.0280561	0
SLC35A4	0.1322304	0	0
SLC35A5	0.0756922	0	0.0756922
SLC35B1	0.0278039	0.0278039	0
SLC35B2	0.1585968	0.1585968	0
SLC35B3	0.0412302	0	0
SLC35B4	0.0684515	0	0.0684515
SLC35C1	0.0727824	0.0727824	0
SLC35C2	0.0307761	0.0307761	0
SLC35D1	0.0473552	0	0.0473552
SLC35D2	0.0160024	0.0160024	0
SLC35D3	0.0042883	0	0
SLC35E1	0.0244652	0	0
SLC35E2	0.001563	0	0.001563
SLC35E3	0.0373223	0	0
SLC35E4	0.0029607	0.0029607	0
SLC35F1	0.003964	0	0.003964
SLC35F2	0.0930039	0.0930039	0
SLC35F3	0.0761316	0	0
SLC35F4	0.0070386	0	0
SLC35F5	0.0417051	0	0
SLC36A1	0.0385592	0	0.0385592
SLC36A2	0.0121818	0	0.0121818
SLC36A3	0.0123895	0	0.0123895
SLC36A4	0.0901862	0	0.0901862
SLC37A1	0.0265604	0.0265604	0
SLC37A2	0.0239283	0	0.0239283
SLC37A3	0.0204171	0	0
SLC37A4	0.0156071	0.0156071	0
SLC38A1	0.2389609	0	0
SLC38A10	0.0105439	0.0105439	0
SLC38A11	0.0053454	0	0.0053454
SLC38A2	0.4300959	0	0.4300959
SLC38A3	0.000759	0	0
SLC38A4	0.0219814	0	0.0219814
SLC38A5	0.0131293	0	0

SLC38A6	0.0832635	0	0.0832635
SLC38A7	0.0076667	0	0.0076667
SLC38A8	0.0015675	0	0
SLC38A9	0.0274194	0	0.0274194
SLC39A1	0.5731466	0.5731466	0
SLC39A10	0.2754558	0	0.2754558
SLC39A11	0.0103271	0.0103271	0
SLC39A12	0.0054671	0	0.0054671
SLC39A13	0.0446483	0	0
SLC39A14	0.1143188	0	0
SLC39A2	0.0395242	0	0.0395242
SLC39A3	0.0067957	0.0067957	0
SLC39A4	0.0116613	0.0116613	0
SLC39A5	0.0144236	0.0144236	0
SLC39A6	0.0885427	0	0.0885427
SLC39A8	0.1141056	0	0.1141056
SLC39A9	0.1403659	0.1403659	0
SLC3A1	0.0050284	0	0.0050284
SLC3A2	0.2218022	0.2218022	0
SLC40A1	0.0068056	0	0
SLC41A1	0.1005973	0	0.1005973
SLC41A2	0.0470121	0	0
SLC41A3	0.0205088	0.0205088	0
SLC43A1	0.0083901	0	0
SLC43A2	0.0052552	0	0.0052552
SLC43A3	0.1785562	0	0.1785562
SLC44A1	0.0461576	0	0
SLC44A2	0.0123601	0	0
SLC44A3	0.018366	0.018366	0
SLC44A5	0.0112184	0	0
SLC45A1	0.0015904	0	0.0015904
SLC45A2	0.0286475	0	0
SLC45A3	0.0157016	0.0157016	0
SLC45A4	0.0853974	0.0853974	0
SLC46A1	0.0134	0	0
SLC46A2	0.0060727	0	0.0060727
SLC46A3	0.2429762	0	0.2429762
SLC47A1	0.003192	0	0.003192
SLC47A2	0.0557038	0.0557038	0
SLC48A1	0.0550673	0.0550673	0
SLC4A1	0.0019024	0	0.0019024
SLC4A10	0.0086372	0	0.0086372
SLC4A11	0.0011673	0.0011673	0
SLC4A1AP	0.0514307	0	0
SLC4A2	0.0147059	0.0147059	0

SLC4A3	0.0007785	0.0007785	0
SLC4A4	0.007333	0	0
SLC4A5	0.0141855	0.0141855	0
SLC4A7	0.0194679	0	0.0194679
SLC4A8	0.0191993	0	0.0191993
SLC4A9	0.014989	0.014989	0
SLC5A1	0.0126652	0	0
SLC5A10	0.0030601	0.0030601	0
SLC5A11	0.0107308	0	0
SLC5A12	0.0258891	0	0
SLC5A2	0.0020295	0	0
SLC5A3	0.0509211	0	0
SLC5A4	0.0025078	0	0.0025078
SLC5A5	0.1182021	0	0.1182021
SLC5A6	0.0573792	0.0573792	0
SLC5A7	0.0052301	0	0.0052301
SLC5A8	0.0063892	0	0.0063892
SLC5A9	0.0029325	0	0.0029325
SLC6A1	0.0039418	0	0.0039418
SLC6A10P	2.14E-05	2.14E-05	0
SLC6A11	0.0038269	0	0
SLC6A12	0.0022773	0	0.0022773
SLC6A13	0.0066642	0	0.0066642
SLC6A14	0.0097971	0	0
SLC6A15	0.0561781	0	0.0561781
SLC6A16	0.0268842	0	0.0268842
SLC6A17	0.0077494	0	0.0077494
SLC6A18	0.0017324	0.0017324	0
SLC6A19	0.0016025	0	0
SLC6A2	0.0102774	0	0
SLC6A20	0.0105366	0	0
SLC6A3	0.0077273	0	0.0077273
SLC6A4	0.0053294	0	0.0053294
SLC6A5	0.0037776	0	0
SLC6A6	0.0162963	0	0.0162963
SLC6A7	0.0032672	0	0
SLC6A8	0.000634	0.000634	0
SLC6A9	0.0164966	0	0
SLC7A1	0.0266616	0.0266616	0
SLC7A10	0.0032825	0	0.0032825
SLC7A11	0.4150831	0.4150831	0
SLC7A13	0.0078979	0	0
SLC7A14	0.012011	0	0.012011
SLC7A2	0.29434	0	0.29434
SLC7A3	0.0018056	0	0.0018056

SLC7A4	0.0043199	0.0043199	0
SLC7A5	0.0685835	0	0
SLC7A5P1	0.0002313	0	0
SLC7A5P2	0.0130858	0.0130858	0
SLC7A6	0.0436729	0	0.0436729
SLC7A6OS	0.0235138	0	0.0235138
SLC7A7	0.0402946	0	0.0402946
SLC7A8	0.0418624	0	0.0418624
SLC7A9	0.0021879	0.0021879	0
SLC8A1	0.0641527	0	0.0641527
SLC8A2	0.0013373	0	0.0013373
SLC8A3	0.0181386	0	0.0181386
SLC9A1	0.0765143	0	0
SLC9A10	0.051042	0.051042	0
SLC9A11	0.0323625	0.0323625	0
SLC9A2	0.0117586	0.0117586	0
SLC9A3	0.0024842	0	0
SLC9A3R1	0.0144532	0.0144532	0
SLC9A3R2	0.0006982	0.0006982	0
SLC9A4	0.0390903	0	0
SLC9A5	0.0197703	0.0197703	0
SLC9A6	0.0136103	0	0
SLC9A7	0.0227949	0	0
SLC9A8	0.2698755	0.2698755	0
SLC9A9	0.0952178	0	0.0952178
SLCO1A2	0.0080394	0	0.0080394
SLCO1B1	0.0016317	0.0016317	0
SLCO1B3	0.0051171	0	0
SLCO1C1	0.0097883	0	0.0097883
SLCO2A1	0.0095332	0	0.0095332
SLCO2B1	0.0059688	0	0.0059688
SLCO3A1	0.0441447	0	0.0441447
SLCO4A1	0.0191786	0.0191786	0
SLCO4C1	0.0094528	0	0.0094528
SLCO5A1	0.0128745	0	0.0128745
SLCO6A1	0.0056641	0.0056641	0
SLED1	0.0753509	0.0753509	0
SLFN11	0.0263303	0	0.0263303
SLFN12	0.0603035	0	0.0603035
SLFN12L	0.0695465	0	0.0695465
SLFN13	0.0234853	0.0234853	0
SLFN14	0.0121764	0	0.0121764
SLFN5	0.4788941	0	0.4788941
SLFNL1	0.0035269	0.0035269	0
SLIT1	0.0017482	0	0.0017482

SLIT2	0.0073768	0	0.0073768
SLIT3	0.0086045	0	0.0086045
SLITRK1	0.0062643	0	0.0062643
SLITRK2	0.0029605	0	0.0029605
SLITRK3	0.0093314	0	0.0093314
SLITRK4	0.0062329	0	0.0062329
SLITRK5	0.0164463	0	0.0164463
SLITRK6	0.0413701	0	0
SLK	0.1020997	0.1020997	0
SLMAP	0.1432275	0.1432275	0
SLMO1	0.0160296	0.0160296	0
SLMO2	0.0688486	0.0688486	0
SLN	0.0267399	0	0.0267399
SLPI	0.6350259	0.6350259	0
SLTM	0.1235531	0	0
SLU7	0.2005167	0	0
SLURP1	0.006135	0	0
SMAD1	0.0468133	0	0.0468133
SMAD2	0.081327	0	0.081327
SMAD3	0.5141788	0.5141788	0
SMAD4	0.0489277	0	0.0489277
SMAD5	0.0744326	0	0.0744326
SMAD5OS	0.0612139	0	0.0612139
SMAD6	0.0501165	0.0501165	0
SMAD7	0.121157	0	0.121157
SMAD9	0.0040118	0	0.0040118
SMAGP	0.0431417	0.0431417	0
SMAP1	0.0449203	0	0
SMAP2	0.0558028	0	0.0558028
SMARCA1	0.0072409	0	0.0072409
SMARCA2	0.135696	0	0.135696
SMARCA4	0.0207452	0.0207452	0
SMARCA5	0.0471296	0	0.0471296
SMARCAD1	0.0474492	0	0.0474492
SMARCAL1	0.0457676	0	0.0457676
SMARCB1	0.0096803	0	0
SMARCC1	0.1787121	0.1787121	0
SMARCC2	0.0515959	0.0515959	0
SMARCD1	0.0358117	0.0358117	0
SMARCD2	0.0749707	0.0749707	0
SMARCD3	0.0082815	0	0.0082815
SMARCE1	0.0448271	0	0.0448271
SMC1A	0.0338963	0	0.0338963
SMC1B	0.0052756	0	0.0052756
SMC2	0.0882523	0	0

SMC3	0.1285141	0	0.1285141
SMC4	0.2079068	0	0
SMC5	0.0853669	0	0
SMC6	0.2252338	0	0.2252338
SMCHD1	0.0930708	0	0.0930708
SMCP	0.0672981	0	0.0672981
SMCR5	0.0042203	0.0042203	0
SMCR7	0.0071863	0.0071863	0
SMCR7L	0.0113167	0	0.0113167
SMCR8	0.0901201	0	0.0901201
SMEK1	0.0406736	0	0
SMEK2	0.1166553	0	0
SMEK3P	0.0046275	0	0
SMG1	0.6969799	0	0.6969799
SMG5	0.1211383	0.1211383	0
SMG6	0.0176984	0	0.0176984
SMG7	0.4657191	0.4657191	0
SMNDC1	0.3239125	0	0.3239125
SMO	0.0074595	0	0.0074595
SMOC1	0.0430143	0	0
SMOC2	0.0020459	0	0.0020459
SMOX	0.0054155	0.0054155	0
SMPD1	0.0718106	0	0.0718106
SMPD2	0.0126222	0.0126222	0
SMPD3	0.0075015	0.0075015	0
SMPD4	0.0037653	0.0037653	0
SMPDL3A	0.0178065	0	0
SMPDL3B	0.0127009	0.0127009	0
SMPX	0.0115932	0	0.0115932
SMR3A	0.0213538	0.0213538	0
SMR3B	0.0062662	0	0
SMS	0.0614362	0.0614362	0
SMTN	0.0067644	0	0.0067644
SMTNL1	0.0034756	0	0.0034756
SMTNL2	0.0007708	0	0.0007708
SMU1	0.1652387	0	0.1652387
SMUG1	0.0409389	0.0409389	0
SMURF1	0.032201	0.032201	0
SMURF2	0.0563246	0	0.0563246
SMYD1	0.0118575	0	0
SMYD2	0.0912083	0.0912083	0
SMYD3	0.0417568	0.0417568	0
SMYD4	0.0281354	0	0.0281354
SMYD5	0.0197012	0.0197012	0
SNAI1	0.0092496	0	0.0092496

SNAI2	0.1315852	0	0.1315852
SNAI3	0.0042888	0	0.0042888
SNAP23	0.3331915	0	0.3331915
SNAP25	0.0087806	0	0.0087806
SNAP29	0.0156092	0	0.0156092
SNAP47	0.0290288	0	0
SNAP91	0.0076258	0	0.0076258
SNAPC1	0.0725337	0	0.0725337
SNAPC2	0.0072902	0.0072902	0
SNAPC3	0.0621439	0	0
SNAPC4	0.0068313	0.0068313	0
SNAPC5	0.2991887	0	0
SNAPIN	0.1470312	0	0
SNAR-C3	2.30E-05	0	0
SNAR-C4	1.53E-05	0	0
SNAR-D	0.0020466	0	0
SNAR-E	0.0072388	0	0
SNAR-F	0.0009144	0	0
SNAR-G1	0.0002116	0	0
SNAR-G2	0.0001731	0	0
SNAR-H	0.0062918	0	0
SNAR-I	0.0131994	0	0
SNCA	0.0131307	0	0.0131307
SNCAIP	0.1621069	0	0.1621069
SNCB	0.0032312	0	0
SNCG	0.009169	0	0.009169
SND1	0.1840681	0.1840681	0
SNED1	0.0050969	0	0.0050969
SNF8	0.0129186	0.0129186	0
SNHG1	0.2203311	0.2203311	0
SNHG10	0.1259471	0.1259471	0
SNHG11	0.0252678	0.0252678	0
SNHG12	0.0630541	0.0630541	0
SNHG3	0.7429307	0.7429307	0
SNHG3-RCC1	0.7169327	0	0.7169327
SNHG4	0.1552333	0.1552333	0
SNHG5	0.2803415	0.2803415	0
SNHG6	0.0905918	0.0905918	0
SNHG7	0.0234835	0.0234835	0
SNHG8	0.2198802	0.2198802	0
SNHG9	0.1065417	0.1065417	0
SNIP1	0.022406	0	0.022406
SNN	0.0237998	0	0.0237998
SNORA1	0.4991809	0	0
SNORA10	0.0450627	0	0

SNORA11	0.0035863	0	0
SNORA11B	0.0162977	0	0
SNORA11C	0.0035718	0	0
SNORA12	0.0299657	0	0
SNORA13	0.068384	0	0
SNORA14A	0.1569895	0	0.1569895
SNORA14B	0.1201214	0	0.1201214
SNORA15	0.0619888	0	0
SNORA16A	0.0587455	0	0
SNORA16B	0.0652278	0	0.0652278
SNORA18	0.5070672	0	0
SNORA19	0.0308717	0	0
SNORA20	0.0685032	0	0
SNORA21	0.0704084	0	0
SNORA22	0.007154	0	0
SNORA23	0.0272198	0	0.0272198
SNORA24	0.1970928	0.1970928	0
SNORA25	0.3116361	0.3116361	0
SNORA26	0.0467569	0	0
SNORA27	0.0727998	0	0
SNORA28	0.1899005	0	0
SNORA29	0.0804793	0	0
SNORA2A	0.0166842	0	0
SNORA2B	0.0129771	0	0.0129771
SNORA3	0.1546399	0.1546399	0
SNORA30	0.0114845	0	0
SNORA31	0.2281216	0.2281216	0
SNORA32	0.3316852	0	0
SNORA34	0.0174017	0	0
SNORA35	0.0064519	0	0
SNORA36A	0.010729	0.010729	0
SNORA36B	0.0535226	0	0
SNORA36C	0.0097151	0	0
SNORA37	0.1091676	0	0
SNORA38B	0.0119694	0	0.0119694
SNORA39	0.020323	0.020323	0
SNORA4	0.306053	0	0
SNORA40	0.5400538	0	0
SNORA41	0.0514565	0	0
SNORA42	0.0797818	0	0
SNORA44	0.0560174	0	0
SNORA45	0.1601096	0	0
SNORA46	0.0276943	0	0
SNORA47	0.0319243	0	0
SNORA48	0.1097544	0	0

SNORA49	0.0034781	0	0
SNORA50	0.0222367	0	0
SNORA51	0.0271003	0	0
SNORA52	0.011952	0	0
SNORA53	0.0569884	0	0
SNORA54	0.0202055	0	0.0202055
SNORA55	0.0274952	0	0
SNORA56	0.0051636	0	0
SNORA57	0.0734667	0	0
SNORA58	0.0213794	0	0.0213794
SNORA5A	0.0101233	0	0
SNORA5B	0.0108978	0	0
SNORA5C	0.0110391	0	0
SNORA6	0.0661209	0	0
SNORA61	0.0544828	0.0544828	0
SNORA62	0.0539158	0	0
SNORA63	0.3051704	0	0
SNORA64	0.0496288	0	0
SNORA65	0.1492491	0	0
SNORA66	0.0858992	0	0
SNORA67	0.1220283	0	0
SNORA68	0.2750788	0	0
SNORA69	0.0213575	0.0213575	0
SNORA70	0.0121113	0	0
SNORA70B	0.0291282	0	0
SNORA70C	0.004629	0	0
SNORA71A	0.0570542	0.0570542	0
SNORA71B	0.0423135	0	0.0423135
SNORA71C	0.0391649	0.0391649	0
SNORA71D	0.0371698	0	0
SNORA72	0.0498543	0.0498543	0
SNORA74A	0.1605526	0	0
SNORA74B	0.0431403	0	0.0431403
SNORA75	0.050871	0	0
SNORA76	0.1395699	0.1395699	0
SNORA77	0.0142113	0	0
SNORA78	0.0999804	0	0
SNORA79	0.028099	0	0
SNORA7B	0.0220345	0.0220345	0
SNORA8	0.5150337	0.5150337	0
SNORA80	0.0221543	0	0
SNORA81	0.296322	0	0
SNORA84	0.0961651	0.0961651	0
SNORA9	0.8816315	0	0
SNORD10	0.108919	0	0

SNORD102	0.0733319	0	0
SNORD104	0.1549053	0	0
SNORD105	0.0400452	0	0
SNORD105B	0.0280044	0	0
SNORD11	0.0193999	0	0
SNORD110	0.0192881	0	0
SNORD111	0.0230411	0	0
SNORD111B	0.0729174	0	0
SNORD113-1	0.0104477	0	0
SNORD113-2	0.0116569	0	0
SNORD113-4	0.0121097	0	0
SNORD113-5	0.0116793	0	0
SNORD113-6	0.0110219	0	0
SNORD113-7	0.0108198	0	0
SNORD113-9	0.0111319	0	0
SNORD114-1	0.0112999	0	0
SNORD114-10	0.014258	0	0
SNORD114-11	0.0128241	0	0
SNORD114-12	0.0134452	0	0
SNORD114-13	0.015804	0	0
SNORD114-14	0.0290296	0	0
SNORD114-15	0.0319705	0	0
SNORD114-16	0.0344242	0	0
SNORD114-17	0.0353231	0	0
SNORD114-18	0.0377731	0	0
SNORD114-19	0.0377625	0	0
SNORD114-2	0.010322	0	0
SNORD114-20	0.0742798	0	0
SNORD114-21	0.0705276	0	0
SNORD114-22	0.064849	0	0
SNORD114-23	0.066861	0	0
SNORD114-24	0.0772234	0	0
SNORD114-25	0.0814356	0	0
SNORD114-26	0.0835054	0	0
SNORD114-27	0.0985862	0	0
SNORD114-28	0.1035345	0	0
SNORD114-29	0.0697659	0	0
SNORD114-3	0.010805	0	0
SNORD114-30	0.0759712	0	0
SNORD114-31	0.0961106	0	0
SNORD114-4	0.011294	0	0
SNORD114-5	0.0101516	0	0
SNORD114-6	0.0099905	0	0
SNORD114-7	0.0130653	0	0
SNORD114-8	0.0125153	0	0

SNORD114-9	0.0137726	0	0
SNORD115-1	0.0007701	0	0
SNORD115-13	0.0002656	0.0002656	0
SNORD115-14	0.0002526	0	0
SNORD115-16	0.0002406	0	0
SNORD115-2	0.0005583	0	0
SNORD115-20	0.000279	0	0
SNORD115-22	0.0003234	0	0
SNORD115-25	0.0003311	0	0
SNORD115-26	0.0002896	0	0
SNORD115-3	0.0004589	0	0
SNORD115-30	0.0002887	0	0
SNORD115-31	0.0002652	0	0
SNORD115-32	0.0002847	0	0
SNORD115-33	0.0002918	0	0
SNORD115-35	0.0003711	0	0
SNORD115-37	0.000348	0	0
SNORD115-38	0.0003369	0	0
SNORD115-39	0.0003597	0	0
SNORD115-4	0.0003783	0	0
SNORD115-40	0.0002926	0	0
SNORD115-41	0.0003324	0	0
SNORD115-44	0.0002636	0	0
SNORD115-48	0.0039524	0	0
SNORD115-6	0.0002243	0	0
SNORD115-7	0.0002169	0.0002169	0
SNORD115-8	0.000264	0	0
SNORD116-1	0.0018001	0	0
SNORD116-10	0.0014428	0	0
SNORD116-11	0.0019032	0	0
SNORD116-12	0.0018065	0	0
SNORD116-13	0.0019453	0	0
SNORD116-14	0.0020466	0	0
SNORD116-15	0.0021091	0	0
SNORD116-16	0.0022846	0	0
SNORD116-18	0.0027665	0	0
SNORD116-20	0.0025673	0	0.0025673
SNORD116-22	0.0027058	0	0
SNORD116-23	0.0030361	0	0
SNORD116-24	0.0029965	0	0
SNORD116-25	0.0030507	0	0
SNORD116-26	0.0034629	0	0
SNORD116-27	0.0034947	0	0
SNORD116-28	0.0034483	0	0.0034483
SNORD116-29	0.0036288	0	0

SNORD116-4	0.0008246	0	0.0008246
SNORD116-8	0.0011704	0	0
SNORD119	0.0653335	0	0
SNORD11B	0.0178134	0	0
SNORD12	0.5455159	0	0
SNORD121A	0.0274944	0	0
SNORD121B	0.010481	0	0
SNORD123	0.0130187	0	0
SNORD124	0.0815033	0	0
SNORD125	0.0016474	0	0
SNORD126	0.0695803	0	0
SNORD127	0.0199164	0	0
SNORD12B	0.5813544	0	0
SNORD12C	0.7440394	0	0
SNORD15A	0.0794859	0	0
SNORD15B	0.0651008	0	0
SNORD16	0.2168006	0	0
SNORD17	0.1943429	0	0
SNORD18A	0.2157441	0	0
SNORD18B	0.216799	0	0
SNORD18C	0.2223386	0	0
SNORD19	0.1978652	0	0
SNORD19B	0.1742412	0	0
SNORD1A	0.1113651	0	0
SNORD1B	0.1345316	0	0
SNORD1C	0.1987815	0	0
SNORD2	0.3143578	0	0
SNORD20	0.0538732	0	0
SNORD21	0.0868178	0	0
SNORD22	0.160544	0	0
SNORD23	0.0032656	0	0
SNORD24	0.048199	0	0
SNORD25	0.2150736	0	0
SNORD26	0.2048292	0	0
SNORD27	0.1920909	0	0
SNORD28	0.1773464	0	0
SNORD29	0.1680871	0	0
SNORD30	0.1690402	0	0
SNORD31	0.1663918	0	0
SNORD32A	0.1047198	0	0
SNORD33	0.1050148	0	0
SNORD34	0.1059441	0	0
SNORD35A	0.1101422	0	0
SNORD35B	0.1128996	0	0
SNORD36A	0.0493885	0	0

SNORD36B	0.0467019	0	0
SNORD36C	0.0498517	0	0
SNORD37	0.0335578	0	0
SNORD38A	0.0419704	0	0
SNORD38B	0.0393078	0	0
SNORD41	0.0040613	0	0
SNORD42A	0.3312499	0	0
SNORD42B	0.3987974	0	0
SNORD43	0.0895424	0	0
SNORD44	0.4077443	0	0
SNORD45A	0.1589453	0	0
SNORD45B	0.1675039	0	0
SNORD45C	0.1939069	0	0
SNORD46	0.0467918	0	0
SNORD47	0.4023521	0	0
SNORD49A	0.088001	0	0
SNORD49B	0.0949156	0	0
SNORD4A	0.3113023	0	0
SNORD4B	0.3478184	0	0
SNORD5	0.5215414	0	0
SNORD50A	0.2757857	0	0
SNORD50B	0.2735037	0	0
SNORD51	0.0526717	0	0
SNORD53	0.0356313	0	0
SNORD54	0.0749455	0	0
SNORD55	0.0435153	0	0
SNORD56	0.0275563	0	0
SNORD56B	0.0895444	0	0
SNORD57	0.0273585	0	0
SNORD58A	0.1309133	0	0
SNORD58C	0.1298161	0	0
SNORD59A	0.1052787	0	0
SNORD59B	0.1006653	0	0
SNORD6	0.3938008	0	0
SNORD60	0.0446057	0	0
SNORD61	0.0111405	0	0
SNORD63	0.0792276	0	0
SNORD65	0.0764833	0	0
SNORD66	0.0141825	0	0
SNORD67	0.0179306	0	0
SNORD68	0.0289315	0	0
SNORD69	0.1214717	0	0
SNORD7	0.0611755	0	0
SNORD70	0.4939633	0	0
SNORD71	0.0188744	0	0

SNORD72	0.4130638	0	0
SNORD73A	0.0397275	0	0
SNORD74	0.5484931	0	0
SNORD75	0.4355751	0	0
SNORD76	0.426247	0	0
SNORD77	0.4194742	0	0
SNORD78	0.4031602	0	0
SNORD79	0.404537	0	0
SNORD8	0.0125741	0	0
SNORD80	0.3898945	0	0
SNORD81	0.4027546	0	0
SNORD82	0.0458054	0	0
SNORD85	0.0294539	0	0
SNORD86	0.0287256	0	0
SNORD87	0.0870065	0	0
SNORD88A	0.0441795	0	0
SNORD88B	0.0442587	0	0
SNORD88C	0.0537858	0	0
SNORD89	0.0421256	0	0
SNORD9	0.0338338	0	0
SNORD90	0.0292836	0	0
SNORD91A	0.0231369	0	0
SNORD91B	0.0245058	0	0
SNORD92	0.0187123	0	0
SNORD93	0.1361778	0	0
SNORD94	0.0232172	0	0
SNORD95	0.237521	0	0
SNORD96A	0.1976757	0	0
SNORD96B	0.0254374	0	0
SNORD97	0.1199986	0	0
SNORD98	0.0081001	0	0
SNORD99	0.0477708	0	0
SNPH	0.0036973	0	0.0036973
SNRK	0.0411056	0	0.0411056
SNRNP200	0.0305731	0	0
SNRNP25	0.005653	0.005653	0
SNRNP27	0.0905313	0	0.0905313
SNRNP35	0.0179768	0.0179768	0
SNRNP40	0.0465911	0	0.0465911
SNRNP48	0.0451725	0	0
SNRNP70	0.2683588	0.2683588	0
SNRPA	0.118058	0.118058	0
SNRPA1	0.1047657	0.1047657	0
SNRPB	0.1151871	0.1151871	0
SNRPB2	0.0414881	0	0

SNRPC	0.0390397	0.0390397	0
SNRPD1	0.0222824	0.0222824	0
SNRPD2	0.0154016	0.0154016	0
SNRPD3	0.0283663	0.0283663	0
SNRPE	0.3572556	0.3572556	0
SNRPF	0.0454018	0.0454018	0
SNRPG	0.0641094	0.0641094	0
SNRPN	0.0065895	0	0.0065895
SNTA1	0.0039912	0	0
SNTB1	0.0578116	0	0.0578116
SNTB2	0.2750869	0	0.2750869
SNTG1	0.0119784	0	0
SNTG2	0.0030216	0	0.0030216
SNTN	0.0012724	0.0012724	0
SNUPN	0.0356209	0	0
SNURF	0.0051089	0	0.0051089
SNW1	0.0343127	0	0
SNX1	0.418858	0	0.418858
SNX10	0.0409938	0	0.0409938
SNX11	0.0769099	0	0
SNX12	0.0418685	0	0
SNX13	0.1690032	0	0
SNX14	0.0686357	0	0
SNX15	0.0464238	0.0464238	0
SNX16	0.4291068	0	0.4291068
SNX17	0.0880896	0	0.0880896
SNX18	0.0161889	0	0.0161889
SNX19	0.0310771	0	0.0310771
SNX2	0.0645101	0	0.0645101
SNX20	0.0317487	0	0.0317487
SNX21	0.0108064	0	0
SNX22	0.0164855	0	0.0164855
SNX24	0.058817	0	0
SNX25	0.0797616	0	0.0797616
SNX27	0.1791137	0	0
SNX29	0.0034013	0	0.0034013
SNX3	0.050624	0	0.050624
SNX30	0.0180898	0	0.0180898
SNX31	0.0085482	0	0.0085482
SNX32	0.007223	0	0.007223
SNX33	0.0437244	0	0
SNX4	0.0378308	0	0
SNX5	0.1739799	0	0.1739799
SNX6	0.0102171	0	0.0102171
SNX7	0.0229812	0	0

SNX8	0.0253717	0	0
SNX9	0.051866	0	0.051866
SOAT1	0.2410706	0	0.2410706
SOAT2	0.0161518	0	0
SOBP	0.0093516	0	0.0093516
SOCS1	0.0245614	0	0.0245614
SOCS2	0.1242386	0	0.1242386
SOCS3	0.5139218	0	0.5139218
SOCS4	0.0771464	0	0
SOCS5	0.0955961	0	0.0955961
SOCS6	0.0699647	0	0
SOCS7	0.0255674	0	0
SOD1	0.0957941	0.0957941	0
SOD2	0.6503408	0	0.6503408
SOD3	0.0016995	0	0.0016995
SOHLH1	0.0007817	0	0
SOHLH2	0.0036495	0	0.0036495
SOLH	0.0060396	0.0060396	0
SON	0.1281885	0	0.1281885
SORBS1	0.0256727	0	0.0256727
SORBS2	0.0172575	0	0
SORBS3	0.0232985	0	0.0232985
SORCS1	0.005109	0	0.005109
SORCS2	0.0013434	0	0.0013434
SORCS3	0.0029369	0	0.0029369
SORD	0.013205	0.013205	0
SORL1	0.1092774	0	0.1092774
SORT1	0.1037118	0	0.1037118
SOS1	0.1044994	0	0.1044994
SOS2	0.087217	0	0.087217
SOST	0.0131608	0	0.0131608
SOSTDC1	0.0095952	0.0095952	0
SOX1	0.0005835	0	0
SOX10	0.0006983	0	0.0006983
SOX11	0.0018233	0	0.0018233
SOX12	0.0210596	0.0210596	0
SOX13	0.0253173	0.0253173	0
SOX14	0.0011412	0.0011412	0
SOX15	0.0237685	0	0
SOX17	0.0152399	0	0.0152399
SOX18	0.0074128	0	0.0074128
SOX2	0.0115858	0	0.0115858
SOX21	0.0016239	0.0016239	0
SOX2OT	0.0146592	0	0.0146592
SOX3	0.0014139	0	0

SOX30	0.0151932	0	0
SOX4	0.1443027	0	0
SOX5	0.0269735	0	0.0269735
SOX6	0.0147638	0	0.0147638
SOX7	0.009405	0	0.009405
SOX8	0.0002927	0	0.0002927
SOX9	0.0395968	0.0395968	0
SP1	0.5378674	0	0
SP100	0.3244388	0	0.3244388
SP110	0.2504517	0	0.2504517
SP140	0.1555825	0	0.1555825
SP140L	0.0322424	0	0
SP2	0.0512828	0	0
SP3	0.056506	0	0.056506
SP4	0.0345357	0	0.0345357
SP5	0.0030127	0.0030127	0
SP6	0.0040811	0	0
SP7	0.0067407	0	0
SP8	0.0052628	0	0
SP9	0.0054847	0	0
SPA17	0.2195515	0.2195515	0
SPACA1	0.0118668	0.0118668	0
SPACA3	0.0041893	0	0.0041893
SPACA4	0.0150404	0.0150404	0
SPAG1	0.1173361	0.1173361	0
SPAG11A	1.70E-05	0	0
SPAG11B	3.54E-05	0	0
SPAG16	0.0308811	0.0308811	0
SPAG17	0.0118785	0	0
SPAG4	0.0974673	0.0974673	0
SPAG5	0.0410666	0.0410666	0
SPAG6	0.0594845	0	0.0594845
SPAG7	0.0497143	0	0
SPAG8	0.026027	0	0
SPAG9	0.0833197	0	0.0833197
SPAM1	0.0335929	0.0335929	0
SPANXC	5.25E-05	0	0
SPANXE	0.000179	0	0
SPANXN1	0.001736	0	0.001736
SPANXN2	0.0011945	0	0
SPANXN3	0.0020487	0	0.0020487
SPANXN4	0.0066538	0.0066538	0
SPANXN5	0.0003753	0	0
SPARC	0.0201507	0	0.0201507
SPARCL1	0.0299731	0	0.0299731

SPAST	0.0551802	0	0.0551802
SPATA1	0.0957871	0	0
SPATA12	0.1426618	0.1426618	0
SPATA13	0.0049704	0	0.0049704
SPATA16	0.0148024	0.0148024	0
SPATA17	0.1573597	0.1573597	0
SPATA18	0.0187596	0	0
SPATA19	0.0018834	0	0
SPATA2	0.0252794	0.0252794	0
SPATA20	0.0082114	0.0082114	0
SPATA21	0.0070524	0	0
SPATA22	0.0048676	0	0.0048676
SPATA24	0.1292778	0.1292778	0
SPATA2L	0.0047086	0.0047086	0
SPATA3	0.0032806	0	0
SPATA4	0.0245174	0	0
SPATA5	0.0635988	0	0
SPATA5L1	0.1932911	0.1932911	0
SPATA6	0.0063314	0	0
SPATA7	0.0172757	0	0
SPATA8	0.0028526	0	0.0028526
SPATA9	0.0194431	0	0.0194431
SPATC1	0.0533205	0	0.0533205
SPATS1	0.0121754	0	0
SPATS2	0.17816	0	0.17816
SPATS2L	0.0763245	0	0
SPC24	0.0425004	0.0425004	0
SPC25	0.0180297	0.0180297	0
SPCS1	0.0537964	0.0537964	0
SPCS2	0.1018129	0	0
SPCS3	0.0549116	0	0.0549116
SPDEF	0.0013867	0.0013867	0
SPDYA	0.027437	0.027437	0
SPDYC	0.0054788	0.0054788	0
SPDYE1	8.05E-05	0	0
SPDYE3	5.83E-05	0	0
SPDYE4	0.0057238	0	0.0057238
SPDYE5	4.55E-05	4.55E-05	0
SPDYE6	9.16E-05	0	0
SPDYE7P	0.0001114	0.0001114	0
SPEF1	0.0152889	0.0152889	0
SPEF2	0.0416724	0	0
SPEG	0.0069721	0	0
SPEM1	0.0007904	0	0
SPEN	0.0639274	0	0.0639274

SPERT	0.079101	0	0
SPESP1	0.0195883	0	0.0195883
SPG11	0.1417048	0	0
SPG20	0.0663357	0	0.0663357
SPG21	0.0593631	0	0.0593631
SPG7	0.0182092	0.0182092	0
SPHAR	0.0204645	0	0
SPHK1	0.0054634	0	0.0054634
SPHK2	0.1028554	0.1028554	0
SPHKAP	0.0032669	0	0.0032669
SPI1	0.0218417	0	0.0218417
SPIB	0.0069255	0	0.0069255
SPIC	0.0060565	0	0.0060565
SPIN1	0.0402668	0	0.0402668
SPIN2A	0.0061177	0	0.0061177
SPIN2B	0.0140359	0.0140359	0
SPIN3	0.0331107	0.0331107	0
SPIN4	0.0142234	0.0142234	0
SPINK1	0.0596652	0	0
SPINK13	0.0267371	0	0
SPINK14	0.0205105	0.0205105	0
SPINK2	0.0140404	0	0.0140404
SPINK4	0.1044528	0	0
SPINK5	0.0672299	0.0672299	0
SPINK6	0.094563	0	0
SPINK7	0.0113096	0.0113096	0
SPINK8	0.0054046	0	0
SPINK9	0.0177568	0	0
SPINLW1	0.0160041	0	0
SPINT1	0.0179466	0.0179466	0
SPINT2	0.0100452	0.0100452	0
SPINT3	0.0631588	0	0
SPINT4	0.0074424	0	0
SPIRE1	0.0150395	0	0
SPIRE2	0.0048783	0.0048783	0
SPN	0.02843	0	0.02843
SPNS1	0.0157586	0	0
SPNS2	0.0015355	0	0
SPNS3	0.0022695	0	0.0022695
SPO11	0.0185766	0	0
SPOCD1	0.0087384	0	0.0087384
SPOCK1	0.0187446	0	0.0187446
SPOCK2	0.0214992	0	0.0214992
SPOCK3	0.0094007	0	0
SPON1	0.0051046	0	0.0051046

SPON2	0.0035356	0	0.0035356
SPOP	0.0372328	0	0.0372328
SPOPL	0.0411779	0	0
SPP1	0.0159264	0	0.0159264
SPP2	0.0127652	0.0127652	0
SPPL2A	0.0755939	0	0.0755939
SPPL2B	0.0199313	0.0199313	0
SPPL3	0.0215984	0.0215984	0
SPR	0.0323938	0.0323938	0
SPRED1	0.1317386	0	0.1317386
SPRED2	0.2888921	0	0
SPRED3	0.0061344	0	0
SPRN	0.0001767	0.0001767	0
SPRR1A	0.0205233	0.0205233	0
SPRR1B	0.0601358	0	0
SPRR2A	0.0320623	0	0
SPRR2B	0.019017	0	0
SPRR2C	0.016987	0	0
SPRR2D	0.1298697	0	0
SPRR2E	0.088904	0	0
SPRR2F	0.0482044	0	0
SPRR2G	0.0171902	0	0
SPRR3	0.0106541	0	0
SPRR4	0.0466706	0.0466706	0
SPRY1	0.057943	0	0.057943
SPRY2	0.0990655	0	0
SPRY4	0.1026556	0	0
SPRYD3	0.0171405	0	0
SPRYD4	0.0564225	0.0564225	0
SPRYD5	0.0010149	0	0
SPSB1	0.0882167	0	0.0882167
SPSB2	0.015537	0.015537	0
SPSB3	0.0175609	0.0175609	0
SPSB4	0.0076888	0	0.0076888
SPTA1	0.0146045	0	0.0146045
SPTAN1	0.1490495	0	0.1490495
SPTB	0.0056077	0	0
SPTBN1	0.3457965	0	0
SPTBN2	0.0073983	0.0073983	0
SPTBN4	0.0167173	0	0.0167173
SPTBN5	0.004449	0.004449	0
SPTLC1	0.0451141	0.0451141	0
SPTLC2	0.0451193	0	0
SPTLC3	0.1018003	0	0.1018003
SPTY2D1	0.0320155	0	0.0320155

SPZ1	0.0031521	0	0
SQLE	0.0971744	0.0971744	0
SQRDL	0.6604711	0.6604711	0
SQSTM1	0.0469907	0.0469907	0
SR140	0.2117221	0	0
SRA1	0.1391839	0.1391839	0
SRBD1	0.2156019	0	0.2156019
SRC	0.0223398	0.0223398	0
SRCAP	0.0262142	0.0262142	0
SRCIN1	0.0028821	0.0028821	0
SRCRB4D	0.003942	0	0
SRD5A1	0.1314758	0.1314758	0
SRD5A2	0.0035447	0	0
SRD5A3	0.0287785	0.0287785	0
SREBF1	0.0557371	0.0557371	0
SREBF2	0.0661824	0.0661824	0
SRF	0.1157434	0	0.1157434
SRFBP1	0.7509926	0	0.7509926
SRGAP1	0.0796356	0	0
SRGAP2	9.72E-05	0	9.72E-05
SRGAP3	0.0195045	0	0.0195045
SRGN	0.1228339	0	0.1228339
SRI	0.3692304	0.3692304	0
SRL	0.0020301	0	0.0020301
SRM	0.0161585	0	0.0161585
SRMS	0.0156476	0.0156476	0
SRP14	0.2396949	0	0
SRP19	0.0498539	0	0
SRP54	0.1331986	0	0
SRP68	0.0114441	0.0114441	0
SRP72	0.0787025	0	0.0787025
SRP9	0.1907427	0.1907427	0
SRPK1	0.0511626	0.0511626	0
SRPK2	0.3035551	0	0.3035551
SRPK3	0.0002494	0.0002494	0
SRPR	0.0180345	0	0.0180345
SRPRB	0.0040703	0	0
SRPX	0.009701	0	0.009701
SRPX2	0.026895	0	0.026895
SRR	0.0181248	0	0.0181248
SRRD	0.0454451	0	0
SRRM1	0.0707222	0	0
SRRM2	0.0279813	0.0279813	0
SRRM3	0.0011395	0	0.0011395
SRRM4	0.0033224	0	0

SRRM5	0.0047476	0.0047476	0
SRRT	0.0252802	0.0252802	0
SRXN1	0.0062311	0	0
SRY	1.53E-05	0	0
SS18	0.1176799	0	0.1176799
SS18L1	0.0131395	0.0131395	0
SS18L2	0.0680112	0	0
SSB	0.0381793	0	0
SSBP1	0.0851227	0.0851227	0
SSBP2	0.1017087	0	0.1017087
SSBP3	0.0341184	0.0341184	0
SSBP4	0.0133275	0.0133275	0
SSC5D	0.0012318	0	0.0012318
SSFA2	0.136826	0	0
SSH1	0.2370291	0	0.2370291
SSH2	0.0942661	0	0.0942661
SSH3	0.0053714	0.0053714	0
SSNA1	0.0123851	0.0123851	0
SSPN	0.0374042	0	0.0374042
SSPO	0.0008993	0	0
SSR1	0.1689916	0	0.1689916
SSR2	0.0569409	0.0569409	0
SSR3	0.128167	0	0.128167
SSR4	0.0017305	0.0017305	0
SSRP1	0.0240292	0.0240292	0
SSSCA1	0.1719896	0.1719896	0
SST	0.0354224	0	0.0354224
SSTR1	0.1809285	0.1809285	0
SSTR2	0.0162646	0	0.0162646
SSTR3	0.0059835	0	0.0059835
SSTR4	0.0034301	0	0.0034301
SSTR5	0.0055147	0.0055147	0
SSU72	0.0106623	0.0106623	0
SSX1	0.0004919	0	0
SSX2IP	0.0883687	0	0
SSX3	0.0012644	0	0
SSX5	0.0021257	0	0.0021257
SSX6	0.0016745	0	0
SSX7	0.0009945	0	0.0009945
SSX8	0.0026225	0	0
ST13	0.0575516	0	0.0575516
ST14	0.0105159	0.0105159	0
ST18	0.0084701	0	0.0084701
ST20	0.0610178	0.0610178	0
ST3GAL1	0.3261414	0	0

ST3GAL2	0.02342	0	0.02342
ST3GAL3	0.0448978	0	0.0448978
ST3GAL4	0.022956	0	0.022956
ST3GAL5	0.0352543	0	0.0352543
ST3GAL6	0.0323947	0	0.0323947
ST5	0.7765115	0	0
ST6GAL1	0.1371505	0	0.1371505
ST6GAL2	0.0012752	0	0.0012752
ST6GALNAC1	0.0607399	0.0607399	0
ST6GALNAC2	0.0081931	0	0
ST6GALNAC3	0.0100407	0	0.0100407
ST6GALNAC4	0.1079457	0	0
ST6GALNAC5	0.0086845	0	0.0086845
ST6GALNAC6	0.026896	0	0.026896
ST7	0.1155476	0.1155476	0
ST7L	0.1916907	0	0.1916907
ST7OT1	0.1250765	0	0
ST7OT2	0.0215815	0	0
ST7OT3	0.0231077	0	0
ST7OT4	0.1174659	0	0
ST8SIA1	0.0049221	0	0.0049221
ST8SIA2	0.0055092	0	0.0055092
ST8SIA3	0.0051752	0	0
ST8SIA4	0.1209222	0	0.1209222
ST8SIA5	0.0020389	0	0
ST8SIA6	0.0042747	0	0.0042747
STAB1	0.0023026	0	0.0023026
STAB2	0.0187391	0	0.0187391
STAC	0.0151734	0	0.0151734
STAC2	0.0027866	0	0
STAC3	0.0064567	0	0.0064567
STAG1	0.2394302	0	0.2394302
STAG2	0.0600618	0	0.0600618
STAG3	0.0048772	0	0.0048772
STAG3L1	1.53E-05	1.53E-05	0
STAG3L2	3.43E-05	3.43E-05	0
STAG3L3	1.53E-05	1.53E-05	0
STAG3L4	0.0104178	0	0
STAM	0.0805746	0	0.0805746
STAM2	0.0451991	0	0
STAMBP	0.1364611	0.1364611	0
STAMBPL1	0.2316863	0	0
STAP1	0.0223108	0	0.0223108
STAP2	0.0071629	0.0071629	0
STAR	0.0031632	0	0.0031632

STARD10	0.0375545	0.0375545	0
STARD13	0.3651406	0	0.3651406
STARD3	0.0226897	0.0226897	0
STARD3NL	0.1271241	0	0.1271241
STARD4	0.1245401	0	0
STARD5	0.0242876	0	0.0242876
STARD6	0.0242135	0	0
STARD7	0.0655846	0	0.0655846
STARD8	0.0038175	0	0.0038175
STAT1	1	0	1
STAT2	1	0	1
STAT3	1	0	1
STAT4	0.0500732	0	0.0500732
STAT5A	0.012744	0	0.012744
STAT5B	0.0856216	0	0.0856216
STAT6	0.1155325	0	0
STATH	0.0127051	0	0
STAU1	0.0818083	0.0818083	0
STAU2	0.0293123	0	0
STBD1	0.0257058	0.0257058	0
STC1	0.0364163	0	0.0364163
STC2	0.1057174	0	0.1057174
STEAP1	0.047649	0	0
STEAP2	0.3529884	0	0
STEAP3	0.007587	0.007587	0
STEAP4	0.1423231	0	0.1423231
STH	0.0029951	0	0
STIL	0.0237374	0.0237374	0
STIM1	0.0743519	0	0.0743519
STIM2	0.0513191	0	0.0513191
STIP1	0.0428879	0.0428879	0
STK10	0.0499043	0	0.0499043
STK11	0.0019587	0.0019587	0
STK11IP	0.0173714	0.0173714	0
STK16	0.0203726	0.0203726	0
STK17A	0.5548681	0	0.5548681
STK17B	0.15707	0	0.15707
STK24	0.1066013	0.1066013	0
STK25	0.0061186	0.0061186	0
STK3	0.1136068	0	0
STK31	0.0337599	0.0337599	0
STK32A	0.0108998	0	0
STK32B	0.0023547	0	0.0023547
STK32C	0.0022424	0.0022424	0
STK33	0.0312747	0	0

STK35	0.2315976	0.2315976	0
STK36	0.0090537	0.0090537	0
STK38	0.0498212	0	0.0498212
STK38L	0.1155845	0	0.1155845
STK39	0.0186198	0.0186198	0
STK4	0.1794042	0	0.1794042
STK40	0.1246718	0	0.1246718
STL	0.1147928	0	0
STMN1	0.0308869	0	0
STMN2	0.040711	0	0.040711
STMN3	0.0038832	0	0.0038832
STMN4	0.0054762	0	0.0054762
STOM	1	0	1
STOML1	0.2950438	0.2950438	0
STOML2	0.0440136	0.0440136	0
STOML3	0.007526	0	0
STON1	0.0046527	0	0.0046527
STON1-GTF2/	0.0480691	0	0.0480691
STON2	0.21399	0.21399	0
STOX1	0.0036443	0	0
STOX2	0.0053418	0	0.0053418
STRA13	0.0018988	0.0018988	0
STRA6	0.09025	0	0.09025
STRA8	0.0144393	0	0
STRADA	0.1038318	0	0.1038318
STRADB	0.0300909	0	0.0300909
STRAP	0.0415021	0	0
STRBP	0.0233992	0.0233992	0
STRC	0.0003642	0.0003642	0
STRN	0.0363591	0	0.0363591
STRN3	0.2455207	0	0
STRN4	0.0047307	0.0047307	0
STS	0.0100638	0	0.0100638
STT3A	0.0463253	0.0463253	0
STT3B	0.1085052	0	0.1085052
STUB1	0.0032036	0.0032036	0
STX10	0.1975166	0.1975166	0
STX11	0.2045269	0	0.2045269
STX12	0.0543267	0	0.0543267
STX16	0.0913434	0.0913434	0
STX17	0.0270337	0	0
STX18	0.0294944	0.0294944	0
STX19	0.0118348	0.0118348	0
STX1A	0.0263522	0.0263522	0
STX1B	0.0010165	0	0.0010165

STX2	0.0114495	0	0.0114495
STX3	0.0230246	0.0230246	0
STX4	0.011189	0	0
STX5	1	0	0
STX6	0.1829945	0	0
STX7	0.0463286	0	0.0463286
STX8	0.0227204	0	0
STXBP1	0.0327416	0	0
STXBP2	0.0045695	0.0045695	0
STXBP3	0.0773254	0	0.0773254
STXBP4	0.07193	0	0.07193
STXBP5	0.0716886	0	0.0716886
STXBP5L	0.0075093	0	0.0075093
STXBP6	0.0103482	0	0
STYK1	0.0300119	0.0300119	0
STYX	0.0408271	0	0.0408271
STYXL1	0.0250833	0.0250833	0
SUB1	0.4044583	0	0.4044583
SUCLA2	0.0496013	0	0
SUCLG1	0.0626885	0.0626885	0
SUCLG2	0.0596192	0.0596192	0
SUCNR1	0.1117007	0	0.1117007
SUDS3	0.0500302	0.0500302	0
SUFU	0.0661438	0	0.0661438
SUGT1	0.0775297	0.0775297	0
SUGT1L1	0.0162643	0.0162643	0
SUGT1P1	0.0377285	0	0.0377285
SULF1	0.0090959	0	0.0090959
SULF2	0.197415	0	0.197415
SULT1A1	0.001616	0	0.001616
SULT1A2	0.0009396	0	0
SULT1B1	0.03364	0	0
SULT1C2	0.0048826	0.0048826	0
SULT1C3	0.0028538	0.0028538	0
SULT1C4	0.0073847	0	0.0073847
SULT1E1	0.031671	0	0
SULT2A1	0.0028375	0.0028375	0
SULT2B1	0.0277457	0.0277457	0
SULT4A1	0.0015857	0	0
SULT6B1	0.049647	0	0
SUMF1	0.1421482	0.1421482	0
SUMF2	0.0405226	0.0405226	0
SUMO1	0.0195322	0.0195322	0
SUMO1P1	0.0782692	0	0.0782692
SUMO1P3	0.0489124	0.0489124	0

SUMO2	0.0303899	0	0
SUMO3	0.0031922	0	0.0031922
SUMO4	0.0232256	0	0
SUN1	0.0798558	0.0798558	0
SUN2	0.0139067	0	0.0139067
SUN3	0.0149847	0	0
SUN5	0.0032181	0	0
SUOX	0.0250468	0	0
SUPT16H	0.0707075	0	0
SUPT3H	0.0660016	0	0.0660016
SUPT4H1	0.0487579	0	0.0487579
SUPT5H	0.0629739	0.0629739	0
SUPT6H	0.0463061	0	0
SUPT7L	0.0579699	0	0.0579699
SUPV3L1	0.0797358	0	0
SURF1	0.0509697	0.0509697	0
SURF2	0.0516388	0.0516388	0
SURF4	0.0239013	0	0
SURF6	0.0194244	0.0194244	0
SUSD1	0.0166911	0	0.0166911
SUSD2	0.0008832	0	0.0008832
SUSD3	0.0069533	0	0.0069533
SUSD4	0.0069532	0.0069532	0
SUSD5	0.0117298	0	0.0117298
SUV39H1	0.0062801	0	0
SUV39H2	0.0395008	0	0.0395008
SUV420H1	0.0210733	0	0
SUV420H2	0.0485663	0.0485663	0
SUZ12	0.0128633	0	0.0128633
SUZ12P	0.0076978	0.0076978	0
SV2A	0.0155166	0	0.0155166
SV2B	0.0073049	0	0.0073049
SV2C	0.0062672	0	0.0062672
SVEP1	0.0423243	0	0.0423243
SVIL	0.3899405	0	0.3899405
SVIP	0.0456551	0.0456551	0
SVOP	0.0078951	0	0.0078951
SVOPL	0.0082368	0	0
SWAP70	0.0709087	0	0.0709087
SYAP1	0.0434789	0	0
SYCE1	0.0006512	0	0.0006512
SYCE1L	0.149158	0.149158	0
SYCE2	0.0048705	0.0048705	0
SYCN	0.0021701	0	0
SYCP1	0.0114229	0	0.0114229

SYCP2	0.0445793	0	0
SYCP2L	0.0094403	0	0
SYCP3	0.0177373	0	0
SYDE1	0.009063	0	0.009063
SYDE2	0.0287974	0	0
SYF2	0.1633089	0	0
SYK	0.0414855	0	0.0414855
SYMPK	0.0112079	0.0112079	0
SYN1	0.0048485	0	0
SYN2	0.0056487	0	0.0056487
SYN3	0.2538243	0	0.2538243
SYNC	0.0037579	0	0.0037579
SYNCRIP	0.0735896	0	0.0735896
SYNE1	0.0170939	0	0.0170939
SYNE2	0.0492814	0	0
SYNGAP1	0.0523836	0	0.0523836
SYNGR1	0.002242	0	0
SYNGR2	0.03927	0.03927	0
SYNGR3	0.0057202	0	0
SYNGR4	0.011072	0	0
SYNJ1	0.0237809	0	0.0237809
SYNJ2	0.0077123	0	0.0077123
SYNJ2BP	0.0374425	0.0374425	0
SYNM	0.010706	0	0.010706
SYNPO	0.4666109	0	0.4666109
SYNPO2	0.0151368	0	0.0151368
SYNPO2L	0.0133487	0	0
SYNPR	0.0049117	0	0.0049117
SYNRG	0.0461811	0	0.0461811
SYP	0.0096082	0	0
SYPL1	0.0562096	0.0562096	0
SYPL2	0.0076035	0	0.0076035
SYS1	0.2657826	0	0.2657826
SYS1-DBNDD2	0.2655077	0	0
SYT1	0.0164289	0	0.0164289
SYT10	0.0043297	0	0
SYT11	0.1555244	0	0.1555244
SYT12	0.0376613	0	0
SYT13	0.0136139	0.0136139	0
SYT14	0.033083	0	0.033083
SYT14L	0.0064615	0	0
SYT15	0.0150548	0	0.0150548
SYT16	0.0151294	0	0.0151294
SYT17	0.0054472	0.0054472	0
SYT2	0.0142888	0	0

SYT3	0.0031954	0	0.0031954
SYT4	0.0086225	0	0.0086225
SYT5	0.0283983	0	0
SYT6	0.0051782	0	0.0051782
SYT7	0.0063282	0	0
SYT8	0.0221441	0.0221441	0
SYT9	0.0060233	0	0
SYTL1	0.0076283	0.0076283	0
SYTL2	0.3463085	0	0
SYTL3	0.1622234	0	0.1622234
SYTL4	0.0050286	0	0
SYTL5	0.0170063	0.0170063	0
SYVN1	0.0524085	0.0524085	0
T	0.0030349	0	0
TAAR1	0.0325439	0	0
TAAR2	0.0103897	0.0103897	0
TAAR5	0.005234	0	0
TAAR6	0.0119027	0.0119027	0
TAAR8	0.0071557	0	0
TAAR9	0.014656	0	0
TAB1	0.0118444	0	0
TAB2	0.0572513	0	0.0572513
TAB3	0.1498676	0	0.1498676
TAC1	0.0066855	0	0.0066855
TAC3	0.0093701	0	0
TAC4	0.0040411	0	0
TACC1	0.1400925	0	0.1400925
TACC2	0.0058306	0.0058306	0
TACC3	0.0169048	0.0169048	0
TACO1	0.028005	0.028005	0
TACR1	0.0093191	0	0.0093191
TACR2	0.0065421	0.0065421	0
TACR3	0.0021421	0.0021421	0
TACSTD2	0.2372136	0.2372136	0
TADA1	0.0895752	0.0895752	0
TADA2A	0.0825589	0	0
TADA2B	0.0042852	0	0
TADA3	0.0864641	0.0864641	0
TAF1	0.0320564	0	0.0320564
TAF10	0.1282602	0.1282602	0
TAF11	0.081525	0	0.081525
TAF12	0.2023726	0	0.2023726
TAF13	0.0439264	0	0.0439264
TAF15	0.1527358	0.1527358	0
TAF1A	0.1978473	0	0

TAF1B	0.1673439	0	0
TAF1C	0.0080793	0.0080793	0
TAF1D	0.6045748	0.6045748	0
TAF1L	0.0954135	0	0.0954135
TAF2	0.1856402	0	0
TAF3	0.0198834	0	0.0198834
TAF4	0.0044716	0.0044716	0
TAF4B	0.0737531	0	0.0737531
TAF5	0.0594148	0	0.0594148
TAF5L	0.0938696	0	0
TAF6	0.0423494	0.0423494	0
TAF6L	0.0270837	0.0270837	0
TAF7	0.0992621	0.0992621	0
TAF7L	0.0046059	0	0.0046059
TAF8	0.0918935	0	0.0918935
TAGAP	0.0561998	0	0.0561998
TAGLN	0.000968	0	0.000968
TAGLN2	0.2352553	0.2352553	0
TAGLN3	0.0202319	0	0.0202319
tAKR	0.0065409	0	0.0065409
TAL1	0.0020418	0	0.0020418
TAL2	0.0081454	0	0
TALDO1	0.0106827	0.0106827	0
TANC1	0.0448728	0	0
TANC2	0.0167099	0	0.0167099
TANK	0.0544532	0	0.0544532
TAOK1	0.0224506	0	0.0224506
TAOK2	0.0107951	0	0
TAOK3	0.0395701	0	0
TAPBPL	0.0372765	0	0
TAPT1	0.0272498	0	0.0272498
TARBP1	0.1090034	0.1090034	0
TARBP2	0.06699	0.06699	0
TARDBP	0.0189415	0	0
TARM1	0.0008977	0	0.0008977
TARP	0.0107184	0	0.0107184
TARS	0.8715566	0	0
TARS2	0.091557	0.091557	0
TARSL2	0.0283657	0	0.0283657
TAS1R1	0.004502	0	0
TAS1R2	0.0013913	0	0
TAS2R1	0.0168841	0	0
TAS2R10	0.0132864	0.0132864	0
TAS2R13	0.0204588	0	0.0204588
TAS2R14	0.0286826	0	0

TAS2R16	0.0107342	0.0107342	0
TAS2R19	0.0259263	0	0
TAS2R20	0.0277812	0.0277812	0
TAS2R3	0.0054385	0	0
TAS2R30	0.0262818	0	0.0262818
TAS2R31	0.0136443	0	0
TAS2R38	0.0047597	0	0
TAS2R39	0.0069819	0	0
TAS2R4	0.0037195	0.0037195	0
TAS2R40	0.0142555	0	0.0142555
TAS2R41	0.0237286	0	0
TAS2R42	0.0178317	0	0
TAS2R43	0.0007027	0	0
TAS2R46	0.0222852	0	0
TAS2R5	0.0088105	0.0088105	0
TAS2R50	0.024542	0.024542	0
TAS2R60	0.0106913	0	0.0106913
TAS2R7	0.0194593	0	0
TAS2R8	0.0153436	0.0153436	0
TAS2R9	0.0164768	0	0
TASP1	0.0204542	0	0.0204542
TAT	0.0102722	0	0
TATDN1	0.1811337	0.1811337	0
TATDN2	0.0168286	0	0.0168286
TATDN3	0.2683746	0	0
TAX1BP1	0.057991	0.057991	0
TAX1BP3	0.0078103	0	0
TAZ	0.0040021	0.0040021	0
TBC1D1	0.0228534	0	0.0228534
TBC1D10A	0.0191607	0	0
TBC1D10B	0.0273632	0.0273632	0
TBC1D10C	0.0087475	0	0.0087475
TBC1D12	0.0398798	0	0.0398798
TBC1D13	0.0102691	0	0
TBC1D14	0.0313266	0	0.0313266
TBC1D15	0.4235549	0	0.4235549
TBC1D16	0.0087034	0	0.0087034
TBC1D17	0.1994046	0.1994046	0
TBC1D19	0.0213124	0	0.0213124
TBC1D2	0.6832509	0	0
TBC1D20	0.0132835	0	0.0132835
TBC1D21	0.0114381	0.0114381	0
TBC1D22A	0.0635742	0	0
TBC1D22B	0.1254406	0.1254406	0
TBC1D23	0.0985016	0	0.0985016

TBC1D24	0.0032031	0	0.0032031
TBC1D25	0.0219836	0	0
TBC1D26	9.60E-05	0	0
TBC1D28	0.0001472	0	0
TBC1D29	0.0014891	0.0014891	0
TBC1D2B	0.0379336	0	0.0379336
TBC1D3G	4.57E-05	4.57E-05	0
TBC1D3H	1.81E-05	1.81E-05	0
TBC1D3P2	6.73E-05	6.73E-05	0
TBC1D4	0.1041555	0	0.1041555
TBC1D5	0.1854725	0	0.1854725
TBC1D7	0.0584954	0	0
TBC1D8	0.0452703	0	0
TBC1D8B	0.0248056	0	0
TBC1D9	0.0247313	0	0.0247313
TBC1D9B	0.0108323	0	0.0108323
TBCA	0.0750925	0.0750925	0
TBCB	0.0207234	0.0207234	0
TBCC	0.0318678	0	0
TBCCD1	0.0952589	0	0.0952589
TBCD	0.0036537	0	0
TBCE	0.2631587	0.2631587	0
TBCEL	0.012362	0	0.012362
TBCK	0.1172689	0	0.1172689
TBK1	0.2610053	0	0.2610053
TBKBP1	0.004712	0	0.004712
TBL1X	0.1992223	0	0.1992223
TBL1XR1	0.1537837	0	0
TBL1Y	1.53E-05	0	0
TBL2	0.0098388	0.0098388	0
TBL3	0.0217136	0.0217136	0
TBP	0.0645679	0	0.0645679
TBPL1	0.0553014	0	0.0553014
TBPL2	0.4715764	0	0
TBR1	0.0114352	0	0
TBRG1	0.0232166	0	0
TBRG4	0.0183837	0.0183837	0
TBX1	0.0005459	0	0.0005459
TBX10	0.0055579	0	0
TBX15	0.0027958	0	0
TBX18	0.0044921	0	0.0044921
TBX19	0.1106261	0	0
TBX2	0.0015503	0	0.0015503
TBX20	0.0126677	0	0
TBX21	0.0057678	0	0.0057678

TBX22	0.0028294	0	0
TBX3	0.0183971	0	0.0183971
TBX4	0.0015821	0	0
TBX5	0.0009854	0	0.0009854
TBX6	0.018949	0.018949	0
TBXA2R	0.0013416	0	0.0013416
TBXAS1	0.1149592	0	0.1149592
TC2N	0.459724	0.459724	0
TCAM1P	0.0055663	0	0.0055663
TCAP	0.0008924	0.0008924	0
TCEA1	0.0668003	0	0
TCEA2	0.0116079	0	0
TCEA3	0.0023165	0.0023165	0
TCEAL1	0.0200304	0	0.0200304
TCEAL2	0.0049427	0	0.0049427
TCEAL3	0.0085961	0	0
TCEAL4	0.0083098	0	0.0083098
TCEAL5	0.0022938	0	0.0022938
TCEAL6	0.0032722	0	0.0032722
TCEAL7	0.0038189	0	0.0038189
TCEAL8	0.0126858	0	0
TCEANC	0.2008702	0	0.2008702
TCEB1	0.1918524	0.1918524	0
TCEB2	0.016266	0.016266	0
TCEB3	0.0403991	0	0.0403991
TCEB3B	0.0025121	0.0025121	0
TCERG1	0.2375154	0.2375154	0
TCERG1L	0.0011109	0	0.0011109
TCF12	0.0900964	0	0.0900964
TCF15	0.0015993	0	0.0015993
TCF21	0.0067222	0	0.0067222
TCF23	0.0022722	0	0.0022722
TCF25	0.0188282	0	0
TCF3	0.0139294	0.0139294	0
TCF4	0.0414812	0	0.0414812
TCF7	0.0195727	0	0.0195727
TCF7L1	0.0028698	0	0.0028698
TCF7L2	0.0321579	0.0321579	0
TCFL5	0.0086649	0	0
TCHH	0.0214463	0	0.0214463
TCHHL1	0.0877	0.0877	0
TCHP	0.0226906	0	0.0226906
TCIRG1	0.011198	0	0
TCL1A	0.0116189	0	0.0116189
TCL1B	0.0044951	0	0.0044951

TCL6	0.0059677	0	0.0059677
TCN1	0.1026111	0	0
TCN2	0.0218679	0	0.0218679
TCOF1	0.0429106	0.0429106	0
TCP1	0.1675106	0	0.1675106
TCP10	0.0001054	0	0
TCP10L	0.0032826	0	0
TCP10L2	0.0004442	0.0004442	0
TCP11	0.0137398	0	0
TCP11L1	0.1510963	0	0.1510963
TCP11L2	0.0790141	0	0.0790141
TCTA	0.0714549	0.0714549	0
TCTE1	0.0031756	0	0
TCTE3	0.0253443	0.0253443	0
TCTEX1D1	0.0168838	0	0.0168838
TCTEX1D2	0.0092179	0.0092179	0
TCTEX1D4	0.1661066	0	0
TCTN1	0.0528391	0	0.0528391
TCTN2	0.0096889	0.0096889	0
TCTN3	0.0507963	0	0.0507963
TDG	0.1913772	0	0
TDGF1	0.0026353	0	0.0026353
TDGF3	0.0043468	0	0.0043468
TDH	0.0064667	0	0.0064667
TDO2	0.0596561	0	0.0596561
TDP1	0.062385	0	0.062385
TDRD1	0.0378397	0	0.0378397
TDRD10	0.0147131	0	0.0147131
TDRD12	0.0020941	0	0.0020941
TDRD3	0.0779395	0	0
TDRD5	0.022727	0.022727	0
TDRD6	0.0066433	0	0.0066433
TDRD7	0.0382256	0	0
TDRD9	0.0589183	0	0.0589183
TDRG1	0.0047313	0	0
TDRKH	0.0736539	0.0736539	0
TEAD1	0.1660924	0	0.1660924
TEAD2	0.0168408	0	0.0168408
TEAD3	0.0087896	0.0087896	0
TEAD4	0.0582677	0.0582677	0
TEC	0.0907576	0	0.0907576
TECPR1	0.0055951	0.0055951	0
TECPR2	0.0231849	0	0.0231849
TECR	0.0586046	0.0586046	0
TECRL	0.0112205	0	0

TECTA	0.0062114	0	0.0062114
TECTB	0.0129666	0	0
TEDDM1	0.0588618	0.0588618	0
TEF	0.0047958	0	0
TEK	0.0140063	0	0.0140063
TEKT1	0.0101706	0.0101706	0
TEKT2	0.0067541	0	0
TEKT3	0.0201345	0	0.0201345
TEKT4	0.004364	0.004364	0
TEKT5	0.0048657	0.0048657	0
TELO2	0.0016523	0.0016523	0
TENC1	0.0542952	0	0.0542952
TEP1	0.1110682	0	0
TEPP	0.0050007	0	0.0050007
TERC	0.5326908	0	0
TERF1	0.0318765	0	0.0318765
TERF2	0.0406121	0	0.0406121
TERF2IP	0.0561218	0	0.0561218
TERT	0.0018931	0.0018931	0
TES	0.3881718	0	0
TESC	0.0080345	0.0080345	0
TESK1	0.0173545	0.0173545	0
TESK2	0.0669221	0	0.0669221
TET1	0.0060591	0	0.0060591
TET2	0.0775854	0	0.0775854
TET3	0.0229511	0	0
TEX10	0.0493511	0	0.0493511
TEX101	0.0036957	0	0
TEX11	0.0036561	0	0.0036561
TEX12	0.1135275	0	0
TEX13A	0.0018067	0.0018067	0
TEX13B	0.0013146	0.0013146	0
TEX14	0.0295525	0	0
TEX15	0.0222026	0	0.0222026
TEX19	0.0045894	0.0045894	0
TEX2	0.0334386	0	0.0334386
TEX261	0.012246	0	0
TEX264	0.0153649	0.0153649	0
TEX28	0.0002072	0	0
TEX9	0.1358358	0	0
TF	0.0017754	0	0.0017754
TFAM	0.0705352	0	0.0705352
TFAMP1	0.0048958	0	0
TFAP2A	0.3037809	0.3037809	0
TFAP2B	0.009625	0	0

TFAP2C	0.0101863	0	0
TFAP2D	0.0030832	0	0
TFAP2E	0.0068605	0	0
TFAP4	0.0151435	0.0151435	0
TFB1M	0.095159	0	0.095159
TFB2M	0.2004459	0.2004459	0
TFCP2	0.0389659	0	0
TFCP2L1	0.0018537	0.0018537	0
TFDP1	0.0061761	0	0
TFDP2	0.0647311	0	0
TFDP3	0.0034905	0.0034905	0
TFE3	0.1102345	0	0.1102345
TFEB	0.0243676	0	0.0243676
TFEC	0.025962	0	0.025962
TFF1	0.00782	0.00782	0
TFF2	0.0042139	0.0042139	0
TFF3	0.0027617	0.0027617	0
TFG	0.4235329	0.4235329	0
TFIP11	0.0190407	0	0
TFPI	0.0942496	0	0.0942496
TFPI2	0.018289	0	0.018289
TFPT	0.008951	0.008951	0
TFR2	0.0017103	0	0
TFRC	0.0763473	0	0.0763473
TG	0.0228404	0	0.0228404
TGDS	0.0467604	0.0467604	0
TGFA	0.0235878	0.0235878	0
TGFB1	0.0082078	0	0.0082078
TGFB1I1	0.0041357	0	0.0041357
TGFB2	0.0746352	0	0.0746352
TGFB3	0.4846265	0	0.4846265
TGFBI	0.3957571	0	0.3957571
TGFBR1	0.0505409	0	0.0505409
TGFBR2	0.2900324	0	0.2900324
TGFBR3	0.1041645	0	0.1041645
TGFBRAP1	0.050408	0	0.050408
TGIF1	0.7670735	0.7670735	0
TGIF2	0.0145854	0	0.0145854
TGIF2LX	0.0031697	0	0
TGIF2LY	1.53E-05	0	0
TGM1	0.0649636	0	0
TGM2	0.1736765	0	0.1736765
TGM3	0.0100434	0	0
TGM4	0.0147848	0	0
TGM5	0.0395934	0	0.0395934

TGM6	0.0036799	0	0
TGM7	0.0095611	0	0
TGOLN2	0.0464236	0	0.0464236
TGS1	0.0678777	0	0.0678777
TH	0.0004254	0.0004254	0
TH1L	0.0170343	0.0170343	0
THADA	0.0501772	0.0501772	0
THAP1	0.0564094	0	0.0564094
THAP10	0.0738309	0.0738309	0
THAP11	0.027681	0	0.027681
THAP2	0.2249641	0	0.2249641
THAP3	0.004564	0.004564	0
THAP5	0.3771996	0	0
THAP6	0.0811192	0.0811192	0
THAP7	0.0066841	0.0066841	0
THAP8	0.0655354	0	0
THAP9	0.1951018	0.1951018	0
THBD	0.0393014	0	0.0393014
THBS1	0.0413918	0	0.0413918
THBS2	0.0009356	0	0.0009356
THBS3	0.0196457	0	0.0196457
THBS4	0.0073323	0	0.0073323
THEG	0.0011613	0	0
THEM4	0.1007646	0	0
THEM5	0.0266791	0	0.0266791
THEMIS	0.0115535	0	0.0115535
THG1L	0.0575196	0.0575196	0
THNSL1	0.0353951	0	0
THNSL2	0.0068316	0	0
THOC1	0.0403798	0.0403798	0
THOC2	0.0099919	0	0
THOC3	3.77E-05	3.77E-05	0
THOC4	0.0065973	0.0065973	0
THOC5	0.0090397	0	0.0090397
THOC6	0.0181055	0.0181055	0
THOC7	0.0334514	0.0334514	0
THOP1	0.0278587	0.0278587	0
THPO	0.0045429	0	0.0045429
THRA	0.0352237	0	0
THRAP3	0.2020347	0	0.2020347
THRB	0.0385574	0	0
THRSP	0.0064381	0	0.0064381
THSD1	0.0391495	0	0.0391495
THSD4	0.0241819	0	0.0241819
THSD7A	0.0079213	0	0.0079213

THSD7B	0.0042663	0	0.0042663
THTPA	0.021776	0	0.021776
THUMPD1	0.0302705	0	0.0302705
THUMPD2	0.0378203	0.0378203	0
THUMPD3	0.0578428	0.0578428	0
THY1	0.0051396	0	0.0051396
THYN1	0.0138737	0.0138737	0
TIA1	0.0674387	0	0
TIAF1	0.0117042	0.0117042	0
TIAL1	0.1187453	0.1187453	0
TIAM1	0.0068211	0	0.0068211
TIAM2	0.0225297	0	0.0225297
TICAM1	0.0600929	0.0600929	0
TICAM2	0.0461691	0	0.0461691
TIE1	0.0087186	0	0.0087186
TIFA	0.1529462	0	0.1529462
TIFAB	0.0204795	0	0.0204795
TIGD1	0.0173414	0.0173414	0
TIGD2	0.0352662	0.0352662	0
TIGD3	0.0070369	0.0070369	0
TIGD4	0.0584362	0.0584362	0
TIGD5	0.01686	0.01686	0
TIGD6	0.0937093	0.0937093	0
TIGD7	0.0130481	0	0.0130481
TIGIT	0.013687	0	0.013687
TIMD4	0.007212	0	0.007212
TIMELESS	0.023296	0	0
TIMM10	0.0540641	0	0
TIMM13	0.0021786	0.0021786	0
TIMM16	0.0050525	0.0050525	0
TIMM17A	0.1014824	0.1014824	0
TIMM17B	0.0179257	0.0179257	0
TIMM22	0.0169627	0	0.0169627
TIMM44	0.0097557	0.0097557	0
TIMM50	0.0066311	0.0066311	0
TIMM8A	0.0142814	0	0
TIMM8B	0.0434787	0.0434787	0
TIMM9	0.7489308	0.7489308	0
TIMP1	0.1061245	0	0.1061245
TIMP2	0.0057661	0	0.0057661
TIMP3	0.0075894	0	0.0075894
TIMP4	0.0182799	0	0.0182799
TINAG	0.0151792	0	0
TINAGL1	0.0190749	0.0190749	0
TINF2	0.1689189	0	0.1689189

TIPARP	0.7037787	0	0.7037787
TIPIN	0.0277461	0	0
TIPRL	0.2834746	0	0
TIRAP	0.0098628	0	0
TJAP1	0.0268681	0.0268681	0
TJP1	0.033995	0	0
TJP2	0.7605675	0.7605675	0
TJP3	0.0068182	0.0068182	0
TK1	0.0301608	0.0301608	0
TK2	0.047242	0	0
TKT	0.0230746	0.0230746	0
TKTL1	0.002306	0	0.002306
TKTL2	0.0065088	0.0065088	0
TLCD1	0.4169431	0.4169431	0
TLE1	0.0228239	0.0228239	0
TLE2	0.0124639	0.0124639	0
TLE3	0.2672427	0	0
TLE4	0.0777898	0	0.0777898
TLE6	0.0144689	0.0144689	0
TLK1	0.073055	0	0
TLK2	0.0062851	0	0
TLL1	0.016086	0	0.016086
TLL2	0.0679609	0	0
TLN1	0.094777	0	0.094777
TLN2	0.0211717	0	0.0211717
TLR1	0.0862506	0	0.0862506
TLR10	0.0699494	0	0.0699494
TLR2	0.0496719	0	0.0496719
TLR3	0.0192957	0	0.0192957
TLR4	0.1037058	0	0.1037058
TLR5	0.373898	0	0.373898
TLR6	0.0982262	0	0.0982262
TLR7	0.0132901	0	0.0132901
TLR8	0.0104635	0	0.0104635
TLR9	0.0275928	0	0.0275928
TLX1	0.0010386	0.0010386	0
TLX1NB	0.0010774	0.0010774	0
TLX2	0.0068988	0	0
TLX3	0.0028067	0	0
TM2D1	0.0428652	0	0
TM2D2	0.4258354	0	0.4258354
TM2D3	0.0702039	0	0
TM4SF1	1	1	0
TM4SF18	0.2115715	0	0.2115715
TM4SF19	0.0285221	0	0.0285221

TM4SF20	0.9366455	0	0
TM4SF4	0.1159416	0	0
TM4SF5	0.0035454	0.0035454	0
TM6SF1	0.0092136	0	0.0092136
TM6SF2	0.0010693	0	0
TM7SF2	0.0270248	0.0270248	0
TM7SF3	0.1747054	0.1747054	0
TM7SF4	0.0150938	0	0.0150938
TM9SF1	0.0395089	0.0395089	0
TM9SF2	0.1521397	0	0
TM9SF3	0.1132085	0.1132085	0
TM9SF4	0.1063411	0.1063411	0
TMBIM1	0.1779857	0.1779857	0
TMBIM4	0.0967952	0.0967952	0
TMBIM6	0.1478819	0.1478819	0
TMC1	0.1774791	0	0.1774791
TMC2	0.0018012	0	0.0018012
TMC3	0.0053394	0	0.0053394
TMC4	0.0012987	0.0012987	0
TMC5	0.2433935	0.2433935	0
TMC6	0.0235983	0	0
TMC7	0.0040176	0.0040176	0
TMC8	0.0203682	0	0.0203682
TMCC1	0.059096	0	0
TMCC2	0.0894645	0	0.0894645
TMCC3	0.0714633	0	0.0714633
TMCO1	0.1284734	0.1284734	0
TMCO2	0.0415536	0.0415536	0
TMCO3	0.0499577	0.0499577	0
TMCO4	0.0123347	0.0123347	0
TMCO5A	0.0072689	0	0
TMCO6	0.2543004	0.2543004	0
TMCO7	0.016151	0	0.016151
TMED1	0.0236003	0.0236003	0
TMED10	0.1239633	0.1239633	0
TMED2	0.0568101	0.0568101	0
TMED3	0.0155792	0.0155792	0
TMED4	0.0684907	0.0684907	0
TMED5	0.1743646	0	0.1743646
TMED6	0.0090149	0	0
TMED7	0.1063206	0	0
TMED7-TICAM	0.1063206	0	0.1063206
TMED8	0.0517908	0	0.0517908
TMED9	0.0291616	0.0291616	0
TMEFF1	0.0065662	0	0.0065662

TMEFF2	0.0131721	0	0.0131721
TMEM100	0.0389048	0	0.0389048
TMEM101	0.0457311	0.0457311	0
TMEM102	0.0871299	0.0871299	0
TMEM104	0.016379	0	0
TMEM105	0.0087112	0.0087112	0
TMEM106B	0.0695507	0	0
TMEM106C	0.0271854	0.0271854	0
TMEM107	0.1948209	0	0
TMEM108	0.0099921	0	0.0099921
TMEM109	0.2085082	0	0.2085082
TMEM11	0.0180143	0.0180143	0
TMEM110	0.0241498	0	0.0241498
TMEM111	0.149161	0.149161	0
TMEM114	0.0183693	0.0183693	0
TMEM115	0.0189865	0.0189865	0
TMEM116	0.1141832	0.1141832	0
TMEM117	0.0352832	0	0.0352832
TMEM119	0.0023984	0	0.0023984
TMEM120A	0.004255	0.004255	0
TMEM120B	0.0447721	0.0447721	0
TMEM121	0.0012109	0	0
TMEM123	0.0918324	0	0
TMEM125	0.0026176	0.0026176	0
TMEM126A	0.1561247	0.1561247	0
TMEM126B	0.4171618	0	0.4171618
TMEM127	0.036421	0	0.036421
TMEM128	0.018463	0.018463	0
TMEM129	0.0152702	0.0152702	0
TMEM130	0.0046022	0	0.0046022
TMEM131	0.059823	0	0.059823
TMEM132A	0.0609989	0	0
TMEM132B	0.0031344	0	0.0031344
TMEM132C	0.0014344	0	0.0014344
TMEM132D	0.0030844	0	0.0030844
TMEM132E	0.0006546	0	0.0006546
TMEM133	0.0148527	0	0
TMEM134	0.0267441	0.0267441	0
TMEM135	0.0419634	0	0
TMEM136	0.0521025	0	0
TMEM138	0.1165316	0	0.1165316
TMEM139	0.0285758	0.0285758	0
TMEM140	0.2869568	0	0.2869568
TMEM141	0.0322638	0.0322638	0
TMEM143	0.0116124	0.0116124	0

TMEM144	0.0089037	0	0
TMEM145	0.0013455	0	0
TMEM146	0.0175141	0	0
TMEM147	0.0086594	0.0086594	0
TMEM149	0.1757052	0	0.1757052
TMEM14A	0.0612564	0.0612564	0
TMEM14B	0.0450418	0	0
TMEM14C	0.0345194	0	0
TMEM14E	0.1571755	0	0.1571755
TMEM150A	0.0276926	0	0
TMEM150B	0.0040934	0	0.0040934
TMEM150C	0.003144	0	0.003144
TMEM151A	0.0049327	0.0049327	0
TMEM151B	0.0140225	0	0
TMEM154	0.0979873	0	0.0979873
TMEM155	0.0132748	0	0.0132748
TMEM156	0.2620917	0	0.2620917
TMEM158	0.0165259	0	0.0165259
TMEM159	0.0239944	0.0239944	0
TMEM160	0.0043983	0.0043983	0
TMEM161A	0.0097579	0.0097579	0
TMEM161B	0.0534295	0.0534295	0
TMEM163	0.0054731	0	0
TMEM164	0.0242088	0.0242088	0
TMEM165	0.1395299	0	0
TMEM167A	0.1034049	0	0.1034049
TMEM168	0.0772088	0	0
TMEM169	0.0140196	0	0.0140196
TMEM17	0.0225709	0.0225709	0
TMEM170A	0.032033	0	0
TMEM170B	0.0397686	0	0.0397686
TMEM171	0.0076052	0.0076052	0
TMEM173	0.4100121	0	0.4100121
TMEM174	0.0099579	0	0
TMEM175	0.0207356	0.0207356	0
TMEM176A	0.0015505	0	0
TMEM176B	0.0015563	0	0
TMEM177	0.0048324	0.0048324	0
TMEM178	0.0067869	0.0067869	0
TMEM179	0.0005395	0	0
TMEM179B	0.0286861	0.0286861	0
TMEM18	0.0372618	0.0372618	0
TMEM180	0.0160231	0.0160231	0
TMEM181	0.0035615	0	0
TMEM182	0.013046	0.013046	0

TMEM183A	0.1373746	0.1373746	0
TMEM184A	0.0053875	0.0053875	0
TMEM184B	0.005556	0.005556	0
TMEM184C	0.0369762	0	0
TMEM185A	0.0058951	0	0.0058951
TMEM185B	0.0398986	0	0
TMEM186	0.0409183	0.0409183	0
TMEM187	0.0107518	0.0107518	0
TMEM188	0.0321254	0	0.0321254
TMEM189	0.0517704	0.0517704	0
TMEM189-UB	0.0517704	0	0
TMEM19	0.0925312	0	0.0925312
TMEM190	0.068734	0.068734	0
TMEM191A	0.0009186	0.0009186	0
TMEM192	0.0157323	0	0
TMEM194A	0.1030564	0	0.1030564
TMEM194B	0.1308308	0	0.1308308
TMEM195	0.0098471	0	0
TMEM196	0.0075185	0	0.0075185
TMEM198	0.0023085	0.0023085	0
TMEM199	0.0188741	0.0188741	0
TMEM2	0.2550797	0	0.2550797
TMEM20	0.0272294	0	0
TMEM200A	0.011654	0	0.011654
TMEM200B	0.0015985	0	0.0015985
TMEM200C	0.0061465	0	0.0061465
TMEM201	0.0038583	0	0
TMEM202	0.0067657	0	0
TMEM203	0.0173014	0	0
TMEM204	0.00292	0	0.00292
TMEM205	0.0485811	0.0485811	0
TMEM206	0.059989	0	0.059989
TMEM207	0.111634	0	0
TMEM208	0.0115877	0.0115877	0
TMEM209	0.1435695	0	0
TMEM211	0.0043953	0.0043953	0
TMEM212	0.1241326	0	0
TMEM213	0.0032014	0	0
TMEM214	0.0116941	0.0116941	0
TMEM215	0.0091566	0	0.0091566
TMEM216	0.0297684	0.0297684	0
TMEM217	0.1031891	0	0.1031891
TMEM218	0.0181665	0	0
TMEM219	0.0074154	0.0074154	0
TMEM22	0.0305144	0	0.0305144

TMEM220	0.0045374	0	0.0045374
TMEM222	0.0361136	0.0361136	0
TMEM223	0.0299323	0.0299323	0
TMEM225	0.0062534	0	0
TMEM229A	0.0038844	0	0
TMEM229B	0.0095935	0	0
TMEM231	0.0648932	0	0
TMEM232	0.0115117	0	0
TMEM233	0.0076403	0	0.0076403
TMEM25	0.0061275	0	0
TMEM26	0.0147186	0	0.0147186
TMEM27	0.0176283	0	0.0176283
TMEM30A	0.299266	0	0.299266
TMEM30B	0.0087382	0.0087382	0
TMEM30C	0.0130699	0	0
TMEM31	0.004334	0	0.004334
TMEM33	0.1964419	0.1964419	0
TMEM35	0.0023201	0	0.0023201
TMEM37	0.0217981	0	0
TMEM38A	0.0140062	0.0140062	0
TMEM38B	0.05344	0	0.05344
TMEM39A	0.0975364	0	0.0975364
TMEM39B	0.0567279	0	0.0567279
TMEM40	0.0273022	0	0
TMEM41A	0.0464789	0.0464789	0
TMEM41B	0.059825	0.059825	0
TMEM42	0.01658	0.01658	0
TMEM43	0.0732291	0	0.0732291
TMEM44	0.0069552	0.0069552	0
TMEM45A	0.0421004	0	0.0421004
TMEM45B	0.0146238	0.0146238	0
TMEM47	0.0109692	0	0.0109692
TMEM48	0.0581589	0	0
TMEM49	0.098402	0.098402	0
TMEM5	0.0667343	0	0
TMEM50A	0.0075363	0	0
TMEM50B	0.0175949	0	0
TMEM51	0.0776165	0	0
TMEM52	0.0009205	0	0
TMEM53	0.2279064	0.2279064	0
TMEM54	0.0053893	0.0053893	0
TMEM55A	0.0650106	0	0.0650106
TMEM55B	0.2214215	0.2214215	0
TMEM56	0.0222655	0.0222655	0
TMEM57	0.0544328	0	0

TMEM59	0.1424332	0.1424332	0
TMEM59L	0.0006799	0	0.0006799
TMEM60	0.0864651	0	0
TMEM61	0.0178797	0.0178797	0
TMEM62	0.0513296	0.0513296	0
TMEM63A	0.0244609	0.0244609	0
TMEM63B	0.0259558	0.0259558	0
TMEM63C	0.0059139	0	0.0059139
TMEM64	0.060079	0	0.060079
TMEM65	0.091896	0	0
TMEM66	0.0818636	0	0.0818636
TMEM67	0.0358633	0	0
TMEM68	0.0764624	0.0764624	0
TMEM69	0.1826693	0	0
TMEM70	0.1150754	0	0.1150754
TMEM71	0.0453866	0	0.0453866
TMEM72	0.0038977	0	0.0038977
TMEM74	0.0335172	0	0.0335172
TMEM79	0.1068779	0.1068779	0
TMEM80	0.0079798	0.0079798	0
TMEM81	0.0558758	0.0558758	0
TMEM82	0.0012103	0.0012103	0
TMEM84	0.0842154	0	0.0842154
TMEM85	0.0507247	0.0507247	0
TMEM86A	0.0075302	0	0.0075302
TMEM86B	0.0015577	0.0015577	0
TMEM87A	0.3665319	0	0
TMEM87B	0.0811254	0	0
TMEM88	0.0346787	0	0.0346787
TMEM88B	0.0010504	0.0010504	0
TMEM89	0.0117115	0.0117115	0
TMEM8A	0.0022679	0.0022679	0
TMEM8B	0.0161461	0.0161461	0
TMEM8C	0.0011274	0	0.0011274
TMEM9	0.0525414	0.0525414	0
TMEM90A	0.0029172	0	0.0029172
TMEM90B	0.0011168	0	0.0011168
TMEM91	0.1806676	0	0
TMEM92	0.0143829	0.0143829	0
TMEM93	0.0081383	0.0081383	0
TMEM95	0.0196288	0	0
TMEM97	0.0330271	0.0330271	0
TMEM98	0.0038761	0	0
TMEM99	0.2988097	0.2988097	0
TMEM9B	0.0583012	0.0583012	0

TMF1	0.0668144	0	0.0668144
TMIE	0.0029107	0	0
TMIGD1	0.0571333	0.0571333	0
TMIGD2	0.000907	0	0.000907
TMLHE	0.0163481	0	0
TMOD1	0.0629271	0	0.0629271
TMOD2	0.0204755	0	0.0204755
TMOD3	0.2083237	0	0
TMOD4	0.0196716	0.0196716	0
TMPO	0.0713406	0	0
TMPPE	0.0272886	0	0
TMPRSS11A	0.0017177	0	0
TMPRSS11B	0.0071259	0	0
TMPRSS11BN	0.0073106	0	0
TMPRSS11D	0.2996496	0	0
TMPRSS11F	0.0097291	0	0
TMPRSS12	0.016349	0	0
TMPRSS13	0.005383	0	0
TMPRSS2	0.0024378	0.0024378	0
TMPRSS3	0.1680934	0.1680934	0
TMPRSS4	0.0552341	0.0552341	0
TMPRSS5	0.0023867	0.0023867	0
TMPRSS6	0.0015913	0	0
TMPRSS7	0.0217231	0.0217231	0
TMPRSS9	0.008672	0.008672	0
TMSB10	0.1853582	0	0
TMSB15A	0.0023436	0	0.0023436
TMSB15B	0.0051121	0	0.0051121
TMSB4Y	1.53E-05	1.53E-05	0
TMTC1	0.2601727	0	0.2601727
TMTC2	0.1590042	0	0
TMTC3	0.1178824	0	0.1178824
TMTC4	0.0193217	0.0193217	0
TMUB1	0.0101796	0.0101796	0
TMUB2	0.0261835	0.0261835	0
TMX1	0.0881758	0	0.0881758
TMX2	0.0245562	0.0245562	0
TMX3	0.136672	0	0.136672
TMX4	0.0386457	0	0.0386457
TNC	0.2328019	0	0.2328019
TNFAIP1	0.7125147	0	0.7125147
TNFAIP2	0.0100842	0	0.0100842
TNFAIP3	0.4717649	0	0.4717649
TNFAIP6	0.0360205	0	0.0360205
TNFAIP8	0.8615758	0	0.8615758

TNFAIP8L1	0.0058573	0	0.0058573
TNFAIP8L2	0.0882744	0	0.0882744
TNFAIP8L3	0.0167863	0	0.0167863
TNFRSF10A	0.0845198	0	0
TNFRSF10B	0.261717	0.261717	0
TNFRSF10C	0.0147483	0.0147483	0
TNFRSF10D	0.2098521	0	0
TNFRSF11A	0.0176492	0.0176492	0
TNFRSF11B	0.1879657	0	0.1879657
TNFRSF12A	0.0093944	0.0093944	0
TNFRSF13B	0.164229	0	0.164229
TNFRSF13C	0.0135953	0	0.0135953
TNFRSF14	0.0079173	0.0079173	0
TNFRSF17	0.0605062	0	0.0605062
TNFRSF18	0.0006868	0	0.0006868
TNFRSF19	0.0355991	0	0.0355991
TNFRSF1A	1	0	0
TNFRSF1B	0.0288029	0	0.0288029
TNFRSF21	0.0400931	0.0400931	0
TNFRSF25	0.0105302	0	0
TNFRSF4	0.0015787	0	0.0015787
TNFRSF6B	0.2583999	0	0
TNFRSF8	0.2174704	0	0.2174704
TNFRSF9	0.0686894	0	0.0686894
TNFSF10	0.2117165	0	0.2117165
TNFSF11	0.0183482	0	0.0183482
TNFSF12	0.0200892	0	0.0200892
TNFSF12-TNF	0.0199724	0	0.0199724
TNFSF13	0.1651712	0	0
TNFSF13B	0.0290596	0	0.0290596
TNFSF14	0.0526254	0	0.0526254
TNFSF15	0.03533	0	0.03533
TNFSF18	0.26722	0	0.26722
TNFSF4	0.0989011	0	0.0989011
TNFSF8	0.016937	0	0.016937
TNFSF9	0.1669003	0	0
TNIK	0.0834541	0	0
TNIP1	0.8816648	0	0
TNIP2	0.046327	0.046327	0
TNIP3	0.3463344	0	0.3463344
TNK1	0.0016117	0.0016117	0
TNK2	0.042873	0.042873	0
TNKS	0.0521007	0	0.0521007
TNKS1BP1	0.0946127	0.0946127	0
TNKS2	0.1299222	0	0.1299222

TNMD	0.0035414	0	0.0035414
TNN	0.0137193	0	0.0137193
TNNC1	0.0060119	0	0.0060119
TNNC2	0.0105061	0	0
TNNI1	0.0098922	0.0098922	0
TNNI2	0.0027773	0.0027773	0
TNNI3	0.012796	0.012796	0
TNNI3K	0.0359339	0	0
TNNT1	0.0033534	0.0033534	0
TNNT2	0.0117858	0.0117858	0
TNNT3	0.0005759	0	0.0005759
TNP1	0.0039305	0.0039305	0
TNP2	0.0031906	0	0
TNPO1	0.5350578	0	0.5350578
TNPO2	0.0350806	0	0
TNPO3	0.1532152	0	0
TNR	0.0104665	0	0.0104665
TNRC18	0.0226864	0.0226864	0
TNRC6A	0.0541661	0	0.0541661
TNRC6B	0.210451	0	0.210451
TNRC6C	0.0166395	0	0.0166395
TNS1	0.001004	0	0.001004
TNS3	0.0022675	0	0.0022675
TNS4	0.1770774	0	0
TOB1	0.0954083	0.0954083	0
TOB2	0.0581249	0	0
TOE1	0.0429009	0	0
TOLLIP	0.0038172	0.0038172	0
TOM1	0.1006471	0	0.1006471
TOM1L1	0.0308581	0.0308581	0
TOM1L2	0.0393014	0	0
TOMM20	0.1500334	0.1500334	0
TOMM20L	0.00818	0.00818	0
TOMM22	0.0179075	0.0179075	0
TOMM34	0.1243277	0.1243277	0
TOMM40	0.0129319	0.0129319	0
TOMM40L	0.0638674	0	0.0638674
TOMM5	0.0770512	0.0770512	0
TOMM6	0.0328838	0.0328838	0
TOMM7	0.0541701	0.0541701	0
TOMM70A	0.1120656	0	0
TOP1	0.5130145	0	0
TOP1MT	0.0084231	0.0084231	0
TOP1P1	0.0277322	0.0277322	0
TOP1P2	0.003573	0.003573	0

TOP2A	0.1322374	0.1322374	0
TOP2B	0.090128	0	0.090128
TOP3A	0.0905024	0	0.0905024
TOP3B	0.00547	0.00547	0
TOPBP1	0.0499836	0	0.0499836
TOPORS	0.1020224	0	0.1020224
TOR1A	0.0319089	0	0.0319089
TOR1AIP1	0.4048538	0	0.4048538
TOR1AIP2	0.4365563	0	0.4365563
TOR1B	0.0101161	0	0
TOR2A	0.0329138	0.0329138	0
TOR3A	0.1023722	0	0
TOX	0.0214142	0	0.0214142
TOX2	0.1151583	0	0.1151583
TOX3	0.0067369	0.0067369	0
TOX4	0.0820142	0	0
TP53	0.0938329	0	0.0938329
TP53AIP1	0.001712	0.001712	0
TP53BP1	0.0506533	0	0.0506533
TP53BP2	0.1394204	0	0
TP53I11	0.0020592	0.0020592	0
TP53I13	0.0060147	0.0060147	0
TP53I3	0.1238326	0.1238326	0
TP53INP1	0.0256107	0	0.0256107
TP53INP2	0.0406306	0	0.0406306
TP53RK	0.0804289	0	0
TP53TG1	0.0708243	0.0708243	0
TP53TG5	0.0242642	0	0
TP63	0.3835837	0	0.3835837
TP73	0.013884	0.013884	0
TPBG	0.0567673	0	0
TPCN1	0.1577184	0	0
TPCN2	0.0186647	0	0
TPD52	0.1054618	0.1054618	0
TPD52L1	0.0376813	0.0376813	0
TPD52L2	0.0460489	0.0460489	0
TPD52L3	0.0089561	0	0.0089561
TPH1	0.0115352	0	0
TPH2	0.0456531	0	0.0456531
TPI1	0.0139588	0.0139588	0
TPK1	0.6035967	0	0.6035967
TPM1	0.1223869	0	0.1223869
TPM2	0.0099128	0	0.0099128
TPM3	0.170518	0	0
TPM4	0.7867262	0	0.7867262

TPMT	0.0905158	0.0905158	0
TPO	0.0032278	0	0.0032278
TPP1	0.0545459	0	0.0545459
TPP2	0.1181495	0	0.1181495
TPPP	0.0004553	0	0
TPPP2	0.0031639	0	0
TPPP3	0.0017048	0	0
TPR	0.4905132	0	0
TPRA1	0.0674306	0.0674306	0
TPRG1	0.0159331	0	0.0159331
TPRG1L	0.0059649	0.0059649	0
TPRKB	0.0289159	0	0
TPRN	0.0175678	0.0175678	0
TPRX1	0.0011094	0	0
TPRXL	0.0023582	0	0.0023582
TPSAB1	9.49E-05	0	9.49E-05
TPSB2	8.90E-05	0	8.90E-05
TPSD1	0.0001777	0	0.0001777
TPSG1	0.0001189	0	0
TPST1	0.0098503	0	0.0098503
TPST2	0.0144835	0	0.0144835
TPT1	0.289475	0	0
TPTE	0.0039828	0	0
TPTE2	0.0028775	0	0.0028775
TPTE2P1	0.0004876	0	0
TPX2	0.1238174	0.1238174	0
TRA2A	0.0596483	0	0
TRA2B	0.2562687	0	0.2562687
TRABD	0.0028455	0.0028455	0
TRADD	0.0251246	0.0251246	0
TRAF1	0.2985345	0	0.2985345
TRAF2	0.0199745	0.0199745	0
TRAF3	0.0459235	0	0.0459235
TRAF3IP1	0.0055078	0	0
TRAF3IP2	0.5348958	0	0
TRAF3IP3	0.1566302	0	0.1566302
TRAF4	0.1746743	0.1746743	0
TRAF5	0.163081	0	0.163081
TRAF6	0.0323079	0	0.0323079
TRAF7	0.0456072	0.0456072	0
TRAFD1	0.22092	0	0.22092
TRAIP	0.0065061	0.0065061	0
TRAK1	0.1079438	0.1079438	0
TRAK2	0.0282205	0	0.0282205
TRAM1	0.0958175	0	0.0958175

TRAM1L1	0.0058018	0	0.0058018
TRAM2	0.0293936	0	0.0293936
TRANK1	0.0059178	0	0.0059178
TRAP1	0.0143639	0.0143639	0
TRAPPC1	0.0123165	0	0
TRAPPC10	0.0266858	0	0.0266858
TRAPPC2	0.0584778	0.0584778	0
TRAPPC2L	0.0020187	0.0020187	0
TRAPPC2P1	0.0171795	0	0
TRAPPC3	0.0905317	0	0
TRAPPC4	0.0531212	0.0531212	0
TRAPPC5	0.0111001	0.0111001	0
TRAPPC6A	0.0362543	0.0362543	0
TRAPPC6B	0.1235135	0	0
TRAPPC9	0.1358958	0.1358958	0
TRAT1	0.0090777	0	0.0090777
TRDMT1	0.0490548	0	0.0490548
TRDN	0.0077608	0	0.0077608
TREH	0.0029108	0	0
TREM1	0.0474068	0	0.0474068
TREM2	0.0027568	0	0.0027568
TREML1	0.0032971	0	0.0032971
TREML2	0.0093546	0	0.0093546
TREML3	0.0075781	0	0.0075781
TREML4	0.0063681	0	0.0063681
TRERF1	0.0331967	0	0
TREX1	0.0267309	0.0267309	0
TREX2	0.0018579	0	0
TRH	0.0021352	0	0.0021352
TRHDE	0.0108035	0	0
TRHR	0.025961	0	0.025961
TRIAP1	0.0470162	0	0
TRIB1	0.6440974	0	0
TRIB2	0.0949147	0	0.0949147
TRIB3	0.0489842	0.0489842	0
TRIL	0.0042051	0	0.0042051
TRIM11	0.0094929	0.0094929	0
TRIM13	0.0323003	0	0.0323003
TRIM14	0.0171415	0	0
TRIM16	0.0049836	0.0049836	0
TRIM16L	0.0007826	0.0007826	0
TRIM17	0.0194889	0	0
TRIM2	0.0636266	0.0636266	0
TRIM21	0.0095936	0	0.0095936
TRIM22	0.0956149	0	0.0956149

TRIM23	0.0920017	0	0.0920017
TRIM24	0.0286353	0	0
TRIM25	1	0	0
TRIM28	0.0084763	0.0084763	0
TRIM29	0.0329923	0	0
TRIM3	0.0296507	0	0
TRIM32	0.0420793	0	0.0420793
TRIM33	0.0946996	0	0.0946996
TRIM34	0.074543	0	0.074543
TRIM35	0.1164578	0	0.1164578
TRIM36	0.0094775	0.0094775	0
TRIM37	0.1142104	0	0.1142104
TRIM38	0.1453024	0	0.1453024
TRIM4	0.0368745	0	0
TRIM41	0.2269266	0.2269266	0
TRIM42	0.0049398	0.0049398	0
TRIM43	0.0001985	0	0
TRIM44	0.1306224	0	0.1306224
TRIM45	0.0358959	0.0358959	0
TRIM46	0.0470918	0.0470918	0
TRIM47	0.14102	0.14102	0
TRIM48	0.3013184	0	0
TRIM49	7.33E-05	0	0
TRIM5	0.0584174	0	0.0584174
TRIM50	0.001675	0	0.001675
TRIM52	0.0424911	0.0424911	0
TRIM54	0.0141075	0.0141075	0
TRIM55	0.1895866	0	0.1895866
TRIM56	0.025756	0	0
TRIM58	0.0073278	0	0.0073278
TRIM59	0.075234	0	0.075234
TRIM6	0.0094201	0	0.0094201
TRIM60	0.0019529	0	0
TRIM61	0.0042914	0	0.0042914
TRIM62	0.0073578	0	0.0073578
TRIM63	0.0028468	0	0.0028468
TRIM64	1.74E-05	0	0
TRIM65	0.0053996	0	0
TRIM66	0.0099564	0	0
TRIM67	0.012649	0	0
TRIM68	0.0549797	0.0549797	0
TRIM69	1	0	1
TRIM6-TRIM3	0.0094128	0.0094128	0
TRIM7	0.0887638	0.0887638	0
TRIM71	0.0014664	0	0.0014664

TRIM72	0.0035401	0	0
TRIM74	1.59E-05	0	0
TRIM77	0.0059398	0	0
TRIM78P	0.0150936	0	0
TRIM8	0.0424536	0.0424536	0
TRIM9	0.0164846	0	0.0164846
TRIML1	0.0020682	0	0
TRIML2	0.0199687	0	0
TRIO	0.0915901	0	0.0915901
TRIOBP	0.0177086	0.0177086	0
TRIP10	0.1214727	0.1214727	0
TRIP11	0.0314743	0	0.0314743
TRIP12	0.0325839	0	0.0325839
TRIP13	0.0136839	0.0136839	0
TRIP4	0.0806651	0	0
TRIP6	0.0139406	0.0139406	0
TRIT1	0.059338	0.059338	0
TRMT1	0.0337633	0.0337633	0
TRMT11	0.1139777	0	0
TRMT112	0.0682073	0.0682073	0
TRMT12	0.1154648	0	0
TRMT2A	0.0100864	0.0100864	0
TRMT2B	0.0043562	0	0
TRMT5	0.0824711	0.0824711	0
TRMT6	0.1945618	0.1945618	0
TRMT61A	0.0025446	0.0025446	0
TRMT61B	0.0369652	0	0
TRMU	0.0053108	0.0053108	0
TRNAU1AP	0.0456946	0	0
TRNP1	0.0085444	0.0085444	0
TRNT1	0.0283813	0	0
TRO	0.0013262	0	0.0013262
TROAP	0.0188115	0.0188115	0
TROVE2	0.3102032	0	0.3102032
TRPA1	0.011052	0	0.011052
TRPC1	0.0133118	0	0.0133118
TRPC2	0.0027497	0	0.0027497
TRPC3	0.0112575	0	0.0112575
TRPC4	0.0064171	0	0.0064171
TRPC4AP	0.1078361	0.1078361	0
TRPC5	0.0045454	0	0
TRPC6	0.0106595	0	0.0106595
TRPC7	0.0235031	0	0
TRPM1	0.0046562	0	0.0046562
TRPM2	0.0026971	0	0.0026971

TRPM3	0.0106404	0	0.0106404
TRPM4	0.0025004	0.0025004	0
TRPM5	0.0004809	0.0004809	0
TRPM6	0.0271511	0	0.0271511
TRPM7	0.0713796	0	0.0713796
TRPM8	0.0079021	0	0.0079021
TRPS1	0.1055556	0	0.1055556
TRPT1	0.0250674	0.0250674	0
TRPV1	0.0025718	0.0025718	0
TRPV2	0.0518321	0	0.0518321
TRPV3	0.0121153	0	0
TRPV4	0.009767	0	0
TRPV5	0.0021154	0	0
TRPV6	0.0044041	0	0
TRRAP	0.0106175	0	0
TRUB1	0.0752972	0	0.0752972
TRUB2	0.0685692	0.0685692	0
TRY6	0.0032	0	0
TRYX3	0.0062789	0	0
TSC1	0.0521653	0	0
TSC2	0.0020917	0.0020917	0
TSC22D1	0.0910987	0.0910987	0
TSC22D2	0.2045082	0	0.2045082
TSC22D3	0.0691981	0	0.0691981
TSC22D4	0.0147572	0.0147572	0
TSEN15	0.1635148	0.1635148	0
TSEN2	0.0549834	0.0549834	0
TSEN34	0.0946654	0.0946654	0
TSEN54	0.0108947	0.0108947	0
TSFM	0.0544002	0.0544002	0
TSG1	0.0088137	0.0088137	0
TSG101	0.1780339	0.1780339	0
TSGA10	0.0816278	0.0816278	0
TSGA10IP	0.0031986	0	0.0031986
TSGA13	0.0115091	0.0115091	0
TSGA14	0.0298815	0	0
TSHB	0.0142231	0	0.0142231
TSHR	0.0289705	0	0.0289705
TSHZ1	0.1108718	0	0.1108718
TSHZ2	0.063947	0	0.063947
TSHZ3	0.000683	0	0.000683
TSIX	0.0048737	0	0
TSKS	0.2087929	0	0.2087929
TSKU	0.0602837	0	0.0602837
TSLP	0.1633831	0	0.1633831

TSN	0.0193805	0	0
TSNARE1	0.0037529	0.0037529	0
TSNAX	0.1603154	0	0
TSNAX-DISC1	0.1603154	0	0
TSNAXIP1	0.0230523	0	0
TSPAN1	0.1403183	0.1403183	0
TSPAN10	0.0034121	0	0
TSPAN11	0.0047485	0	0.0047485
TSPAN12	0.0822329	0.0822329	0
TSPAN13	0.015223	0.015223	0
TSPAN14	0.0397249	0	0.0397249
TSPAN15	0.1425633	0.1425633	0
TSPAN16	0.0100334	0	0
TSPAN17	0.0101457	0.0101457	0
TSPAN18	0.0112841	0	0.0112841
TSPAN19	0.0390271	0	0
TSPAN2	0.0093729	0	0.0093729
TSPAN3	0.0821681	0.0821681	0
TSPAN31	0.1264097	0.1264097	0
TSPAN32	0.0002815	0	0.0002815
TSPAN33	0.0325518	0	0.0325518
TSPAN4	0.0095362	0	0.0095362
TSPAN5	0.0981368	0	0.0981368
TSPAN6	0.0226573	0	0
TSPAN7	0.0176687	0	0.0176687
TSPAN8	0.0941414	0.0941414	0
TSPAN9	0.0063104	0	0.0063104
TSPO	0.0056187	0.0056187	0
TSPO2	0.0297875	0.0297875	0
TSPY1	1.53E-05	0	0
TSPY2	1.53E-05	0	0
TSPYL1	0.0773129	0	0.0773129
TSPYL2	0.010241	0	0.010241
TSPYL3	0.0093636	0	0.0093636
TSPYL4	0.0890655	0	0.0890655
TSPYL5	0.006243	0	0.006243
TSPYL6	0.0088837	0	0
TSR1	0.0186502	0	0.0186502
TSR2	0.0117671	0.0117671	0
TSSC1	0.0224011	0.0224011	0
TSSC4	0.0046309	0.0046309	0
TSSK1B	0.0042578	0	0
TSSK2	0.0013151	0	0
TSSK3	0.0036663	0	0
TSSK4	0.0211925	0	0

TSSK6	0.0084624	0.0084624	0
TST	0.0021713	0.0021713	0
TSTA3	0.0812468	0.0812468	0
TSTD1	0.0957247	0.0957247	0
TSTD2	0.2030467	0	0.2030467
TTBK1	0.0059466	0	0
TTBK2	0.294256	0	0.294256
TTC1	0.1091535	0	0
TTC12	0.0165314	0.0165314	0
TTC13	0.134243	0.134243	0
TTC14	0.1259442	0	0
TTC15	0.0185446	0.0185446	0
TTC16	0.0342806	0	0.0342806
TTC17	0.056518	0	0
TTC18	0.0320168	0.0320168	0
TTC19	0.0448966	0.0448966	0
TTC21A	0.0128124	0	0
TTC21B	0.0363599	0	0.0363599
TTC22	0.0250701	0.0250701	0
TTC23	0.3386064	0	0.3386064
TTC23L	0.0177923	0	0
TTC24	0.1001329	0	0.1001329
TTC25	0.0226997	0	0
TTC26	0.0261935	0	0.0261935
TTC27	0.0326855	0	0
TTC28	0.006271	0	0.006271
TTC29	0.0129914	0	0
TTC3	0.0653718	0	0.0653718
TTC30A	0.023154	0.023154	0
TTC30B	0.0396592	0	0
TTC31	0.0168893	0.0168893	0
TTC32	0.220666	0.220666	0
TTC33	0.1442099	0	0.1442099
TTC35	0.4615796	0	0
TTC36	0.0071529	0.0071529	0
TTC37	0.1133589	0	0.1133589
TTC38	0.0062819	0.0062819	0
TTC39A	0.0405412	0.0405412	0
TTC39B	0.0642094	0	0.0642094
TTC39C	0.4229967	0	0.4229967
TTC4	0.0322106	0	0
TTC5	0.0329096	0	0.0329096
TTC7A	0.0587961	0	0
TTC7B	0.0031786	0	0.0031786
TTC8	0.0537118	0	0

TTC9	0.0365896	0.0365896	0
TTC9B	0.0078532	0.0078532	0
TTC9C	0.2040749	0	0
TTF1	0.044979	0	0.044979
TTF2	0.1862444	0	0.1862444
TTK	0.0370069	0.0370069	0
TTL	0.0163588	0	0.0163588
TTLL1	0.0184209	0.0184209	0
TTLL10	0.0006102	0.0006102	0
TTLL11	0.0317801	0	0.0317801
TTLL12	0.0120499	0.0120499	0
TTLL13	0.0098665	0.0098665	0
TTLL2	0.001118	0.001118	0
TTLL3	0.0128025	0.0128025	0
TTLL4	0.0237989	0.0237989	0
TTLL5	0.0852602	0.0852602	0
TTLL6	0.0119327	0.0119327	0
TTLL7	0.0136332	0	0.0136332
TTLL8	0.0004774	0	0
TTLL9	0.014045	0	0
TTN	0.0115836	0	0.0115836
TTPA	0.0027533	0	0
TTPAL	0.0873567	0	0.0873567
TTR	0.0086438	0	0
TTTY10	1.53E-05	0	0
TTTY11	1.53E-05	0	0
TTTY12	1.53E-05	0	0
TTTY13	1.53E-05	0	0
TTTY14	1.53E-05	1.53E-05	0
TTTY15	1.53E-05	0	0
TTTY16	1.53E-05	0	0
TTTY18	1.53E-05	0	0
TTTY19	1.53E-05	0	0
TTTY20	1.53E-05	0	0
TTTY22	1.53E-05	0	0
TTTY4C	1.53E-05	1.53E-05	0
TTTY5	1.53E-05	0	0
TTYH1	0.0012191	0	0
TTYH2	0.0319188	0	0.0319188
TTYH3	0.0088606	0	0
TUB	0.0036643	0	0.0036643
TUBA1A	0.0139778	0	0.0139778
TUBA1B	0.1011285	0	0.1011285
TUBA1C	0.1605075	0.1605075	0
TUBA3C	0.0007849	0	0.0007849

TUBA3D	0.00045	0	0.00045
TUBA3E	0.0002388	0	0.0002388
TUBA4A	0.0244407	0.0244407	0
TUBA4B	0.0237315	0.0237315	0
TUBA8	0.017862	0	0
TUBAL3	0.0229361	0	0
TUBB1	0.0683475	0	0
TUBB2A	0.0097083	0	0
TUBB2B	0.0051129	0	0.0051129
TUBB2C	0.0199477	0.0199477	0
TUBB3	0.0966215	0	0
TUBB4	0.0016965	0	0
TUBB4Q	0.0163651	0.0163651	0
TUBB6	0.1432281	0	0.1432281
TUBB8	0.0001589	0.0001589	0
TUBBP5	0.0003369	0.0003369	0
TUBD1	0.0362749	0	0.0362749
TUBE1	0.0583063	0	0
TUBG1	0.0320171	0.0320171	0
TUBG2	0.028283	0.028283	0
TUBGCP2	0.0078656	0.0078656	0
TUBGCP3	0.0417228	0	0.0417228
TUBGCP4	0.0383475	0	0
TUBGCP5	0.005252	0	0.005252
TUBGCP6	0.0053855	0	0
TUFM	0.0124146	0.0124146	0
TUFT1	0.6149747	0.6149747	0
TUG1	0.0353684	0	0
TULP1	0.0021024	0	0.0021024
TULP2	0.0895472	0	0.0895472
TULP3	0.0335656	0	0.0335656
TULP4	0.0339153	0	0.0339153
TUSC1	0.309429	0	0
TUSC2	0.019042	0.019042	0
TUSC3	0.0128356	0	0
TUSC5	0.0009875	0	0.0009875
TUT1	0.3823329	0.3823329	0
TWF1	0.066013	0.066013	0
TWF2	0.0325786	0	0
TWIST1	0.0054771	0	0.0054771
TWISTNB	0.0658868	0	0.0658868
TWSG1	0.0258413	0	0.0258413
TXK	0.0288907	0	0.0288907
TXLNA	0.0856061	0	0.0856061
TXLNB	0.0539698	0	0.0539698

TXN	0.3838894	0.3838894	0
TXN2	0.0211916	0.0211916	0
TXNDC11	0.1147775	0.1147775	0
TXNDC12	0.1399136	0.1399136	0
TXNDC15	0.0195508	0	0.0195508
TXNDC16	0.0429074	0	0.0429074
TXNDC17	0.076743	0.076743	0
TXNDC2	0.0260976	0	0.0260976
TXNDC3	0.0097828	0	0.0097828
TXNDC5	0.0304535	0	0.0304535
TXNDC6	0.0512643	0	0
TXNDC8	0.0233798	0	0
TXNDC9	0.1143301	0	0
TXNIP	0.3194074	0	0.3194074
TXNL1	0.1115361	0	0
TXNL4A	0.0178146	0	0
TXNL4B	0.1172595	0	0
TXNRD1	0.595155	0	0.595155
TXNRD2	0.0106224	0.0106224	0
TXNRD3IT1	0.0092808	0	0.0092808
TYK2	0.0224683	0	0
TYMP	1	0	1
TYMS	0.0255672	0	0
TYR	0.01681	0	0.01681
TYRO3	0.0061195	0	0.0061195
TYROBP	0.0530765	0	0.0530765
TYRP1	0.0362145	0	0
TYSND1	0.0514717	0.0514717	0
TYW1	0.0350645	0.0350645	0
TYW1B	0.0608173	0	0.0608173
TYW3	0.0875954	0	0.0875954
U2AF1	0.0687523	0.0687523	0
U2AF1L4	0.2037411	0	0
U2AF2	0.0357452	0.0357452	0
UACA	0.1261443	0	0.1261443
UAP1	0.2207842	0	0
UAP1L1	0.0256636	0	0.0256636
UBA1	0.0536752	0.0536752	0
UBA2	0.0143848	0	0
UBA3	0.3883294	0	0.3883294
UBA5	0.0997402	0	0
UBA52	0.0107251	0	0
UBA6	0.0714838	0.0714838	0
UBA7	0.0067668	0	0.0067668
UBAC1	0.1965025	0.1965025	0

UBAC2	0.0427175	0	0
UBAP1	0.2824503	0	0
UBAP2	0.0291963	0.0291963	0
UBAP2L	0.1670477	0	0
UBASH3A	0.060363	0	0.060363
UBASH3B	0.0364545	0	0.0364545
UBB	0.1423239	0	0
UBC	1	1	0
UBE2A	0.0202027	0	0
UBE2B	0.305976	0	0
UBE2C	0.0361077	0.0361077	0
UBE2CBP	0.1050503	0	0
UBE2D1	0.0662657	0	0.0662657
UBE2D2	0.0670485	0.0670485	0
UBE2D3	0.9389083	0	0
UBE2D4	0.0172094	0	0.0172094
UBE2DNL	0.0024977	0	0
UBE2E1	0.0616242	0	0.0616242
UBE2E2	0.0378421	0	0.0378421
UBE2E3	0.046221	0.046221	0
UBE2F	0.0393398	0	0.0393398
UBE2G1	0.0511548	0	0.0511548
UBE2G2	0.0348472	0	0.0348472
UBE2H	0.2022406	0.2022406	0
UBE2I	0.0069246	0.0069246	0
UBE2J1	0.1135793	0	0.1135793
UBE2J2	0.0029084	0.0029084	0
UBE2K	0.0373012	0.0373012	0
UBE2L3	0.0312092	0.0312092	0
UBE2L6	0.2183756	0	0.2183756
UBE2M	0.0263979	0.0263979	0
UBE2MP1	0.0003079	0.0003079	0
UBE2N	0.0887175	0	0.0887175
UBE2NL	0.0031745	0	0
UBE2O	0.0312319	0	0
UBE2Q1	0.1887716	0.1887716	0
UBE2Q2	0.066303	0	0.066303
UBE2Q2P1	0.0087756	0	0
UBE2QL1	0.0261606	0	0.0261606
UBE2R2	0.1028327	0	0
UBE2S	0.0244865	0.0244865	0
UBE2T	0.2453602	0.2453602	0
UBE2U	0.0077699	0	0.0077699
UBE2V1	0.136748	0.136748	0
UBE2V2	0.0085575	0	0.0085575

UBE2W	0.0654034	0	0.0654034
UBE2Z	0.0612238	0	0
UBE3A	0.0568011	0	0.0568011
UBE3B	0.0260233	0.0260233	0
UBE3C	0.0367559	0	0
UBE4A	0.0349571	0	0.0349571
UBE4B	0.0185754	0	0.0185754
UBFD1	0.0357726	0	0
UBIAD1	0.0137075	0	0.0137075
UBL3	0.1029143	0	0
UBL4A	0.0034749	0.0034749	0
UBL4B	0.006784	0	0.006784
UBL5	0.0120654	0.0120654	0
UBL7	0.0963936	0.0963936	0
UBLCP1	0.0680422	0	0.0680422
UBN1	0.0295406	0	0
UBN2	0.0498994	0.0498994	0
UBOX5	0.01282	0.01282	0
UBP1	0.131464	0	0.131464
UBQLN1	0.162148	0	0
UBQLN2	0.1584862	0	0.1584862
UBQLN3	0.0116643	0	0
UBQLN4	0.0665415	0.0665415	0
UBQLNL	0.0061035	0	0
UBR1	0.2701936	0	0.2701936
UBR2	0.2354412	0	0.2354412
UBR3	0.0292539	0	0.0292539
UBR4	0.0931295	0	0
UBR5	0.1888581	0	0.1888581
UBR7	0.0567522	0	0.0567522
UBTD1	0.047977	0	0.047977
UBTD2	0.1798991	0	0.1798991
UBTF	0.0686617	0	0.0686617
UBTFL1	2.71E-05	0	2.71E-05
UBXN1	0.0620343	0.0620343	0
UBXN10	0.0118306	0.0118306	0
UBXN11	0.0473163	0.0473163	0
UBXN2A	0.0214343	0	0.0214343
UBXN2B	0.0419922	0	0.0419922
UBXN4	0.0781089	0	0
UBXN6	0.0062879	0.0062879	0
UBXN7	0.055329	0	0.055329
UBXN8	0.252126	0	0
UCA1	0.0780945	0	0
UCHL1	0.0072882	0	0.0072882

UCHL3	0.0834494	0.0834494	0
UCHL5	0.3230223	0	0
UCK1	0.0049929	0	0
UCK2	0.1039982	0.1039982	0
UCKL1	0.0056138	0.0056138	0
UCKL1AS	0.0039967	0.0039967	0
UCMA	0.00081	0	0.00081
UCN	0.0067064	0.0067064	0
UCN2	0.0089836	0	0
UCN3	0.0046428	0	0.0046428
UCP1	0.0042842	0	0.0042842
UCP2	0.0336865	0	0.0336865
UCP3	0.0095558	0	0.0095558
UEVLD	0.1266601	0	0.1266601
UFC1	0.0530059	0.0530059	0
UFD1L	0.0561595	0	0
UFM1	0.0576112	0.0576112	0
UFSP1	0.0073903	0.0073903	0
UFSP2	0.0369094	0	0.0369094
UGCG	0.185596	0	0.185596
UGDH	0.0494631	0	0.0494631
UGGT1	0.022054	0	0.022054
UGGT2	0.0293488	0	0.0293488
UGP2	0.1472744	0	0.1472744
UGT1A1	0.0275928	0.0275928	0
UGT1A10	0.0051871	0.0051871	0
UGT1A3	0.0015857	0.0015857	0
UGT1A4	0.0056841	0.0056841	0
UGT1A5	0.0278384	0.0278384	0
UGT1A6	0.097994	0.097994	0
UGT1A7	0.0466717	0.0466717	0
UGT1A8	0.0042169	0.0042169	0
UGT1A9	0.0076159	0.0076159	0
UGT2A1	0.0075042	0	0
UGT2A3	0.0207512	0	0
UGT2B10	0.010671	0.010671	0
UGT2B11	0.0020709	0	0
UGT2B28	0.002234	0	0
UGT2B4	0.0076532	0	0
UGT2B7	0.033644	0	0
UGT3A1	0.0116558	0	0
UGT3A2	0.0211711	0	0.0211711
UGT8	0.018252	0.018252	0
UHMK1	0.0680276	0	0.0680276
UHRF1	0.2179819	0.2179819	0

UHRF1BP1	0.0278948	0	0.0278948
UHRF1BP1L	0.0383828	0	0.0383828
UHRF2	0.0722507	0	0.0722507
UIMC1	0.054407	0	0
ULBP1	0.0019344	0	0
ULBP2	0.2899849	0	0
ULBP3	0.0088222	0.0088222	0
ULK1	0.0017487	0.0017487	0
ULK2	0.006819	0	0.006819
ULK3	0.0055898	0.0055898	0
ULK4	0.046335	0	0
UMOD	0.0019185	0	0
UMODL1	0.0059498	0	0.0059498
UMPS	0.0507008	0.0507008	0
UNC119	0.0123354	0	0
UNC119B	0.0165638	0	0.0165638
UNC13A	0.0061686	0	0.0061686
UNC13B	0.0107339	0.0107339	0
UNC13C	0.0142227	0	0.0142227
UNC13D	0.0162975	0	0
UNC45A	0.116964	0.116964	0
UNC45B	0.1776544	0	0.1776544
UNC50	0.050069	0	0
UNC5A	0.0020295	0	0.0020295
UNC5B	0.0092103	0	0.0092103
UNC5C	0.0348032	0	0.0348032
UNC5CL	0.0710527	0.0710527	0
UNC5D	0.0025266	0	0.0025266
UNC80	0.0063512	0	0.0063512
UNC93A	0.004464	0.004464	0
UNC93B1	0.0280482	0.0280482	0
UNCX	0.0003274	0.0003274	0
UNG	0.035556	0.035556	0
UNK	0.5222838	0	0
UNKL	0.0112504	0	0
UOX	0.0325463	0	0
UPB1	0.0172645	0	0.0172645
UPF0639	0.0028907	0	0
UPF1	0.0154671	0.0154671	0
UPF2	0.0965763	0	0.0965763
UPF3A	0.0523763	0.0523763	0
UPF3B	0.0245938	0.0245938	0
UPK1A	0.0035376	0	0
UPK1B	0.0050804	0	0.0050804
UPK2	0.0013604	0	0

UPK3A	0.0028039	0	0
UPK3B	0.0016461	0.0016461	0
UPK3BL	1.55E-05	1.55E-05	0
UPP1	0.3893237	0	0
UPP2	0.0119303	0	0
UPRT	0.0097361	0	0
UQCC	0.0331218	0.0331218	0
UQCR10	0.0202097	0.0202097	0
UQCR11	0.0080919	0.0080919	0
UQCRB	0.0756413	0.0756413	0
UQCRC1	0.0346728	0.0346728	0
UQCRC2	0.1155019	0	0
UQCRFS1	0.0162991	0.0162991	0
UQCRH	0.0546721	0.0546721	0
UQCRHL	0.0180675	0.0180675	0
UQCRQ	0.0938196	0.0938196	0
URB1	0.0221149	0	0.0221149
URB2	0.0917916	0	0.0917916
URGCP	0.0445141	0.0445141	0
URM1	0.0453384	0.0453384	0
UROC1	0.0019522	0	0
UROD	0.0470147	0	0.0470147
UROS	0.0510801	0	0
USE1	0.0209201	0.0209201	0
USF1	0.1016671	0	0
USF2	0.0032136	0	0
USH1C	0.00296	0.00296	0
USH1G	0.0009766	0.0009766	0
USH2A	0.0206591	0	0
USHBP1	0.0151619	0	0.0151619
USMG5	0.0457941	0.0457941	0
USO1	0.0539945	0	0
USP1	0.1867497	0	0.1867497
USP10	0.0191697	0	0
USP11	0.0325332	0	0.0325332
USP12	0.0562415	0	0.0562415
USP13	0.0439526	0	0.0439526
USP14	0.0177149	0	0.0177149
USP15	0.6829203	0	0.6829203
USP16	0.0993374	0	0.0993374
USP17L2	0.0009817	0	0
USP17L6P	0.000119	0	0
USP18	0.0167194	0	0.0167194
USP19	0.0030564	0.0030564	0
USP2	0.0392864	0	0.0392864

USP20	0.0298291	0	0
USP21	0.0816192	0.0816192	0
USP22	0.0445929	0	0.0445929
USP24	0.0752202	0	0.0752202
USP25	0.1174149	0	0.1174149
USP26	0.0044232	0	0
USP27X	0.013885	0	0
USP28	0.0329792	0	0.0329792
USP29	0.0014191	0	0
USP3	0.6197834	0	0
USP30	0.0219111	0	0
USP31	0.0280511	0	0.0280511
USP32	0.8742705	0	0.8742705
USP33	0.0738699	0	0.0738699
USP34	0.0515373	0	0.0515373
USP35	0.0404066	0	0
USP36	0.0289502	0	0
USP37	0.0923732	0	0
USP38	0.1101938	0	0.1101938
USP39	0.027186	0.027186	0
USP4	0.0704038	0	0.0704038
USP40	0.0290269	0.0290269	0
USP42	0.0312257	0	0
USP43	0.0794224	0.0794224	0
USP44	0.0054013	0	0.0054013
USP45	0.0669748	0	0.0669748
USP46	0.0221204	0	0.0221204
USP47	0.367031	0	0.367031
USP48	0.2687734	0	0.2687734
USP49	0.0350515	0	0
USP5	0.0280932	0.0280932	0
USP50	0.0202942	0	0.0202942
USP51	0.0014145	0	0.0014145
USP53	0.1601705	0	0
USP54	0.0667897	0.0667897	0
USP6	0.0012417	0	0
USP6NL	0.0794113	0	0
USP7	0.0202722	0	0
USP8	0.0834644	0	0.0834644
USP9X	0.1608227	0	0.1608227
USP9Y	1.53E-05	0	0
USPL1	0.2302269	0	0.2302269
UST	0.0439877	0	0.0439877
UTF1	0.0001546	0	0.0001546
UTP11L	0.0899279	0	0.0899279

UTP14A	0.008712	0	0
UTP14C	0.0138732	0	0
UTP15	0.0594376	0	0.0594376
UTP18	0.1121467	0	0
UTP20	0.0273973	0	0.0273973
UTP23	0.238239	0	0.238239
UTP3	0.0844667	0	0.0844667
UTP6	0.029627	0	0
UTRN	0.0430465	0	0.0430465
UTS2	0.0042648	0	0.0042648
UTS2D	0.2866358	0	0.2866358
UTS2R	0.0010964	0	0.0010964
UTY	1.53E-05	0	0
UVRAG	0.2214488	0	0.2214488
UXS1	0.0272533	0	0.0272533
UXT	0.055213	0.055213	0
VAC14	0.0351119	0	0
VAMP1	0.0703999	0	0.0703999
VAMP2	0.0673058	0	0.0673058
VAMP3	0.0206134	0	0.0206134
VAMP4	0.4302882	0	0
VAMP5	0.027975	0	0.027975
VAMP8	0.0275323	0.0275323	0
VANGL1	0.0258307	0	0
VANGL2	0.0140818	0	0
VAPA	0.1719539	0.1719539	0
VAPB	0.0350809	0.0350809	0
VASH1	0.0022591	0	0.0022591
VASH2	0.0790945	0	0.0790945
VASN	0.001227	0	0.001227
VASP	0.0409939	0.0409939	0
VAT1	0.0055173	0	0.0055173
VAT1L	0.0133996	0	0.0133996
VAV1	0.0276995	0	0.0276995
VAV2	0.0186199	0.0186199	0
VAV3	0.4188856	0	0.4188856
VAX1	0.0015996	0	0
VAX2	0.0016257	0.0016257	0
VBP1	0.0092576	0	0
VCAM1	0.036547	0	0.036547
VCAN	0.0234654	0	0.0234654
VCL	0.064257	0	0.064257
VCP	0.0831072	0	0
VCPIP1	0.2501892	0	0.2501892
VCX	0.0017792	0	0

VCX2	0.0021697	0.0021697	0
VCX3A	0.0016196	0.0016196	0
VCX3B	0.0022053	0	0
VDAC1	0.0268136	0.0268136	0
VDAC2	0.1429182	0.1429182	0
VDAC3	0.047459	0	0
VDR	0.058238	0	0.058238
VEGFA	0.0285572	0.0285572	0
VEGFB	0.026252	0	0.026252
VEGFC	0.0212678	0	0.0212678
VENTX	0.000349	0	0.000349
VENTXP1	0.0037436	0	0
VENTXP7	0.0078103	0	0
VEPH1	0.0410048	0	0.0410048
VEZF1	0.1158618	0	0.1158618
VEZT	0.1456091	0	0
VGF	0.0127506	0	0
VGLL1	0.0032974	0	0
VGLL2	0.0036726	0	0.0036726
VGLL3	0.1432702	0	0.1432702
VGLL4	0.157726	0	0.157726
VHL	0.0119849	0	0.0119849
VHLL	0.0133075	0.0133075	0
VIL1	0.0010176	0.0010176	0
VILL	0.0095008	0.0095008	0
VIM	0.1057434	0	0.1057434
VIP	0.0199927	0	0.0199927
VIPAR	0.0605422	0	0
VIPR1	0.0032949	0.0032949	0
VIPR2	0.0021341	0	0.0021341
VIT	0.080194	0	0.080194
VKORC1	0.017234	0	0
VKORC1L1	0.0028836	0.0028836	0
VLDLR	0.0173632	0	0.0173632
VMA21	0.0230383	0	0
VMAC	0.0064601	0.0064601	0
VMO1	0.0963025	0	0.0963025
VN1R1	0.0031712	0.0031712	0
VN1R2	0.0024522	0	0
VN1R4	0.0015873	0	0
VN1R5	0.0198798	0	0
VNN1	0.0156884	0	0
VNN2	0.0603427	0	0.0603427
VNN3	0.454498	0	0.454498
VOPP1	0.101375	0	0.101375

VPRBP	0.0757991	0	0
VPREB1	0.000892	0	0.000892
VPREB3	0.0029492	0	0.0029492
VPS11	0.0184779	0	0.0184779
VPS13A	0.0322097	0.0322097	0
VPS13B	0.0725072	0	0.0725072
VPS13C	0.2316814	0	0.2316814
VPS13D	0.0137135	0	0.0137135
VPS16	0.0180024	0	0
VPS18	0.0536577	0	0.0536577
VPS24	0.0755323	0	0
VPS25	0.1041077	0.1041077	0
VPS26A	0.0454422	0	0.0454422
VPS26B	0.0229022	0	0
VPS28	0.0074917	0.0074917	0
VPS29	0.0964541	0.0964541	0
VPS33A	0.0252508	0	0.0252508
VPS33B	0.0597418	0	0
VPS35	0.0699634	0	0
VPS36	0.053103	0.053103	0
VPS37A	0.1035459	0	0.1035459
VPS37B	0.3994458	0.3994458	0
VPS37C	0.1248045	0.1248045	0
VPS37D	0.0042975	0.0042975	0
VPS39	0.0879438	0	0.0879438
VPS41	0.0301148	0	0.0301148
VPS45	0.2275073	0	0
VPS4A	0.0104084	0.0104084	0
VPS4B	0.0651986	0	0.0651986
VPS53	0.0136354	0	0
VPS54	0.064487	0	0
VPS72	0.2660574	0.2660574	0
VPS8	0.0891406	0	0.0891406
VRK1	0.0347741	0	0
VRK2	0.2134422	0	0.2134422
VRK3	0.9834698	0.9834698	0
VSIG1	0.0408637	0.0408637	0
VSIG10	0.0058881	0.0058881	0
VSIG10L	0.0059033	0	0
VSIG2	0.006998	0.006998	0
VSIG4	0.0186984	0	0.0186984
VSIG8	0.0144423	0.0144423	0
VSNL1	0.1231401	0	0.1231401
VSTM1	0.0006726	0	0.0006726
VSTM2A	0.0051291	0	0.0051291

VSTM2B	0.0005987	0	0.0005987
VSTM2L	0.0056083	0.0056083	0
VSX1	0.0298392	0	0
VSX2	0.0017485	0	0.0017485
VTA1	0.0930996	0	0.0930996
VTCN1	0.0035699	0	0.0035699
VTI1A	0.0497206	0	0
VTI1B	0.0573663	0.0573663	0
VTN	0.1114971	0	0
VTRNA1-1	0.306006	0	0
VTRNA1-2	0.3286494	0	0
VTRNA1-3	0.1395743	0	0
VWA1	0.0006083	0.0006083	0
VWA2	0.0166937	0.0166937	0
VWA3A	0.0150322	0.0150322	0
VWA3B	0.0101739	0.0101739	0
VWA5A	0.8105148	0.8105148	0
VWA5B1	0.0024711	0.0024711	0
VWA5B2	0.0043638	0	0.0043638
VWC2	0.0106328	0	0.0106328
VWC2L	0.0132691	0.0132691	0
VWCE	0.0197015	0	0.0197015
VWDE	0.0184635	0	0.0184635
VWF	0.0053612	0	0.0053612
WAC	0.0402864	0	0.0402864
WAPAL	0.0398689	0	0.0398689
WARS	0.2079158	0	0.2079158
WARS2	0.2754789	0.2754789	0
WAS	0.0140949	0	0.0140949
WASF1	0.1307925	0	0.1307925
WASF2	0.0739949	0	0.0739949
WASF3	0.0044889	0	0.0044889
WASH2P	0.0007104	0.0007104	0
WASH3P	2.34E-05	2.34E-05	0
WASH5P	1.53E-05	1.53E-05	0
WASL	0.1325703	0.1325703	0
WBP1	0.1382257	0.1382257	0
WBP11	0.1488921	0	0
WBP11P1	0.0089968	0	0
WBP2	0.1137913	0.1137913	0
WBP2NL	0.0083136	0	0
WBP4	0.2147955	0	0.2147955
WBP5	0.0059123	0	0.0059123
WBSCR16	0.002285	0.002285	0
WBSCR17	0.0006881	0	0.0006881

WBSCR22	0.0105694	0.0105694	0
WBSCR26	0.0369668	0.0369668	0
WBSCR27	0.0269067	0.0269067	0
WBSCR28	0.0279446	0.0279446	0
WDFY1	0.0732729	0	0.0732729
WDFY2	0.2741806	0	0
WDFY3	0.0231823	0	0.0231823
WDFY4	0.0196637	0	0.0196637
WDHD1	0.0744382	0	0
WDR1	0.0144263	0	0.0144263
WDR11	0.0349125	0	0.0349125
WDR12	0.0360251	0	0
WDR13	0.0297159	0.0297159	0
WDR16	0.0213483	0.0213483	0
WDR17	0.0073398	0	0.0073398
WDR18	0.0028241	0.0028241	0
WDR19	0.0432763	0	0.0432763
WDR20	0.0168509	0.0168509	0
WDR24	0.0075391	0.0075391	0
WDR25	0.166112	0.166112	0
WDR26	0.1475904	0	0.1475904
WDR27	0.0285011	0.0285011	0
WDR3	0.0885499	0	0.0885499
WDR31	0.0280502	0	0
WDR33	0.0282008	0	0
WDR34	0.023053	0.023053	0
WDR35	0.0515314	0	0
WDR36	0.0340453	0	0.0340453
WDR37	0.0475754	0	0.0475754
WDR38	0.0379954	0	0
WDR4	0.0243763	0.0243763	0
WDR41	0.0565932	0	0.0565932
WDR43	0.0760599	0	0
WDR44	0.0681686	0	0.0681686
WDR45	0.010536	0	0
WDR45L	0.005222	0.005222	0
WDR47	0.0118241	0	0.0118241
WDR48	0.0272366	0	0.0272366
WDR49	0.0188201	0	0.0188201
WDR5	0.0097779	0.0097779	0
WDR52	0.012835	0.012835	0
WDR53	0.1040398	0	0.1040398
WDR54	0.0099301	0.0099301	0
WDR55	0.0328726	0.0328726	0
WDR59	0.0124665	0.0124665	0

WDR5B	0.1385568	0.1385568	0
WDR6	0.0077121	0.0077121	0
WDR60	0.0425506	0.0425506	0
WDR61	0.0761134	0	0
WDR62	0.0685032	0.0685032	0
WDR63	0.0182041	0	0.0182041
WDR64	0.0459007	0	0.0459007
WDR65	0.0187084	0	0
WDR66	0.01365	0.01365	0
WDR67	0.115236	0	0
WDR69	0.3399098	0	0
WDR7	0.0595489	0	0.0595489
WDR70	0.0516289	0	0
WDR72	0.0070237	0	0
WDR73	0.0787464	0.0787464	0
WDR74	1	1	0
WDR75	0.0638213	0	0
WDR76	0.1001018	0	0.1001018
WDR77	0.1012811	0	0
WDR78	0.159596	0	0.159596
WDR8	0.0152299	0.0152299	0
WDR81	0.1593249	0	0.1593249
WDR82	0.1033224	0	0.1033224
WDR83	0.0148821	0.0148821	0
WDR85	0.011307	0.011307	0
WDR86	0.0208562	0	0.0208562
WDR87	0.0361447	0	0
WDR88	0.0022751	0	0
WDR89	0.1228961	0	0
WDR90	0.0026095	0.0026095	0
WDR91	0.0506501	0	0.0506501
WDR92	0.0935261	0	0.0935261
WDR93	0.0664609	0.0664609	0
WDSUB1	0.0296411	0	0
WDTC1	0.0132513	0	0.0132513
WDYHV1	0.0491749	0.0491749	0
WEE1	0.4008354	0	0
WEE2	0.0125624	0.0125624	0
WFDC1	0.0020834	0	0.0020834
WFDC10A	0.0155674	0.0155674	0
WFDC10B	0.0094228	0.0094228	0
WFDC11	0.0056151	0	0
WFDC12	0.022145	0	0
WFDC13	0.005819	0.005819	0
WFDC2	0.0064877	0.0064877	0

WFDC3	0.0182471	0.0182471	0
WFDC5	0.0126153	0.0126153	0
WFDC6	0.0156691	0.0156691	0
WFDC8	0.0098164	0	0
WFDC9	0.0110789	0	0
WFIKKN1	0.009442	0.009442	0
WFIKKN2	0.001765	0	0.001765
WFS1	0.0049524	0.0049524	0
WHAMM	0.1573504	0	0
WHAMML1	0.0004088	0	0.0004088
WHAMML2	9.94E-05	0	9.94E-05
WHSC1	0.0669289	0.0669289	0
WHSC1L1	0.1009054	0	0.1009054
WHSC2	0.013139	0.013139	0
WIBG	0.1897921	0.1897921	0
WIF1	0.0120319	0	0
WIPF1	0.0350053	0	0.0350053
WIPF2	0.0121424	0.0121424	0
WIPF3	0.0079059	0	0.0079059
WIPI1	0.1255775	0	0.1255775
WIPI2	0.0203477	0.0203477	0
WISP1	0.1005316	0	0.1005316
WISP2	0.0426427	0	0.0426427
WISP3	0.0040224	0	0
WIT1	0.002927	0	0.002927
WIZ	0.0037522	0.0037522	0
WLS	0.0164242	0	0.0164242
WNK1	0.2311491	0	0.2311491
WNK2	0.0010746	0.0010746	0
WNK3	0.0018007	0	0.0018007
WNK4	0.0342725	0.0342725	0
WNT1	0.0087229	0	0.0087229
WNT10A	0.012196	0	0
WNT10B	0.0093047	0	0.0093047
WNT11	0.0042782	0	0.0042782
WNT16	0.0113206	0	0.0113206
WNT2	0.0151684	0	0.0151684
WNT2B	0.0495421	0	0.0495421
WNT3	0.0014529	0	0
WNT3A	0.0112616	0.0112616	0
WNT4	0.0019175	0	0
WNT5A	0.0335767	0	0.0335767
WNT5B	0.0363978	0	0.0363978
WNT6	0.0044335	0	0
WNT7A	0.0028065	0	0

WNT7B	0.0035537	0.0035537	0
WNT8A	0.0094842	0	0
WNT8B	0.003099	0	0
WNT9A	0.006914	0	0.006914
WNT9B	0.002226	0	0.002226
WRAP53	0.0966918	0	0
WRB	0.0339082	0	0
WRN	0.0428012	0	0.0428012
WRNIP1	0.0755116	0.0755116	0
WSB1	0.0970401	0	0
WSB2	0.0587676	0.0587676	0
WSCD1	0.0012499	0	0.0012499
WSCD2	0.0026038	0	0.0026038
WT1	0.0031392	0	0.0031392
WTAP	0.1290805	0	0.1290805
WTIP	0.0035086	0	0.0035086
WWC1	0.0628846	0.0628846	0
WWC2	0.0215608	0	0.0215608
WWC3	0.074451	0	0.074451
WWOX	0.0596096	0	0.0596096
WWP1	0.0502983	0	0
WWP2	0.0419816	0	0
WWTR1	0.1333515	0	0.1333515
XAB2	0.0069931	0.0069931	0
XAF1	0.0159437	0	0.0159437
XAGE3	0.0064218	0	0
XAGE5	0.005445	0	0
XBP1	0.092111	0	0
XCL1	0.0289913	0	0.0289913
XCL2	0.0271369	0	0.0271369
XCR1	0.0079672	0	0.0079672
XDH	0.0638719	0.0638719	0
XG	0.0040423	0	0.0040423
XIAP	0.0144567	0	0
XIRP1	0.0065176	0	0.0065176
XIRP2	0.0024493	0	0.0024493
XIST	0.0485223	0	0.0485223
XK	0.015103	0.015103	0
XKR3	0.0148469	0	0.0148469
XKR4	0.0083409	0	0.0083409
XKR5	0.0044907	0	0.0044907
XKR6	0.0406587	0	0
XKR7	0.0054389	0	0.0054389
XKR8	0.0092541	0	0.0092541
XKR9	0.0825951	0.0825951	0

XKRX	0.005738	0	0
XPA	0.1335924	0	0
XPC	0.1633445	0	0.1633445
XPNPEP1	0.0485986	0.0485986	0
XPNPEP2	0.0103773	0	0.0103773
XPNPEP3	0.0576592	0	0
XPO1	0.0904175	0	0
XPO4	0.0080978	0	0.0080978
XPO5	0.0375253	0	0
XPO6	0.0398846	0	0.0398846
XPO7	0.0563354	0	0
XPOT	0.0308969	0	0.0308969
XPR1	0.160427	0	0
XRCC1	0.0176988	0.0176988	0
XRCC2	0.0265873	0.0265873	0
XRCC3	0.0052359	0.0052359	0
XRCC4	0.0980245	0	0
XRCC5	0.1078023	0	0
XRCC6	0.0467357	0	0
XRCC6BP1	0.047352	0.047352	0
XRN1	0.0743437	0	0.0743437
XRN2	0.1956464	0	0.1956464
XRRA1	0.0945083	0	0
XYLB	0.0094393	0.0094393	0
XYLT1	0.008814	0	0.008814
XYLT2	0.0095516	0.0095516	0
YAF2	0.0293978	0	0.0293978
YAP1	0.3419345	0	0.3419345
YARS	0.1662717	0.1662717	0
YARS2	0.0735531	0.0735531	0
YBX1	0.1187261	0	0.1187261
YBX2	0.0036684	0.0036684	0
YDJC	0.0099846	0.0099846	0
YEATS2	0.0228983	0	0.0228983
YEATS4	0.0514309	0.0514309	0
YES1	0.0645651	0	0
YIF1A	0.0095307	0.0095307	0
YIF1B	0.0169358	0.0169358	0
YIPF1	0.5006933	0	0
YIPF2	0.0102277	0.0102277	0
YIPF3	0.0502557	0.0502557	0
YIPF4	0.0426296	0.0426296	0
YIPF5	0.109744	0	0.109744
YIPF6	0.0246	0	0
YIPF7	0.006857	0.006857	0

YJEFN3	0.002763	0.002763	0
YKT6	0.0133114	0	0
YLPM1	0.0601642	0	0.0601642
YME1L1	0.0591384	0	0.0591384
YOD1	0.5182498	0.5182498	0
YPEL1	0.0037965	0	0.0037965
YPEL2	0.019746	0	0.019746
YPEL3	0.0131515	0.0131515	0
YPEL4	0.0199578	0	0.0199578
YPEL5	0.0762777	0	0.0762777
YRDC	0.0146333	0	0
YSK4	0.003426	0.003426	0
YTHDC1	0.2483421	0	0
YTHDC2	0.0900877	0	0.0900877
YTHDF1	0.0839036	0.0839036	0
YTHDF2	0.0451567	0	0.0451567
YTHDF3	0.0594278	0	0.0594278
YWHAB	0.0667147	0	0
YWHAE	0.074286	0.074286	0
YWHAG	0.2398266	0	0
YWHAH	0.0459655	0	0
YWHAQ	0.0807722	0	0.0807722
YWHAZ	0.2341758	0.2341758	0
YY1	0.0536059	0.0536059	0
YY1AP1	0.0552957	0.0552957	0
YY2	0.0047741	0	0.0047741
ZACN	0.0047288	0	0
ZADH2	0.0871111	0	0
ZAK	0.0470264	0	0.0470264
ZAN	0.0019643	0	0
ZAP70	0.0043856	0	0.0043856
ZAR1	0.0069025	0.0069025	0
ZAR1L	0.0568463	0	0
ZBBX	0.0156341	0	0
ZBED2	0.1952414	0	0.1952414
ZBED3	0.0494928	0	0.0494928
ZBED4	0.0055013	0	0.0055013
ZBED5	0.2868483	0	0
ZBP1	0.0946572	0	0.0946572
ZBTB1	0.2878949	0	0.2878949
ZBTB10	0.1059578	0	0.1059578
ZBTB11	0.1719194	0	0.1719194
ZBTB16	0.0022291	0	0.0022291
ZBTB17	0.1648262	0	0
ZBTB2	0.0513391	0	0.0513391

ZBTB20	0.1383808	0	0.1383808
ZBTB24	0.0957492	0	0.0957492
ZBTB25	0.3048369	0	0.3048369
ZBTB26	0.1112656	0	0
ZBTB3	0.0209668	0	0
ZBTB32	0.0327308	0	0.0327308
ZBTB33	0.0395979	0	0.0395979
ZBTB34	0.0494119	0	0
ZBTB37	0.5690723	0	0.5690723
ZBTB38	0.0823124	0	0.0823124
ZBTB39	0.0183769	0	0.0183769
ZBTB4	0.1632172	0	0.1632172
ZBTB40	0.0371426	0	0.0371426
ZBTB41	0.2110945	0	0
ZBTB42	0.0028636	0.0028636	0
ZBTB43	0.1467021	0	0
ZBTB44	0.0315977	0	0.0315977
ZBTB45	0.0210416	0.0210416	0
ZBTB46	0.0010056	0	0.0010056
ZBTB47	0.0128522	0	0.0128522
ZBTB48	0.0033165	0.0033165	0
ZBTB49	0.0120997	0	0.0120997
ZBTB5	0.1742259	0	0
ZBTB6	0.1494272	0	0.1494272
ZBTB7A	0.0060684	0.0060684	0
ZBTB7B	0.0836612	0.0836612	0
ZBTB7C	0.0042657	0.0042657	0
ZBTB8A	0.0245803	0	0.0245803
ZBTB8B	0.0042426	0	0
ZBTB8OS	0.0482472	0.0482472	0
ZBTB9	0.0082508	0	0
ZC3H10	0.5336383	0	0.5336383
ZC3H11A	0.348483	0.348483	0
ZC3H12A	0.1438055	0	0
ZC3H12B	0.0114478	0	0
ZC3H12C	0.2920898	0	0.2920898
ZC3H12D	0.0646339	0	0.0646339
ZC3H13	0.0540948	0	0.0540948
ZC3H14	0.0317666	0	0
ZC3H15	0.0978415	0	0
ZC3H18	0.0083733	0.0083733	0
ZC3H3	0.6151943	0.6151943	0
ZC3H4	0.0462723	0.0462723	0
ZC3H6	0.0139738	0	0.0139738
ZC3H7A	0.0934448	0	0.0934448

ZC3H7B	0.004109	0	0.004109
ZC3H8	0.0276483	0.0276483	0
ZC3HAV1	0.2388328	0	0.2388328
ZC3HAV1L	0.0168783	0	0
ZC3HC1	0.0203012	0.0203012	0
ZC4H2	0.0055771	0	0
ZCCHC10	0.0696089	0	0.0696089
ZCCHC11	0.0450616	0	0.0450616
ZCCHC12	0.0030307	0	0.0030307
ZCCHC13	0.0061301	0.0061301	0
ZCCHC14	0.0232297	0	0.0232297
ZCCHC16	0.0047337	0	0.0047337
ZCCHC17	0.0478182	0	0.0478182
ZCCHC18	0.0047112	0	0.0047112
ZCCHC2	0.0758749	0	0.0758749
ZCCHC24	0.0563405	0	0.0563405
ZCCHC3	0.0553056	0	0
ZCCHC4	0.0324748	0.0324748	0
ZCCHC5	0.0168631	0	0.0168631
ZCCHC6	0.1414696	0	0.1414696
ZCCHC7	0.0589736	0	0
ZCCHC8	0.0998135	0.0998135	0
ZCCHC9	0.0428767	0	0
ZCRB1	0.0924157	0.0924157	0
ZCWPW1	0.0996238	0	0.0996238
ZCWPW2	0.007911	0	0.007911
ZDBF2	0.0153151	0	0.0153151
ZDHHC1	0.0055882	0.0055882	0
ZDHHC11	0.0016859	0.0016859	0
ZDHHC12	0.0199257	0.0199257	0
ZDHHC13	0.0420178	0.0420178	0
ZDHHC14	0.024558	0	0.024558
ZDHHC15	0.0038864	0	0.0038864
ZDHHC16	0.0348145	0.0348145	0
ZDHHC17	0.0334197	0	0.0334197
ZDHHC18	0.0114401	0	0.0114401
ZDHHC19	0.0046055	0	0.0046055
ZDHHC2	0.0439364	0	0
ZDHHC20	0.0202704	0	0.0202704
ZDHHC21	0.0149928	0	0.0149928
ZDHHC22	0.0050045	0	0.0050045
ZDHHC23	0.0168521	0.0168521	0
ZDHHC24	0.00725	0.00725	0
ZDHHC3	0.0562066	0.0562066	0
ZDHHC4	0.0127759	0.0127759	0

ZDHHC5	0.1081972	0.1081972	0
ZDHHC6	0.0511196	0.0511196	0
ZDHHC7	0.0297662	0.0297662	0
ZDHHC8	0.0012457	0.0012457	0
ZDHHC8P1	0.0006132	0.0006132	0
ZDHHC9	0.0066134	0.0066134	0
ZEB1	0.0494998	0	0.0494998
ZEB2	0.0241427	0	0.0241427
ZER1	0.0102823	0.0102823	0
ZFAND1	0.0548034	0.0548034	0
ZFAND2A	0.0072139	0.0072139	0
ZFAND2B	0.0050864	0.0050864	0
ZFAND3	0.0963647	0	0.0963647
ZFAND5	0.1833507	0	0.1833507
ZFAND6	0.1135797	0	0
ZFAT	0.0615981	0	0.0615981
ZFATAS	0.0766644	0	0
ZFC3H1	0.2430945	0	0
ZFHX3	0.1277119	0	0.1277119
ZFHX4	0.012526	0	0.012526
ZFP1	0.0382924	0	0.0382924
ZFP106	0.0278895	0	0.0278895
ZFP112	0.0202831	0.0202831	0
ZFP14	0.0034204	0	0.0034204
ZFP161	0.0294069	0	0.0294069
ZFP2	0.0225025	0	0.0225025
ZFP28	0.0015711	0	0.0015711
ZFP3	0.0039688	0	0.0039688
ZFP30	0.0266149	0	0.0266149
ZFP36	0.963208	0	0.963208
ZFP36L1	0.6669318	0	0.6669318
ZFP36L2	0.2738244	0	0.2738244
ZFP37	0.0042777	0	0.0042777
ZFP41	0.0031362	0.0031362	0
ZFP42	0.0036976	0	0.0036976
ZFP64	0.046915	0.046915	0
ZFP82	0.0085034	0	0.0085034
ZFP90	0.0075887	0	0.0075887
ZFP91	0.5767364	0	0.5767364
ZFP91-CNTF	0.5767364	0	0
ZFP92	0.0010031	0	0.0010031
ZFPL1	0.046223	0.046223	0
ZFPM1	0.0016836	0.0016836	0
ZFPM2	0.0076476	0	0.0076476
ZFR	0.0868293	0	0.0868293

ZFR2	0.0015668	0	0.0015668
ZFX	0.2240943	0	0.2240943
ZFY	1.53E-05	0	0
ZFYVE1	0.100559	0	0.100559
ZFYVE16	0.1112069	0	0.1112069
ZFYVE19	0.024626	0.024626	0
ZFYVE20	0.0497204	0	0.0497204
ZFYVE21	0.0051668	0.0051668	0
ZFYVE26	0.0867844	0	0.0867844
ZFYVE27	0.0607704	0.0607704	0
ZFYVE28	0.0595894	0.0595894	0
ZFYVE9	0.0392949	0	0.0392949
ZG16	0.007901	0	0
ZG16B	0.0110364	0.0110364	0
ZGLP1	0.0017696	0.0017696	0
ZGPAT	0.0587755	0.0587755	0
ZHX1	0.1533373	0	0.1533373
ZHX2	0.0806438	0	0.0806438
ZHX3	0.034462	0	0.034462
ZIC1	0.0031433	0	0
ZIC2	0.0032549	0.0032549	0
ZIC3	0.0006868	0	0
ZIC4	0.0027856	0	0
ZIC5	0.0046941	0	0
ZIK1	0.0089548	0	0.0089548
ZIM2	0.0019421	0	0.0019421
ZIM3	0.0008722	0	0
ZKSCAN1	0.0686132	0	0
ZKSCAN2	0.0231571	0	0.0231571
ZKSCAN3	0.0759711	0	0.0759711
ZKSCAN4	0.0510166	0	0.0510166
ZKSCAN5	0.0382437	0	0
ZMAT1	0.0076378	0	0
ZMAT2	0.0890516	0.0890516	0
ZMAT3	0.1465417	0	0.1465417
ZMAT4	0.0023814	0	0.0023814
ZMAT5	0.0202959	0	0
ZMIZ1	0.0230961	0	0.0230961
ZMIZ2	0.0167583	0.0167583	0
ZMPSTE24	0.0846316	0	0.0846316
ZMYM1	0.0273528	0	0.0273528
ZMYM2	0.0264498	0.0264498	0
ZMYM3	0.0041785	0.0041785	0
ZMYM4	0.0399774	0	0.0399774
ZMYM5	0.048286	0	0

ZMYM6	0.0716755	0	0.0716755
ZMYND10	0.01879	0.01879	0
ZMYND11	0.0260009	0	0.0260009
ZMYND12	0.0621544	0	0
ZMYND15	0.0121521	0	0
ZMYND17	0.0185093	0.0185093	0
ZMYND19	0.0036429	0.0036429	0
ZMYND8	0.8599506	0.8599506	0
ZNF10	0.017762	0	0
ZNF100	0.0094218	0	0.0094218
ZNF101	0.0285853	0	0.0285853
ZNF107	0.0256916	0	0.0256916
ZNF114	0.0346948	0	0.0346948
ZNF117	0.0124319	0.0124319	0
ZNF12	0.0106749	0	0
ZNF121	0.0240495	0	0
ZNF124	0.0338586	0	0.0338586
ZNF131	0.0610166	0	0.0610166
ZNF132	0.0071872	0	0.0071872
ZNF133	0.03127	0.03127	0
ZNF134	0.0194715	0	0.0194715
ZNF135	0.0005745	0	0.0005745
ZNF136	0.0138838	0	0.0138838
ZNF137	0.0045722	0	0
ZNF138	0.0159117	0.0159117	0
ZNF14	0.0047379	0	0.0047379
ZNF140	0.0367446	0	0
ZNF141	0.0416469	0	0
ZNF142	0.0172386	0	0
ZNF143	0.1380518	0	0.1380518
ZNF146	0.0381108	0	0
ZNF148	0.0627138	0	0.0627138
ZNF154	0.0019874	0	0.0019874
ZNF155	0.0239079	0	0
ZNF157	0.0050612	0	0.0050612
ZNF16	0.0063809	0.0063809	0
ZNF160	0.0050709	0	0
ZNF165	0.079722	0.079722	0
ZNF167	0.0047095	0	0.0047095
ZNF169	0.0117348	0	0
ZNF17	0.0100138	0	0.0100138
ZNF174	0.0278284	0	0
ZNF175	0.0510259	0	0.0510259
ZNF177	0.0096478	0	0
ZNF18	0.0193803	0	0.0193803

ZNF180	0.0097754	0	0.0097754
ZNF181	0.0687644	0	0.0687644
ZNF182	0.0073802	0	0
ZNF184	0.2289516	0	0.2289516
ZNF185	0.0005896	0	0
ZNF187	0.0805687	0	0.0805687
ZNF189	0.0856395	0	0.0856395
ZNF19	0.0231928	0.0231928	0
ZNF192	0.1175773	0	0.1175773
ZNF193	0.0983967	0.0983967	0
ZNF195	0.045614	0.045614	0
ZNF197	0.1565324	0	0.1565324
ZNF2	0.0342883	0	0.0342883
ZNF20	0.0178897	0	0
ZNF200	0.0255041	0	0.0255041
ZNF202	0.017982	0	0
ZNF204P	0.0150103	0.0150103	0
ZNF205	0.0075174	0.0075174	0
ZNF207	0.1147491	0	0
ZNF208	0.001376	0	0.001376
ZNF211	0.015849	0	0
ZNF212	0.0265304	0.0265304	0
ZNF213	0.0221191	0.0221191	0
ZNF214	0.0103297	0.0103297	0
ZNF215	0.0210711	0.0210711	0
ZNF217	0.8082721	0	0.8082721
ZNF219	0.1716312	0.1716312	0
ZNF22	0.0294588	0	0.0294588
ZNF221	0.0578913	0	0.0578913
ZNF222	0.0118477	0	0.0118477
ZNF223	0.0107883	0.0107883	0
ZNF224	0.0260078	0	0
ZNF225	0.0512623	0	0.0512623
ZNF226	0.0734787	0.0734787	0
ZNF227	0.1548935	0	0.1548935
ZNF229	0.0048661	0	0
ZNF23	0.0215926	0	0
ZNF230	0.0484993	0	0.0484993
ZNF232	0.0014077	0	0
ZNF233	0.0106694	0.0106694	0
ZNF234	0.0247205	0	0.0247205
ZNF235	0.0282297	0	0.0282297
ZNF236	0.0217528	0	0.0217528
ZNF238	0.0982026	0.0982026	0
ZNF239	0.0161473	0	0

ZNF24	0.0845191	0	0.0845191
ZNF248	0.0237664	0	0
ZNF25	0.0296498	0	0.0296498
ZNF250	0.0311972	0	0
ZNF251	0.0263787	0	0
ZNF252	0.0380091	0.0380091	0
ZNF253	0.0103249	0	0.0103249
ZNF254	0.0131798	0	0.0131798
ZNF256	0.0015608	0	0.0015608
ZNF257	0.0014117	0	0.0014117
ZNF259	0.0600538	0.0600538	0
ZNF26	0.0224471	0.0224471	0
ZNF260	0.0360909	0	0.0360909
ZNF263	0.0330507	0.0330507	0
ZNF264	0.0072272	0	0.0072272
ZNF266	0.0129266	0.0129266	0
ZNF267	0.2416833	0	0.2416833
ZNF268	0.0185248	0	0.0185248
ZNF271	0.1558601	0	0.1558601
ZNF273	0.0279953	0.0279953	0
ZNF274	0.0076872	0	0
ZNF275	0.0070394	0	0.0070394
ZNF276	0.1905833	0.1905833	0
ZNF277	0.1535899	0.1535899	0
ZNF28	0.001449	0	0
ZNF280A	0.0024934	0	0.0024934
ZNF280B	0.0040358	0	0
ZNF280C	0.0243681	0	0.0243681
ZNF280D	0.0886983	0	0.0886983
ZNF281	0.6481804	0	0.6481804
ZNF282	0.0390553	0.0390553	0
ZNF283	0.0123582	0	0.0123582
ZNF284	0.0181932	0	0.0181932
ZNF286A	0.0041707	0	0.0041707
ZNF286B	0.0007631	0	0.0007631
ZNF287	0.0075462	0	0.0075462
ZNF292	0.2065848	0	0.2065848
ZNF295	0.0421092	0	0.0421092
ZNF296	0.0097634	0.0097634	0
ZNF3	0.0392264	0.0392264	0
ZNF30	0.0074916	0	0
ZNF300	0.0435634	0	0
ZNF302	0.0554451	0	0
ZNF304	0.0175621	0	0.0175621
ZNF317	0.0313705	0	0.0313705

ZNF318	0.0708139	0	0.0708139
ZNF319	0.0186455	0	0.0186455
ZNF32	0.0080884	0	0
ZNF320	0.0036065	0.0036065	0
ZNF321	0.010578	0.010578	0
ZNF322A	0.031081	0	0.031081
ZNF322B	0.0007848	0	0.0007848
ZNF323	0.0789847	0	0
ZNF324	0.0238135	0	0
ZNF324B	0.0069705	0	0
ZNF326	0.2342889	0	0
ZNF329	0.0019202	0	0.0019202
ZNF330	0.0642629	0	0.0642629
ZNF331	0.0127877	0	0.0127877
ZNF333	0.0206741	0	0.0206741
ZNF334	0.0125203	0	0
ZNF335	0.0984792	0.0984792	0
ZNF337	0.0722417	0.0722417	0
ZNF33A	0.0227959	0	0
ZNF33B	0.1176026	0.1176026	0
ZNF34	0.0488266	0	0
ZNF341	0.0400143	0.0400143	0
ZNF343	0.0045976	0	0
ZNF345	0.0106546	0	0.0106546
ZNF346	0.1073505	0	0.1073505
ZNF347	0.0022119	0	0.0022119
ZNF35	0.034937	0	0.034937
ZNF350	0.0361379	0	0
ZNF354A	0.0314509	0	0.0314509
ZNF354B	0.0315681	0	0
ZNF354C	0.0038178	0	0.0038178
ZNF358	0.0083988	0.0083988	0
ZNF362	0.0114351	0	0.0114351
ZNF365	0.0455499	0	0.0455499
ZNF366	0.0265597	0	0.0265597
ZNF367	0.0909966	0	0
ZNF37A	0.0142176	0	0.0142176
ZNF37B	0.0300319	0	0
ZNF382	0.0060951	0	0.0060951
ZNF383	0.0245594	0	0.0245594
ZNF384	0.0930907	0.0930907	0
ZNF385A	0.0595813	0	0.0595813
ZNF385B	0.0121956	0	0.0121956
ZNF385D	0.0125238	0	0.0125238
ZNF389	0.0279346	0.0279346	0

ZNF391	0.0141347	0	0.0141347
ZNF394	0.037166	0.037166	0
ZNF395	0.0686089	0.0686089	0
ZNF396	0.0275709	0	0
ZNF397	0.0711239	0	0
ZNF397OS	0.1467032	0	0
ZNF398	0.0315725	0	0
ZNF404	0.0085203	0	0
ZNF407	0.010394	0	0.010394
ZNF408	0.0364172	0.0364172	0
ZNF41	0.0221027	0	0.0221027
ZNF410	0.2285972	0	0.2285972
ZNF414	0.0036832	0.0036832	0
ZNF415	0.0018468	0	0.0018468
ZNF416	0.0190397	0	0
ZNF417	0.0041342	0	0.0041342
ZNF418	0.0028441	0	0.0028441
ZNF419	0.0250369	0.0250369	0
ZNF420	0.0262512	0	0.0262512
ZNF423	0.0018718	0	0.0018718
ZNF425	0.0220873	0	0
ZNF426	0.0063063	0	0.0063063
ZNF428	0.005552	0	0
ZNF429	0.0095598	0	0
ZNF43	0.0021384	0	0.0021384
ZNF430	0.0292396	0	0.0292396
ZNF431	0.0372335	0	0.0372335
ZNF432	0.0119087	0.0119087	0
ZNF433	0.0032975	0	0
ZNF434	0.0282784	0	0
ZNF436	0.0568338	0	0.0568338
ZNF438	0.0381272	0	0.0381272
ZNF439	0.0050503	0	0.0050503
ZNF44	0.0144212	0.0144212	0
ZNF440	0.0083389	0.0083389	0
ZNF441	0.0172367	0	0.0172367
ZNF442	0.0031782	0	0.0031782
ZNF443	0.0104691	0.0104691	0
ZNF444	0.0110842	0.0110842	0
ZNF445	0.0275406	0	0.0275406
ZNF446	0.0412706	0.0412706	0
ZNF449	0.0249223	0	0.0249223
ZNF45	0.0317363	0	0.0317363
ZNF451	0.0437493	0	0.0437493
ZNF454	0.0031064	0	0.0031064

ZNF460	0.7121283	0	0.7121283
ZNF461	0.0121252	0	0.0121252
ZNF462	0.0811547	0	0
ZNF467	0.0008799	0	0
ZNF468	0.0042166	0.0042166	0
ZNF469	0.0002514	0	0.0002514
ZNF470	0.0033144	0	0.0033144
ZNF471	0.0014847	0	0.0014847
ZNF473	0.9812932	0.9812932	0
ZNF474	0.2463383	0	0.2463383
ZNF479	0.0012161	0	0
ZNF48	0.0072181	0.0072181	0
ZNF480	0.0111698	0	0
ZNF483	0.0074445	0	0.0074445
ZNF484	0.3746713	0	0.3746713
ZNF485	0.0113496	0	0
ZNF486	0.0010417	0	0.0010417
ZNF487	0.00877	0	0.00877
ZNF488	0.0097279	0.0097279	0
ZNF490	0.0314818	0	0.0314818
ZNF491	0.0151639	0	0.0151639
ZNF492	0.0007944	0	0.0007944
ZNF493	0.0102788	0	0
ZNF496	0.0398124	0	0.0398124
ZNF497	0.0075213	0.0075213	0
ZNF498	0.0188627	0	0.0188627
ZNF500	0.0123707	0	0
ZNF501	0.0152847	0	0.0152847
ZNF502	0.0085752	0	0
ZNF503	0.0080266	0	0
ZNF506	0.0109795	0	0
ZNF507	0.0437731	0	0
ZNF510	0.0409775	0	0.0409775
ZNF511	0.0080362	0.0080362	0
ZNF512	0.0437075	0	0
ZNF512B	0.0031872	0	0
ZNF513	0.0251752	0.0251752	0
ZNF514	0.0192789	0.0192789	0
ZNF516	0.0461911	0	0.0461911
ZNF517	0.0115744	0.0115744	0
ZNF518A	0.0873176	0.0873176	0
ZNF518B	0.0065618	0	0.0065618
ZNF519	0.0032571	0	0
ZNF521	0.0109833	0	0.0109833
ZNF524	0.0866529	0.0866529	0

ZNF525	0.0043514	0	0
ZNF526	0.009998	0.009998	0
ZNF527	0.0239113	0	0.0239113
ZNF528	0.0027335	0	0
ZNF529	0.0228392	0	0.0228392
ZNF530	0.010265	0	0
ZNF532	0.0219687	0	0.0219687
ZNF534	0.0014644	0	0
ZNF536	0.0014397	0	0.0014397
ZNF540	0.0923888	0	0.0923888
ZNF541	0.0024225	0	0
ZNF542	0.0053761	0	0.0053761
ZNF543	0.0244786	0	0.0244786
ZNF544	0.0057494	0	0
ZNF546	0.0795167	0	0.0795167
ZNF547	0.0171181	0	0
ZNF548	0.0289426	0	0.0289426
ZNF549	0.0096294	0	0.0096294
ZNF550	0.0083636	0	0
ZNF551	0.014748	0	0.014748
ZNF552	0.0187828	0	0
ZNF554	0.0034339	0	0
ZNF555	0.0047621	0	0.0047621
ZNF556	0.0029465	0	0.0029465
ZNF557	0.0089459	0	0
ZNF558	0.0411016	0	0
ZNF559	0.0092781	0	0.0092781
ZNF560	0.0015299	0	0
ZNF561	0.0236606	0	0
ZNF562	0.0182238	0	0
ZNF563	0.0196239	0	0
ZNF564	0.0214228	0.0214228	0
ZNF565	0.0453175	0.0453175	0
ZNF566	0.0277483	0	0.0277483
ZNF567	0.0121535	0	0.0121535
ZNF568	0.0056134	0	0.0056134
ZNF569	0.0196734	0	0.0196734
ZNF57	0.0061122	0.0061122	0
ZNF570	0.0157413	0	0.0157413
ZNF571	0.0751294	0	0.0751294
ZNF572	0.0265797	0.0265797	0
ZNF573	0.0189322	0	0.0189322
ZNF574	0.0121779	0.0121779	0
ZNF575	0.1463504	0	0
ZNF576	0.0164493	0.0164493	0

ZNF577	0.005057	0	0.005057
ZNF578	0.0022081	0	0.0022081
ZNF579	0.0122724	0.0122724	0
ZNF580	0.0324844	0.0324844	0
ZNF581	0.0230093	0.0230093	0
ZNF582	0.0051267	0	0.0051267
ZNF583	0.0040178	0	0.0040178
ZNF584	0.0135955	0	0
ZNF585A	0.0215253	0	0.0215253
ZNF585B	0.0530089	0	0.0530089
ZNF586	0.0112295	0	0.0112295
ZNF587	0.0127677	0.0127677	0
ZNF589	0.0380924	0	0.0380924
ZNF592	0.0339707	0	0.0339707
ZNF593	0.0041453	0.0041453	0
ZNF594	0.025036	0	0
ZNF595	1	0	1
ZNF596	0.0112866	0	0.0112866
ZNF597	0.0137676	0	0.0137676
ZNF598	0.1188276	0.1188276	0
ZNF599	0.0130781	0	0
ZNF600	0.0049577	0.0049577	0
ZNF605	0.0047609	0	0
ZNF606	0.0034037	0	0
ZNF607	0.0110612	0	0
ZNF608	0.4459086	0	0
ZNF609	0.0288919	0	0.0288919
ZNF610	0.0016316	0	0.0016316
ZNF611	0.0110141	0	0.0110141
ZNF613	0.0322108	0	0.0322108
ZNF614	0.0365934	0	0.0365934
ZNF615	0.0159848	0	0.0159848
ZNF616	0.0145073	0	0
ZNF618	0.0051657	0	0
ZNF619	0.0151578	0	0.0151578
ZNF620	0.0449262	0.0449262	0
ZNF621	0.01712	0	0.01712
ZNF622	0.1674784	0.1674784	0
ZNF623	0.0229586	0	0
ZNF624	0.026296	0	0.026296
ZNF625	0.0100573	0	0.0100573
ZNF626	0.0020953	0	0.0020953
ZNF627	0.0178243	0	0
ZNF628	0.0085016	0.0085016	0
ZNF629	0.0068441	0.0068441	0

ZNF630	0.0025828	0.0025828	0
ZNF638	0.0591091	0	0
ZNF639	0.0846875	0	0.0846875
ZNF641	0.0616112	0	0.0616112
ZNF642	0.0070981	0	0.0070981
ZNF643	0.0302102	0	0.0302102
ZNF644	0.1157224	0	0.1157224
ZNF645	0.012445	0.012445	0
ZNF646	0.0389603	0	0.0389603
ZNF648	0.0091154	0	0
ZNF649	0.0092265	0	0.0092265
ZNF652	0.0387355	0	0
ZNF653	0.0044514	0.0044514	0
ZNF654	0.0387712	0	0.0387712
ZNF655	0.0819439	0	0
ZNF658	0.0038676	0	0.0038676
ZNF660	0.0062166	0	0.0062166
ZNF662	0.0042744	0	0
ZNF664	0.0302534	0.0302534	0
ZNF665	0.0011277	0	0.0011277
ZNF667	0.0015604	0	0.0015604
ZNF668	0.0378817	0	0.0378817
ZNF669	0.0716223	0	0
ZNF670	0.1171388	0	0.1171388
ZNF671	0.0025305	0	0.0025305
ZNF672	0.1952918	0.1952918	0
ZNF673	0.0125168	0	0.0125168
ZNF674	0.0221787	0	0.0221787
ZNF675	0.0115004	0	0.0115004
ZNF676	0.0028641	0	0.0028641
ZNF677	0.0026333	0	0.0026333
ZNF678	0.0867446	0	0.0867446
ZNF679	0.0016084	0	0
ZNF680	0.0181632	0	0
ZNF681	0.0026793	0	0.0026793
ZNF682	0.0047734	0	0
ZNF683	0.0989274	0	0.0989274
ZNF684	0.0361497	0	0.0361497
ZNF687	0.0778024	0.0778024	0
ZNF688	0.0637463	0.0637463	0
ZNF689	0.0247801	0	0.0247801
ZNF69	0.0023752	0.0023752	0
ZNF691	0.0286457	0	0
ZNF692	0.0640966	0.0640966	0
ZNF695	0.0416053	0	0

ZNF696	0.0085198	0.0085198	0
ZNF697	0.0537517	0	0.0537517
ZNF699	0.0068984	0	0.0068984
ZNF7	0.0084388	0.0084388	0
ZNF70	0.0031493	0	0.0031493
ZNF700	0.0439375	0.0439375	0
ZNF701	0.0067988	0	0.0067988
ZNF702P	0.0028764	0	0
ZNF703	0.0058751	0.0058751	0
ZNF704	0.0119867	0	0.0119867
ZNF705A	0.0005112	0	0
ZNF705D	1.55E-05	0	0
ZNF706	0.1556438	0.1556438	0
ZNF707	0.0055699	0.0055699	0
ZNF708	0.0136278	0	0.0136278
ZNF709	0.0075275	0	0.0075275
ZNF71	0.0036128	0	0.0036128
ZNF710	0.0667362	0	0
ZNF711	0.0086464	0.0086464	0
ZNF713	0.0113353	0	0
ZNF714	0.0135847	0	0
ZNF716	0.0007999	0.0007999	0
ZNF717	0.0125035	0	0
ZNF718	1	0	1
ZNF720	0.0241524	0	0.0241524
ZNF721	0.0109856	0.0109856	0
ZNF727	0.0013625	0	0.0013625
ZNF732	0.0022833	0	0.0022833
ZNF735	0.0017208	0.0017208	0
ZNF737	0.0039176	0	0.0039176
ZNF738	0.008269	0	0.008269
ZNF74	0.0129665	0	0
ZNF740	0.0567214	0	0.0567214
ZNF746	0.008552	0	0
ZNF747	0.0597229	0	0
ZNF749	0.0148509	0.0148509	0
ZNF750	0.0087454	0	0.0087454
ZNF75A	0.0147777	0	0
ZNF75D	0.0044405	0	0
ZNF76	0.050928	0	0
ZNF761	0.0043279	0.0043279	0
ZNF763	0.0107319	0	0
ZNF764	0.0243789	0.0243789	0
ZNF765	0.0054797	0	0
ZNF766	0.0096659	0	0

ZNF767	0.0122108	0.0122108	0
ZNF768	0.027569	0.027569	0
ZNF77	0.0310282	0.0310282	0
ZNF770	0.0927434	0	0.0927434
ZNF771	0.0191447	0.0191447	0
ZNF772	0.0104211	0	0
ZNF773	0.0078987	0	0.0078987
ZNF774	0.0449221	0.0449221	0
ZNF775	0.0755248	0.0755248	0
ZNF776	0.0189778	0	0
ZNF777	0.0118198	0.0118198	0
ZNF778	0.0208808	0	0.0208808
ZNF780A	0.0266105	0	0
ZNF780B	0.0369173	0	0
ZNF781	0.0049749	0	0.0049749
ZNF782	0.0730056	0	0
ZNF784	0.0126838	0.0126838	0
ZNF785	0.0368054	0.0368054	0
ZNF786	0.0087727	0	0
ZNF787	0.1130191	0.1130191	0
ZNF788	0.0039701	0	0.0039701
ZNF789	0.0283361	0.0283361	0
ZNF79	0.0560332	0	0.0560332
ZNF790	0.0078395	0	0.0078395
ZNF791	0.0346228	0	0.0346228
ZNF792	0.0113633	0	0
ZNF793	0.0039389	0	0.0039389
ZNF799	0.0236478	0	0
ZNF8	0.0240573	0	0
ZNF80	0.0038187	0	0.0038187
ZNF800	0.0508916	0	0.0508916
ZNF804A	0.010215	0	0.010215
ZNF804B	0.0127996	0	0.0127996
ZNF805	0.0106583	0	0
ZNF808	0.0084083	0	0
ZNF81	0.0408662	0	0.0408662
ZNF813	0.0018981	0	0.0018981
ZNF814	0.0019799	0	0
ZNF815	0.0092423	0	0
ZNF816A	0.0089919	0.0089919	0
ZNF821	0.0184402	0	0
ZNF823	0.0236587	0.0236587	0
ZNF826	0.0017251	0	0.0017251
ZNF827	0.0403569	0	0.0403569
ZNF828	0.4079132	0	0.4079132

ZNF829	0.0057818	0	0.0057818
ZNF83	0.0342264	0.0342264	0
ZNF830	0.0285212	0	0
ZNF831	0.0026066	0	0.0026066
ZNF833	0.0025534	0	0.0025534
ZNF835	0.0005221	0	0.0005221
ZNF836	0.005719	0	0
ZNF837	0.0376907	0.0376907	0
ZNF839	0.0492971	0.0492971	0
ZNF84	0.0169084	0	0
ZNF841	0.0155482	0.0155482	0
ZNF843	0.0200904	0	0.0200904
ZNF844	0.0025448	0	0.0025448
ZNF845	0.0041873	0	0.0041873
ZNF846	0.0061561	0	0
ZNF85	0.0064661	0	0.0064661
ZNF853	0.0010437	0	0.0010437
ZNF860	0.0552572	0.0552572	0
ZNF862	0.0227782	0	0
ZNF876P	0.0020997	0	0.0020997
ZNF878	0.0025997	0	0
ZNF879	0.0043605	0	0.0043605
ZNF880	0.0041392	0	0.0041392
ZNF883	0.0054136	0	0.0054136
ZNF90	0.0018395	0	0
ZNF91	0.0201622	0	0
ZNF92	0.0425604	0	0
ZNF93	0.0166201	0	0.0166201
ZNF98	0.001066	0	0.001066
ZNF99	0.0020441	0	0.0020441
ZNFX1	0.709589	0	0.709589
ZNHIT1	0.0060621	0.0060621	0
ZNHIT2	0.0815492	0.0815492	0
ZNHIT3	0.0219571	0.0219571	0
ZNHIT6	0.0633783	0	0.0633783
ZNRF1	0.0569495	0.0569495	0
ZNRF2	0.4111155	0.4111155	0
ZNRF3	0.0098992	0	0
ZNRF4	0.001509	0.001509	0
ZP1	0.0041076	0	0
ZP2	0.0055867	0	0
ZP3	0.0314301	0.0314301	0
ZP4	0.0253379	0	0.0253379
ZPBP	0.0140804	0	0
ZPBP2	0.0784062	0	0.0784062

ZPLD1	0.0141216	0	0.0141216
ZRANB1	0.0372816	0	0.0372816
ZRANB2	0.1055634	0	0
ZRANB3	0.0793721	0	0.0793721
ZRSR2	0.0358306	0	0
ZSCAN1	0.0003479	0	0
ZSCAN10	0.0059979	0	0
ZSCAN12	0.0629495	0	0.0629495
ZSCAN16	0.0400968	0.0400968	0
ZSCAN18	0.0020399	0	0.0020399
ZSCAN2	0.0468379	0	0.0468379
ZSCAN20	0.0274712	0	0
ZSCAN21	0.0227759	0.0227759	0
ZSCAN22	0.0355866	0	0
ZSCAN23	0.0208751	0	0.0208751
ZSCAN29	0.0367554	0	0
ZSCAN4	0.002478	0.002478	0
ZSCAN5A	0.0025626	0	0
ZSCAN5B	0.0023577	0	0
ZSWIM1	0.0224937	0.0224937	0
ZSWIM2	0.0185854	0	0
ZSWIM3	0.0565469	0	0
ZSWIM4	0.0198641	0	0
ZSWIM5	0.0274806	0	0
ZSWIM6	0.1464787	0	0
ZSWIM7	0.0440724	0	0
ZUFSP	0.0378458	0	0.0378458
ZW10	0.0418785	0	0
ZWILCH	0.2117846	0	0
ZWINT	0.0273178	0.0273178	0
ZXDA	0.026426	0	0.026426
ZXDB	0.0747135	0	0
ZXDC	0.0080021	0	0
ZYG11A	0.0181209	0	0.0181209
ZYG11B	0.0140164	0	0.0140164
ZYX	0.0266525	0	0.0266525
ZZEF1	0.0569232	0	0.0569232
ZZZ3	0.0795958	0	0.0795958

Suppl. Table 3

STAT3 Weight matrices for BASE. G-STAT3 = General STAT3, T-STAT3 = Tumor STAT3, E-ST

AT3 = Environmental STAT3

Suppl. Table 4. Thirty-five identified TFs i

Transcription factor	rank-t-score	scc.exp.purity
STAT2	9.5420112	-0.556275
ZFPM2	10.9167	-0.79257582
LEF1	9.3128754	-0.67205821
TCF4	24.118467	-0.84321795
EGR2	11.831001	-0.6338
PRDM6	7.2548695	-0.60782783
MAFB	34.386056	-0.57136124
CSRP2	7.2324103	-0.56310955
RIN3	6.4936787	-0.66819834
TFEC	13.552766	-0.88673825
PRDM1	9.6817367	-0.69156819
MNDA	26.365273	-0.88734043
HIC1	9.6647691	-0.50596481
LMO2	12.476712	-0.51671669
ERG	12.217281	-0.73579494
STAT3	6.8933067	-0.50291139
LDB2	17.881904	-0.65204393
TRIM22	9.9773759	-0.71023769
MEOX2	7.1379466	-0.75435326
MEF2C	11.420536	-0.82452717
FLI1	6.7660554	-0.8853041
MAF	25.651676	-0.67669473
TCF21	8.5481638	-0.67124751
KLF13	6.6045785	-0.518087
FHL5	8.511923	-0.60691925
STAT5A	10.925869	-0.60045918
POU2AF1	7.8570413	-0.510446
HEYL	15.026978	-0.5510746
FHL1	6.5182143	-0.54920425
HOXD8	9.9505241	-0.70025396
ZNF80	10.010097	-0.62535596
GREB1	6.8394909	-0.52214689
MEFV	6.7137595	-0.75964076
LZTS1	16.698199	-0.66546835
ZNF366	7.4161845	-0.6085832

Suppl. Table 4

Thirty-five identified TFs in analysis. Rank t-score = t-statictic comparing expression of TF ir pancreatic cancer to pancreatic cell lines. SCC exp. Purity = Spearman correlation coefficient expression and tumor purity.

ı primary
of TF

Suppl. Table 5. GSEA results T-STAT3 and E-STAT3 weight profiles

		T-STAT3	
**NAME**	**SIZE**	**ES**	**NES**
REACTOME_PACKAGING_OF_TELOMERE_ENDS	39	0.8982841	1.5945615
REACTOME_AMYLOIDS	55	0.8888156	1.5877569
REACTOME_MEIOTIC_SYNAPSIS	51	0.8858143	1.5769411
REACTOME_RNA_POL_I_PROMOTER_OPENING	50	0.8905287	1.5707911
REACTOME_RNA_POL_I_TRANSCRIPTION	63	0.870779	1.5496132
KEGG_SYSTEMIC_LUPUS_ERYTHEMATOSUS	77	0.865182	1.5477244
REACTOME_MEIOTIC_RECOMBINATION	62	0.8593246	1.5348747
ABBUD_LIF_SIGNALING_1_UP	23	0.8869809	1.5269803
REACTOME_SMAD2_SMAD3_SMAD4_HETEROTRIMER_	15	0.8966956	1.5260038
REACTOME_MEIOSIS	75	0.849294	1.5257642
FUJII_YBX1_TARGETS_UP	20	0.8875659	1.5215025
AMIT_EGF_RESPONSE_40_HELA	16	0.8895316	1.5188824
REACTOME_DEPOSITION_OF_NEW_CENPA_CONTAININ	49	0.8561688	1.5164193
REACTOME_RNA_POL_I_RNA_POL_III_AND_MITOCHON	84	0.8481358	1.5159186
PHONG_TNF_TARGETS_UP	21	0.8778043	1.5137916
NAGASHIMA_EGF_SIGNALING_UP	22	0.8663616	1.5079395
ZHU_CMV_24_HR_DN	20	0.8787738	1.5059758
GEORGANTAS_HSC_MARKERS	26	0.8605532	1.4946517
DAZARD_UV_RESPONSE_CLUSTER_G24	17	0.8756962	1.4911653
REACTOME_TELOMERE_MAINTENANCE	64	0.8402435	1.489729
LIANG_SILENCED_BY_METHYLATION_2	17	0.8721228	1.4850346
NAKAMURA_METASTASIS_MODEL_DN	24	0.8583946	1.4849343
AMIT_SERUM_RESPONSE_60_MCF10A	21	0.8490688	1.472438
RODRIGUES_NTN1_AND_DCC_TARGETS	17	0.8555195	1.4702917
WONG_ENDMETRIUM_CANCER_UP	16	0.8568322	1.4683086
BURTON_ADIPOGENESIS_PEAK_AT_2HR	22	0.8536946	1.4657819
REACTOME_TRANSCRIPTIONAL_ACTIVITY_OF_SMAD2_	19	0.8580276	1.4615966
GRAHAM_CML_QUIESCENT_VS_NORMAL_DIVIDING_U	16	0.8553636	1.4601029
DORN_ADENOVIRUS_INFECTION_24HR_DN	20	0.8463357	1.4599088
CHIBA_RESPONSE_TO_TSA	29	0.834919	1.4468979
DAUER_STAT3_TARGETS_UP	20	0.8450896	1.4456153
HALMOS_CEBPA_TARGETS_UP	16	0.8411979	1.4453778
KEGG_HEMATOPOIETIC_CELL_LINEAGE	17	0.8457719	1.442068
ZHU_CMV_8_HR_DN	19	0.8378131	1.4372654
GRAHAM_NORMAL_QUIESCENT_VS_NORMAL_DIVIDIN	20	0.8433909	1.4371728
REACTOME_CHROMOSOME_MAINTENANCE	88	0.7964599	1.436095
REACTOME_TRANSCRIPTION	138	0.7867876	1.4285266
LIU_VAV3_PROSTATE_CARCINOGENESIS_UP	15	0.8453243	1.4269801
ZHANG_TLX_TARGETS_UP	48	0.8052756	1.4266826

Suppl. Table 5.
GSEA results T-STAT3 and E-STAT3 weight profiles

adaptive immune response (GO:0002250)	602	89	302.26
immune response (GO:0006955)	1848	580	927.87
immune system process (GO:0002376)	2778	1024	1394.82
regulation of immune system process (GO:0002682)	1651	556	828.96
B cell receptor signaling pathway (GO:0050853)	127	4	63.77
regulation of immune response (GO:0050776)	1134	350	569.38
positive regulation of immune system process (GO:0002	1141	355	572.89
complement activation (GO:0006956)	178	16	89.37
complement activation, classical pathway (GO:0006958	161	13	80.84
phagocytosis, recognition (GO:0006910)	106	3	53.22
phagocytosis, engulfment (GO:0006911)	127	7	63.77
humoral immune response mediated by circulating imm	167	16	83.85
positive regulation of cell activation (GO:0050867)	415	93	208.37
regulation of leukocyte activation (GO:0002694)	595	158	298.75
positive regulation of leukocyte activation (GO:0002696	403	90	202.34
regulation of cell activation (GO:0050865)	633	173	317.83
phagocytosis (GO:0006909)	325	65	163.18
cell surface receptor signaling pathway (GO:0007166)	2406	925	1208.04
positive regulation of immune response (GO:0050778)	847	260	425.28
B cell mediated immunity (GO:0019724)	207	31	103.93
positive regulation of lymphocyte activation (GO:005125	360	80	180.75
immunoglobulin mediated immune response (GO:0016	204	31	102.43
plasma membrane invagination (GO:0099024)	136	13	68.29
defense response (GO:0006952)	1331	469	668.29
regulation of lymphocyte activation (GO:0051249)	511	137	256.57
adaptive immune response based on somatic recombin	275	54	138.08
immune response-regulating signaling pathway (GO:00	592	169	297.24
lymphocyte mediated immunity (GO:0002449)	263	51	132.05
immune response-regulating cell surface receptor signa	482	129	242.01
innate immune response (GO:0045087)	760	239	381.59
membrane invagination (GO:0010324)	143	18	71.8
positive regulation of response to stimulus (GO:004858	2435	971	1222.6
immune response-activating signal transduction (GO:00	555	162	278.66
activation of immune response (GO:0002253)	624	190	313.31
regulation of B cell activation (GO:0050864)	207	38	103.93
leukocyte migration (GO:0050900)	375	96	188.29
immune response-activating cell surface receptor signa	447	123	224.44
positive regulation of B cell activation (GO:0050871)	165	26	82.85
regulation of immune effector process (GO:0002697)	465	135	233.47
defense response to other organism (GO:0098542)	940	329	471.97

response to external biotic stimulus (GO:0043207)	1302	486	653.73
regulation of response to stimulus (GO:0048583)	4293	1866	2155.5
response to other organism (GO:0051707)	1300	486	652.72
immune effector process (GO:0002252)	1076	390	540.25
response to biotic stimulus (GO:0009607)	1326	499	665.78
cell migration (GO:0016477)	951	341	477.49
regulation of cell migration (GO:0030334)	847	298	425.28
immunoglobulin production (GO:0002377)	156	29	78.33
endocytosis (GO:0006897)	553	178	277.66
leukocyte mediated immunity (GO:0002443)	760	266	381.59
response to external stimulus (GO:0009605)	2391	999	1200.51
antigen receptor-mediated signaling pathway (GO:005C	295	80	148.12
locomotion (GO:0040011)	1301	505	653.23
response to bacterium (GO:0009617)	691	241	346.95
inflammatory response (GO:0006954)	495	160	248.54
humoral immune response (GO:0006959)	344	100	172.72
production of molecular mediator of immune response	178	39	89.37
regulation of complement activation (GO:0030449)	117	20	58.75
positive regulation of cell migration (GO:0030335)	502	165	252.05
regulation of multicellular organismal process (GO:005	3130	1358	1571.56
cell recognition (GO:0008037)	235	61	117.99
regulation of humoral immune response (GO:0002920)	136	27	68.29
regulation of cell motility (GO:2000145)	907	340	455.4
biological adhesion (GO:0022610)	933	352	468.46
cell motility (GO:0048870)	1064	410	534.23
localization of cell (GO:0051674)	1064	410	534.23
regulation of cytokine production (GO:0001817)	700	251	351.47
cell adhesion (GO:0007155)	927	350	465.44
positive regulation of cell adhesion (GO:0045785)	408	130	204.86
regulation of cell adhesion (GO:0030155)	680	244	341.43
positive regulation of leukocyte cell-cell adhesion (GO:1	218	57	109.46
leukocyte activation (GO:0045321)	918	349	460.92
circulatory system development (GO:0072359)	867	328	435.32
blood vessel development (GO:0001568)	487	165	244.52
receptor-mediated endocytosis (GO:0006898)	236	65	118.49
import into cell (GO:0098657)	675	245	338.91
regulation of locomotion (GO:0040012)	981	379	492.56
cell activation (GO:0001775)	1065	417	534.73
Fc-gamma receptor signaling pathway (GO:0038094)	139	30	69.79
regulation of cellular component movement (GO:00512	991	384	497.58
cardiovascular system development (GO:0072358)	518	179	260.09
positive regulation of multicellular organismal process (	1742	726	874.65
positive regulation of cell motility (GO:2000147)	524	182	263.1
Fc receptor mediated stimulatory signaling pathway (G	137	30	68.79
positive regulation of T cell activation (GO:0050870)	204	54	102.43

cellular response to chemokine (GO:1990869)	90	15	45.19
response to chemokine (GO:1990868)	90	15	45.19
vasculature development (GO:0001944)	508	176	255.06
chemokine-mediated signaling pathway (GO:0070098)	80	12	40.17
regulation of multicellular organismal development (GC	2037	866	1022.77
blood vessel morphogenesis (GO:0048514)	406	134	203.85
Fc-gamma receptor signaling pathway involved in phag	135	30	67.78
immune response-regulating cell surface receptor signa	135	30	67.78
extracellular matrix organization (GO:0030198)	338	107	169.71
positive regulation of cellular component movement (G	540	191	271.13
angiogenesis (GO:0001525)	315	98	158.16
positive regulation of cytokine production (GO:0001819)	454	155	227.95
regulation of T cell activation (GO:0050863)	317	99	159.16
signaling (GO:0023052)	5332	2447	2677.17
positive regulation of cell-cell adhesion (GO:0022409)	256	75	128.54
regulation of leukocyte cell-cell adhesion (GO:1903037)	298	92	149.62
cell chemotaxis (GO:0060326)	208	57	104.44
taxis (GO:0042330)	548	196	275.15
regulation of vasculature development (GO:1901342)	321	102	161.17
negative regulation of multicellular organismal process	1207	489	606.03
cellular response to organic substance (GO:0071310)	2379	1033	1194.49
positive regulation of locomotion (GO:0040017)	556	200	279.17
chemotaxis (GO:0006935)	546	196	274.14
defense response to bacterium (GO:0042742)	327	105	164.19
cytokine-mediated signaling pathway (GO:0019221)	694	261	348.45
lymphocyte activation (GO:0046649)	382	128	191.8
regulation of response to external stimulus (GO:003210	1081	434	542.77
cell communication (GO:0007154)	5437	2508	2729.89
extracellular structure organization (GO:0043062)	383	129	192.3
regulation of developmental process (GO:0050793)	2571	1129	1290.89
lymphocyte migration (GO:0072676)	67	10	33.64
movement of cell or subcellular component (GO:000692	1537	647	771.72
signal transduction (GO:0007165)	5005	2305	2512.99
negative regulation of developmental process (GO:0051	949	379	476.49
second-messenger-mediated signaling (GO:0019932)	335	112	168.2
regulation of cell-cell adhesion (GO:0022407)	398	139	199.83
anatomical structure formation involved in morphogene	884	352	443.85
negative regulation of immune system process (GO:000	447	160	224.44
anatomical structure morphogenesis (GO:0009653)	2145	936	1077
antigen processing and presentation of endogenous ant	22	0	11.05
regulation of angiogenesis (GO:0045765)	290	94	145.61
response to cytokine (GO:0034097)	1114	457	559.33
cellular response to interferon-gamma (GO:0071346)	163	45	81.84
cellular response to cytokine stimulus (GO:0071345)	1024	418	514.15
regulation of leukocyte proliferation (GO:0070663)	225	69	112.97

positive regulation of developmental process (GO:0051(	1361	573	683.35
negative regulation of cell differentiation (GO:0045596)	712	278	357.49
positive regulation of immune effector process (GO:000	216	66	108.45
regulation of leukocyte mediated immunity (GO:000270	203	61	101.93
response to interferon-gamma (GO:0034341)	184	54	92.39
regulation of interferon-gamma production (GO:003264	103	24	51.72
regulation of localization (GO:0032879)	2755	1233	1383.27
regulation of B cell receptor signaling pathway (GO:005	26	1	13.05
regulation of adaptive immune response (GO:0002819)	160	45	80.34
regulation of cell differentiation (GO:0045595)	1814	788	910.8
positive regulation of response to external stimulus (GC	597	229	299.75
leukocyte chemotaxis (GO:0030595)	144	39	72.3
regulation of leukocyte migration (GO:0002685)	196	59	98.41
regulation of cytokine secretion (GO:0050707)	206	63	103.43
cytokine production (GO:0001816)	153	43	76.82
regulation of alpha-beta T cell activation (GO:0046634)	94	21	47.2
hemopoiesis (GO:0030097)	568	217	285.19
leukocyte differentiation (GO:0002521)	341	119	171.21
positive regulation of adaptive immune response based	99	23	49.71
cellular extravasation (GO:0045123)	39	4	19.58
positive regulation of adaptive immune response (GO:0	104	25	52.22
regulation of inflammatory response (GO:0050727)	334	116	167.7
regulation of adaptive immune response based on soma	145	40	72.8
regulation of lymphocyte proliferation (GO:0050670)	213	67	106.95
regulation of mononuclear cell proliferation (GO:00329	214	67	107.45
response to stimulus (GO:0050896)	8428	4020	4231.66
cellular response to molecule of bacterial origin (GO:00	193	59	96.9
bone development (GO:0060348)	202	63	101.42
negative regulation of cell migration (GO:0030336)	259	86	130.04
tube morphogenesis (GO:0035239)	652	256	327.37
hematopoietic or lymphoid organ development (GO:004	617	241	309.79
regulation of tumor necrosis factor superfamily cytokine	151	43	75.82
macrophage activation (GO:0042116)	51	8	25.61
cellular response to lipopolysaccharide (GO:0071222)	186	57	93.39
positive regulation of interferon-gamma production (GC	67	13	33.64
positive regulation of biological process (GO:0048518)	6166	2905	3095.92
mononuclear cell migration (GO:0071674)	47	7	23.6
response to molecule of bacterial origin (GO:0002237)	329	116	165.19
negative regulation of locomotion (GO:0040013)	322	113	161.67
muscle structure development (GO:0061061)	481	182	241.51
regulation of tumor necrosis factor production (GO:003	148	42	74.31
negative regulation of cell activation (GO:0050866)	196	61	98.41
negative regulation of leukocyte activation (GO:000269	175	53	87.87
regulation of signaling (GO:0023051)	3543	1625	1778.92
regulation of defense response (GO:0031347)	763	308	383.1

p-value	FDR q-val	FWER p-val	RANK AT MAX
0	9.98E-04	0.001	530
0	9.97E-04	0.002	530
0	0.0033216	0.01	530
0	0.0042346	0.016	530
0	0.0113588	0.056	1150
0	0.010462	0.061	551
0	0.0142346	0.095	530
0	0.017188	0.129	783
0	0.015389	0.13	312
0	0.0139497	0.13	530
0	0.0153083	0.155	611
0	0.0159423	0.176	910
0	0.0162492	0.192	1395
0	0.0151598	0.193	1275
0	0.0155439	0.21	968
0.001	0.0186824	0.264	881
0	0.019107	0.282	941
0	0.0274006	0.385	416
0	0.0290523	0.422	243
0	0.0287951	0.434	541
0	0.0326906	0.487	697
0	0.0312047	0.487	467
0.003003	0.045487	0.636	1216
0.001002	0.0464147	0.665	783
0.0020121	0.0471882	0.684	666
0	0.0490522	0.713	1152
0.001004	0.0526228	0.755	778
0.0040282	0.0530208	0.769	960
0	0.0515362	0.771	1338
0	0.0735308	0.885	968
0	0.0737302	0.894	558
0.0050302	0.0719554	0.894	713
0.0050302	0.0765681	0.915	676
0.003003	0.0850707	0.939	775
0.004008	0.0830102	0.94	393
0	0.0825034	0.943	1150
0	0.0981804	0.975	1150
0.0090361	0.0992932	0.978	718
0	0.0975137	0.979	706

-	0.29	9.27E-36	1.47E-31
-	0.63	2.18E-26	1.73E-22
-	0.73	4.32E-20	9.82E-17
-	0.67	3.99E-18	6.35E-15
-	0.06	6.61E-18	9.57E-15
-	0.61	2.72E-17	3.09E-14
-	0.62	7.22E-17	7.66E-14
-	0.18	1.30E-16	1.21E-13
-	0.16	5.53E-16	4.89E-13
-	0.06	1.86E-15	1.55E-12
-	0.11	3.67E-15	2.92E-12
-	0.19	4.25E-15	3.22E-12
-	0.45	2.66E-14	1.84E-11
-	0.53	3.14E-14	2.00E-11
-	0.44	5.01E-14	2.85E-11
-	0.54	4.95E-14	2.92E-11
-	0.4	1.04E-13	5.32E-11
-	0.77	1.29E-13	6.21E-11
-	0.61	2.38E-13	1.08E-10
-	0.3	7.37E-13	3.17E-10
-	0.44	9.11E-13	3.81E-10
-	0.3	1.54E-12	6.14E-10
-	0.19	1.53E-12	6.25E-10
-	0.7	4.04E-12	1.53E-09
-	0.53	3.94E-12	1.53E-09
-	0.39	5.06E-12	1.87E-09
-	0.57	7.86E-12	2.84E-09
-	0.39	8.92E-12	3.15E-09
-	0.53	1.48E-11	5.12E-09
-	0.63	3.28E-11	1.09E-08
-	0.25	7.87E-11	2.50E-08
-	0.79	7.76E-11	2.52E-08
-	0.58	1.59E-10	4.88E-08
-	0.61	2.20E-10	6.59E-08
-	0.37	3.45E-10	1.02E-07
-	0.51	4.23E-10	1.22E-07
-	0.55	4.99E-10	1.42E-07
-	0.31	6.10E-10	1.67E-07
-	0.58	4.04E-09	1.05E-06
-	0.7	4.24E-09	1.09E-06

-	0.74	5.19E-09	1.29E-06
-	0.87	5.59E-09	1.37E-06
-	0.74	5.99E-09	1.44E-06
-	0.72	8.22E-09	1.90E-06
-	0.75	8.51E-09	1.93E-06
-	0.71	2.84E-08	5.95E-06
-	0.7	3.82E-08	7.70E-06
-	0.37	7.13E-08	1.40E-05
-	0.64	7.80E-08	1.51E-05
-	0.7	1.45E-07	2.77E-05
-	0.83	1.94E-07	3.66E-05
-	0.54	2.63E-07	4.86E-05
-	0.77	3.10E-07	5.66E-05
-	0.69	4.41E-07	7.79E-05
-	0.64	4.74E-07	8.19E-05
-	0.58	4.74E-07	8.28E-05
-	0.44	4.87E-07	8.33E-05
-	0.34	7.01E-07	1.19E-04
-	0.65	9.25E-07	1.50E-04
-	0.86	1.02E-06	1.63E-04
-	0.52	1.20E-06	1.88E-04
-	0.4	1.63E-06	2.48E-04
-	0.75	1.99E-06	2.91E-04
-	0.75	2.09E-06	3.02E-04
-	0.77	2.28E-06	3.23E-04
-	0.77	2.28E-06	3.26E-04
-	0.71	2.35E-06	3.31E-04
-	0.75	2.60E-06	3.63E-04
-	0.63	3.00E-06	4.07E-04
-	0.71	3.40E-06	4.54E-04
-	0.52	4.46E-06	5.82E-04
-	0.76	4.99E-06	6.40E-04
-	0.75	6.56E-06	8.09E-04
-	0.67	6.80E-06	8.26E-04
-	0.55	6.76E-06	8.27E-04
-	0.72	7.58E-06	9.06E-04
-	0.77	7.77E-06	9.16E-04
-	0.78	8.18E-06	9.49E-04
-	0.43	8.15E-06	9.54E-04
-	0.77	8.67E-06	9.85E-04
-	0.69	9.18E-06	1.04E-03
-	0.83	9.66E-06	1.08E-03
-	0.69	1.05E-05	1.15E-03
-	0.44	1.09E-05	1.15E-03
-	0.53	1.10E-05	1.16E-03

-	0.33	1.07E-05	1.16E-03
-	0.33	1.07E-05	1.16E-03
-	0.69	1.13E-05	1.17E-03
-	0.3	1.15E-05	1.19E-03
-	0.85	1.44E-05	1.45E-03
-	0.66	1.44E-05	1.45E-03
-	0.44	1.47E-05	1.46E-03
-	0.44	1.47E-05	1.47E-03
-	0.63	1.61E-05	1.58E-03
-	0.7	1.78E-05	1.71E-03
-	0.62	1.97E-05	1.84E-03
-	0.68	2.04E-05	1.89E-03
-	0.62	2.11E-05	1.94E-03
-	0.91	2.13E-05	1.95E-03
-	0.58	2.27E-05	2.05E-03
-	0.61	2.37E-05	2.11E-03
-	0.55	2.57E-05	2.27E-03
-	0.71	2.96E-05	2.60E-03
-	0.63	3.14E-05	2.75E-03
-	0.81	3.23E-05	2.81E-03
-	0.86	3.30E-05	2.86E-03
-	0.72	3.41E-05	2.93E-03
-	0.71	3.44E-05	2.94E-03
-	0.64	3.80E-05	3.20E-03
-	0.75	4.48E-05	3.68E-03
-	0.67	4.52E-05	3.69E-03
-	0.8	4.55E-05	3.70E-03
-	0.92	4.71E-05	3.80E-03
-	0.67	5.70E-05	4.47E-03
-	0.87	6.14E-05	4.77E-03
-	0.3	8.10E-05	6.19E-03
-	0.84	8.58E-05	6.47E-03
-	0.92	8.92E-05	6.66E-03
-	0.8	9.82E-05	7.23E-03
-	0.67	1.21E-04	8.86E-03
-	0.7	1.51E-04	1.08E-02
-	0.79	1.53E-04	1.09E-02
-	0.71	1.55E-04	1.09E-02
-	0.87	1.56E-04	1.10E-02
-	< 0.01	1.61E-04	1.13E-02
-	0.65	1.65E-04	1.15E-02
-	0.82	1.70E-04	1.17E-02
-	0.55	2.14E-04	1.45E-02
-	0.81	2.27E-04	1.53E-02
-	0.61	2.35E-04	1.58E-02

-	0.84	2.53E-04	1.69E-02
-	0.78	2.81E-04	1.83E-02
-	0.61	2.86E-04	1.86E-02
-	0.6	2.98E-04	1.91E-02
-	0.58	3.21E-04	2.02E-02
-	0.46	3.21E-04	2.03E-02
-	0.89	3.28E-04	2.05E-02
-	0.08	3.41E-04	2.12E-02
-	0.56	3.48E-04	2.15E-02
-	0.87	3.73E-04	2.30E-02
-	0.76	3.80E-04	2.33E-02
-	0.54	3.80E-04	2.34E-02
-	0.6	3.92E-04	2.39E-02
-	0.61	4.34E-04	2.61E-02
-	0.56	4.59E-04	2.75E-02
-	0.44	4.68E-04	2.79E-02
-	0.76	4.82E-04	2.84E-02
-	0.7	4.80E-04	2.85E-02
-	0.46	4.85E-04	2.85E-02
-	0.2	4.93E-04	2.87E-02
-	0.48	5.02E-04	2.88E-02
-	0.69	5.01E-04	2.89E-02
-	0.55	5.46E-04	3.11E-02
-	0.63	5.75E-04	3.23E-02
-	0.62	5.77E-04	3.23E-02
-	0.95	5.75E-04	3.24E-02
-	0.61	6.03E-04	3.33E-02
-	0.62	6.57E-04	3.62E-02
-	0.66	6.89E-04	3.73E-02
-	0.78	6.88E-04	3.74E-02
-	0.78	7.29E-04	3.93E-02
-	0.57	7.40E-04	3.98E-02
-	0.31	7.81E-04	4.18E-02
-	0.61	7.94E-04	4.23E-02
-	0.39	7.92E-04	4.23E-02
-	0.94	8.10E-04	4.28E-02
-	0.3	8.10E-04	4.29E-02
-	0.7	8.22E-04	4.33E-02
-	0.7	8.59E-04	4.48E-02
-	0.75	8.67E-04	4.49E-02
-	0.57	8.58E-04	4.49E-02
-	0.62	8.65E-04	4.50E-02
-	0.6	8.97E-04	4.63E-02
-	0.91	9.47E-04	4.88E-02
-	0.8	9.74E-04	4.98E-02

		E-STAT3	
**NAME**	**SIZE**	**ES**	**NES**
MURATA_VIRULENCE_OF_H_PILORI	18	0.9127162	1.5622102
REACTOME_AMYLOIDS	47	0.8769679	1.5531404
REACTOME_RNA_POL_I_PROMOTER_OPENING	27	0.8864408	1.5221887
AMIT_SERUM_RESPONSE_40_MCF10A	27	0.8778387	1.5208755
DALESSIO_TSA_RESPONSE	20	0.8897792	1.504981
REACTOME_PACKAGING_OF_TELOMERE_ENDS	23	0.8768678	1.4948015
ZHANG_RESPONSE_TO_CANTHARIDIN_UP	17	0.8876854	1.4909762
PHONG_TNF_TARGETS_UP	54	0.8415276	1.4860927
ABBUD_LIF_SIGNALING_1_UP	35	0.8558502	1.4839661
KEGG_SYSTEMIC_LUPUS_ERYTHEMATOSUS	71	0.8353054	1.4744277
AMIT_EGF_RESPONSE_40_HELA	32	0.8550248	1.4733493
DUTTA_APOPTOSIS_VIA_NFKB	22	0.8564051	1.46828
ONO_FOXP3_TARGETS_UP	19	0.8636978	1.4668331
AMIT_EGF_RESPONSE_60_MCF10A	29	0.8389477	1.4604192
GESERICK_TERT_TARGETS_DN	16	0.8612456	1.4548247
REACTOME_RNA_POL_I_TRANSCRIPTION	40	0.8338847	1.4544529
REACTOME_REGULATION_OF_IFNA_SIGNALING	21	0.8564451	1.4520079
DER_IFN_GAMMA_RESPONSE_UP	50	0.8263179	1.4497129
REACTOME_DEPOSITION_OF_NEW_CENPA_CONTAININ(	29	0.8403838	1.449602
BURTON_ADIPOGENESIS_PEAK_AT_2HR	38	0.8236067	1.4470501
MAHADEVAN_IMATINIB_RESISTANCE_UP	17	0.8542755	1.4446918
SCHLINGEMANN_SKIN_CARCINOGENESIS_TPA_UP	25	0.835412	1.4445021
DAUER_STAT3_TARGETS_UP	39	0.8248235	1.4444224
SUZUKI_RESPONSE_TO_TSA_AND_DECITABINE_1A	20	0.8466033	1.444401
FALVELLA_SMOKERS_WITH_LUNG_CANCER	60	0.8132518	1.4407676
ZWANG_CLASS_3_TRANSIENTLY_INDUCED_BY_EGF	165	0.7980502	1.4381597
BRACHAT_RESPONSE_TO_METHOTREXATE_UP	18	0.8506144	1.4378457
BILBAN_B_CLL_LPL_DN	34	0.8246969	1.4378061
KANNAN_TP53_TARGETS_UP	38	0.8189232	1.4370531
PID_IL27_PATHWAY	25	0.8387151	1.4370517
NAGASHIMA_EGF_SIGNALING_UP	44	0.8207358	1.4358509
REACTOME_INTERFERON_GAMMA_SIGNALING	46	0.8163022	1.4344316
MCDOWELL_ACUTE_LUNG_INJURY_UP	41	0.818434	1.4344164
TIAN_TNF_SIGNALING_NOT_VIA_NFKB	18	0.8396978	1.4337345
DAZARD_UV_RESPONSE_CLUSTER_G2	15	0.8471012	1.4303119
DAIRKEE_CANCER_PRONE_RESPONSE_E2	18	0.8424959	1.4299301
MANTOVANI_NFKB_TARGETS_UP	35	0.8172302	1.4297763
BOWIE_RESPONSE_TO_TAMOXIFEN	18	0.8386605	1.4252769
DIRMEIER_LMP1_RESPONSE_LATE_DN	16	0.8439798	1.4245584
REACTOME_MEIOTIC_SYNAPSIS	43	0.8158723	1.42332
RASHI_RESPONSE_TO_IONIZING_RADIATION_2	88	0.7974777	1.4229465
HUMMERICH_SKIN_CANCER_PROGRESSION_UP	66	0.8074701	1.422804

REACTOME_TELOMERE_MAINTENANCE	32	0.8174235	1.4207743
SCHOEN_NFKB_SIGNALING	33	0.8246312	1.4206938
RASHI_NFKB1_TARGETS	15	0.8446405	1.4204749
HOFMANN_MYELODYSPLASTIC_SYNDROM_LOW_RISK_I	18	0.8353905	1.4194114
LIANG_SILENCED_BY_METHYLATION_2	46	0.8117907	1.4176342
HAN_JNK_SINGALING_DN	34	0.8118976	1.4164621
SIMBULAN_UV_RESPONSE_NORMAL_DN	23	0.8267452	1.414274
SARRIO_EPITHELIAL_MESENCHYMAL_TRANSITION_DN	113	0.7880222	1.4136621
BIOCARTA_IGF1MTOR_PATHWAY	15	0.8365247	1.4129351
DORN_ADENOVIRUS_INFECTION_24HR_DN	27	0.8200346	1.4125706
GARGALOVIC_RESPONSE_TO_OXIDIZED_PHOSPHOLIPIDS	26	0.8143615	1.4119136
REACTOME_RNA_POL_I_RNA_POL_III_AND_MITOCHONI	59	0.7977365	1.4082834
BIOCARTA_CASPASE_PATHWAY	17	0.8274595	1.4058888
KIM_LRRC3B_TARGETS	20	0.8218377	1.4055997
PID_INTEGRIN_A9B1_PATHWAY	17	0.8298323	1.4047103
UZONYI_RESPONSE_TO_LEUKOTRIENE_AND_THROMBIN	34	0.8161576	1.4041536
YAN_ESCAPE_FROM_ANOIKIS	18	0.8275056	1.4040787
MAHAJAN_RESPONSE_TO_IL1A_UP	70	0.7938794	1.4039755
OSADA_ASCL1_TARGETS_DN	21	0.8173939	1.4037354
DIRMEIER_LMP1_RESPONSE_EARLY	51	0.7965531	1.4031633
SEKI_INFLAMMATORY_RESPONSE_LPS_DN	15	0.8329844	1.4006307
BRACHAT_RESPONSE_TO_CISPLATIN	16	0.843152	1.399525
VARELA_ZMPSTE24_TARGETS_UP	26	0.8134152	1.3995123
GARGALOVIC_RESPONSE_TO_OXIDIZED_PHOSPHOLIPIDS	67	0.7887759	1.3988166
REACTOME_MEIOTIC_RECOMBINATION	45	0.800328	1.3987408
PID_HIV_NEF_PATHWAY	23	0.8138363	1.3970717
BIOCARTA_IL22BP_PATHWAY	15	0.8348516	1.397031
VANHARANTA_UTERINE_FIBROID_DN	54	0.7967224	1.396758

complement activation, classical pathway (GO:0006958)	161	25	97.52
humoral immune response mediated by circulating immu	167	27	101.15
phagocytosis, recognition (GO:0006910)	106	14	64.2
oxidative phosphorylation (GO:0006119)	116	18	70.26
complement activation (GO:0006956)	178	40	107.81
ATP synthesis coupled electron transport (GO:0042773)	91	14	55.12
mitochondrial ATP synthesis coupled electron transport (	90	14	54.51
immunoglobulin production (GO:0002377)	156	39	94.49
immunoglobulin mediated immune response (GO:00160	204	59	123.56
B cell mediated immunity (GO:0019724)	207	61	125.38
respiratory electron transport chain (GO:0022904)	111	23	67.23
mitochondrial translation (GO:0032543)	112	24	67.84
mitochondrial translational termination (GO:0070126)	89	16	53.91

phagocytosis, engulfment (GO:0006911)	127	30	76.92
adaptive immune response (GO:0002250)	602	254	364.62
mitochondrial translational elongation (GO:0070125)	88	16	53.3
mitochondrial respiratory chain complex assembly (GO:0	99	20	59.96
membrane invagination (GO:0010324)	143	37	86.61
mitochondrial respiratory chain complex I assembly (GO:	66	9	39.98
NADH dehydrogenase complex assembly (GO:0010257)	66	9	39.98
plasma membrane invagination (GO:0099024)	136	35	82.37
B cell receptor signaling pathway (GO:0050853)	127	32	76.92
mitochondrial gene expression (GO:0140053)	139	37	84.19
translational termination (GO:0006415)	97	21	58.75
production of molecular mediator of immune response ((	178	56	107.81
mitochondrial electron transport, NADH to ubiquinone (G	49	6	29.68
ATP metabolic process (GO:0046034)	205	71	124.17
translational elongation (GO:0006414)	125	36	75.71
mitochondrion organization (GO:0007005)	443	190	268.32
cellular respiration (GO:0045333)	166	55	100.54
lymphocyte mediated immunity (GO:0002449)	263	102	159.3
electron transport chain (GO:0022900)	180	63	109.02
translation (GO:0006412)	390	168	236.22
cellular protein complex disassembly (GO:0043624)	137	45	82.98
mitochondrial transmembrane transport (GO:1990542)	92	26	55.72
peptide biosynthetic process (GO:0043043)	415	185	251.36
mitochondrial ATP synthesis coupled proton transport (G	21	1	12.72
cristae formation (GO:0042407)	31	4	18.78

p-value	FDR q-val	FWER p-val	RANK AT MAX
0	0.0049714	0.005	643
0	0.0079541	0.016	660
0	0.0338067	0.099	660
0	0.0268461	0.105	1105
0.001003	0.0431519	0.194	772
0	0.0541897	0.272	1449
0.0020121	0.0563911	0.32	894
0	0.0586645	0.362	903
0	0.057228	0.396	1037
0	0.072883	0.508	660
0	0.0691498	0.524	1149
0.001	0.0734955	0.584	1443
0.001	0.0709016	0.601	1789
0	0.0788333	0.658	1862
0.006012	0.0895527	0.727	1267
0	0.0852607	0.734	1449
0	0.0867963	0.765	738
0	0.0878847	0.785	1692
0	0.0833115	0.786	1449
0	0.0854602	0.812	1115
0.0020101	0.0865039	0.825	31
0.001001	0.0830239	0.825	1019
0	0.0796303	0.825	1112
0.001001	0.0764781	0.825	398
0	0.0816914	0.857	1248
0	0.0851273	0.874	2002
0.001003	0.0826004	0.876	910
0	0.079828	0.877	1065
0	0.0792013	0.882	783
0	0.0765613	0.882	1563
0	0.0765933	0.887	1869
0	0.0770893	0.895	1160
0	0.0748136	0.895	840
0.005	0.0744556	0.902	1309
0.003012	0.0794305	0.921	1105
0.004004	0.0779975	0.925	1346
0	0.0761583	0.925	1552
0.002	0.0840183	0.954	1440
0.00501	0.0833937	0.959	1261
0	0.0845898	0.966	1449
0	0.0833269	0.967	1665
0	0.0815559	0.967	1108

0	0.0844459	0.973	1449
0	0.0827301	0.973	1659
0.0110331	0.081444	0.974	861
0.005005	0.0821376	0.977	863
0	0.0839863	0.983	1029
0	0.0845773	0.985	1912
0.005	0.0882895	0.988	1583
0	0.0880152	0.989	1183
0.0070281	0.087732	0.989	1798
0.002	0.0871156	0.99	1692
0.001	0.0869165	0.991	1534
0	0.0941264	0.995	1705
0.0120361	0.0985253	0.995	1261
0.00501	0.0975649	0.995	644
0.0090271	0.0979465	0.995	1272
0	0.0973549	0.995	1642
0.0120482	0.0958228	0.995	548
0	0.0944411	0.995	1938
0.003003	0.0935449	0.996	541
0	0.0934794	0.997	1529
0.005015	0.0980087	0.998	703
0.006012	0.0991027	0.999	1443
0.004008	0.0975781	0.999	2054
0	0.0977116	0.999	1480
0	0.0965055	0.999	1861
0.006	0.0990195	1	1314
0.0120846	0.0976566	1	260
0	0.0967587	1	1105

-	0.26	2.64E-13	1.50E-10
-	0.27	2.79E-13	1.53E-10
-	0.22	2.69E-10	8.74E-08
-	0.26	5.35E-10	1.70E-07
-	0.37	6.17E-10	1.92E-07
-	0.25	4.41E-08	9.22E-06
-	0.26	6.75E-08	1.39E-05
-	0.41	1.06E-07	1.98E-05
-	0.48	1.29E-07	2.30E-05
-	0.49	1.74E-07	2.94E-05
-	0.34	2.47E-07	4.01E-05
-	0.35	4.69E-07	7.10E-05
-	0.3	5.07E-07	7.61E-05

-	0.39	5.42E-07	7.91E-05
-	0.7	6.52E-07	9.26E-05
-	0.3	7.42E-07	1.03E-04
-	0.33	8.99E-07	1.21E-04
-	0.43	9.12E-07	1.22E-04
-	0.23	9.95E-07	1.29E-04
-	0.23	9.95E-07	1.30E-04
-	0.42	1.60E-06	1.87E-04
-	0.42	2.27E-06	2.58E-04
-	0.44	2.33E-06	2.61E-04
-	0.36	3.58E-06	3.87E-04
-	0.52	9.44E-06	8.73E-04
-	0.2	1.69E-05	1.47E-03
-	0.57	2.71E-05	2.14E-03
-	0.48	3.98E-05	2.99E-03
-	0.71	4.81E-05	3.45E-03
-	0.55	6.56E-05	4.48E-03
-	0.64	8.85E-05	5.87E-03
-	0.58	1.10E-04	7.08E-03
-	0.71	1.76E-04	1.06E-02
-	0.54	2.19E-04	1.27E-02
-	0.47	3.84E-04	2.03E-02
-	0.74	4.13E-04	2.16E-02
-	0.08	6.12E-04	3.05E-02
-	0.21	7.61E-04	3.71E-02

Suppl. Table 6. Description of 386 utilized STAT3 targets.

Gene	Compartment	Gene Description	Cancer Ge	Cancer So	Mutation ⁻	Translocation Partner
ABCC2	General	ATP binding cassette subfamily C member 2 [Source:HGNC Symbol;Acc:HGN				
ABL2	Envir.			yes	T	ETV6
ABLIM1	Tumor	actin binding LIM protein 1 [Source:HGNC Symbol;Acc:HGNC:78]				
ADCK4	Tumor					
AFF1	Envir.	AF4/FMR2 family member 1 [Source:HGNC Symbol;Acc:HGNC:7135]				
AGTPBP1	Envir.	ATP/GTP binding protein 1 [Source:HGNC Symbol;Acc:HGNC:17258]				
AIG1	Tumor	androgen induced 1 [Source:HGNC Symbol;Acc:HGNC:21607]				
AIM2	Envir.	absent in melanoma 2 [Source:HGNC Symbol;Acc:HGNC:357]				
AKAP2	Envir.	A-kinase anchoring protein 2 [Source:HGNC Symbol;Acc:HGNC:372]				
ALPK1	General	alpha kinase 1 [Source:HGNC Symbol;Acc:HGNC:20917]				
ANKHD1	General	ankyrin repeat and KH domain containing 1 [Source:HGNC Symbol;Acc:HGN				
ANKHD1-E	General	ANKHD1-EIF4EBP3 readthrough [Source:HGNC Symbol;Acc:HGNC:33530]				
ANKRD46	General	ankyrin repeat domain 46 [Source:HGNC Symbol;Acc:HGNC:27229]				
ANXA1	Envir.	annexin A1 [Source:HGNC Symbol;Acc:HGNC:533]				
ANXA2	Tumor	annexin A2 [Source:HGNC Symbol;Acc:HGNC:537]				
ARGLU1	Tumor	arginine and glutamate rich 1 [Source:HGNC Symbol;Acc:HGNC:25482]				
ARID4B	Envir.	AT-rich interaction domain 4B [Source:HGNC Symbol;Acc:HGNC:15550]				
ARID5B	Envir.	AT-rich interaction domain 5B [Source:HGNC Symbol;Acc:HGNC:17362]				
ARL6IP5	Envir.	ADP ribosylation factor like GTPase 6 interacting protein 5 [Source:HGNC Sy				
ARNTL2	General	aryl hydrocarbon receptor nuclear translocator like 2 [Source:HGNC Symbol;				
ARPC5L	Tumor	actin related protein 2/3 complex subunit 5 like [Source:HGNC Symbol;Acc:H				
ASB7	Envir.	ankyrin repeat and SOCS box containing 7 [Source:HGNC Symbol;Acc:HGNC:				
ASPH	Tumor	aspartate beta-hydroxylase [Source:HGNC Symbol;Acc:HGNC:757]				
ASXL1	Envir.	ASXL transcriptional r		yes	F, N, Mis	
ATF3	Envir.	activating transcription factor 3 [Source:HGNC Symbol;Acc:HGNC:785]				
ATF6	Envir.	activating transcription factor 6 [Source:HGNC Symbol;Acc:HGNC:791]				
ATP1A1	Tumor	ATPase Na+/K+ transporting subunit alpha 1 [Source:HGNC Symbol;Acc:HGN				
ATP2B4	Envir.	ATPase plasma membrane Ca2+ transporting 4 [Source:HGNC Symbol;Acc:H				
ATXN1	Envir.	ataxin 1 [Source:HGNC Symbol;Acc:HGNC:10548]				
B4GALT1	Envir.					
BCL3	Tumor	B cell CLL/lymphoma		yes	T	IGH@
BCL6	Envir.	B cell CLL/lymphoma		yes	T, Mis	IG loci, ZNFN1A1, LCP1, PIM1, T
BHLHE40	Tumor	basic helix-loop-helix family member e40 [Source:HGNC Symbol;Acc:HGNC:				
BIRC3	Envir.	baculoviral IAP repea		yes	D, F, N, T	MALT1
BRD8	Tumor	bromodomain containing 8 [Source:HGNC Symbol;Acc:HGNC:19874]				
BRP44	Tumor					
BTG1	Envir.	BTG anti-proliferatio		yes	T	MYC
C10orf26	Envir.					
C10orf78	Tumor					
C11orf54	General	chromosome 11 open reading frame 54 [Source:HGNC Symbol;Acc:HGNC:3(				
C12orf59	Envir.					

C14orf179	Tumor				
C14orf181	Tumor				
C14orf34	Tumor				
C17orf53	Tumor	chromosome 17 open reading frame 53 [Source:HGNC Symbol;Acc:HGNC:2[illegible]			
C17orf62	Envir.				
C19orf61	General				
C1GALT1	Tumor				
C1orf111	Tumor				
C1orf226	Tumor	chromosome 1 open reading frame 226 [Source:HGNC Symbol;Acc:HGNC:3[illegible]			
C1QTNF6	Envir.	C1q and TNF related 6 [Source:HGNC Symbol;Acc:HGNC:14343]			
C1RL	Envir.	complement C1r subcomponent like [Source:HGNC Symbol;Acc:HGNC:2126[illegible]			
C1S	Envir.	complement C1s [Source:HGNC Symbol;Acc:HGNC:1247]			
C20orf199	Tumor				
C2orf18	General				
C5orf27	General				
C7orf40	Tumor				
C9orf30	Envir.				
C9orf68	Tumor				
CASP4	General	caspase 4 [Source:HGNC Symbol;Acc:HGNC:1505]			
CBX5	Envir.	chromobox 5 [Source:HGNC Symbol;Acc:HGNC:1555]			
CCNL1	General	cyclin L1 [Source:HGNC Symbol;Acc:HGNC:20569]			
CD274	Envir.	CD274 molecule [Sou	yes	T	CIITA
CD46	Tumor	CD46 molecule [Source:HGNC Symbol;Acc:HGNC:6953]			
CD55	Tumor	CD55 molecule (Cromer blood group) [Source:HGNC Symbol;Acc:HGNC:266[illegible]			
CD59	General	CD59 molecule (CD59 blood group) [Source:HGNC Symbol;Acc:HGNC:1689]			
CDC42EP4	Tumor	CDC42 effector protein 4 [Source:HGNC Symbol;Acc:HGNC:17147]			
CDC7	Envir.	cell division cycle 7 [Source:HGNC Symbol;Acc:HGNC:1745]			
CEP63	Envir.	centrosomal protein 63 [Source:HGNC Symbol;Acc:HGNC:25815]			
CHD2	General	chromodomain helicase DNA binding protein 2 [Source:HGNC Symbol;Acc:H			
CHEK2	General	checkpoint	yes	F	
CHI3L2	Envir.	chitinase 3 like 2 [Source:HGNC Symbol;Acc:HGNC:1933]			
CHML	General				
CKS1B	Tumor	CDC28 protein kinase regulatory subunit 1B [Source:HGNC Symbol;Acc:HGN[illegible]			
CLIP4	Envir.	CAP-Gly domain containing linker protein family member 4 [Source:HGNC S			
CPNE8	Envir.	copine 8 [Source:HGNC Symbol;Acc:HGNC:23498]			
CREB3L4	Tumor	cAMP responsive element binding protein 3 like 4 [Source:HGNC Symbol;Ac[illegible]			
CSDA	Envir.				
CXCL2	Envir.	C-X-C motif chemokine ligand 2 [Source:HGNC Symbol;Acc:HGNC:4603]			
DCUN1D3	Envir.	defective in cullin neddylation 1 domain containing 3 [Source:HGNC Symbol			
DDB2	General	damage s[illegible]	yes	Mis, N	
DDX23	Tumor	DEAD-box helicase 23 [Source:HGNC Symbol;Acc:HGNC:17347]			
DDX5	Envir.	DEAD-box helicase 5	yes	T	ETV4
DDX59	Envir.	DEAD-box helicase 59 [Source:HGNC Symbol;Acc:HGNC:25360]			
DENND1A	General	DENN domain containing 1A [Source:HGNC Symbol;Acc:HGNC:29324]			
DENND4A	Envir.	DENN domain containing 4A [Source:HGNC Symbol;Acc:HGNC:24321]			

DHRS13	Tumor	dehydrogenase/reductase 13 [Source:HGNC Symbol;Acc:HGNC:28326]				
DICER1	Envir.		yes	yes	Mis F, N	
DIRC2	General	disrupted in renal carcinoma 2 [Source:HGNC Symbol;Acc:HGNC:16628]				
DKFZP686I	Tumor					
DTL	Tumor	denticleless E3 ubiquitin protein ligase homolog [Source:HGNC Symbol;Acc:				
DTX3L	General	deltex E3 ubiquitin ligase 3L [Source:HGNC Symbol;Acc:HGNC:30323]				
DUSP1	Envir.	dual specificity phosphatase 1 [Source:HGNC Symbol;Acc:HGNC:3064]				
DUSP22	Envir.	dual specificity phosphatase 22 [Source:HGNC Symbol;Acc:HGNC:16077]				
DUSP5	Envir.	dual specificity phosphatase 5 [Source:HGNC Symbol;Acc:HGNC:3071]				
EDEM2	General	ER degradation enhancing alpha-mannosidase like protein 2 [Source:HGNC				
EFNA1	Tumor	ephrin A1 [Source:HGNC Symbol;Acc:HGNC:3221]				
EGOT	General	eosinophil granule ontogeny transcript [Source:HGNC Symbol;Acc:HGNC:37				
EIF1	Tumor	eukaryotic translation initiation factor 1 [Source:HGNC Symbol;Acc:HGNC:3				
ENAH	Envir.					
EPAS1	Envir.	endothelial PAS domain protein 1 [Source:HGNC Symbol;Acc:HGNC:3374]				
ERGIC2	Envir.	ERGIC and golgi 2 [Source:HGNC Symbol;Acc:HGNC:30208]				
ERI1	Envir.	exoribonuclease 1 [Source:HGNC Symbol;Acc:HGNC:23994]				
ETS2	Envir.					
ETV6	Envir.	ETS variant 6 [Source	yes		T	NTRK3, RUNX1, PDGFRB, ABL1,
EXT1	Envir.	exostosin	yes		Mis, N, F, S	
FAAH2	Tumor	fatty acid amide hydrolase 2 [Source:HGNC Symbol;Acc:HGNC:26440]				
FAM129B	Tumor	family with sequence similarity 129 member B [Source:HGNC Symbol;Acc:H				
FAM186A	Tumor	family with sequence similarity 186 member A [Source:HGNC Symbol;Acc:H				
FANK1	Tumor	fibronectin type III and ankyrin repeat domains 1 [Source:HGNC Symbol;Acc				
FBXO9	General	F-box protein 9 [Source:HGNC Symbol;Acc:HGNC:13588]				
FGL1	General	fibrinogen like 1 [Source:HGNC Symbol;Acc:HGNC:3695]				
FHL2	Tumor	four and a half LIM domains 2 [Source:HGNC Symbol;Acc:HGNC:3703]				
FKBP5	Envir.	FK506 binding protein 5 [Source:HGNC Symbol;Acc:HGNC:3721]				
FLJ35776	General					
FLOT2	General	flotillin 2 [Source:HGNC Symbol;Acc:HGNC:3758]				
FOS	General					
FOSL2	Envir.					
FTH1	Envir.	ferritin heavy chain 1 [Source:HGNC Symbol;Acc:HGNC:3976]				
G0S2	General	G0/G1 switch 2 [Source:HGNC Symbol;Acc:HGNC:30229]				
GADD45A	Envir.	growth arrest and DNA damage inducible alpha [Source:HGNC Symbol;Acc:I				
GAS5	Tumor	growth arrest specific 5 [Source:HGNC Symbol;Acc:HGNC:16355]				
GBAP1	Tumor	glucosylceramidase beta pseudogene 1 [Source:HGNC Symbol;Acc:HGNC:41				
GBP2	Envir.	guanylate binding protein 2 [Source:HGNC Symbol;Acc:HGNC:4183]				
GCET2	Envir.					
GGPS1	Tumor	geranylgeranyl diphosphate synthase 1 [Source:HGNC Symbol;Acc:HGNC:42				
GMDS	Tumor					
GPRC5A	Tumor	G protein-coupled receptor class C group 5 member A [Source:HGNC Symbo				
GRAMD3	General					
GSDMC	Envir.	gasdermin C [Source:HGNC Symbol;Acc:HGNC:7151]				
GSDMD	Tumor	gasdermin D [Source:HGNC Symbol;Acc:HGNC:25697]				

H3F3B	General	H3 histone family member 3B [Source:HGNC Symbol;Acc:HGNC:4765]		
HDAC7	Envir.	histone deacetylase 7 [Source:HGNC Symbol;Acc:HGNC:14067]		
HEATR1	General	HEAT repeat containing 1 [Source:HGNC Symbol;Acc:HGNC:25517]		
HELB	Envir.	DNA helicase B [Source:HGNC Symbol;Acc:HGNC:17196]		
HIST1H2AI	General	histone cluster 1 H2A family member b [Source:HGNC Symbol;Acc:HGNC:47		
HIST1H2A(	Tumor	histone cluster 1 H2A family member c [Source:HGNC Symbol;Acc:HGNC:47		
HIST1H2AI	General	histone cluster 1 H2A family member d [Source:HGNC Symbol;Acc:HGNC:47		
HIST1H2A(	General	histone cluster 1 H2A family member g [Source:HGNC Symbol;Acc:HGNC:47		
HIST1H2B(	Tumor	histone cluster 1 H2B family member c [Source:HGNC Symbol;Acc:HGNC:47		
HIST1H2BI	Tumor	histone cluster 1 H2B family member d [Source:HGNC Symbol;Acc:HGNC:47		
HIST1H2BI	General	histone cluster 1 H2B family member f [Source:HGNC Symbol;Acc:HGNC:47		
HIST1H2BJ	Tumor	histone cluster 1 H2B family member j [Source:HGNC Symbol;Acc:HGNC:47		
HIST1H3A	General	histone cluster 1 H3 family member a [Source:HGNC Symbol;Acc:HGNC:476		
HIST1H3B	Tumor	histone cluster 1 H3 f yes	Mis	
HIST1H3D	General	histone cluster 1 H3 family member d [Source:HGNC Symbol;Acc:HGNC:476		
HIST1H4A	General	histone cluster 1 H4 family member a [Source:HGNC Symbol;Acc:HGNC:478		
HIST1H4B	Tumor	histone cluster 1 H4 family member b [Source:HGNC Symbol;Acc:HGNC:478		
HIST1H4E	Tumor	histone cluster 1 H4 family member e [Source:HGNC Symbol;Acc:HGNC:479		
HIST1H4H	General	histone cluster 1 H4 family member h [Source:HGNC Symbol;Acc:HGNC:478		
HIST1H4I	General	histone cluster 1 H4 f yes	T	BCL6
HIST1H4K	General	histone cluster 1 H4 family member k [Source:HGNC Symbol;Acc:HGNC:478		
HIST2H2AI	Tumor	histone cluster 2 H2A family member b [Source:HGNC Symbol;Acc:HGNC:20		
HIST2H2A(	Tumor	histone cluster 2 H2A family member c [Source:HGNC Symbol;Acc:HGNC:47		
HIST2H2BI	Tumor	histone cluster 2 H2B family member e [Source:HGNC Symbol;Acc:HGNC:47		
HIVEP2	Envir.	human immunodeficiency virus type I enhancer binding protein 2 [Source:H		
HNRNPA1	General	heterogeneous nuclear ribonucleoprotein A1 [Source:HGNC Symbol;Acc:HGN		
HNRNPU	General	heterogeneous nuclear ribonucleoprotein U [Source:HGNC Symbol;Acc:HGN		
HRH1	General	histamine receptor H1 [Source:HGNC Symbol;Acc:HGNC:5182]		
HSCB	General	HscB mitochondrial iron-sulfur cluster cochaperone [Source:HGNC Symbol;A		
HSD11B1	Envir.	hydroxysteroid 11-beta dehydrogenase 1 [Source:HGNC Symbol;Acc:HGNC:5		
HSPBAP1	Envir.	HSPB1 associated protein 1 [Source:HGNC Symbol;Acc:HGNC:16389]		
HSPD1	Tumor	heat shock protein family D (Hsp60) member 1 [Source:HGNC Symbol;Acc:H		
HSPE1	Tumor	heat shock protein family E (Hsp10) member 1 [Source:HGNC Symbol;Acc:H		
HTA	Envir.			
ICAM1	Envir.	intercellular adhesion molecule 1 [Source:HGNC Symbol;Acc:HGNC:5344]		
ICK	Envir.	intestinal cell kinase [Source:HGNC Symbol;Acc:HGNC:21219]		
ID1	Tumor			
IER5	General	immediate early response 5 [Source:HGNC Symbol;Acc:HGNC:5393]		
IFI16	Envir.	interferon gamma inducible protein 16 [Source:HGNC Symbol;Acc:HGNC:53		
IFNGR1	Envir.	interferon gamma receptor 1 [Source:HGNC Symbol;Acc:HGNC:5439]		
IFT20	Tumor	intraflagellar transport 20 [Source:HGNC Symbol;Acc:HGNC:30989]		
IL20	General	interleukin 20 [Source:HGNC Symbol;Acc:HGNC:6002]		
IL24	Envir.	interleukin 24 [Source:HGNC Symbol;Acc:HGNC:11346]		
IL4R	Envir.	interleukin 4 receptor [Source:HGNC Symbol;Acc:HGNC:6015]		
IL6ST	Envir.	interleukin 6 signal tr yes	O	

INTS10	Envir.	integrator complex subunit 10 [Source:HGNC Symbol;Acc:HGNC:25548]
INTS7	General	integrator complex subunit 7 [Source:HGNC Symbol;Acc:HGNC:24484]
IQGAP1	Envir.	IQ motif containing GTPase activating protein 1 [Source:HGNC Symbol;Acc:I
IRAK4	Envir.	interleukin 1 receptor associated kinase 4 [Source:HGNC Symbol;Acc:HGNC:
IRF1	Envir.	interferon regulatory factor 1 [Source:HGNC Symbol;Acc:HGNC:6116]
IRF2BP2	Tumor	interferon regulatory factor 2 binding protein 2 [Source:HGNC Symbol;Acc:H
IRF9	Envir.	interferon regulatory factor 9 [Source:HGNC Symbol;Acc:HGNC:6131]
ITPKC	General	inositol-trisphosphate 3-kinase C [Source:HGNC Symbol;Acc:HGNC:14897]
JMJD1C	Envir.	jumonji domain containing 1C [Source:HGNC Symbol;Acc:HGNC:12313]
JUNB	General	
KCNJ15	Envir.	potassium voltage-gated channel subfamily J member 15 [Source:HGNC Syı
KIAA0586	Envir.	KIAA0586 [Source:HGNC Symbol;Acc:HGNC:19960]
KIF20A	Tumor	kinesin family member 20A [Source:HGNC Symbol;Acc:HGNC:9787]
KLF5	Tumor	Kruppel like factor 5 [Source:HGNC Symbol;Acc:HGNC:6349]
KLF6	Envir.	Kruppel like factor 6 [Source:HGNC Symbol;Acc:HGNC:2235]
KLF9	Envir.	Kruppel like factor 9 [Source:HGNC Symbol;Acc:HGNC:1123]
KLHDC7B	Envir.	kelch domain containing 7B [Source:HGNC Symbol;Acc:HGNC:25145]
KSR1	Envir.	kinase suppressor of ras 1 [Source:HGNC Symbol;Acc:HGNC:6465]
KYNU	Envir.	kynureninase [Source:HGNC Symbol;Acc:HGNC:6469]
LAMB3	Tumor	laminin subunit beta 3 [Source:HGNC Symbol;Acc:HGNC:6490]
LAMC2	General	laminin subunit gamma 2 [Source:HGNC Symbol;Acc:HGNC:6493]
LAPTM4A	Envir.	lysosomal protein transmembrane 4 alpha [Source:HGNC Symbol;Acc:HGNC
LDHA	Tumor	lactate dehydrogenase A [Source:HGNC Symbol;Acc:HGNC:6535]
LDLR	Tumor	low density lipoprotein receptor [Source:HGNC Symbol;Acc:HGNC:6547]
LEPR	Envir.	leptin receptor [Source:HGNC Symbol;Acc:HGNC:6554]
LEPROT	Envir.	leptin receptor overlapping transcript [Source:HGNC Symbol;Acc:HGNC:294
LIMA1	General	LIM domain and actin binding 1 [Source:HGNC Symbol;Acc:HGNC:24636]
LINS1	Envir.	lines homolog 1 [Source:HGNC Symbol;Acc:HGNC:30922]
LMNA	Tumor	lamin A/C [Source:HGNC Symbol;Acc:HGNC:6636]
LMNB1	General	lamin B1 [Source:HGNC Symbol;Acc:HGNC:6637]
LOC10021	General	
LOC10028	Tumor	
LOC28331	General	
LOC28340	Tumor	
LOC40109	Envir.	
LPXN	Envir.	leupaxin [Source:HGNC Symbol;Acc:HGNC:14061]
LYRM1	General	LYR motif containing 1 [Source:HGNC Symbol;Acc:HGNC:25074]
MALAT1	General	metastasis associated lung adenocarcinoma transcript 1 [Source:HGNC Sym
MAP3K14	Envir.	mitogen-activated protein kinase kinase kinase 14 [Source:HGNC Symbol;Ac
MBNL1	Envir.	muscleblind like splicing regulator 1 [Source:HGNC Symbol;Acc:HGNC:6923]
MDM4	Tumor	yes A
MED16	Tumor	mediator complex subunit 16 [Source:HGNC Symbol;Acc:HGNC:17556]
MON1B	General	
MPV17	Envir.	
MPZL1	Envir.	myelin protein zero like 1 [Source:HGNC Symbol;Acc:HGNC:7226]

MREG	Tumor	melanoregulin [Source:HGNC Symbol;Acc:HGNC:25478]			
MRPL24	Tumor	mitochondrial ribosomal protein L24 [Source:HGNC Symbol;Acc:HGNC:1403			
MT1L	Envir.				
MT2A	Envir.	metallothionein 2A [Source:HGNC Symbol;Acc:HGNC:7406]			
MTHFS	Tumor	methenyltetrahydrofolate synthetase [Source:HGNC Symbol;Acc:HGNC:743			
MUC1	Tumor		yes	T	IGH@
MVP	Tumor	major vault protein [Source:HGNC Symbol;Acc:HGNC:7531]			
MYC	Envir.		yes	A, T	IGK@, BCL5, BCL7A , BTG1, TRA
MYO1G	Envir.	myosin IG [Source:HGNC Symbol;Acc:HGNC:13880]			
NAMPT	Envir.	nicotinamide phosphoribosyltransferase [Source:HGNC Symbol;Acc:HGNC:3			
NBPF1	Envir.	NBPF member 1 [Source:HGNC Symbol;Acc:HGNC:26088]			
NCRNA00	General				
NDRG1	Tumor	N-myc downstream r	yes	T	ERG
NEAT1	Tumor	nuclear paraspeckle assembly transcript 1 [Source:HGNC Symbol;Acc:HGNC			
NEDD4	Envir.				
NEDD4L	Tumor				
NEK6	Envir.	NIMA related kinase 6 [Source:HGNC Symbol;Acc:HGNC:7749]			
NFE2	Envir.				
NFIL3	Envir.				
NFKBIZ	Envir.	NFKB inhibitor zeta [Source:HGNC Symbol;Acc:HGNC:29805]			
NIT1	Tumor	nitrilase 1 [Source:HGNC Symbol;Acc:HGNC:7828]			
NNMT	Envir.	nicotinamide N-methyltransferase [Source:HGNC Symbol;Acc:HGNC:7861]			
NPC1	General	NPC intracellular cholesterol transporter 1 [Source:HGNC Symbol;Acc:HGNC			
NR1D1	Tumor	nuclear receptor subfamily 1 group D member 1 [Source:HGNC Symbol;Acc:			
NR3C1	Envir.	nuclear receptor subfamily 3 group C member 1 [Source:HGNC Symbol;Acc:			
NRD1	Envir.				
NUAK2	Envir.	NUAK family kinase 2 [Source:HGNC Symbol;Acc:HGNC:29558]			
NUF2	Tumor				
NUP107	General	nucleoporin 107 [Source:HGNC Symbol;Acc:HGNC:29914]			
ODC1	Envir.	ornithine decarboxylase 1 [Source:HGNC Symbol;Acc:HGNC:8109]			
ODF3B	Tumor	outer dense fiber of sperm tails 3B [Source:HGNC Symbol;Acc:HGNC:34388]			
OLR1	Envir.	oxidized low density lipoprotein receptor 1 [Source:HGNC Symbol;Acc:HGN			
OPN3	Envir.	opsin 3 [Source:HGNC Symbol;Acc:HGNC:14007]			
OSMR	Envir.	oncostatin M receptor [Source:HGNC Symbol;Acc:HGNC:8507]			
PANX1	Envir.	pannexin 1 [Source:HGNC Symbol;Acc:HGNC:8599]			
PARP9	Envir.	poly(ADP-ribose) polymerase family member 9 [Source:HGNC Symbol;Acc:			
PDCD1LG2	Envir.	programmed cell death 1 ligand 2 [Source:HGNC Symbol;Acc:HGNC:18731]			
PDE4DIP	Envir.	phosphodiesterase 4I	yes	T	PDGFRB
PDE6A	Envir.	phosphodiesterase 6A [Source:HGNC Symbol;Acc:HGNC:8785]			
PFDN2	Tumor	prefoldin subunit 2 [Source:HGNC Symbol;Acc:HGNC:8867]			
PGLYRP4	Envir.	peptidoglycan recognition protein 4 [Source:HGNC Symbol;Acc:HGNC:30015			
PGS1	Envir.	phosphatidylglycerophosphate synthase 1 [Source:HGNC Symbol;Acc:HGNC:			
PHLDB2	Envir.	pleckstrin homology like domain family B member 2 [Source:HGNC Symbol;			
PI3	General	peptidase inhibitor 3 [Source:HGNC Symbol;Acc:HGNC:8947]			
PICALM	Envir.	phosphatidylinositol l	yes	T	MLLT10, MLL

PKM2	Tumor	
PLA2G4E	General	phospholipase A2 group IVE [Source:HGNC Symbol;Acc:HGNC:24791]
PLD1	General	phospholipase D1 [Source:HGNC Symbol;Acc:HGNC:9067]
PLEKHG2	Envir.	pleckstrin homology and RhoGEF domain containing G2 [Source:HGNC Syml
PLSCR1	Envir.	phospholipid scramblase 1 [Source:HGNC Symbol;Acc:HGNC:9092]
PMF1	Tumor	polyamine modulated factor 1 [Source:HGNC Symbol;Acc:HGNC:9112]
PMVK	Tumor	phosphomevalonate kinase [Source:HGNC Symbol;Acc:HGNC:9141]
PPP1R15B	General	protein phosphatase 1 regulatory subunit 15B [Source:HGNC Symbol;Acc:HG
PPP2R5A	Tumor	protein phosphatase 2 regulatory subunit B'alpha [Source:HGNC Symbol;Acc
PRDX2	Tumor	peroxiredoxin 2 [Source:HGNC Symbol;Acc:HGNC:9353]
PSMC6	Tumor	
PTGER4	Envir.	prostaglandin E receptor 4 [Source:HGNC Symbol;Acc:HGNC:9596]
PTP4A1	General	
PTP4A2	Envir.	
PTTG1IP	General	PTTG1 interacting protein [Source:HGNC Symbol;Acc:HGNC:13524]
PUS7L	Envir.	pseudouridylate synthase 7 like [Source:HGNC Symbol;Acc:HGNC:25276]
RAB27A	Envir.	
RAD23B	General	
RAG1AP1	Tumor	
RASSF5	Envir.	Ras association domain family member 5 [Source:HGNC Symbol;Acc:HGNC:
RGS2	Envir.	regulator of G protein signaling 2 [Source:HGNC Symbol;Acc:HGNC:9998]
RHOV	Tumor	ras homolog family member V [Source:HGNC Symbol;Acc:HGNC:18313]
RNF145	General	ring finger protein 145 [Source:HGNC Symbol;Acc:HGNC:20853]
RNF213	Envir.	ring finger protein 213 [Source:HGNC Symbol;Acc:HGNC:14539]
RPL13AP2(	Tumor	ribosomal protein L13a pseudogene 20 [Source:HGNC Symbol;Acc:HGNC:35
RPL28	Tumor	ribosomal protein L28 [Source:HGNC Symbol;Acc:HGNC:10330]
RPL35	Tumor	ribosomal protein L35 [Source:HGNC Symbol;Acc:HGNC:10344]
RPL37	Tumor	ribosomal protein L37 [Source:HGNC Symbol;Acc:HGNC:10347]
RPS16	Tumor	ribosomal protein S16 [Source:HGNC Symbol;Acc:HGNC:10396]
RRM2B	Envir.	ribonucleotide reductase regulatory TP53 inducible subunit M2B [Source:HG
RUNX1	Envir.	runt related transcrip yes T RPL22, MDS1, EVI1, CBFA2T3, CI
S100A10	Tumor	S100 calcium binding protein A10 [Source:HGNC Symbol;Acc:HGNC:10487]
S100A11	Tumor	S100 calcium binding protein A11 [Source:HGNC Symbol;Acc:HGNC:10488]
S100A2	General	S100 calcium binding protein A2 [Source:HGNC Symbol;Acc:HGNC:10492]
SAMSN1	Envir.	
SAT1	Envir.	spermidine/spermine N1-acetyltransferase 1 [Source:HGNC Symbol;Acc:HG
SBNO2	General	strawberry notch homolog 2 [Source:HGNC Symbol;Acc:HGNC:29158]
SCYL3	Tumor	SCY1 like pseudokinase 3 [Source:HGNC Symbol;Acc:HGNC:19285]
SEC22B	Envir.	
SELPLG	Envir.	selectin P ligand [Source:HGNC Symbol;Acc:HGNC:10722]
SEMA4B	Tumor	semaphorin 4B [Source:HGNC Symbol;Acc:HGNC:10730]
9-Sep	General	septin 9 [Source:HGNC Symbol;Acc:HGNC:7323]
SERPINB1	Tumor	serpin family B member 1 [Source:HGNC Symbol;Acc:HGNC:3311]
SFRS2IP	Envir.	
SHC1	Envir.	SHC adaptor protein 1 [Source:HGNC Symbol;Acc:HGNC:10840]

SKAP2	Envir.	src kinase associated phosphoprotein 2 [Source:HGNC Symbol;Acc:HGNC:15		
SLC25A44	General	solute carrier family 25 member 44 [Source:HGNC Symbol;Acc:HGNC:29036		
SLC39A1	Tumor	solute carrier family 39 member 1 [Source:HGNC Symbol;Acc:HGNC:12876]		
SLPI	Tumor	secretory leukocyte peptidase inhibitor [Source:HGNC Symbol;Acc:HGNC:11(		
SMAD3	Tumor	SMAD family member 3 [Source:HGNC Symbol;Acc:HGNC:6769]		
SMG1	Envir.			
SNHG3	Tumor	small nucleolar RNA host gene 3 [Source:HGNC Symbol;Acc:HGNC:10118]		
SNHG3-R(	Envir.			
SNORA18	General			
SNORA40	General			
SNORA8	Tumor			
SNORA9	General			
SOCS3	Envir.	suppressor of cytokine signaling 3 [Source:HGNC Symbol;Acc:HGNC:19391]		
SOD2	Envir.	superoxide dismutase 2 [Source:HGNC Symbol;Acc:HGNC:11180]		
SP1	General	Sp1 transcription factor [Source:HGNC Symbol;Acc:HGNC:11205]		
SQRDL	Tumor			
SRFBP1	Envir.	serum response factor binding protein 1 [Source:HGNC Symbol;Acc:HGNC:2(		
ST5	General	suppression of tumorigenicity 5 [Source:HGNC Symbol;Acc:HGNC:11350]		
STAT1	Envir.	signal transducer and activator of transcription 1 [Source:HGNC Symbol;Acc		
STAT2	Envir.	signal transducer and activator of transcription 2 [Source:HGNC Symbol;Acc		
STAT3	Envir.	signal transducer anc yes	Mis,O	
STK17A	Envir.	serine/threonine kinase 17a [Source:HGNC Symbol;Acc:HGNC:11395]		
STOM	Envir.	stomatin [Source:HGNC Symbol;Acc:HGNC:3383]		
STX5	General	syntaxin 5 [Source:HGNC Symbol;Acc:HGNC:11440]		
TAF1D	Tumor			
TARS	General	threonyl-tRNA synthetase [Source:HGNC Symbol;Acc:HGNC:11572]		
TBC1D2	General	TBC1 domain family member 2 [Source:HGNC Symbol;Acc:HGNC:18026]		
TERC	General	telomerase RNA component [Source:HGNC Symbol;Acc:HGNC:11727]		
TGIF1	Tumor	TGFB induced factor homeobox 1 [Source:HGNC Symbol;Acc:HGNC:11776]		
TIMM9	Tumor	translocase of inner mitochondrial membrane 9 [Source:HGNC Symbol;Acc:		
TIPARP	Envir.	TCDD inducible poly(ADP-ribose) polymerase [Source:HGNC Symbol;Acc:HGI		
TJP2	Tumor	tight junction protein 2 [Source:HGNC Symbol;Acc:HGNC:11828]		
TM4SF1	Tumor	transmembrane 4 L six family member 1 [Source:HGNC Symbol;Acc:HGNC:1		
TM4SF20	General	transmembrane 4 L six family member 20 [Source:HGNC Symbol;Acc:HGNC		
TNFAIP1	Envir.	TNF alpha induced protein 1 [Source:HGNC Symbol;Acc:HGNC:11894]		
TNFAIP8	Envir.	TNF alpha induced protein 8 [Source:HGNC Symbol;Acc:HGNC:17260]		
TNFRSF1A	General	TNF receptor superfamily member 1A [Source:HGNC Symbol;Acc:HGNC:119		
TNIP1	General	TNFAIP3 interacting protein 1 [Source:HGNC Symbol;Acc:HGNC:16903]		
TNPO1	Envir.	transportin 1 [Source:HGNC Symbol;Acc:HGNC:6401]		
TOP1	General	DNA topoisomerase I yes	T	NUP98
TPK1	Envir.	thiamin pyrophosphokinase 1 [Source:HGNC Symbol;Acc:HGNC:17358]		
TPM4	Envir.	tropomyosin 4 [Sourc yes	T	ALK
TRAF3IP2	General	TRAF3 interacting protein 2 [Source:HGNC Symbol;Acc:HGNC:1343]		
TRIB1	General	tribbles pseudokinase 1 [Source:HGNC Symbol;Acc:HGNC:16891]		
TRIM25	General	tripartite motif containing 25 [Source:HGNC Symbol;Acc:HGNC:12932]		

TRIM69	Envir.	tripartite motif containing 69 [Source:HGNC Symbol;Acc:HGNC:17857]
TUFT1	Tumor	tuftelin 1 [Source:HGNC Symbol;Acc:HGNC:12422]
TXNRD1	Envir.	thioredoxin reductase 1 [Source:HGNC Symbol;Acc:HGNC:12437]
TYMP	Envir.	thymidine phosphorylase [Source:HGNC Symbol;Acc:HGNC:3148]
UBC	Tumor	ubiquitin C [Source:HGNC Symbol;Acc:HGNC:12468]
UBE2D3	General	ubiquitin conjugating enzyme E2 D3 [Source:HGNC Symbol;Acc:HGNC:1247
UNK	General	unkempt family zinc finger [Source:HGNC Symbol;Acc:HGNC:29369]
USP15	Envir.	ubiquitin specific peptidase 15 [Source:HGNC Symbol;Acc:HGNC:12613]
USP3	General	ubiquitin specific peptidase 3 [Source:HGNC Symbol;Acc:HGNC:12626]
USP32	Envir.	ubiquitin specific peptidase 32 [Source:HGNC Symbol;Acc:HGNC:19143]
VRK3	Tumor	vaccinia related kinase 3 [Source:HGNC Symbol;Acc:HGNC:18996]
VWA5A	Tumor	von Willebrand factor A domain containing 5A [Source:HGNC Symbol;Acc:H
WDR74	Tumor	WD repeat domain 74 [Source:HGNC Symbol;Acc:HGNC:25529]
YIPF1	General	Yip1 domain family member 1 [Source:HGNC Symbol;Acc:HGNC:25231]
YOD1	Tumor	YOD1 deubiquitinase [Source:HGNC Symbol;Acc:HGNC:25035]
ZBTB37	Envir.	zinc finger and BTB domain containing 37 [Source:HGNC Symbol;Acc:HGNC:
ZC3H10	Envir.	zinc finger CCCH-type containing 10 [Source:HGNC Symbol;Acc:HGNC:25893
ZC3H3	Tumor	zinc finger CCCH-type containing 3 [Source:HGNC Symbol;Acc:HGNC:28972]
ZFP36	Envir.	ZFP36 ring finger protein [Source:HGNC Symbol;Acc:HGNC:12862]
ZFP36L1	Envir.	ZFP36 ring finger protein like 1 [Source:HGNC Symbol;Acc:HGNC:1107]
ZFP91	Envir.	ZFP91 zinc finger protein [Source:HGNC Symbol;Acc:HGNC:14983]
ZFP91-CN	General	ZFP91-CNTF readthrough (NMD candidate) [Source:HGNC Symbol;Acc:HGN
ZMYND8	Tumor	zinc finger MYND-type containing 8 [Source:HGNC Symbol;Acc:HGNC:9397]
ZNF217	Envir.	zinc finger protein 217 [Source:HGNC Symbol;Acc:HGNC:13009]
ZNF281	Envir.	zinc finger protein 281 [Source:HGNC Symbol;Acc:HGNC:13075]
ZNF460	Envir.	zinc finger protein 460 [Source:HGNC Symbol;Acc:HGNC:21628]
ZNF473	Tumor	zinc finger protein 473 [Source:HGNC Symbol;Acc:HGNC:23239]
ZNF595	Envir.	zinc finger protein 595 [Source:HGNC Symbol;Acc:HGNC:27196]
ZNF718	Envir.	zinc finger protein 718 [Source:HGNC Symbol;Acc:HGNC:26889]
ZNFX1	Envir.	zinc finger NFX1-type containing 1 [Source:HGNC Symbol;Acc:HGNC:29271]

Suppl. Table 6
Description of 386 utilized STAT3 targets.

NC:53]

C:24714]

ymbol;Acc:HGNC:16937]
Acc:HGNC:18984]
HGNC:23366]
17182]

NC:799]
HGNC:817]

FRC, CIITA, NACA, HSPCB, HSPCA, HIST1H4I, IL21R, POU2AF1, ARHH, EIF4A2, SFRS3
1046]

0204]

3460]

4351]

5]

5]

GNC:1917]

C:19083]
ymbol;Acc:HGNC:26108]

c:HGNC:18854]

;Acc:HGNC:28734]

HGNC:30288]

Symbol;Acc:HGNC:15877]

129]
249]

MN1, ABL2, FACL6, CHIC2, ARNT, JAK2, EVI1, CDX2, STL, HLXB9, MDS2, PER1, SYK, TTL, FGFR3, PA

HGNC:25282]
HGNC:26980]
:HGNC:23527]

HGNC:4095]

.78]

49]

ol;Acc:HGNC:9836]

34]
33]
29]
37]
57]
47]
52]
1]
6]

7]
1]
9]
0]
8]

4]
508]
38]
60]
GNC Symbol;Acc:HGNC:4921]
NC:5031]
C:5048]

cc:HGNC:28913]
208]

GNC:5261]
GNC:5269]

95]

HGNC:6110]
17967]

IGNC:21729]

mbol;Acc:HGNC:6261]

C:6924]

77]

nbol;Acc:HGNC:29665]
cc:HGNC:6853]

7]

7]

@, IGH@

0092]

:30815]

:7897]
HGNC:7962]
HGNC:7978]

]
C:8133]

HGNC:24118]

]
30029]
Acc:HGNC:29573]

bol;Acc:HGNC:29515]

GNC:14951]
c:HGNC:9309]

:17609]

709]

GNC Symbol;Acc:HGNC:17296]
BFA2T1, ETV6, LAF4

NC:10540]

687]
]

)92]

6333]

:HGNC:11362]
:HGNC:11363]

HGNC:11819]
NC:23696]

1853]
:26230]

16]

;]

GNC:6658]

:28365]
]

C:33441]
